# Practical and divergent electrochemical access to thiocarbamoyl fluorides and *N*-trifluoromethyl amines from secondary amines

**DOI:** 10.1038/s41467-026-74660-2

**Published:** 2026-06-20

**Authors:** Feba T. Pulikkottil, Daniel F. Carvalho, Kohei Taniguchi, John S. Burnett, Charles A. I. Goodall, Victoria K. Elmes, Andrew Hurt, Ewan R. Clark, Anthony J. H. M. Meijer, Kevin Lam

**Affiliations:** 1 School of Science, Faculty of Engineering and Science, University of Greenwich, Chatham Maritime, Kent ME4 4TB UK; 2 Department of Chemical Science and Engineering, School of Materials and Chemical Technology, Institute of Science Tokyo, Yokohama, Japan; 3 Chemistry, School of Mathematics and Physical Science, University of Sheffield, Brook Hill, Sheffield, S3 7HF UK

**Keywords:** Synthetic chemistry methodology, Electrocatalysis

## Abstract

We report a practical and divergent electrochemical method for converting simple secondary amines into thiocarbamoyl fluorides and *N*-trifluoromethyl amines. Using inexpensive, bench-stable reagents, including Et₃N·3HF and CS₂, these one-pot transformations proceed under mild, open-air conditions without solvent drying, degassing, inert atmosphere, or stoichiometric silver reagents. By tuning the reaction conditions, the same general strategy provides either product class in moderate to excellent yields across a broad substrate scope, with high functional-group tolerance and applicability to late-stage modification of complex bioactive molecules. The method is readily scalable and offers a practical alternative to existing fluorination approaches that rely on hazardous or costly reagents. Mechanistic studies combining cyclic voltammetry, ¹⁹F NMR spectroscopy, UV–Vis spectroscopy, HRMS, XRD, SEM–EDX, authentic intermediate analysis, and DFT calculations support a multistep pathway involving dithiocarbamate salts and thiuram intermediates, followed by bromide-assisted fluorination. This operationally simple, metal-free platform provides practical access to valuable fluorinated nitrogen-containing building blocks for synthesis, medicinal chemistry, and materials science.

## Abstract (web summary)

The authors report a practical and divergent electrochemical method for converting simple secondary amines into thiocarbamoyl fluorides and N-trifluoromethyl amines, proceeding under mild, open-air conditions without solvent drying, degassing, inert atmosphere, or stoichiometric silver reagents.

## Introduction

Despite fluorine being the 13th most abundant element in the Earth’s crust, naturally occurring organofluorine compounds are rare^1^. Over the past three decades, organofluorine chemistry has become a major area of research, driven by the growing demand for fluorinated molecules in pharmaceuticals, agrochemicals, and materials science. In drug discovery, fluorine substitution can profoundly influence physicochemical and biological properties, including lipophilicity, absorption, metabolic stability, and target binding^1,2^.

Among fluorinated functional groups, carbamoyl fluorides^3^, thiocarbamoyl fluorides^4^, and N-trifluoromethyl amines^5–12^ are particularly valuable motifs and synthetic building blocks. Carbamoyl and thiocarbamoyl fluorides represent a unique class of compounds that combine high stability and reactivity. In contrast to their unstable chloride analogues, their synthesis avoids the use of highly hazardous reagents such as thiophosgene and chlorine gas, offering a safer and more practical alternative. Furthermore, these fluorides exhibit broad functional group tolerance and demonstrate remarkable resistance to hydrolysis, even under aqueous and physiologically relevant conditions^4^. In parallel, landmark studies by Schoenebeck and co-workers have established elegant umpolung and SCF_3_-based approaches to *N*-trifluoromethyl amines, highlighting the growing importance of fluorine-nitrogen motifs in modern synthesis^5–11^. Together, these structural features have emerged as attractive targets for synthetic chemistry and medicinally relevant molecular design.

**Fig. 1:**
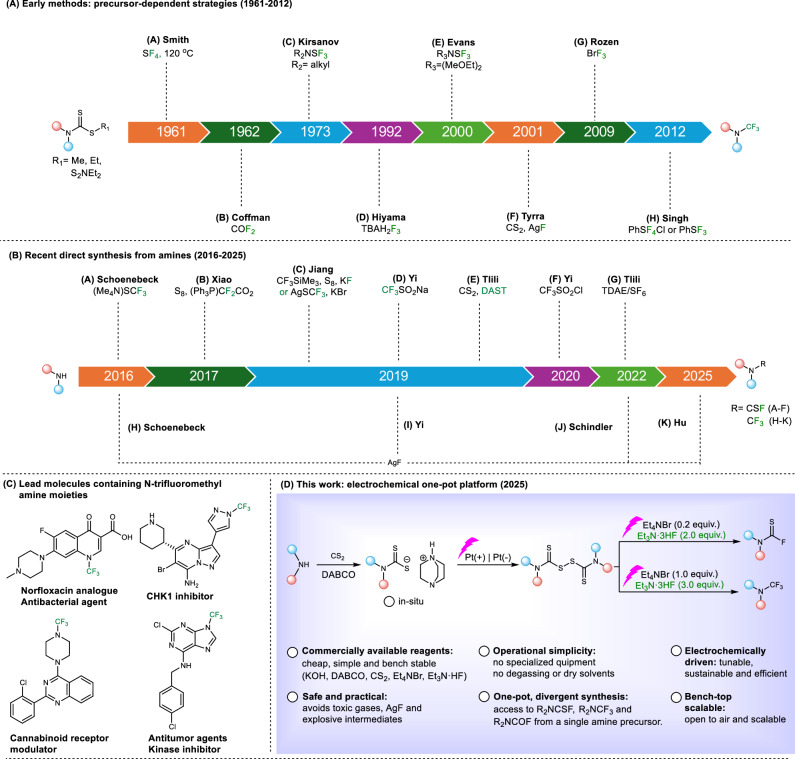
Historical and modern strategies for accessing thiocarbamoyl fluorides and N–CF₃ amines. **A** Early approaches (1961–2012) relied on hazardous fluorination reagents and precursor-based routes. **B** More recent direct-amine protocols (2016–2025) achieved important breakthroughs, notably via –SCF₃ reagents (Schoenebeck) and AgF-mediated desulfurisation–fluorination, but remained limited by reagent cost and scalability. **C** Lead molecules containing N-trifluoromethyl amine moieties. **D** This work introduces a practical electrochemical platform that enables divergent access to R₂NCSF and R₂NCF₃ from simple secondary amines under mild, scalable, and AgF-free conditions.

A range of strategies has been developed to access *N*-trifluoromethyl amines, including fluorine-halogen exchange, fluorodesulfurisation, and deoxyfluorination. Early methods relied on highly toxic and volatile reagents such as sulfur tetrafluoride^13,14^, antimony trifluoride^15^, and fluorophosgene^16^, and typically required harsh conditions, specialised equipment, and generated large quantities of hydrogen fluoride. Later approaches based on direct fluorination of thiocarbamates^17^ or dithiocarbamates with BrF_3_^18^, phenylsulfur fluorides^19^, or acidic HF-based systems^20–23^ represented important advances, but often required prefunctionalised substrates and excess reagents (Fig. 1A).

To address these limitations, one-pot desulfurisation-fluorination protocols have been developed that proceed through thiocarbamoyl fluoride intermediates. In many cases, in situ-generated thiofluorophosgene equivalents react with secondary amines to give R_1_R_2_NCSF, which can then be converted into *N*-trifluoromethyl amines using stoichiometric AgF. Representative approaches include the decomposition of Me_4_N·SCF_3_ salts^6,24^, the reduction of CF_3_SO_2_Na^25^, the trapping of difluorocarbene with sulfur, and activation of CS_2_ with sulfur-fluoride reagents^26,27^. Despite their synthetic utility, these methods still depend on unstable, hazardous, or expensive reagents and remain poorly suited to practical scale-up. Even bench-stable SCF_3_ reagents such as [Me_4_N][SCF_3_] are costly, while one-step CS_2_/AgF protocols still rely on stoichiometric AgF and generate substantial waste (Fig. 1B)^12,28^.

These limitations highlight the need for a more practical and sustainable route to thiocarbamoyl fluorides and *N*-trifluoromethyl amines from simple, bench-stable precursors. Such a goal is particularly relevant given the prominence of fluorine and nitrogen in bioactive molecules: by 2019, nearly 43% of FDA-approved small-molecule drugs contained at least one fluorine atom, while nitrogen is present in roughly 80% of pharmaceuticals and other biologically active compounds (Fig. 1C)^1,2,25,29,30^.

In this work, we report a practical and divergent electrochemical platform that converts simple secondary amines into either thiocarbamoyl fluorides or *N*-trifluoromethyl amines by tuning the reaction conditions (Fig. 1D). The method avoids stoichiometric silver salts and hazardous fluorination reagents and proceeds under mild conditions with an eco-scale score of approximately 70 (see Supplementary Information). This protocol uses only inexpensive, bench-stable reagents, including Et_3_N·3HF, CS_2_, and either KOH or DABCO, and does not require solvent drying, degassing, inert atmosphere or toxic gases. Unlike many HF-derived fluorination reagents, Et_3_N·3HF is a near-neutral liquid that is compatible with glass at room temperature, straightforward to handle, and enables controlled reactivity under mild aerobic conditions^31^. Combined with a favourable eco-scale score, this operational simplicity makes the method attractive for practical scale-up at an estimated reagent cost of approximately £4 per mmol of amine. The platform, therefore, offers a practical and versatile route to fluorinated nitrogen-containing building blocks of relevance to medicinal chemistry and materials science.

## Results

### Optimisation for thiocarbamoyl fluoride formation

We first optimised the electrochemical conditions for the direct synthesis of thiocarbamoyl fluorides **2** (Table 1). Guided by our previous work on thioisocyanates^32–34^, carbamoyl fluorides^3^, and fluorothioformates^35^, we selected Pt/Pt electrodes and generated dithiocarbamate salt **M1** in situ from the parent amine **1** and 2.0 equivalents of CS_2_. Under these conditions, electrolysis in ACN with 2.0 equivalents of Et_3_N·3HF at 11.11 mA cm^−2^ afforded R_2_NCSF in 22% yield (**entry 1**). Addition of KOH, intended to favour the formation of the corresponding potassium dithiocarbamate, reduced the yield to 13% (**entry 2**), whereas inclusion of catalytic Et_4_NBr, a known mediator in fluorodesulfurisation^31^, slightly improved the outcome to 23% (**entry 3**). Notably, combining KOH and Et_4_NBr produced a pronounced synergistic effect, increasing the yield to 78% (**entry 4**).

**Table 1:** Optimisation of *N,N*-dibenzyl thiocarbamoyl fluorides synthesis^c^


Entry	Deviation from the above conditions	Ratio (2a:3a)	Yield (%)^a, b^
1	Amine, CS_2_ and Et_3_N·3HF, no base	1:1.10	22^b^
2	With KOH	1:0.00	13^b^
3	Et_4_NBr, with no base	1:0.00	23^b^
4	KOH and Et_4_NBr	1:0.18	78^a^
5	Stainless steel as cathode	1:0.00	30^b^
6	Nickel as cathode	1:0.00	47^b^
7	Carbon as cathode	1:0.00	28^b^
8	Carbon as anode	1:0.06	53^b^
9	Carbon as cathode and anode	1:0.04	42^b^
10	ACN: DMSO (1:1) as solvent, 20 F mol^−1^	1:0.00	87^b^
11	15 mA	1:0.00	51^b^
12	25 mA	1:0.03	72^b^
13	1.0 eq Et_3_N·3HF	1:0.00	56^b^
14	3.0 equiv. Et_3_N·3HF	1:0.00	83^b^
15	0.5 equiv. Et_4_NBr	1:0.02	95^b^
16	1.0 equiv. Et_4_NBr	1:0.02	85^b^
17	0.5 equiv. DABCO	1:0.03	72^b^
18^[c]^	No deviation	1:0.00	91^a^
19	2.0 equiv. DABCO	1:0.16	95^b^
20	TBAF·3HF as F^-^ source	1:0.47	3^b^
21	NH_4_F as F^-^ source	1:0.24	9^b^
22	BF_3_OEt_2_ as F^-^ source	0:0	15^b^
23	CF_3_CO_2_Na as F^-^ source	0:0	n.d.
24	TMSCF_3_ as F^-^ source	0:0	n.d.
25	TBAH_2_F_3_ as F^-^ source	0:0	n.d
26	TMAF as F^-^ source	1:0	Traces
27	CsF as F^-^ source	0:0	n.d.
28	KF as F^-^ source	0:0	n.d.
29	No electricity	0:0	n.d.

^a^Isolated yield.

^b^Determined by ^1^H NMR, bromoform as internal standard.

^c^Dibenzylamine **1a** (39.5 mg, 0.2 mmol, 1.0 equiv.), DABCO (22.4 mg, 0.2 mmol, 1.0 equiv.), carbon disulfide [(**CS**_**2**_), 76.1 mg, 1.0 mmol, 5.0 equiv.], ACN (3.0 mL, 0.067 M), Et_4_NBr (8.4 mg, 0.04 mmol, 0.2 equiv.), Et_3_N·3HF (64.5 mg, 0.4 mmol, 2.0 equiv.).

Alternative electrode materials, including stainless steel, Ni, and carbon, proved less effective, confirming Pt/Pt as the preferred electrode combination (**entries 5**–**9**). Switching to a mixed ACN/DMSO solvent system suppressed over-fluorination of the thiocarbamoyl fluoride to the corresponding N-trifluoromethyl amine, although at the expense of a longer electrolysis time (20 F mol^−1^ versus 7 F mol^−1^, **entry 10**). Variation of the current density gave moderate to good yields across the range examined (**entries 11 and 12**). Screening the amount of Et_3_N·3HF showed that 2.0 equivalents provided a suitable balance between efficiency and reagent loading under the standard conditions (**entries 13, 14 and 18**). By contrast, increasing the amount of Et_4_NBr lowered the yield and selectivity, and led to greater side-product formation (**entries 15 and 16**). Replacing KOH with DABCO gave comparable, and in some cases improved, results (**entries 17**−**19**). Given its lower hygroscopicity, DABCO also offers a more practical and user-friendly base for small-scale use. Other fluorine sources were examined, but in most cases, no desired product was detected (**entries 20**−**28**). Finally, no product was formed in the absence of electrolysis, confirming that electricity is essential for the transformation.

### Optimisation for *N*-trifluoromethyl amine formation

Having established efficient conditions for thiocarbamoyl fluoride formation, we next optimised the further electrochemical conversion of these intermediates into *N*-trifluoromethyl amines **3** (Table 2). Initial experiments were conducted in ACN using 5.0 equivalents of Et₃N·3HF, a graphite anode, a platinum cathode, and electrolysis at 20 mA to 8 F mol^−1^. Under these conditions, the desired product was obtained in only 9% yield (**entry 1**). Guided by our earlier observations, inclusion of Et_4_NBr as an additive improved the yield to 28−30%, depending on the loading (**entries 2 and 3**). Replacing bromide with other halides proved detrimental: Et_4_NCl lowered the yield to 5%, whereas Et_4_NI was ineffective (**entries 4 and 5**). Variation of the cathode material, including Ni, graphite, and stainless steel, gave no improvement over platinum (**entries 6–8**), although increasing the current to 40 mA led to a modest increase in yield to 40% (**entry 9**).

**Table 2:** Optimisation of *N*-trifluoromethyl amines


Entry	Cathode (−)	I (mA)	X (F mol^−1^)	Et_4_NBr equiv.	yield (%)^a^
1	Pt	20	8	–	9
2	Pt	20	8	0.2	28
3	Pt	20	8	2	30
4^b^	Pt	20	8	2	5
5^c^	Pt	20	8	2	0
6	Ni	20	8	2	17
7	SS	20	8	2	5
8	Cgr	40	8	2	14
9^d^	Pt	40	8	2	40
10	Pt	20	20	1	74
11	Pt	20	30	1	81
12^d^	Pt	20	20	1	94
13^d, e^	Pt	20	20	1	95
14^d, e, f^	Pt	40	20	1	92

^a^^1^H NMR yield, bromoform as internal standard.

^b^Et_4_NCl instead of Et_4_NBr.

^c^Et_4_NI instead of Et_4_NBr.

^d^Pt as anode (+).

^e^3.0 equiv. of Et_3_N·3HF.

^f^Dibenzylamine **1a** (39.5 mg, 0.2 mmol, 1.0 equiv.), KOH (11.2 mg, 0.2 mmol, 1.0 equiv.), carbon disulfide [(CS_2_), 76.1 mg, 1.0 mmol, 5.0 equiv.], ACN [3.0 mL, 0.067 M)], Et_4_NBr (42.0 mg, 0.2 mmol, 1.0 equiv.), Et_3_N·3HF (96.7 mg, 0.6 mmol, 3.0 equiv.).

A much greater improvement was achieved by increasing the total charge passed, which raised the yield to 74% at 20 F mol^−1^ and 81% at 30 F mol^−1^ (**entries 10 and 11**). Replacing the graphite anode with platinum proved decisive, affording the *N*-trifluoromethyl amine in 94% yield at 20 F mol^−1^ when Pt/Pt electrodes were used (**entry 12**). Further optimisation showed that the loading of Et_3_N·3HF could be reduced to 3.0 equivalents and the current increased without compromising reaction efficiency, thereby shortening the electrolysis time (**entries 13 and 14**). The final conditions therefore employed Pt/Pt electrodes, 3.0 equivalents of Et_3_N·3HF, 1.0 equivalent each of KOH and Et_4_NBr, a current of 40 mA, and a total charge of 20 F mol^−1^, delivering the desired *N*-trifluoromethyl amine in 92% yield (**entry 14**). Full optimisation details are provided in the Supplementary Information.

### Substrate scope for thiocarbamoyl fluorides (R₂NCSF)

**Fig. 2:**
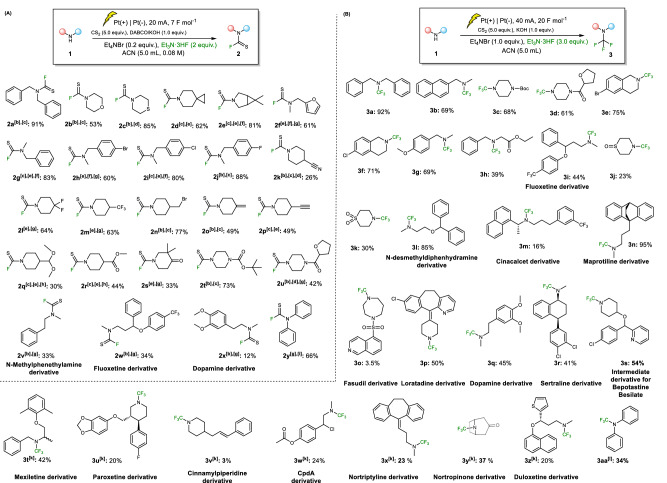
Substrate scope of the electrochemical fluorination platform. **A** *N*,*N*-dialkyl thiocarbamoyl fluorides. **B** *N*-trifluoromethyl amines. [a] Amine (0.4 mmol, 1.0 equiv.), DABCO (0.4 mmol, 1.0 equiv.), carbon disulfide [(CS₂), 2.0 mmol, 5.0 equiv.], ACN (5.0 mL, 0.067 M), Et_4_NBr (0.08 mmol, 0.2 equiv.), Et_3_N·3HF (0.8 mmol, 2.0 equiv.). [b] KOH instead of DABCO. [c] Silica pad filtration. [d] 10 mA. [e] DABCO. [f] 9 F mol^−1^. [g] Column purification. [h] 6 F mol^−1^. [i] Obtained from ethyl diphenylcarbamodithioate (**1aa**) following general procedure **1A**. [j] Amine (0.4 mmol, 1.0 equiv.), KOH (0.4 mmol, 1.0 equiv.), carbon disulfide [(CS₂), 2.0 mmol, 5.0 equiv.], ACN (5 mL, 0.067 M), Et_4_NBr (0.4 mmol, 1.0 equiv.), Et_3_N·3HF (1.2 mmol, 3.0 equiv.). [k] qNMR yield. [l] Obtained from ethyl diphenylcarbamodithioate (**1aa**) following general procedure **2A**.

With the optimised conditions in hand, we first evaluated the scope of the thiocarbamoyl fluoride synthesis (Fig. 2A). A broad range of functionalised secondary amines proved compatible, including both aliphatic and heterocyclic substrates. The standard substrate **2a** was obtained in 78% isolated yield using KOH, whereas replacing KOH with DABCO increased the yield to 91%. Cyclic amines of medicinal relevance, such as morpholine **2b** and thiomorpholine **2c**, were smoothly converted into the corresponding products in 53 and 85% yield, respectively. Spirocyclic scaffolds **2d** and **2e**, which are valued for their three-dimensionality in drug design, were also well tolerated and afforded the corresponding thiocarbamoyl fluorides in 62−81% yield. A heteroaromatic furan derivative **2f** gave 61% yield, whereas benzyl amines, both unsubstituted **2g** and halogen-substituted **2h**−**j**, delivered the desired products in 60–88% yield.

Electronic effects influenced the reaction outcome. A nitrile-substituted cyclic amine **2k** gave only 26% yield, whereas other electron-withdrawing substituents, including –CF_2_R **2l** and –CF_3_ **2m**, were better tolerated and afforded the corresponding products in 64 and 63% yield, respectively. Halogen-substituted cyclic amines were also compatible, as illustrated by **2n**, which was obtained in 77% yield. Notably, substrates bearing alkene **2o** and alkyne **2p** groups reacted cleanly to give the desired products in moderate yields, without detectable competitive fluorination of the unsaturated moieties. Other functional groups, including ethers **2q**, esters **2r**, and ketones **2s**, were likewise tolerated, although with more modest efficiency. Protected amines also proved compatible: a Boc-protected substrate **2t** gave 73% yield, whereas the corresponding amide-protected analogue **2u** was obtained in 42% yield. Drug-derived amines **2v**−**x** were less effective substrates, furnishing the corresponding thiocarbamoyl fluorides in moderate to low yields, consistent with a greater tendency towards overoxidation and decomposition to carbamoyl fluoride derivatives. Because aromatic amines are less nucleophilic towards CS_2_ and form dithiocarbamate salts less readily, the aryl-substituted product **2y** was instead prepared from the corresponding dithiocarbamate ester precursor **1y** in 66% yield (See Supplementary Information). Overall, these results highlight the broad functional-group tolerance of the thiocarbamoyl fluoride-forming protocol and its potential for late-stage diversification of medicinally relevant scaffolds.

### Substrate scope for *N*-trifluoromethyl amines

The results are summarised in Fig. 2B. Under the optimised electrochemical conditions, a broad range of secondary amines were converted into the corresponding *N*-trifluoromethyl derivatives in moderate to excellent yields. Functional-group tolerance was generally high. The standard substrate **3a** and the naphthyl analogue **3b** were obtained in good and excellent yield, respectively. A notable advantage of using Et_3_N·3HF as the fluorine source is its compatibility with acid-sensitive protecting groups. Unlike more acidic fluorination reagents, it did not promote Boc deprotection, allowing the Boc-protected substrate **3c** to be isolated in 68% yield^26^. Similarly, the amide-protected substrate **3d** furnished the desired product in 61% yield. Substituted benzylic amines bearing electron-withdrawing halogens **3e** and **3f** or an electron-donating methoxy group **3g** performed particularly well, affording the corresponding products in 69−75% yield. By contrast, the ester-substituted benzyl glycine derivative **3h** was obtained in only 39% yield, likely owing to reduced product stability.

Several potentially bioactive scaffolds also proved compatible with the reaction conditions. Electrolysis of fluoxetine delivered the corresponding N-trifluoromethyl analogue **3i** in 44% yield, while thiomorpholine oxide **3j** and thiomorpholine dioxide **3k**, motifs relevant to antibiotic chemistry, were obtained in 23% and 30% yield, respectively. A further series of complex drug-derived amines also underwent efficient *N*-trifluoromethylation, affording products **3l–s** in moderate to excellent yields, further highlighting the breadth of the method. Because some *N*-trifluoromethyl amines decomposed rapidly on silica gel, thereby limiting chromatographic purification^36^, compounds **3t**−**z** were characterised by NMR yield. Diarylamines as substrates proved more challenging because their low nucleophilicity renders addition to CS_2_ reversible, leading to instability of the corresponding dithiocarbamate salt. To circumvent this problem, the intermediate was trapped by alkylation with EtBr as the corresponding S-ethyl dithiocarbamate ester **1aa**^37^, from which the aryl-substituted product **3aa** was obtained in 34% yield. S-ethyl diphenylcarbamothioate **2y’** was formed as the major by-product under these conditions (see Supplementary General Procedure 2A). Overall, these results demonstrate the broad applicability of the method and highlight its potential for late-stage modification of drug-like molecules.

### Scale-up reactions

**Fig. 3:**
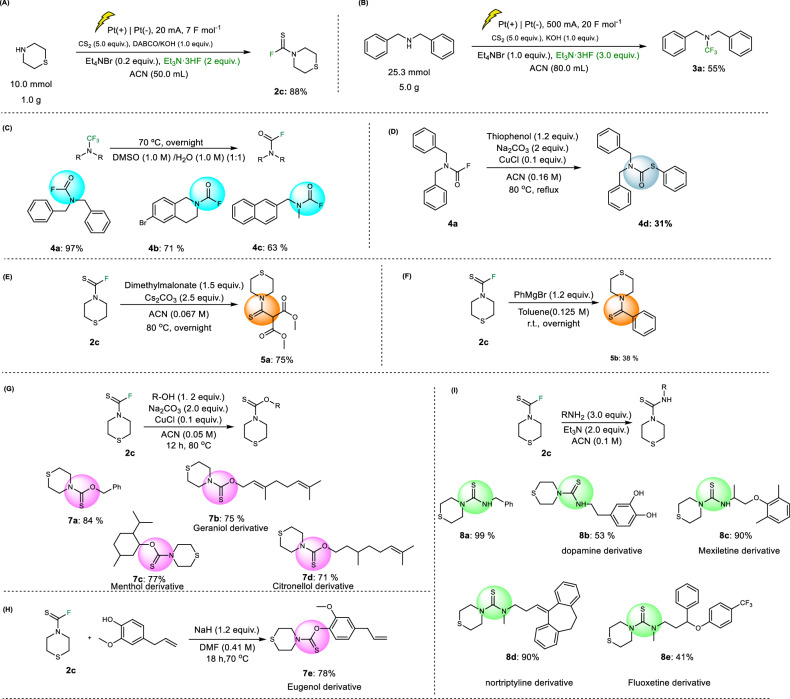
Scale-up reactions and applications of thiocarbamoyl fluorides and *N*-trifluoromethyl amines. **A** 1.0 g scale reaction of R_2_NCSF synthesis; **B** 5.0 g scale reaction of R_2_N-CF_3_ synthesis; **C** R_2_NCOF synthesis and scope; **D** Thiocarbamate synthesis from R_2_NCOF; **E** Reaction of R_2_NCSF with dimethyl malonate; **F** Reaction of R_2_NCSF with Grignard reagent; **G**, **H** reaction of R_2_NCSF with alcohols; **I** reaction of R_2_NCSF with amines.

To demonstrate the practicality of the method, we carried out gram-scale electrolysis under the optimised conditions. Thiomorpholine was converted on a 10.0 mmol (1.0 g) scale into the corresponding thiocarbamoyl fluoride **2c** in 88% isolated yield (Fig. 3A), with no substantial loss in efficiency relative to the small-scale reaction. Likewise, dibenzylamine **1a** was electrolysed on a 5.07 mmol (1.0 g) scale to furnish *N*-trifluoromethyl product **3a** in excellent yield (see Supplementary scale-up section). The crude reaction mixture could then be directly subjected to hydrolysis in DMSO/H₂O (1:1) at 70 °C overnight to give carbamoyl fluoride **4a** in 97% yield without chromatographic purification (see Supplementary applications section). Further scale-up to 25.3 mmol (5.0 g) was also successful, although with diminished yield (Fig. 3B), likely owing to the longer electrolysis time and partial decomposition of the *N*-trifluoromethyl intermediate during prolonged reaction times. This limitation should be alleviated in setups equipped with larger electrode surface areas, which would permit higher current and shorter electrolysis times. Nevertheless, these experiments demonstrate that multigram quantities of *N*-trifluoromethyl products can be obtained even with standard small-electrode configurations. Overall, the scale-up studies highlight the robustness and operational simplicity of the protocol and support its suitability for larger-scale applications under mild conditions.

### Applications

Having established the scope of the *N,N*-dialkyl thiocarbamoyl fluoride and *N*-trifluoromethyl amine products, we next explored their value in downstream transformations (Fig. 3C, I). In particular, the thiocarbamoyl fluorides proved to be versatile intermediates that could be further elaborated through reaction with suitable nucleophiles. Guided by previous reports^36^, we found that the in situ generated *N*-trifluoromethyl amines could be converted directly into carbamoyl fluorides **4a**−**c** in excellent yields, thereby providing access to these products from simple amines without the need for multistep sequences via oxamic acids^3^. The resulting carbamoyl fluoride **4a** could then be further transformed with thiophenol to give S-phenyl dibenzylcarbamothioate **4d**.

Further diversification of the thiocarbamoyl fluorides was also demonstrated. Thiomorpholine-4-carbothioyl fluoride **2c** reacted with dimethyl malonate to afford dimethyl 2-(thiomorpholine-4-carbonothioyl)malonate **5a** in 75% yield, and with a Grignard reagent to give phenyl(thiomorpholino)methanethione **5b** in 38% yield. Reactions with naturally occurring alcohols furnished fluorinated derivatives of geraniol, menthol, citronellol, and eugenol in excellent yields **7a**−**e**. Finally, thiourea formation was demonstrated by coupling thiomorpholine-4-carbothioyl fluoride **2c** with bioactive amines such as dopamine, mexiletine, nortriptyline, and fluoxetine, affording the corresponding products in excellent yields **8a**−**e**.

### Mechanistic studies

**Fig. 4:**
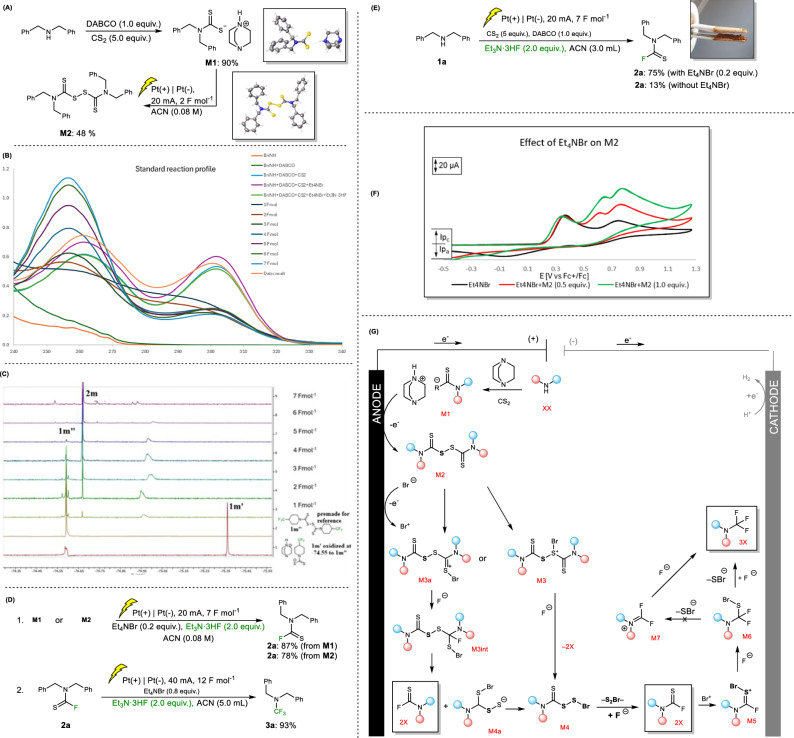
Control experiments and plausible mechanism. **A** X-ray crystallography of **M1** and **M2**; **B** UV−Vis experiments; **C** ^19^F NMR experiments; **D1** Synthesis of N−CSF (**2a**) starting from **M1** and **M2 and** **D2** formation of **3a** from **2a**; **E** Experimental Et_4_NBr control reaction; **F** Cyclic voltammetry studies of Et_4_NBr (−), Et_4_NBr + M2 (0.5 equiv.) (−), and Et_4_NBr + M2 (1.0 equiv.) (−) vs. Fc^+^/Fc; **G** Plausible mechanism.

The transformation appears to proceed through a multistep sequence involving consecutive redox and nucleophilic events (Fig. 4). To probe this mechanism, we combined electrochemical methods with DFT calculations, spectroscopic and analytical techniques, including cyclic voltammetry (CV), ^19^F NMR spectroscopy, UV−Vis spectroscopy, HRMS, single-crystal and powder XRD, and SEM–EDX. For these studies, 4-(trifluoromethyl)piperidine **1m** was used as the model substrate for ^19^F NMR experiments, whereas dibenzylamine **1a** was used for UV−Vis analysis. In addition, representative dithiocarbamate salts **M1** and the corresponding thiuram intermediates **M2** were independently prepared and fully characterised, providing authentic reference materials for the intermediates observed during electrolysis (Fig. 4A).

Crystallisation of the dibenzylamine-derived salt [Bn_2_N−CS_2_^-^][DABCO−H⁺] **M1** from dichloromethane instead of THF/hexane (1:1) solvent mixture unexpectedly afforded crystals of thiuram derivative **M10**, identified as N-Bn_2_N−CS_2_−CH_2_−S_2_C−NBn_2_ (see Supplementary crystallographic data). This observation indicates that these dithiocarbamate salts can slowly react with dichloromethane, and therefore that chlorinated solvents should be avoided in this chemistry.

### Formation and behaviour of the dithiocarbamate salt M1

Treatment of dibenzylamine with CS_2_ and DABCO gave rise to two new UV absorptions at 260 and 302 nm (Fig. 4B), matching those of authentic [Bn_2_N−CS_2_^-^][DABCO−H⁺] **M1** (Supplementary Fig. 5). HRMS analysis further supported formation of this species by confirming its elemental composition (Supplementary Fig. 7). In ^19^F NMR experiments, treatment of 4-(trifluoromethyl)piperidine **1m** with CS_2_ and DABCO shifted the fluorine resonance from −75.38 to −75.35 ppm, consistent with formation of the corresponding dithiocarbamate **1m’** (Supplementary Fig. 12b). Unlike the dibenzylamine-derived salt **M1**, which proved relatively stable, the corresponding piperidine derivative **1m′** was only transiently stable and gradually oxidised in the NMR tube. Within one hour, a new ^19^F NMR signal appeared at −74.59 ppm, corresponding to thiuram species **1m″** (Fig. 4C). These observations suggest that spontaneous oxidation of the dithiocarbamate to the thiuram can occur in air, likely mediated by dissolved oxygen.

Electrolysis of isolated [Bn_2_N−CS_2_^−^][DABCO−H⁺] **M1** under the standard conditions afforded the corresponding thiocarbamoyl fluoride in 87% yield (Fig. 4D1). Likewise, when isolated thiocarbamoyl fluoride **2a** was subjected to the standard electrochemical conditions for *N*-trifluoromethyl amine formation, product **3a** was obtained in 93% yield (Fig. 4D2). Together, these experiments support the intermediacy of both dithiocarbamate and thiocarbamoyl fluoride species in the overall reaction pathway.

### Anodic oxidation and dimerisation

CV experiments clarified the anodic events. Bromide was oxidised at *E*_pox_ = 0.27 V *versus* Fc⁺/Fc⁰ (Supplementary Fig. 14), whereas dithiocarbamate [Bn_2_N−CS_2_^−^][DABCO−H⁺] **M1** exhibited a chemically irreversible oxidation at *E*_pox_ = −0.23 V *versus* Fc⁺/Fc⁰ (Supplementary Fig. 15), indicating that **M1** is oxidised preferentially. Formation of the corresponding [Bn₂N−CS₂•] radical was further supported by trapping experiments with TEMPO (Supplementary Fig. 18). A second anodic wave appeared at *E*_pox_ = 0.75 V *versus* Fc⁺/Fc⁰, matching the oxidation potential of independently prepared thiuram **M2** and supporting the assignment of **M2** as the dimerisation product formed after initial oxidation of **M1** (Supplementary Fig. 16). Consistent with this proposal, ^19^F NMR monitoring of electrolysis aliquots showed rapid conversion of **1m′** into **1m″** at the outset of the reaction (Fig. 4C), and bromide had no discernible effect on this initial oxidation step. When electrolysis of dibenzylamine **1a** was interrupted after 2 F mol^−1^, thiuram **M2** was isolated in 48% yield and fully characterised by NMR spectroscopy and XRD (Fig. 4A). Electrolysis of independently prepared **M2** under the standard fluorination conditions then furnished the corresponding thiocarbamoyl fluoride in 78% yield, supporting its role as a key intermediate in the process (Fig. 4D1).

### Role of bromide and fluorination step

A catalytic amount of Et_4_NBr markedly accelerated the conversion of thiuram **M2** into the corresponding thiocarbamoyl fluoride. Although the product could still be obtained in the absence of bromide, the yield was substantially lower and a higher charge was required, with concomitant formation of numerous by-products (Supplementary Fig. 19). Bromide proved even more important for the subsequent formation of the *N*-trifluoromethyl amine, which was obtained in only 13% yield from dibenzylamine **1a** under bromide-free conditions (Fig. 4E).

CV data showed that bromide (*E*_pox_ = 0.27 V *versus* Fc⁺/Fc⁰) is oxidised at lower potential than thiuram **M2** (*E*_pox_ = 0.75 V *versus* Fc⁺/Fc⁰) (Supplementary Figs. 14 and 16). Although literature precedent suggests that the Br^−^/Br⁺ redox couple can act as an electrochemical mediator^31^, CV titration of Et_4_NBr with increasing concentrations of **M2** led only to a shift of the bromide oxidation wave to lower potential, without catalytic current enhancement, arguing against a classical electron-transfer mediation pathway (Fig. 4F). Instead, these observations are consistent with direct chemical reaction between thiuram **M2** and electrogenerated bromonium species (Br⁺), leading to bromosulfonium intermediates such as **M3** or **M3a** (Fig. 4G). Subsequent reaction with fluoride is proposed to furnish the thiocarbamoyl fluoride R_2_NCSF (**2X**) together with transient brominated intermediates, which undergo further rearrangement and fragmentation. In this way, the proposed sequence allows one equivalent of thiuram to give rise to two equivalents of thiocarbamoyl fluoride, while bromide is released and reoxidised at the anode, sustaining a catalytic bromine cycle. As electrolysis proceeds, UV–Vis, HRMS, and ^19^F NMR analyses reveal a progressive decrease in **M2** and a corresponding increase in thiocarbamoyl fluoride concentration (Supplementary Figs. 6, 8–10, and 12b). Attempts to isolate intermediates **M3**−**M7** were unsuccessful, presumably owing to their inherent instability, although uncoordinated **M5** was detected by HRMS (Supplementary Fig. 10).

### Continuation to *N*-trifluoromethyl amines

Under extended electrolysis, the same bromide-mediated manifold appears to promote further activation of the thiocarbamoyl fluoride en route to the *N*-trifluoromethyl amine (Fig. 4G). In this proposal, anodically regenerated bromide is reoxidised to bromonium at the anode, after which activation of the carbon centre in R_2_NCSF enables nucleophilic attack by fluoride to furnish the *N*-trifluoromethyl amine **3X**. This stepwise bromide-assisted fluorination is consistent with earlier reports in which thiuram derivatives react with chlorine- or bromine-based reagents to give N = CX₂ and N−CX₃ products^38,39^. Further monitoring by ^19^F NMR and HRMS supported sequential consumption of thiuram **M2**, accumulation of thiocarbamoyl fluoride, and eventual formation of the *N*-trifluoromethyl amine under prolonged electrolysis conditions (Supplementary Figs. 9, 11, and 13).

### Sulfur balance and by-products

SEM–EDX and powder XRD analyses of the insoluble solids formed during electrolysis revealed the presence of sulfate (SO₄²^−^) and elemental sulfur (S₈), indicating that sulfur-containing fragments released during the reaction undergo further oxidation at the anode (Supplementary Figs. 21–24).

Collectively, these observations support a mechanistic sequence involving: (i) formation of dithiocarbamate salt **M1**; (ii) anodic oxidation to thiuram **M2**; (iii) bromonium-assisted fluorination and cleavage to form thiocarbamoyl fluoride R_2_NCSF **2X**; and (iv) further bromide-mediated activation of R_2_NCSF en route to *N*-trifluoromethyl amine **3X**. Together, the combination of authentic intermediates, electrochemical analysis, spectroscopy, and product evolution studies provides a coherent picture of this multistep transformation (Fig. 4G).

### DFT calculations

**Fig. 5:**
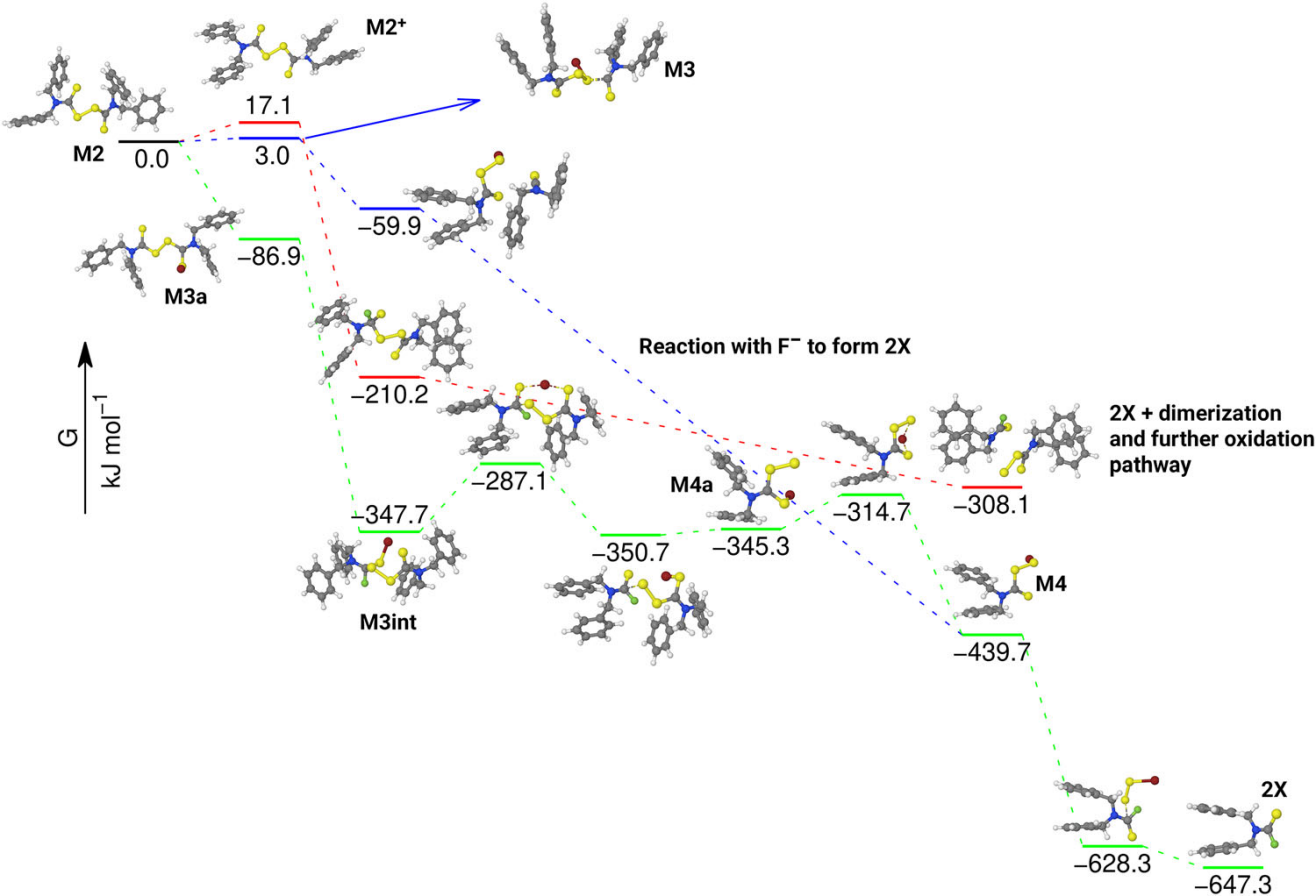
Computational investigations. Gibbs energy profile showing the formation of **2X** from **M2** via three distinct pathways: Red trace: oxidation of **M2** and subsequent reaction with F^–^. Blue trace: Oxidation of Br^–^ to Br^+^ and reaction with **M2** via attack on the perthio (C–S–S–C) bridge. Green trace: Oxidation of Br^–^ to Br^+^ and reaction with **M2** via attack on the thioketone (C = S) group. (F = fluorine, int = intermediate, TS = transition state, diss = dissociation).

**Fig. 6:**
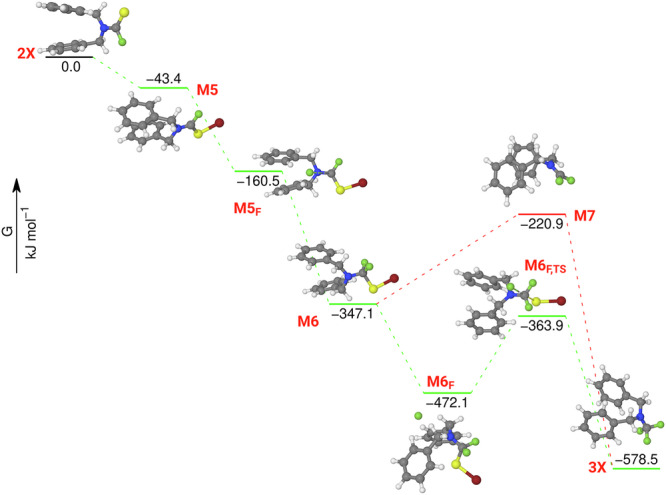
Computational investigations. Gibbs energy profile showing the formation of **3X** from **2X** via two distinct pathways: Red trace: Oxidation of Br^–^ to Br^+^ and reaction with **2X**. Formation of **3X** via SBr^–^ dissociation. Green trace: Oxidation of Br^–^ to Br^+^ and reaction with **2X**. Formation of **3X** via concerted association of F^–^ and dissociation of SBr^–^.

To further interrogate the proposed mechanism, we performed density functional theory calculations, as outlined in the supplementary computational details section. Given that the initial stages of the reaction, culminating in isolable thiuram intermediate **M2**, are strongly supported by experiment, the calculations were directed towards the later stages of the process, namely the conversion of **M2** into thiocarbamoyl fluoride **2X** and the subsequent formation of *N*-trifluoromethyl amine **3X** from **2X**. The computed free-energy profiles are presented in Figs. 5 and 6, and the overall mechanistic proposal is summarised in Fig. 4G.

Figure 5 summarises the computed pathways for the conversion of **M2** into R_2_NCSF **2X**. Three possible scenarios were considered: direct oxidation of **M2** followed by fluoride attack (red trace), or oxidation of Br^−^ to Br⁺^40,41^ followed by reaction of Br⁺ with **M2** either at the thioketone sulfur atom (green trace, **M3a**) or at the disulfide bridge (blue trace, **M3**). As described in the supplementary computational details, oxidation of Br^−^ was balanced by reduction of two equivalents of FeCp_2_⁺, whereas oxidation of **M2** was balanced by reduction of one equivalent of FeCp_2_⁺, thereby maintaining an internally consistent electron count for comparison of the computed pathways.

Among the three possibilities, oxidation of Br⁻ followed by reaction of Br⁺ at the C = S group of **M2** was calculated to be the most favourable initial step. By contrast, the direct oxidation pathway, although formally viable, would generate **2X**, together with a disulfide radical fragment, after fluoride addition and dissociation of the intermediate **M2F**. This route (red trace) is therefore less consistent with the experimental requirement for bromide and was not pursued further. The remaining bromide-mediated pathways were examined in greater detail.

In the blue pathway, Br⁺ reacts with one of the sulfur atoms of the S−S bridge to give intermediate **M3**. Although formation of **M3** is endoergic, it is followed by fragmentation to a tightly associated complex, **M3diss**, which then reacts readily with F^−^ to give **2X** and **M4**. In the green pathway, the reaction of Br⁺ with the thioketone sulfur atom is exoergic and furnishes **M3a**. Subsequent fluoride attack gives **M3int**, which rearranges through bromine migration to form **M4adiss** and then dissociates to **M4a** and **2X**. Further isomerisation of **M4a** to **M4**, followed by fluoride attack, affords a second equivalent of **2X** together with S_2_Br^−^. The elongated S−Br bond in S_2_Br^−^ suggests that it should dissociate readily into Br^−^ and S_2_, with the latter expected to oligomerise to elemental sulfur (S_8_), in agreement with the experimental elemental analysis.

Figure 6 shows the corresponding calculations for the conversion of **2X** into *N*-trifluoromethyl amine **3X**. In this case, the calculations again support initial oxidation of Br^−^ to Br⁺, followed by reaction with **2X** to give **M5**. Fluoride then associates with **M5** to form a weakly bound complex, **M5F**, before passage through a submerged barrier to generate **M6**. From **M6**, two mechanistic possibilities were considered. In the first, dissociation of SBr^−^ occurs before fluoride attack, generating **M7** en route to **3X**. In the second, fluoride first coordinates to form **M6F**, after which C−F bond formation and SBr^−^ departure occur in a concerted step. The latter pathway is markedly lower in free energy and is therefore the preferred mechanism for the formation of **3X**.

Overall, the calculations support the bromide-mediated mechanism proposed in Fig. 4G and are consistent with the experimental observations. In particular, they identify bromide oxidation followed by chemical reaction with sulfur-containing intermediates as more favourable than direct oxidation of **M2**. The calculated free-energy profiles further suggest that the overall transformation is thermodynamically favourable.

In summary, we report a practical and operationally simple one-pot electrochemical method for the divergent conversion of secondary amines into thiocarbamoyl fluorides and *N*-trifluoromethyl amines, using readily available, bench-stable, and near-neutral Et_3_N·3HF as the fluoride source. Under the reaction conditions, secondary amines are converted in situ into dithiocarbamate intermediates, which undergo anodic oxidation to thiuram species before furnishing either thiocarbamoyl fluorides ( ≈ 4 h, 7 F mol^−1^) or, upon extended electrolysis, *N*-trifluoromethyl amines ( ≈ 11.4 h, 20 F mol^−1^). The method proceeds under mild conditions, requires minimal purification for many substrates, and displays broad functional-group tolerance, including carbonyls, alkenes, alkynes, nitriles, heterocycles, protected amines, and pharmaceutical derivatives. Mechanistic studies support a pathway involving dithiocarbamate and thiuram intermediates, followed by bromide-assisted fluorination. Overall, this work provides practical electrochemical access to valuable fluorinated nitrogen-containing building blocks and highlights the potential of this strategy for late-stage functionalization in medicinal chemistry and materials science.

## Methods

### General procedure for thiocarbamoyl fluoride synthesis

Amine **1** (0.4 mmol, 1.0 equiv.), DABCO (45.0 mg, 0.4 mmol, 1.0 equiv.) or KOH (22.0 mg, 0.4 mmol, 1.0 equiv.), and CS_2_ (152 mg, 2.0 mmol, 5.0 equiv.) were added to a 5 mL one-compartment ElectraSyn 2.0 vial to generate the dithiocarbamate salt in situ. Acetonitrile (ACN, 5 mL, 0.08 M), Et_4_NBr (16.8 mg, 0.08 mmol, 0.2 equiv.), and Et_3_N·3HF (120 μL, 0.8 mmol, 2.0 equiv.) were then added. The resulting solution was electrolysed at a current density of 11.11 mA cm^−2^ to 7 F mol^−1^ (3.75 h) using a platinum anode (WE) and platinum cathode (CE). Reaction progress was monitored by HPLC. After complete consumption of the starting amine, the reaction mixture was quenched with saturated aqueous NaHCO_3_ and extracted with DCM. The combined organic layers were dried over Na_2_SO_4_, and the solvent was removed under reduced pressure. The crude residue was filtered through silica and, where necessary, purified by flash column chromatography.

### General procedure for *N*-trifluoromethyl amine synthesis

Amine **1** (0.4 mmol, 1.0 equiv.), KOH (22.4 mg, 0.4 mmol, 1.0 equiv.), and CS_2_ (152 mg, 2.0 mmol, 5.0 equiv.) were added to a 5 mL one-compartment ElectraSyn vial to generate the dithiocarbamate salt in situ. Acetonitrile (ACN, 5 mL, 0.08 M), Et_4_NBr (84.0 mg, 0.4 mmol, 1.0 equiv.), and Et_3_N·3HF (196 μL, 1.2 mmol, 3.0 equiv.) were then added. The solution was electrolysed at a current density of 22.2 mA cm^−2^ to 20 F mol^−1^, unless otherwise stated, using a platinum anode (WE) and platinum cathode (CE). Reaction progress was monitored by ^19^F NMR until complete consumption of the N-CSF intermediate. The solvent was then removed under reduced pressure, and the residue was diluted with hexane and filtered through Celite to afford the final product.

Full optimisation, substrate-scope, experimental, and characterisation data, including NMR, FTIR, UV, CV, HRMS, elemental analysis, SEM-EDX, and X-ray data, are provided in the Supplementary Information.

## Supplementary information

Supplementary information

Transparent Peer Review file

## Data Availability

The data supporting the findings of this study are available within the paper, the Supplementary Information and the Source Data file provided with this article. The X-ray crystallographic data generated in this study have been deposited at the Cambridge Crystallographic Data Centre (CCDC) under deposition numbers CCDC 2497049 (M1), CCDC 2497050 (M2), and CCDC 2497048 (M10). These data can be obtained free of charge from the Cambridge Crystallographic Data Centre via https://www.ccdc.cam.ac.uk/structures/ or https://www.ccdc.cam.ac.uk/data_request/cif. All other supporting data, including experimental procedures, compound characterisation data, and optimised geometries, are provided in the Supplementary Information. Data supporting the findings of this manuscript are also available from the corresponding author upon request.
