# Supplementary material for: Practical and divergent electrochemical access to thiocarbamoyl fluorides and N-trifluoromethyl amines from secondary amines
Source: Nat Commun. 2026 Jun 20;17:7762. doi: 10.1038/s41467-026-74660-2 (PMC13434417; doi:10.1038/s41467-026-74660-2)
Supplement: Supplementary file 1 [file 41467_2026_74660_MOESM1_ESM.pdf]

# Practical and Divergent Electrochemical Access to Thiocarbamoyl Fluorides and *N*-Trifluoromethyl Amines from Secondary Amines

Feba T. Pulikkottil,[a] Daniel F. Carvalho,[a] Kohei Taniguchi,[b] John S. Burnett,[a] Charles A. I. Goodall,[a] Victoria K. Elmes,[a] Andrew Hurt,[a] Ewan R. Clark,[a] Anthony J. H. M. Meijer[c] and Kevin Lam*[a]

[a] School of Science, Faculty of Engineering and Science, University of Greenwich, Chatham Maritime, Chatham, Kent, ME4 4TB, U.K.

[b] Department of Chemical Science and Engineering, School of Materials and Chemical Technology, Institute of Science Tokyo, 4259 Nagatsuta-cho, Midori-ku, Yokohama 226-8501, Japan

[c] Chemistry, School of Mathematics and Physical Science, University of Sheffield, Brook Hill, Sheffield, UK

*K.Lam@greenwich.ac.uk

## Supplementary Contents

Material and methods……………………………………………………………... 002
Batch Electrochemical Reaction Setup………………………………………………… 005
Optimisation tables……………………………………………………………………... 006
Electrochemical preparation of thiocarbamoyl fluorides………………………………. 009
Electrochemical preparation of trifluoromethyl amines………………………………. 020
Large scale reactions…………………………………………………………………… 033
Applications of thiocarbamoyl fluorides and trifluoromethyl………............................ 034
Mechanistic Studies…………………………………………………………………. 044
Unsuccessful substrates ……..………………………………………………………… 065
NMR Spectra …………………………………………………………………………... 067
Bibliography……………………………………………………………………………. 157

**Supplementary material and methods**
**General Experimental Procedures**
All reactions were carried out under aerobic conditions unless otherwise stated. All solvents and commercially available reagents were purchased from standard vendors and used without further purification unless otherwise stated. Electrolysis was performed using an IKA Electrasyn 2.0 using platinum (**Pt**) working electrode (**WE**) and counter electrode (**CE**) (distance between the electrodes = 6 mm) using a stirring rate 1200 rpm with a stir bar of size 10 x 3 mm. Analytical thin-layer chromatography (TLC) was performed using silica gel plates (0.25 mm thickness) on aluminium support. Visualization was accomplished by irradiation with a UV lamp and/or staining with either $KMnO_4$ stain. Column chromatography was performed over Silica gel 60 Å (40-63μ mesh) using a CombiFlash $R_f$ Lumen automatic flash chromatography system. Residual solvent was removed using a static oil pump (< 10 mbar). The cooling of reaction mixtures was achieved using an ice bath (0 °C).

NMR spectra were obtained using a JEOL ECZR 400 ($^1$H 399.78 MHz; $^{19}$F 376.17 MHz; $^{13}$C 100.53 MHz) or ECA 500 ($^1$H 500.16 MHz; $^{13}$C 125.77 MHz) spectrometer and are reported relative to the residual solvent resonances. All heteronuclear NMR spectra were $^1$H decoupled and recorded at room temperature unless otherwise stated. Data for $^1$H NMR spectra are reported as follows: chemical shift (δ, ppm), coupling constant (Hz), multiplicity (s, singlet; d, doublet; t, triplet; m, multiplet; br, broad) and integration. Data for $^{13}$C and $^{19}$F NMR are reported in terms of chemical shift (δ, ppm). IR spectra were recorded on a Perkin Elmer Spectrum Two instrument as neat samples or in chloroform solvent.

High Resolution Mass Spectrometry (HRMS) data were obtained by Dr. Iain Goodall of the University of Greenwich Mass Spectrometry Service using a Waters Synapt G2 hybrid Quadrupole-orthogonal acceleration time-of-flight configuration (Waters, Manchester, UK) operating in Resolution Mode ($M/\Delta M \geq 18{,}000$), fitted with a Waters Acquity UPLC binary solvent chromatographic pump system. The column used was a reversed-phase Acquity BEH C18 2.1 x 50 mm, 1.7-micron bead, running a 3- minute separation with an A:B eluent mixture comprising of either deionised water with 0.1% (v:v) formic acid and acetonitrile with 0.1% (v:v) formic acid (negative mode) respectively or deionised water with 0.1% (v:v) ammonium hydroxide and acetonitrile with 0.1% (v:v) ammonium hydroxide (positive mode) respectively. Mass calibration of the instrument was performed using sodium formate cluster ions, and an orthogonal Lock-SprayTM ESI probe was used with a lock mass calibrant, leucine-enkephalin. The pseudomolecular leucineenkephalin ion at *m/z* = 554.2615 (Negative Ion Mode), and *m/z* = 556.2771 (Positive Ion Mode), was used as the internal mass correction calibrant. Additional samples were analyzed on a Thermo LTQ Orbitrap XL coupled with a heated electrospray source (HESI). The capillary temperature was set to 275 °C and a voltage of 21 V. The sheath gas and auxiliary gas flow were set to 10 and 5 L $h^{-1}$ respectively and the source current and voltage set to 100 μA and 5 kV. A solution of analyte (0.1 mg/ml) and sodium formate (1% v/v) in acetonitrile was added by direct infusion (10 μL/min) into the mass spectrometer using a Hamilton syringe (250 μL).

Gas-Chromatography Mass Spectrometry (GC-MS) data were obtained using a Shimadzu Nexis GC-2030 gas chromatograph connected to a GCMS-QP2020 NX gas chromatograph mass spectrometer, equipped with an AOC-20i Plus auto injector. The column was a CD-5MS capillary column (30 m x 0.25 mm x 0.25 μm), with helium as the carrier gas. The sample injection volume was 1 μL, and separations run over a 5-minute period with an increasing oven temperature (gradient) between 40 – 280 °C. Results were visualised and processed using LabSolutions GCMS solution version 4.50.

High-Performance Liquid Chromatography-Mass Spectrometry (HPLC-MS) data were obtained using a Shimadzu LC-2050C 3D coupled with a Shimadzu LCMS-2020 FCV-20AH2. The column was an Ascentis Express 90Å AQ-C18, 2.7 μm. Results were visualised and processed using LabSolutions GCMS solution version 5.114.
Accurate mass and isotope-profile fitting were performed with MassWorks (Shimadzu GCMSsolution with MassWorks add-on). Profile export was enabled so that CRN profile data were written for each run. Mass calibration used perfluorotributylamine (PFTBA) acquired and processed according to the Shimadzu MassWorks Quick Guide. A calibration file was generated in MassWorks using the vendor PFTBA standard-ion table, and spectral accuracy was then applied to sample spectra using that calibration. Detector voltage was set to avoid PFTBA peak saturation as recommended, and auto-tuning was performed before calibration. Elemental-composition candidates were evaluated with CLIPS using restricted element sets appropriate to the samples, with DBE limits and empirical rules adjusted as needed. Reported formula assignments are supported by MassWorks spectral-accuracy fits to the isotope envelope together with accurate-mass agreement.
Profile export: GCMS solution "Profile raw data additionally stored: CRN format." Calibration: PFTBA acquired per the guide, then used to create a MassWorks calibration file; peaks verified on the Review tab before saving.
Acquisition caution: ensure PFTBA ions are not saturated; the guide recommends tuning and adjusting detector voltage so m/z 69 is roughly in the 3.0–4.0 × $10^6$ count range before measurements.
Analysis: CLIPS searches run on the monoisotopic ion of interest with sensible element ranges; isotope-pattern fits and RMSE from the calibration report were inspected alongside accurate mass.[1]

Cyclic voltammetry studies were carried out using an Autolab 302N potentiostat interfaced through Nova 2.1 software to a personal computer. Electrochemical measurements were performed in a glovebox under an atmosphere of dinitrogen with oxygen and water levels of less than 5 ppm at 298 K, with solvents that had been thoroughly degassed and purified by passing through an alumina-based purification system. Sample concentrations of 1.0 mM were used, alongside 0.1 M $[nBu_4N][PF_6]$ supporting electrolyte concentrations. Experiments were conducted using a standard three-electrode setup comprising of a glassy carbon disc working electrode, platinum wire counter electrode, and AgCl-coated silver wire as a pseudo-reference electrode. Potentials are reported relative to the $[FeCp_2]^{+/0}$ redox couple, obtained through the addition of ferrocene to the analyte solution. The glassy carbon surface of the working electrode was prepared by successive polishings with Buehler diamond paste interspersed with washings (acetone and water) and sonications and final drying under ante-chamber vacuum.

All the UV measurements were recorded on Agilent Cary 100 UV-Vis Spectrophotometer at room temperature, using the Agilent Bio 100 software. The data were measured between 200 and 800 nm with two laser beams at 25 °C, using Hellma analytics high performance quartz glass possessing a path length of 10 mm.

XRD data was collected on a D8 Advance X-ray Diffractometer (Bruker, Germany) in theta-theta geometry in reflection mode using Cu Kα radiation at 40 kV and 40 mA. Data collection was between 2-80° 2θ, step size of 0.02° and a counting time of 0.3 seconds per step.

Data was collected using DIFFRAC plus XRD Commander version 2.6.1 software (Bruker-AXS). Quantitative analysis was performed with TOPAS version 5 (Bruker-AXS).

SEM-EDS samples were attached to an aluminium sample stub using conductive carbon tabs (Lab Tech EM). Samples were analysed on a Zeiss Sigma 300 (Cambridge, UK) field emission gun SEM operated at an accelerating voltage of 20 kV in low vacuum mode. Samples were imaged uncoated. EDX data was collected using an Oxford Instruments X-Max 50 EDX detector (Abingdon, UK).

Single-crystal XRD data were collected on a Rigaku Oxford Diffraction XtaLAB Mini II single-crystal diffractometer using Mo Kα radiation. Crystals were mounted on a MiTeGen loop and data were collected at 293 K. The structures were solved using Olex2 with the ShelXT structure-solution program by intrinsic phasing and refined with the ShelXL refinement package by least-squares minimization. All hydrogen atoms were refined using a riding model. Sample-specific crystallographic details are provided in the CIF files and in Supplementary Table 4.[3]
Deposition Number(s) 2497049 (for **M1**), 2497050 (for **M2**), 2497048 (for **M10**) contain the supplementary crystallographic data for this paper, including structure factors and refinement instructions, and can be obtained free of charge from The Cambridge Crystallographic Data Centre, 12 Union Road, Cambridge CB2 1EZ, UK (email: depos-it@ccdc.cam.ac.uk), or via https://www.ccdc.cam.ac.uk/getstructures.

**Computational details**

Density functional theory (DFT) calculations were performed using Gaussian16, version C.01.[4] Gaussian was compiled with Gaussian-supplied versions of BLAS and ATLAS.[5-6] The PW6B95D3(BJ) functional[7] was used throughout this study. In a benchmark study,[8] this functional was found to perform well in the calculation of barrier heights, in particular. The def2-SVP basis set[9] was used throughout with the ultrafine setting for the integrals. All calculations performed on these systems included bulk solvent effects through the IEFPCM algorithm[10-11] as implemented in Gaussian 16 using the parameters for MeCN. Minima were confirmed through the absence of any imaginary frequencies. All transition state calculations were performed either through QST2 or QST3 calculations.[12-13] Intrinsic reaction coordinate (IRC)[14-16[IRC]] calculations were performed with updated frequencies at every 3rd step of the profile to check the validity of the transition states, which were identified by a single imaginary frequency. All energies reported in the figures of this paper are Gibbs energies, evaluated at 298K and 1 bar. All oxidation steps have been counter-balanced by corresponding reductions of $FeCp_2^+$, commensurate with the total number of electrons involved, calculated using exactly the same protocol as outlined above. Within this protocol the oxidation of 2 $Br^-$ to $Br_2$ occurs at 0.69 V, in reasonable agreement with the literature. The ESI for the calculations was created by using in-house developed software based on the OpenEye toolkit.[17] Images of molecules were created using Jmol (version 16.1.45)[18] and POV-Ray (version 3.7).[19]

**Supplementary batch electrochemical reaction setup**

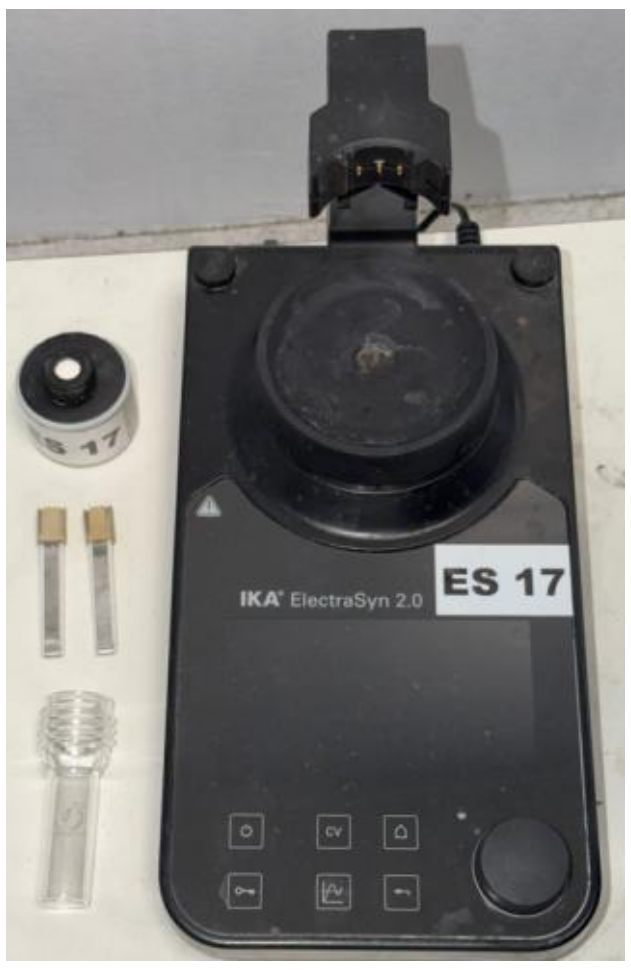


**Supplementary Fig. 1**: Electrochemical set up

**Supplementary eco scale factor calculations**

1. Yield (91%)
   - Yield points = 91 ÷ 2 = 45.5.
   - Yield penalty = 50 − 45.5 = 4.5.
2. Price / availability
   - Platinum electrodes: reused >100×, so per-run cost/impact is very small. Therefore penalty = 3.
3. Safety / environmental
   - $CS_2$ (toxic, flammable) remains a major hazard. $Et_3N \cdot 3HF$ is less corrosive at neutral pH. Therefore penalty = 12.
4. Technical setup (electrochemistry) = 6.
5. Energy / time (electrolysis 7 F $mol^{-1}$) = 3.
6. Work-up & purification (silica pad filtration + rotavap) = 2.

Therefore, total penalty = 4.5 (yield) +3 (price) +12 (safety) +6 (tech) +3 (energy) +2 (workup) = 30.5

EcoScale = 100 − 30.5 = 69.5 → ≈ 70.

**Supplementary Faraday efficiency**

Faradaic efficiency of the reaction is calculated as shown below.[20]

$$\mathrm{n} = \frac{\mathrm{Qtheo}}{Qexp} \times 100$$

$$Qtheo = z_p \times N_p \times F = z \times N \times Y \times F$$

$$Qexp = I \times t = z \times N \times F \times equiv$$

$$n = \frac{Y}{equiv}$$

n: Faradaic efficiency in percent [%], Qtheo: theoretical charge in Coulomb [C], Qexp: experimental charge in Coulomb [C], equiv: electron equivalents, $z_p$: Number of electrons per product [−], $N_p$: Number of moles of the product [mol], Y: yield in percent [%], F: Faraday constant [96485 C $mol^{-1}$], I: current [A], t: reaction time [s].

In the plausible mechanistic pathway, 5 equivalents of electrons are theoretically required to give 1 mmol of thiocarbamoyl fluoride (**2a**) and *N*-trifluoromethylamines (**3a**) products. Hence, the yield presented was defined as the yield of 2a ($Y_{2a}$ = 91%) and 3a ($Y_{3a}$ = 92%).

Therefore, for thiocarbamoyl fluoride reaction

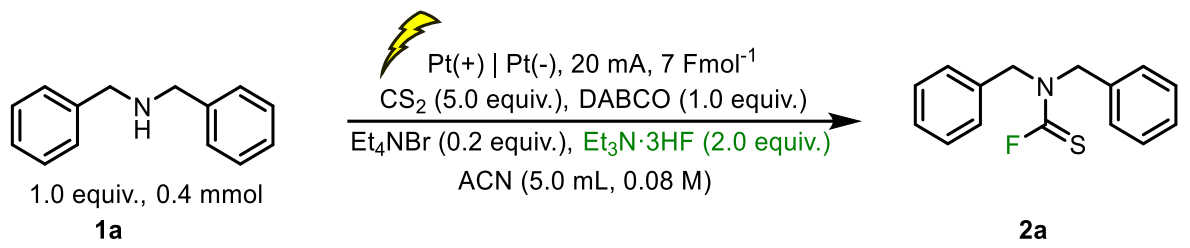


$$n = \frac{Y_{2a}}{equiv}$$

$$n = \frac{5}{7} \times 91\% = 65\%$$

For N−τrifluoromethylamine reaction

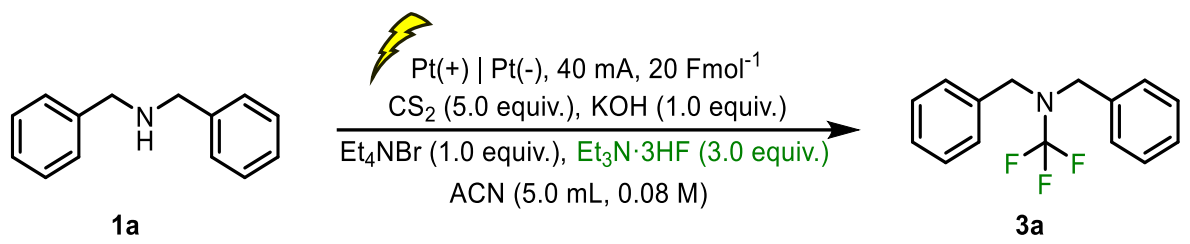


$$n = \frac{Y_{3a}}{equiv}$$

$$n = \frac{5}{20} \times 92\% = 23\%$$

**Supplementary Table 1: Optimization of the electrochemical thiocarbamoyl fluorination**

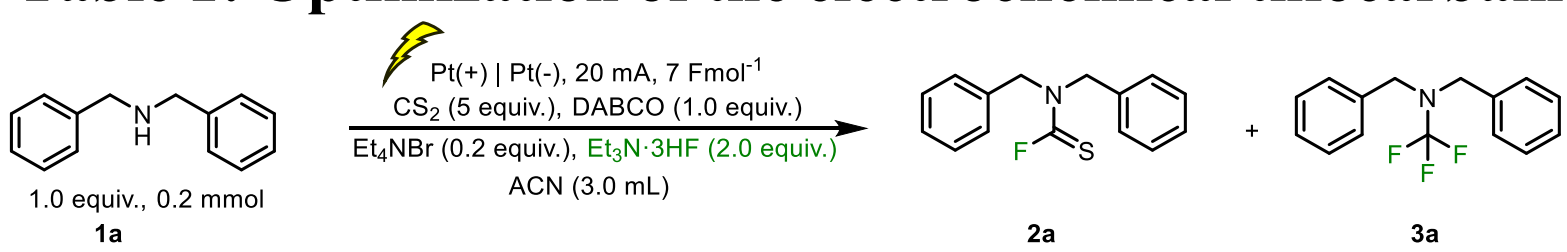


| entry | deviation from above | ratio (2a:3a) | yield (%)[a, b] |
|---|---|---|---|
| **1** | none | 1:0.00 | 91[a] |
| **2** | stainless steel as cathode | 1:0.00 | 30[b] |
| **3** | nickel as cathode | 1:0.00 | 47[b] |
| **4** | carbon as cathode | 1:0.00 | 28[b] |
| **5** | carbon as anode | 1:0.06 | 53[b] |
| **6** | carbon as cathode and anode | 1:0.04 | 42[b] |

| | | | |
|---|---|---|---|
| **7** | THF as solvent, 11 F $mol^{-1}$ | 1:0.55 | 0.7 [b] |
| **8** | DME as solvent, 11 F $mol^{-1}$ | 1:0.48 | 3.0 [b] |
| **9** | DMF as solvent, 20 F $mol^{-1}$ | 1:0.07 | 67 [a] |
| **10** | DMSO as solvent, 20 F $mol^{-1}$ | 1:0.00 | 89 [a] |
| **11** | ACN:DMSO (1:1) as solvent, 20 F $mol^{-1}$ | 1:0.00 | 87 [a] |
| **12** | 0.03 M, 8 F $mol^{-1}$ | 1:0.31 | 89 [a] |
| **13** | 0.13 M, 11 F $mol^{-1}$ | 1:1.38 | 92 [a] |
| **14** | NaOH (1 equiv.) instead of DABCO | 1:3.82 | 79 [a] |
| **15** | KOH (1 equiv.) instead of DABCO | 1:0.18 | 78 [a] |
| **16** | TBAF·3HF as $F^-$ source | 1:0.47 | 3.0 [b] |
| **17** | With LiBr, 6 F $mol^{-1}$ | 1:0.14 | 64 [a] |
| **18** | With MgBr, 6 F $mol^{-1}$ | 1:0.06 | 48 [a] |
| **19** | With $Ca(OTf)_2$, 6 F $mol^{-1}$ | 1:31.7 | 0.1 [b] |
| **20** | With $CaI_2$ (1eq), 20 F $mol^{-1}$ (not completed) | 1:0.06 | 8.5 [b] |
| **21** | With $CaI_2$ (0.2 equiv.), 17 F $mol^{-1}$ | 1:2.13 | 3.8 [b] |
| **22** | Without DABCO | 1:0.00 | 13 [b] |
| **23** | 15 mA | 1:0.00 | 51 [b] |
| **24** | 25 mA | 1:0.03 | 72 [b] |
| **25** | 1.0 equiv. $Et_3N$·3HF | 1:0.00 | 56 [b] |
| **26** | 3.0 equiv. $Et_3N$·3HF | 1:0.00 | 83 [b] |
| **27** | 0.5 equiv. $Et_4NBr$ | 1:0.02 | 95 [b] |
| **28** | 1.0 equiv $Et_4NBr$ | 1: 0.02 | 85 [b] |
| **29** | 0.5 equiv. DABCO | 1: 0.03 | 72 [b] |
| **30** | 2.0 equiv. DABCO | 1: 0.16 | 95 [b] |
| **31** | $NH_4F$ as $F^-$ source | 1:0.24 | 9.0[b] |
| **32** | $BF_3OEt_2$ as $F^-$ source | 0:0 | 15 [b] |
| **33** | $CF_3CO_2Na$ as $F^-$ source | 0:0 | n.d. |
| **34** | $TMSCF_3$ as $F^-$ source | 0:0 | n.d. |
| **35** | $TBAH_2F_3$ as $F^-$ source | 0:0 | n.d |
| **36** | TMAF as $F^-$ source | 1:0 | traces |
| **37** | CsF as $F^-$ source | 0:0 | n.d. |
| **38** | KF as $F^-$ source | 0:0 | n.d. |
| **39** | No electricity | 0:0 | n.d. |

[a)]Isolated yield, [b)]Determined by $^1$H NMR. [c)] Dibenzylamine **1a** (39.5 mg, 0.2 mmol, 1.0 equiv.), DABCO (22.4 mg, 0.2 mmol, 1.0 equiv.), carbon disulfide [($CS_2$), 76.1 mg, 1.0 mmol, 5.0 equiv.], acetonitrile [(ACN) 3.0 mL, 0.067 M)], $Et_4NBr$ (8.4 mg, 0.04 mmol, 0.2 equiv.), $Et_3N$·3HF (64.5 mg, 0.4 mmol, 2.0 equiv.).

**Supplementary Table 2: Optimization of the electrochemical N-trifluoromethylation[a]**

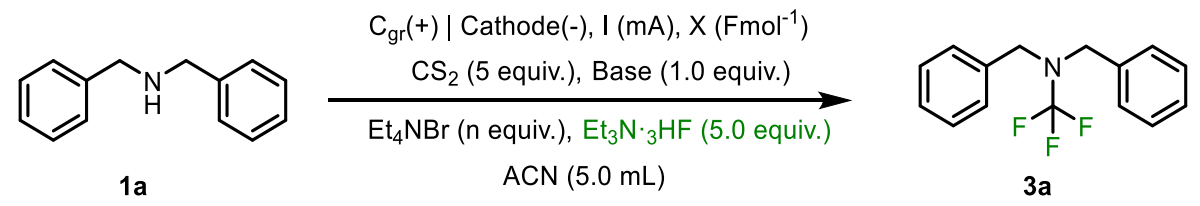


| Entry | Cathode (-) | *I* (mA) | C (F mol$^{-1}$) | Et4NBr equiv. | yield (%)[o] |
|---|---|---|---|---|---|
| **1** | Pt | 20 | 8 | -- | 9 |
| **2** | Pt | 20 | 8 | 0.2 | 28 |
| **3** | Pt | 20 | 8 | 2 | 30 |
| **4[a)]** | Pt | 20 | 8 | 2 | 16 |
| **5[b)]** | Pt | 20 | 8 | 2 | 0 |
| **6[c)]** | Pt | 20 | 8 | 2 | 0 |
| **7** | Ni | 20 | 8 | 2 | 17 |
| **8** | SS | 20 | 8 | 2 | 5 |
| **9[d)]** | Pt | 20 | 8 | 2 | 12 |
| **10[e)]** | Pt | 20 | 8 | 2 | 4 |
| **11[f)]** | Pt | 20 | 8 | 2 | 5 |
| **12[g)]** | Pt | 20 | 8 | 2 | 0 |
| **13[h)]** | Pt | 20 | 8 | 2 | 29 |
| **14** | Pt | 40 | 8 | 2 | 40 |
| **15** | Pt | 50 | 8 | 2 | 39 |
| **16** | Pt | 60 | 8 | 2 | 31 |
| **17** | Pt | 80 | 8 | 2 | 22 |
| **18** | Cgr | 40 | 8 | 2 | 14 |
| **19[i)]** | Pt | 40 | 8 | 2 | 29 |
| **20** | Pt | 40 | 8 | 0.2 | 30 |
| **21[j)]** | Pt | 40 | 8 | 2 | 32 |
| **22** | Pt | 40 | 8 | -- | 28 |
| **23** | Pt | 40 | 8 | 0.5 | 44 |
| **24** | Pt | 40 | 8 | 1 | 43 |
| **25** | Pt | 40 | 8 | 3 | 56 |
| **26** | Pt | 40 | 8 | 4 | 52 |
| **27** | Pt | 40 | 8 | 5 | 45 |
| **28[k)]** | Pt | 40 | 8 | 0.5 | 3 |
| **29[l)]** | Pt | 40 | 8 | 0.5 | 30 |
| **30[m)]** | Pt | 40 | 20 | 1 | 65 |
| **31[m)]** | Pt | 40 | 30 | 1 | 64 |
| **32[m)]** | Pt | 20 | 20 | 1 | 74 |
| **33[m)]** | Pt | 20 | 30 | 1 | 81 |
| **34[m)]** | Pt | 20 | 25 | 1 | 72 |
| **35[m)]** | Pt | 40 | 25 | 1 | 60 |
| **36[m)]** | Pt | 20 | 35 | 1 | 70 |
| **37[m)]** | Pt | 10 | 30 | 1 | 54 |
| **38[m)]** | Pt | 20 | 30 | -- | 31 |
| **39[m)]** | Pt | 20 | 30 | 0.5 | 42 |
| **40[m)]** | Pt | 20 | 30 | 1 | 69 |
| **41[m)]** | Pt | 20 | 30 | 2 | 91 |
| **42[i), m)]** | Pt | 20 | 20 | 1 | 94 |

| | | | | | |
|---|---|---|---|---|---|
| **43**[i), m), n)] | Pt | 20 | 20 | 1 | 95 |
| **44**[i), m), n)] | Pt | 40 | 20 | 1 | 92 |

[a)]DCM as solvent, [b)]DMF as solvent, [c)]HFIP as solvent ,[d)]DME as solvent, [e)]$MeNO_2$ as solvent, [f)]$Et_4NCl$ instead of $Et_4NBr$, [g)]$Et_4NI$ instead of $Et_4NBr$, [h)]10 equiv. of $Et_3N{\cdot}3HF$, [i)]Pt as anode (+), [j)]2.5 equiv. of $Et_3N{\cdot}3HF$, [k)]using 5 equiv. of DBU as base, [l)]$Bu_4N{\cdot}H_2F_3$ instead of $Et_3N{\cdot}3HF$, [m)]using 1 equiv. of KOH as base, [n)]3 equiv. of $Et_3N{\cdot}3HF$, [o)]NMR yield, bromoform as internal standard.

**Supplementary Table 3: Optimization of carbamoyl fluoride**[a21-22]

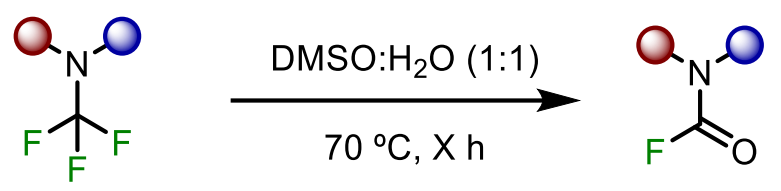


3 4

| Entry | $H_2O$ | DMSO | time | temperature | 6X:3X[b] |
|---|---|---|---|---|---|
| 1 | 4 M | - | 30 min | r.t. | 1.0:43.4 |
| 2 | 1 M | - | 12 h | r.t. | 1.0:11.2 |
| 3 | 0.4 M | 0.4 M | 4 h | 50 ºC | 1.0:0.2 |
| 4[c] | 0.4 M | 0.4 M | 12 h | 70 ºC | 1.0:0.0 |

[a)]0.4 mmol, DMSO: $H_2O$, (1:1, 50 mL), 70 ºC, 12 h. [b)]$^{19}F$ NMR ratio, [c)]1.0 g scale (5.08 mmol).

**Supplementary electrochemical preparation of N,N-dialkylthiocarbamoyl fluorides**

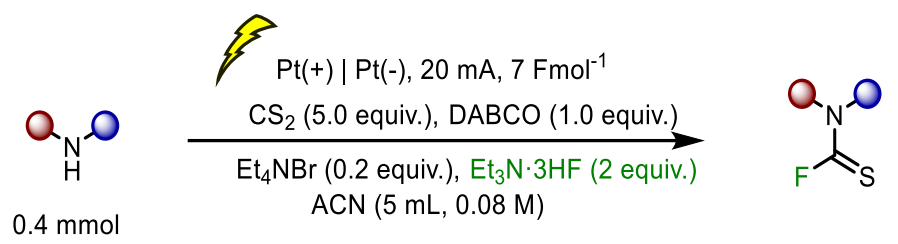


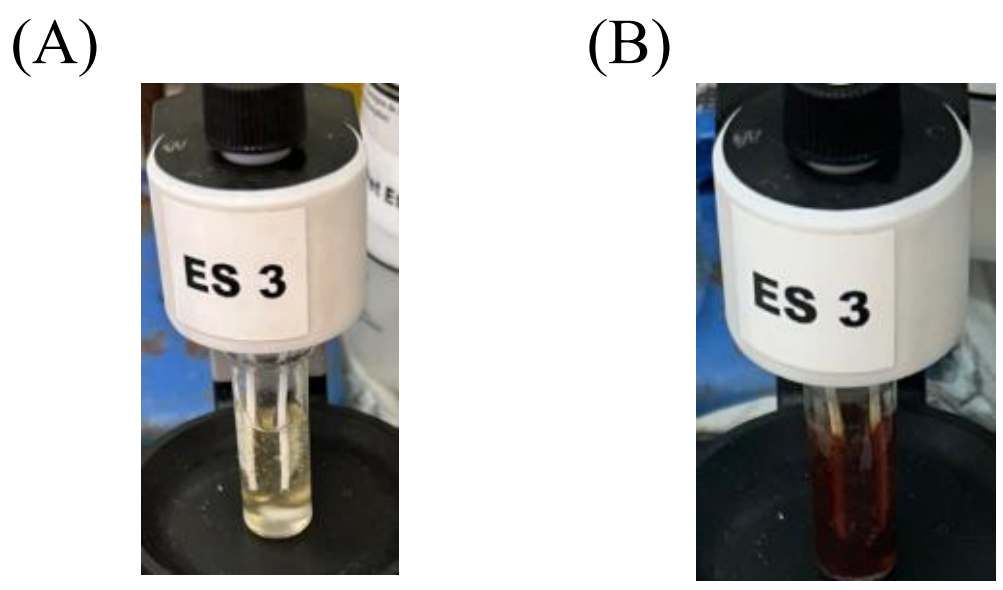


**Supplementary Fig. 2:** Colour change observed during the reaction of *N,N*-dialkylthiocarbamoyl fluorides: **(A)** before the reaction and **(B)** after the reaction.

**General procedure 1:** Amine (**1**, 0.4 mmol, 1.0 equiv.), DABCO (45.0 mg, 0.4 mmol, 1.0 equiv.) or KOH (22.0 mg, 0.4 mmol, 1.0 equiv.), $CS_2$ (152 mg, 2.0 mmol, 5.0 equiv.) was added into a 5 mL one compartment Electrasyn vial to generate in situ salt. Then acetonitrile (ACN) (5 mL, 0.08 M), $Et_4NBr$ (16.8 mg, 0.08 mmol, 0.2 equiv.) and $Et_3N{\cdot}3HF$ (120 µL, 0.8 mmol, 2.0 equiv.) was added. The solution was then electrolyzed at 11.11 mA $cm^{-2}$ for 7 F $mol^{-1}$ (i.e., 3.75 h) using a platinum anode (WE) and platinum cathode (CE). The reaction was monitored by HPLC. After full consumption of the starting amine, the reaction mixture was quenched with a saturated aqueous solution of $NaHCO_3$, extracted with DCM. The organic layers are then combined and dried with $Na_2SO_4$. The solvent was removed under reduced pressure. The

crude mixture was filtered through silica and purified via flash column chromatography if necessary.

**Supplementary scope of *N,N*-dialkylthiocarbamoyl fluorides**

dibenzylcarbamothioic fluoride (**2a)**

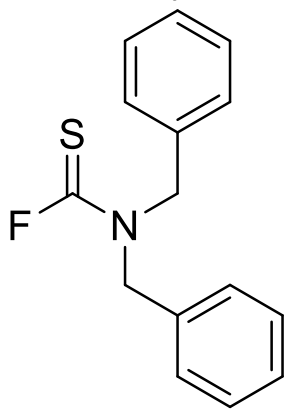

**2a**

Dibenzylcarbamothioic fluoride **2a** was obtained following the general procedure 1, using KOH as base and by silica pad filtration as a dark brown liquid (86 mg, 0.332 mmol, 83%).

$^1$H NMR (400 MHz, $CDCl_3$) δ 7.44 – 7.24 (m, 8H), 7.20 (d, *J* = 7.4 Hz, 2H), 4.96 (s, 2H), 4.58 (s, 2H).
$^{13}$C NMR (101 MHz, $CDCl_3$) δ 182.5 (d, *J* = 321.3 Hz), 134.1, 134.0, 129.2, 129.0, 128.5, 128.3, 127.8, 55.6 (d, *J* = 5.8 Hz), 51.2 (d, *J* = 5.8 Hz).
$^{19}$F NMR (376 MHz, $CDCl_3$) δ 15.64.
IR (cm$^{-1}$) ν = 3030, 2925, 2854, 1514, 1442, 1355, 1232, 1174, 1079, 1029, 938, 697, 601, 540, 454
HRMS (ESI) m/z: [M+H]$^+$ calcd for $C_{15}H_{15}FNS$ 260.0909, found 260.0919.

Morpholine-4-carbothioyl fluoride (**2b)**

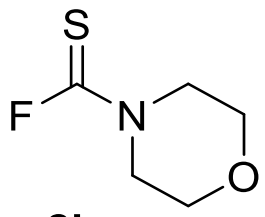

**2b**

Morpholine-4-carbothioyl fluoride **2b** was obtained following the general procedure 1 using KOH as base and by silica pad filtration as a yellow liquid (31.6 mg, 0.212 mmol, 53%).

$^1$H NMR (400 MHz, $CDCl_3$) δ 4.02 – 3.95 (m, 2H), 3.84 – 3.77 (m, 2H), 3.77 – 3.67 (m, 4H).
$^{13}$C NMR (101 MHz, $CDCl_3$) δ 180.3 (d, *J* = 320.3 Hz), 65.9, 50.5 (d, *J* = 6.3 Hz), 47.1 (d, *J* = 5.3 Hz).
$^{19}$F NMR (376 MHz, $CDCl_3$) δ 13.00.
IR (cm$^{-1}$) ν = 2923, 2855, 1737, 1516, 1444, 1278, 1113, 1029, 951, 846, 730, 677, 585, 462
HRMS (ESI) m/z: [M+H]$^+$ calcd for $C_5H_9FNOS$ 150.0389, found 150.0403.

Thiomorpholine-4-carbothioyl fluoride (**2c)**

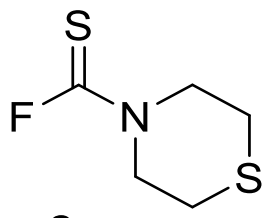

**2c**

Thiomorpholine-4-carbothioyl fluoride **2c** was obtained following the general procedure 1, using KOH as base and by silica pad filtration as a reddish-brown liquid (56 mg, 0.340 mmol, 85%)

$^1$H NMR (400 MHz, $CDCl_3$) δ 4.28 – 4.20 (m, 2H), 4.01 – 3.93 (m, 2H), 2.83 – 2.76 (m, 2H), 2.73 – 2.66 (m, 2H).

$^{13}$C NMR (101 MHz, $CDCl_3$) δ 180.1 (d, *J* = 320.3 Hz), 53.6 (d, *J* = 5.8 Hz), 49.5 (d, *J* = 6.3 Hz), 27.7, 26.7.
$^{19}$F NMR (376 MHz, $CDCl_3$) δ 14.62.
IR (cm$^{-1}$) ν = 2921, 2853, 1737, 1511, 1443, 1229, 1171, 1097, 1032, 949, 884, 665, 539, 494, 436
HRMS (ESI) m/z: $[M+H]^+$ calcd for $C_5H_9FNS_2$ 166.0160, found 166.0175.

6-azaspiro[2.5]octane-6-carbothioyl fluoride (**2d)**

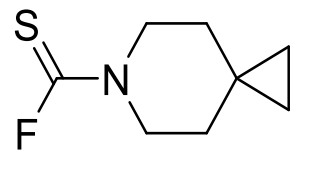


**2d**

6-azaspiro[2.5]octane-6-carbothioyl fluoride **2d** was obtained following the general procedure 1 and silica pad filtration as a brown liquid (43 mg, 0.248 mmol, 62%)

$^{1}$H NMR (400 MHz, $CDCl_3$) δ 4.03 – 3.95 (m, 2H), 3.76 – 3.68 (m, 2H), 1.52 (t, *J* = 5.7 Hz, 2H), 1.48 – 1.40 (m, 2H), 0.46 – 0.36 (m, 4H).
$^{13}$C NMR (101 MHz, $CDCl_3$) δ 180.0 (d, *J* = 319.3 Hz), 51.4 (d, *J* = 6.7 Hz), 47.0 (d, *J* = 6.7 Hz), 34.8, 33.8, 17.2, 11.5.
$^{19}$F NMR (376 MHz, $CDCl_3$) δ 12.92.
IR (cm$^{-1}$) ν = 3071, 2995, 2922, 2848, 1522, 1455, 1272, 1187, 1096, 991, 911, 866, 731, 671, 627, 574, 503
HRMS (ESI) m/z: $[M+H]^+$ calcd for $C_8H_{13}FNS$ 174.0753, found 174.0762.

6,6-dimethyl-3-azabicyclo[3.1.0]hexane-3-carbothioyl fluoride (**2e)**

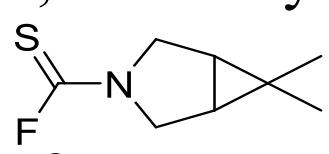


**2e**

6,6-dimethyl-3-azabicyclo[3.1.0]hexane-3-carbothioyl fluoride **2e** was obtained following the general procedure 1, using DABCO and electrolysing up to 9F. The pure product was obtained by silica pad filtration as a yellow liquid (56 mg, 0.324 mmol, 81%) .

$^{1}$H NMR (400 MHz, $CDCl_3$) δ 3.82 – 3.71 (m, 2H), 3.63 (d, *J* = 3.4 Hz, 1H), 3.59 (d, *J* = 2.5 Hz, 1H), 1.52 – 1.45 (m, 2H), 1.04 (s, 3H), 0.90 (s, 3H).
$^{13}$C NMR (101 MHz, $CDCl_3$) δ 177.8 (d, *J* = 321.7 Hz), 53.1 (d, *J* = 6.3 Hz), 48.9, 27.7, 26.9, 26.1, 19.7, 12.5.
$^{19}$F NMR (376 MHz, $CDCl_3$) δ 21.20.
IR (cm$^{-1}$) ν = 3037, 2951, 2881, 1508, 1405, 1332, 1275, 1205, 1109, 1028, 936, 844, 780, 673, 629, 573, 489
HRMS (ESI) m/z: $[M+H]^+$ calcd for $C_8H_{13}FNS$ 174.0753, found 174.0768.

(furan-2-ylmethyl)(methyl)carbamothioic fluoride (**2f'** and **2f'')**

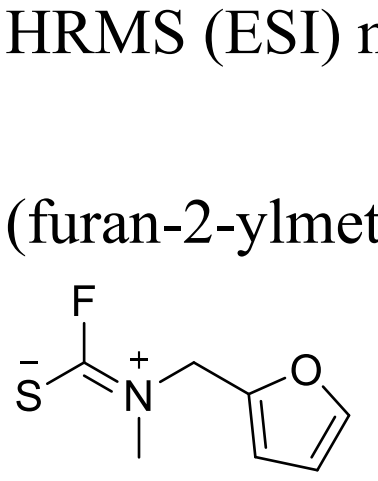


**2f'**

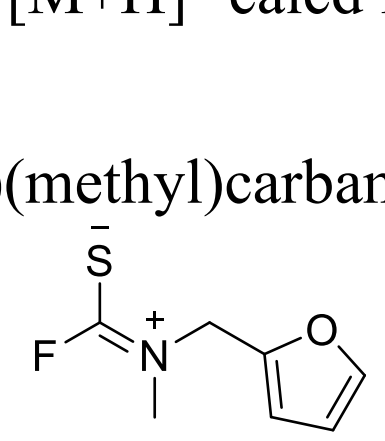


**2f''**

(furan-2-ylmethyl)(methyl)carbamothioic fluoride **2f** was obtained following the general procedure 1, using DABCO and electrolysing up to 8 F. The pure product was obtained by column purification as a pale brown liquid (42 mg, 0.244 mmol, 61%) (**2f'**:**2f''**=4:5).

$^{1}$H NMR (500 MHz, $CDCl_3$) δ 7.42 – 7.40 (m, 1H+1H, **2f’**+**2f”**), 6.46 (d, *J* = 3.3 Hz, 1H, **2f”**), 6.39 – 6.35 (m, 1H+1H, **2f’**+**2f”**), 6.33 (d, *J* = 3.3 Hz, 1H, **2f’**), 4.93 (s, 2H, **2f”**), 4.63 (s, 2H, **2f’**), 3.33 (s, 3H, **2f’**), 3.16 (d, *J* = 2.1 Hz, 3H, **2f”**).
$^{13}$C NMR (101 MHz, $CDCl_3$) δ 182.1 (d, *J* = 321.7 Hz, **2f”**), 181.5 (d, *J* = 321.0 Hz, **2f’**), 147.7 (m, **2f”**+**2f’**), 143.5 (**2f’**), 143.3 (**2f”**), 110.8–110.0 (m, **2f’**+**2f”**), 51.6 (d, *J* = 5.8 Hz, **2f’**), 47.5 (d, *J* = 7.0 Hz, **2f”**), 41.1 (d, *J* = 6.3 Hz, **2f’**), 36.2 (d, *J* = 6.5 Hz, **2f”**).
$^{19}$F NMR (471 MHz, $CDCl_3$) δ 17.35 (**2f”**), 14.46 (**2f’**).
IR ($cm^{-1}$) ν = 2927, 1791, 1688, 1535, 1407, 1268, 1195, 1133, 1080, 1014, 824, 753, 682, 599, 467
HRMS (CI) m/z: $[M+H]^+$ calcd for $C_7H_8FNOS$ 173.0305 found 173.0418 (spectral accuracy: 91.71).

Notably, resonance effects provide two conformations of thiocarbamoyl fluoride, which appear as separate signals in the NMR spectra.[23-24] The NMR spectra of asymmetric thiocarbamoyl fluorides (e.g., 2f, 2g, 2h, 2i, 2j, 4b and 4c) show two sets of resonances corresponding to these conformers, as illustrated in the NMR figures. According to the literature, the N-$CH_3$ protons *cis* to the C–S bond were assigned to the downfield signals.[25] Furthermore, through-space coupling between the fluorine atom and the N-$CH_3$ protons was observed to be larger when they were in a *cis* relationship to fluorine (i.e., *trans* to sulfur).[26] For example, **2f”** exhibits a doublet at 3.16 ppm in its $^{1}$H NMR spectrum.

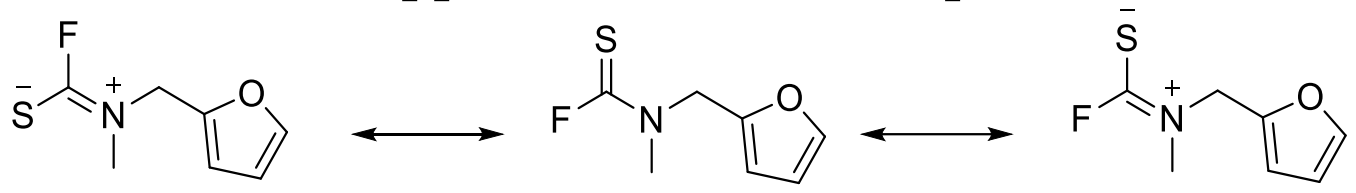
2f' 2f 2f''

Benzyl(methyl)carbamothioic fluoride **(2g’** and **2g”)**

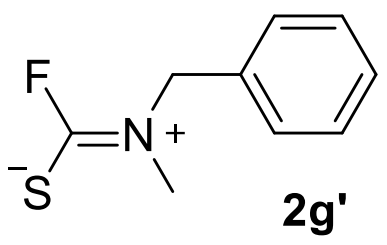
2g'
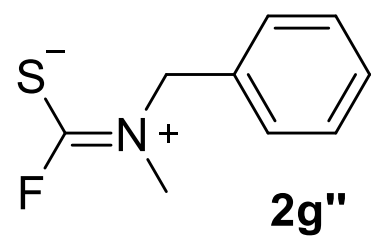
2g''

Benzyl(methyl)carbamothioic fluoride **2g** was obtained following the general procedure 1, using DABCO and electrolysing up to 8 F. The pure product was obtained by silica pad filtration as a yellow liquid (61.0 mg, 0.332 mmol, 83%) (**2g’**:**2g”** = 1:1).

$^{1}$H NMR (500 MHz, $CDCl_3$) δ 7.43 – 7.35 (m, 4H+4H, **2g’**+**2g”**), 7.22 (d, *J* = 6.7 Hz, 1H+1H, **2g’**+**2g”**), 4.99 (s, 2H, **2g”**), 4.69 (s, 2H, **2g’**), 3.27 (s, 3H, **2g’**), 3.07 (d, *J* = 2.4 Hz, 3H, **2g”**).
$^{13}$C NMR (101 MHz, $CDCl_3$) δ 182.7 (d, *J* = 320.9 Hz, **2g”**), 181.4 (d, *J* = 320.0 Hz, **2g’**), 134.1, 129.3, 129.1, 128.6 (d, *J* = 2.4 Hz), 128.2, 127.7 (aromatic signals, **2g’+2g”**), 58.9 (d, *J* = 5.1 Hz, **2g”**), 55.0 (d, *J* = 4.0 Hz, **2g’**), 41.1 (d, *J* = 5.1 Hz, **2g’**), 35.8 (d, *J* = 5.1 Hz, **2g”**).
$^{19}$F NMR (471 MHz, $CDCl_3$) δ 17.34 (**2g’**), 13.32 (**2g”**).
IR ($cm^{-1}$) ν = 3031, 2931, 1787, 1656, 1531, 1453, 1407, 1262, 1135, 1087, 1029, 981, 818, 701, 631, 589, 534, 456
HRMS (EI) m/z: $[M+H]^+$ calcd for $C_9H_{11}FNS$ 184.0596, found 184.0600.
In accordance with previous reports.[27-28]

(4-bromobenzyl)(methyl)carbamothioic fluoride (**2h’** and **2h’’)**

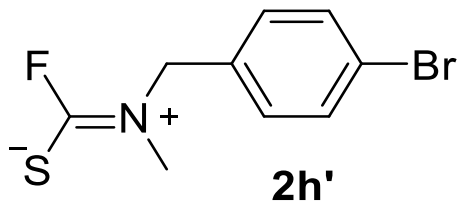
2h'
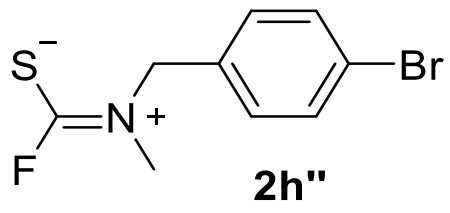
2h''

(4-bromobenzyl)(methyl)carbamothioic fluoride **2h** was obtained following the general procedure 1, using DABCO and electrolysing up to 9 F. The amount of $Et_3N \cdot 3HF$ was increased from 2.0 equiv. to 4.0 equiv. for better conversion of the starting amine. The pure product was obtained by silica pad filtration as a green liquid (62 mg, 0.240 mmol, 60%) (**2h’**:**2h”**=9:10).

$^{1}$H NMR (500 MHz, $CDCl_3$) δ 7.55 – 7.46 (m, 2H+2H, **2h’**+**2h”**), 7.24 (d, *J* = 8.4 Hz, 2H, **2h”**), 7.10 (d, *J* = 8.4 Hz, 2H, **2h’**), 4.93 (s, 2H, **2h”**), 4.63 (s, 2H, **2h’**), 3.26 (s, 3H, **2h’**), 3.07 (d, *J* = 2.1 Hz, 3H, **2h”**).
$^{13}$C NMR (126 MHz, $CDCl_3$) δ 182.5 (d, *J* = 320.9 Hz, **2h”**), 181.1 (d, *J* = 320.3 Hz, **2h’**), 133.0 (**2h’**), 132.9 (**2h”**), 132.2 (**2h’**), 132.1 (**2h”**), 129.7 (**2h”**), 129.2 (**2h’**), 122.5 (**2h’**), 122.5 (**2h”**), 58.1 (d, *J* = 6.0 Hz, **2h”**), 54.3 (d, *J* = 6.0 Hz, **2h’**), 41.0 (d, *J* = 6.0 Hz, **2h’**), 35.8 (d, *J* = 6.6 Hz, **2h”**).
$^{19}$F NMR (471 MHz, $CDCl_3$) δ 17.47 (**2h”**), 13.75 (**2h’**).
IR ($cm^{-1}$) ν = 2924, 2854, 1724, 1459, 1378, 1270, 1099, 731, 472
HRMS (EI) m/z: $[M+H]^+$ calcd for $C_9H_{10}BrFNS$ 261.9701, found 261.9696.

(4-chlorobenzyl)(methyl)carbamothioic fluoride (**2i’** and **2i’’)**

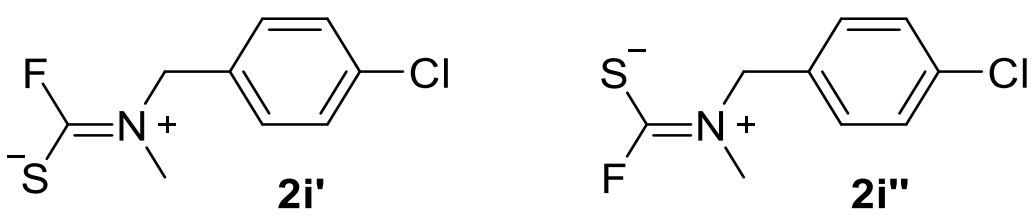
2i' 2i''

(4-chlorobenzyl)(methyl)carbamothioic fluoride **2i** was obtained following the general procedure 1, using DABCO and electrolysing up to 8 F. The amount of $Et_3N \cdot 3HF$ was increased from 2.0 equiv. to 4.0 equiv. for better conversion of the starting amine. The pure product was obtained by silica pad filtration as a pale brown liquid (69 mg, 0.320 mmol, 80%) (2r':2r'' = 9:10).

$^{1}$H NMR (500 MHz, $CDCl_3$) δ 7.37 – 7.32 (m, 2H+2H, **2i’**+**2i”**), 7.32 – 7.29 (m, 2H, **2i”**), 7.18 – 7.14 (m, 2H, **2i’**), 4.95 (s, 2H, **2i”**), 4.65 (s, 2H, **2i’**), 3.26 (s, 3H, **2i’**), 3.07 (d, *J* = 2.1 Hz, 3H, **2i”**).
$^{13}$C NMR (126 MHz, $CDCl_3$) δ 182.7 (d, *J* = 320.9 Hz, **2i”**) 181.3 (d, *J* = 320.3 Hz, **2i’**), 134.6, 134.5, 132.6, 129.5, 129.4, 129.3, 129.1 (aromatic signals, **2i’**+**2i”**), 58.1 (d, *J* = 6.0 Hz, **2i”**), 54.3 (d, *J* = 6.0 Hz, **2i’**), 41.0 (d, *J* = 6.6 Hz, **2i’**), 35.9 (d, *J* = 6.6 Hz, **2i”**).
$^{19}$F NMR (471 MHz, $CDCl_3$) δ 17.5 (**2i”**), 13.7 (**2i’**).
IR ($cm^{-1}$) ν = 2927, 1528, 1403, 1261, 1092, 1015, 919, 800, 733, 681, 625, 561, 483
HRMS (EI) m/z: $[M+H]^+$ calcd for $C_9H_{10}ClFNS$ 218.0207, found 218.0194.

(4-fluorobenzyl)(methyl)carbamothioic fluoride (**2j’ and 2j’’)**

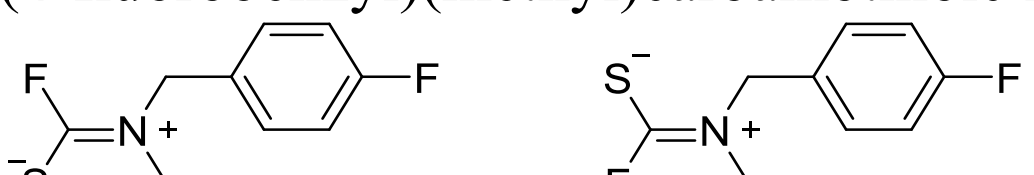
2j' 2j''

(4-fluorobenzyl)(methyl)carbamothioic fluoride **2j’** and **2j’’** was obtained following the general procedure 1, using KOH as base and by silica pad filtration as a yellow liquid (70.8 mg, 0.352 mmol, 88%) (**2j’**:**2j”** = 9:10).

$^{1}$H NMR (400 MHz, $CDCl_3$) δ 7.40 – 7.32 (m, 2H, **2j”**), 7.25 – 7.17 (m, 2H, **2j’**), 7.12 – 6.99 (m, 4H, **2j’**+**2j”**), 4.95 (s, 2H, **2j”**), 4.65 (s, 2H, **2j’**), 3.26 (s, 3H, **2j’**), 3.06 (d, *J* = 2.2 Hz, 3H, **2j”**).
$^{13}$C NMR (101 MHz, $CDCl_3$) δ 182.7 (d, *J* = 320.8 Hz, **2j”**), 181.3 (d, *J* = 320.3 Hz, **2j’**), 164.1 (d, *J* = 5.8 Hz, **2j”**), 161.6 (d, *J* = 5.8 Hz, **2j’**), 130.1 – 129.9 (m, **2j’**+**2j”**), 129.7 –

129.5 (m, **2j’+2j”**), 116.6 – 115.6 (m, **2j’+2j”**), 58.1 (d, *J* = 5.8 Hz, **2j”**), 54.3 (d, *J* = 5.8 Hz, **2j’**), 41.0 (d, *J* = 6.3 Hz, **2j’**), 35.7 (d, *J* = 6.7 Hz, **2j”**). (some signals are overlapped).
$^{19}$F NMR (376 MHz, $CDCl_3$) δ 17.53 (**2j’’**), 13.52 (**2j’**), -112.88 (**2j’’**), -112.99 (**2j’**).
IR (cm$^{-1}$) ν = 3039, 2926, 2853, 1899, 1735, 1606, 1509, 1407, 1222, 1094, 1015, 918, 822, 726, 679, 628, 571, 502, 423.
HRMS (ESI) m/z: $[M+H]^+$ calcd for $C_9H_{10}F_2NS$ 202.0502, found 202.0516.

4-cyanopiperidine-1-carbothioyl fluoride (**2k)**

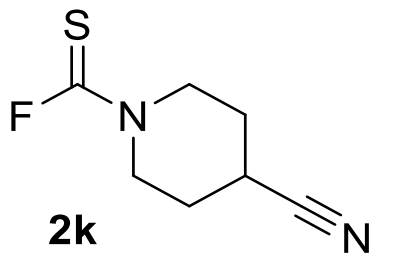

**2k**

4-cyanopiperidine-1-carbothioyl fluoride **2k** was obtained following the general procedure 1, electrolysing at 10 mA, KOH as base, and by silica pad filtration, as a yellow liquid (18 mg, 0.104 mmol, 26%).

$^1$H NMR (400 MHz, $CDCl_3$) δ 4.14 – 4.00 (m, 2H), 3.88 – 3.73 (m, 2H), 3.03 – 2.90 (m, 1H), 2.13 – 1.85 (m, 4H).
$^{13}$C NMR (101 MHz, $CDCl_3$) δ 180.2 (d, *J* = 321.3 Hz), 119.9, 48.6 (d, *J* = 6.3 Hz), 44.4 (d, *J* = 7.2 Hz), 28.4, 27.6, 25.7.
$^{19}$F NMR (376 MHz, $CDCl_3$) δ 14.23.
IR (cm$^{-1}$) ν = 2924, 2854, 2242, 1520, 1450, 1362, 1274, 1107, 1008, 932, 671, 626, 564, 435
HRMS (ESI) m/z: $[M+H]^+$ calcd for $C_7H_{10}FN_2S$ 173.0549, found 173.0561.

4,4-difluoropiperidine-1-carbothioyl fluoride (**2l)**

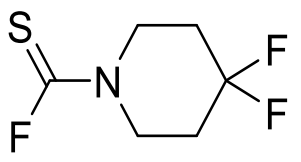

**2l**

4,4-difluoropiperidine-1-carbothioyl fluoride **2l** was obtained following the general procedure 1 using DABCO and by column purification as a colourless liquid (46 mg, 0.256 mmol, 64%).

$^1$H NMR (400 MHz, $CDCl_3$) δ 4.16 – 4.09 (m, 2H), 3.88 – 3.80 (m, 2H), 2.24 – 2.02 (m, 4H).
$^{13}$C NMR (101 MHz, $CDCl_3$) δ 180.40 (d, *J* = 321.5 Hz), 120.41 (td, *J* = 242.8, 1.2 Hz), 47.92 (q, *J* = 5.5 Hz), 43.33 (q), 33.99 (t, *J* = 24.6 Hz), 33.05 (t, *J* = 24.3 Hz).
$^{19}$F NMR (376 MHz, $CDCl_3$) δ 14.54, -99.03 (p, *J* = 13.1 Hz).
IR (cm$^{-1}$) ν = 2926, 2853, 1520, 1457, 1363, 1244, 1186, 1113, 956, 732, 610, 484
HRMS (EI) m/z: $[M+H]^+$ calcd for $C_6H_9F_3NS$ 184.0408, found 184.0420.

4-(trifluoromethyl)piperidine-1-carbothioyl fluoride (**2m)**

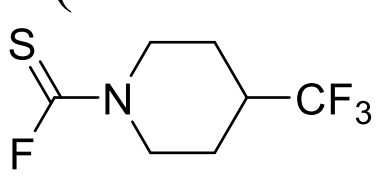

**2m**

4-(trifluoromethyl)piperidine-1-carbothioyl fluoride **2m** was obtained following the general procedure 1 using DABCO and by column purification as a brown liquid (54 mg, 0.252 mmol, 63%).

$^1$H NMR (400 MHz, $CDCl_3$) δ 4.83 (d, *J* = 13.6 Hz, 1H), 4.45 (d, *J* = 13.9 Hz, 1H), 3.19 (tt, *J* = 13.2, 3.0 Hz, 1H), 3.04 (td, *J* = 13.1, 3.0 Hz, 1H), 2.43 – 2.25 (m, 1H), 2.08 – 1.93 (m, 2H), 1.80 – 1.54 (m, 2H).

$^{13}C$ NMR (101 MHz, $CDCl_3$) δ 180.13 (d, *J* = 320.5 Hz), 126.64 (q, *J* = 278.4 Hz), 49.67 (d, *J* = 6.5 Hz), 45.30 (d, *J* = 7.0 Hz), 39.72 (qd, *J* = 28.2, 1.3 Hz), 24.45 (q, *J* = 2.6 Hz), 23.58 (q, *J* = 2.8 Hz).
$^{19}F$ NMR (376 MHz, $CDCl_3$) δ 13.81, -73.57 (d, *J* = 8.2 Hz).
IR ($cm^{-1}$) ν = 2921, 1520, 1454, 1397, 1339, 1281, 1141, 1082, 1001, 938, 882, 814, 702, 528, 419
HRMS (EI) m/z: $[M+H]^+$ calcd for $C_7H_{10}F_4NS$ 216.0470, found 216.0480.

4-(bromomethyl)piperidine-1-carbothioyl fluoride (**2n)**

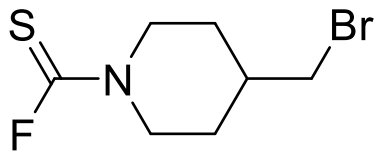


**2n**

4-(bromomethyl)piperidine-1-carbothioyl fluoride **2n** was obtained following the general procedure 1, using KOH and silica pad filtration as a yellow liquid (73 mg, 0.308 mmol, 77%).

$^{1}H$ NMR (500 MHz, $CDCl_3$) δ 4.83 – 4.70 (m, 1H), 4.46 – 4.37 (m, 1H), 3.37 – 3.24 (m, 2H), 3.23 – 3.12 (m, 1H), 3.05 – 2.96 (m, 1H), 2.08 – 1.87 (m, 3H), 1.53 – 1.39 (m, 1H), 1.41 – 1.31 (m, 1H).
$^{13}C$ NMR (126 MHz, $CDCl_3$) δ 179.9 (d, *J* = 320.3 Hz), 50.8 (d, *J* = 6.6 Hz), 46.4 (d, *J* = 7.2 Hz), 37.8, 37.5, 30.6, 29.8.
$^{19}F$ NMR (471 MHz, $CDCl_3$) δ 13.40.
IR ($cm^{-1}$) ν = 2922, 2853, 1784, 1520, 1446, 1372, 1276, 1195, 1093, 962, 730, 673, 626, 568, 588, 492
HRMS (EI) m/z: $[M+H]^+$ calcd for $C_7H_{12}FNSBr$ 239.9858, found 239.9855.

4-methylene piperidine-1-carbothioyl fluoride (**2o)**

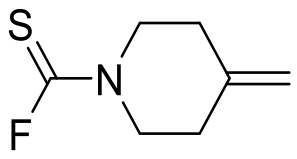


**2o**

4-methylenepiperidine-1-carbothioyl fluoride **2o** was obtained following the general procedure 1, using KOH and silica pad filtration as a brown liquid (31 mg, 0.196 mmol, 49%).

$^{1}H$ NMR (500 MHz, $CDCl_3$) δ 4.87 (d, *J* = 6.3 Hz, 2H), 4.02 – 3.96 (m, 2H), 3.75 – 3.68 (m, 2H), 2.42 – 2.38 (m, 2H), 2.34 – 2.29 (m, 2H).
$^{13}C$ NMR (126 MHz, $CDCl_3$) δ 180.1 (d, *J* = 320.3 Hz), 141.9, 111.6, 52.4 (d, *J* = 6.6 Hz), 47.9 (d, *J* = 6.6 Hz), 34.1, 33.1.
$^{19}F$ NMR (471 MHz, $CDCl_3$) δ 13.21.
IR ($cm^{-1}$) ν = 3075, 2925, 1789, 1653, 1519, 1444, 1359, 1278, 1239, 1098, 984, 900, 808, 764, 685, 638, 495
HRMS (EI) m/z: $[M+H]^+$ calcd for $C_7H_{11}FNS$ 160.0596, found 160.0596.

4-ethynylpiperidine-1-carbothioyl fluoride (**2p)**

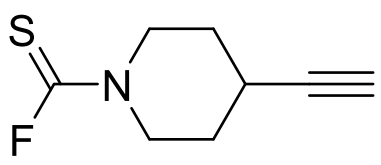


**2p**

4-ethynylpiperidine-1-carbothioyl fluoride **2p** was obtained following the general procedure 1, using DABCO, and silica pad filtration as a brown liquid (33.5 mg, 0.196 mmol, 49%).

$^{1}$H NMR (400 MHz, $CDCl_3$) δ 4.10 – 3.94 (m, 2H), 3.85 – 3.66 (m, 2H), 2.83 – 2.72 (m, 1H), 2.15 (d, $J$ = 2.4 Hz, 1H), 2.00 – 1.66 (m, 4H).
$^{13}$C NMR (101 MHz, $CDCl_3$) δ 179.9 (d, $J$ = 319.8 Hz), 84.5, 71.0, 48.9 (d, $J$ = 6.5 Hz), 44.6 (d, $J$ = 6.9 Hz), 30.9, 30.0, 25.7 (d, $J$ = 1.1 Hz).
$^{19}$F NMR (376 MHz, $CDCl_3$) δ 13.10.
IR (cm$^{-1}$) ν = 3291, 2928, 2856, 1786, 1722, 1664, 1526, 1446, 1366, 1274, 1232, 1104, 1008, 948, 644, 512
HRMS (EI) m/z: $[M+H]^+$ calcd for $C_8H_{11}FNS$ 172.0596, found 172.0607.

4-(dimethoxymethyl)piperidine-1-carbothioyl fluoride (**2q)**

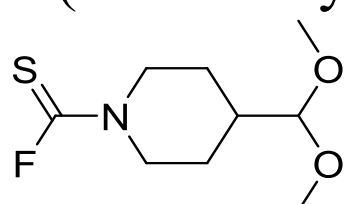


**2q**

4-(dimethoxymethyl)piperidine-1-carbothioyl fluoride **2q** was obtained following the general procedure 1, using DABCO and electrolysing up to 6 F. The pure product was obtained by silica pad filtration as a orangish brown liquid (26 mg, 0.120 mmol, 30%).

$^{1}$H NMR (500 MHz, $CDCl_3$) δ 4.81 – 4.72 (m, 1H), 4.45 – 4.37 (m, 1H), 4.05 (d, $J$ = 6.6 Hz, 1H), 3.36 (d, $J$ = 2.6 Hz, 9H), 3.19 – 3.09 (m, 1H), 3.02 – 2.93 (m, 1H), 1.95 – 1.76 (m, 3H), 1.52 – 1.41 (m, 1H), 1.38 – 1.32 (m, 1H).
$^{13}$C NMR (126 MHz, $CDCl_3$) δ 178.0 (d, $J$ = 319.7 Hz), 107.3, 54.4, 51.0 (d, $J$ = 6.6 Hz), 46.6 (d, $J$ = 6.6 Hz), 38.3, 27.1, 26.3.
$^{19}$F NMR (471 MHz, $CDCl_3$) δ 12.97.
IR (cm$^{-1}$) ν = 2928, 1727, 1527, 1449, 1373, 1274, 1195, 1116, 977, 671, 627, 517, 422
HRMS (EI) m/z: $[M-CH_3O]^+$ calcd for $C_8H_{13}FNOS$ 190.0703, found 190.0702.

Methyl 1-(fluorocarbonothioyl)piperidine-4-carboxylate (**2r**)

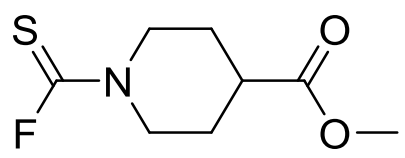


**2r**

Methyl 1-(fluorocarbonothioyl)piperidine-4-carboxylate **2r** was obtained following the general procedure 1, using DABCO and electrolysing up to 6 F. The pure product was obtained by silica pad filtration as a pale yellow liquid ( 36mg, 0.176 mmol, 44%).

$^{1}$H NMR (500 MHz, $CDCl_3$) δ 4.54 – 4.45 (m, 1H), 4.22 – 4.14 (m, 1H), 3.71 (s, 3H), 3.53 – 3.44 (m, 1H), 3.32 – 3.25 (m, 1H), 2.67 – 2.58 (m, 1H), 2.11 – 2.03 (m, 1H), 2.03 – 1.96 (m, 1H), 1.95 – 1.76 (m, 2H).
$^{13}$C NMR (126 MHz, $CDCl_3$) δ 180.0 (d, $J$ = 320.3 Hz), 173.8, 52.2, 50.1 (d, $J$ = 6.6 Hz), 45.7 (d, $J$ = 7.2 Hz), 39.7, 27.7, 27.0.
$^{19}$F NMR (471 MHz, $CDCl_3$) δ 13.36.
IR (cm$^{-1}$) ν = 2953, 1731, 1524, 1449, 1378, 1276, 1194, 1109, 1037, 937, 893, 772, 666, 626, 537
HRMS (EI) m/z: $[M+H]^+$ calcd for $C_8H_{13}FNSO_2$ 206.0651, found 206.0656.

3,3-dimethyl-4-oxopiperidine-1-carbothioyl fluoride (**2s’** and **2s”)**

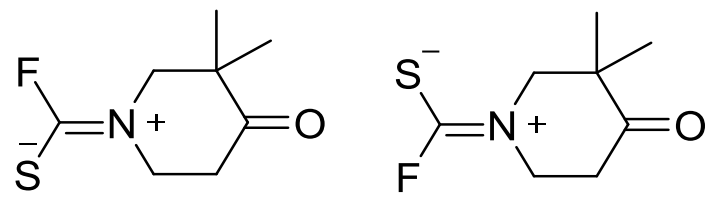

**2s'** **2s''**

3,3-dimethyl-4-oxopiperidine-1-carbothioyl fluoride **2s** was obtained following the general procedure 1, using DABCO and electrolysing at 10 mA. The pure product was obtained by column purification as a yellow liquid (23 mg, 0.120 mmol, 30%) (**2s'**:**2s"** = 5:3).

$^{1}$H NMR (500 MHz, $CDCl_3$) δ 4.19 (t, *J* = 6.7 Hz, 2H, **2s'**), 4.03 (s, 1H, **2s''**), 3.99 (td, *J* = 6.7, 0.9 Hz, 1H, **2s''**), 3.71 (d, *J* = 0.8 Hz, 2H, **2s'**), 2.69 (td, *J* = 6.7, 1.3 Hz, 2H, **2s'**), 2.62 (t, 1H, **2s''**), 1.19 (s, 4H, **2s''**), 1.14 (s, 6H, **2s'**).
$^{13}$C NMR (126 MHz, $CDCl_3$) δ 209.0 (**2s'**+**2s''**), 181.4 (d, *J* = 320.9 Hz, **2s''**). 180.7 (d, *J* = 321.5 Hz, **2s'**), 59.9 (d, *J* = 6.6 Hz, **2s''**), 56.0 (d, *J* = 4.8 Hz, **2s'**), 50.0 (d, *J* = 6.6 Hz, **2s'**), 46.9 (**2s'**), 46.5 (**2s''**), 45.3 (d, *J* = 7.2 Hz, **2s''**), 36.8 (**2s''**), 36.3 (**2s'**), 22.5 (**2s''**), 22.5 (**2s'**).
$^{19}$F NMR (471 MHz, $CDCl_3$) δ 15.20 (**2s''**), 12.34 (**2s'**).
IR ($cm^{-1}$) ν = 2981, 2883, 1721, 1656, 1523, 1439, 1371, 1287, 1181, 1106, 1015, 952, 730, 674, 625, 548, 478
HRMS (EI) m/z: $[M+H]^+$ calcd for $C_8H_{13}FNOS$ 190.0702, found 190.0698.

tert-butyl 4-(fluorocarbonothioyl)piperazine-1-carboxylate (**2t)**

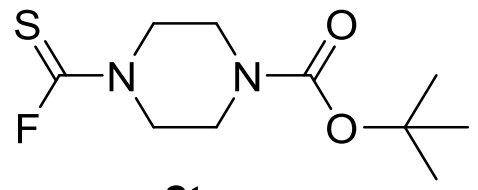

**2t**

tert-butyl 4-(fluorocarbonothioyl)piperazine-1-carboxylate **2t** was obtained following the general procedure 1, using KOH and silica pad filtration as a pale brown solid (72 mg, 0.292 mmol, 73%).

$^{1}$H NMR (500 MHz, $CDCl_3$) δ 3.98 – 3.92 (m, 2H), 3.70 (t, *J* = 5.4 Hz, 2H), 3.61 – 3.55 (m, 2H), 3.53 – 3.47 (m, 2H), 1.47 (s, 9H).
$^{13}$C NMR (126 MHz, $CDCl_3$) δ 180.2 (d, *J* = 320.9 Hz), 154.2, 80.9, 50.2, 46.5, 28.3.
$^{19}$F NMR (471 MHz, $CDCl_3$) δ 14.29.
IR ($cm^{-1}$) ν = 2977, 2933, 2872, 1682, 1526, 1411, 1366, 1263, 1164, 1104, 1023, 909, 857, 731, 676, 623, 558, 494, 453
HRMS (EI) m/z: $[M-O^tBu]^+$ calcd for $C_6H_8FN_2OS$ 175.0341, found 175.0342.

4-(tetrahydrofuran-2-carbonyl)piperidine-1-carbothioyl fluoride (**2u)**

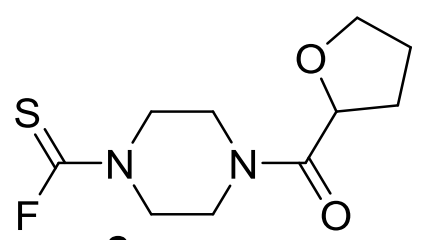

**2u**

4-(tetrahydrofuran-2-carbonyl)piperidine-1-carbothioyl fluoride **2u** was obtained following the general procedure 1, using KOH and electrolysing at 10 mA. The pure product was obtained by column purification as a yellow liquid (41 mg, 0.167 mmol, 42%).

$^{1}$H NMR (400 MHz, $CDCl_3$) δ 4.58 – 4.48 (m, 1H), 4.12 (d, *J* = 12.6 Hz, 1H), 4.00 – 3.68 (m, 6H), 3.66 – 3.36 (m, 3H), 2.42 – 2.24 (m, 1H), 2.04 – 1.82 (m, 3H).
$^{13}$C NMR (126 MHz, $CDCl_3$) (two isomers) δ 180.1 (d, *J* = 320.9 Hz), 180.0 (d, *J* = 320.1 Hz),

169.9, 169.7, 76.0, 76.0, 69.1, 50.5 (d, $J$ = 6.0 Hz), 50.0 (d, $J$ = 6.0 Hz), 46.7 (d, $J$ = 4.8 Hz), 46.2 (d, $J$ = 5.4 Hz), 44.5, 44.0, 41.2, 40.7, 27.8 (d, $J$ = 12.6 Hz), 25.7.
$^{19}$F NMR (471 MHz, $CDCl_3$) δ 14.36, 14.07.
IR ($cm^{-1}$) ν = 2979, 2930, 2872, 1747, 1654, 1521, 1437, 1369, 1287, 1226, 1181, 1110, 1060, 1014, 673, 625, 549, 464
HRMS (EI) m/z: $[M+H]^+$ calcd for $C_{11}H_{16}FNO_2S$ 247.0917, found 247.0914.

methyl(phenethyl)carbamothioic fluoride (**2v’** and **2v”**)

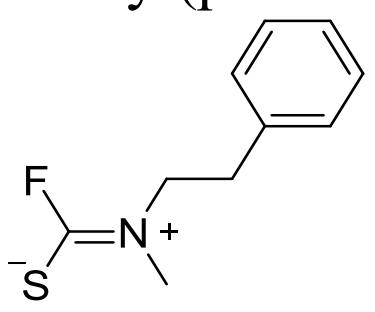

**2v'**

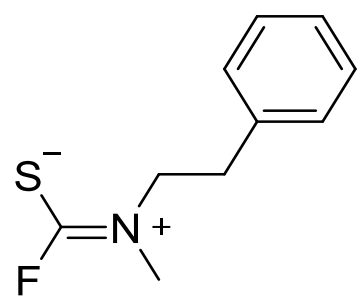

**2v"**

Methyl(phenethyl)carbamothioic fluoride **2v** was obtained following the modified general procedure 1, using KOH as base. Due to the high tendency of $R_2NCOF$ by product formation and resulting in difficult column purification, amount of $Et_4NBr$ was reduced to 8.4 mg (0.04 mmol, 0.1 equiv.) and electron density was reduced to 5.55 mA $cm^{-2}$ for 10 F $mol^{-1}$ (i.e., 10.72 h) to obtain the pure product as a pale yellow oil in 33% (26.0 mg, 0.132 mmol).

$^{1}$H NMR (400 MHz, $CDCl_3$) δ 7.34 – 7.30 (m, 2H+2H, **2v’**+**2v”**), 7.25 – 7.22 (m, 2H+2H, **2v’**+**2v”**), 7.18 – 7.15 (m, 1H+1H, **2v’**+**2v”**), 3.92 – 3.88 (m, 2H, **2v”**), 3.73 – 3.69 (m, 2H, **2v’**), 3.19 (s, 3H, **2v’**), 3.06 – 3.03 (m, 2H, **2v”**), 3.00 (s, 3H, **2v”**), 2.93 – 2.90 (m, 2H, **2v’**)
$^{13}$C NMR (101 MHz, $CDCl_3$) δ 181.45 (d, $J$ = 316.4 Hz, **2v’**+**2v”**), 137.7 (**2v”**), 137.2 (**2v’**), 129.0, 128.9, 128.8, 128.7 (**2v’**+**2v”**) 127.2 (**2v’**), 127.0 (**2v”**), 57.3 (d, $J$ = 6.0 Hz, **2v”**), 53.6 (d, $J$ = 4.3 Hz, **2v’**), 42.1 (d, $J$ = 6.0 Hz, **2v’**), 37.5 (d, $J$ = 6.5 Hz, **2v”**), 34.6 (**2v’**), 31.7 (d, $J$ = 1.7 Hz, **2v”**).
$^{19}$F NMR (376 MHz, $CDCl_3$) δ 17.52 (**2v”**), 13.40 (**2v’**).
IR ($cm^{-1}$) ν = 2977, 1787, 1400, 1114 .
HRMS (ESI) m/z: $[M+H]^+$ calcd for $C_{10}H_{13}FNS$ 198.0753, found 198.0746.

methyl(3-phenyl-3-(4-(trifluoromethyl)phenoxy)propyl)carbamothioic fluoride (**2w’** and **2w”)**

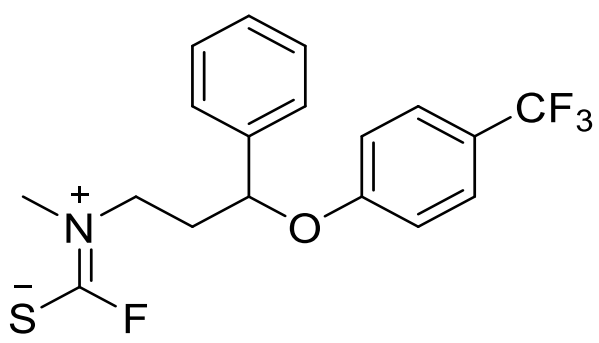

**2w'**

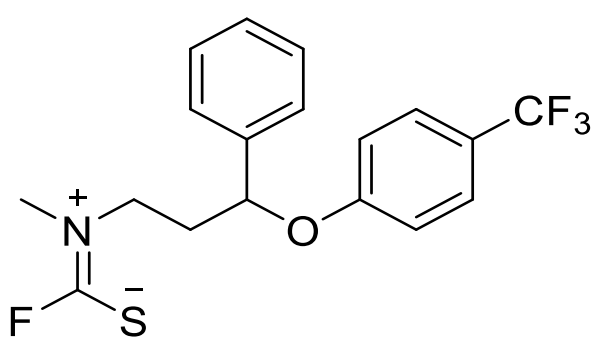

**2w"**

methyl(3-phenyl-3-(4-(trifluoromethyl)phenoxy)propyl)carbamothioic fluoride **2w** was obtained as an yellow oil in 34% (51.0 mg, 0.136 mmol) following the general procedure 1, using KOH as base and by using cyclohexane/DCM solvent system for column purification.

$^{1}$H NMR (500 MHz, $CDCl_3$) δ 7.35 (d, $J$ = 9.0 Hz, 4H, **2w’+2w”**), 7.30 – 7.14 (m, 10H, **2w’+2w”),** 6.84 – 6.78 (m, 4H, **2w’+2w”),** 5.18 (dd, $J$ = 8.5, 4.3 Hz, 1H, **2w”**), 5.10 (dd, $J$ = 9.0, 3.7 Hz, 1H, **2w’**), 3.91 – 3.78 (m, 2H, **2w”**), 3.76 – 3.56 (m, 2H, **2w’**), 3.23 (s, 3H, **2w’**), 3.05 (d, $J$ = 2.3 Hz, 3H, **2w”),** 2.38 – 2.07 (m, 4H, **2w’+2w”).**
$^{13}$C NMR (126 MHz, $CDCl_3$) δ 181.62 (d, $J$ = 321.6 Hz, **2w’**), 181.58 (d, $J$ = 320.3 Hz, **2w”**), 160.1 (**2w”**), 159.9 (**2w’**), 140.0 (**2w’**), 139.6 (**2w”**), 129.2 (**2w’**),129.1 (**2w”**) 128.5 (**2w’**), 129.4 (**2w”**) 127.1 – 126.9 (m, **2w’+2w”),** 125.7 (**2w”),** 125.6 (**2w’**), 123.4 (q, $J$ = 17.4 Hz,

**2w”+2w’**), 115.8 (**2w’**), 115.8 (**2w”**), 77.9 (**2w”+2w’**), 52.8 (d, *J* = 6.0 Hz, **2w”**), 48.8 (d, *J* = 5.4 Hz, **2w’**), 41.9 (d, *J* = 6.0 Hz, **2w”),** 37.2 (d, *J* = 6.6 Hz, **2w’**), 36.9 (**2w”**), 34.6 (**2w’**).
$^{19}$F NMR (471 MHz, $CDCl_3$) δ 17.88 (**2w”),** 13.87 (**2w’**), -61.48 (**2w”**), -61.51 (**2w’**).
IR (cm$^{-1}$) ν = 2958, 1538, 1409, 1322, 1244, 1106, 1063, 834, 699.
HRMS (ESI) m/z: $[M+H]^+$ calcd for $C_{18}H_{18}F_4NOS$ 372.1045, found 372.1037.

(3,4-dimethoxyphenethyl)(methyl)carbamothioic fluoride (**2x’** and **2x”)**

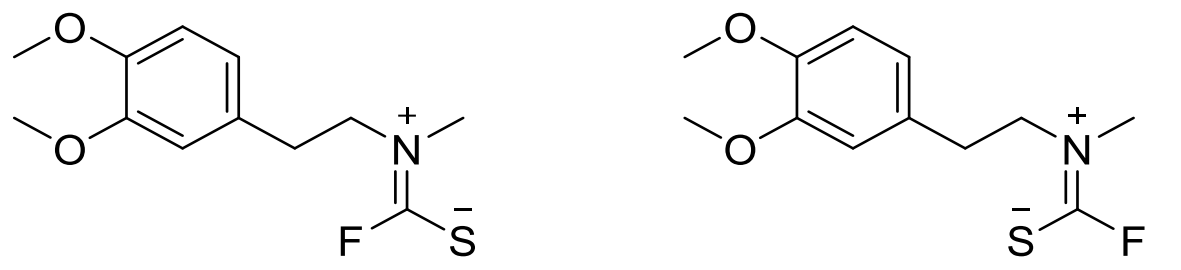

**2x'** **2x"**

(3,4-dimethoxyphenethyl)(methyl)carbamothioic fluoride **2x** was obtained following the modified general procedure 1, using KOH as base. Due to the high tendency of $R_2NCOF$ by product formation and resulting in difficult column purification, amount of $Et_4NBr$ was reduced to 8.4 mg (0.04 mmol, 0.1 equiv.) and electron density was reduced to 5.55 mA cm$^{-2}$ for 10 F mol$^{-1}$ (i.e., 10.72 h) to obtain the pure product as a pale yellow oil in 12% (12.0 mg, 0.048 mmol).

$^1$H NMR (500 MHz, $CDCl_3$) δ 6.83 (s, 0.62H**, 2x’**), 6.81 (s, 1H, **2x”**), 6.80 – 6.77 (m, 1.67H, **2x’+2x”**), 6.72 (dd, *J* = 8.1, 2.1 Hz, 0.89H, **2x’**), 6.66 (d, *J* = 2.0 Hz, 0.84H, **2x’**), 3.91 – 3.88 (m, 8H, **2x”**), 3.87 (s, 5.68H, **2x’**), 3.74 – 3.67 (m, 1.88H, **2x’**), 3.18 (s, 2.41H, **2x’**), 3.02 (d, *J* = 2.2 Hz, 3H, **2x”),** 3.00 (d, *J* = 7.3 Hz, 1.33 H, **2x”**), 2.90 – 2.84 (m, 2H, **2x’**).
$^{13}$C NMR (126 MHz, $CDCl_3$) δ 181.5 (d, *J* = 319 Hz, **2x’**), 149.3 (**2x’**), 149.3 (**2x”**), 148.2 (**2x”),** 148.1 (**2x’**), 130.1 (**2x”**), 129.7 (**2x’**), 120.9 (**2x’**), 120.8 (**2x”**), 112.0 (**2x’**), 111.8 (**2x”**), 111.6 (**2x’**), 111.5 (**2x”),** 57.44 (d, *J* = 7.2 Hz, **2x’**), 56.1 – 56.0 (m, **2x’+2x”**), 53.9 (d, *J* = 4.2 Hz, **2x”**), 42.3 (d, *J* = 6.6 Hz, **2x”**), 37.6 (d, *J* = 6.6 Hz, **2x’**), 34.3 (**2x’**), 31.4 (**2x”**). C-F carbon peak from another rotamer is missing due to lower concentration of the rotamer.
$^{19}$F NMR (471 MHz, $CDCl_3$) δ 17.47(**2x”**), 13.49 (**2x’**).
IR (cm$^{-1}$) ν = 2957, 1516, 1409, 1261, 1114.
HRMS (ESI) m/z: $[M+H]^+$ calcd for $C_{12}H_{17}FNO_2S$ 258.0964, found 258.0959.

**Supplementary thiocarbamoyl synthesis of aromatic amines**

**General procedure 1A:** Ethyl diphenylcarbamodithioate **1aa** (109.0 mg, 0.4 mmol, 1.0 equiv.) (prepared according to a previously reported procedure)[29] was added into a 5 mL one compartment Electrasyn vial, then acetonitrile (ACN) (5 mL, 0.08 M), $Et_4NBr$ (8.4 mg, 0.04 mmol, 0.1 equiv.) and $Et_3N{\cdot}3HF$ (120 µL, 0.8 mmol, 2.0 equiv.) was added. The solution was then electrolyzed at 5.56 mA $cm^{-2}$ for 10 F $mol^{-1}$ (i.e. 10.72 h) using a platinum anode (WE) and platinum cathode (CE). The reaction is monitored by HPLC. After full consumption of the starting ethyl diphenylcarbamodithioate (**1aa**), the reaction mixture was quenched with a saturated aqueous solution of $NaHCO_3$, extracted with DCM. The organic layers were then combined and dried with $Na_2SO_4$. The solvent was removed under reduced pressure. The crude mixture was filtered through silica and purified via flash column chromatography to obtain a colourless liquid (**2y**) in 66% yield (61.0 mg, 0.264 mmol).

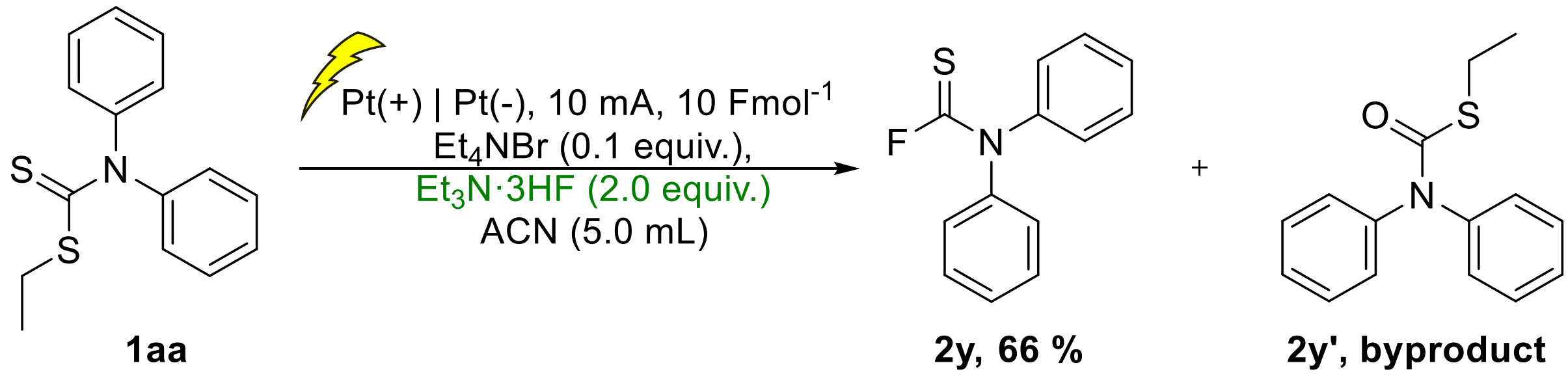


Diphenylcarbamothioic fluoride (**2y**)

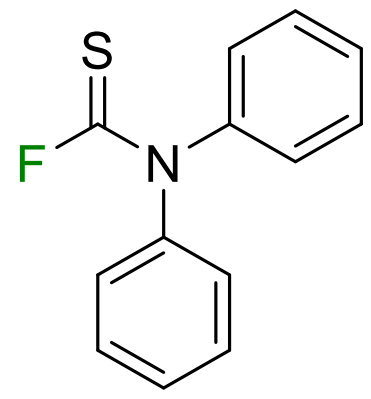

**2y**

$^{1}$H NMR (500 MHz, $CDCl_3$) δ 7.47 – 7.24 (m, 10H).
$^{13}$C NMR (101 MHz, $CDCl_3$) δ 181.2 (d, $J$ = 323.9 Hz), 129.7 (d, $J$ = 10.6 Hz), 128.5 (d, $J$ = 30.0 Hz), 127.3, 126.0.
$^{19}$F NMR (376 MHz, $CDCl_3$) δ 26.5.
IR ($cm^{-1}$) ν = 2978, 1422, 1254, 1154, 954
HRMS (ESI) m/z: $[M+H]^+$ calcd for $C_{13}H_{11}FNS$ 232.0596, found 232.0594.

**Supplementary electrochemical preparation of trifluoromethylamine:**

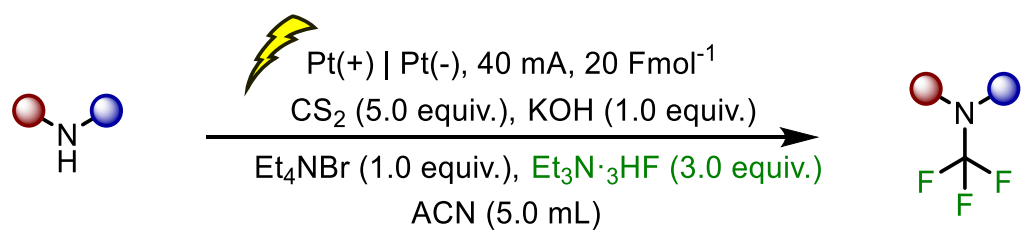


**General procedure 2:** Amine (0.4 mmol, 1.0 equiv.), KOH (22.4 mg, 0.4 mmol, 1.0 equiv.) and $CS_2$ (152 mg, 2.0 mmol, 5.0 equiv.) was added into a 5 mL one compartment Electrasyn vial to generate in situ salt. Then acetonitrile (ACN) (5 mL, 0.08 M), $Et_4NBr$ (84.0 mg, 0.4 mmol, 1.0 equiv.) and $Et_3N{\cdot}3HF$ (196 µL, 1.2 mmol, 3.0 equiv.) was added. The solution was then electrolyzed at 22.2 mA $cm^{-2}$ up to 20 F $mol^{-1}$ unless mentioned otherwise using a platinum anode (WE) and platinum cathode (CE). The reaction was monitored by $^{19}$F NMR until full consumption of the NCSF intermediate. Then, the solvent was evaporated, diluted in Hexane and filtered through celite to obtain the final product.

N,N-dibenzyl-1,1,1-trifluoromethanamine (**3a**)

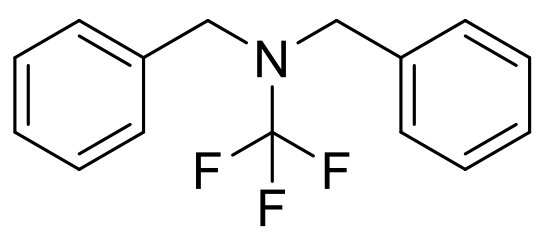


**3a**

Obtained following the general procedure **2** after 20 F mol$^{-1}$ as a yellow liquid (97.7 mg, 0.368 mmol, 92%).

$^{1}$H NMR (400 MHz, $CDCl_3$) δ 7.34 – 7.20 (m, 10H), 4.02 (s, 4H).
$^{13}$C NMR (101 MHz, $CDCl_3$) δ 136.6, 128.7, 128.6, 127.7, 125.22 (q, $J_{C-F}$ = 255.5 Hz), 49.7.
$^{19}$F NMR (376 MHz, $CDCl_3$) δ -59.10.
IR (cm$^{-1}$) ν = 3034, 2930, 1495, 1398, 1222, 1094, 1076, 1027, 750, 696.
GC-MS (EI) m/z: $[M]^+$ calcd for $C_{15}H_{14}F_3N$, found 265.1000
In accordance with previous reports.[23]

N-methyl-N-(naphthalen-2-ylmethyl)trifluoromethanamine (**3b**)

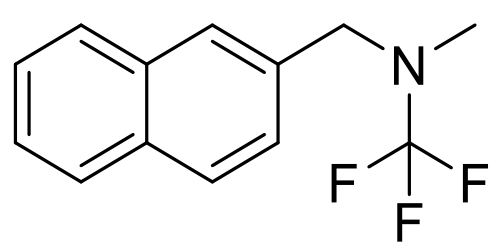


**3b**

Obtained following the general procedure **2** after 26 F mol$^{-1}$ as a yellow liquid (65.7 mg, 0.275 mmol, 69%).

$^{1}$H NMR (400 MHz, $CDCl_3$) δ 7.92 – 7.80 (m, 3H), 7.76 (s, 1H), 7.56 – 7.43 (m, 3H), 4.12 (s, 2H), 2.49 (s, 3H).
$^{13}$C NMR (101 MHz, $CDCl_3$) δ 134.1, 133.4, 133.1, 128.6, 127.9, 127.3, 126.4, 126.3, 126.2, 125.5 (q, $J_{C-F}$ = 259.5 Hz), 53.06 (q, $J_{C-F}$ = 2.6 Hz), 33.3 (d, $J_{C-F}$ = 3.1 Hz).
$^{19}$F NMR (376 MHz, $CDCl_3$) δ -65.51.
IR (cm$^{-1}$) ν = 3063, 2930, 2854, 1431, 1404, 1281, 1231, 1106.
HRMS (ESI) m/z: $[M-H]^-$ calcd for $C_{13}H_{11}NF_3$ 238.0844, found 238.0840.

tert-butyl 4-(trifluoromethyl)piperazine-1-carboxylate (**3c**)

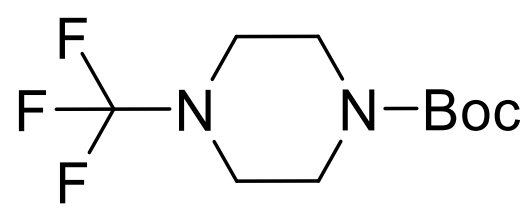


**3c**

Obtained following the general procedure **2** after 32 F mol$^{-1}$ as a yellow solid (69.8 mg, 0.274 mmol, 68%).

$^{1}$H NMR (400 MHz, $CDCl_3$) δ 3.48 (t, J = 5.2 Hz, 4H), 2.87 (t, J = 5.2 Hz, 4H), 1.47 (s, 9H).
$^{13}$C NMR (101 MHz, $CDCl_3$) δ 154.5, 124.4 (q, $J_{C-F}$ = 256.2 Hz), 80.3, 44.23 (d, $J_{C-F}$ = 3.0 Hz), 28.4.
$^{19}$F NMR (376 MHz, $CDCl_3$) δ -67.92.
IR (cm$^{-1}$) ν = 2972, 2927, 2857, 1693, 1426, 1361, 1240, 1164, 1130, 1006, 769, 747.

GC-MS (EI) m/z: $[M]^+$ calcd for $C_{10}H_{17}F_3N_2O_2$ found 254.1500
In accordance with previous reports.[30]

(tetrahydrofuran-2-yl)(4-(trifluoromethyl)piperazin-1-yl)methanone (**3d**)

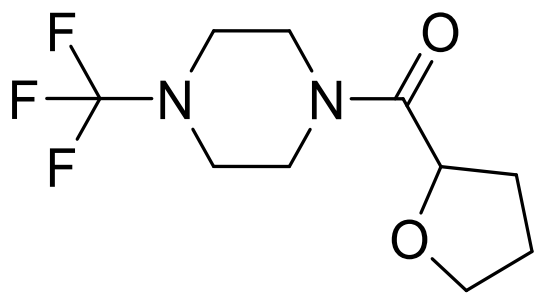

**3d**

Obtained following the general procedure **2** after 26 F $mol^{-1}$ as a yellow liquid (66.9 mg, 0.243 mmol, 61%).

$^{1}H$ NMR (400 MHz, $CDCl_3$) δ 4.56 (dd, J = 7.2, 5.5 Hz, 1H), 3.94 – 3.72 (m, 4H), 3.62 – 3.50 (m, 2H), 2.96 – 2.82 (m, 4H), 2.38 – 2.27 (m, 1H), 2.06 – 1.94 (m, 2H), 1.94 – 1.84 (m, 1H).
$^{13}C$ NMR (101 MHz, $CDCl_3$) δ 169.9, 124.2 (q, $J_{C-F}$ = 256.5 Hz), 76.1, 69.2, 44.7, 44.5, 44.2 (d, $J_{C-F}$ = 3.8 Hz), 41.0, 28.2, 25.8.
$^{19}F$ NMR (376 MHz, $CDCl_3$) δ -67.94.
IR ($cm^{-1}$) ν = 2939, 2875, 1650, 1437, 1358, 1282, 1228, 1103, 1034.
HRMS (ESI) m/z: $[M+H]^+$ calcd for $C_{10}H_{16}N_2O_2F_3$ 253.1164, found 253.1170.

6-Bromo-3,4-dihydro-1H-isoquinoline-2-trifluoromethylamine (**3e**)

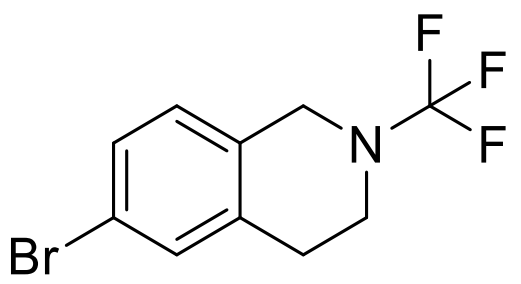

**3e**

Obtained following the general procedure **2** after 20 F $mol^{-1}$ as a yellow liquid (84.0 mg, 0.300 mmol, 75%).

$^{1}H$ NMR (400 MHz, $CDCl_3$) δ 7.31 – 7.28 (m, 2H), 6.94 (d, $J$ = 8.7 Hz, 1H), 4.08 (s, 2H), 3.19 (t, $J$ = 5.9 Hz, 2H), 2.91 (t, $J$ = 6.0 Hz, 2H).
$^{13}C$ NMR (101 MHz, $CDCl_3$) δ 135.8, 131.8, 131.1, 129.5, 128.3, 120.4, 46.1, 41.9 (d, $J_{C-F}$ = 2.9 Hz), 28.2.
$^{19}F$ NMR (376 MHz, $CDCl_3$) δ -66.85.
IR ($cm^{-1}$) ν = 2960, 2917, 2860, 1486, 1364, 1249, 1282, 1224, 1134, 1091, 578.
HRMS ESI (-) m/z: $[M-CF_3]^-$ calcd for $C_9H_9NBr$ 209.9918, found 209.9927.
In accordance with previous reports.[30]

6-Chloro-3,4-dihydro-1H-isoquinoline-2-trifluoromethylamine (**3f**)

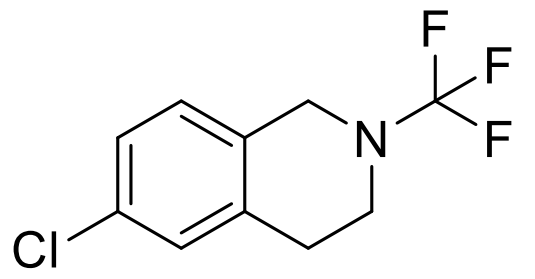

**3f**

Obtained following the general procedure **2** after 22 F $mol^{-1}$ as a yellow liquid (76.1 mg, 0.322 mmol, 71%.).

$^{1}$H NMR (400 MHz, $CDCl_3$) δ 7.17 – 7.11 (m, 2H), 7.00 (dd, J = 7.9, 0.9 Hz, 1H), 4.10 (s, 2H), 3.19 (t, J = 5.9 Hz, 2H), 2.90 (t, J = 5.9 Hz, 2H).

$^{13}$C NMR (101 MHz, $CDCl_3$) δ 135.4, 132.4, 130.6, 128.8, 128.0, 126.6, 124.75 (q, $J_{C-F}$ = 256.1 Hz), 46.0 (d, $J_{C-F}$ = 3.6 Hz), 41.9 (q, $J_{C-F}$ = 3.0 Hz), 28.3.

$^{19}$F NMR (376 MHz, $CDCl_3$) δ -66.85.

IR ($cm^{-1}$) ν = 3058, 2933, 2854, 1401, 1264, 1215, 1116, 739.

HRMS (ESI) m/z: $[M-H]^-$ calcd for $C_{10}H_8NF_3Cl$ 234.0297, found 234.0304.

1,1,1-trifluoro-N-(4-methoxybenzyl)-N-methylmethanamine (**3g**)

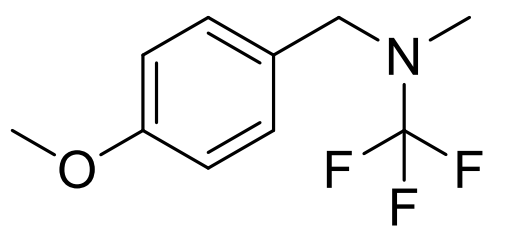

**3g**

Obtained following the general procedure **2** after 28 F $mol^{-1}$ as a yellow liquid (60.6 mg, 0.276 mmol, 69%).

$^{1}$H NMR (400 MHz, $CDCl_3$) δ 7.25 – 7.21 (m, 2H), 6.92 – 6.85 (m, 2H), 3.89 (s, 2H), 3.81 (s, 3H), 2.42 (d, *J* = 1.2 Hz, 3H).

$^{13}$C NMR (101 MHz, $CDCl_3$) δ 159.3, 131.0, 129.8, 128.5, 114.1, 55.4, 52.2 (q, $J_{C-F}$ = 2.7 Hz), 33.0 (q, $J_{C-F}$ = 3.0 Hz).

$^{19}$F NMR (376 MHz, $CDCl_3$) δ -65.45.

IR ($cm^{-1}$) ν = 2960, 2933, 2857, 1617, 1513, 1404, 1249, 1173, 1118, 1042.

GC-MS (EI) m/z: $[M]^+$ calcd for $C_{10}H_{12}F_3NO$ found 219.1000.

Ethyl N-benzyl-N-(trifluoromethyl)glycinate (**3h**)

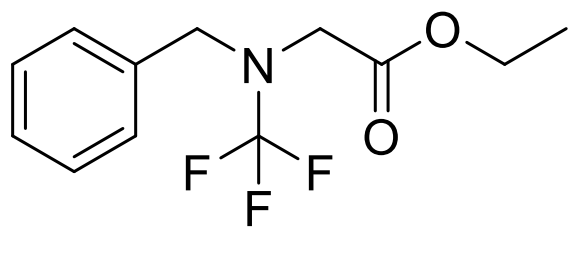

**3h**

Obtained following the general procedure **2** after 22 F $mol^{-1}$ as a yellow liquid (41.2 mg, 0.158 mmol, 39%).

$^{1}$H NMR (400 MHz, $CDCl_3$) δ 7.35 – 7.34 (m, 1H), 7.34 – 7.32 (m, 3H), 7.31 (d, J = 1.6 Hz, 1H), 4.25 (s, 2H), 4.14 (q, J = 7.1 Hz, 2H), 3.57 (q, J = 1.4 Hz, 2H), 1.24 (t, J = 7.2 Hz, 3H).

$^{13}$C NMR (101 MHz, $CDCl_3$) δ 169.4, 135.9, 128.8, 128.6, 128.0, 124.5 (q, $J_{C-F}$ = 255.3 Hz), 61.2, 50.5, 46.7 (d, $J_{C-F}$ = 2.1 Hz), 14.2.

$^{19}F$ NMR (376 MHz, $CDCl_3$) δ -59.77.
IR ($cm^{-1}$) ν = 2984, 2936, 1747, 1647, 1431, 1228, 1191, 1034.
GC-MS (EI) m/z: $[M]^+$ calcd for $C_{12}H_{14}F_3NO_2$ found 261.0000.
In accordance with previous reports.[23]

N-methyl-3-phenyl-3-(4-(trifluoromethyl)phenoxy)propan-1-amine (**3i**)

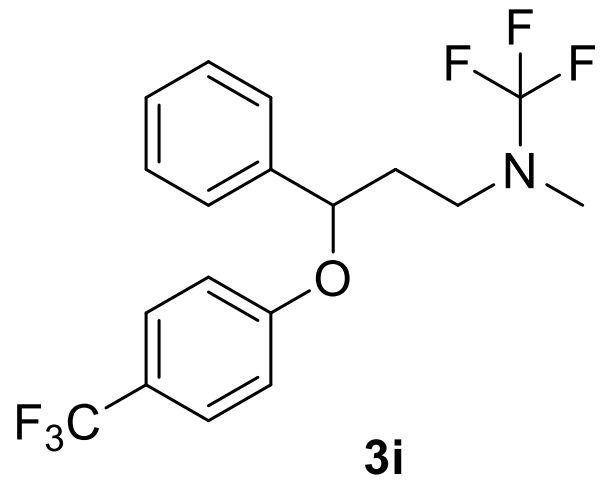


**3i**

Obtained following the general procedure **2** after 20 F $mol^{-1}$ as a yellow liquid (66.9 mg, 0.216 mmol, 44%).

$^1H$ NMR (400 MHz, $CDCl_3$) δ 7.35 (d, J = 8.7 Hz, 2H), 7.27 (d, J = 2.6 Hz, 4H), 7.24 – 7.18 (m, 1H), 6.82 (d, J = 9.2 Hz, 2H), 5.17 (dd, J = 8.9, 4.2 Hz, 1H), 3.01 (dt, J = 14.5, 7.5 Hz, 1H), 2.91 – 2.82 (m, 1H), 2.47 (s, 3H), 2.12 (td, J = 14.0, 8.8 Hz, 1H), 2.02 – 1.90 (m, 1H).
$^{13}C$ NMR (101 MHz, $CDCl_3$) δ 160.2, 160.1, 140.3, 140.1, 129.2, 129.1, 128.4, 128.3, 127.0, 125.8, 125.7, 123.4, 115.8, 115.8, 78.0, 77.6, 47.4, 46.6, 37.1, 36.0, 35.8, 35.0.
$^{19}F$ NMR (376 MHz, $CDCl_3$) δ -61.48, -65.11.
IR ($cm^{-1}$) ν = 2934, 1620, 1519, 1325, 1246, 1106, 1067.
HRMS (ESI) m/z: $[M+H]^+$ calcd for $C_{18}H_{18}NOF_6$ 378.1293, found 378.1334.
In accordance with previous reports.[30]

4-(trifluoromethyl)thiomorpholine 1-oxide (**3j)**

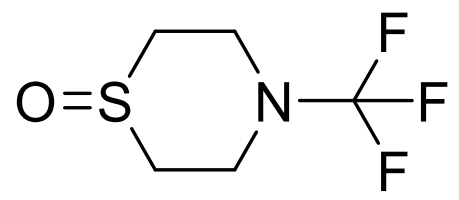


**3j**

Obtained following the general procedure **2** after 24 F $mol^{-1}$ as a brown liquid (17.5 mg, 0.092 mmol, 23%).

$^1H$ NMR (400 MHz, $CDCl_3$) δ 3.61 (t, *J* = 12.6 Hz, 2H), 3.29 – 3.20 (m, 2H), 2.98 – 2.69 (m, 4H).
$^{13}C$ NMR (101 MHz, $CDCl_3$) δ 123.96 (q, $J_{C-F}$ = 256.5 Hz), 44.75, 35.41 (q, $J_{C-F}$ = 2.9 Hz).
$^{19}F$ NMR (376 MHz, $CDCl_3$) δ -66.14.
IR ($cm^{-1}$) ν = 2980, 2929, 1786, 1430, 1293, 1187, 1128, 1058, 1016, 947, 746, 639, 478
HRMS (ESI) m/z: $[M+H]^+$ calcd for $C_5H_9F_3NOS$ 188.0357, found 188.0373.

4-(trifluoromethyl)thiomorpholine 1,1-dioxide (**3k)**

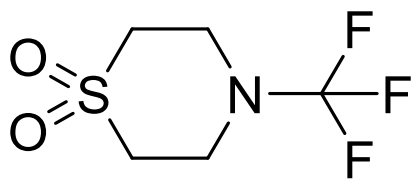

**3k**

4-(trifluoromethyl)thiomorpholine 1,1-dioxide **3k** was obtained following the general procedure **2**, as a brown solid (25 mg, 0.120 mmol, 30%)
$^{1}$H NMR (400 MHz, $CDCl_3$) δ 3.51 (s, 4H), 3.09 (s, 4H).
$^{13}$C NMR (101 MHz, $CDCl_3$) δ 123.7 (q, $J_{C\text{-}F}$ = 257.7 Hz), 51.0, 43.4 (q, $J_{C\text{-}F}$ = 2.8 Hz).
$^{19}$F NMR (376 MHz, $CDCl_3$) δ -64.43.
IR ($cm^{-1}$) ν = 2994, 1776, 1634, 1433, 1279, 1185, 1123, 1052, 954, 871, 746, 670, 571, 475
HRMS (CI) m/z: $[M+H]^+$ calcd for $C_5H_9F_3NO_2S$ 204.0301, found 204.0460 (spectral accuracy: 96.29%).

2-(benzhydryloxy)-N-methyl-N-(trifluoromethyl)ethan-1-amine (**3l)**

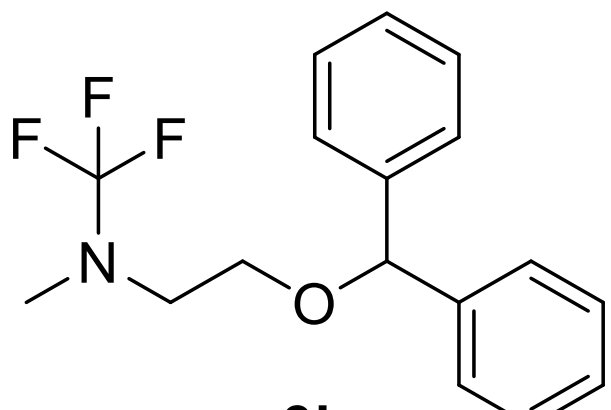

**3l**

2-(benzhydryloxy)-N-methyl-N-(trifluoromethyl)ethan-1-amine **3l** was obtained following the general procedure **2** after 24 F $mol^{-1}$ as a orange liquid (105.1 mg, 0.340 mmol, 85%).

$^{1}$H NMR (500 MHz, $CDCl_3$) δ 7.38 – 7.26 (m, 8H), 7.25 – 7.19 (m, 2H), 5.35 (s, 1H), 3.54 (t, *J* = 5.8 Hz, 2H), 3.05 (t, *J* = 5.7 Hz, 2H), 2.61 (s, 3H).
$^{13}$C NMR (126 MHz, $CDCl_3$) δ 142.2, 128.5, 127.6, 127.0, 126.8 (d, *J* = 7.2 Hz) (poor clarity for quartet), 84.1, 67.4, 48.8 (q, *J* = 2.1 Hz), 34.8 (q, *J* = 3.0 Hz).
$^{19}$F NMR (471 MHz, $CDCl_3$) δ -65.57.
IR ($cm^{-1}$) ν = 2871, 1679, 1453, 1367, 1263.
HRMS (EI) m/z: $[M]^+$ calcd for $C_{17}H_{18}F_3NO$ 309.0760, found 309.1335 (spectral accuracy: 98.06%).

(R)-N-(1-(naphthalen-1-yl)ethyl)-N-(trifluoromethyl)-3-(3-(trifluoromethyl)phenyl)propan-1-amine (**3m)**

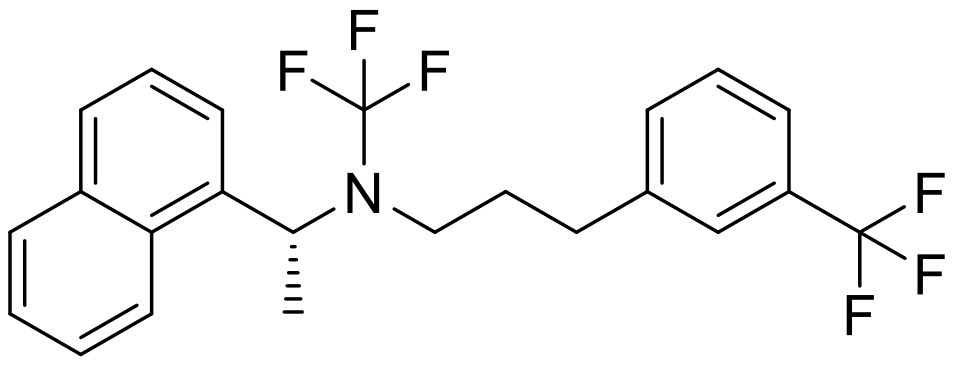

**3m**

(R)-N-(1-(naphthalen-1-yl)ethyl)-N-(trifluoromethyl)-3-(3-(trifluoromethyl)phenyl)propan-1-amine **3m** was obtained following the general procedure 2 after 20 F $mol^{-1}$ as a pale-yellow liquid (22.5 mg, 0.064 mmol, 16%).

$^{1}$H NMR (500 MHz, $CDCl_3$) δ 8.27 (d, *J* = 8.6 Hz, 1H), 7.88 (d, *J* = 8.0 Hz, 1H), 7.82 (d, *J* = 8.0 Hz, 1H), 7.61 – 7.57 (m, 1H), 7.55 – 7.51 (m, 1H), 7.48 – 7.36 (m, 3H),

7.34 – 7.29 (m, 1H), 7.24 – 7.15 (m, 1H), 6.69 (d, *J* = 7.6 Hz, 2H), 5.45 (q, *J* = 6.7 Hz, 1H), 3.01 – 2.61 (m, 4H), 2.06 – 1.98 (m, 2H), 1.66 (d, *J* = 6.9 Hz, 3H).
$^{13}$C NMR (126 MHz, $CDCl_3$) δ 141.6, 134.1, 132.1, 131.9, 131.5, 129.6, 129.1, 128.8, 127.5, 126.4, 125.0, 124.8 (q), 124.7 (d, *J* = 8.7 Hz), 123.3, 123.0 (q, *J* = 5.7 Hz), 52.2, 42.6, 32.7, 30.7, 16.9 (Some -$CF_3$ carbon signals and quartet multiplicity are missing due to low concentration of the sample).
$^{19}$F NMR (471 MHz, $CDCl_3$) δ -58.45, -63.45.
IR (cm$^{-1}$) ν = 2940, 1597, 1501, 1325, 1162, 1072.
HRMS (EI) m/z: $[M]^+$ calcd for $C_{23}H_{21}F_6N$ 425.1220, found 425.1195 (spectral accuracy: 88.99%).

3-((9R,10R)-9,10-ethanoanthracen-9(10H)-yl)-N-methyl-N-(trifluoromethyl)propan-1-amine (**3n**)

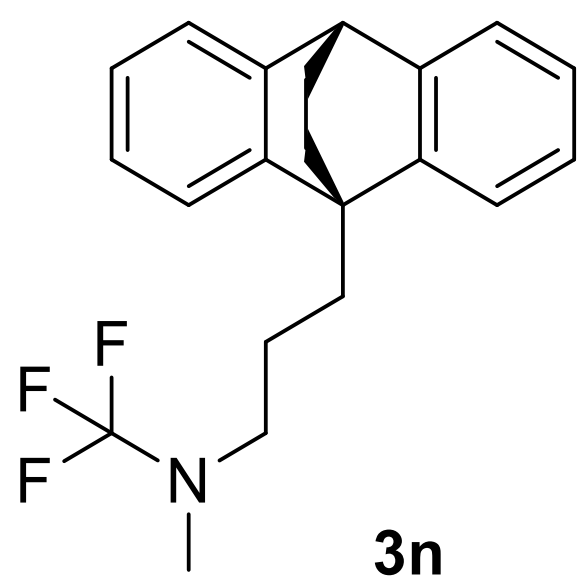

**3n**

Obtained according to the general procedure 2 after 30F as an orange oil (130.4 mg, 0.377 mmol, 95%).

$^{1}$H NMR (500 MHz, $CDCl_3$) δ 7.34 – 7.24 (m, 4H), 7.21 – 7.09 (m, 4H), 4.32 (t, *J* = 2.8 Hz, 1H), 3.13 (t, *J* = 7.2 Hz, 2H), 2.70 (q, *J* = 1.1 Hz, 3H), 2.55 – 2.48 (m, 2H), 2.07 – 1.98 (m, 2H), 1.91 – 1.84 (m, 2H), 1.65 – 1.60 (m, 2H).
$^{13}$C NMR (126 MHz, $CDCl_3$) δ 145.4, 145.1, 125.6 (q, *J* = 254.3 Hz), 125.4, 125.4, 123.5, 121.3, 50.0, 44.8, 44.6, 33.8 (q, *J* = 3.2 Hz), 29.8, 28.3, 27.8, 22.9.
$^{19}$F NMR (471 MHz, $CDCl_3$) δ -66.01.
IR (cm$^{-1}$) ν = 3658, 2978, 2890
HRMS (ESI) m/z: $[M+H]^+$ calcd for $C_{21}H_{23}NF_3$ 346.1783, found 346.1777.

5-((4-(trifluoromethyl)-1,4-diazepan-1-yl)sulfonyl)isoquinoline (**3o**)

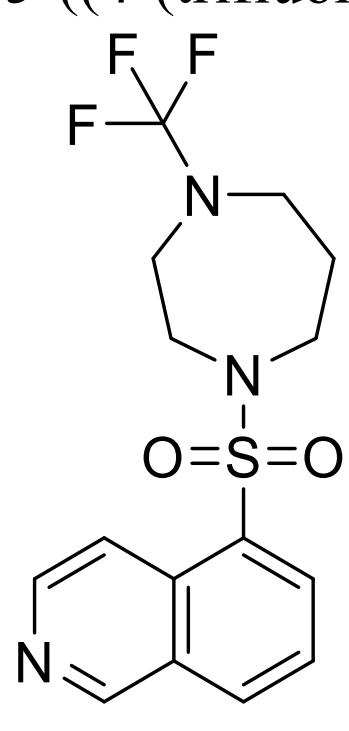

**3o**

Obtained following the general procedure **2** after 20 F mol$^{-1}$ as a pale yellow liquid (10.0 mg, 0.014 mmol, 3.5%).

$^{1}$H NMR (500 MHz, $CDCl_3$) δ 9.37 (s, 1H), 8.71 (d, $J$ = 6.7 Hz, 1H), 8.41 (d, $J$ = 6.2 Hz, 1H), 8.33 (dd, $J$ = 7.4, 1.2 Hz, 1H), 8.24 – 8.18 (m, 1H), 7.73 – 7.67 (m, 1H), 3.53 – 3.46 (m, 4H), 3.23 – 3.18 (m, 4H), 1.97 – 1.89 (m, 2H).
$^{13}$C NMR (101 MHz, $CDCl_3$) δ 153.5, 145.4, 134.5, 133.78, 133.0, 131.7, 129.4, 126.0, 117.6, 50.3, 49.0 (q, $J$ = 3.1 Hz), 47.1, 45.84. [124.4 (q) and 45.7 (q) are missing due to low concentration of the sample].
$^{19}$F NMR (471 MHz, $CDCl_3$) δ -62.82.
IR (cm$^{-1}$) ν = 2982, 1458, 1380, 1288, 1196, 1148.
HRMS (ESI) m/z: $[M+H]^+$ calcd for $C_{15}H_{17}F_3N_3O_2S$ 360.0994, found 360.0992
In accordance with previous reports.[21]

8-chloro-11-(1-(trifluoromethyl)piperidin-4-ylidene)-6,11-dihydro-5H-benzo[5,6]cyclohepta [1,2-b]pyridine (**3p**)

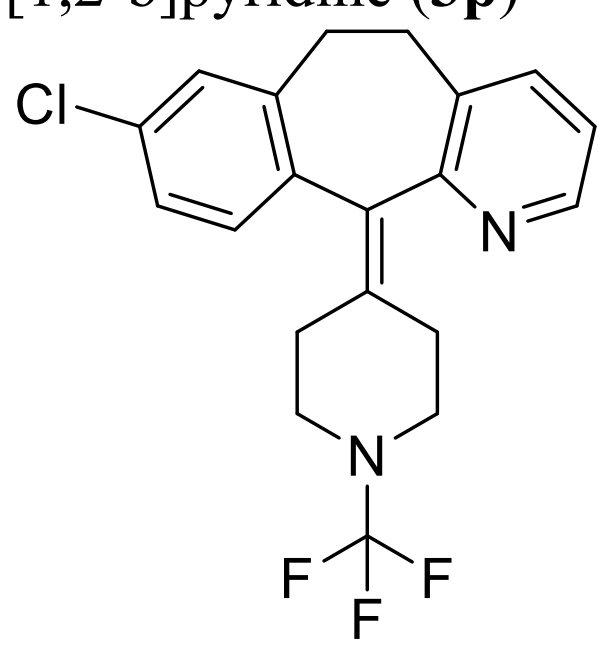


**3p**

Obtained according to the general procedure **2** after 19 F mol$^{-1}$ as an orange oil (76.9 mg, 0.203 mmol, 50%).

$^{1}$H NMR (500 MHz, $CDCl_3$) δ 8.43 – 8.38 (m, 1H), 7.44 (m, 1H), 7.21 – 7.07 (m, 4H), 3.45 – 3.28 (m, 2H), 3.23 – 3.12 (m, 2H), 2.90 – 2.75 (m, 2H), 2.70 – 2.61 (m, 2H), 2.61 – 2.50 (m, 1H), 2.49 – 2.36 (m, 3H).
$^{13}$C NMR (101 MHz, $CDCl_3$) δ 157.0, 146.8, 139.7, 137.7, 137.6, 136.7, 134.4, 133.5, 133.1, 130.6, 129.1, 126.3, 124.9 (q, $J$ = 256.2 Hz),122.5, 45.7 (q, $J$ = 2.9 Hz), 31.8, 31.6, 29.9, 29.6.
$^{19}$F NMR (376 MHz, $CDCl_3$) δ -67.03.
IR (cm$^{-1}$) ν = 3658, 2978, 2891
HRMS (ESI) m/z: $[M+H]^+$ calcd for $C_{20}H_{19}N_2F_3Cl$ 379.1189, found 379.1195.
In accordance with previous reports.[31]

2-(3,4-dimethoxyphenyl)-N-methyl-N-(trifluoromethyl)ethan-1-amine (**3q**)

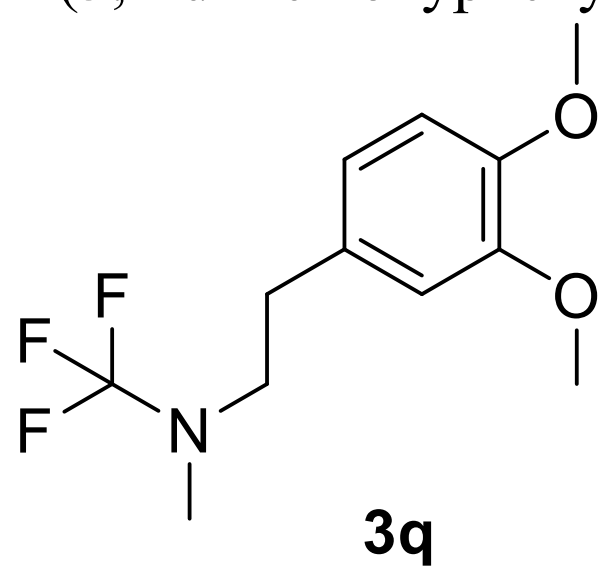


**3q**

Obtained according to the general procedure **2** after 22 F mol$^{-1}$ as an orange oil (49.7 mg, 0.189 mmol, 45%).

$^{1}H$ NMR (500 MHz, $CDCl_3$) δ 6.80 (d, *J* = 8.1 Hz, 1H), 6.75 – 6.70 (m, 2H), 3.88 (s, 3H), 3.86 (s, 3H), 3.05 – 2.99 (m, 2H), 2.79 – 2.74 (m, 2H), 2.57 (q, *J* = 1.1 Hz, 3H).
$^{13}C$ NMR (126 MHz, $CDCl_3$) δ 148.9, 147.7, 131.5, 125.3 (q, *J* = 254.3 Hz), 120.7, 111.9, 111.3, 56.0, 55.9, 50.8, 33.9, 33.8.
$^{19}F$ NMR (471 MHz, $CDCl_3$) δ -64.55.
IR ($cm^{-1}$) ν = 2941, 1511, 1466, 1378, 1220, 1261, 1160, 1062
HRMS (EI) m/z: $[M\text{-}CF_3]^+$, calcd for $C_{11}H_{16}NO_2$ 194.1176, found 194.0840 (spectral accuracy: 98.26%).

(1S,4S)-4-(3,4-dichlorophenyl)-N-methyl-N-(trifluoromethyl)-1,2,3,4-tetrahydronaphthalen-1-amine (**3r**)

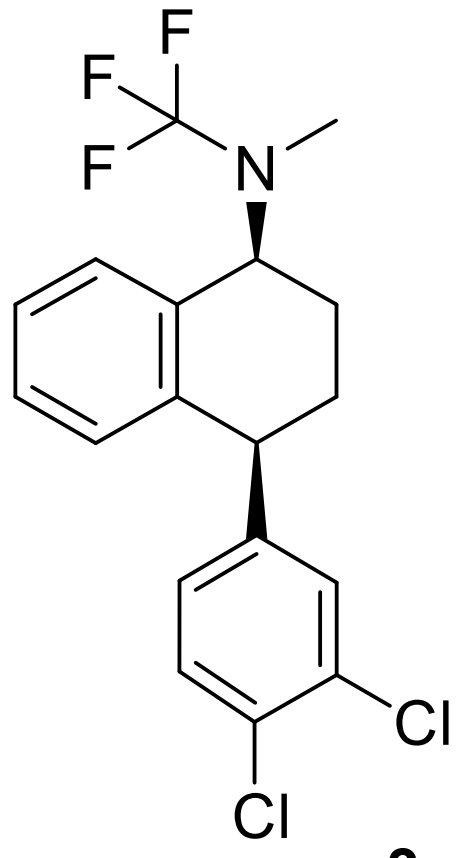


**3r**

Obtained according to the general procedure 2 after 22 F $mol^{-1}$ as an orange oil (61.6 mg, 0.165 mmol, 41%).

$^{1}H$ NMR (500 MHz, $CDCl_3$) δ 7.62 (dt, *J* = 7.9, 1.3 Hz, 1H), 7.36 – 7.28 (m, 2H), 7.21 (tt, *J* = 7.4, 1.1 Hz, 1H), 7.08 (d, *J* = 2.0 Hz, 1H), 6.97 – 6.93 (m, 1H), 6.80 (ddd, *J* = 8.3, 2.2, 0.7 Hz, 1H), 4.60 (dd, *J* = 10.5, 6.2 Hz, 1H), 4.18 (dd, *J* = 5.7, 2.9 Hz, 1H), 2.43 (q, *J* = 1.5 Hz, 3H), 2.31 – 2.14 (m, 1H), 2.06 – 1.98 (m, 1H), 1.90 – 1.74 (m, 2H).
$^{13}C$ NMR (126 MHz, $CDCl_3$) δ 147.1, 138.4, 136.2, 132.4, 130.8, 130.8, 130.3, 130.2, 128.2, 128.0, 127.7, 127.6, 125.5 (q, *J* = 254.3 Hz), 55.3, 43.1, 30.0, 28.3, 20.5.
$^{19}F$ NMR (471 MHz, $CDCl_3$) δ -60.02.
IR ($cm^{-1}$) ν = 2947, 1467,1391, 1315, 1255,1125, 1053, 988, 819, 789, 742
GC-MS (CI) m/z: $[M+H]^+$ calcd for $C_{18}H_{17}Cl_2F_3N$, found 374.0000
In accordance with previous reports.[32]

2-((4-chlorophenyl)((1-(trifluoromethyl)piperidin-4-yl)oxy)methyl)pyridine (**3s**)

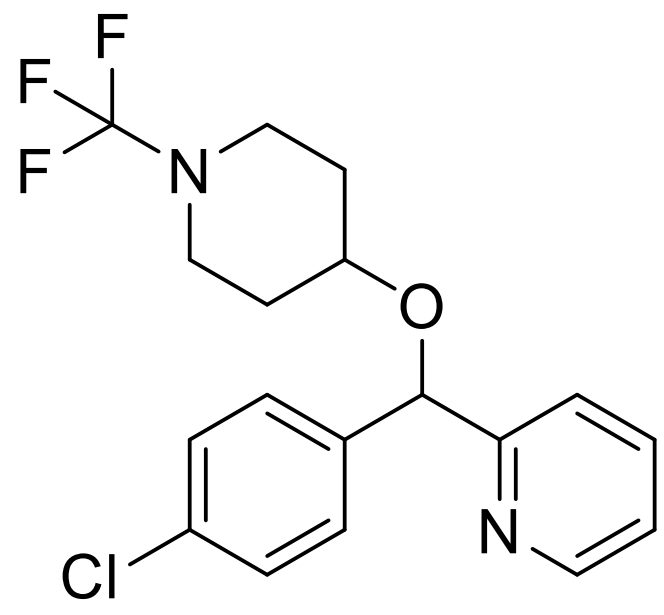

**3s**

Obtained according to the general procedure 2 after 25 F mol$^{-1}$ as an orange oil (80.0 mg, 0.216 mmol, 54%).

$^{1}$H NMR (500 MHz, CHLOROFORM-*D*) δ 8.5 (d, *J* = 3.9 Hz, 1H), 7.7 – 7.6 (m, 1H), 7.5 (d, *J* = 7.5 Hz, 1H), 7.4 (d, *J* = 8.3 Hz, 2H), 7.3 (d, *J* = 7.9 Hz, 2H), 7.2 – 7.1 (m, 1H), 5.6 (s, 1H), 3.6 – 3.5 (m, 1H), 3.2 – 3.1 (m, 2H), 2.8 – 2.7 (m, 2H), 1.9 – 1.8 (m, 2H), 1.8 – 1.7 (m, 2H).

$^{13}$C NMR (126 MHz, CHLOROFORM-*D*) δ 161.8, 149.0, 140.1, 137.1, 133.5, 128.7, 128.2, 124.7 (q, *J* = 255.5 Hz), 122.7, 120.6, 81.2, 71.8, 41.6, 30.2.

$^{19}$F NMR (471 MHz, CHLOROFORM-*D*) δ -67.0.
IR (cm$^{-1}$) ν = 2954, 1589, 1360, 1265, 1092.
HRMS (EI) (m/z): $[M]^+$, calcd for $C_{18}H_{18}ClF_3N_2O$ 370.1068, found 370.1054 (spectral accuracy: 77.26 %)

N-benzyl-1-(2,6-dimethylphenoxy)-N-(trifluoromethyl)propan-2-amine (**3t**)

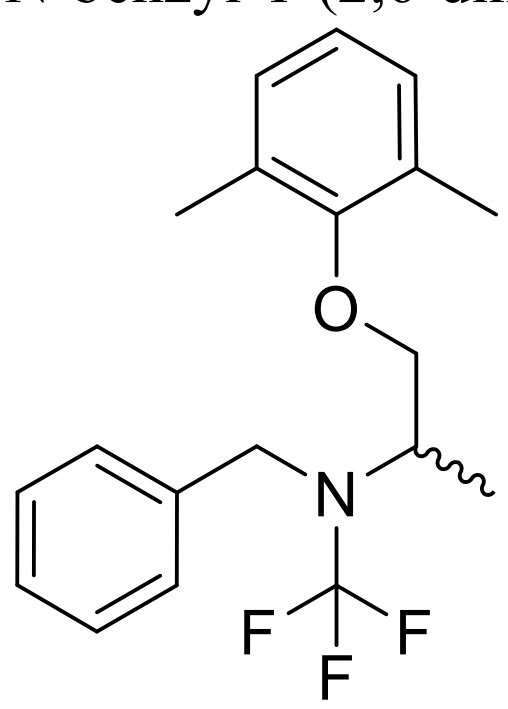

**3t**

Obtained according to the general procedure 2 after 19 F mol$^{-1}$ as an orange oil (NMR yield = 42%).

(3S,4R)-3-((benzo[d][1,3]dioxol-5-yloxy)methyl)-4-(4-fluorophenyl)-1-(trifluoromethyl) piperidine **(3u)**

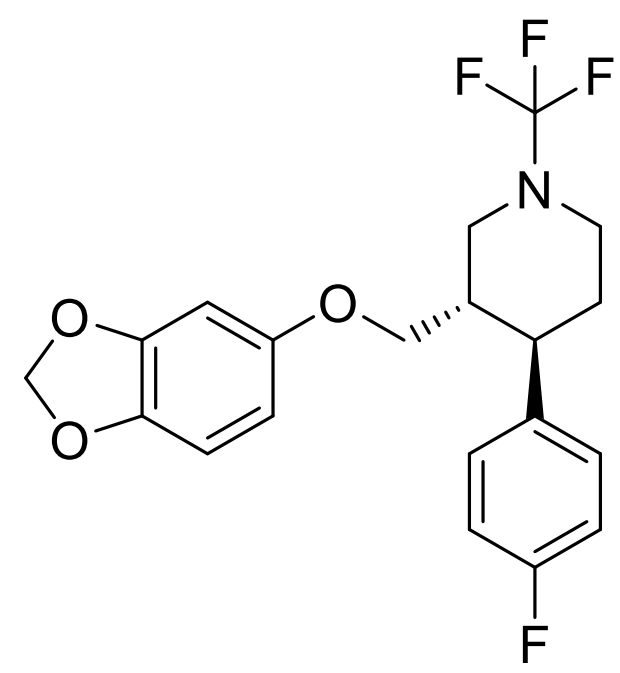

**3u**

Obtained according to the general procedure **2** after 22 F mol$^{-1}$ as an orange oil (NMR yield = 20%).

4-cinnamyl-1-(trifluoromethyl)piperidine (**3v**)

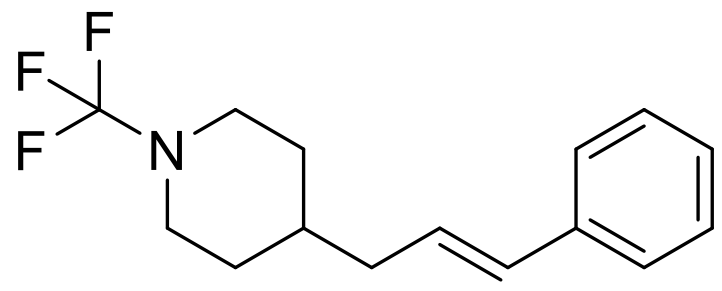

**3v**

Obtained according to the general procedure **2** after 30 F mol$^{-1}$ as an orange oil (NMR yield = 3%).

3-(10,11-dihydro-5H-dibenzo[a,d][7]annulen-5-ylidene)-N-methyl-N-(trifluoromethyl) propan-1-amine (**3w)**

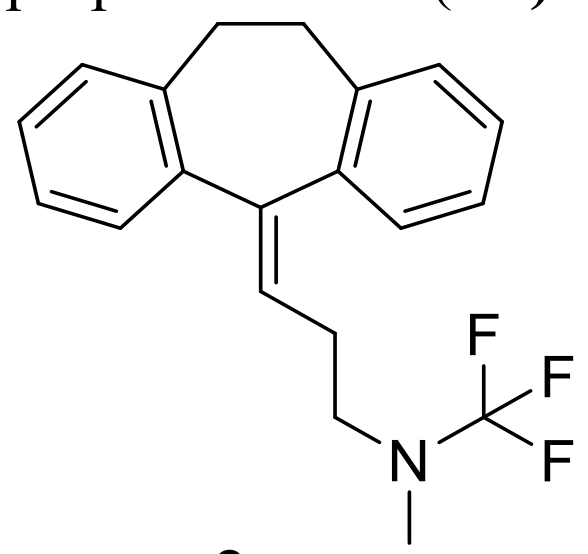

**3w**

Obtained according to the general procedure **2** after 22 F mol$^{-1}$ as an orange oil (NMR yield = 23%).

4-(1-chloro-2-(methyl(trifluoromethyl)amino)ethyl)phenyl acetate **(3x)**

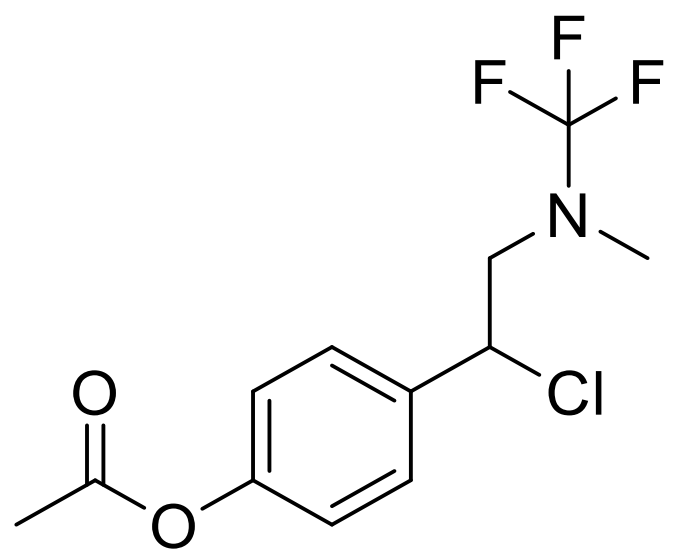

**3x**

Obtained according to the general procedure **2** after 22 F mol$^{-1}$ as an orange oil (NMR yield = 24%).

(1R,5S)-8-(trifluoromethyl)-8-azabicyclo[3.2.1]octan-3-one (**3y**)

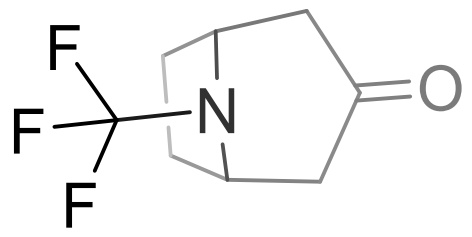

**3y**

Obtained according to the general procedure **2** after 20 F mol$^{-1}$ as an orange oil (NMR yield = 37%).

ethyl-3-(naphthalen-1-yloxy)-3-(thiophen-2-yl)-N-(trifluoromethyl)propan-1-amine (**3z**)

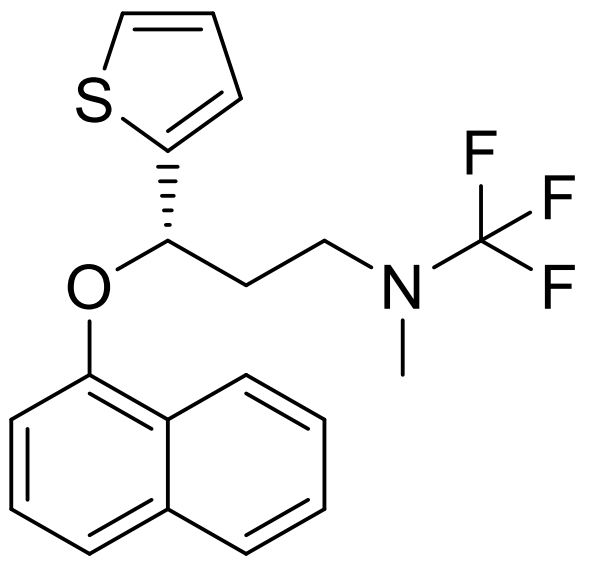

**3z**

Obtained according to the general procedure **2** after 20 F mol$^{-1}$ as an amber oil (NMR yield = 20%).

**Supplementary general procedure 2A:** Into a 5 mL one compartment Electrasyn vial was added ethyl diphenylcarbamodithioate **1aa** (109.0 mg, 0.4 mmol, 1.0 equiv.), acetonitrile (ACN) (5 mL, 0.08 M), $Et_4NBr$ (16.8 mg, 0.08 mmol, 0.2 equiv.) and $Et_3N{\cdot}3HF$ (196 µL, 1.2 mmol, 3.0 equiv.). The solution was electrolyzed at 11.11 mA cm$^{-2}$ for 10 F mol$^{-1}$ (i.e. 5.36 h) using a platinum anode (WE) and platinum cathode (CE). The reaction was monitored by HPLC. After full consumption of the starting ethyl diphenylcarbamodithioate **1aa**, the reaction mixture was quenched with a saturated aqueous solution of $NaHCO_3$ and extracted with DCM. The organic layers were then combined and dried with $Na_2SO_4$. The solvent was removed under reduced pressure. The crude mixture was filtered through silica and purified via flash column chromatography to obtain N-phenyl-N-(trifluoromethyl)aniline (**3aa**) as a colourless liquid in 34% yield (32.2 mg, 0.136 mmol).

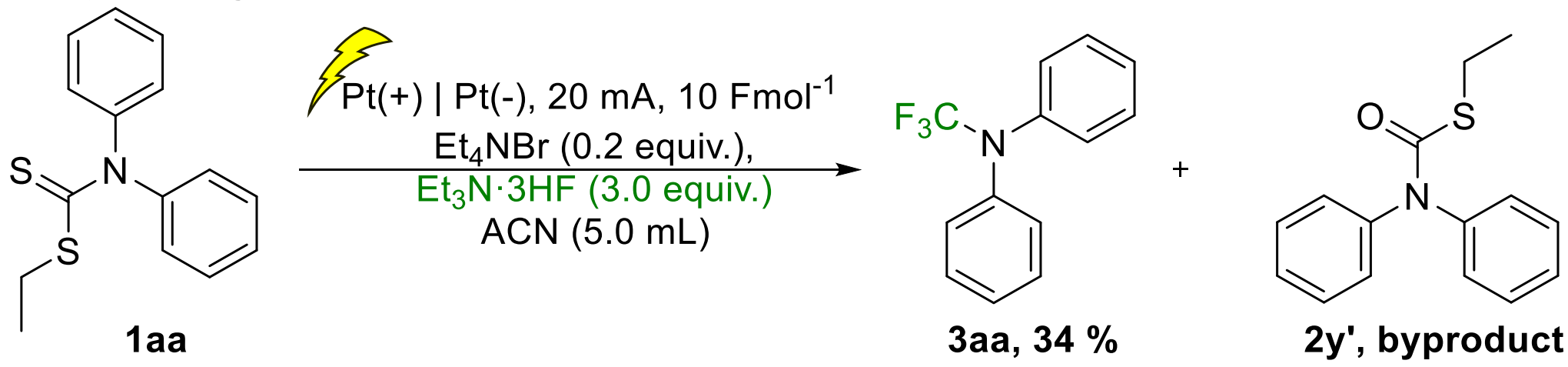


**N-phenyl-N-(trifluoromethyl)aniline (3aa)**

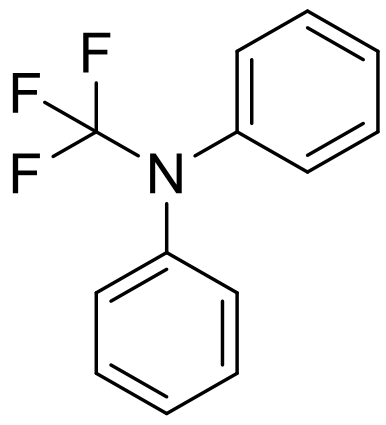

**3aa**

$^{1}$H NMR (500 MHz, $CDCl_3$) δ 7.38 – 7.33 (m, 5H), 7.28 – 7.19 (m, 5H).
$^{13}$C NMR (101 MHz, $CDCl_3$) δ 141.7, 129.4, 126.3, 126.2 (q).
$^{19}$F NMR (376 MHz, $CDCl_3$) δ -54.17.
IR ($cm^{-1}$) ν = 2977, 1493, 1294.
HRMS (ESI) m/z: $[M+H]^+$ calcd for $C_{13}H_{11}F_3N$ 238.0844, found 238.0837

## Supplementary scale up

**Large-scale reaction for synthesis of thiomorpholine-4-carbothioyl fluoride (2c) and N,N-dibenzyl-1,1,1-trifluoromethanamine (3a) (1.0 mmol scale)**

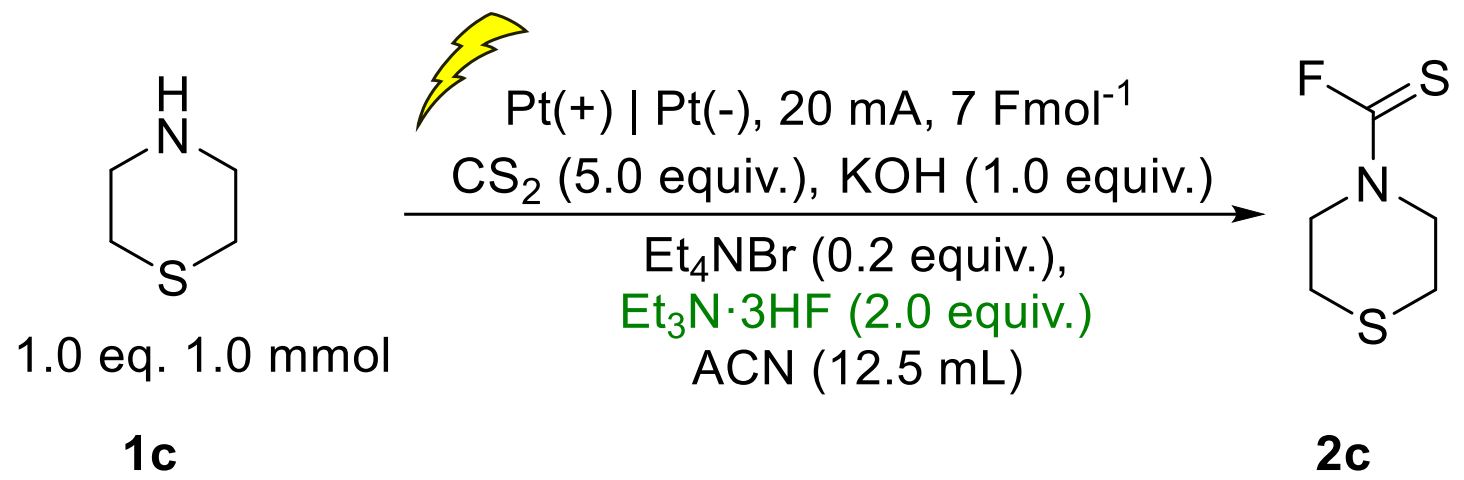


**General procedure 3:** Thiomorpholine (103.0 mg, 1.0 mmol, 1.0 equiv.), KOH (56.0 mg, 1.0 mmol, 1.0 equiv.) and $CS_2$ (381 mg, 5.0 mmol, 5.0 equiv.) was added into a 20 mL one compartment Electrasyn vial to generate in situ salt. Then acetonitrile (ACN) (12.5 mL, 0.08 M), $Et_4NBr$ (42.0 mg, 0.2 mmol, 0.2 equiv.) and $Et_3N{\cdot}3HF$ (326 µL, 2.0 mmol, 2.0 equiv.) were added. The solution was electrolyzed at 11.11 mA $cm^{-2}$ for 7 F $mol^{-1}$ (i.e. 9.375 h) using a platinum anode (WE) and platinum cathode (CE). The reaction was monitored by HPLC. After full consumption of the starting amine, the reaction mixture was quenched with a saturated aqueous solution of $NaHCO_3$ and extracted with DCM. The organic layers were then combined and dried with $Na_2SO_4$. The solvent was removed under reduced pressure. The crude mixture was filtered through silica and the final product thiomorpholine-4-carbothioyl fluoride **2c** obtained in 95% yield (156.8 mg, 0.95 mmol).

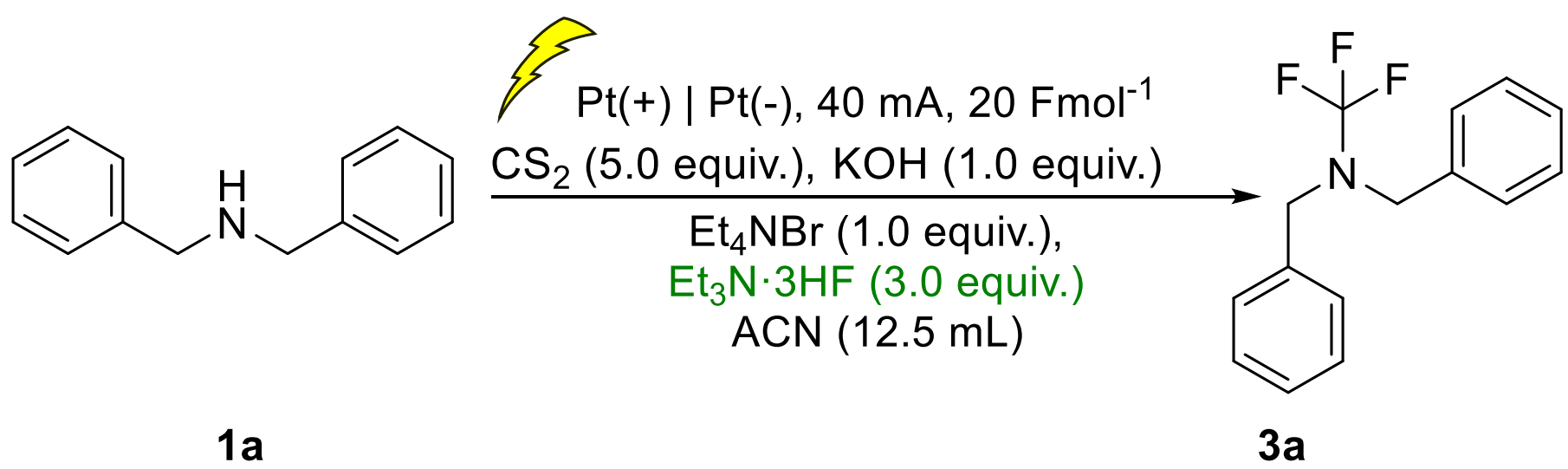


**General procedure 4:** Dibenzylamine (192 µL 1.0 mmol, 1.0 equiv.), KOH (56.0 mg, 1.0 mmol, 1.0 equiv.) and $CS_2$ (382 mg, 5.0 mmol, 5.0 equiv.) was added into a 20 mL one compartment Electrasyn vial to generate in situ salt. Then acetonitrile (ACN) (12.5 mL, 0.08 M), $Et_4NBr$ (210.0 mg, 1.0 mmol, 1.0 equiv.) and $Et_3N{\cdot}3HF$ (489 µL, 3.0 mmol, 3.0 equiv.) was added. The solution was electrolyzed at 22.22 mA $cm^{-2}$ for 20 F $mol^{-1}$ (13 h, 24 mins) using a platinum anode (WE) and platinum cathode (CE). The reaction was monitored by $^{19}$F NMR until full consumption of the NCSF intermediate. The solvent was evaporated, the mixture was

diluted with Hexane and then filtered through celite to obtain the final product N,N-dibenzyl-1,1,1-trifluoromethanamine **3a** in 95% yield (252.0 mg, 0.95 mmol).

**Large-scale reaction for synthesis of thiomorpholine-4-carbothioyl fluoride (2c) (1.0 g scale)**

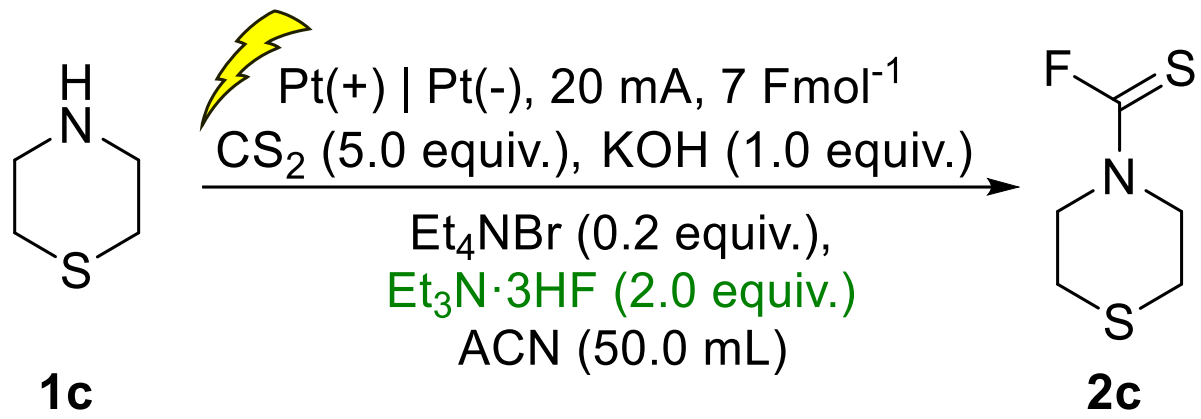


**1c** **2c**

**(A)** **(B)** **(C)** **(D)**

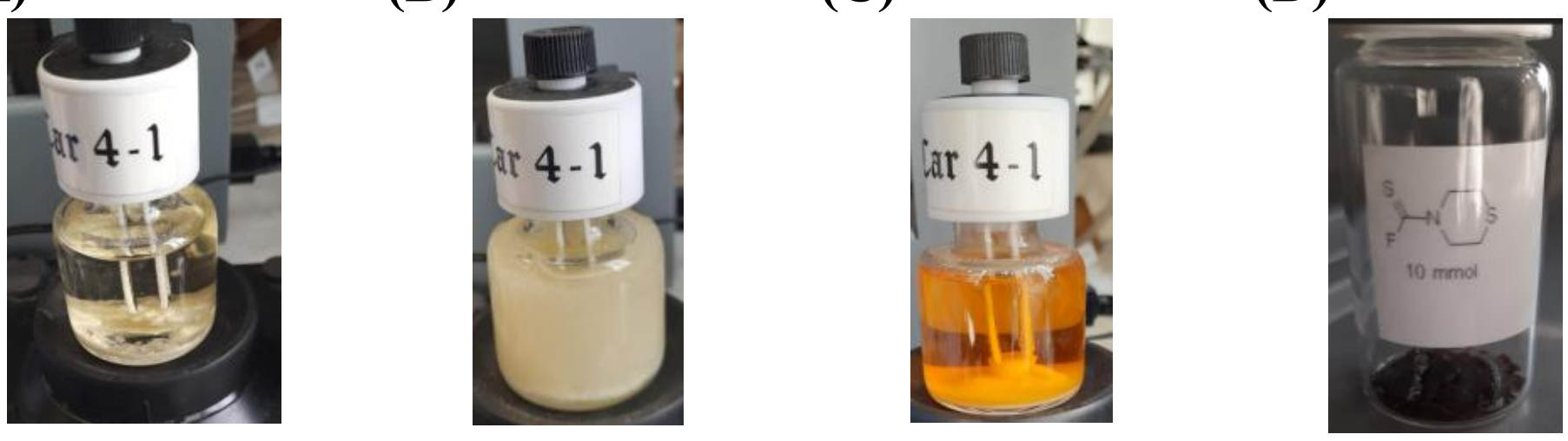

**Supplementary Fig. 3:** Colour change observed during large-scale synthesis of thiomorpholine-4-carbothioyl fluoride (**2c**): **(A)** before the reaction; **(B)** during the reaction; **(C)** after the reaction and **(D)** isolated product.

**General procedure 5:** Thiomorpholine (1.03 g, 10.0 mmol, 1.0 equiv.), KOH (561.0 mg, 10.0 mmol, 1.0 equiv.) and $CS_2$ (3.81 g, 50.0 mmol, 5.0 equiv.) was added into a 50 mL one compartment Electrasyn vial to generate in situ salt. Then acetonitrile (ACN) (50 mL, 0.2 M), $Et_4NBr$ (420 mg, 2.0 mmol, 0.2 equiv.) and $Et_3N \cdot 3HF$ (3.26 mL, 20.0 mmol, 2.0 equiv.) was added. The solution was then electrolyzed with a current density of 11.11 mA cm$^{-2}$ for 7 F mol$^{-1}$ (i.e. 93.75 h) using a platinum anode (WE) and platinum cathode (CE). The reaction was monitored by HPLC. After full consumption of the starting amine, the reaction mixture was quenched with a saturated aqueous solution of $NaHCO_3$ and extracted with DCM. The organic layers were then combined and dried with $Na_2SO_4$. The solvent was removed under reduced pressure. The crude mixture was filtered through silica to obtain the final product thiomorpholine-4-carbothioyl fluoride **2c** as a brown solid in 88% yield (1.45 g, 8.80 mmol).

**Large scale electrochemical preparation of dibenzylcarbamic fluoride (3a) through N,N-dibenzyl-1,1,1-trifluoromethanamine (3a)**

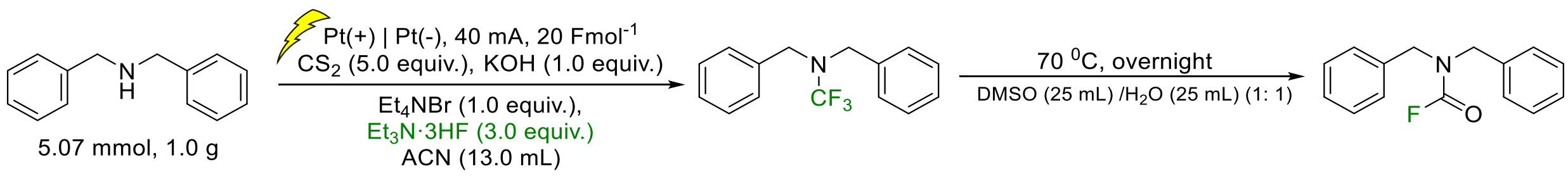


**1a** **3a** **4a**

**General procedure 6:** Dibenzylamine (1.0 g 5.08 mmol, 1.0 equiv.), KOH (285.0 mg, 5.08 mmol, 1.0 equiv.) and $CS_2$ (1.93 g, 25.4 mmol, 5.0 equiv.) was added into a 20 mL one compartment Electrasyn vial to generate in situ salt. Then acetonitrile (ACN) (15.0 mL, 0.34 M), $Et_4NBr$ (1.07 g, 5.08 mmol, 1.0 equiv.) and $Et_3N \cdot 3HF$ (2.48 mL, 15.2 mmol, 3.0

equiv.) was added. The solution was electrolyzed at 22.22 mA $cm^{-2}$ for 20 F $mol^{-1}$ (68 h) using a platinum anode (WE) and platinum cathode (CE). The reaction was monitored by $^{19}F$ NMR until full consumption of the NCSF intermediate. Then, the solvent was evaporated, and the resulting crude mixture was dissolved in a total of 50 mL of a 1:1 mixture of DMSO and $H_2O$ and stirred at 70 ºC. The reaction was monitored by $^{19}F$ NMR. After full consumption of the starting $NCF_3$, more $H_2O$ was added and the crude extracted with DCM. The organic layers were then combined and dried with $Na_2SO_4$. The solvent was removed under reduced pressure. The crude mixture was filtered through silica to obtain the final product dibenzylcarbamic fluoride (**4a**) in 97% yield (1.2 g, 4.93 mmol).

$^1H$ NMR (400 MHz, $CDCl_3$) δ 7.44 – 7.33 (m, 6H), 7.32 – 7.28 (m, 2H), 7.25 – 7.22 (m, 2H), 4.45 (s, 2H), 4.36 (s, 2H).
$^{13}C$ NMR (101 MHz, $CDCl_3$) δ 148.2 (d, $J$ = 288.0 Hz), 135.4 (d, $J$ = 3.4 Hz), 129.0 (d, $J$ = 7.0 Hz), 128.4, 128.2 (d, $J$ = 2.9 Hz), 127.8, 50.5 (d, $J$ = 1.4 Hz), 49.7 (d, $J$ = 3.6 Hz).
$^{19}F$ NMR (376 MHz, $CDCl_3$) δ -22.42.
IR ($cm^{-1}$) ν = 3032, 2934, 1779, 1496, 1419, 1220, 1082, 1037, 956, 806, 746, 696, 618, 555, 456
HRMS (ESI) m/z: $[M+H]^+$ calcd for $C_{15}H_{15}FNO$ 244.1138, found 244.1126

**Large scale (5.0 g) electrochemical preparation of N,N-dibenzyl-1,1,1-trifluoromethanamine**

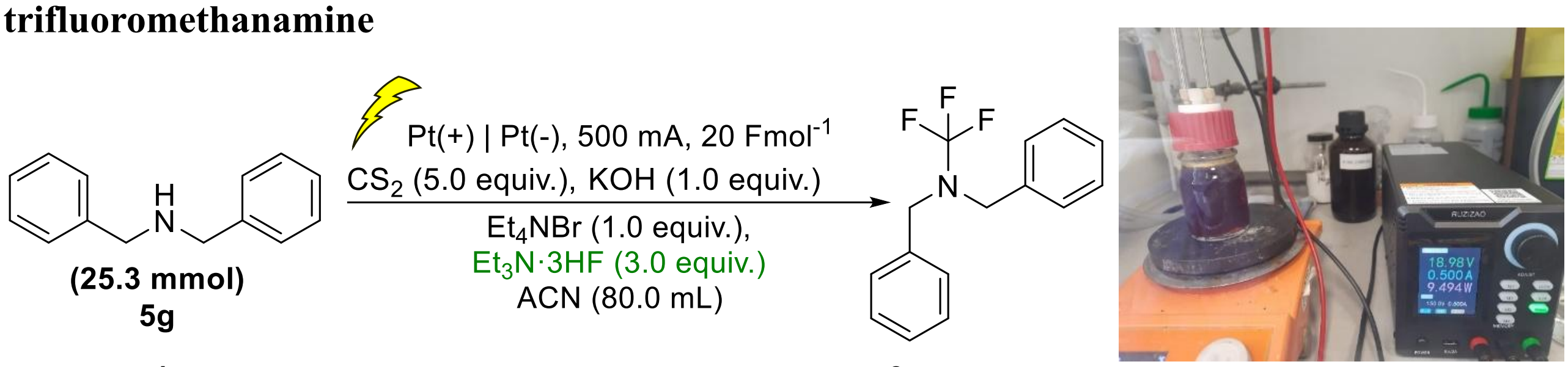


**1a** **3a**

**Supplementary Fig. 4:** Large scale electrochemical reaction set up: 5.0 g scale electrochemical preparation of N,N-dibenzyl-1,1,1-trifluoromethanamine.

**General procedure 7:** Dibenzylamine (4.87 mL, 25.3 mmol, 1.0 equiv.), KOH (1.42 g, 25.3 mmol, 1.0 equiv.) and $CS_2$ (7.62 mL, 127.0 mmol, 5.0 equiv.) was added into a 100 mL one compartment Electrasyn vial to generate in situ salt. Then acetonitrile (ACN) (80.0 mL, 0.32 M), $Et_4NBr$ (5.33 g, 25.3 mmol, 1.0 equiv.) and $Et_3N{\cdot}3HF$ (12.4 mL, 76.0 mmol, 3.0 equiv.) was added. The solution was then electrolyzed at 81.7 mA $cm^{-2}$ for 20 F $mol^{-1}$ (27.23 h) using a platinum anode (WE) and platinum cathode (CE). The reaction was monitored by $^{19}F$ NMR until full consumption of the NCSF intermediate. Then, the solvent was evaporated, the mixture diluted in Hexane and filtered through celite to obtain the final product N,N-dibenzyl-1,1,1-trifluoromethanamine **3a** in 55% yield (3.7 g, 13.92 mmol).

**Supplementary applications**

**Small scale (0.4 mmol) electrochemical preparation of carbamoyl fluorides through trifluoromethylamines:**

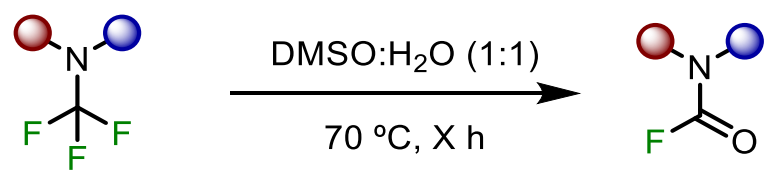


**3** **4**

**General procedure 8:** Amine (0.4 mmol, 1.0 equiv.), KOH (22.4 mg, 0.4 mmol, 1.0 equiv.) and $CS_2$ (152 mg, 2.0 mmol, 5.0 equiv.) was added into a 5.0 mL one compartment Electrasyn vial to generate in situ salt. Then acetonitrile (ACN) (5.0 mL, 0.08 M), $Et_4NBr$ (84.0 mg, 0.4 mmol, 1.0 equiv.) and $Et_3N{\cdot}3HF$ (196 µL, 1.2 mmol, 3.0 equiv.) was added. The solution was then electrolyzed at 22.22 mA $cm^{-2}$ up to 20 F $mol^{-1}$ unless mentioned otherwise using a platinum anode (WE) and platinum cathode (CE). The reaction was monitored by $^{19}F$ NMR until full consumption of the NCSF intermediate. Then, the solvent was evaporated, and the resulting crude was dissolved in a total of 4 mL of DMSO: H2O (1:1) mixture and stirred at 70 ºC. The reaction was monitored by $^{19}F$ NMR. After full consumption of the starting $NCF_3$, more $H_2O$ was added and the crude mixture extracted with DCM. The organic layers were then combined and dried with $Na_2SO_4$. The solvent was removed under reduced pressure. The crude mixture was filtered through silica to obtain the final product.

**6-bromo-3,4-dihydroisoquinoline-2(1H)-carbonyl fluoride (4b' and 4b")**

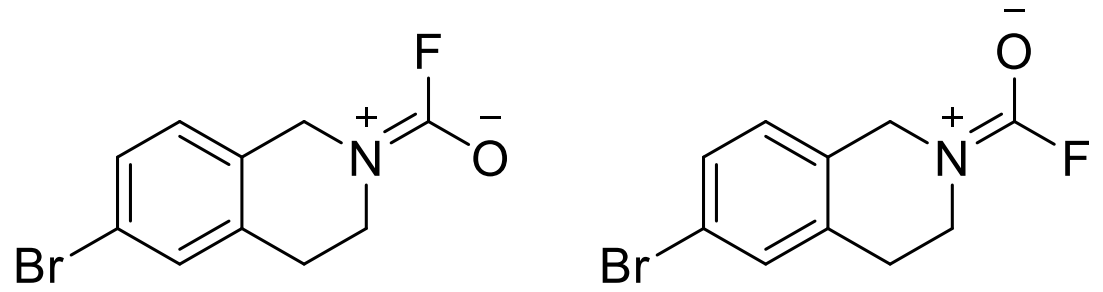

**4b'** **4b"**

Obtained after 4 h following the general procedure 8 as a brown liquid (76.9 mg, 0.298 mmol, 71%) (**4b'**:**4b"**=9:10).

$^1H$ NMR (400 MHz, $CDCl_3$) δ 7.40 – 7.32 (m, 2H+2H, **4b'**+**4b"**), 6.99 (m, 1H+1H, **4b'**+**4b"**), 4.58 (m, 2H+2H, **4b'**+**4b"**), 3.69 (m, 2H+2H, **4b'**+**4b"**), 2.91 (m, 2H+2H, **4b'**+**4b"**).
$^{13}C$ NMR (101 MHz, $CDCl_3$) δ 147.8 (d, $J$ = 8.8 Hz, **4b'**), 145.5 (d, $J$ = 8.5 Hz, **4b"**), 136.1 (**4b'**), 135.8 (**4b"**), 131.8(**4b'**), 131.6 (**4b"**), 130.8 (**4b"**), 130.5 (**4b'**), 130.0 (**4b"**), 129.97 (**4b'**), 128.05 (**4b"**), 127.84(**4b'**), 120.90 (**4b'**), 120.8 (**4b"**), 46.28 (**4b"**), 45.83 (d, $J$ = 4.1 Hz, **4b'**), 42.41 (**4b'**), 42.20 (d, $J$ = 3.7 Hz, **4b"**), 28.64 (**4b"**), 28.09 (**4b'**).
$^{19}F$ NMR (376 MHz, $CDCl_3$) δ -19.95 (**4b'**), -22.96 (**4b"**).
IR ($cm^{-1}$) ν = 3071, 2920, 2861, 1787, 1400, 1264, 1220, 1106, 736.
HRMS (EI) m/z: $[M]^+$ calcd for $C_{10}H_9BrFNO$ 256.9846, found 256.9682 (Spectral accuracy: 98.6).

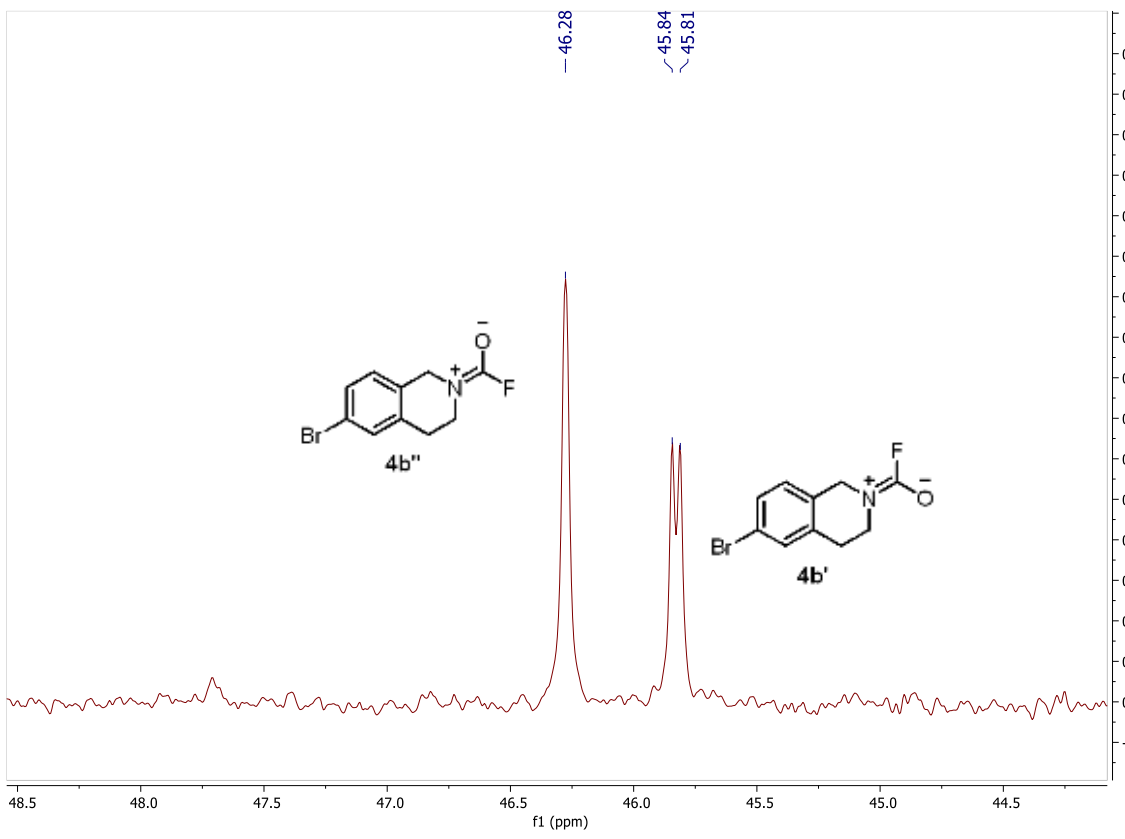

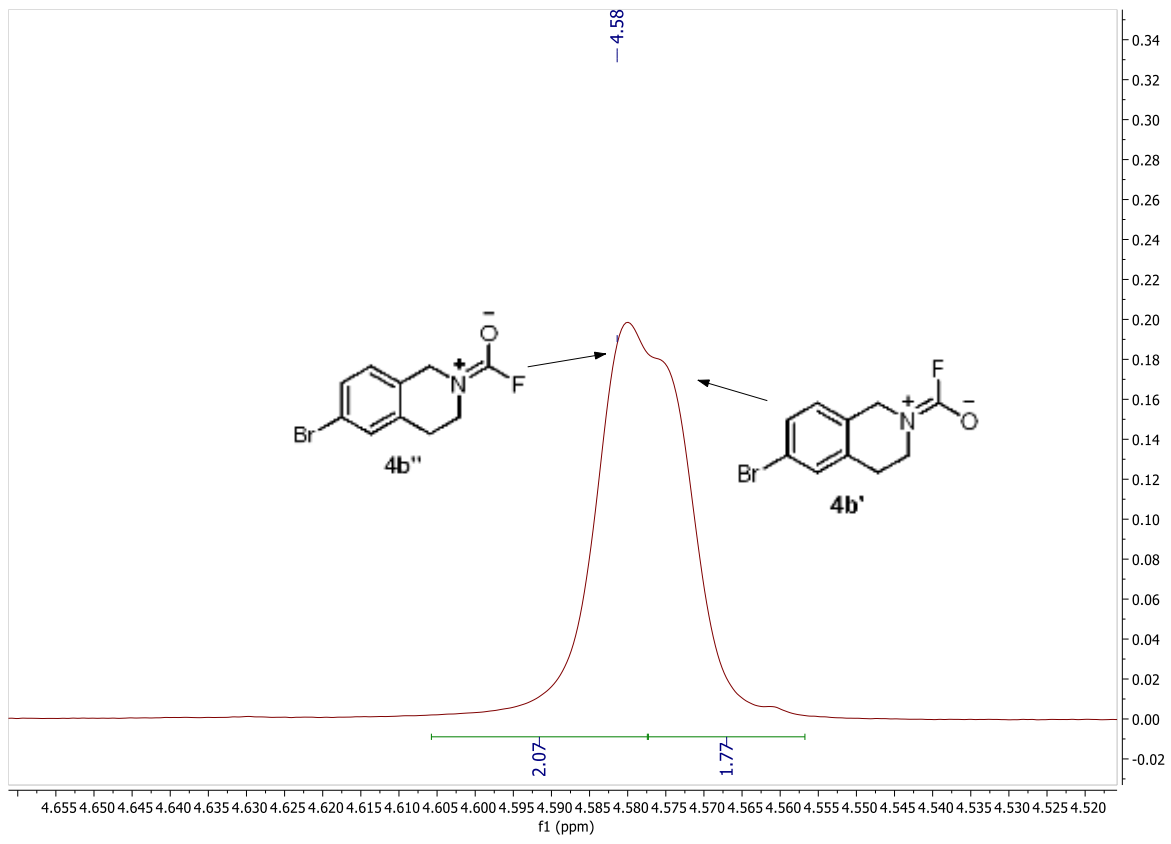


In the $^{13}C$ NMR, the downfield peaks were assigned to **4b”**. Consistent with this assignment, the major rotamer was identified from $^{1}H$ NMR.

**methyl(naphthalen-2-ylmethyl)carbamic fluoride (4c' and 4c'')**

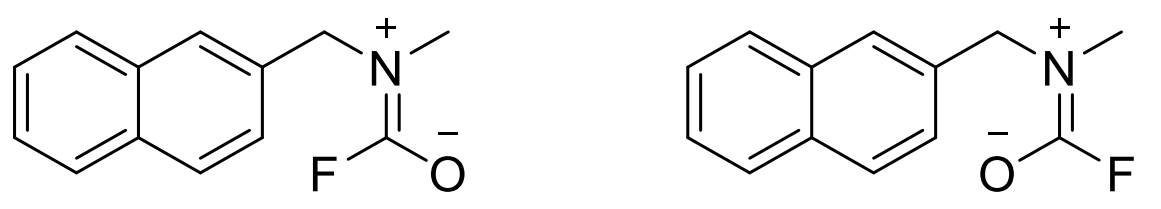

**4c'** **4c"**

Obtained after 28 h following the general procedure 8 as a yellow liquid (54.4 mg, 0.145 mmol, 63%).

$^{1}$H NMR (400 MHz, $CDCl_3$) δ 7.88 – 7.82 (m, 3H+3H, **4c'**+**4c''**), 7.73 (d, J = 1.8 Hz, 1H, **4c''**), 7.71 – 7.66 (m, 1H, **4c'**), 7.56 – 7.48 (m, 2H+2H, **4c'**+**4c''**), 7.42 (dd, J = 8.4, 1.8 Hz, 1H, **4c''**), 7.35 (dd, J = 8.5, 1.8 Hz, 1H, **4c'**), 4.64 (s, 2H, **4c''**), 4.59 (s, 2H, **4c'**), 2.96 (s, 3H, **4c'**), 2.91 (s, 3H, **4c''**).
$^{13}$C NMR (101 MHz, $CDCl_3$) δ 149.3 (d, J = 92.7 Hz, **4c''**), 147.0 (d, J = 92.1 Hz, **4c'**), 133.4, 133.2, 133.1, 132.9, 132.8, 129.1, 129.0, 127.9, 127.3, 126.8, 126.6, 126.5, 126.4, 125.8, 125.2 (aromatic signals, **4c''+4c')**, 53.8 (**4c''**), 53.4 (d, J = 3.6 Hz, **4c'**), 35.1 (**4c'**), 33.9 (d, J = 3.6 Hz, **4c''**).
$^{19}$F NMR (376 MHz, $CDCl_3$) δ -21.48 (**4c''**), -23.23 (**4c'**).
IR ($cm^{-1}$) ν = 3055, 2925, 2854, 1784, 1433, 1409, 1279, 1223, 1114, 750, 475.
HRMS (EI) m/z: $[M]^+$ calcd for $C_{13}H_{12}FNO$ 217.0897, found 217.0833. (Spectral accuracy: 99.2).

**Synthesis of S-phenyl dibenzylcarbamothioate (4d)**

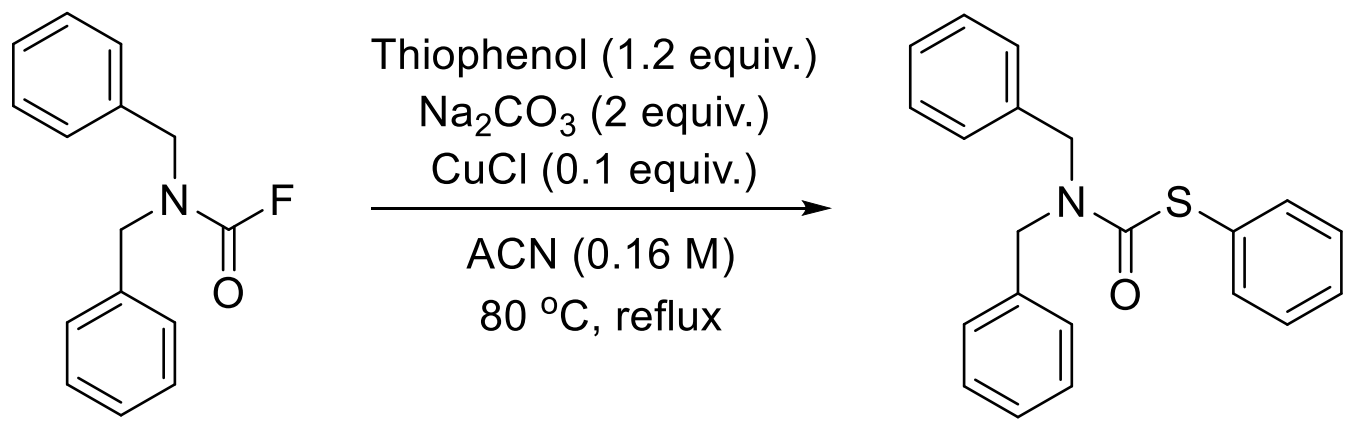


**4a** **4d**

A suspension of dibenzylcarbamic fluoride (198.7 mg, 0.817 mmol, 1.0 equiv.), thiophenol (0.10 mL, 0.974 mmol, 1.2 equiv.), sodium carbonate (176.0 mg, 1.661 mmol, 2.0 equiv.) and copper(I) chloride (9.3 mg, 0.094 mmol, 0.1 equiv.) in acetonitrile (5.0 mL) was heated at 80 °C under reflux. When no further consumption of starting material was observed by HPLC-MS (after ca. 16 hours), the reaction mixture was diluted with water (ca. 10 mL) and brine (ca. 10 mL) and extracted with diethyl ether. The organic layers were combined and dried over $Na_2SO_4$, and the solvent was removed under reduced pressure. The pure product was obtained after flash column chromatography (Hex:EtOAc 1:0 to 19:1) as a colourless liquid (84.1 mg, 0.252 mmol, 31%).

$^{1}$H NMR (400 MHz, DMSO-$D_6$, 100 °C) δ 7.55 – 7.49 (m, 2H), 7.46 – 7.40 (m, 3H), 7.40 – 7.26 (m, 10H), 4.61 (s, 4H).
$^{13}$C NMR (101 MHz, DMSO-$D_6$, 100 °C) δ 165.68, 135.99, 134.65, 128.47, 128.35, 128.22, 128.04, 127.04, 126.91, 50.12.
IR ($cm^{-1}$) ν = 1662, 1398, 1178
HRMS (ESI) m/z: $[M+H]^+$ calcd for $C_{21}H_{20}NOS$ 334.1266, found 334.1273.

**Synthesis of dimethyl 2-(thiomorpholine-4-carbonothioyl)malonate (5a)**

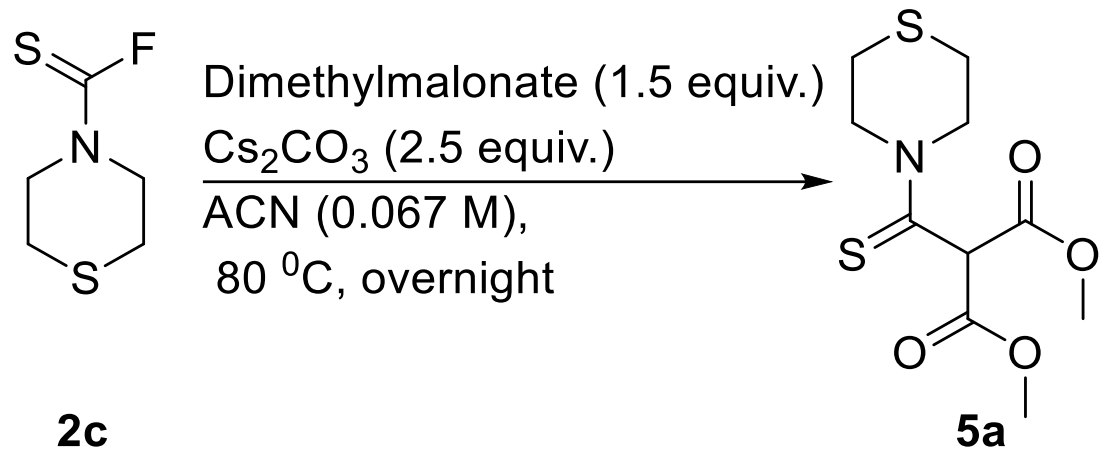


According to a modified reported procedure[33], to a solution of dimethyl malonate (159.0 mg, 1.2 mmol, 1.5 equiv.) and $Cs_2CO_3$ (652 mg, 2.0 mmol, 2.5 equiv.) in ACN (12 mL, 0.067M) was added thiomorpholine-4-carbothioyl fluoride (**2c**, 132.0 mg, 0.8 mmol, 1.0 equiv.). The mixture was stirred overnight at 80 °C in oil bath until the thiocarbamoyl fluorides **2c** was consumed completely. To this reaction mixture water was added and then extracted with DCM (dichloromethane) (3×). The combined organic extract was dried ($MgSO_4$) and concentrated in vacuo. The residue was purified by column chromatography (eluent: petroleum ether/DCM) to give the final product dimethyl 2-(thiomorpholine-4-carbonothioyl)malonate **5a** as a colourless liquid in 75% yield (166 mg, 0.6 mmol).

$^1$H NMR (400 MHz, $CDCl_3$) δ 5.12 (s, 1H), 4.60 – 4.56 (m, 2H), 3.95 – 3.88 (m, 2H), 3.82 (s, 6H), 2.84 – 2.77 (m, 2H), 2.73 – 2.66 (m, 2H).
$^{13}$C NMR (101 MHz, $CDCl_3$) δ 190.2, 165.6, 65.4, 54.00, 53.6, 53.4, 27.8, 27.2.
IR (cm$^{-1}$) ν = 2951, 2921, 1739, 1480, 1433, 1293, 1237, 1190, 1038, 952, 760, 699
HRMS (ESI) m/z: $[M+H]^+$ calcd for $C_{10}H_{16}NO_4S_2$ 278.0521, found 278.0528

**Synthesis of phenyl(thiomorpholino)methanethione (5b)**

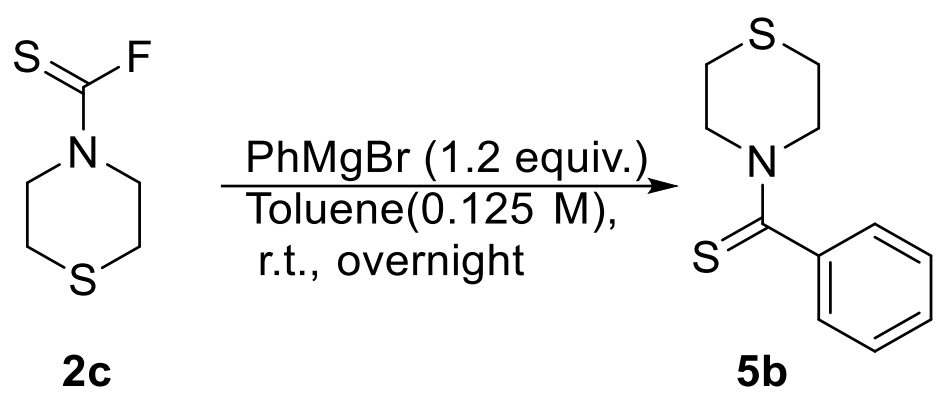


According to a modified reported procedure[30], to an oven dried 25 mL round bottom flask, thiomorpholine-4-carbothioyl fluoride (**2c**,132.0 mg, 0.8 mmol, 1.0 equiv.), and toluene (6.4 mL, 0.125 M), were added. Then to the same flask, 3 M phenyl magnesium bromide in diethyl ether (320 µL, 0.96 mmol, 1.2 equiv.) was added under inert atmosphere. The reaction mixture was then allowed to stir overnight and monitored through TLC (Thin Layer Chromatography) for reaction completion. The reaction was then quenched with saturated aqueous solution of ammonium chloride solution (6.4 mL) and the layers were separated by solvent extraction technique. The aqueous layer was extracted with DCM (dichloromethane) (3×). The combined organic extract was dried ($MgSO_4$) and concentrated in vacuo. The residue was purified by column chromatography (eluent: petroleum ether/DCM) to give the final product phenyl(thiomorpholino)methanethione **5b** as a yellow liquid in 38% yield (68 mg, 0.304 mmol).

$^1$H NMR (400 MHz, $CDCl_3$) δ 7.39 – 7.30 (m, 3H), 7.28 – 7.19 (m, 2H), 4.67 (t, 2H), 3.84 (t, 2H), 2.89 (t, 2H), 2.59 (t, 2H).
$^{13}$C NMR (101 MHz, $CDCl_3$) δ 201.7, 143.1, 128.8, 128.7, 125.6, 54.6, 52.0, 28.6, 27.4.
IR (cm$^{-1}$) ν = 2915, 1746, 1472, 1434, 1296, 1236, 1189, 1039, 951, 758, 698
HRMS (ESI) m/z: $[M+H]^+$ calcd for $C_{11}H_{14}NS_2$ 224.0568, found 224.0566

**Synthesis of thiocarbamate ester derivatives**

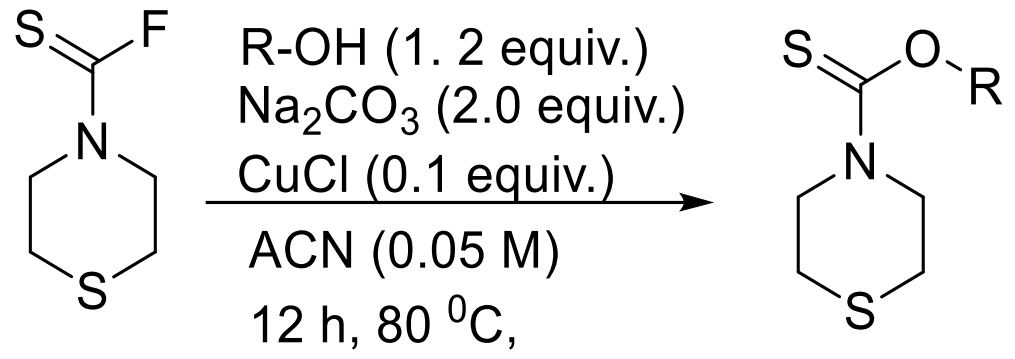


**2c** **7**

According to a modified reported procedure,[34] thiomorpholine-4-carbothioyl fluoride (**2c**, 132.0 mg, 0.8 mmol, 1.0 equiv.), alcohols (1.6 mmol, 2.0 equiv.), CuCl (7.92 mg, 0.08 mmol, 0.1 equiv.), $Na_2CO_3$ (170 mg, 1.6 mmol, 2.0 equiv.) and ACN (16.0 mL, 0.05 M) were added to a 25 mL round bottom flask. The reaction mixture was stirred at 80 °C for 12 h and monitored through TLC (Thin Layer Chromatography) for reaction completion. When the reaction completed, the mixture was quenched with water and extracted with DCM (3 × 20 mL). The organic layers were then combined and concentrated in vacuo. The product was purified by flash column chromatography (petroleum ether/DCM) to obtain the final product.

**O-benzyl thiomorpholine-4-carbothioate (7a)**

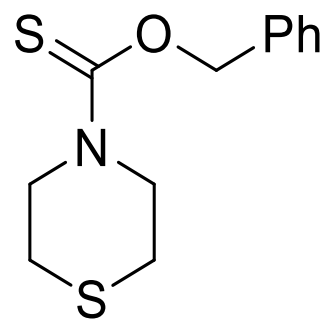

**7a**

O-benzyl thiomorpholine-4-carbothioate **7a** was obtained following the above general procedure with benzyl alcohol (173.0 mg) as a pale-yellow liquid (170.0 mg, 0.672 mmol, 84%).

$^1$H NMR (400 MHz, $CDCl_3$) δ 7.40 – 7.30 (m, 5H), 5.53 (s, 2H), 4.38 (t, 2H), 4.00 (t, 2H), 2.72 (t, $J$ = 5.2 Hz, 2H), 2.57 (t, $J$ = 5.2 Hz, 2H).
$^{13}$C NMR (101 MHz, $CDCl_3$) δ 187.12, 135.75, 128.52, 128.25, 128.06, 73.04, 52.71, 48.24, 27.41, 26.83.
IR (cm$^{-1}$) ν = 3031, 2916, 1719, 1482, 1424, 1366, 1291, 1223, 1137, 1048, 951, 859, 699, 597, 520, 452
HRMS (ESI) m/z: $[M+H]^+$ calcd for $C_{12}H_{16}NOS_2$ 254.0673, found 254.0667

**(E)-O-(3,7-dimethylocta-2,6-dien-1-yl) thiomorpholine-4-carbothioate (7b)**

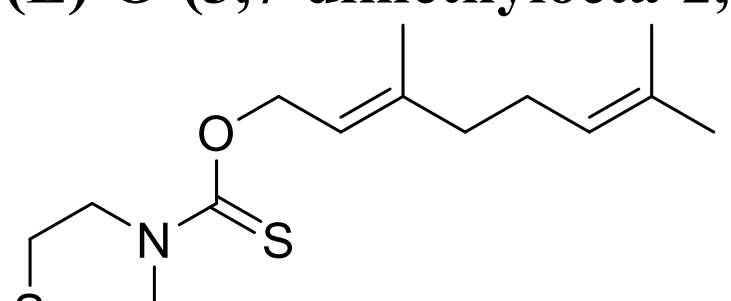

**7b**

(E)-O-(3,7-dimethylocta-2,6-dien-1-yl) thiomorpholine-4-carbothioate **7b** was obtained following the above general procedure as geraniol (247.0 mg) as a yellow liquid (179.3 mg, 0.60 mmol, 75%).

$^{1}H$ NMR (500 MHz, $CDCl_3$) δ 6.12 (dd, $J$ = 17.4, 10.7 Hz, 1H), 5.29 – 5.07 (m, 2H), 5.10 – 5.06 (m, 1H), 3.81 – 3.75 (m, 4H), 2.64 – 2.55 (m, 4H), 2.07 – 1.79 (m, 4H), 1.66 (s, 3H), 1.59 (s, 6H).
$^{13}C$ NMR (126 MHz, $CDCl_3$) δ 166.7, 143.0, 132.0, 123.9, 113.7, 54.7, 40.3, 27.5, 25.8, 23.8, 23.5, 17.8.
IR ($cm^{-1}$) ν = 2968, 2914, 1645, 1400, 1288, 1165, 1080, 1024, 947, 859, 666
HRMS (ESI) m/z: $[M+H]^+$ calcd for $C_{15}H_{26}NOS_2$ 300.1456, found 300.1454

**O-(2-isopropyl-5-methylcyclohexyl) thiomorpholine-4-carbothioate (7c)**

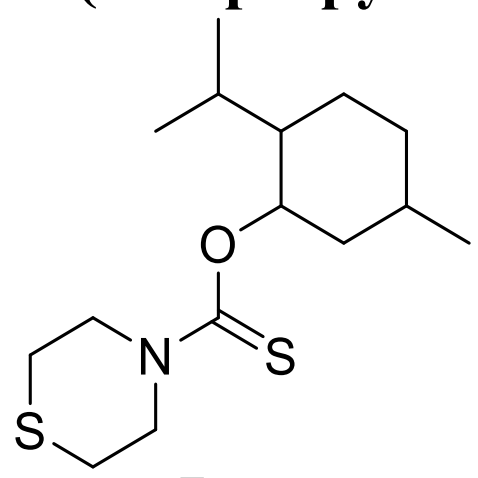

**7c**

O-(2-isopropyl-5-methylcyclohexyl) thiomorpholine-4-carbothioate **7c** was obtained following the general procedure with menthol (250.0 mg) as a colourless liquid (185.5 mg, 0.616 mmol, 77%).

$^{1}H$ NMR (400 MHz, $CDCl_3$) δ 5.31 (td, $J$ = 10.8, 4.5 Hz, 1H), 4.37 (d, $J$ = 3.7 Hz, 2H), 4.02 – 3.95 (m, 2H), 2.71 (d, $J$ = 4.3 Hz, 2H), 2.64 – 2.50 (m, 2H), 2.24 – 2.17 (m, 1H), 1.90 – 1.74 (m, 1H), 1.73 – 1.63 (m, 2H), 1.56 – 1.45 (m, 2H), 1.18 – 1.04 (m, 1H), 0.96 – 0.81 (m, 8H), 0.80 (d, 3H).
$^{13}C$ NMR (101 MHz, $CDCl_3$) δ 187.1, 82.0, 52.7, 48.2, 47.6, 40.7, 34.4, 31.3, 27.6, 26.9, 26.8, 23.7, 22.1, 20.8, 17.1.
IR ($cm^{-1}$) ν = 2953, 1734, 1478, 1421, 1367, 1293, 1221, 1139, 1044, 951, 837, 699, 596, 480
HRMS (ESI) m/z: $[M+H]^+$ calcd for $C_{15}H_{28}NOS_2$ 302.1612, found 302.1606

**O-(3,7-dimethyloct-6-en-1-yl) thiomorpholine-4-carbothioate (7d)**

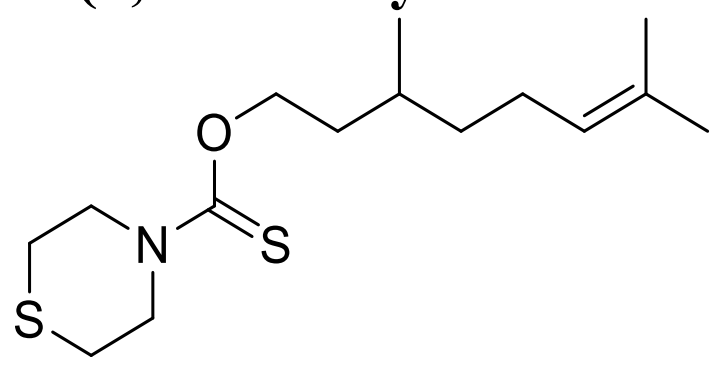

**7d**

O-(3,7-dimethyloct-6-en-1-yl) thiomorpholine-4-carbothioate **7d** was obtained following the general procedure with β-citronellol (125.0 mg) as a yellow liquid (170.0 mg, 0.565 mmol, 70.6%)
$^{1}H$ NMR (500 MHz, $CDCl_3$) δ 5.07 – 4.99 (m, 1H), 4.54 – 4.41 (m, 2H), 4.38 – 4.28 (m, 2H), 4.00 – 3.90 (m, 2H), 2.72 – 2.63 (m, 2H), 2.60 – 2.51 (m, 2H), 2.03 – 1.86 (m, 2H), 1.79 – 1.64 (m, 1H), 1.63 (s, 3H), 1.56 (s, 3H), 1.54 – 1.45 (m, 2H), 1.38 – 1.27 (m, 1H), 1.22 – 1.11 (m, 1H), 0.89 (d, $J$ = 6.6 Hz, 3H).
$^{13}C$ NMR (126 MHz, $CDCl_3$) δ 187.5, 131.3, 124.5, 70.4, 52.5, 48.1, 36.9, 35.5, 29.5, 27.4, 26.9, 25.7, 25.4, 19.5, 17.7.
IR ($cm^{-1}$) ν = 2913, 1480, 1421, 1292, 1196, 1050, 949, 829, 646, 582, 481
HRMS (ESI) m/z: $[M+H]^+$ calcd for $C_{15}H_{28}NOS_2$ 302.1612, found 302.1611

**Synthesis of O-(4-allyl-2-methoxyphenyl) thiomorpholine-4-carbothioate (7e)**

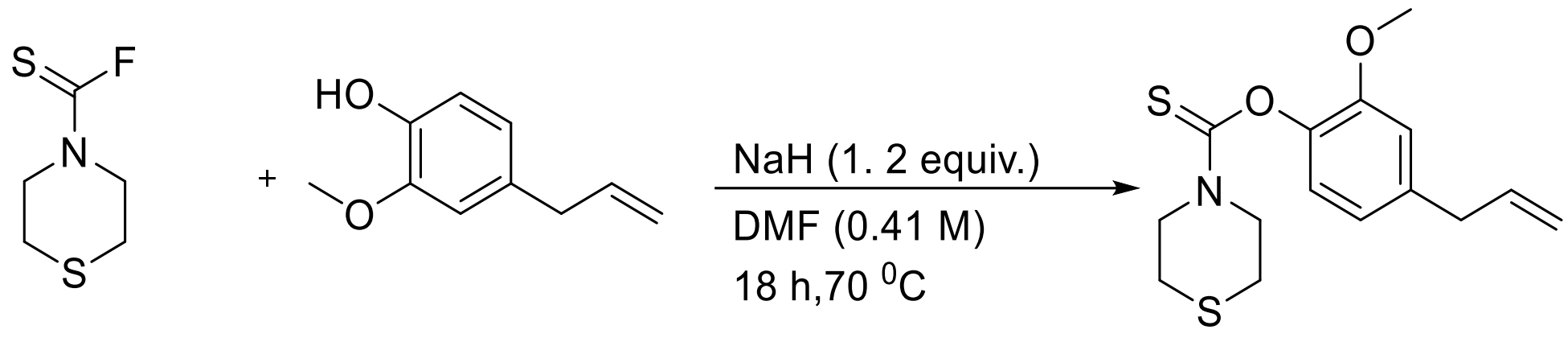


**2c** **7e**

According to a modified reported procedure[35] O-(4-allyl-2-methoxyphenyl) thiomorpholine-4-carbothioate **7e** was obtained in 78% yield (192.9 mg, 0.624 mmol).

$^1$H NMR (400 MHz, $CDCl_3$) δ 6.94 (t, *J* = 7.9 Hz, 1H), 6.86 – 6.75 (m, 2H), 6.04 – 5.90 (m, 1H), 5.15 – 5.04 (m, 2H), 4.44 – 4.37 (m, 2H), 4.25 – 4.18 (m, 2H), 3.79 (s, 3H), 3.39 (d, *J* = 6.8 Hz, 2H), 2.83 – 2.71 (m, 4H).
$^{13}$C NMR (101 MHz, $CDCl_3$) δ 187.4, 151.0, 140.9, 139.0, 136.9, 123.5, 120.5, 116.2, 112.8, 55.9, 53.43, 49.5, 40.1, 27.5, 26.8.
IR (cm$^{-1}$) ν = 2913, 1603, 1489, 1420, 1291, 1267, 1225, 1204, 1173, 1150, 1034, 952, 817, 733, 677, 542, 458
HRMS (ESI) m/z: $[M+H]^+$ calcd for $C_{15}H_{20}NO_2S_2$ 310.0935, found 310.0943

**Synthesis of thiourea derivatives**

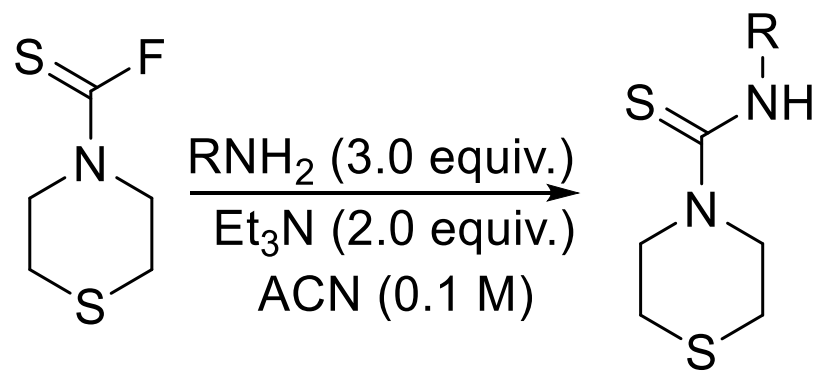


**2c** **8**

According to our previously reported procedure,[36] thiomorpholine-4-carbothioyl fluoride (**2c**, 132.0 mg, 0.8 mmol, 1.0 equiv.), amines (2.4 mmol, 3.0 equiv.) and triethylamine ($Et_3N$, 162 mg, 1.6 mmol, 2.0 equiv.) were added to a round bottom flask and stirred overnight. Reaction completion was monitored through TLC. Once the reaction was completed, HCl 1M was added and extracted with ethyl acetate three times. The organic layers were then combined and concentrated in vacuo. The product was purified by flash column chromatography (petroleum ether/ethyl acetate) to obtain the final product.

**N-benzylthiomorpholine-4-carbothioamide (8a)**

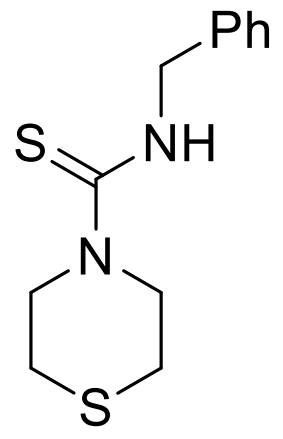

**8a**

N-benzylthiomorpholine-4-carbothioamide **8a** was obtained following the above general procedure with benzyl amine (257.0 mg) as a colourless liquid (200.0 mg, 0.792 mmol, 99%).

$^{1}$H NMR (400 MHz, $CDCl_3$) δ 7.37 – 7.23 (m, 5H), 5.75 (s, 1H), 4.84 (d, *J* = 4.9 Hz, 2H), 4.15 – 4.08 (m, 4H), 2.68 – 2.61 (m, 4H).
$^{13}$C NMR (101 MHz, $CDCl_3$) δ 181.6, 137.9, 128.9, 128.0, 127.8, 50.8, 50.4, 26.7.
IR (cm$^{-1}$) ν = 3244, 2913, 1532, 1482, 1401, 1325, 1182, 944, 850, 737, 694, 658, 518, 477, 451
HRMS (ESI) m/z: $[M+H]^+$ calcd for $C_{12}H_{17}N_2S_2$ 253.0833, found 253.0836

**N-(3,4-dihydroxyphenethyl)thiomorpholine-4-carbothioamide (8b)**

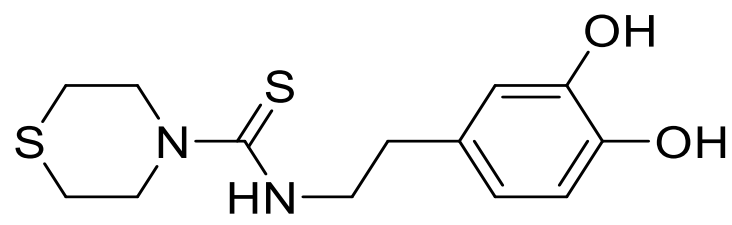

**8b**

N-(3,4-dihydroxyphenethyl)thiomorpholine-4-carbothioamide **8b** was obtained following the above general procedure with dopamine hydrochloric salt (455.0 mg) and and triethylamine ($Et_3N$, 405 mg, 4.0 mmol, 5.0 equiv.) as a colourless liquid (126.4 mg, 0.424 mmol, 53%).

$^{1}$H NMR (400 MHz, $CDCl_3$) δ 6.81 (d, *J* = 8.0 Hz, 1H), 6.72 (s, 1H), 6.61 (dd, *J* = 8.0, 2.1 Hz, 1H), 5.47 (t, *J* = 5.3 Hz, 1H), 4.09 – 4.00 (m, 4H), 3.86 (q, *J* = 6.8 Hz, 2H), 2.82 (t, *J* = 6.8 Hz, 2H), 2.66 – 2.56 (m, 4H).
$^{13}$C NMR (101 MHz, $CDCl_3$) δ 181.2, 144.2, 142.6, 131.6, 121.2, 115.8, 115.7, 50.7, 47.1, 34.4, 26.6.
IR (cm$^{-1}$) ν = 3338, 2917, 1520, 1438, 1336, 1290, 1227, 1169, 951, 813, 730
HRMS (ESI) m/z: $[M-H]^+$ calcd for $C_{13}H_{17}N_2O_2S_2$ 297.0731, found 297.0723

**N-(1-(2,6-dimethylphenoxy)propan-2-yl)thiomorpholine-4-carbothioamide (8c)**

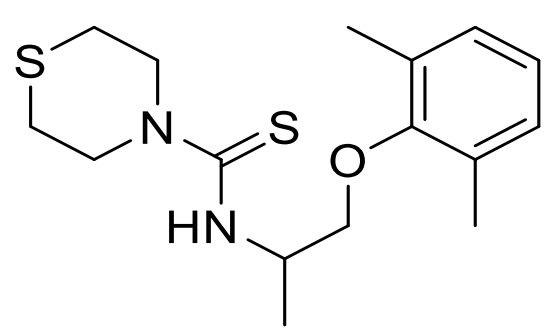

**8c**

N-(1-(2,6-dimethylphenoxy)propan-2-yl)thiomorpholine-4-carbothioamide **8c** was obtained following the above general procedure with 1-(2,6-dimethylphenoxy)-2-propanamine

hydrochloride salt (518.0 mg) and and triethylamine ($Et_3N$, 405 mg, 4.0 mmol, 5.0 equiv.) as a colourless liquid (233.3 mg, 0.720 mmol, 90%).

$^1$H NMR (400 MHz, $CDCl_3$) δ 7.03 – 6.99 (m, 2H), 6.96 – 6.91 (m, 1H), 6.08 (d, *J* = 8.4 Hz, 1H), 5.03 – 4.92 (m, 1H), 4.26 (dt, *J* = 14.4, 5.4 Hz, 2H), 4.13 (dt, *J* = 14.3, 5.4 Hz, 2H), 4.00 (dd, *J* = 9.1, 3.5 Hz, 1H), 3.77 (dd, *J* = 9.1, 2.2 Hz, 1H), 2.71 (t, *J* = 5.2 Hz, 4H), 2.26 (s, 6H), 1.52 (d, *J* = 6.8 Hz, 3H).
$^{13}$C NMR (101 MHz, $CDCl_3$) δ 180.6, 154.7, 130.7, 129.2, 124.4, 73.8, 51.7, 50.7, 26.7, 17.9, 16.3.
IR (cm$^{-1}$) ν = 3424, 3349, 2918, 1520, 1477, 1350, 1294, 1199, 1092, 1026, 949, 842, 769, 680, 639, 480
HRMS (ESI) m/z: $[M+H]^+$ calcd for $C_{16}H_{25}N_2OS_2$ 325.1408, found 325.1418

**N-(3-(10,11-dihydro-5H-dibenzo[a,d][7]annulen-5-ylidene)propyl)-N-methylthiomorpholine-4-carbothioamide (8d)**

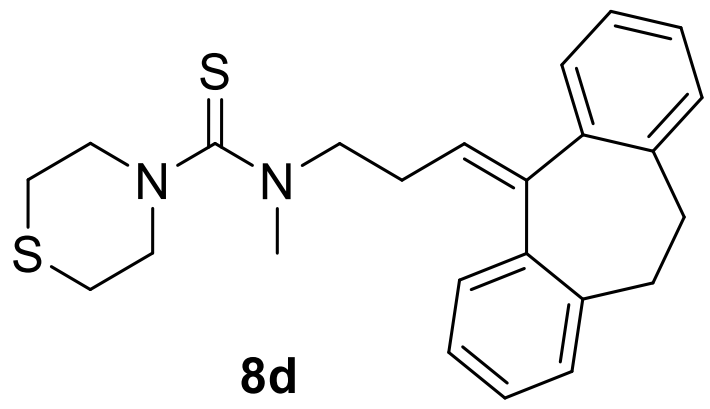


**8d**

N-(3-(10,11-dihydro-5H-dibenzo[a,d][7]annulen-5-ylidene)propyl)-N-methylthiomorpholine-4-carbothioamide **8d** was obtained following the above general procedure with nortriptyline hydrochloridesalt (299.8 mg) and and triethylamine ($Et_3N$, 405 mg, 4.0 mmol, 5.0 equiv.) as a pale yellow liquid (293.9 mg, 0.720 mmol, 90%).

$^1$H NMR (500 MHz, $CDCl_3$) δ 7.31 – 7.09 (m, 7H), 7.07 – 6.99 (m, 1H), 5.81 (t, *J* = 7.4 Hz, 1H), 3.71 – 3.56 (m, 6H), 3.44 – 3.22 (m, 2H), 3.13 – 2.89 (m, 4H), 2.86 – 2.72 (m, 1H), 2.62 (d, *J* = 5.3 Hz, 4H), 2.54 – 2.43 (m, 2H).
$^{13}$C NMR (126 MHz, $CDCl_3$) δ 195.3, 145.1, 140.7, 139.5, 139.2, 136.9, 130.0, 128.5, 128.1, 128.0, 127.7, 127.3, 127.0, 126.1, 125.8, 54.6, 54.0, 40.7, 33.7, 32.0, 27.2, 27.1.
IR (cm$^{-1}$) ν = 2913, 2853, 1486, 1390, 1359, 1295, 1188, 950, 756, 633, 598
HRMS (ESI) m/z: $[M+H]^+$ calcd for $C_{24}H_{29}N_2S_2$ 409.1772, found 409.1765

**N-methyl-N-(3-phenyl-3-(4-(trifluoromethyl)phenoxy)propyl)thiomorpholine-4-carbothioamide (8e)**

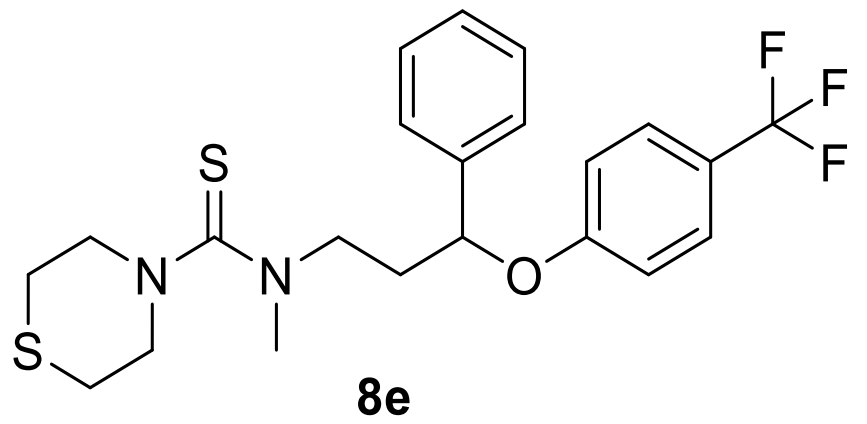


**8e**

N-methyl-N-(3-phenyl-3-(4-(trifluoromethyl)phenoxy)propyl)thiomorpholine-4-carbothioamide **8e** was obtained following the above general procedure with Fluoxetine hydrochloride salt (345.8 mg) and and triethylamine ($Et_3N$, 405 mg, 4.0 mmol, 5.0 equiv.) as a yellow liquid (148.9 mg, 0.328 mmol, 41%).

$^{1}H$ NMR (500 MHz, $CDCl_3$) δ 7.42 (d, *J* = 9.2 Hz, 2H), 7.36 – 7.29 (m, 4H), 7.29 – 7.22 (m, 2H), 6.89 (d, *J* = 8.4 Hz, 2H), 5.19 (dd, *J* = 8.2, 4.5 Hz, 1H), 3.88 – 3.79 (m, 1H), 3.72 – 3.53 (m, 5H), 3.08 (s, 3H), 2.66 – 2.51 (m, 4H), 2.34 – 2.19 (m, 2H).
$^{13}C$ NMR (126 MHz, $CDCl_3$) δ 195.5, 160.3, 140.3, 128.9, 128.1, 126.9 (q, *J* = 3.6 Hz), 125.7, 125.5 (q, *J* = 271.5 Hz), 123.1 (q, *J* = 32.6 Hz), 115.7, 77.8, 54.1, 51.8, 40.9, 36.1, 27.2.
$^{19}F$ NMR (376 MHz, $CDCl_3$) δ -61.57.
IR ($cm^{-1}$) ν = 2917, 1614, 1494, 1393, 1325, 1248, 1160, 1110, 1009, 957, 835, 758, 701, 637, 593, 511
HRMS (ESI) m/z: $[M+H]^+$ calcd for $C_{22}H_{26}F_3N_2OS_2$ 455.1439, found 455.1436

**Supplementary mechanistic studies**

**Synthesis of intermediates**

**Synthesis of 1,4-diazabicyclo[2.2.2]octan-1-ium dibenzylcarbamodithioate (M1)**

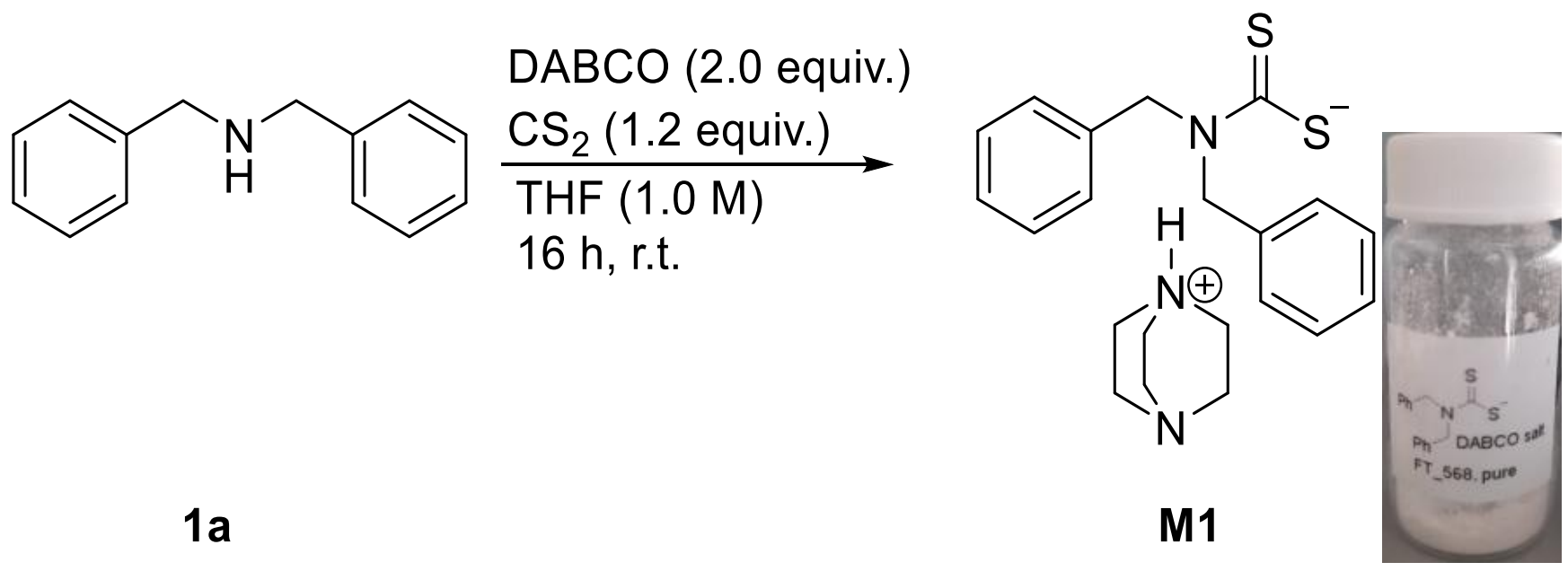


1a M1

According to a modified reported procedure,[36] to an ice cooled solution of DABCO (1.12 g, 10.0 mmol, 2.0 equiv.) and dibenzylamine (986.0 mg, 5.0 mmol, 1.0 equiv.) in THF (5.0 mL, 1 M) was added $CS_2$ (761 mg, 10 mmol, 2.0 equiv.). The reaction mixture was stirred for 1 h at 0 °C and overnight at room temperature. The solid was filtered, washed with THF- petroleum ether (1:1, 2 mL×2) and dried under ambient atmosphere to give the title compound in 84% yield (1.62 g, 4.2 mmol) as colourless solid.

$^{1}H$ NMR (400 MHz, $CDCl_3$) δ 7.34 – 7.15 (m, 10H), 5.36 (s, 2H), 3.19 (d, *J* = 6.1 Hz, 13H).
$^{13}C$ NMR (126 MHz, $CDCl_3$) δ 213.7, 137.5, 128.5, 127.6, 127.0, 55.0, 45.2.
IR ($cm^{-1}$) ν = 3350, 2906, 2770, 2649, 1604, 1494, 1437, 1351, 1186, 1053, 986, 837, 696, 593, 520, 456
HRMS (ESI) m/z: $[M]^+$ calcd for $C_{15}H_{14}NS_2^-$ (dibenzylcarbamodithioate) 272.0568, found 272.0562
HRMS (ESI) m/z: $[M]^+$ calcd for $C_6H_{13}N2^+$ 1,4-diazabicyclo[2.2.2]octan-1-ium) 113.1079, found 113.1087.

**Synthesis of 1,4-diazabicyclo[2.2.2]octan-1-ium 4-(trifluoromethyl)piperidine-1-carbodithioate (1m')**

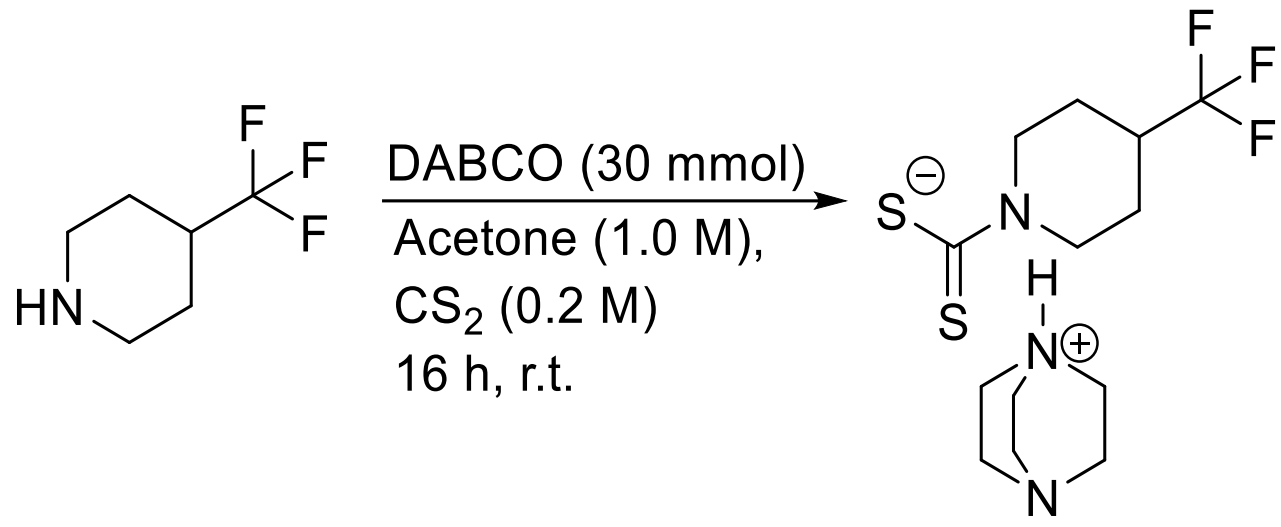


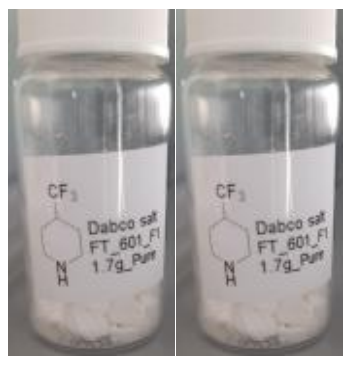

1m 1m'

According to a modified procedure,[37] $CS_2$ (32.5 mL, 0.167 M) was added to a stirring mixture of DABCO (2.19 g, 19.5 mmol, 3.0 equiv.) and 4-Trifluoromethylpiperidine (1.01 g, 6.50 mmol, 1.0 equiv.) in acetone (6.50 mL, 0.167 M). The resulting mixture was further stirred at room temperature. After completion of the reaction, the solution was filtered and the solid was washed with acetone to obtain 1,4-diazabicyclo[2.2.2]octan-1-ium 4-(trifluoromethyl)piperidine-1-carbodithioate in 77% yield (1.71 g, 5.0 mmol).

$^1$H NMR (500 MHz, $CDCl_3$) δ 11.03 (s, 1H), 5.86 (d, *J* = 12.0 Hz, 2H), 3.16 (s, 12H), 2.95 (t, *J* = 12.7 Hz, 2H), 2.34 – 2.18 (m, 1H), 1.86 (d, *J* = 11.7 Hz, 2H), 1.67 – 1.54 (m, 2H).
$^{13}$C NMR (126 MHz, $CDCl_3$) δ 210.9, 127.3 (q, *J* = 278.3 Hz), 49.2, 45.6 (d, *J* = 4.8 Hz), 40.4 (q, *J* = 27.6 Hz), 24.5.
$^{19}$F NMR (376 MHz, $CDCl_3$) δ -73.46.
IR (cm$^{-1}$) ν = 2965, 2888, 2770, 2657, 1461, 1392, 1329, 1256, 1209, 1130, 1079, 995, 914, 844, 783, 698, 593, 454
HRMS (ESI) m/z: $[M]^+$ calcd for $C_7H_9N_3$ $F_3S_2^-$ (4-(trifluoromethyl)piperidine-1-carbodithioate) 228.0129, found 228.0127
HRMS (ESI) m/z: $[M]^+$ calcd for $C_6H_{13}N_2^+$ (1,4-diazabicyclo[2.2.2]octan-1-ium) 113.1079, found 113.1086.

**Synthesis of tetrabenzylthiuram disulfide (M2)**

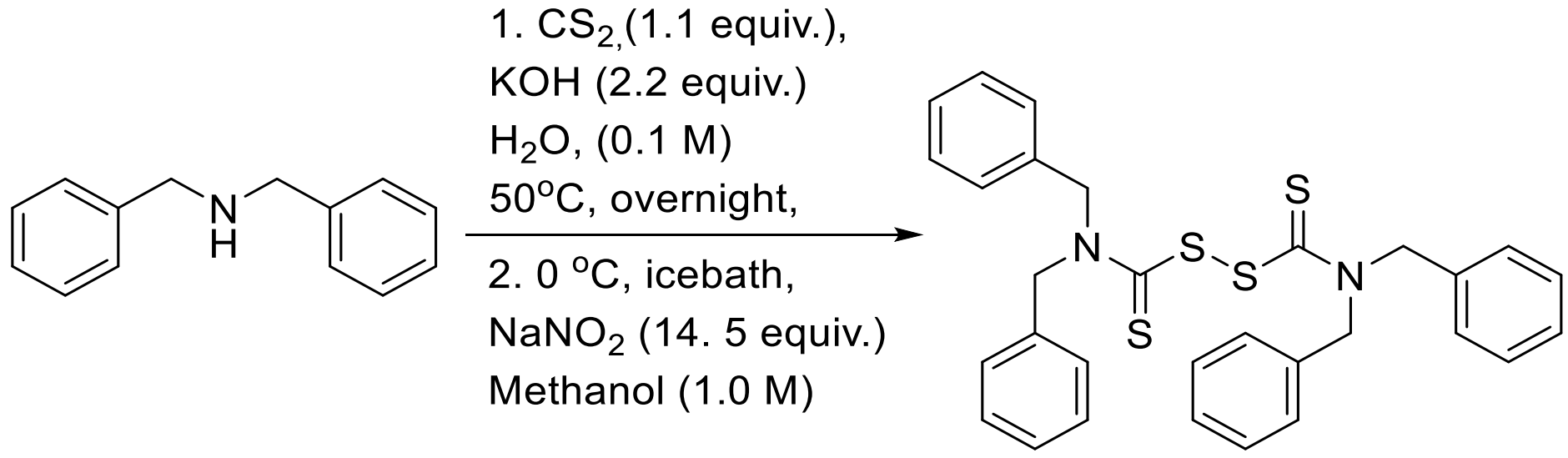


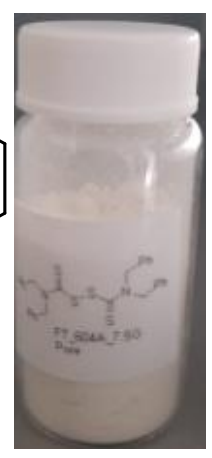

1a M2

According to a modified reported procedure[38] tetrabenzylthiuram disulfide was as obtained and crystallized from the ethanol to produce the desired product in 88% yield (13.2 g , 27.5 mmol).

$^1$H NMR (500 MHz, $CDCl_3$) δ 7.47 – 7.32 (m, 20H), 5.38 (s, 4H), 5.20 (s, 4H).
$^{13}$C NMR (126 MHz, $CDCl_3$) δ 196.4, 134.9, 134.3, 129.2, 129.0, 128.4, 128.0, 127.6, 59.1, 55.0.
IR (cm$^{-1}$) ν = 3019, 2906, 1471, 1401, 1348, 1232, 1120, 1076, 1025, 942, 901, 694, 635, 547, 502, 454
HRMS (EI) m/z: $[M+H]^+$ calcd for $C_{30}H_{29}N_2S_4$ 545.1214, found 545.1204
In accordance with previous reports.[39-42]

**Synthesis of 4-(trifluoromethyl)piperidine-1-carbothioic dithioperoxyanhydride (1m'')**

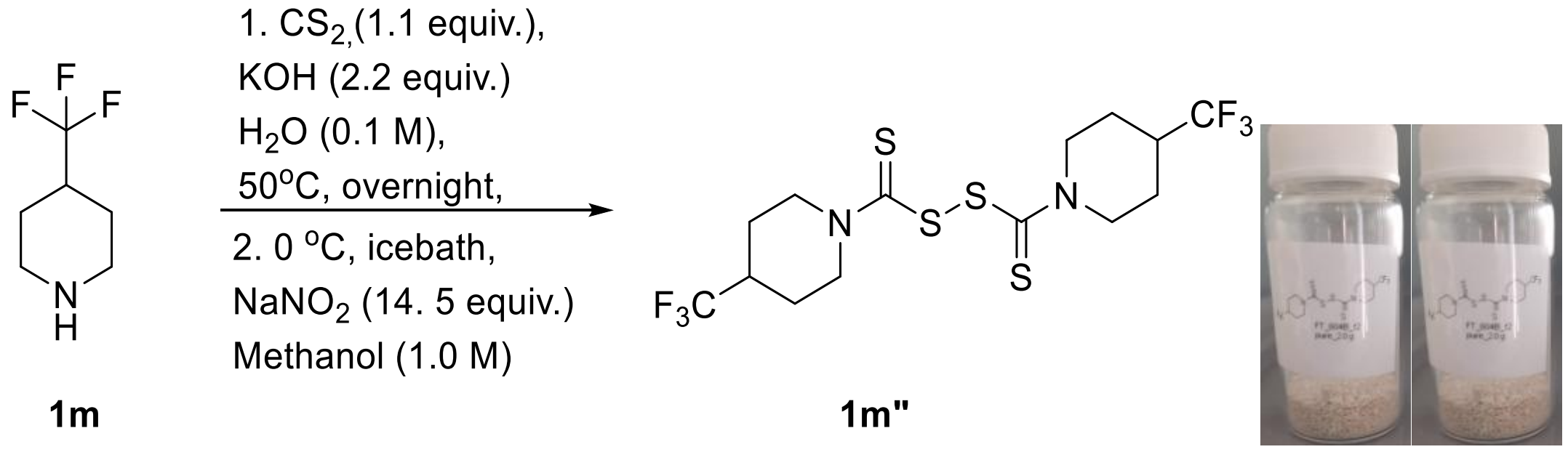


According to a modified reported procedure,[38] 4-(trifluoromethyl)piperidine-1-carbothioic dithioperoxyanhydride was obtained in 50% yield (7.1 g, 15.6 mmol) as a colourless solid.

$^1$H NMR (400 MHz, $CDCl_3$) δ 5.26 (s, 4H), 3.36 (s, 4H), 2.55 – 2.36 (m, 2H), 2.06 (d, *J* = 11.7 Hz, 4H), 1.90 – 1.75 (m, 4H).
$^{13}$C NMR (101 MHz, $CDCl_3$) δ 193.82, 126.74 (q, *J* = 278.5 Hz), 51.01, 40.20 (q, *J* = 28.1 Hz), 24.69.
$^{19}$F NMR (376 MHz, $CDCl_3$) δ -73.44.
IR ($cm^{-1}$) ν = 2981, 1486, 1433, 1337, 1254, 1133, 1079, 966, 921, 823, 699, 604, 560, 452
HRMS (EI) m/z: $[M+H]^+$ calcd for $C_{14}H_{19}N_2S_4F_6$ 457.0335, found 457.0332

**Standard reaction with 1,4-diazabicyclo[2.2.2]octan-1-ium dibenzylcarbamodithioate intermediate (M1)**

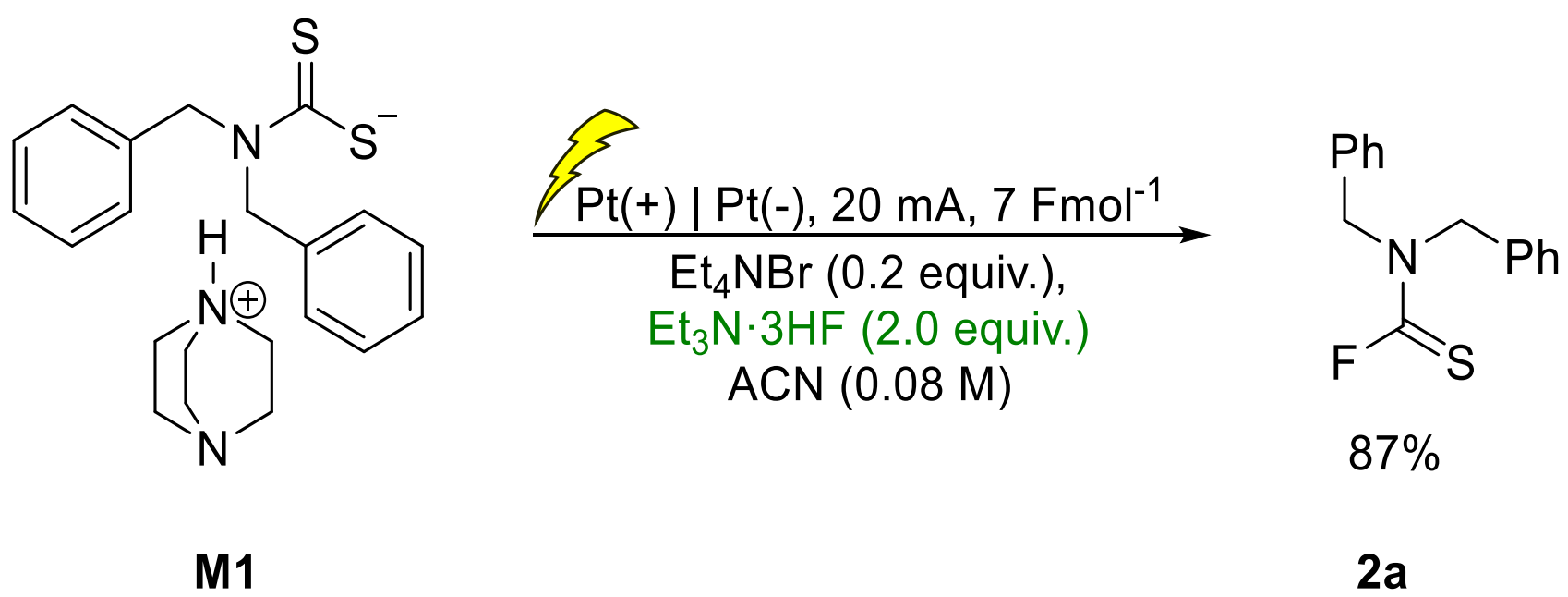


The pre-made 1,4-diazabicyclo[2.2.2]octan-1-ium dibenzylcarbamodithioate (154.0 mg, 0.4 mmol, 1.0 equiv.) intermediate salt was subjected to electrolysis by addition of acetonitrile (ACN) (5 mL, 0.08 M), $Et_4NBr$ (16.8 mg, 0.08 mmol, 0.2 equiv.) and $Et_3N·3HF$ (120 µL, 0.8 mmol, 2.0 equiv.) in an electrasyn vial at 11.11 mA $cm^{-2}$ current density for 7 F $mol^{-1}$ (i.e. 3.75 h) using a platinum anode (WE) and platinum cathode (CE). The reaction was monitored by HPLC. After full consumption of the intermediate thiuram, the reaction mixture was quenched with a saturated aqueous solution of $NaHCO_3$, extracted with DCM. The organic layers were then combined and dried with $Na_2SO_4$. The solvent was removed under reduced pressure. The crude mixture was filtered through silica to obtain the final product dibenzylcarbamothioic fluoride in 87% yield (90.0 mg, 0.348 mmol).

**Standard reaction with tetrabenzylthiuram disulfide intermediate (M2)**

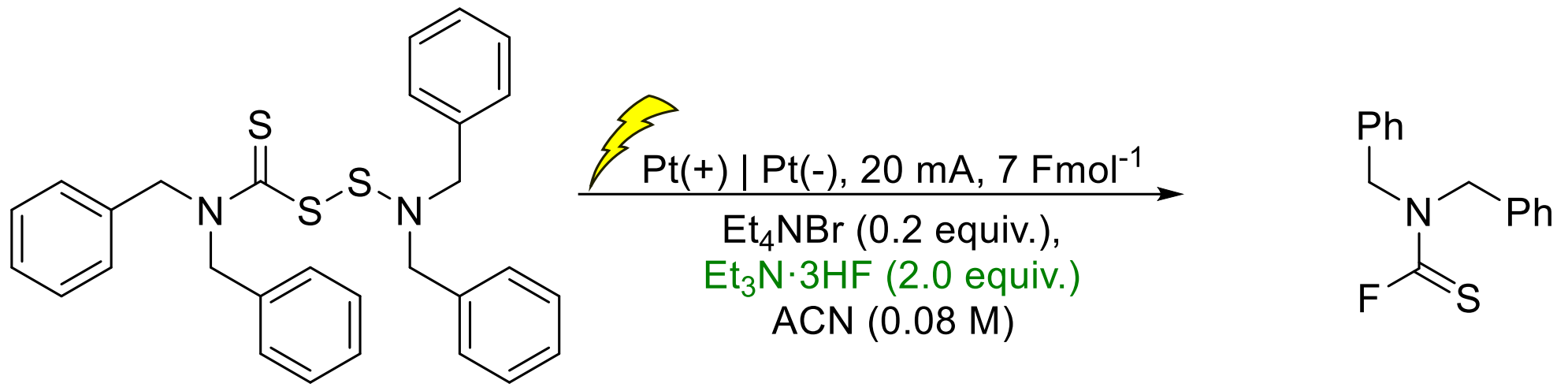


**M2** **2a**

The pre-made tetrabenzylthiuram disulfide (218.0 mg, 0.4 mmol, 1.0 equiv.) intermediate was subjected to electrolysis by addition of acetonitrile (ACN) (5 mL, 0.08 M), $Et_4NBr$ (16.8 mg, 0.08 mmol, 0.2 equiv.) and $Et_3N{\cdot}3HF$ (130 µL, 0.8 mmol, 2.0 equiv.) in an electrasyn vial at 11.11 mA $cm^{-2}$ current density for 7 F $mol^{-1}$ (i.e., 3.75 h) using a platinum anode (WE) and platinum cathode (CE). The reaction was monitored by HPLC. After full consumption of the starting material thiuram, the reaction mixture was quenched with a saturated aqueous solution of $NaHCO_3$ and extracted with DCM. The organic layers were then combined and dried with $Na_2SO_4$. The solvent was removed under reduced pressure. The crude mixture was filtered through silica to obtain the final product dibenzylcarbamothioic fluoride in 78% yield (80.5 mg, 0.312 mmol).

**1,4-diazabicyclo[2.2.2]octan-1-ium dibenzylcarbamodithioate Intermediate isolation (M1)**

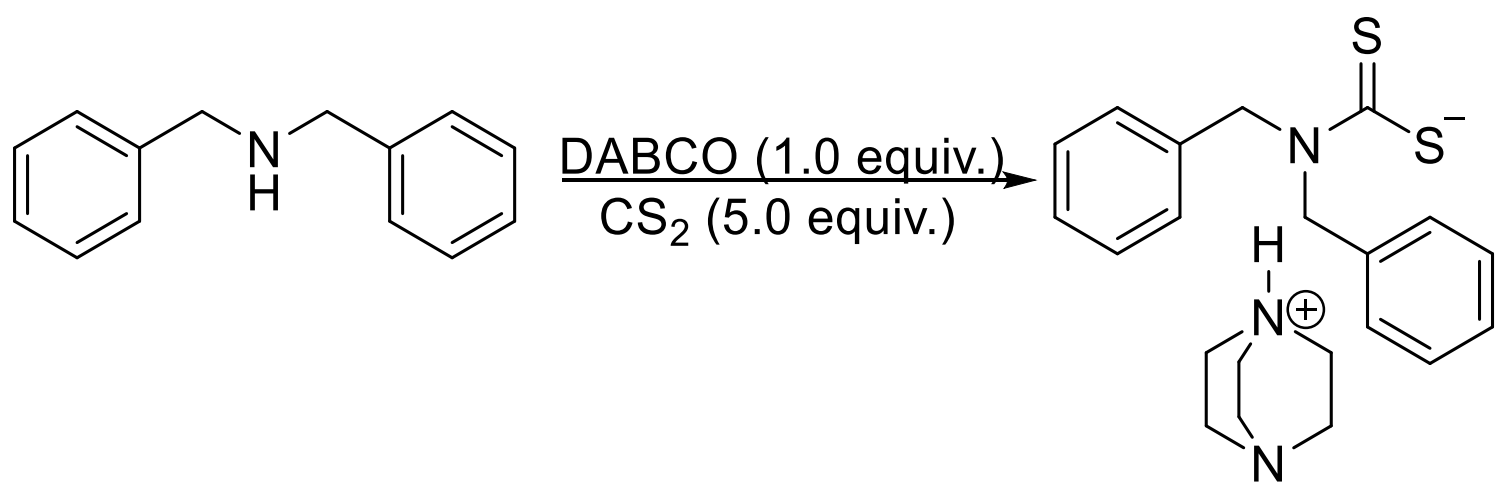


**1a** **M1**

Dibenzylamine (78.9 mg, 0.4 mmol, 1.0 equiv.), DABCO (45.0 mg, 0.4 mmol, 1.0 equiv.), and $CS_2$ (152 mg, 2.0 mmol, 5.0 equiv.) were added into a 5 mL one compartment Electrasyn vial to generate in situ salt 1,4-diazabicyclo[2.2.2]octan-1-ium dibenzylcarbamodithioate. Excess $CS_2$ was removed by rotary evaporation to obtain the crude salt, which was recrystallised from a THF/hexane solvent mixture to give crystals of the desired product M1 in 90% yield (138 mg, 0.36 mmol).

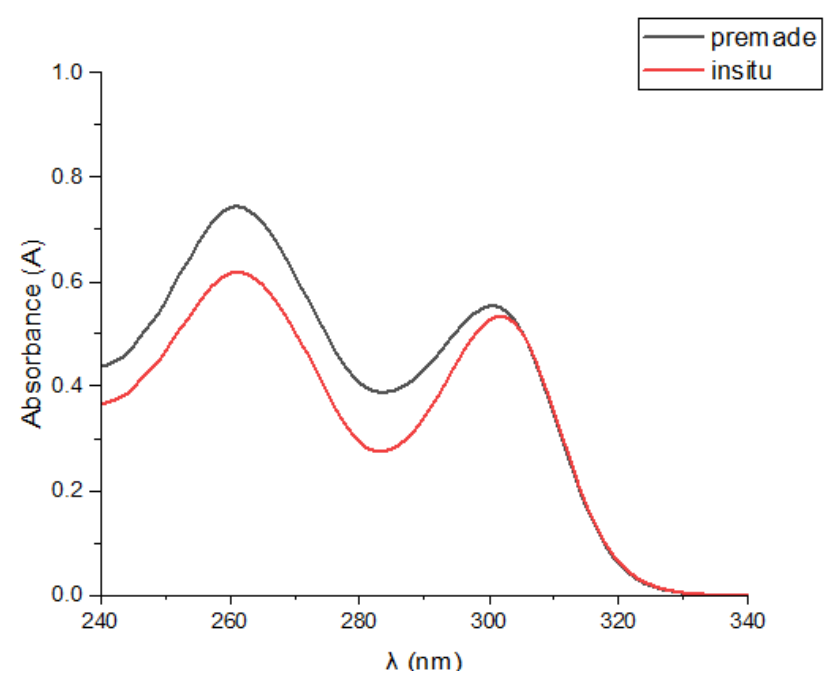


**Supplementary Fig. 5**: UV absorption spectra: Comparison of premade (black) and in situ (red) formed **M1** salt.

**Tetrabenzylthiuram disulfide Intermediate isolation (M2)**

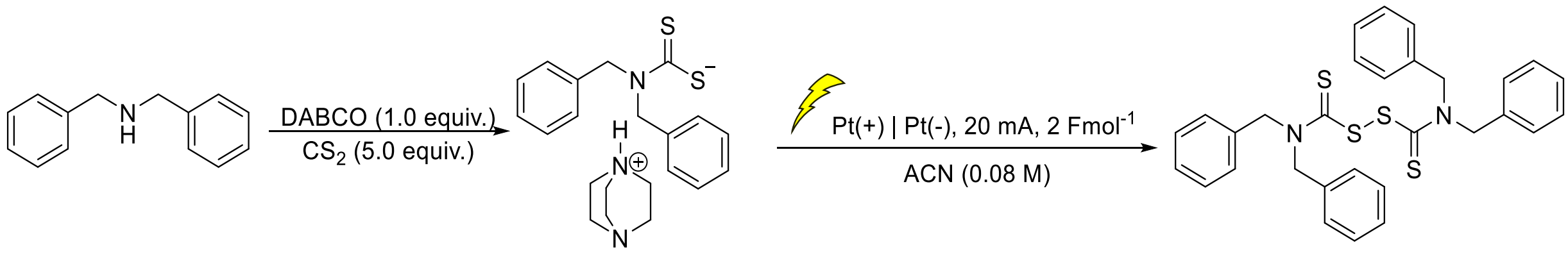


**1a** **M1** **M2** 48 %

Dibenzylamine (78.9 mg, 0.4 mmol, 1.0 equiv.), DABCO (45.0 mg, 0.4 mmol, 1.0 equiv.), and $CS_2$ (152 mg, 2.0 mmol, 5.0 equiv.) were added into a 5 mL one compartment Electrasyn vial to generate in situ salt. Then acetonitrile (ACN) (5 mL, 0.08 M) was added, and the solution was electrolyzed at 11.11 mA $cm^{-2}$ for 2 F $mol^{-1}$ (i.e. 1.1 h) using a platinum anode (WE) and platinum cathode (CE). The crude reaction mixture was extracted with DCM and aqueous $NaHCO_3$ solution. The organic layers were then combined and dried with $Na_2SO_4$. The solvent was removed under reduced pressure. The crude mixture was filtered through silica to obtain the **tetrabenzylthiuram disulfide (M2)** in 48% yield (104.4 mg, 0.192 mmol).

**Supplementary crystallography data**

**1,4-diazabicyclo[2.2.2]octan-1-ium dibenzylcarbamodithioate (M1)**

Crystals of M1 suitable for single-crystal XRD were obtained from the THF/hexane (1:1) filtrate generated during washing of the crude solid. The filtrate was left to stand overnight at room temperature.

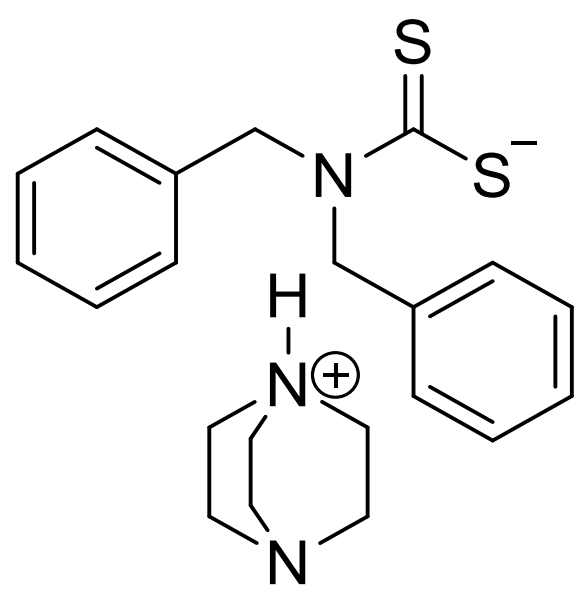

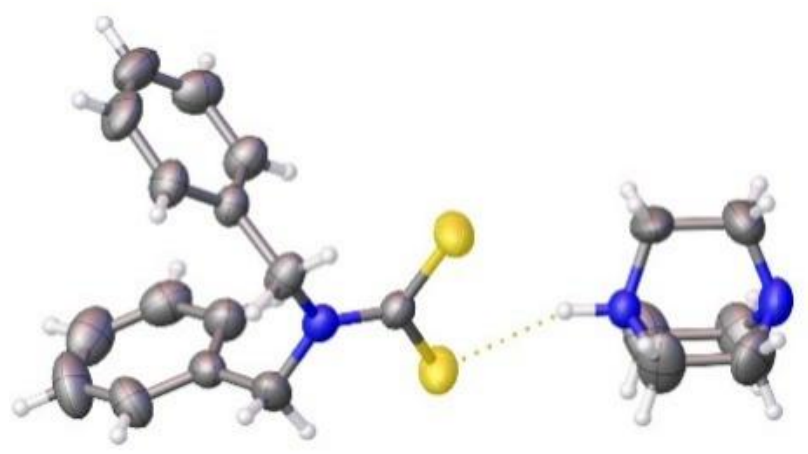

**M1**

**Tetrabenzylthiuram disulfide (M2)**

Crystals of M2 suitable for single-crystal XRD were grown from a saturated ethanol solution left to stand overnight at room temperature.

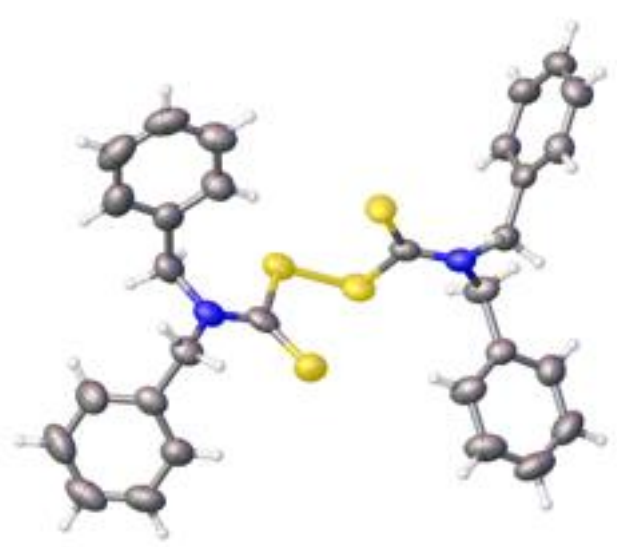

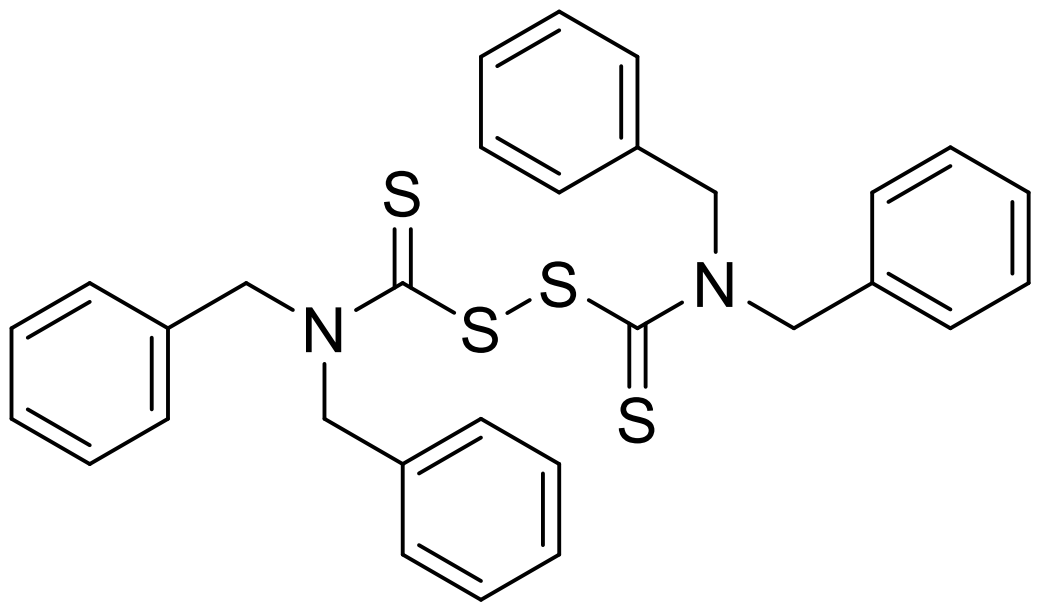

**M2**

**methylene bis(dibenzylcarbamodithioate) (M10)**

Crystals of M10 suitable for single-crystal XRD were obtained when a saturated dichloromethane solution of the dibenzylamine-derived dithiocarbamate salt M1 was left to stand overnight at room temperature, unexpectedly affording methylene bis(dibenzylcarbamodithioate) M10.

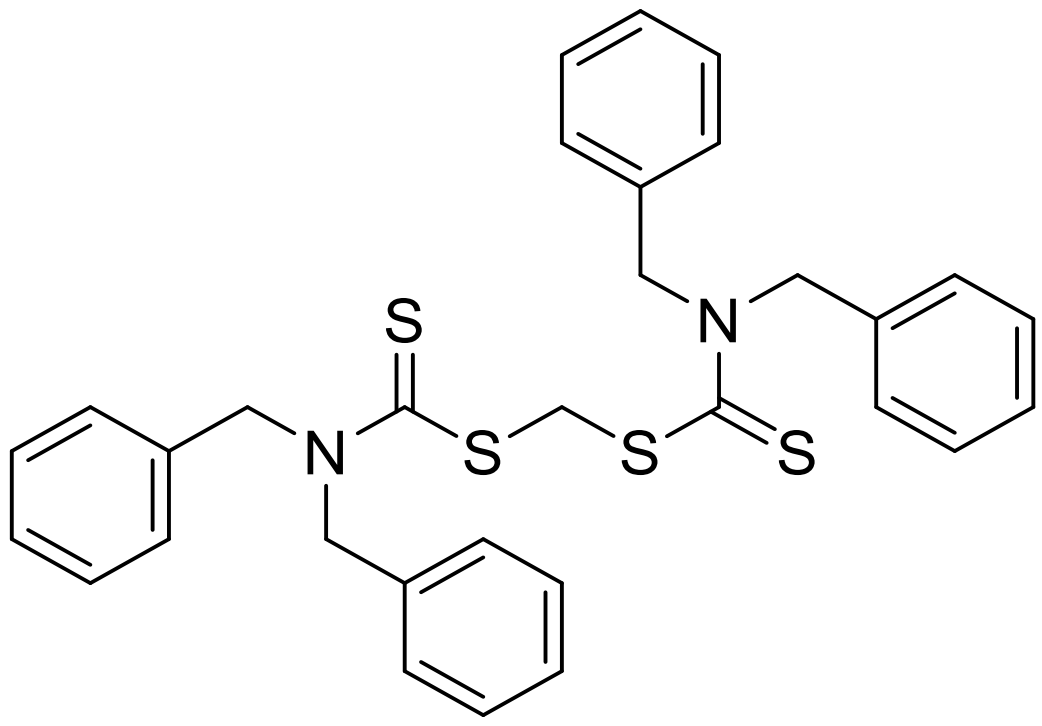

**M10**

**Supplementary Table 4: Crystallographic data for M1, M2 and M10.**

| Identification code | **M1** | **M2** | **M10** |
|---|---|---|---|
| Empirical formula | $C_{21}H_{27}N_3S_2$ | $C_{30}H_{28}N_2S_4$ | $C_{31}H_{30}N_2S_4$ |
| Formula weight | 385.57 | 544.78 | 558.861 |
| Temperature/K | 293 | 293 | 293 |
| Crystal system | monoclinic | monoclinic | orthorhombic |
| Space group | $P2_1/c$ | C2/c | $P2_12_12_1$ |
| a/Å | 12.3592(6) | 19.5471(6) | 10.5971(10) |
| b/Å | 6.3905(3) | 14.3436(4) | 10.9112(8) |
| c/Å | 26.4370(12) | 22.0182(7) | 25.280(2) |
| α/° | 90 | 90 | 90 |
| β/° | 100.881(4) | 115.819(4) | 90 |
| γ/° | 90 | 90 | 90 |
| Volume/$Å^3$ | 2050.49(17) | 5557.1(3) | 2923.1(4) |
| Z | 4 | 8 | 4 |
| $\rho_{calc}g/cm^3$ | 1.249 | 1.302 | 1.270 |
| $\mu/mm^{-1}$ | 0.269 | 0.364 | 0.348 |
| F(000) | 824.0 | 2288.0 | 1178.5 |
| Crystal size/$mm^3$ | 0.94 × 0.25 × 0.11 | 0.5 × 0.3 × 0.1 | 1.07 × 0.67 × 0.4 |

| | | | |
|---|---|---|---|
| Radiation | Mo Kα (λ = 0.71073) | Mo Kα (λ = 0.71073) | Mo Kα (λ = 0.71073) |
| 2Θ range for data collection/° | 6.278 to 52.736 | 4.664 to 52.744 | 5.6 to 55.76 |
| Index ranges | -15 ≤ h ≤ 15, -7 ≤ k ≤ 7, -33 ≤ l ≤ 33 | -24 ≤ h ≤ 24, -17 ≤ k ≤ 17, -27 ≤ l ≤ 27 | -14 ≤ h ≤ 15, -15 ≤ k ≤ 15, -35 ≤ l ≤ 35 |
| Reflections collected | 35372 | 47294 | 23526 |
| Independent reflections | 4173 [$R_{int}$ = 0.0398, $R_{sigma}$ = 0.0217] | 5653 [$R_{int}$ = 0.0217, $R_{sigma}$ = 0.0145] | 6820 [$R_{int}$ = 0.0583, $R_{sigma}$ = 0.0929] |
| Data/restraints/parameters | 4173/0/235 | 5653/0/325 | 6820/0/334 |
| Goodness-of-fit on $F^2$ | 1.096 | 1.098 | 1.044 |
| Final R indexes [I>=2σ (I)] | $R_1$ = 0.0778, $wR_2$ = 0.1968 | $R_1$ = 0.0504, $wR_2$ = 0.1261 | $R_1$ = 0.0725, $wR_2$ = 0.1561 |
| Final R indexes [all data] | $R_1$ = 0.0895, $wR_2$ = 0.2026 | $R_1$ = 0.0613, $wR_2$ = 0.1322 | $R_1$ = 0.1256, $wR_2$ = 0.1756 |
| Largest diff. peak/hole / e $Å^{-3}$ | 0.44/-0.26 | 0.49/-0.28 | 0.38/-0.42 |
| Flack parameter 0.16(5) | | | 0.16(5) |

## UV-Visible spectroscopy

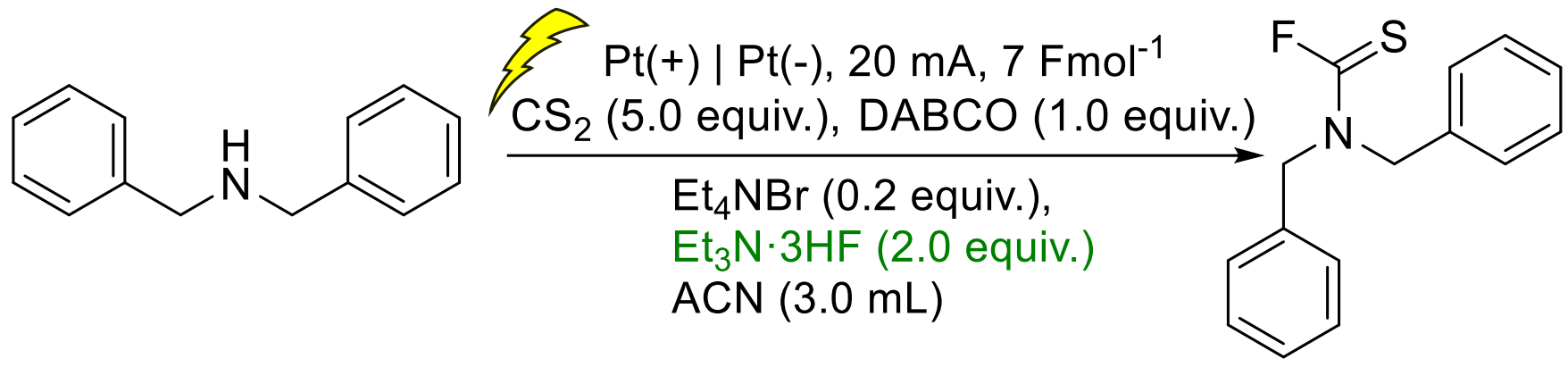


**1a** **2a**

Dibenzylamine (38.4 µL, 0.2 mmol, 1.0 equiv.) was dissolved in 3 mL of ACN, and 10 µL of the solution were removed, and diluted to 0.05 mM concentration. Then UV-Vis spectrum were acquired. Then, DABCO (22.5 mg, 0.2 mmol, 1.0 equiv.), $CS_2$ (76.1 mg, 1 mmol, 5.0 equiv.), $Et_4NBr$ (8.4 mg, 0.04 mmol, 0.2 equiv.) and $Et_3N{\cdot}3HF$ (60 µL, 0.4 mmol, 2.0 equiv.) were individually added to the reaction mixture and 10 µL were removed from the mixture, and diluted to 0.05 mM concentration and the UV-Vis spectra were acquired after the addition of every reagent. The reaction was then electrolysed at 11.11 mA $cm^{-2}$ current density for 7 F $mol^{-1}$ (i.e., 1 h 52 min) using a platinum anode (WE) and platinum cathode (CE). During the reaction, after every 1 F $mol^{-1}$, 10 µL were removed and the UV-Vis spectra were acquired. The pre-made 1,4-diazabicyclo[2.2.2]octan-1-ium dibenzylcarbamodithioate (**M1**), the tetrabenzylthiuram disulfide (**M2**) and the dibenzylcarbamothioic fluoride (**2a)** were also measured in the same way for reference.

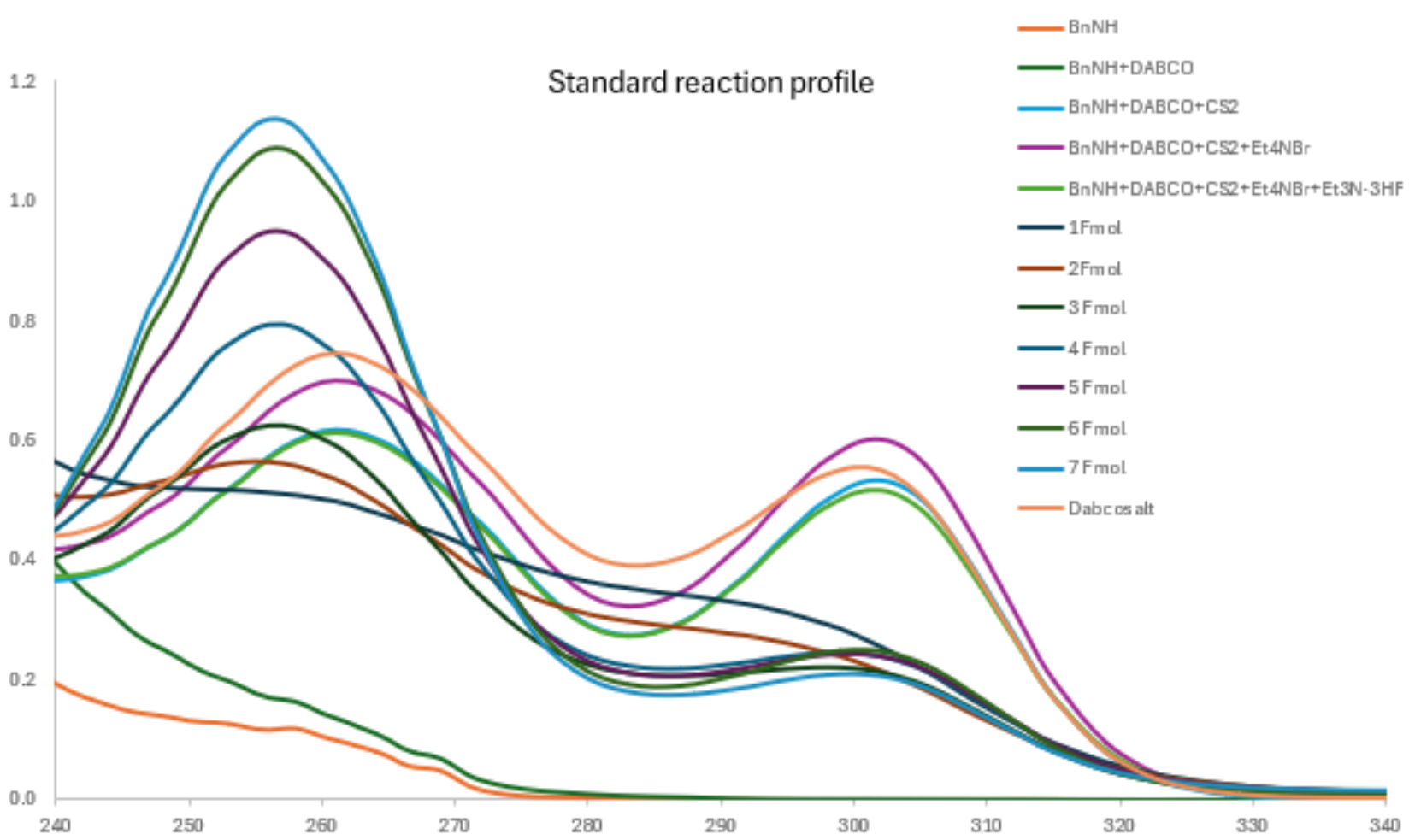


**Supplementary Fig. 6**: UV reaction profile: Electrochemical thiocarbamoyl fluoride synthesis reaction course from 1 F $mol^{-1}$ to 7 F $mol^{-1}$

## Reaction monitoring through HRMS

Dibenzylamine (38.4 µL, 0.2 mmol, 1.0 equiv.) was dissolved in 3 mL of ACN, and 10 µL of the solution was removed, diluted to 0.001 mM concentration, and subjected to HRMS analysis through direct injection technique. Then, DABCO (22.5 mg, 0.2 mmol, 1.0 equiv.), $CS_2$ (76.1 mg, 1 mmol, 5.0 equiv.), $Et_4NBr$ (8.4 mg, 0.04 mmol, 0.2 equiv.) and Et3N·3HF (60 µL, 0.4 mmol, 2.0 equiv.) were individually added to the same reaction vial and 10 µL was removed each time from the mixture, diluted to 0.001 mM concentration and subjected to HRMS analysis after the addition of every reagent. Then, the same reaction mixture was electrolysed at 11.11 mA $cm^{-2}$ current density for 7 F $mol^{-1}$ (i.e. 1 h 52 min) using a platinum anode (WE) and platinum cathode (CE). During the reaction, after every 1 F $mol^{-1}$, 10 µL was removed and

the HRMS spectra were acquired. The pre-made 1,4-diazabicyclo[2.2.2]octan-1-ium dibenzylcarbamodithioate (**M1**), the tetrabenzylthiuram disulfide (**M2**) and the dibenzylcarbamothioic fluoride (**2a**) were also measured in the same way for reference.

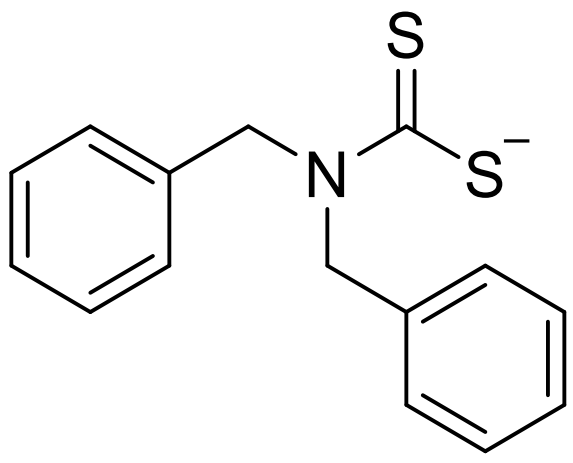

HRMS (ESI) m/z: $[M]^-$ calcd for $C_{15}H_{14}NS_2$ 272.0568, found 272.0562

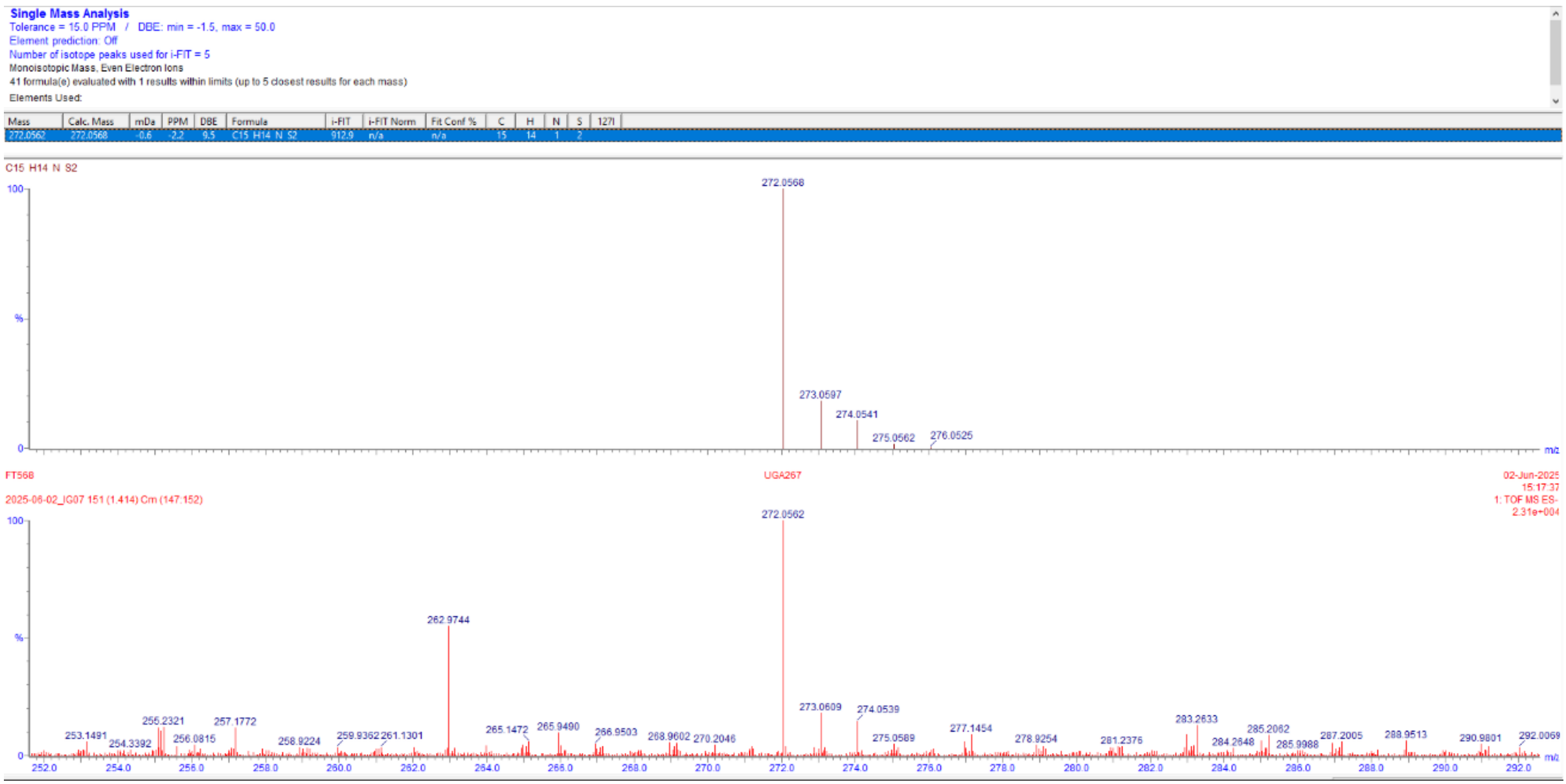


**Supplementary Fig. 7:** Reaction course: Detection of M1 anion

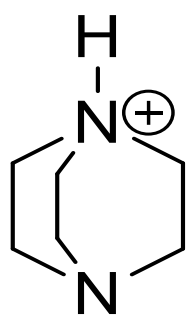

HRMS (ESI) m/z: $[M]^+$ calcd for $C_6H_{13}N_2$ 113.1079, found 113.1087

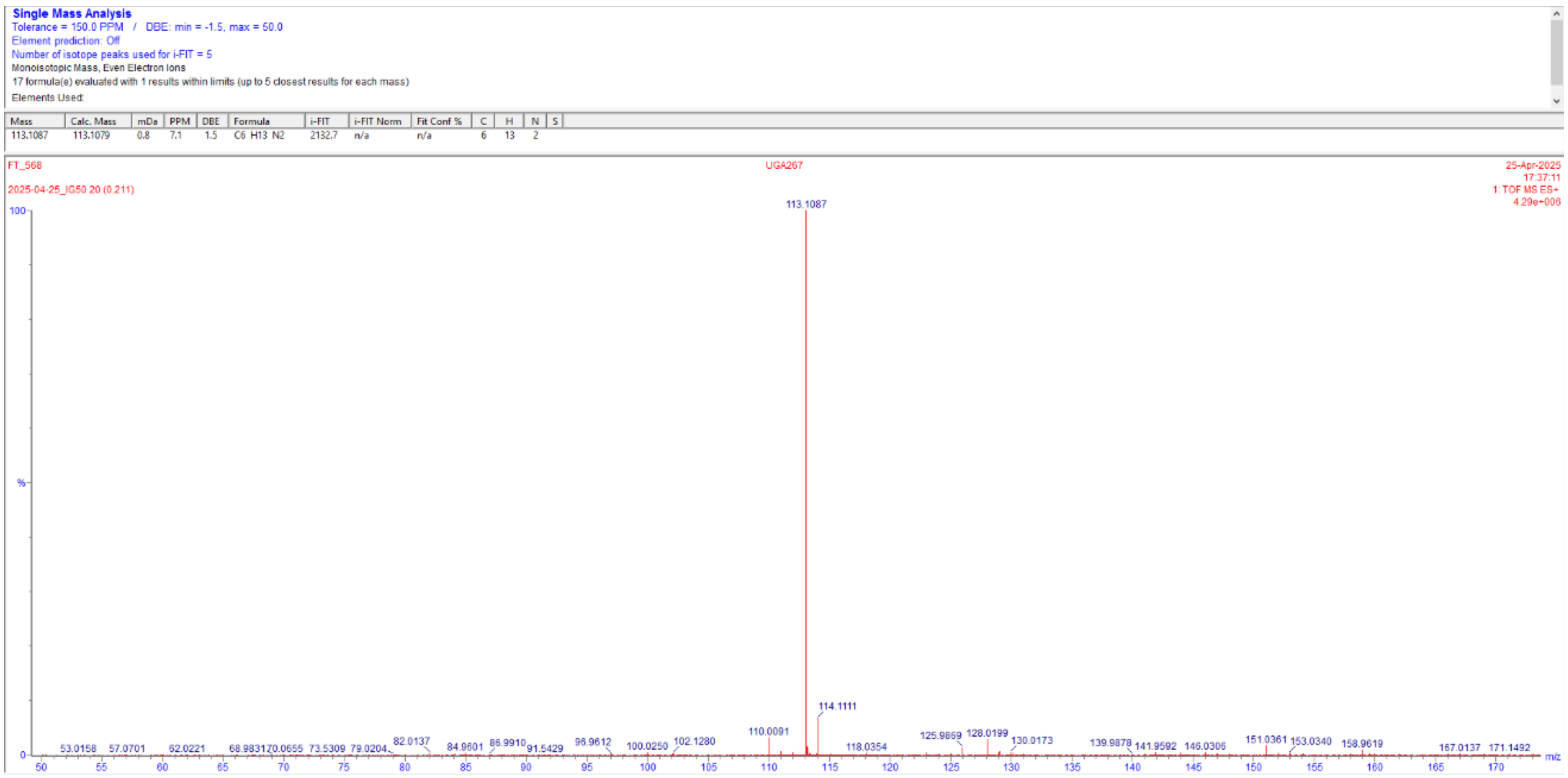


**Supplementary Fig. 8:** Reaction course: Detection of M1 cation

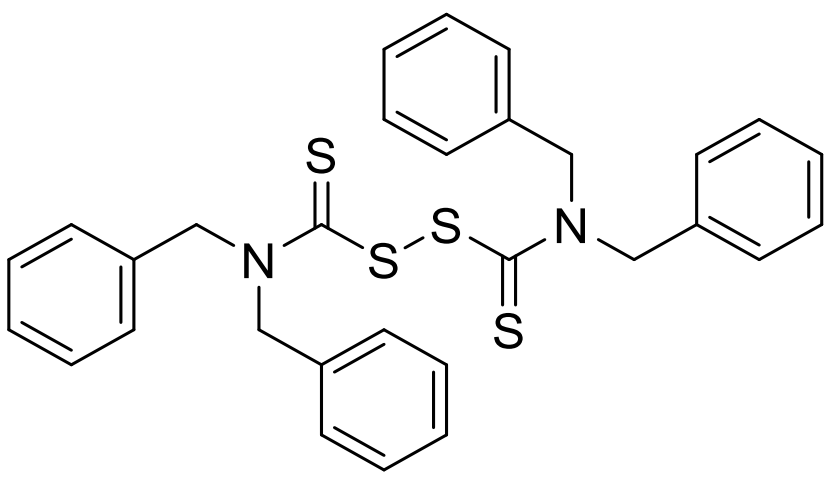


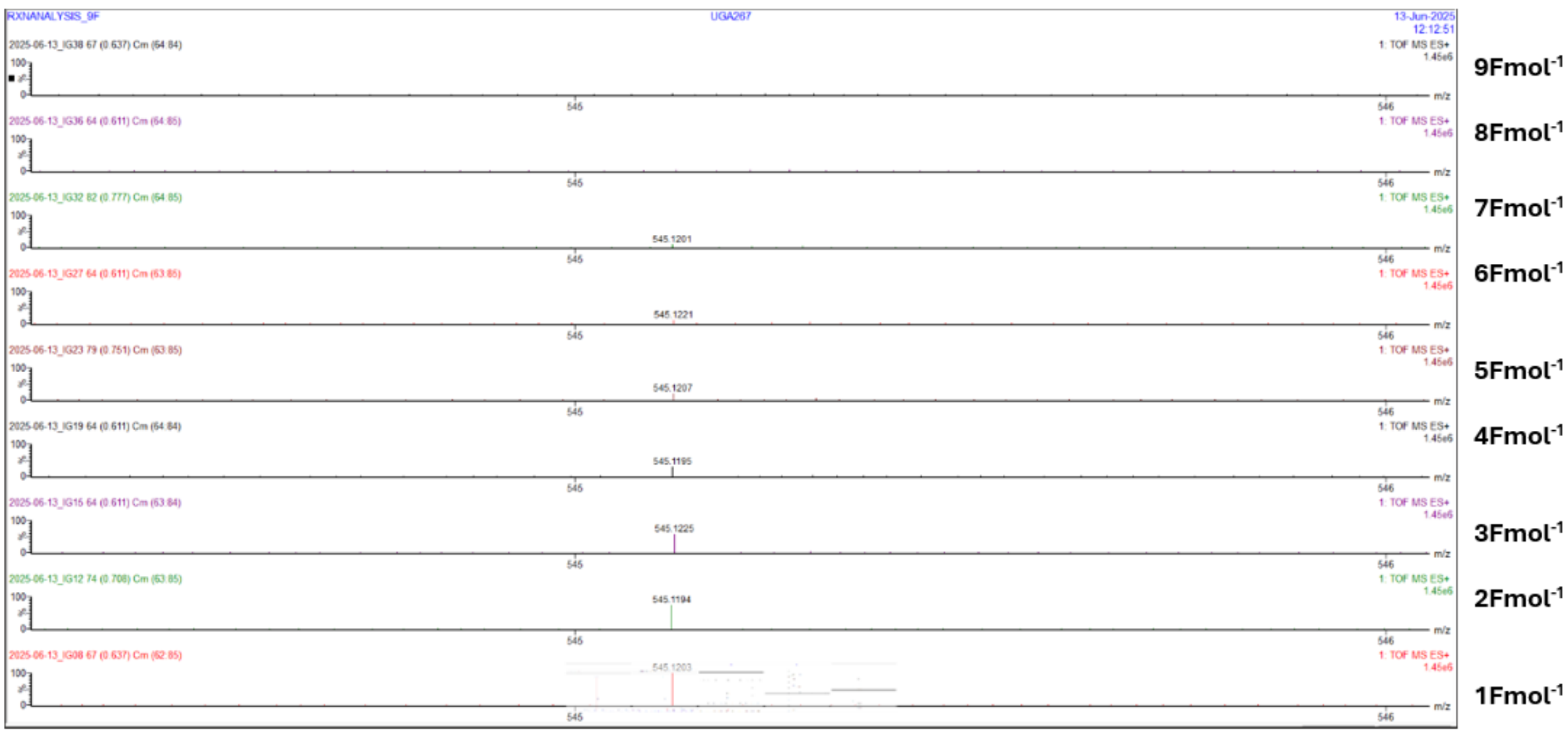


**Supplementary Fig. 9:** HRMS reaction profile**:** Electrochemical reaction course from 1 F mol$^{-1}$ to 9 F mol$^{-1}$ and sample measured at every 1 F mol$^{-1}$.

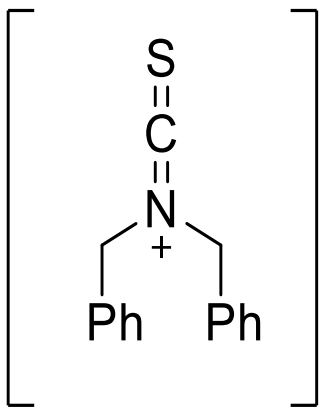


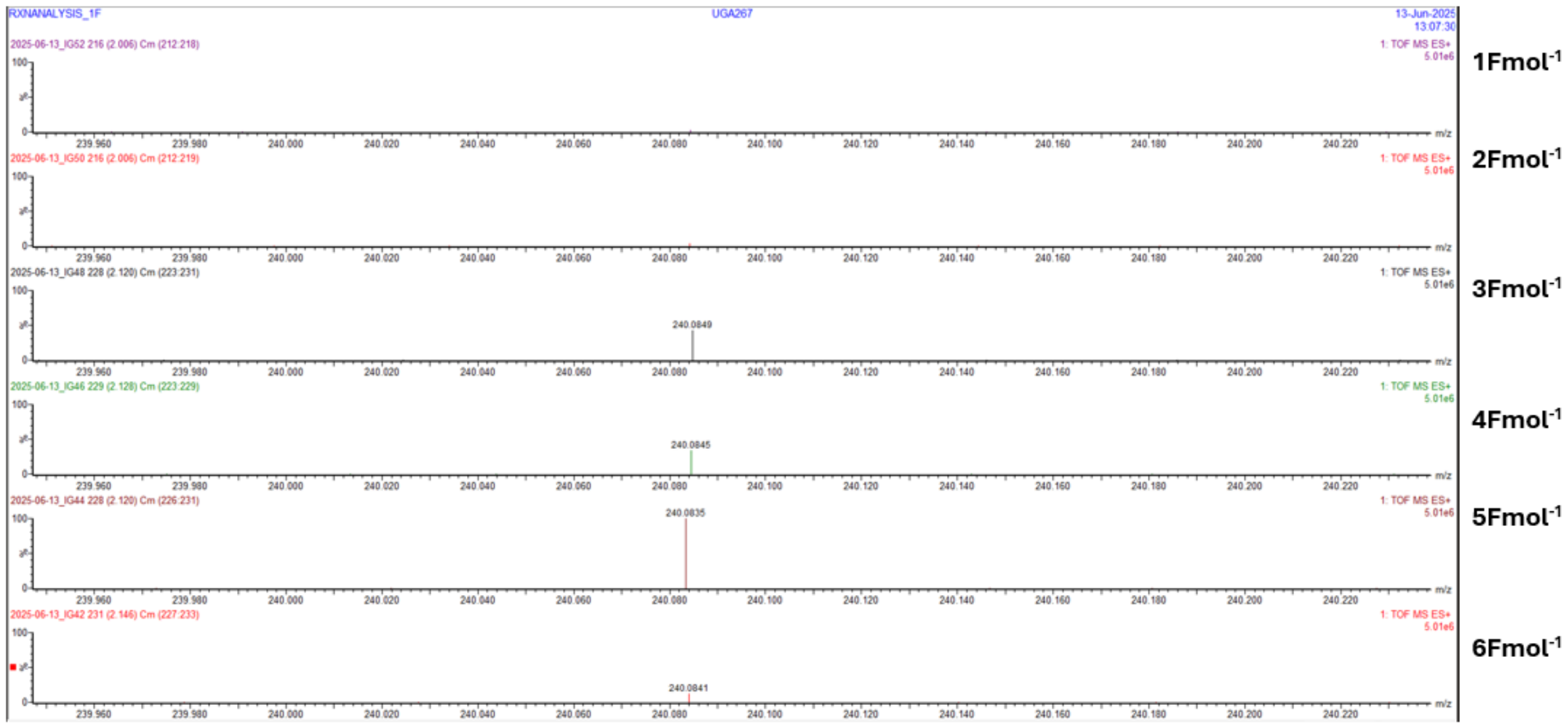


**Supplementary Fig. 10:** HRMS reaction profile: Electrochemical reaction course from 1 F mol$^{-1}$ to 6 F mol$^{-1}$ and sample measured at every 1 F mol$^{-1}$.

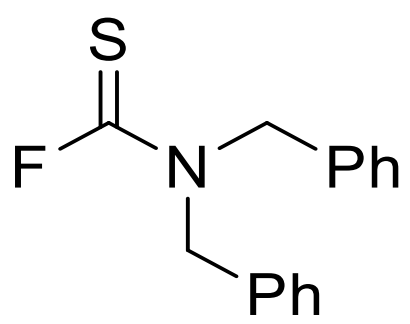


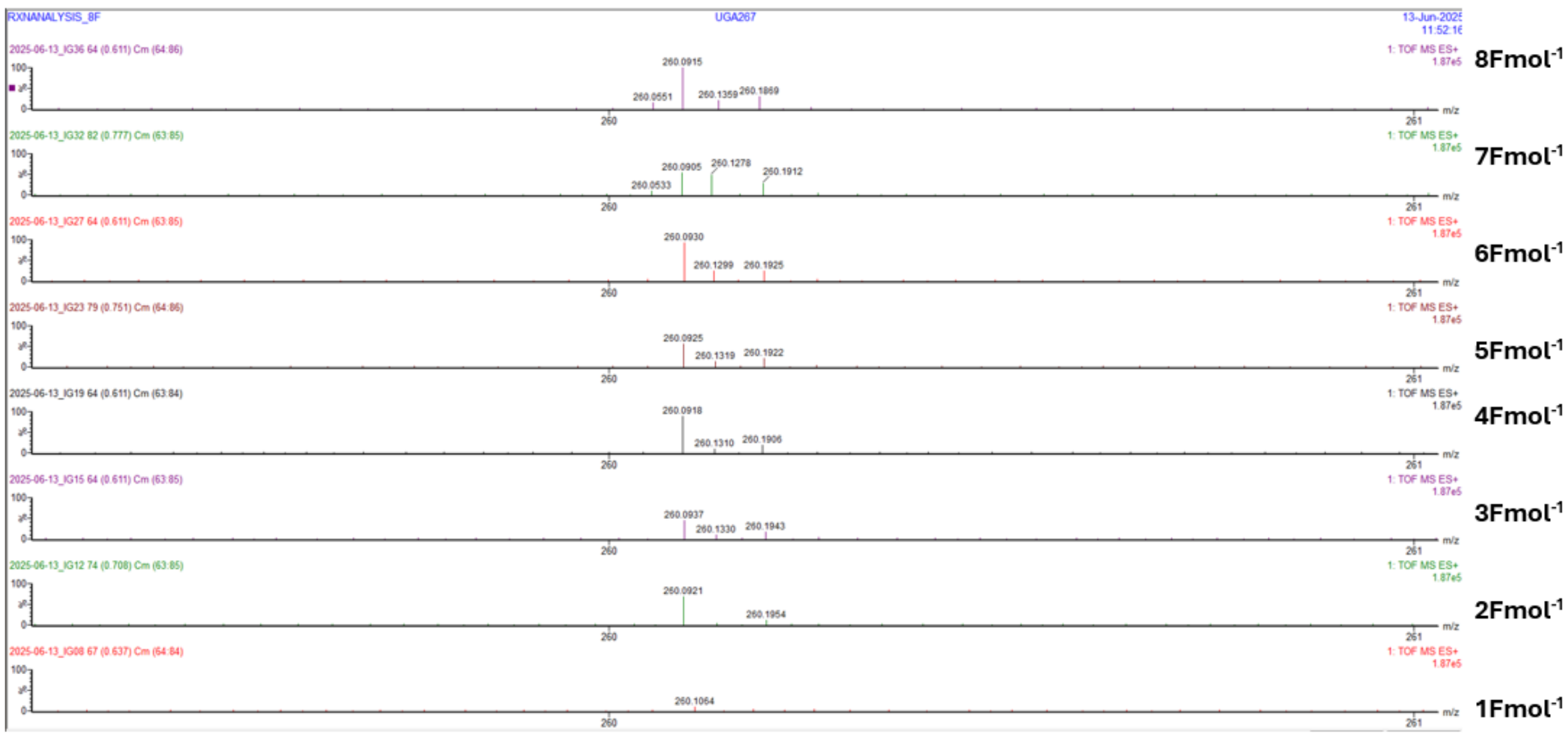


**Supplementary Fig. 11:** HRMS reaction profile: Electrochemical reaction from course 1 F mol$^{-1}$ to 8 F mol$^{-1}$ and sample measured at every 1 F mol$^{-1}$.

### $^{19}$F NMR studies

4-Trifluoromethylpiperidine (31.1 mg, 0.2 mmol, 1.0 equiv.) and (trifluoromethyl)benzene (9.73 mg, 0.067 M, 0.33 equiv. as internal standard) was dissolved in 3.0 mL of ACN, then 50 µL of the solution was removed and diluted with $CDCl_3$ to a volume of 0.6 mL in the NMR tube. This was then subjected to $^{19}$F NMR analysis. In the same way, DABCO (22.5 mg, 0.2 mmol, 1.0 equiv.), $CS_2$ (76.1 mg, 1 mmol, 5.0 equiv.), $Et_4NBr$ (8.4 mg, 0.04 mmol, 0.2 equiv.) and $Et_3N·3HF$ (60 µL, 0.4 mmol, 2.0 equiv.) were individually added to the same reaction vial and 50 µL were removed each time from the mixture, and diluted to make up to 0.6 mL total in the NMR tube using $CDCl_3$ to measure $^{19}$F NMR. Then, this reaction mixture was electrolysed at 11.11 mA cm$^{-2}$ current density for 7 F mol$^{-1}$ (i.e. 1 h 52 min) using a platinum anode (WE) and platinum cathode (CE). During the reaction, after every 1 F mol$^{-1}$, 50 µL was removed and the $^{19}$F NMR spectra were acquired. The pre-made 1,4-diazabicyclo[2.2.2]octan-1-ium 4-(trifluoromethyl)piperidine-1-carbodithioate (**1m'**), the 4-(trifluoromethyl)piperidine-1-carbothioic dithioperoxyanhydride (**1m"**) and the 4-(trifluoromethyl)piperidine-1-carbothioyl fluoride (**2m)** were also measured in the same way for reference.

### Interaction between the reaction and reagents

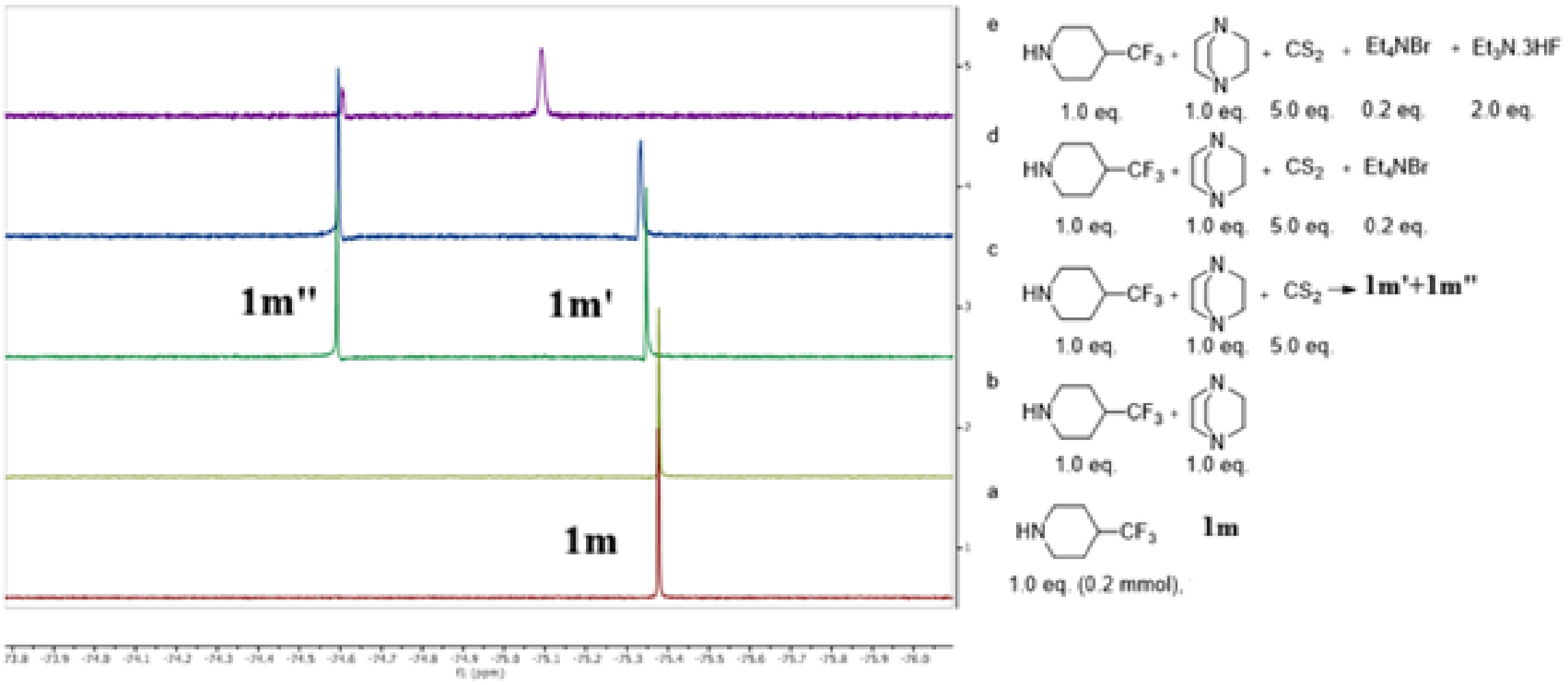


**Supplementary Fig. 12a**: $^{19}$F NMR reaction profile: Addition of each reagent before electrolysis

### Electrochemical reaction analysis for thiocarbamoyl formation 2m ($^{19}$F NMR)

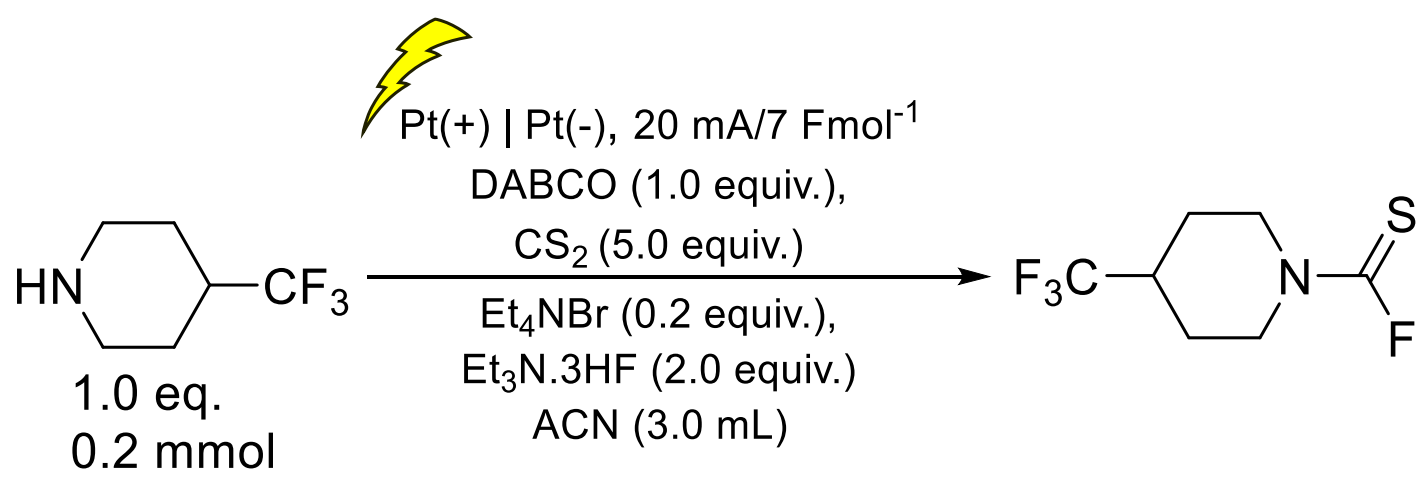


**1m** **2m**

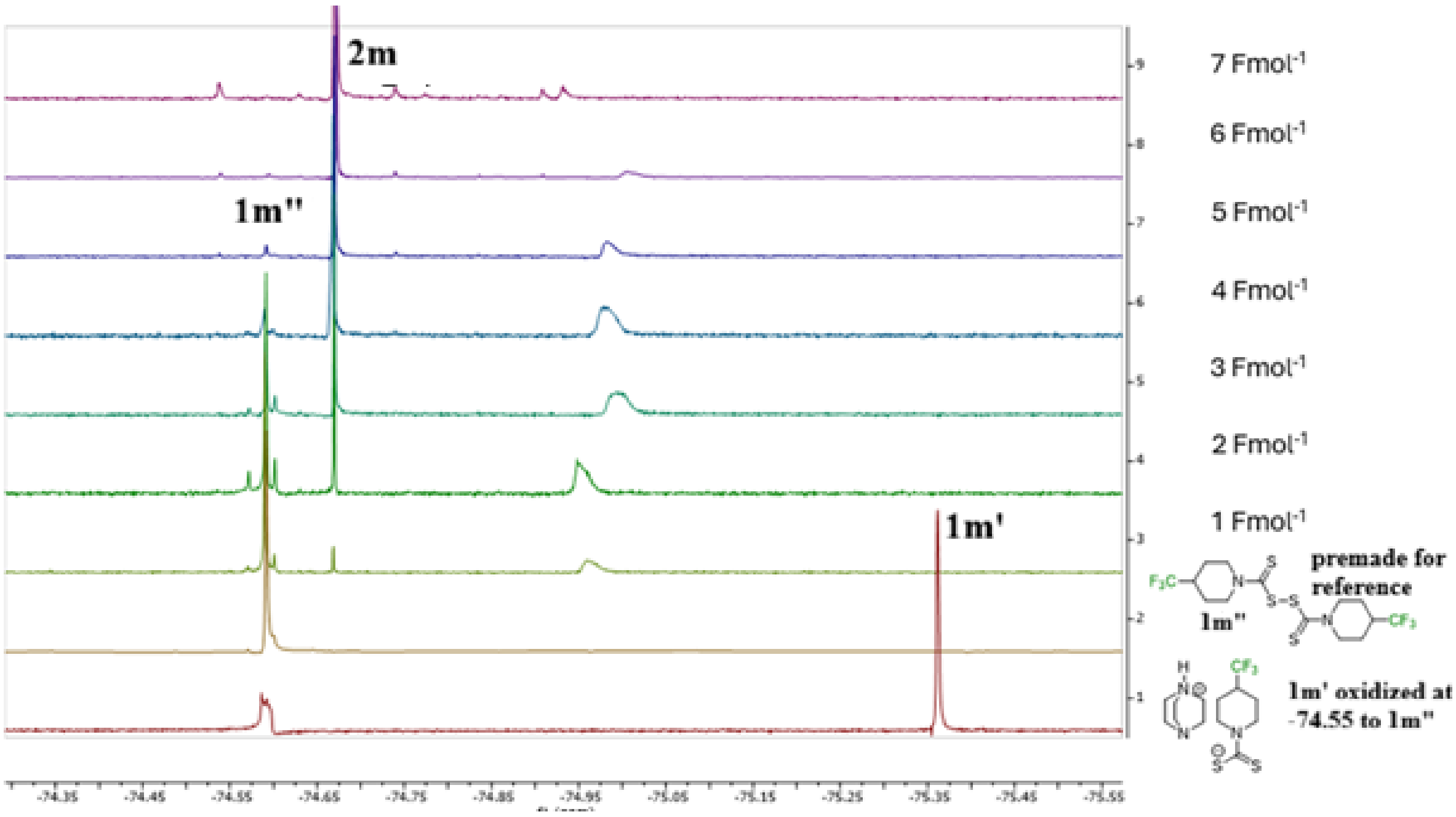


**Supplementary Fig. 12b**: $^{19}$F NMR reaction profile: Electrochemical reaction course from 1 F to 7 F and sample measured at every 1 F mol$^{-1}$.

**Electrochemical reaction analysis for N-trifluoromethyl amine formation 3bb ($^{19}$F NMR)**

4-Trifluoromethylpiperidine (31.1 mg, 0.2 mmol, 1.0 equiv.) and (trifluoromethyl)benzene (9.73 mg, 0.067 M, 0.33 equiv. as internal standard) was dissolved in 3.0 mL of ACN. Then, KOH (11.2 mg, 0.2 mmol, 1.0 equiv.), $CS_2$ (76.1 mg, 1 mmol, 5.0 equiv.), $Et_4NBr$ (42.0 mg, 0.2 mmol, 1.0 equiv.) and $Et_3N{\cdot}3HF$ (98 µL, 0.6 mmol, 3.0 equiv.) were individually added to the same reaction vial and 50 µL were removed each time from the mixture, and diluted to make up to 0.6 mL total in the NMR tube using $CDCl_3$ to measure $^{19}$F NMR. Then, this reaction mixture was electrolysed at 22.22 mA cm$^{-2}$ current density for 25 F mol$^{-1}$ using a platinum anode (WE) and platinum cathode (CE). During the reaction, after every 1 F mol$^{-1}$, 50 µL was removed and the $^{19}$F NMR spectra were acquired.

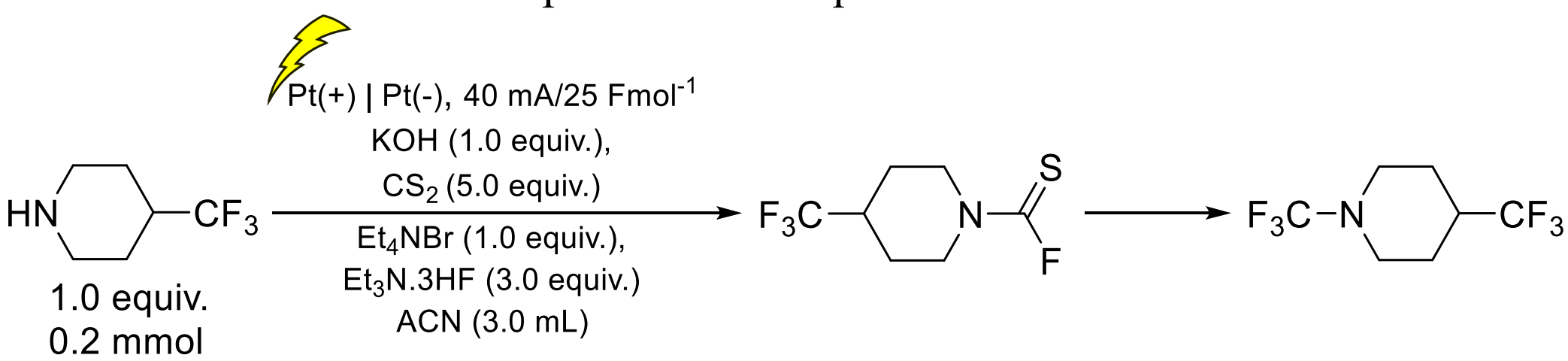


**1m** **2m** **3bb**

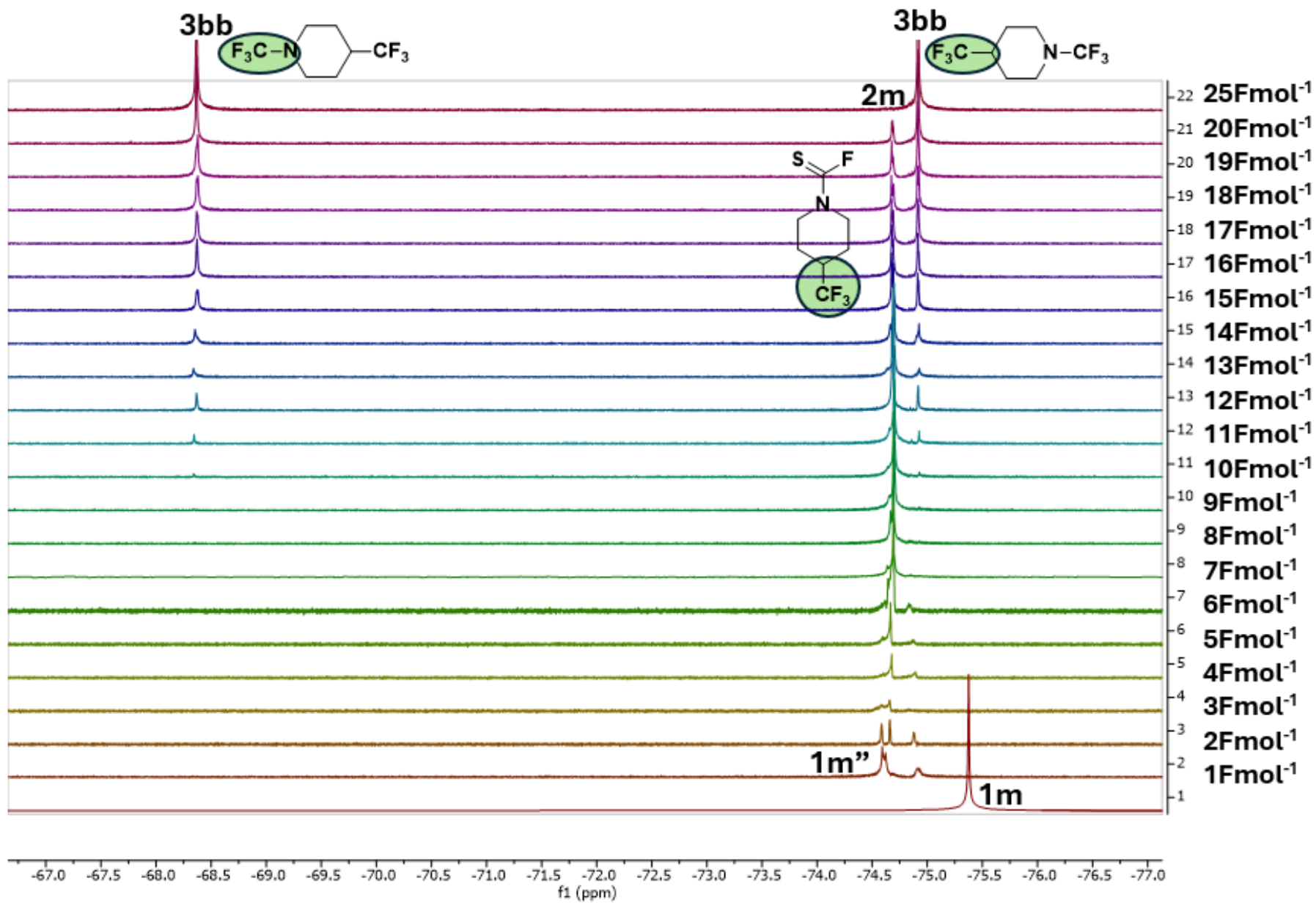


**Supplementary Fig. 13**: $^{19}$F NMR reaction profile: Electrochemical reaction course from 1 F to 25 F and sample measured at every 1 F $mol^{-1}$. (The simultaneous appearance of fluorine peak at lower ppm from -NSCF functional group is not shown here)

## Cyclic Voltammetry

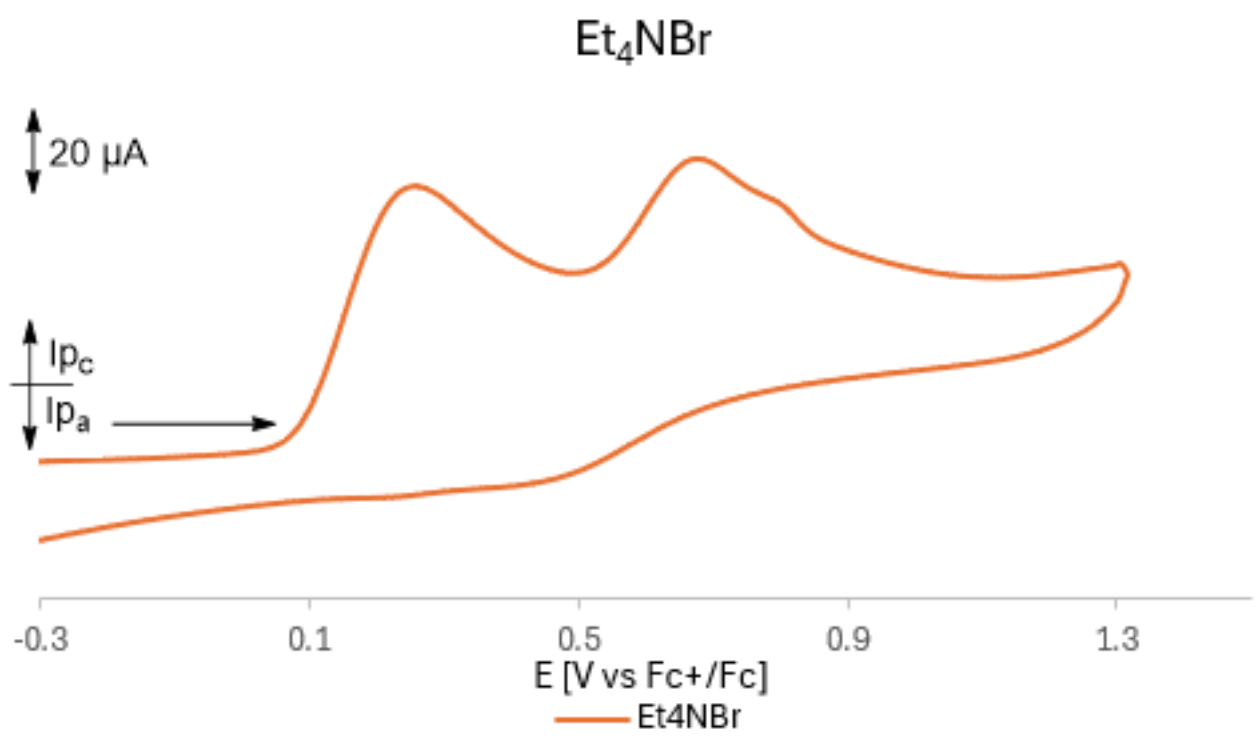


**Supplementary Fig. 14**: Cyclic voltammogram on glassy carbon (diameter 3 mm): Pt wire as the counter electrode; Ag/AgCl as pseudo reference, of a 2 mM solution of tetraethylammonium bromide ($Et_4NBr$) in ACN containing 0.1M of $[NBu_4][PF_6]$, at 0.25 $V.s^{-1}$ at room temperature. IUPAC plotting convention. $Et_4NBr$ exhibits two chemically non-reversible oxidations at $E_{pa}$=0.27 and $E_{pa}$=0.69 vs $Fc^+/Fc$

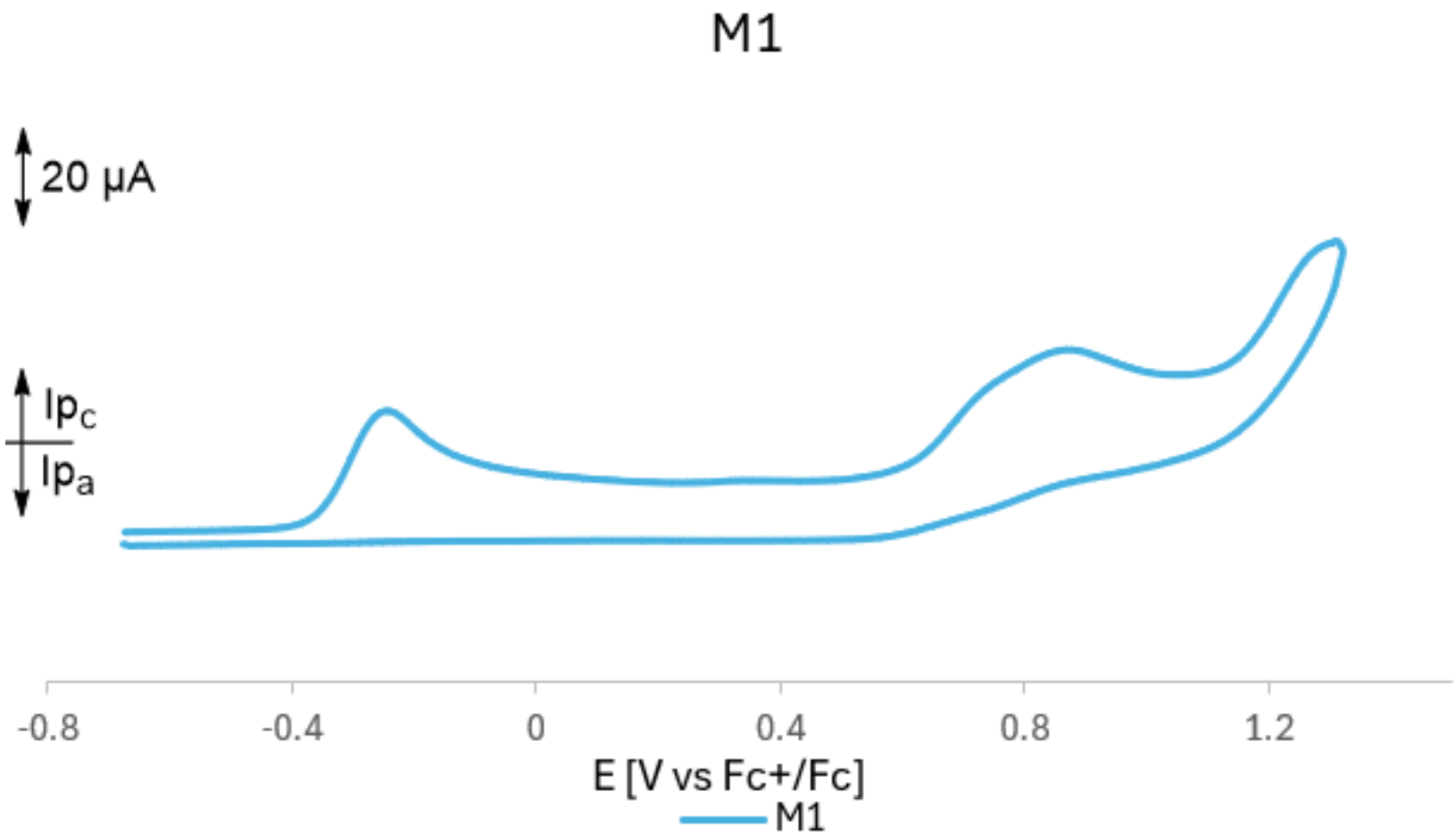


**Supplementary Fig. 15:** Cyclic voltammogram on glassy carbon (diameter 3 mm): Pt wire as the counter electrode; Ag/AgCl as pseudo reference, of a 2 mM solution of 1,4-diazabicyclo[2.2.2]octan-1-ium dibenzylcarbamodithioate (**M1**) in ACN containing 0.1M of $[NBu_4][PF_6]$, at 0.25 $V.s^{-1}$ at room temperature. IUPAC plotting convention. 1,4-diazabicyclo[2.2.2]octan-1-ium dibenzylcarbamodithioate (**M1**), exhibits two chemically non-reversible oxidation at $E_{pa}$=-0.23 and $E_{pa}$=0.75 vs $Fc^{+}$/Fc

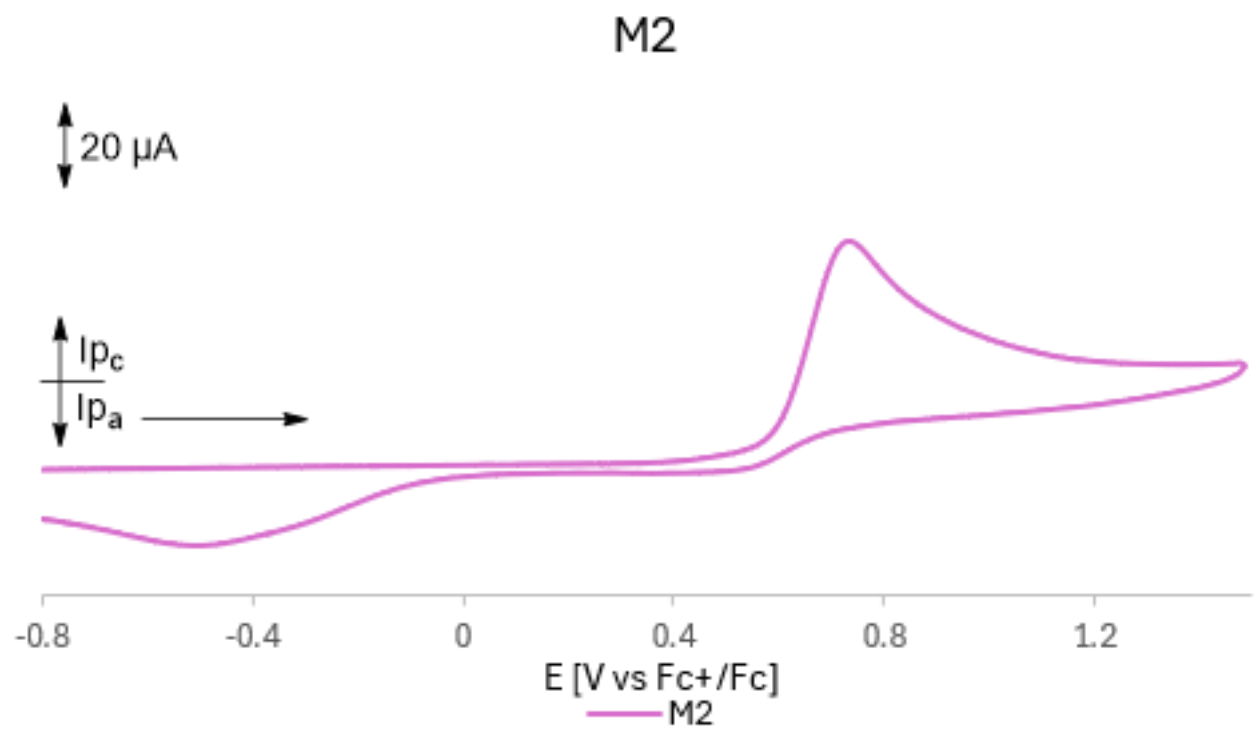


**Supplementary Fig. 16**: Cyclic voltammogram on glassy carbon (diameter 3 mm): Pt wire as the counter electrode; Ag/AgCl as pseudo reference, of a 2 mM solution of tetrabenzylthiuram disulfide (**M2**), in ACN containing 0.1 M of $[NBu_4][PF_6]$, at 0.25 $V.s^{-1}$ at room temperature. IUPAC plotting convention. tetrabenzylthiuram disulfide (**M2**), exhibits a chemically non-reversible reduction at $E_{pa}$=0.75 vs $Fc^{+}$/Fc

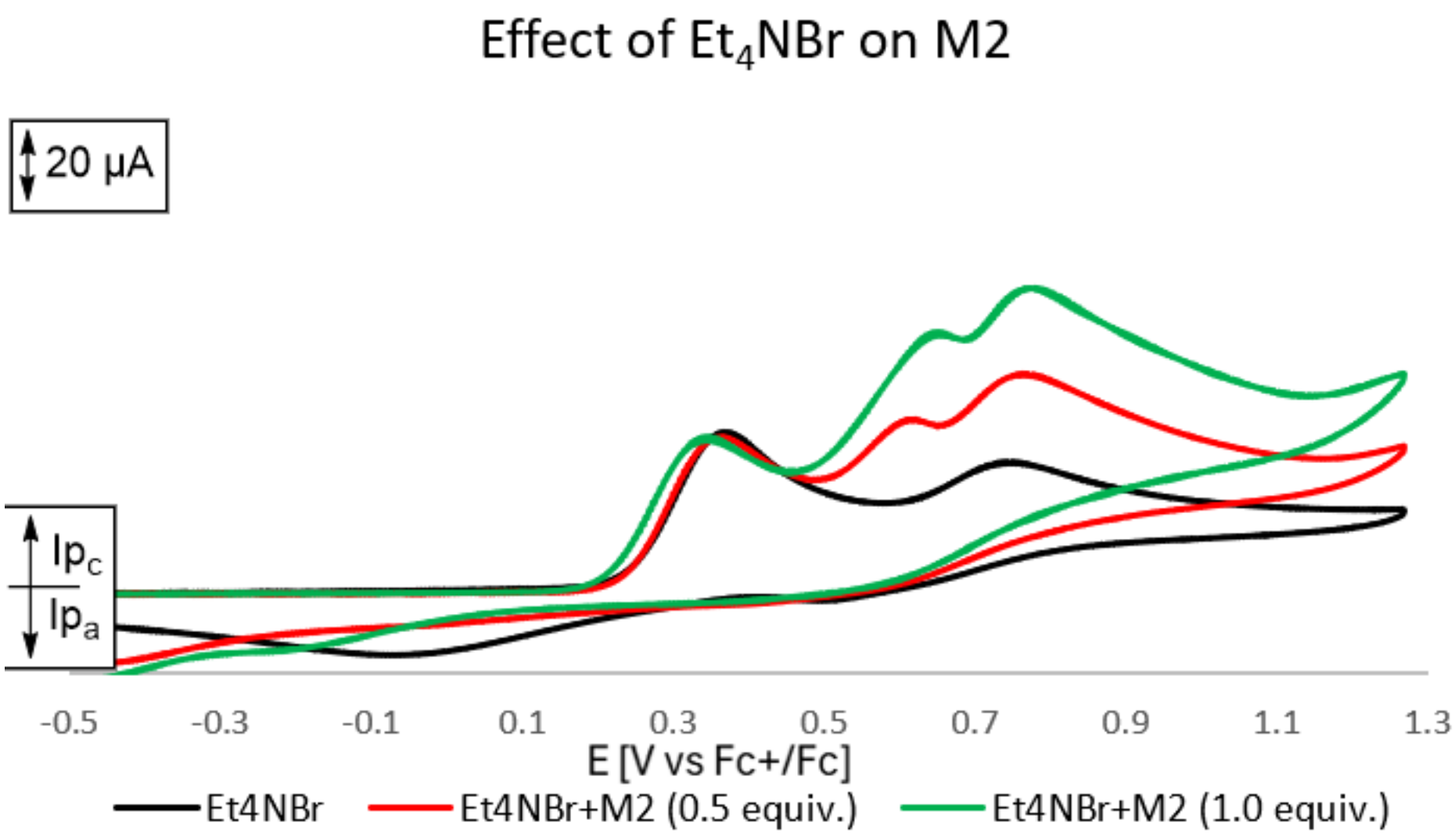


**Supplementary Fig. 17**: Cyclic voltammogram on glassy carbon (diameter 3 mm): Pt wire as the counter electrode; Ag/AgCl as pseudo reference, of a 2 mM solution of tetraethylammonium bromide in ACN containing 0.1M of $[NBu_4][PF_6]$ titrated with tetrabenzylthiuram disulfide **M2 (0.5 and 1.0 equiv.)** at 0.25 $V.s^{-1}$ at room temperature. IUPAC plotting convention.

### Tempo radical trap experiment

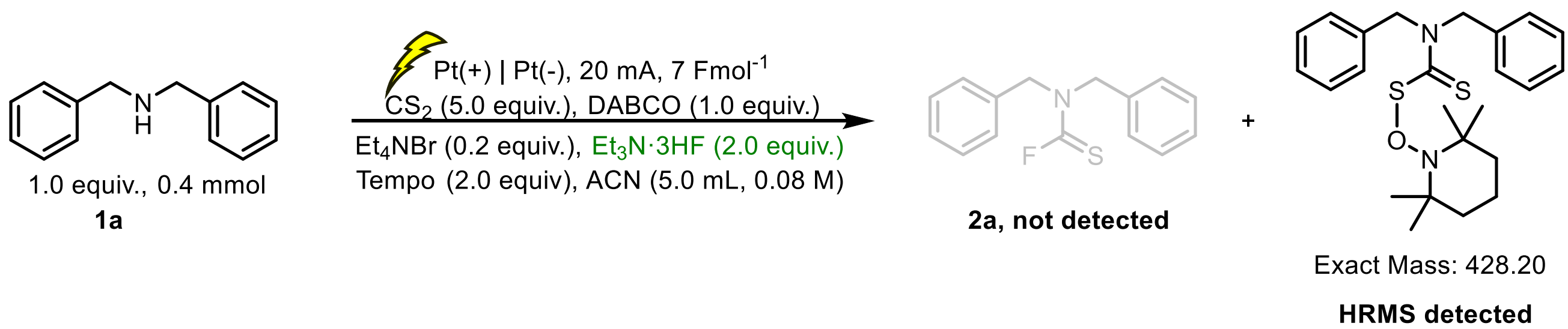


According to our standard reaction conditions amine (**1**, 0.4 mmol, 1.0 equiv.), DABCO (45.0 mg, 0.4 mmol, 1.0 equiv.) and $CS_2$ (152 mg, 2.0 mmol, 5.0 equiv.) were added into a 5 mL one compartment Electrasyn vial to generate in situ salt. Then acetonitrile (ACN) (5 mL, 0.08 M), $Et_4NBr$ (16.8 mg, 0.08 mmol, 0.2 equiv.), $Et_3N{\cdot}3HF$ (120 µL, 0.8 mmol, 2.0 equiv.) and Tempo (125 mg, 0.8 mmol, 2.0 equiv.) were added. The solution was then electrolyzed at 11.11 mA $cm^{-2}$ for 7 F $mol^{-1}$ (i.e. 3.75 h) using a platinum anode (WE) and platinum cathode (CE). After 7 F $mol^{-1}$ the solvent was removed under reduced pressure and subjected to HRMS analysis to observe the tempo adduct.

HRMS (EI) m/z: $[M+H]^+$ calcd for $C_{24}H_{33}N_2OS_2$ 429.2034, found 429.2025.

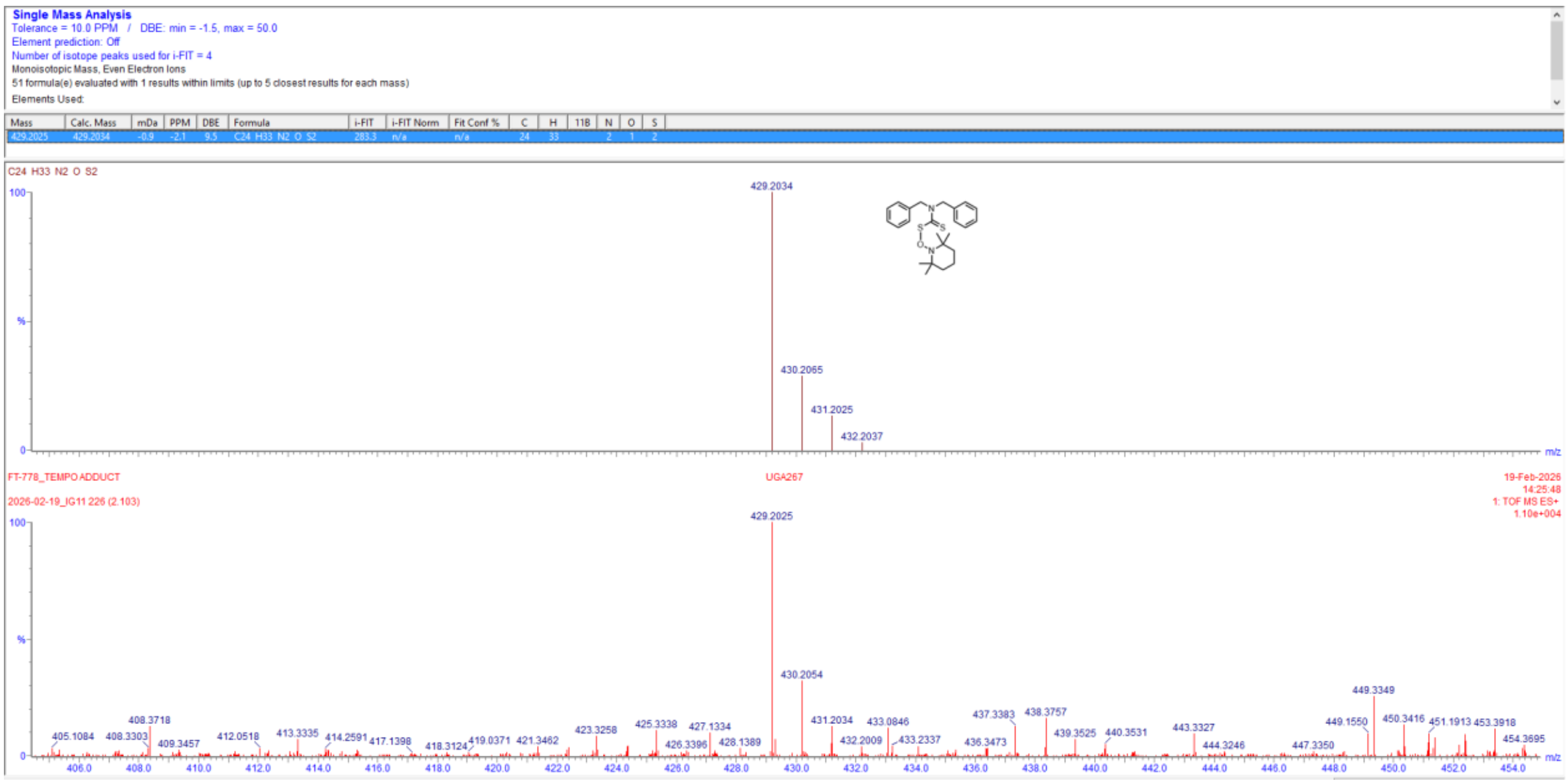


**Supplementary Fig. 18:** HRMS analysis: Detection of TEMPO adduct

**Study on the effect and role of $Et_4NBr$ during electrochemical N,N-dialkylthiocarbamoyl fluoride synthesis[a]**

With $Et_4NBr$ (qNMR: 75%)

Without $Et_4NBr$ (qNMR: 13%)

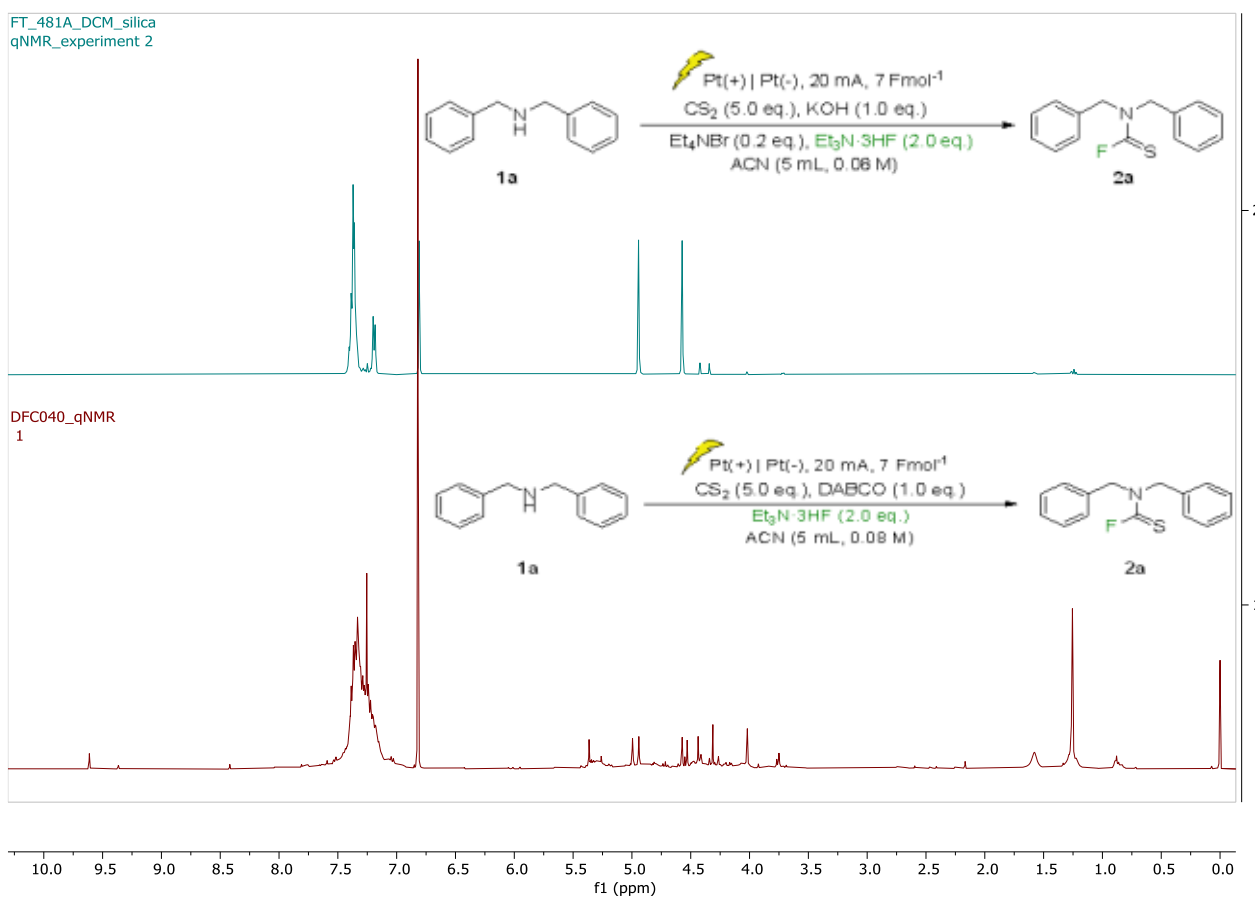


**Supplementary Fig. 19**: Role of $Et_4NBr$: Reaction in the presence and absence of $Et_4NBr$

Dibenzylamine **1a** (39.5 mg, 0.2 mmol, 1.0 equiv.), KOH or DABCO (11.2 or 22.4 mg, 0.2 mmol, 1.0 equiv.), carbon disulfide [$(CS_2)$, 76.1 mg, 1.0 mmol, 5.0 equiv.], acetonitrile [(ACN) 3 mL, 0.067 M)], $Et_4NBr$ (8.4 mg, 0.04 mmol, 0.2 equiv.), $Et_3N\cdot 3HF$ (64.5 mg, 0.4 mmol, 2.0 equiv.) IS: Bromoform (6.82 ppm).

**Plausible mechanism**

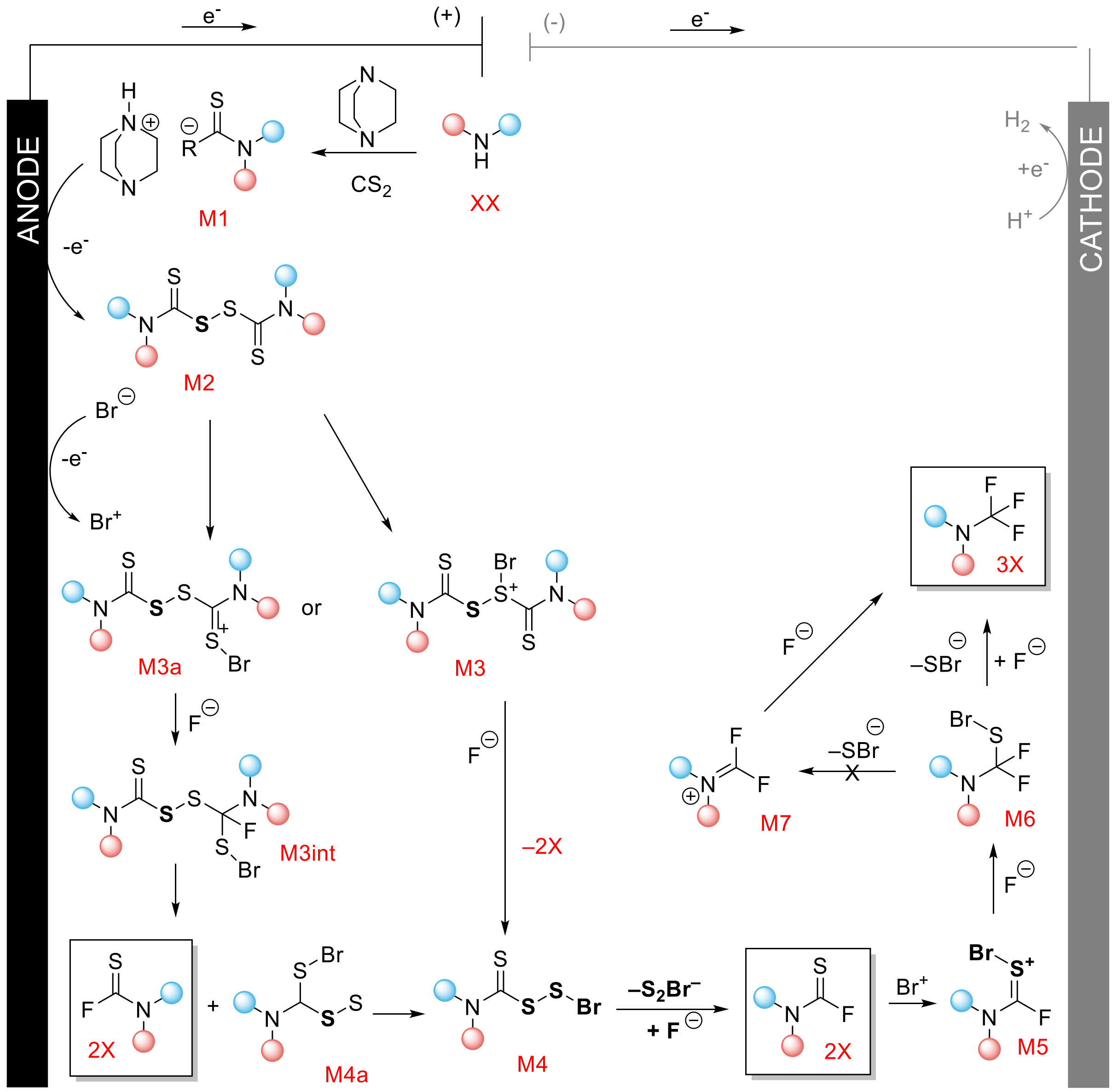


**Supplementary Fig. 20**: Plausible mechanism: Schematic representation of reaction mechanism.

**Elemental Analysis of solid residue of the reaction mixture**

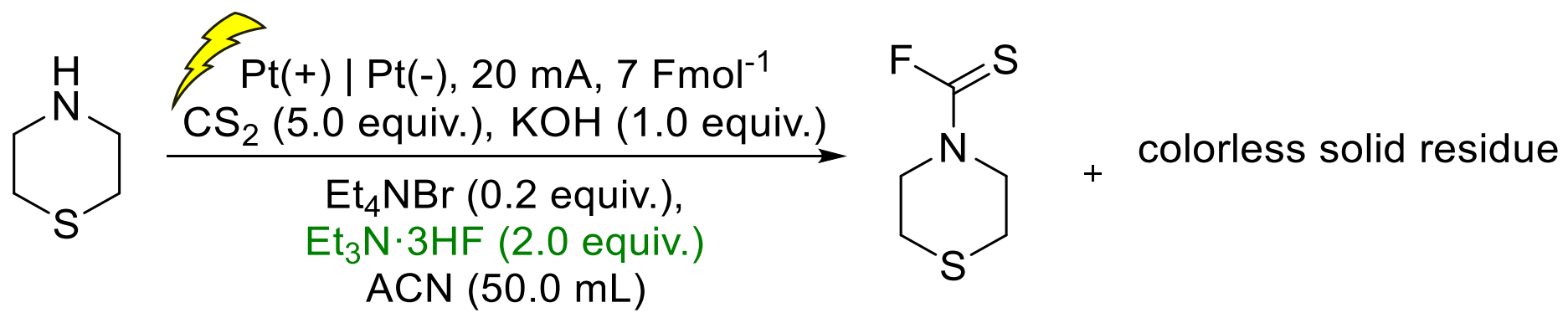


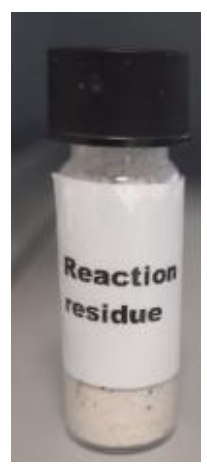


**1d** **2d**

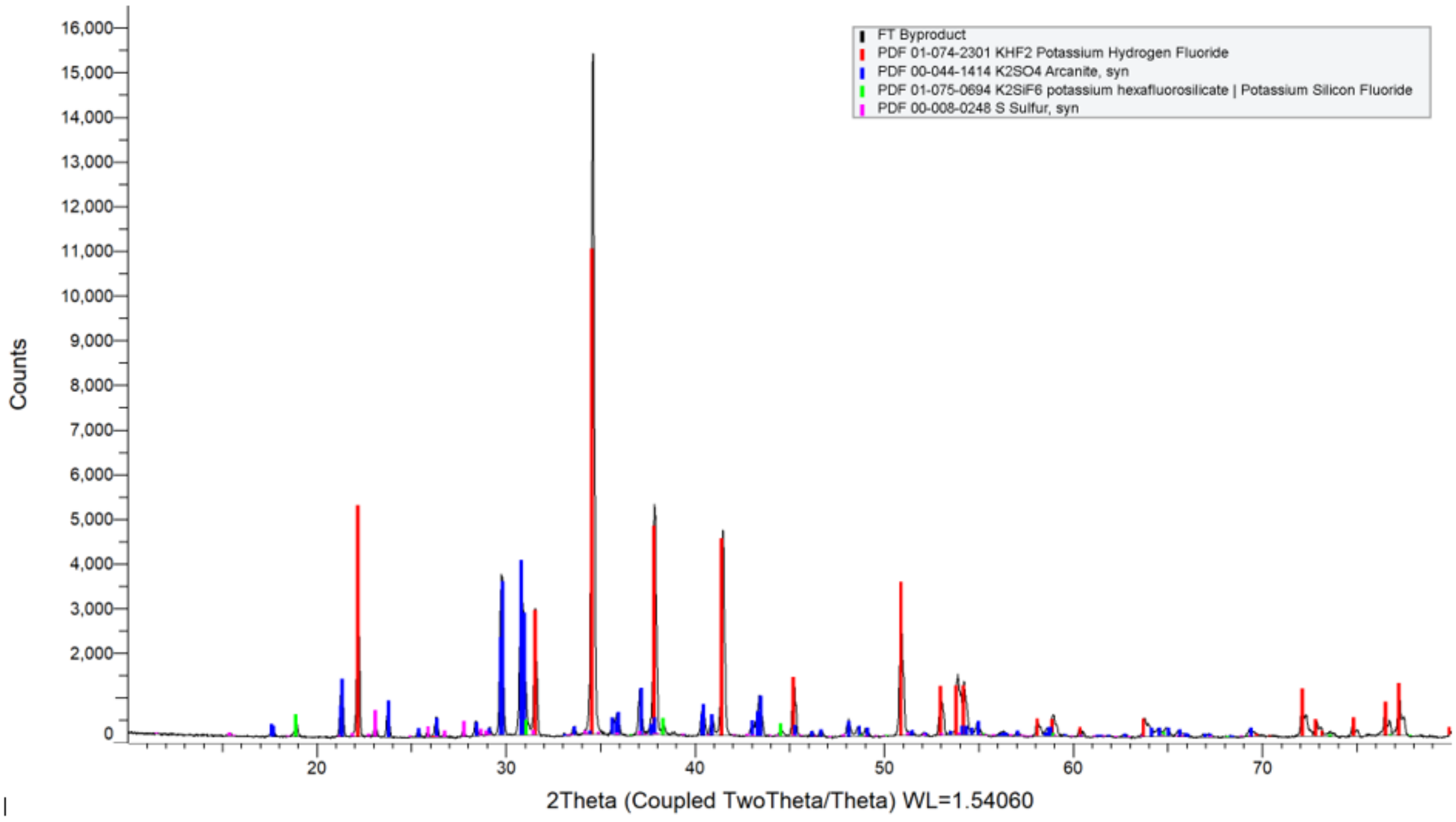


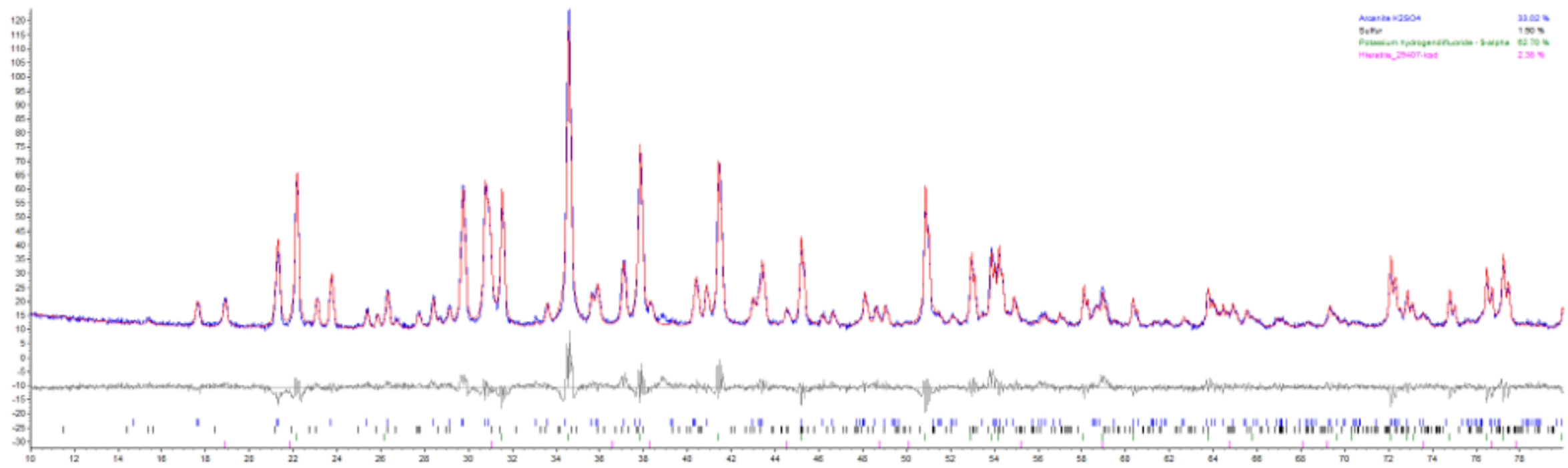

```
Range Number :   1

R-Values

Rexp : 5.32    Rwp : 15.50    Rp  : 12.16   GOF : 2.92
Rexp`: 4.82    Rwp`: 14.05    Rp` : 11.53   DW  : 0.49

Quantitative Analysis - Rietveld
   Phase 1  : "Arcanite K2SO4"               33.021 %
   Phase 2  : Sulfur                         1.903 %
   Phase 3  : "Potassium hydrogendifluoride - $-alpha"   62.697 %
   Phase 4  : Hieratite_29407-icsd           2.379 %
```

**Supplementary Fig. 21**: Powder XRD data: Elemental analysis of solid residue of the reaction mixture

## SEM-EDX Analysis

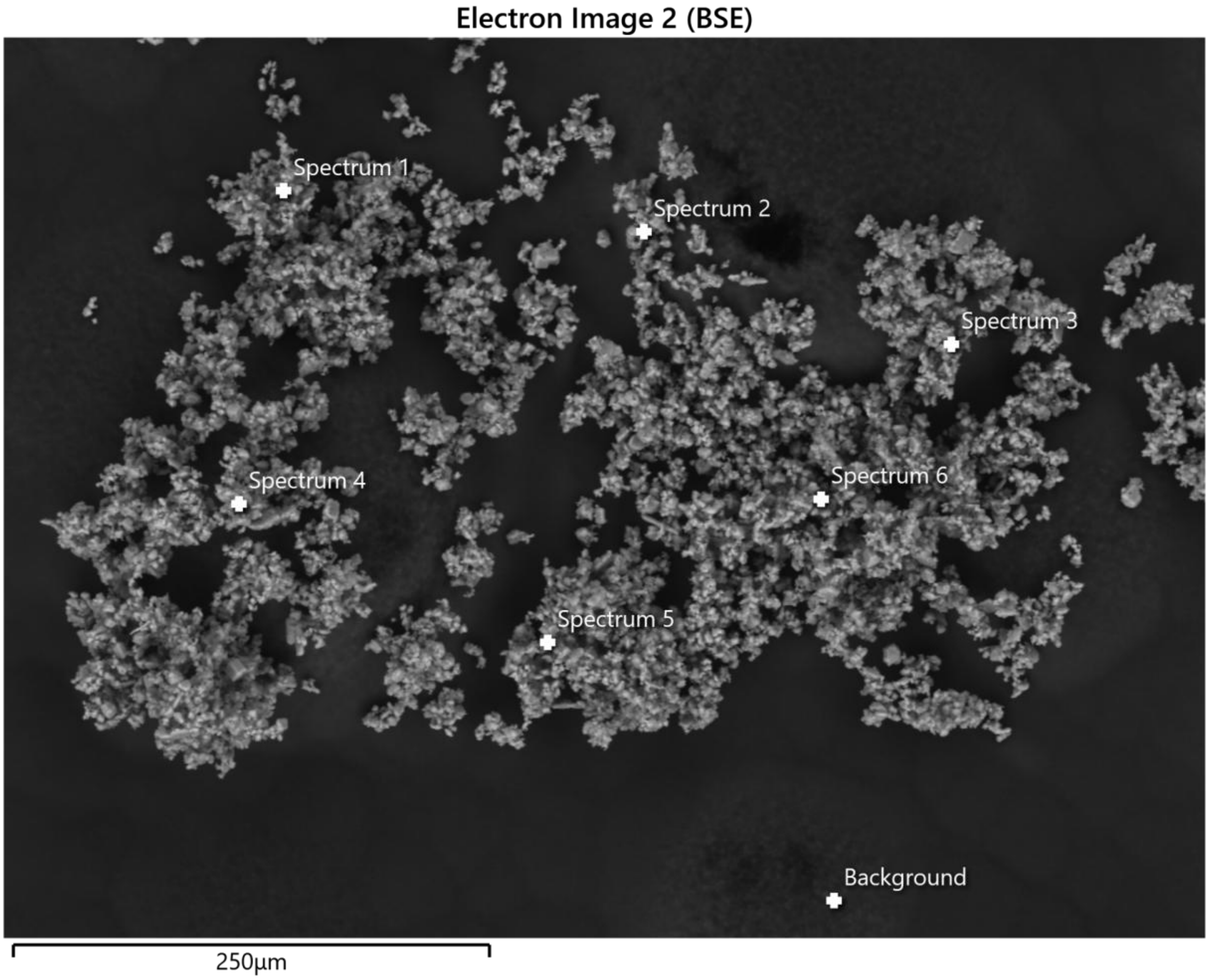


**Supplementary Fig. 22**: Electron image

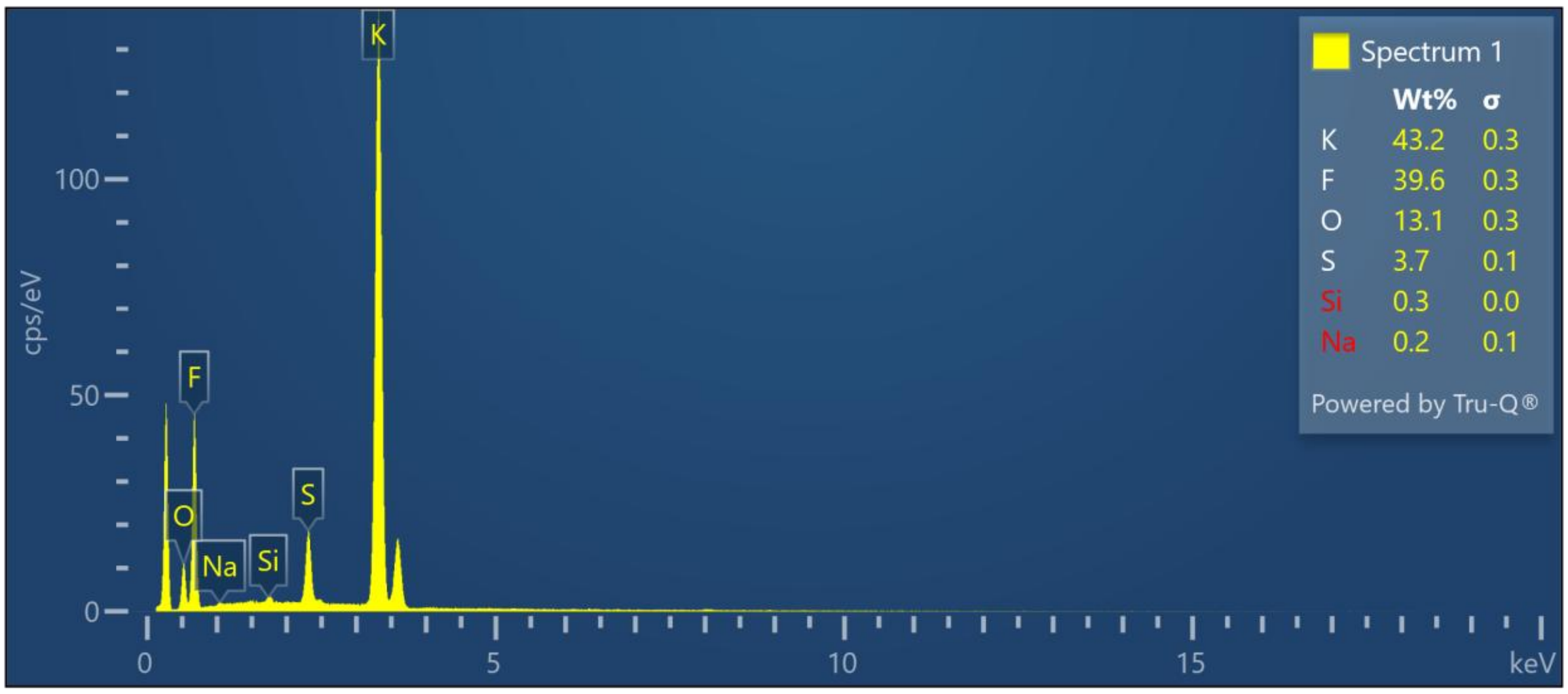


**Supplementary Fig. 23**: Element composition spectra

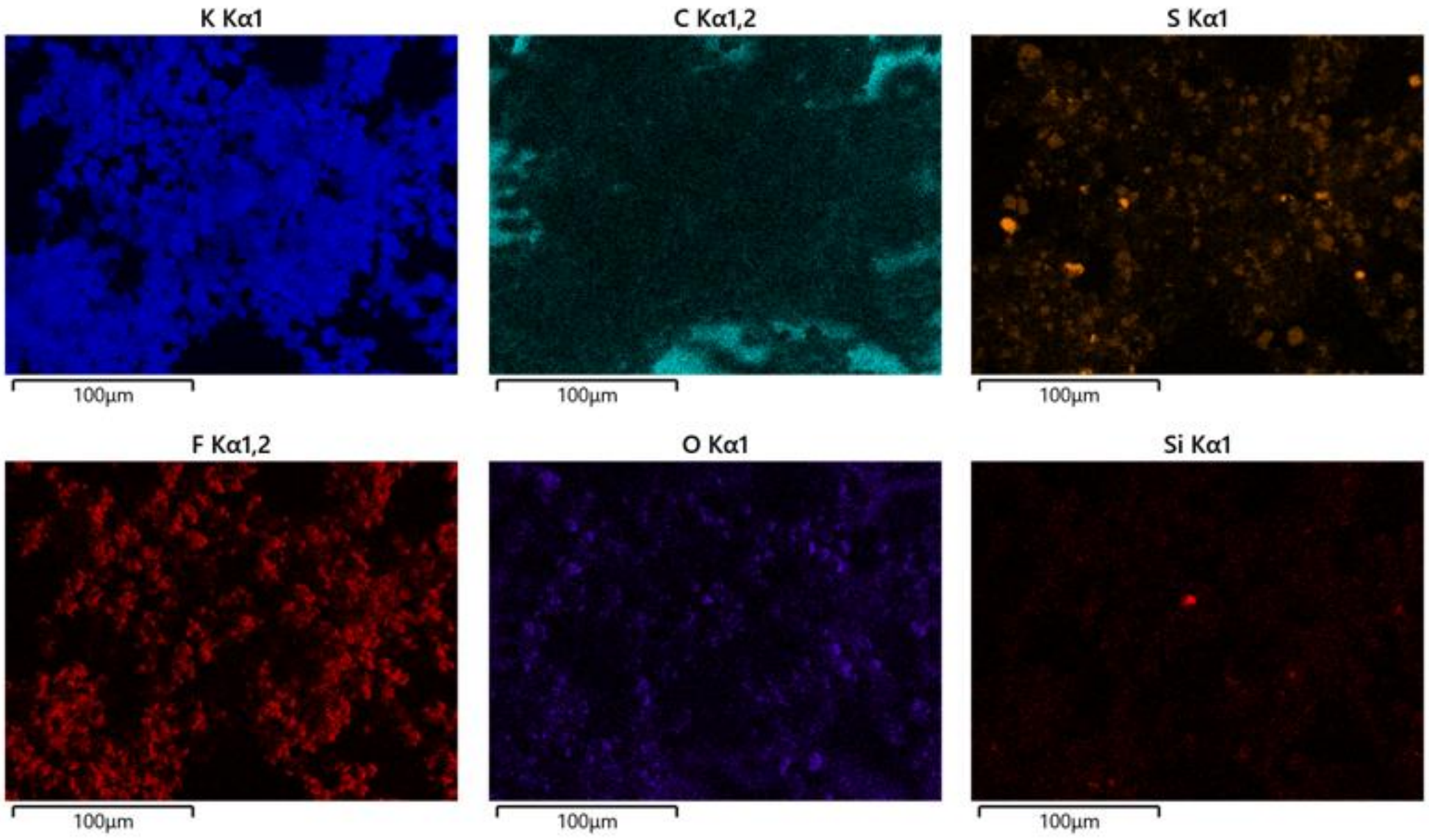


**Supplementary Fig. 24**: Elemental analysis

## Supplementary DFT calculations

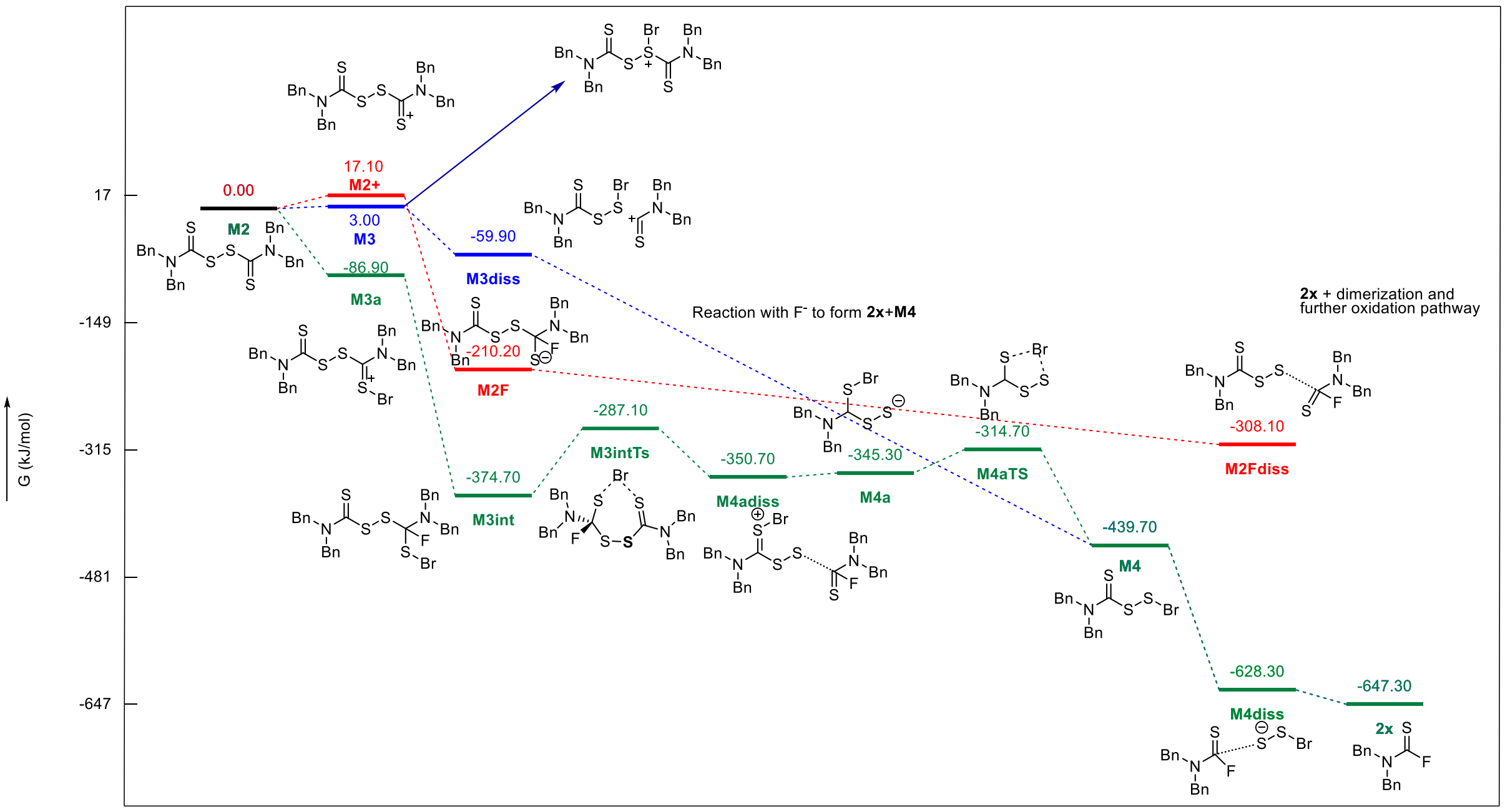


**Supplementary Fig. 25**: Gibbs energy profile: Demonstrating the formation of **2X** from **M2** via three distinct pathways. **Red trace**: oxidation of **M2** and subsequent reaction with **F⁻**. **Blue trace**: Oxidation of $Br^-$ to $Br^+$ and reaction with **M2** via attack on the perthio (C-S-S-C) bridge. **Green trace**: Oxidation of $Br^-$ to $Br^+$ and reaction with **M2** via attack on the thioketone (C=S) group. (F=fluorine, int= intermediate, TS= transition state, diss= dissociation).

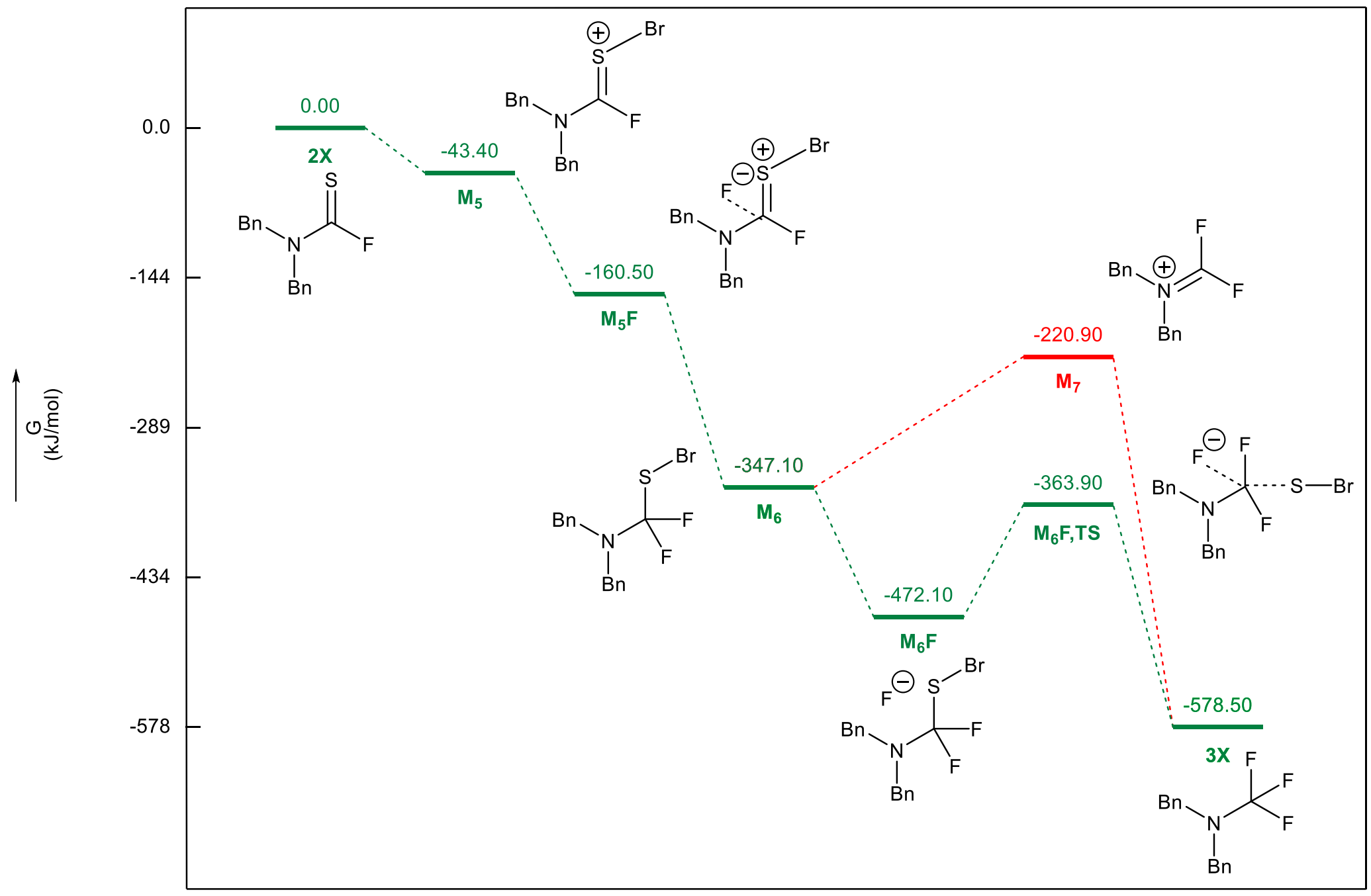


**Supplementary Fig. 26**: Gibbs energy profile: Demonstrating the formation of **3X** from **2X** via two distinct pathways. **Red trace**: Oxidation of $Br^-$ to $Br^+$ and reaction with **2X**. Formation of **3X** via $SBr^-$ dissociation. Green trace: Oxidation of $Br^-$ to $Br^+$ and reaction with **2X**. Formation of **3X** via concerted association of $F^-$ and dissociation of $SBr^-$.

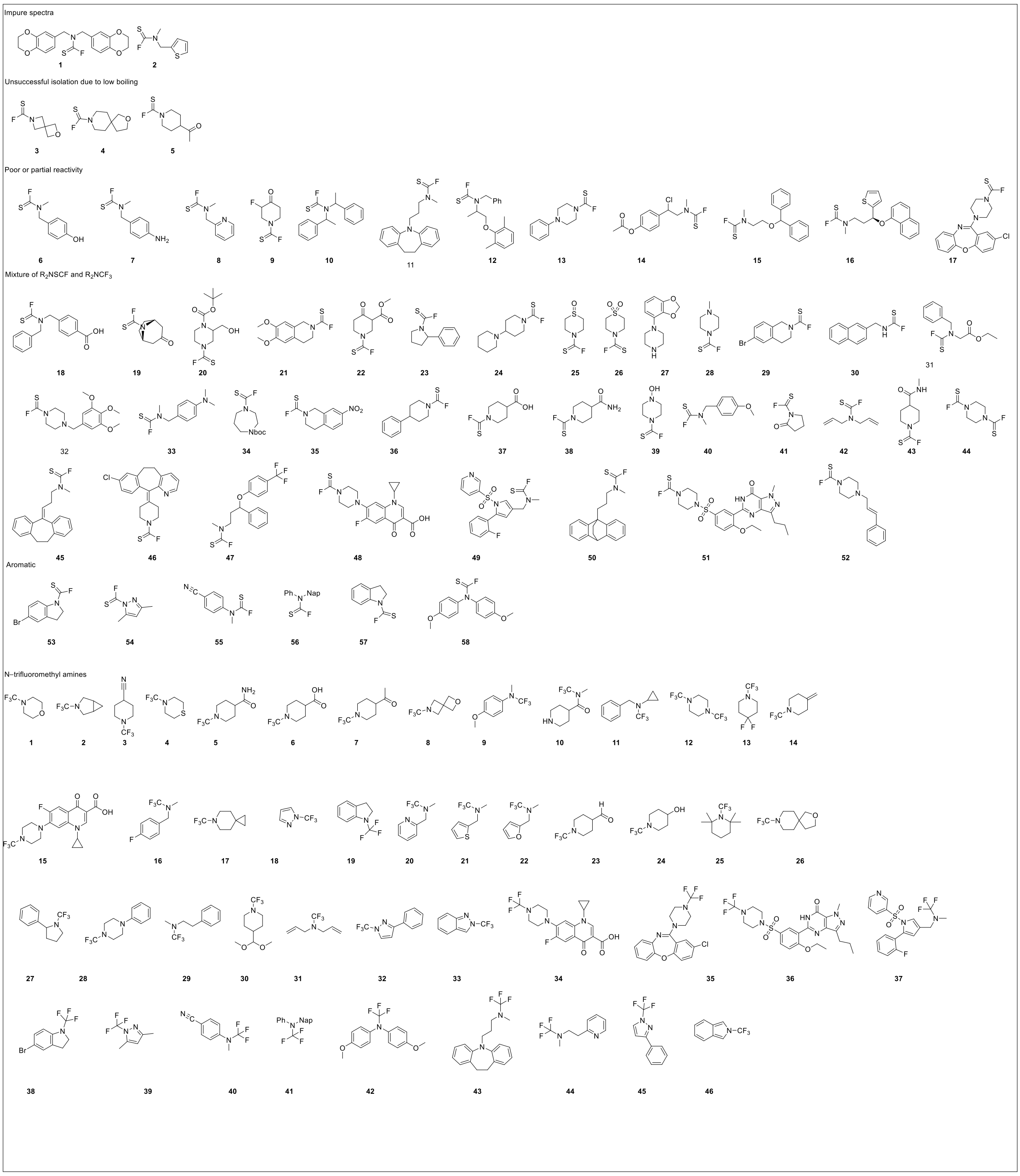


**Supplementary Fig. 27: Unsuccessful substrates involved in $R_2NCSF$ and N-$CF_3$ formation**

**NMR Spectra:**

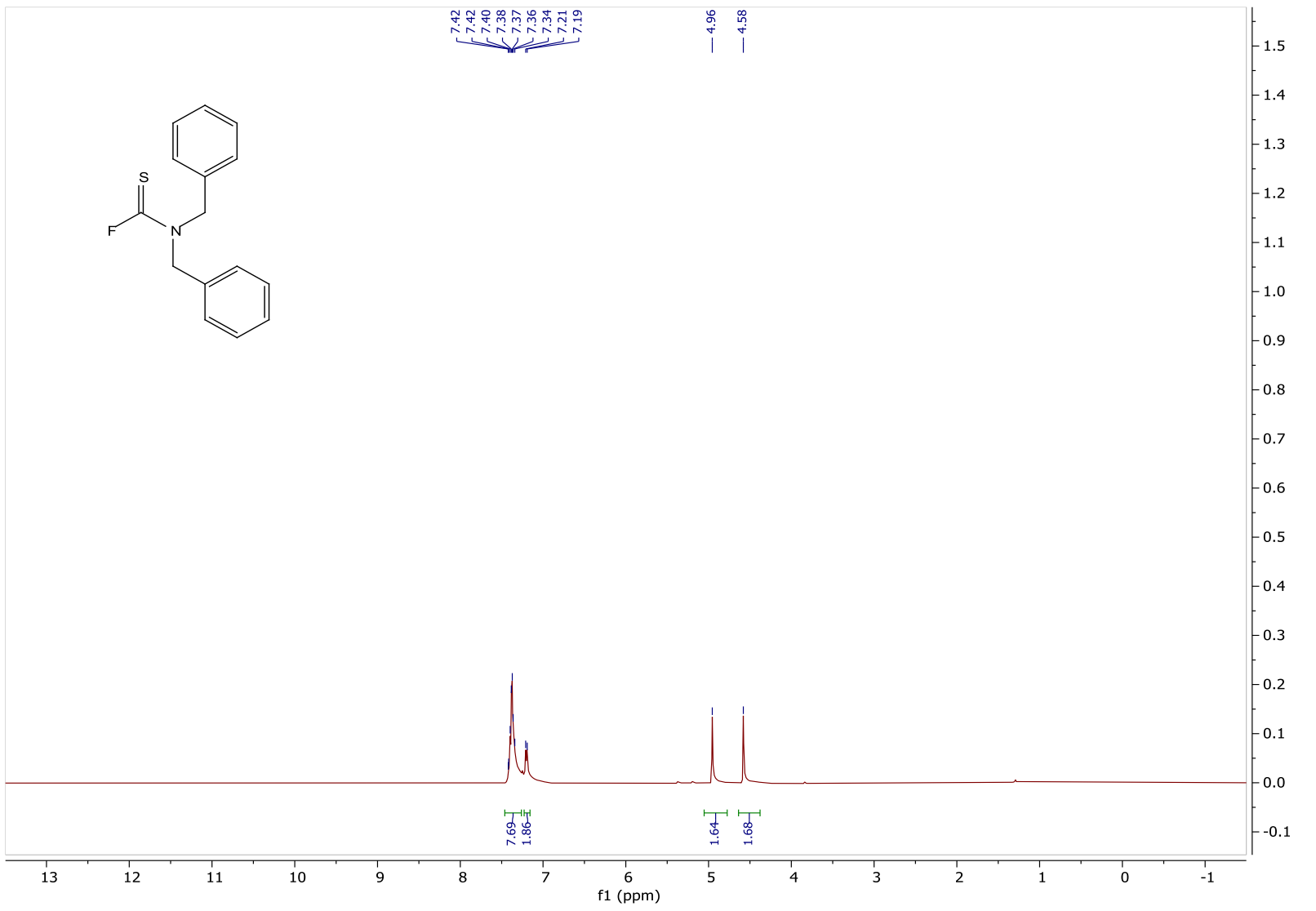

$^{1}$H NMR (400 MHz, $CDCl_3$) of **2a**

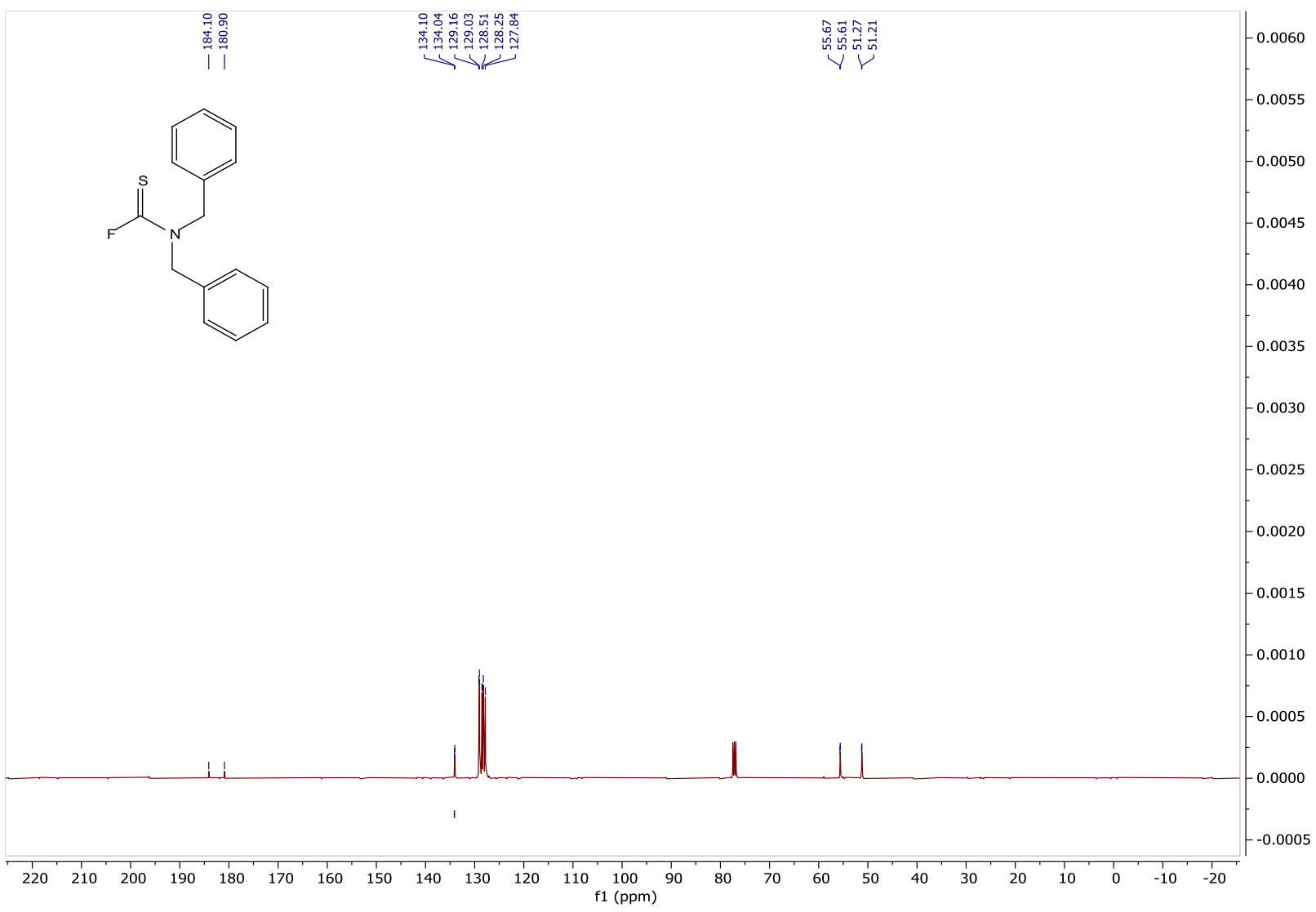

$^{13}$C NMR (101 MHz, $CDCl_3$) of **2a**

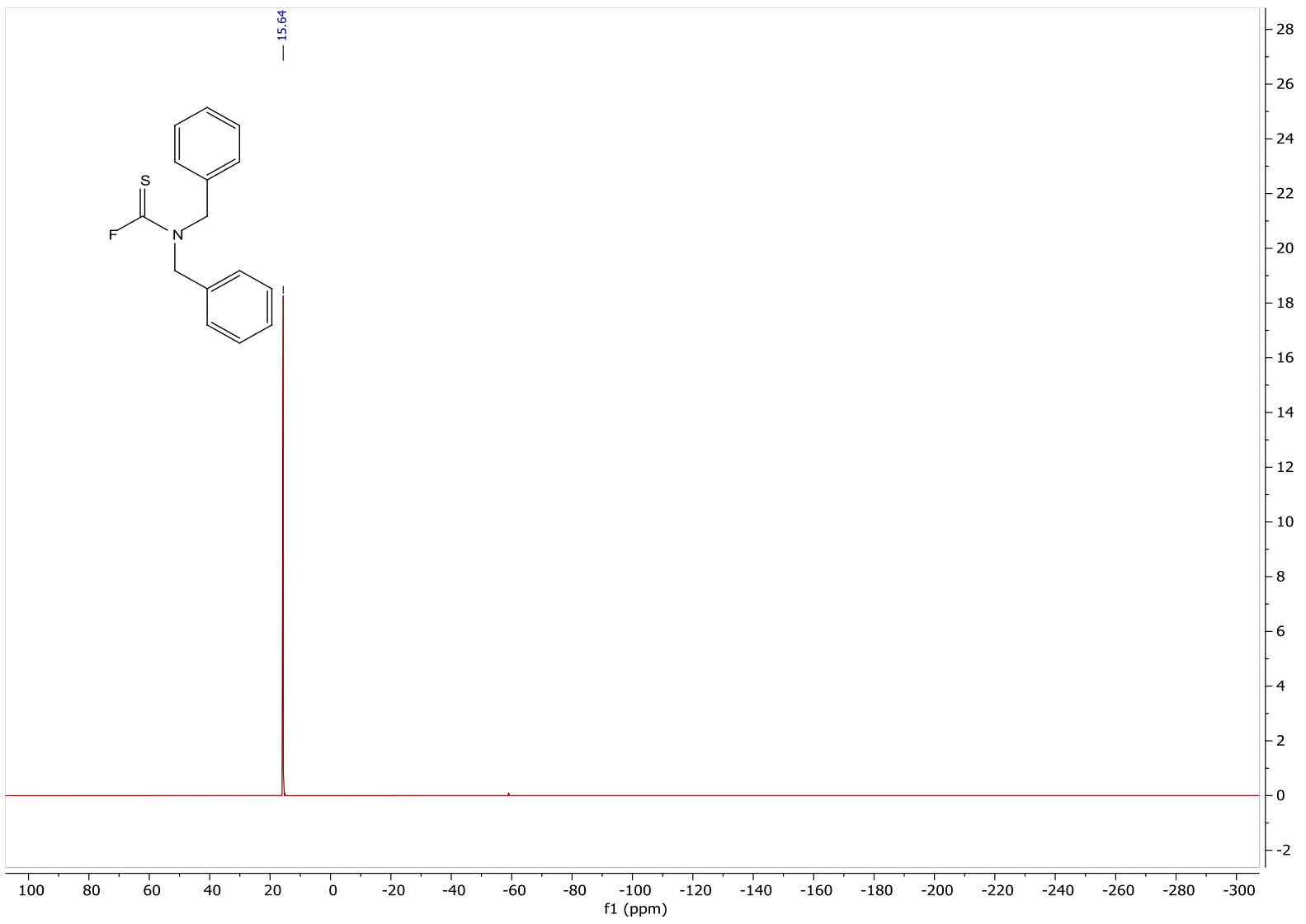

$^{19}$F NMR (376 MHz, $CDCl_3$) of **2a**.

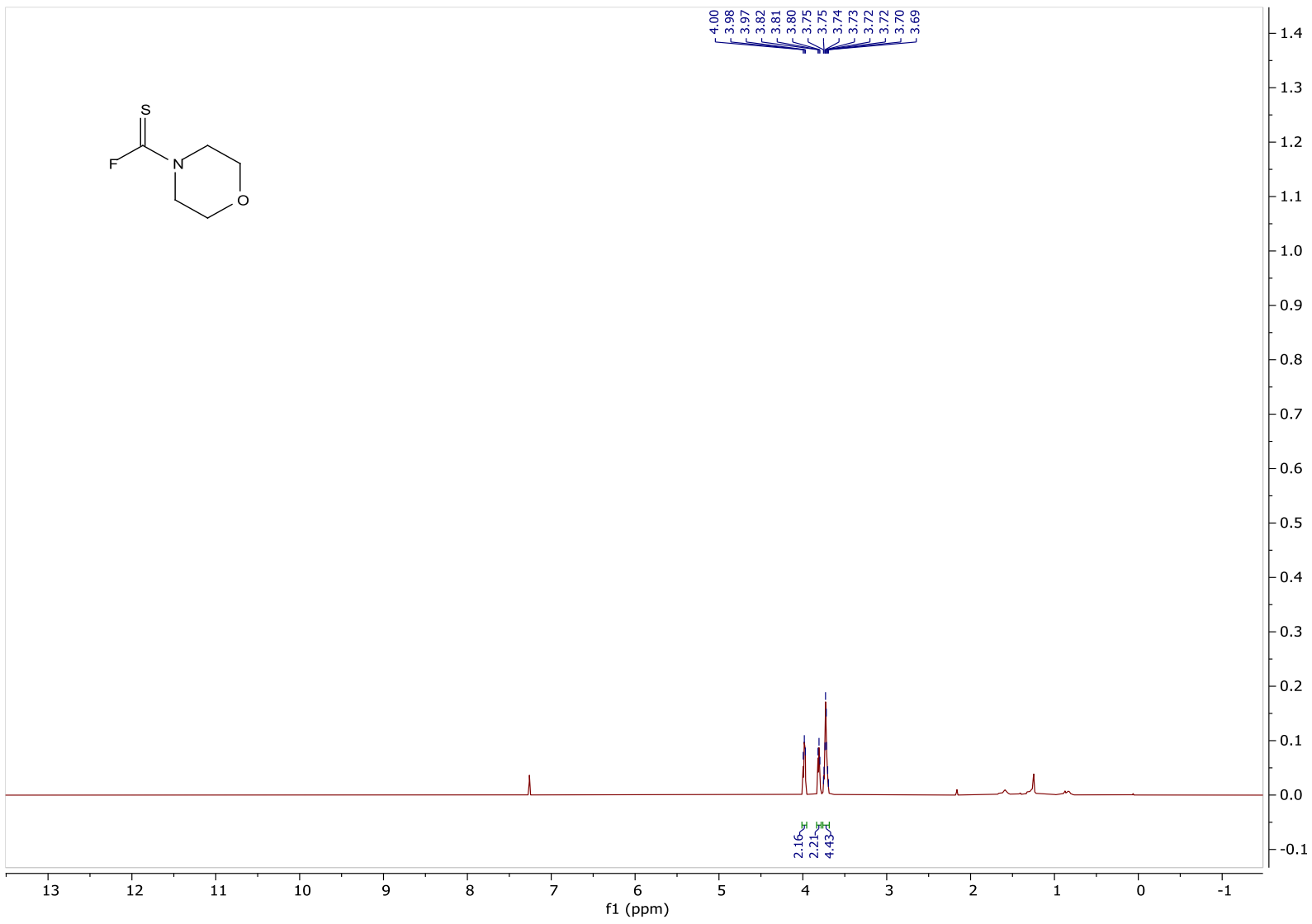

$^{1}$H NMR (400 MHz, $CDCl_3$) of **2b**

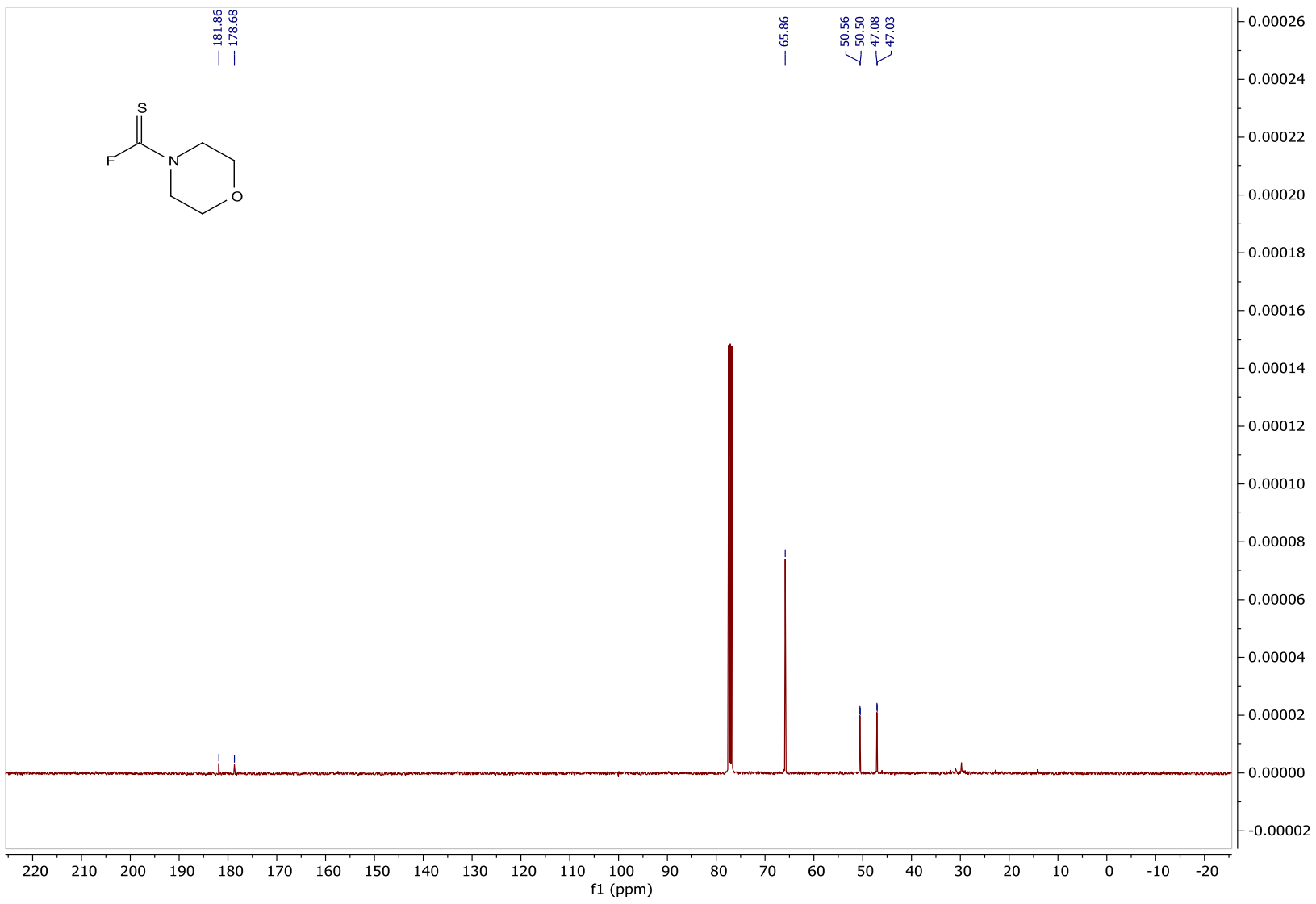

$^{13}C$ NMR (101 MHz, $CDCl_3$) δ of **2b**

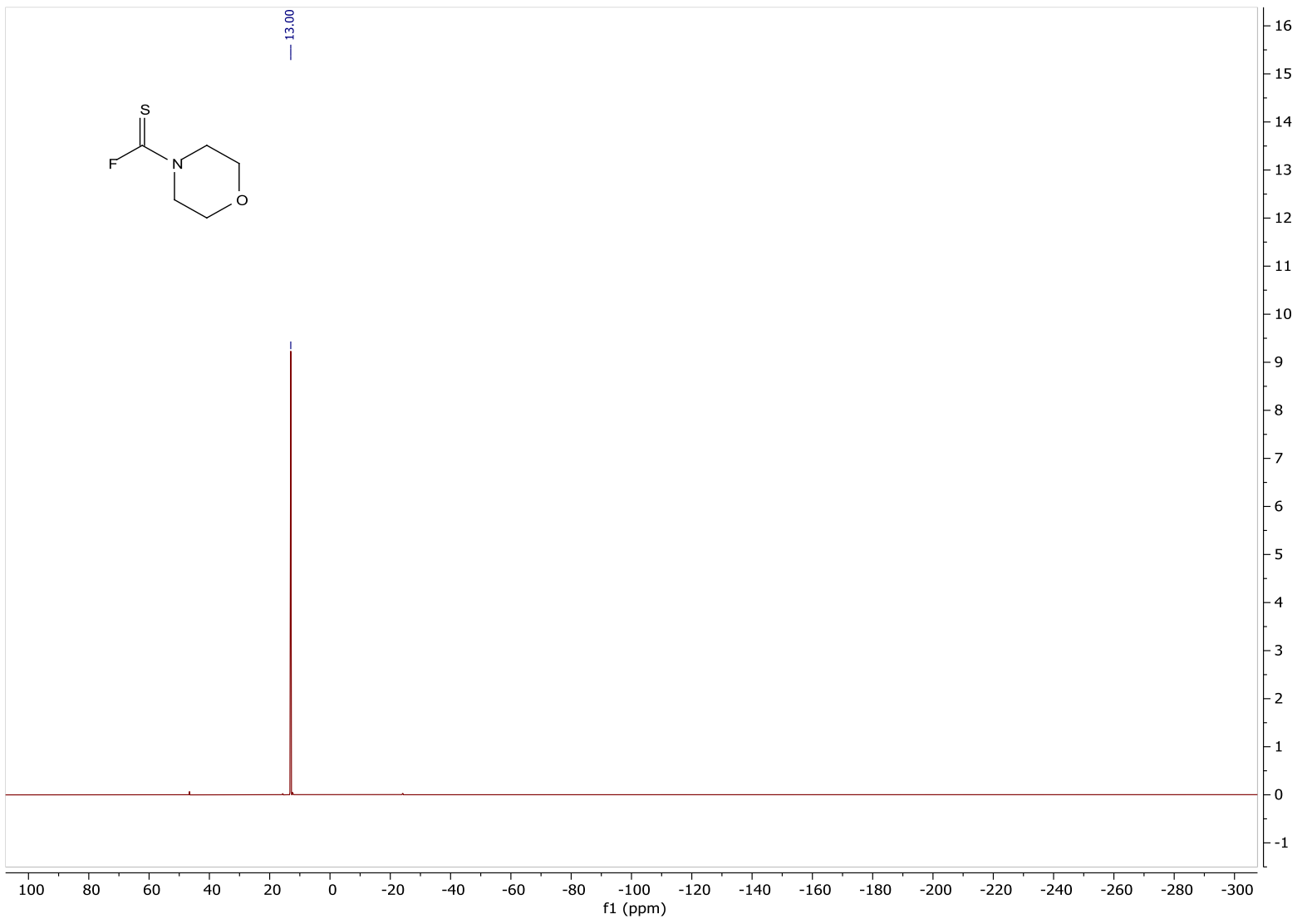

$^{19}F$ NMR (376 MHz, $CDCl_3$) δ of **2b**

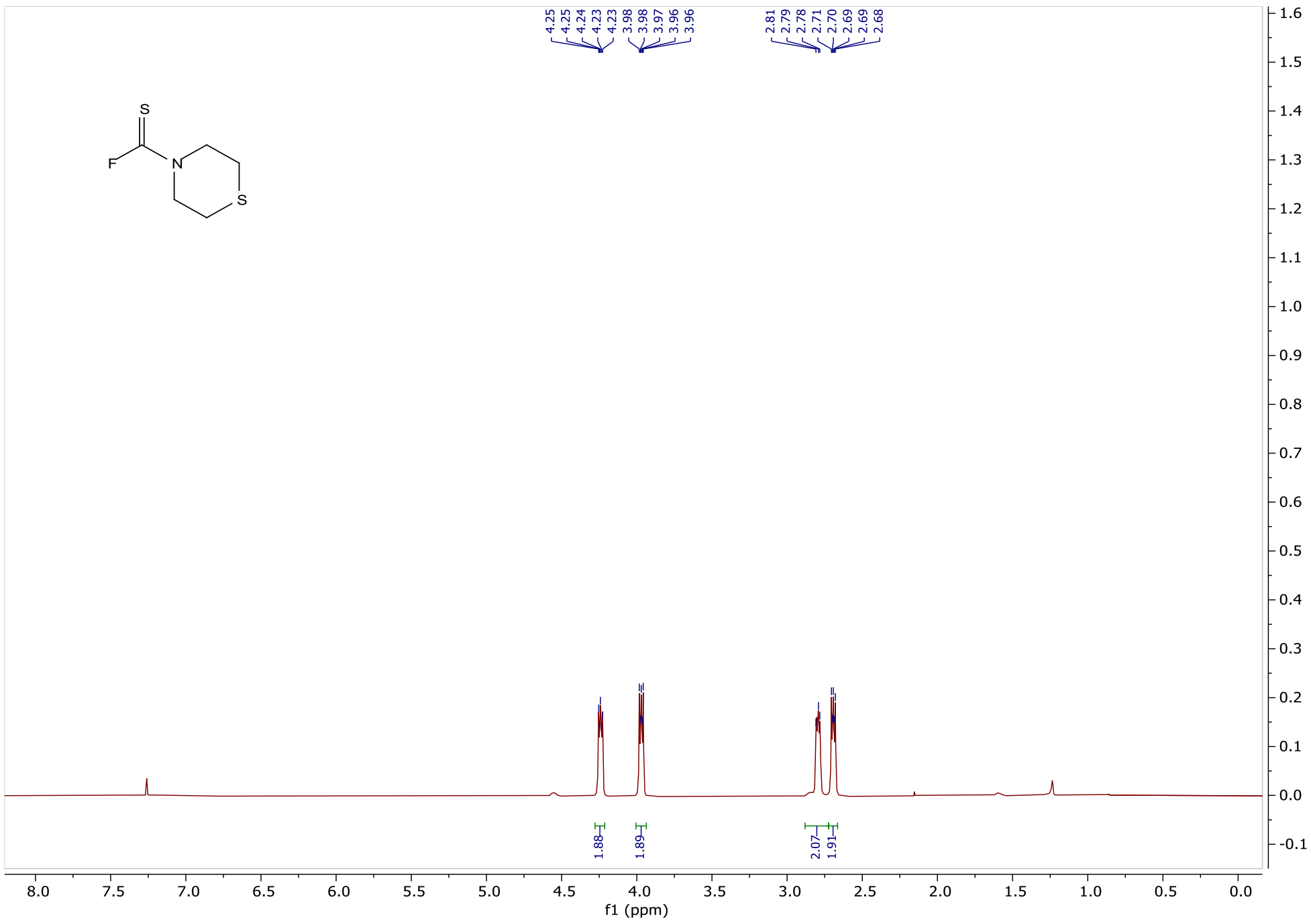


$^{1}H$ NMR (400 MHz, $CDCl_3$) of **2c**.

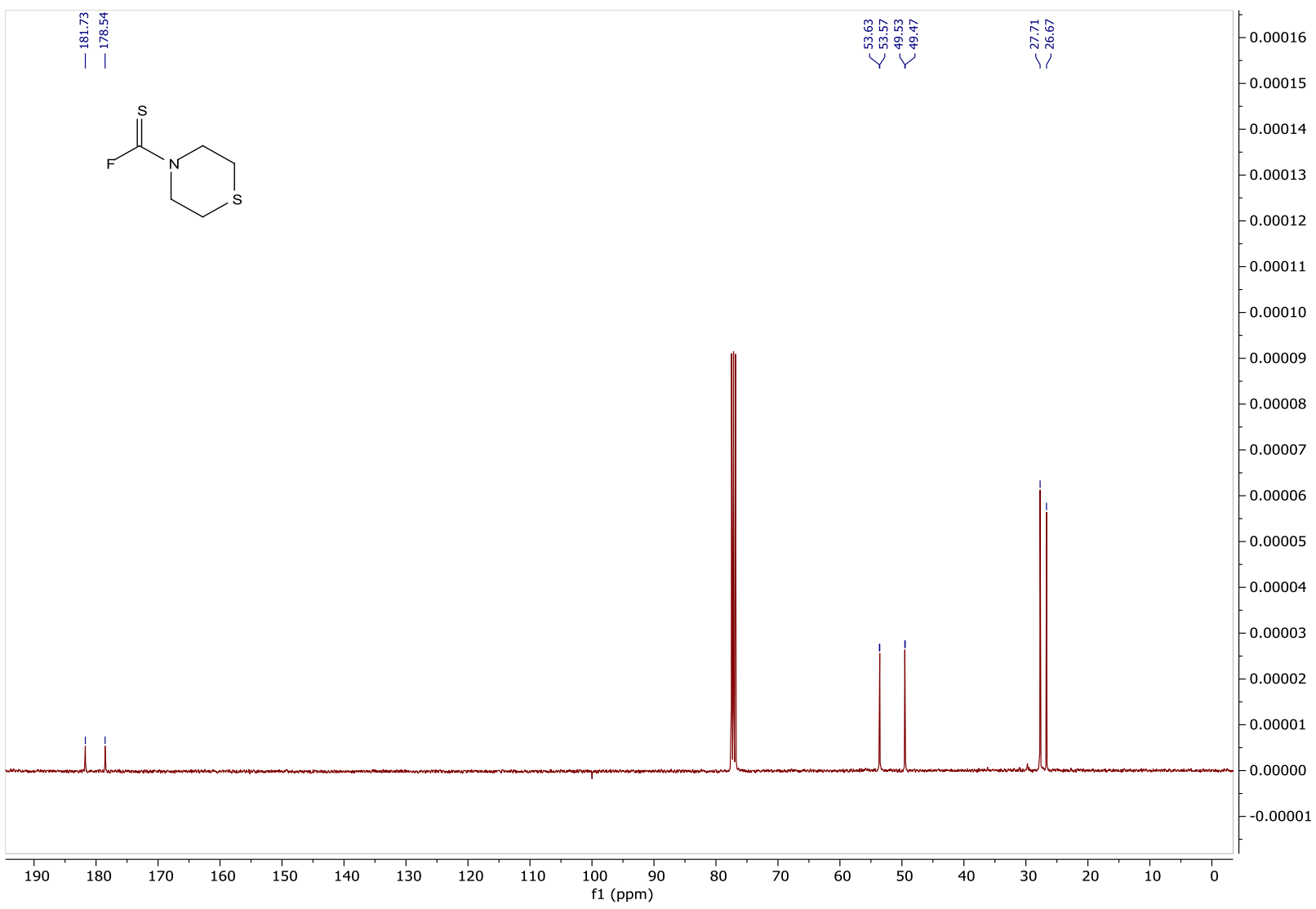


$^{13}C$ NMR (101 MHz, $CDCl_3$) of **2c.**

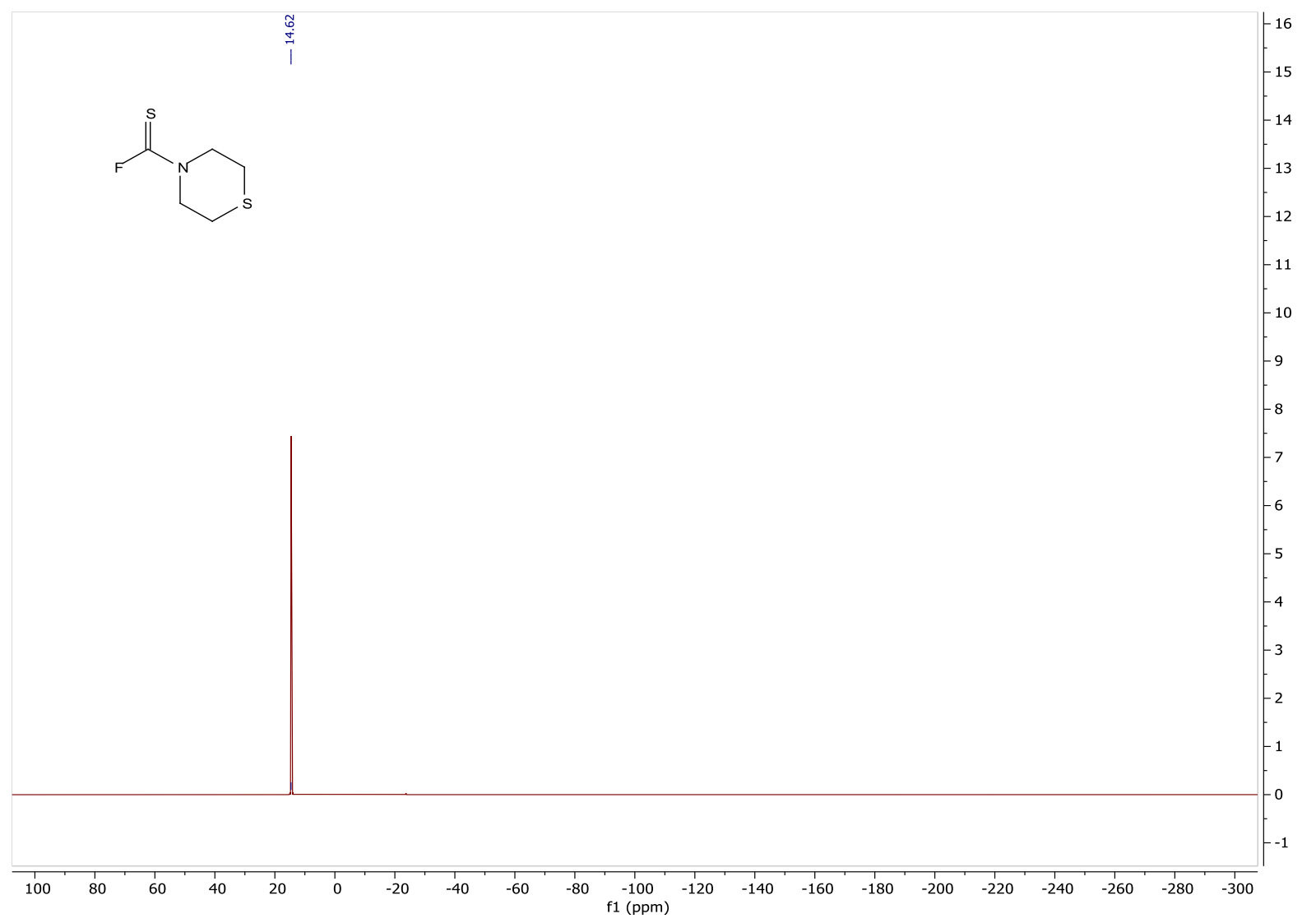

$^{19}F$ NMR (376 MHz, $CDCl_3$) of **2c**.

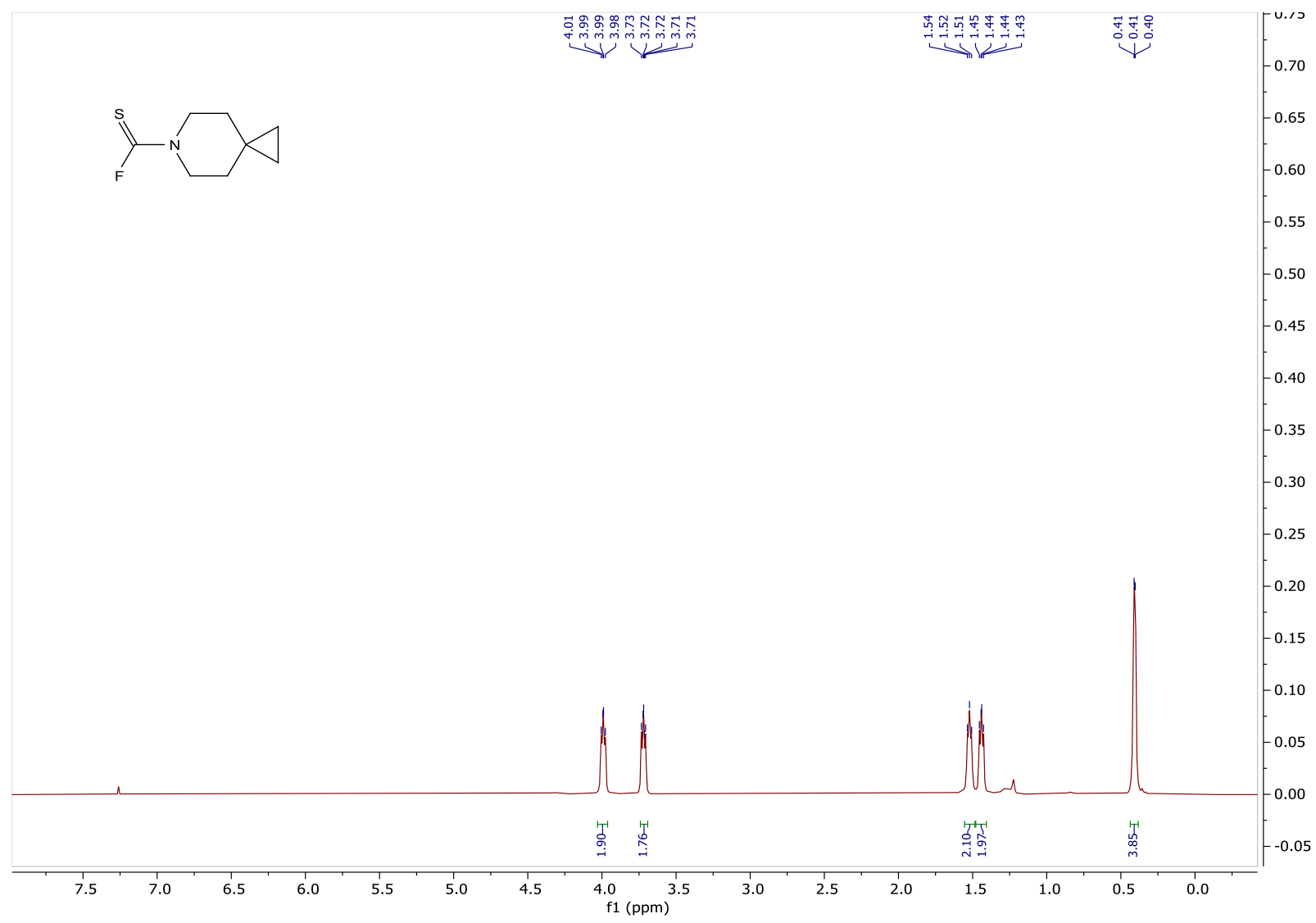

$^{1}H$ NMR (400 MHz, $CDCl_3$) of **2d**.

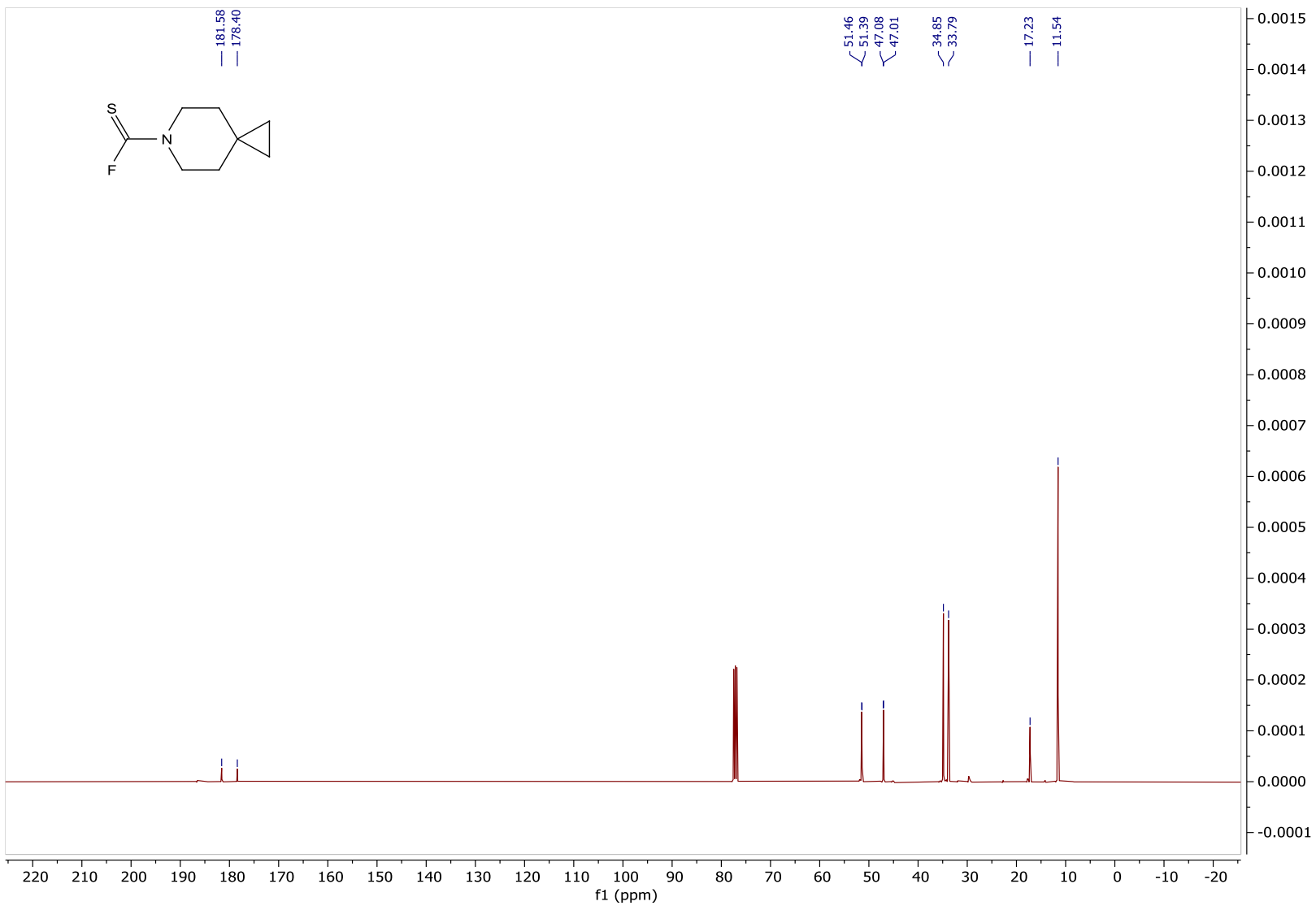


$^{13}C$ NMR (101 MHz, $CDCl_3$) of **2d**.

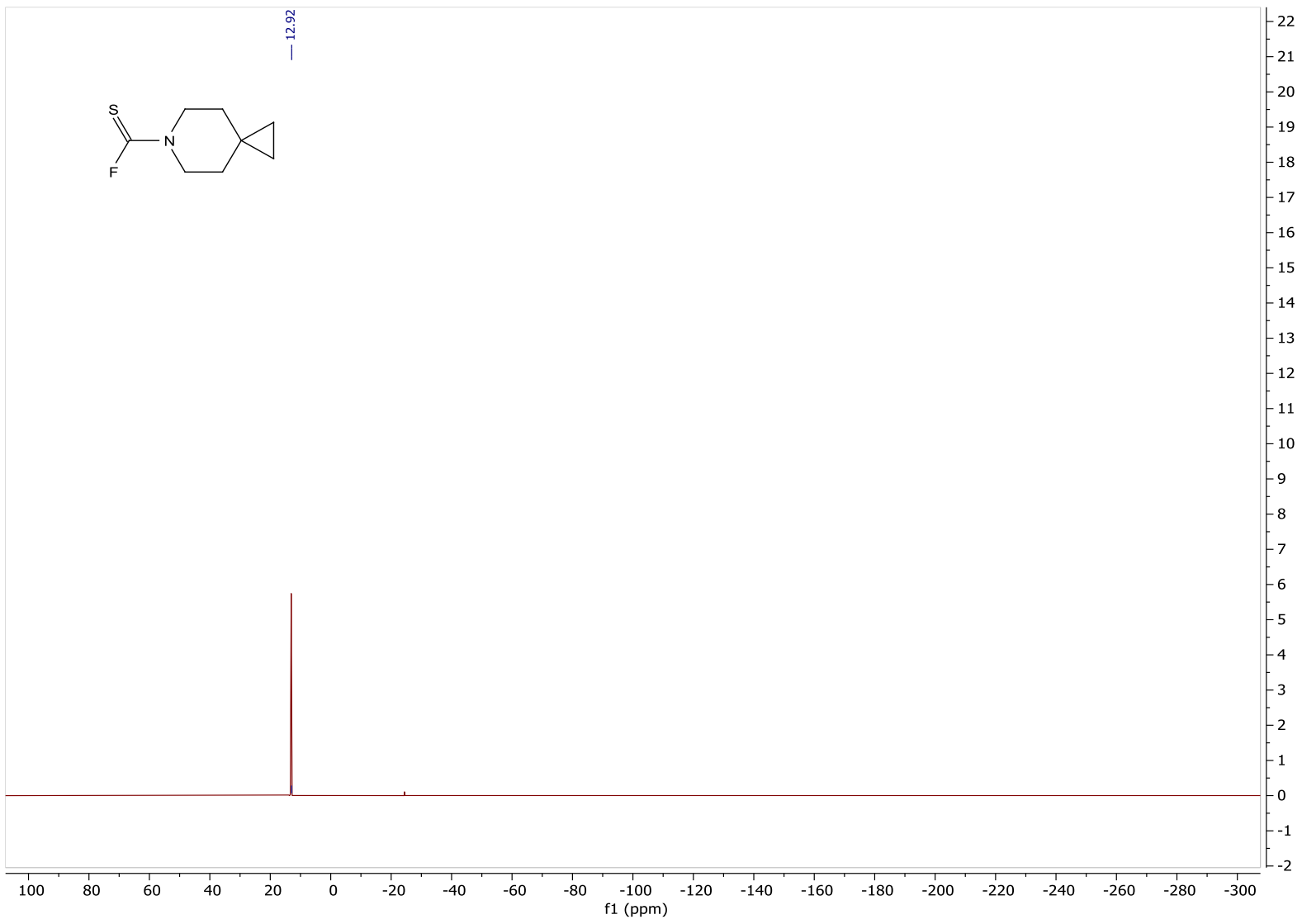


$^{19}F$ NMR (376 MHz, $CDCl_3$) of **2d**.

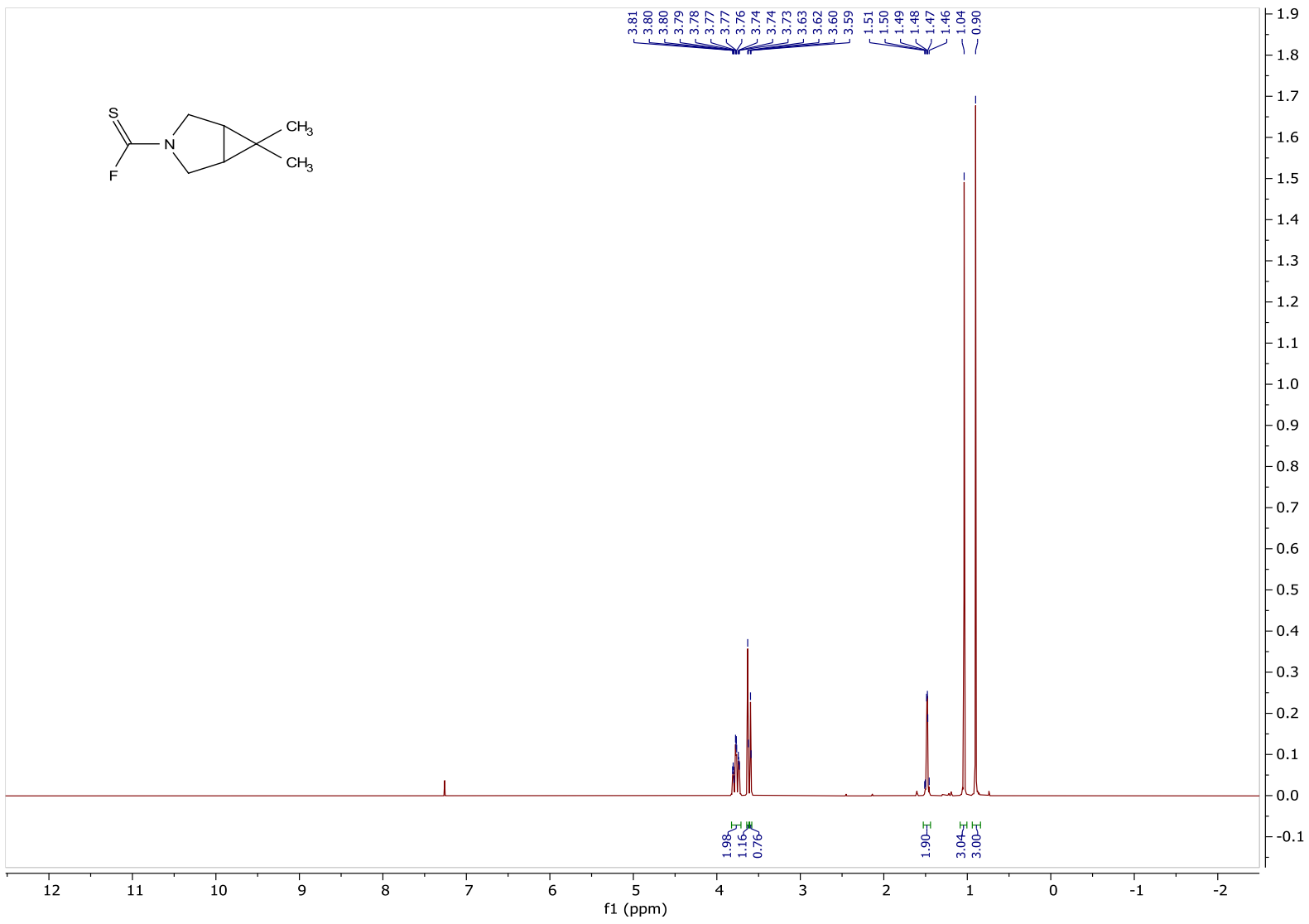

$^{1}$H NMR (400 MHz, $CDCl_3$) of **2e.**

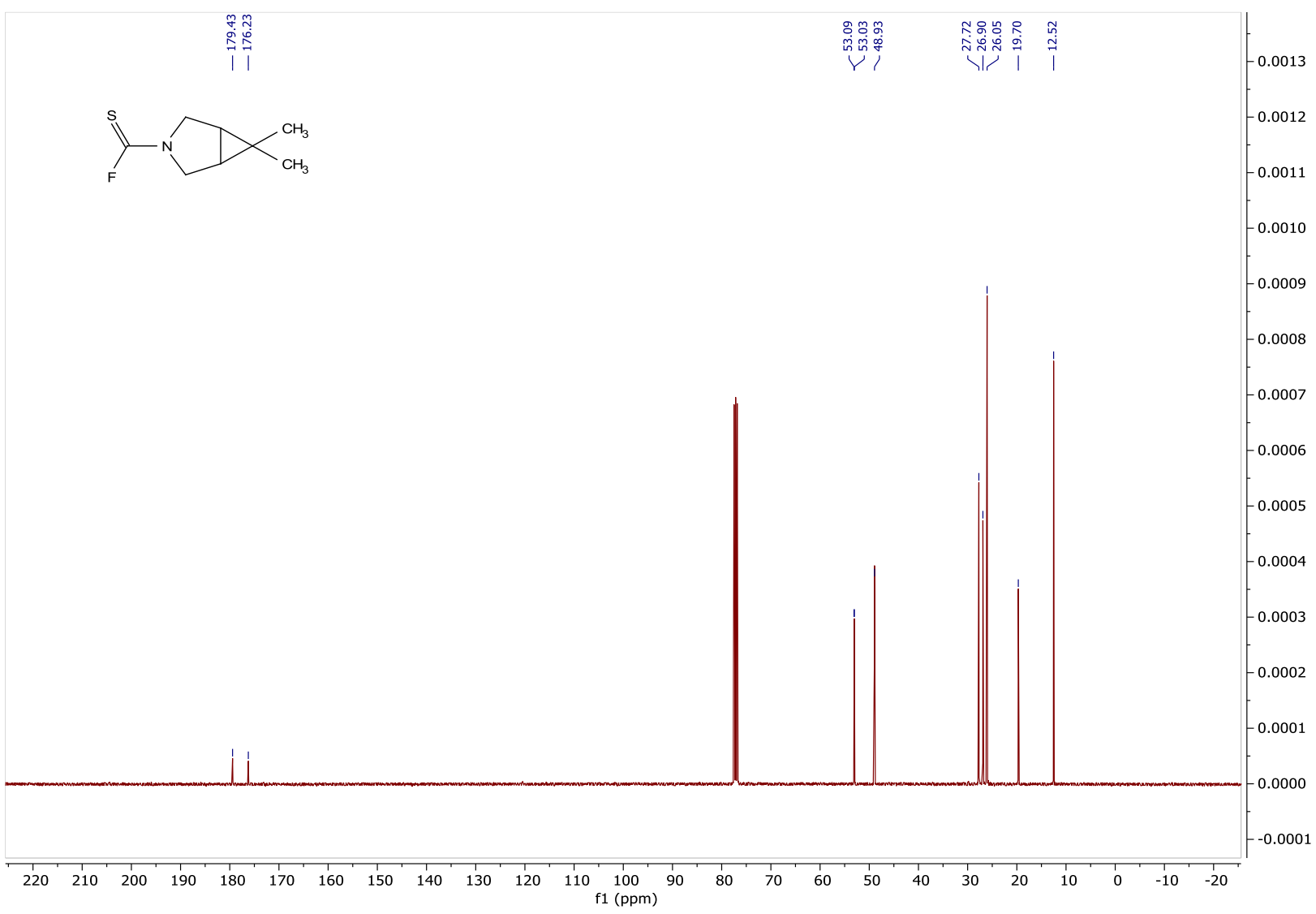

$^{13}$C NMR (101 MHz, $CDCl_3$) of **2e.**

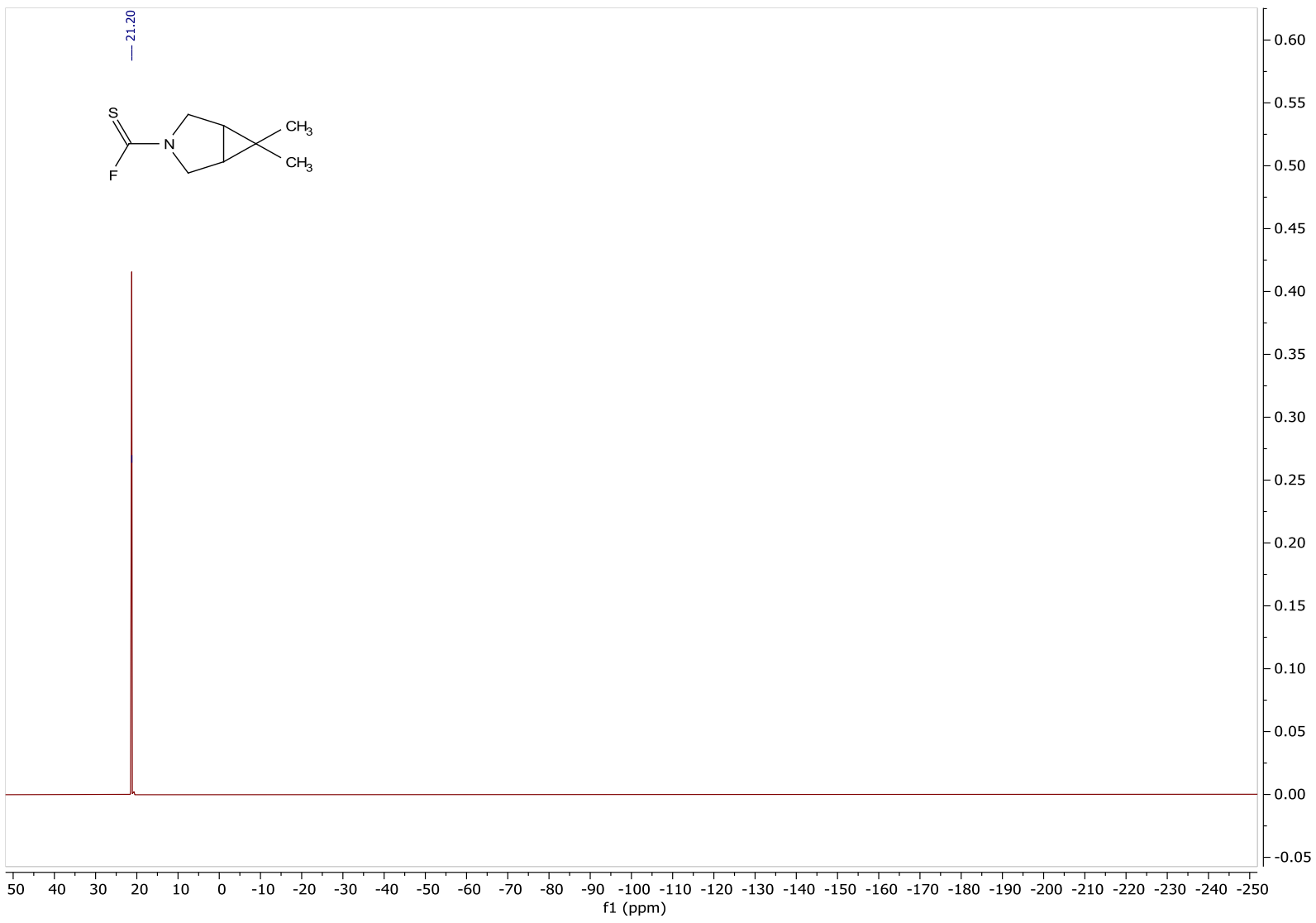

$^{19}F$ NMR (376 MHz, $CDCl_3$) of **2e**.

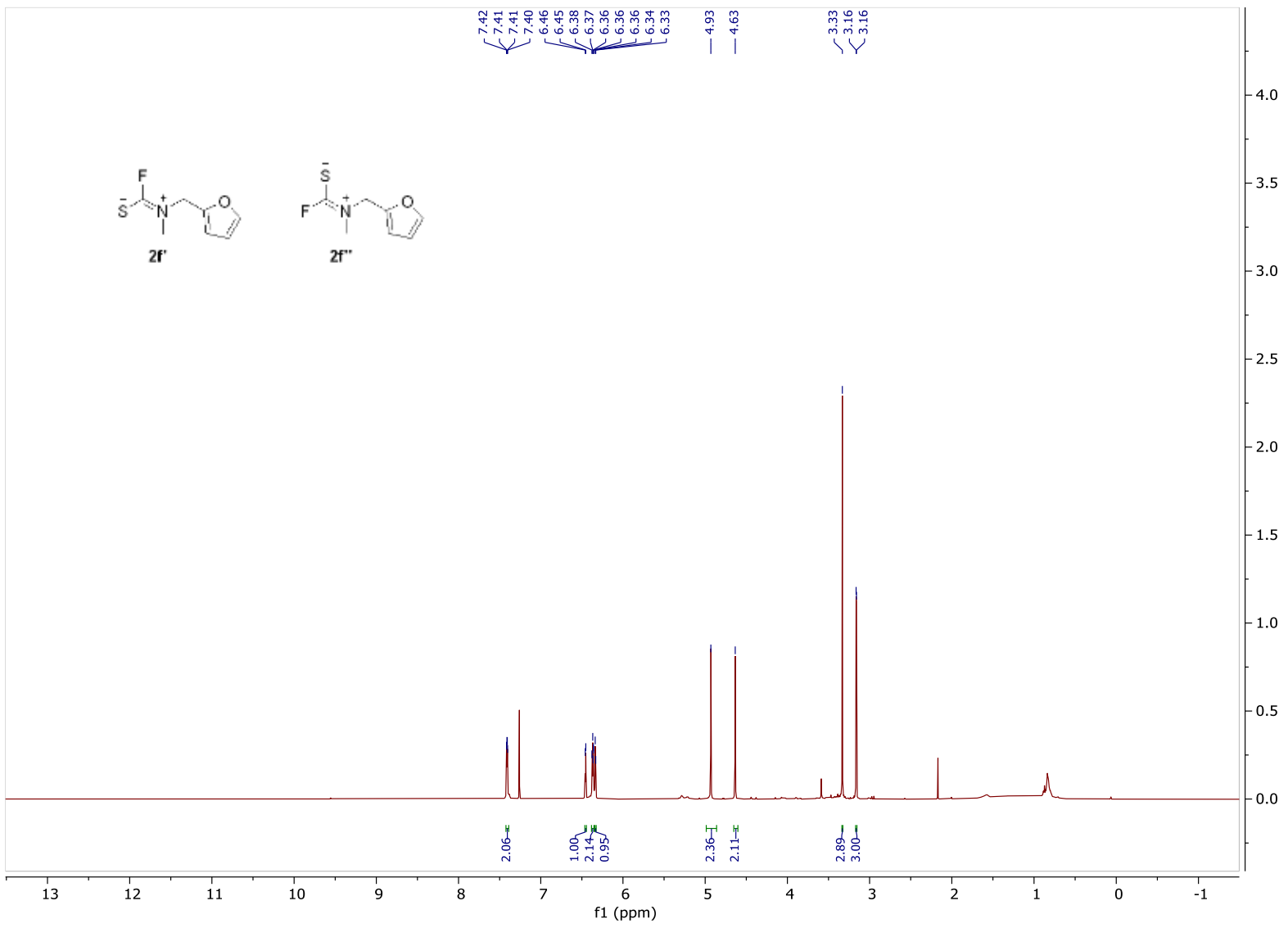

$^{1}H$ NMR (500 MHz, $CDCl_3$) of **2f’** and **2f’’.**

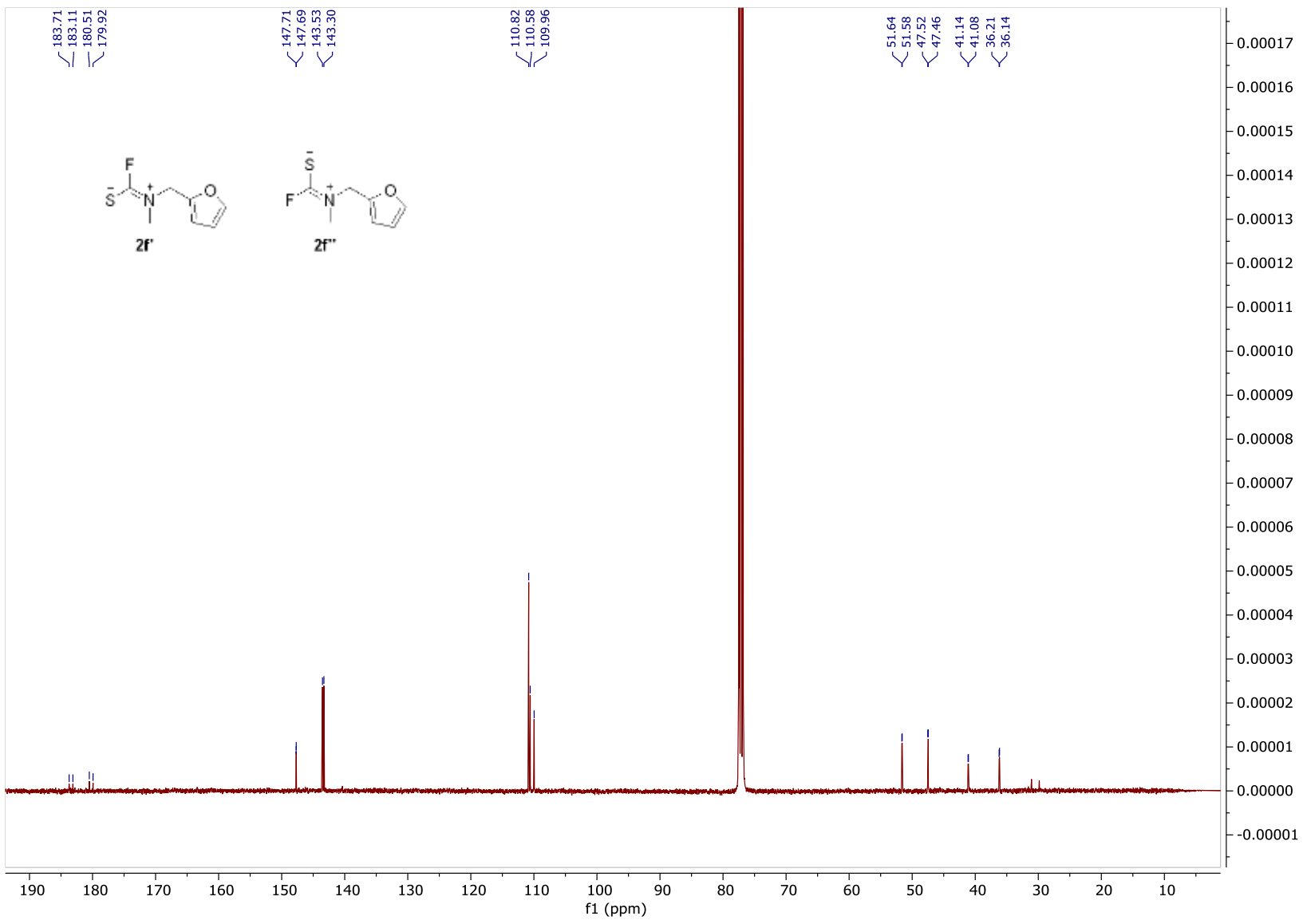

$^{13}C$ NMR (101 MHz, $CDCl_3$) of **2f’** and **2f’’.**

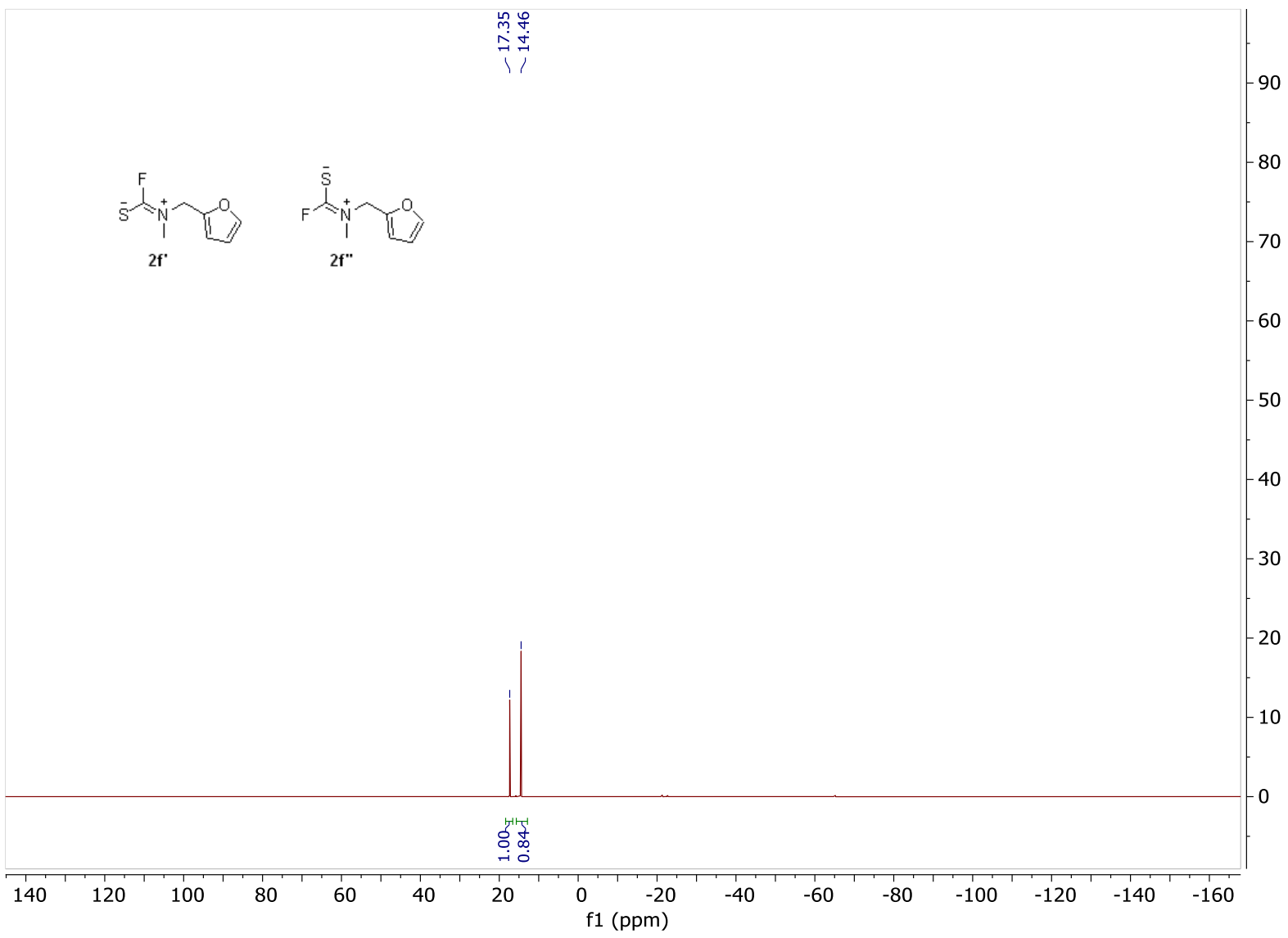

$^{19}F$ NMR (471 MHz, $CDCl_3$) of **2f’** and **2f’’**.

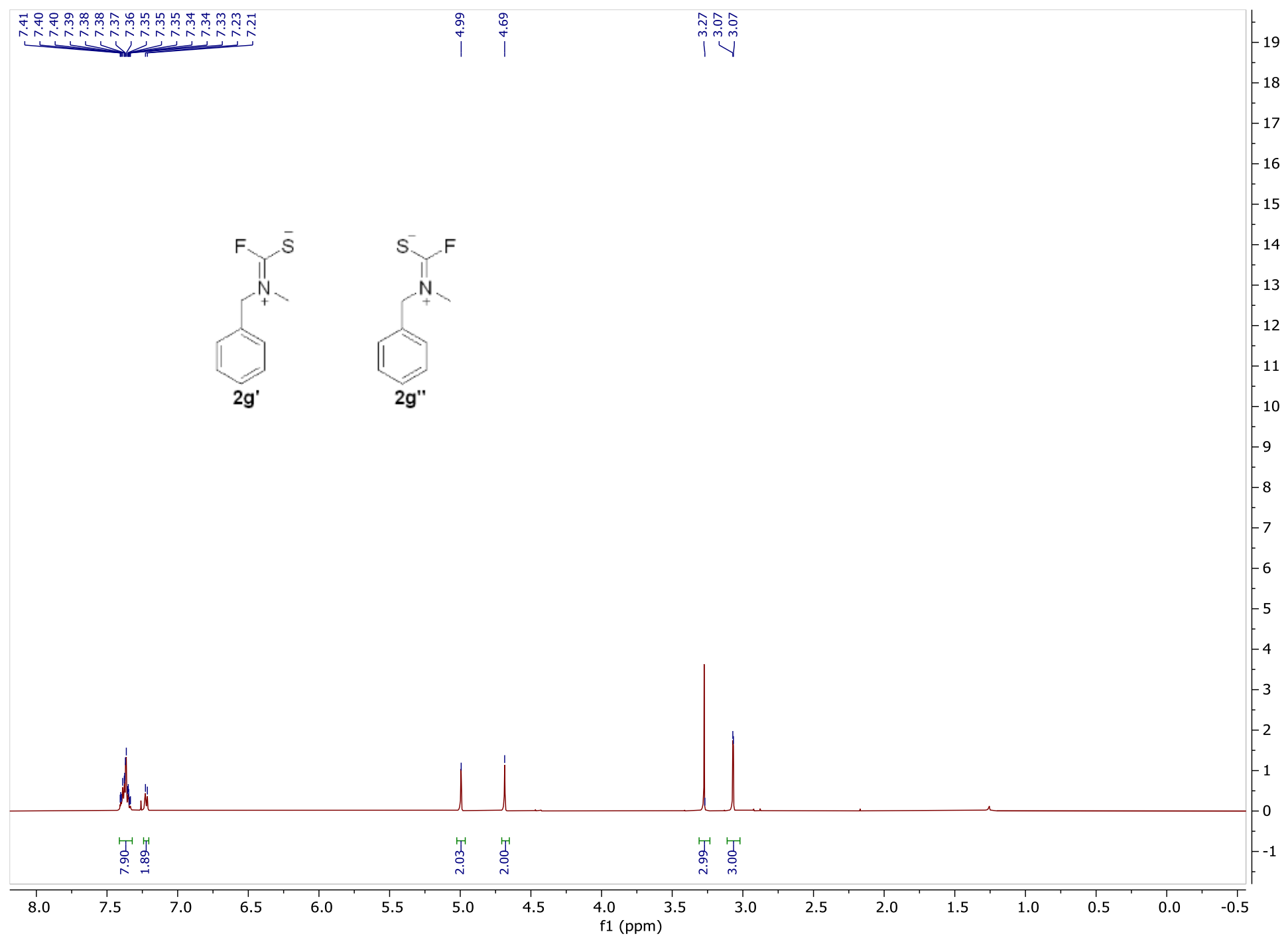

$^{1}H$ NMR (500 MHz, $CDCl_3$) of **2g'** and **2g''**.

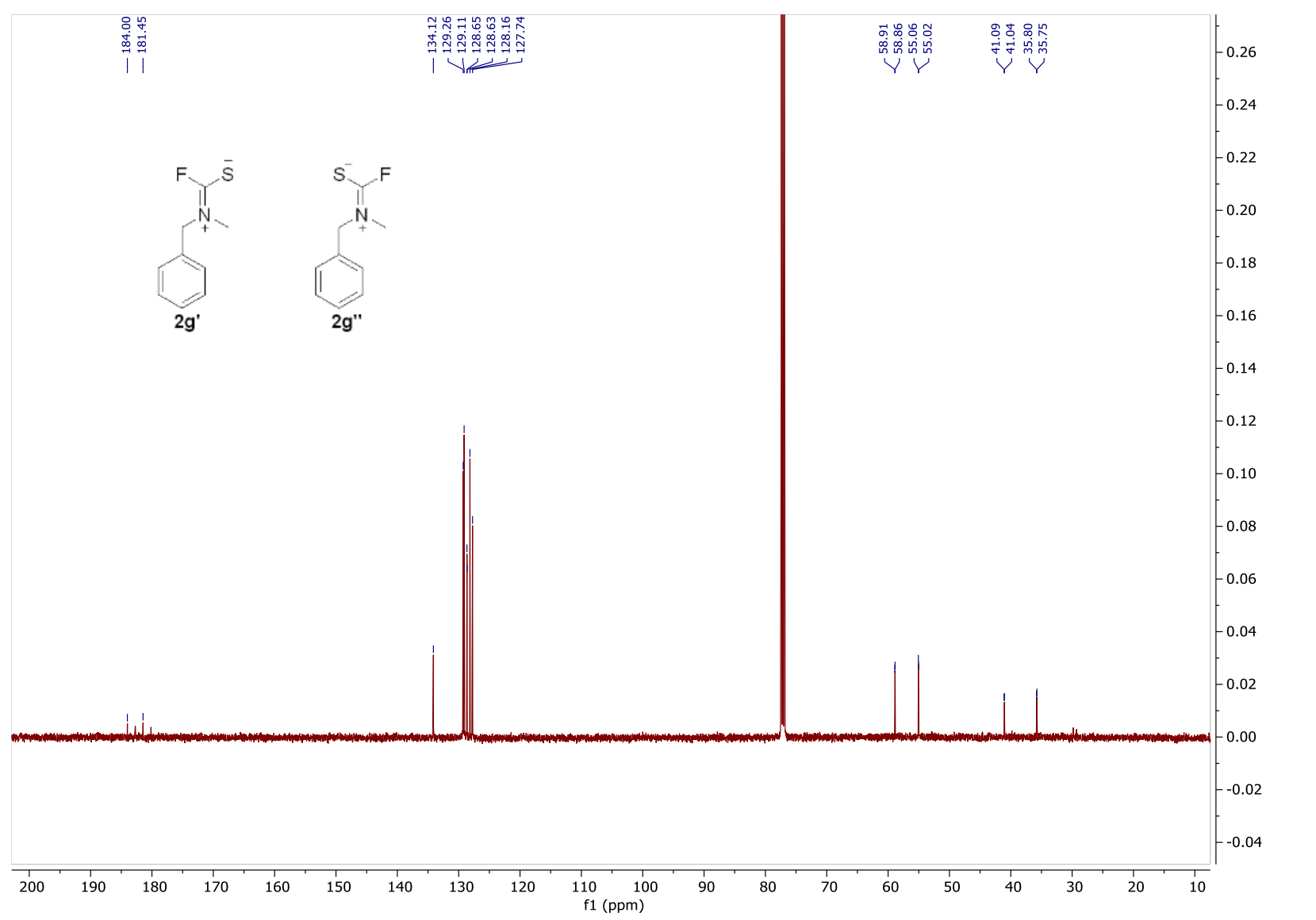

$^{13}C$ NMR (126 MHz, $CDCl_3$) of **2g'** and **2g''**.

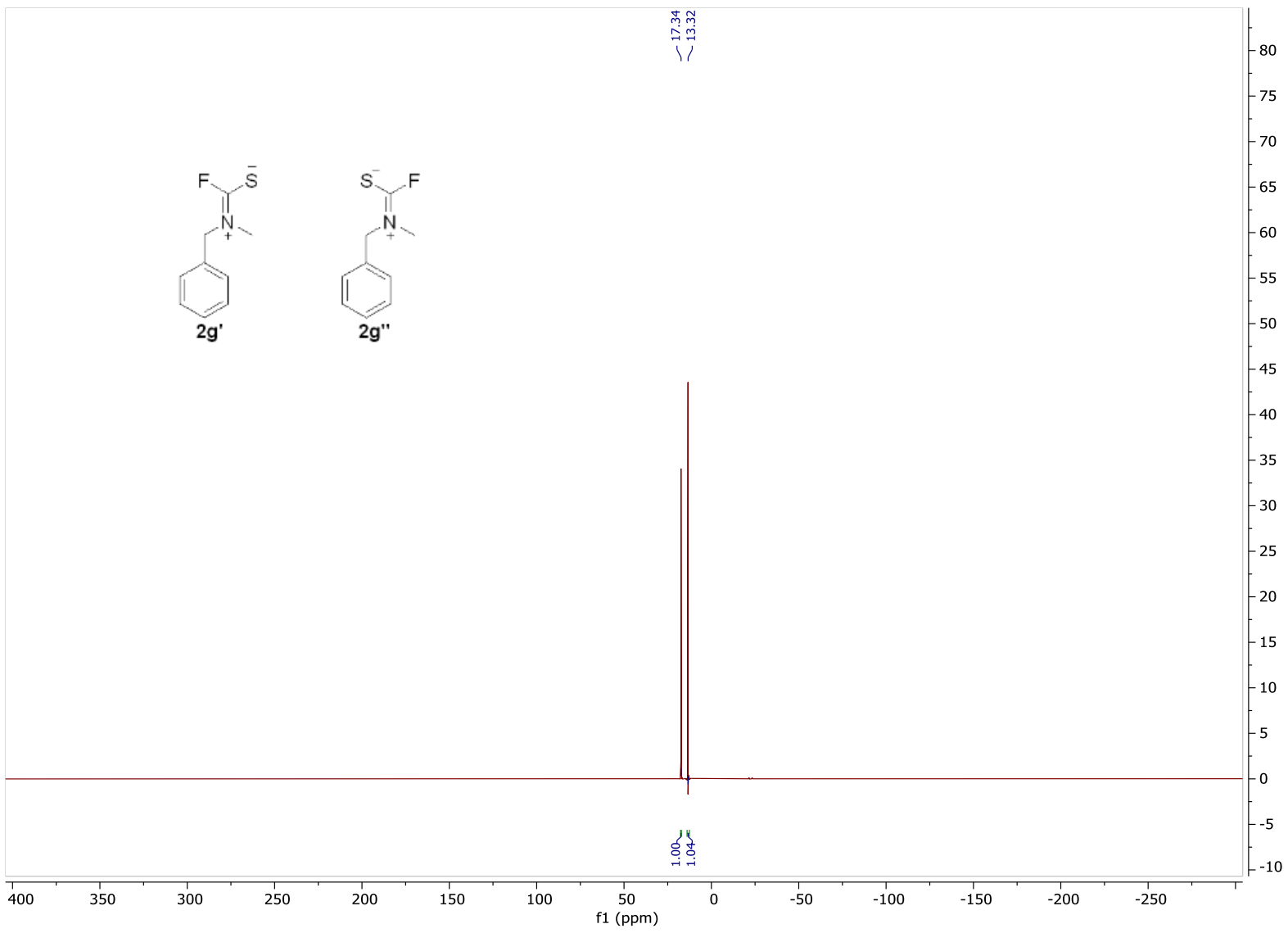


$^{19}$F NMR (471 MHz, $CDCl_3$) of **2g'** and **2g''**.

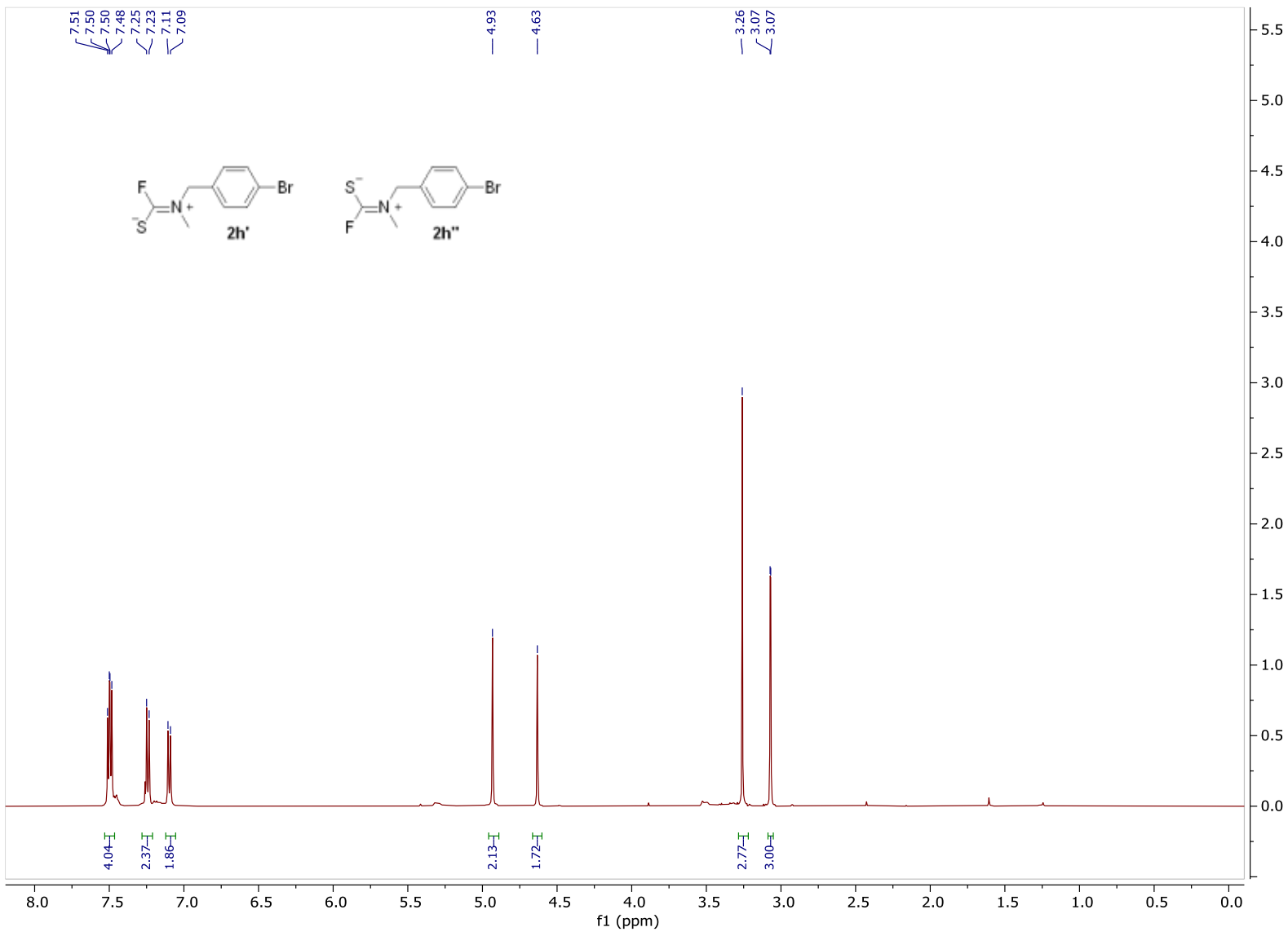


$^{1}$H NMR (500 MHz, $CDCl_3$) of **2h'** and **2h''**.

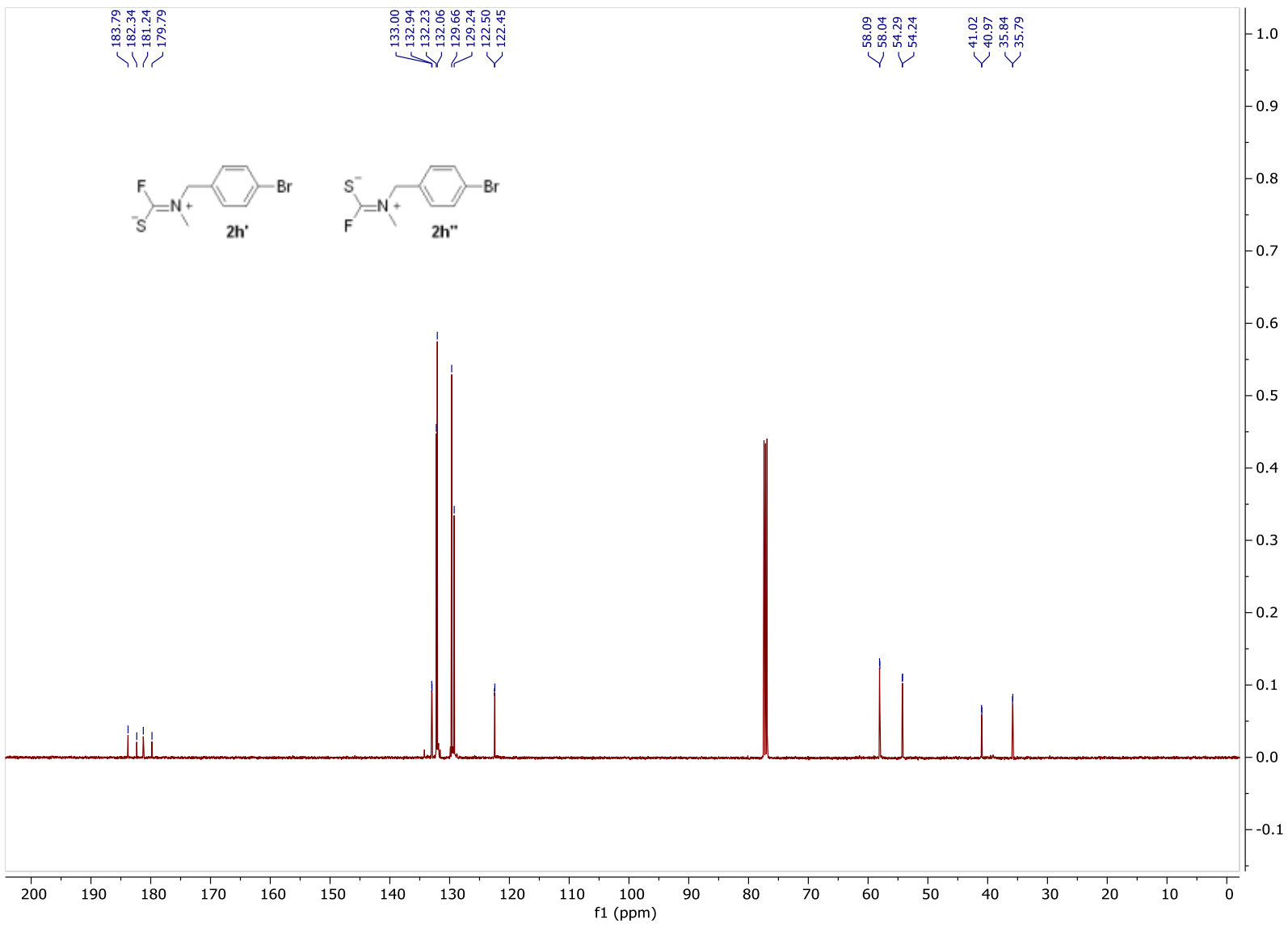

$^{13}C$ NMR (126 MHz, $CDCl_3$) of **2h’** and **2h”**.

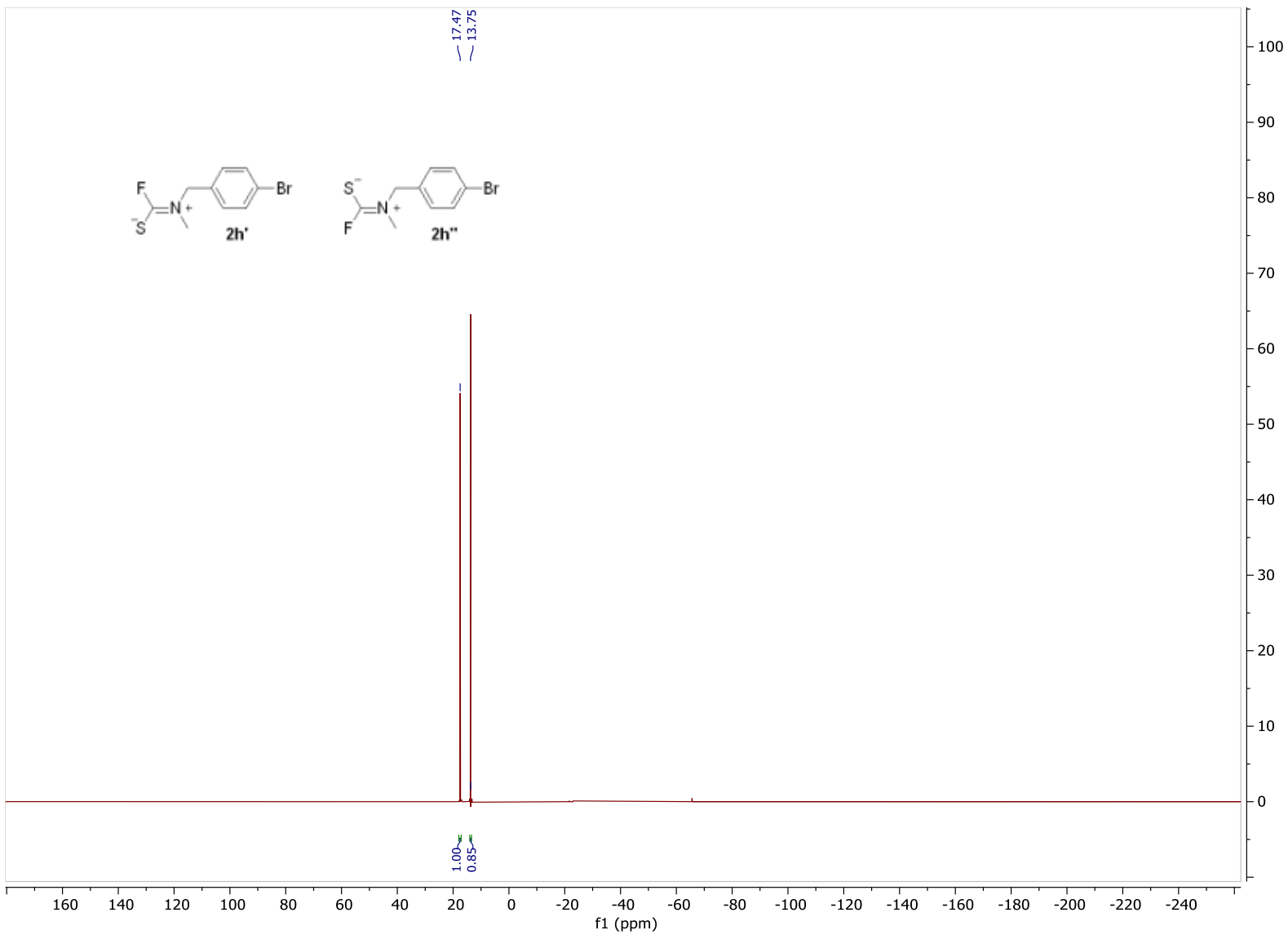

$^{19}F$ NMR (471 MHz, $CDCl_3$) of **2h’** and **2h”**.

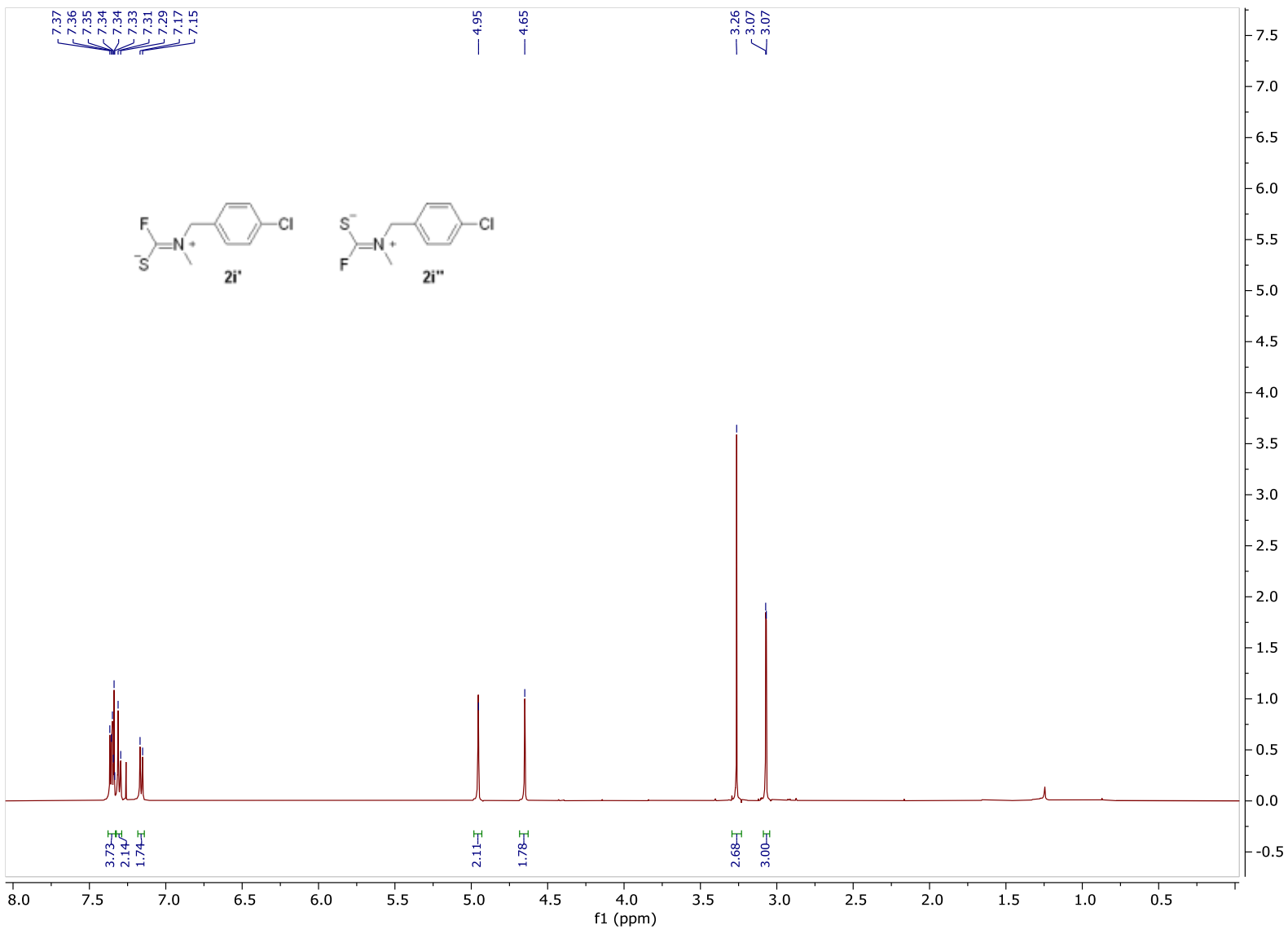

$^{1}H$ NMR (500 MHz, $CDCl_3$) of **2i'** and **2i''**.

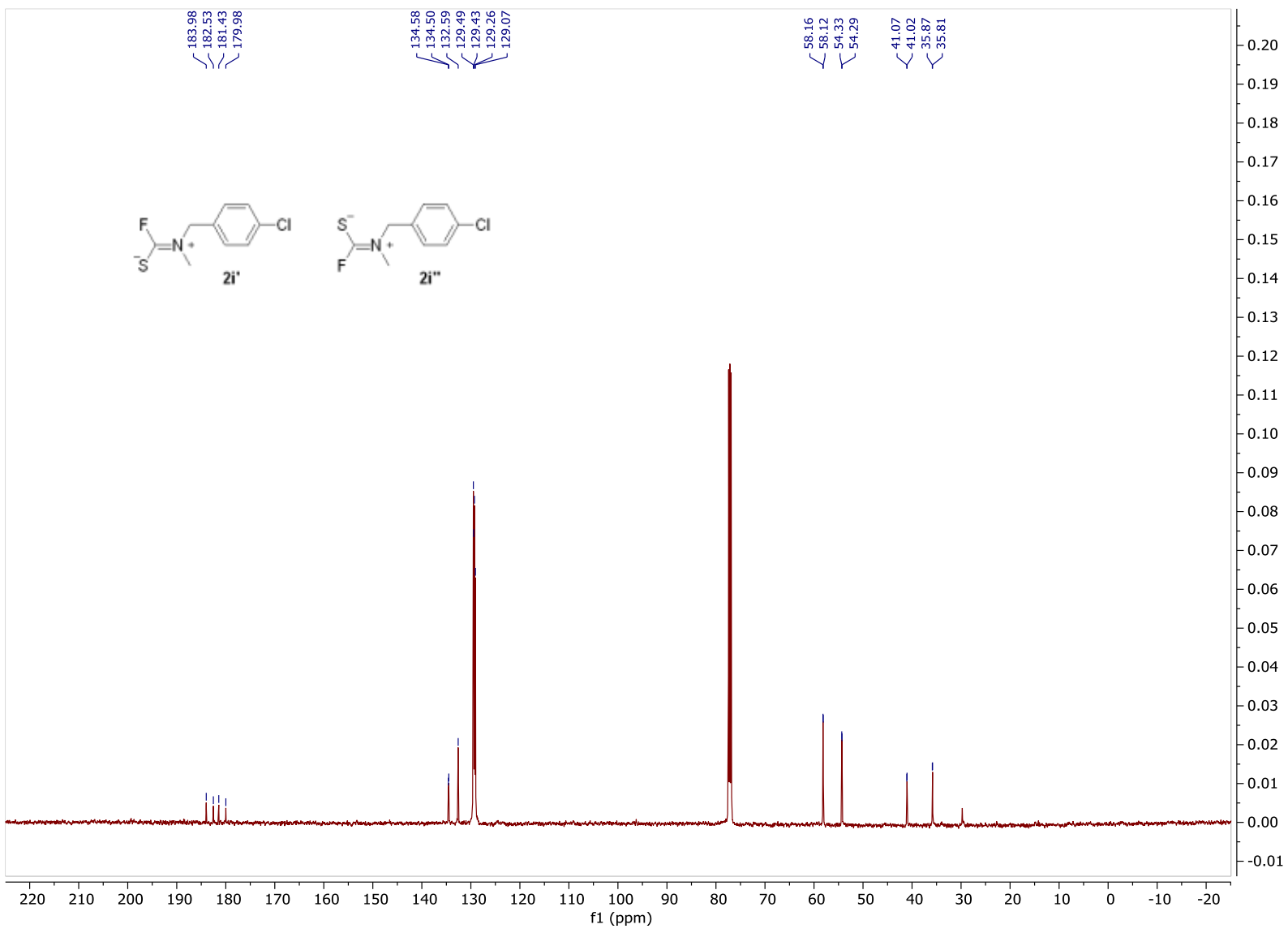

$^{13}C$ NMR (126 MHz, $CDCl_3$) of **2i'** and **2i''**.

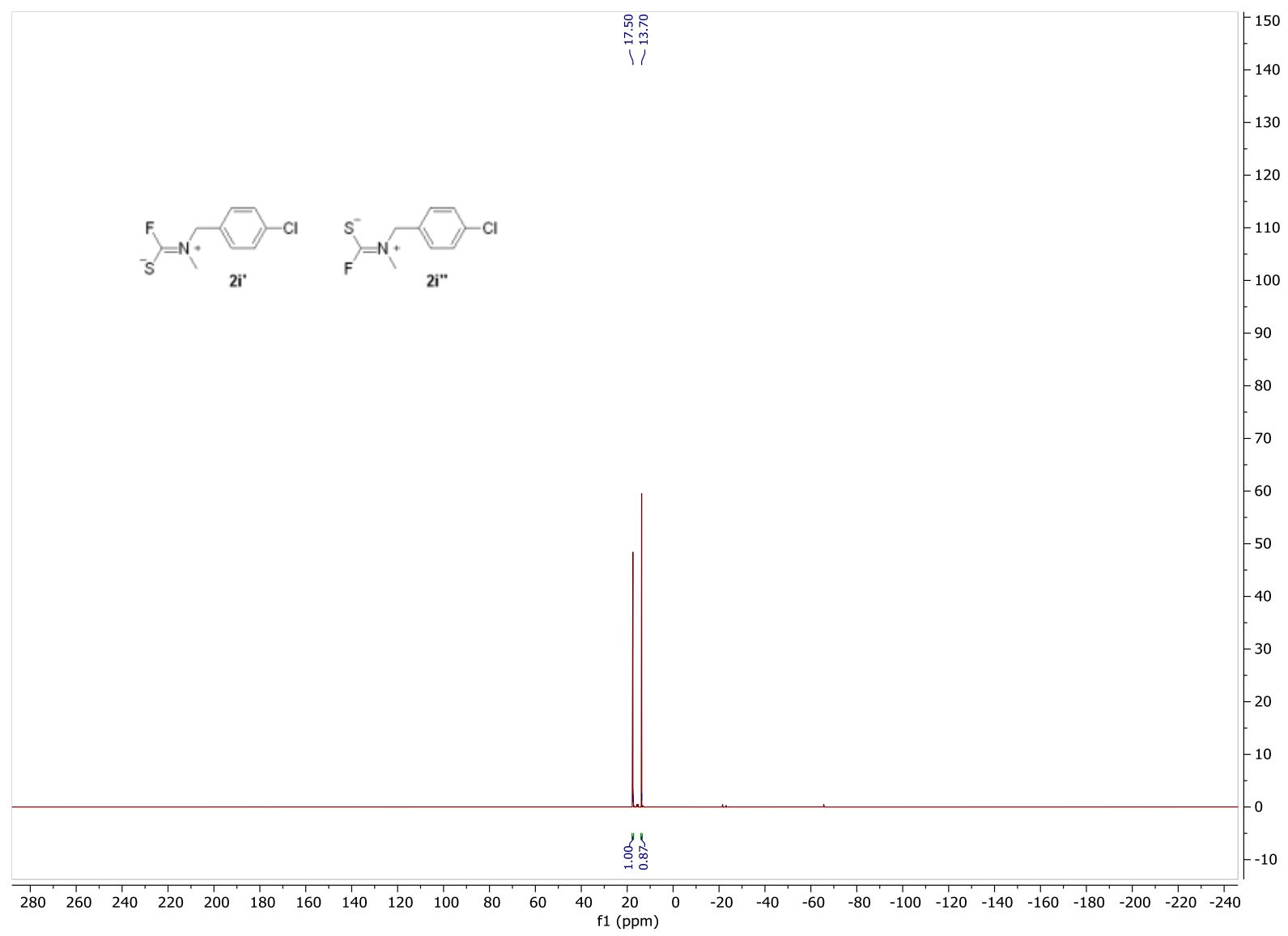

$^{19}$F NMR (471 MHz, $CDCl_3$) of **2i’** and **2i”**.

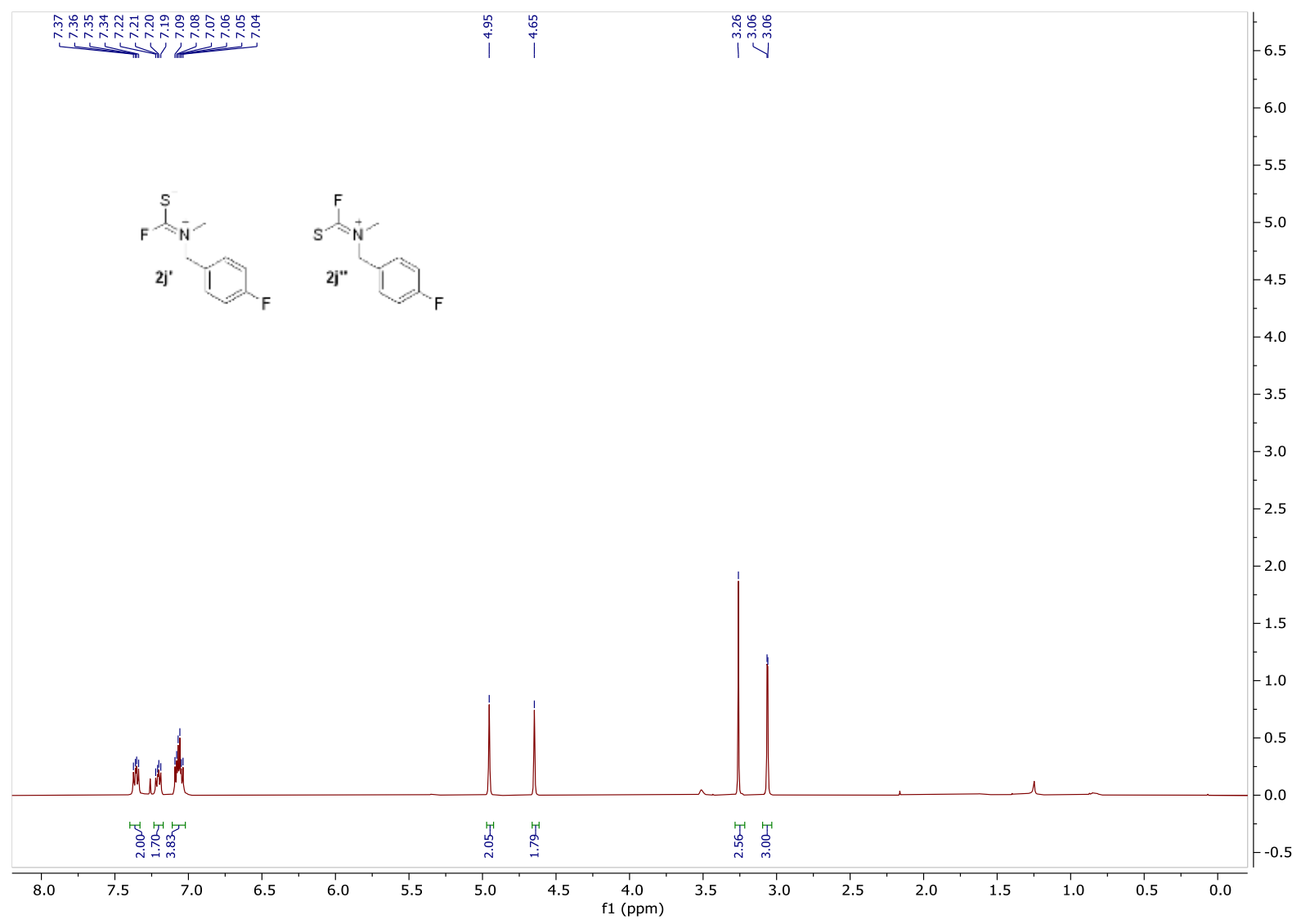

$^{1}$H NMR (400 MHz, $CDCl_3$) of **2j’** and **2j”**.

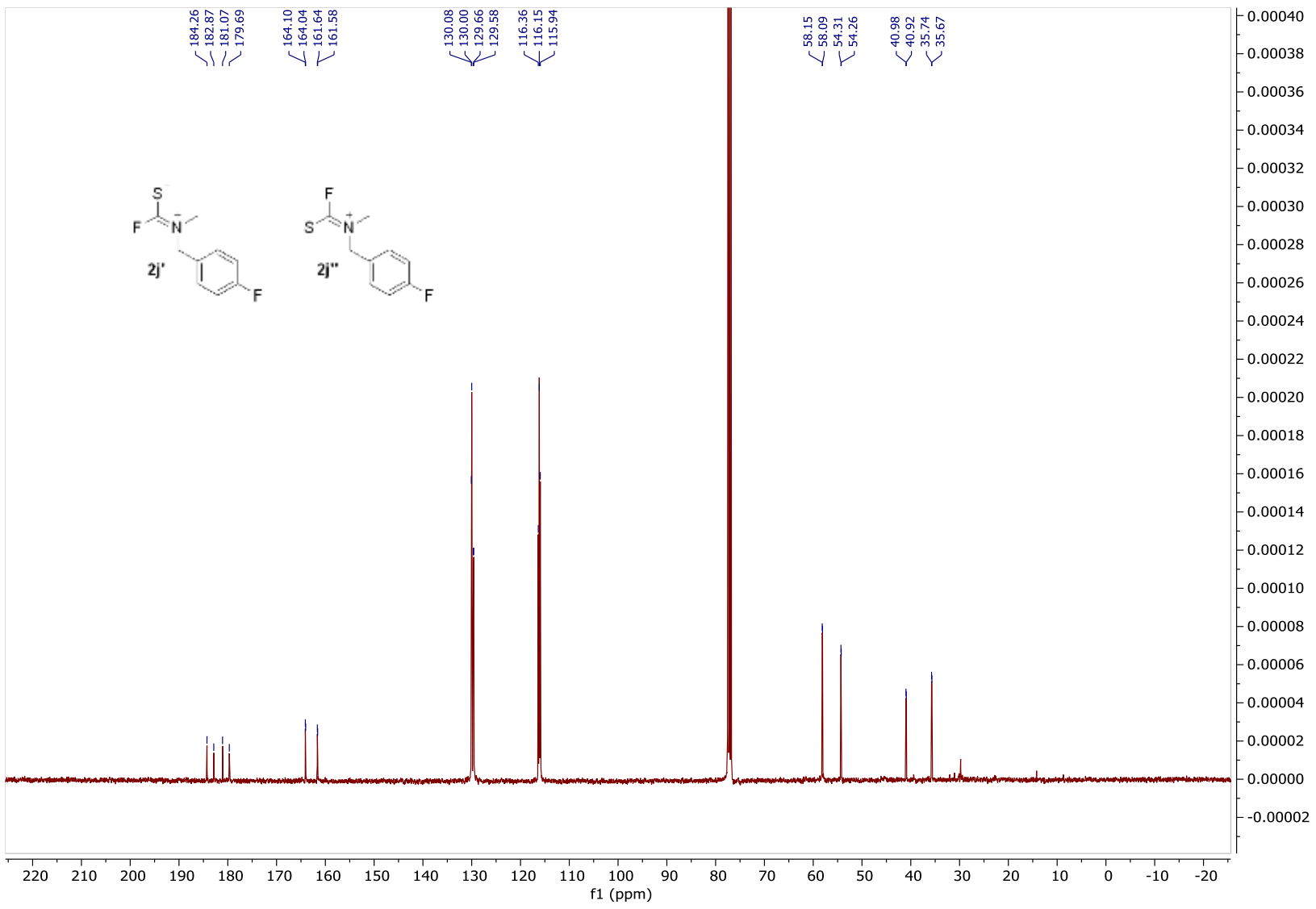


$^{13}$C NMR (101 MHz, $CDCl_3$) of **2j’** and **2j”**

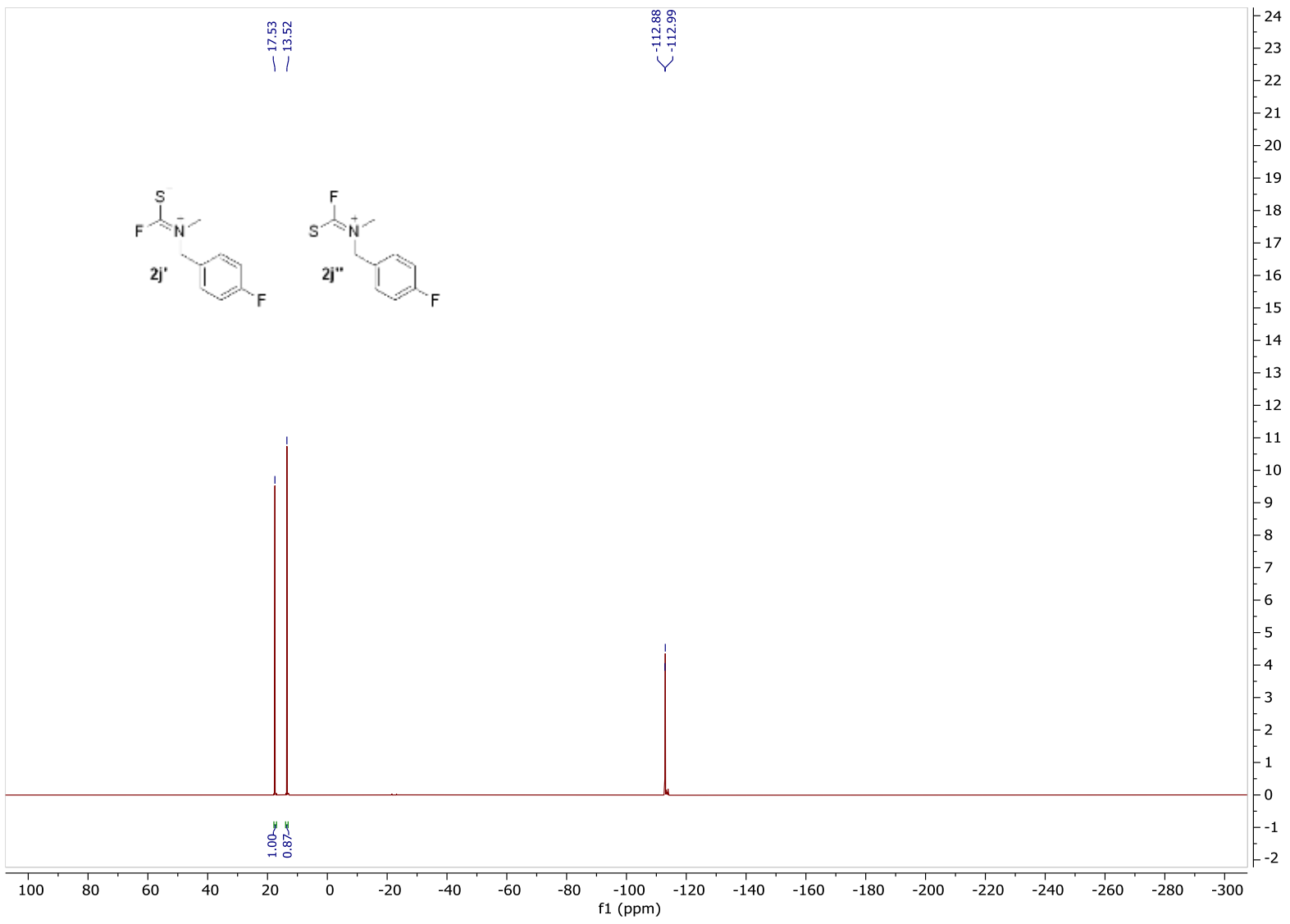


$^{19}$F NMR (376 MHz, $CDCl_3$) of **2j’** and **2j”**.

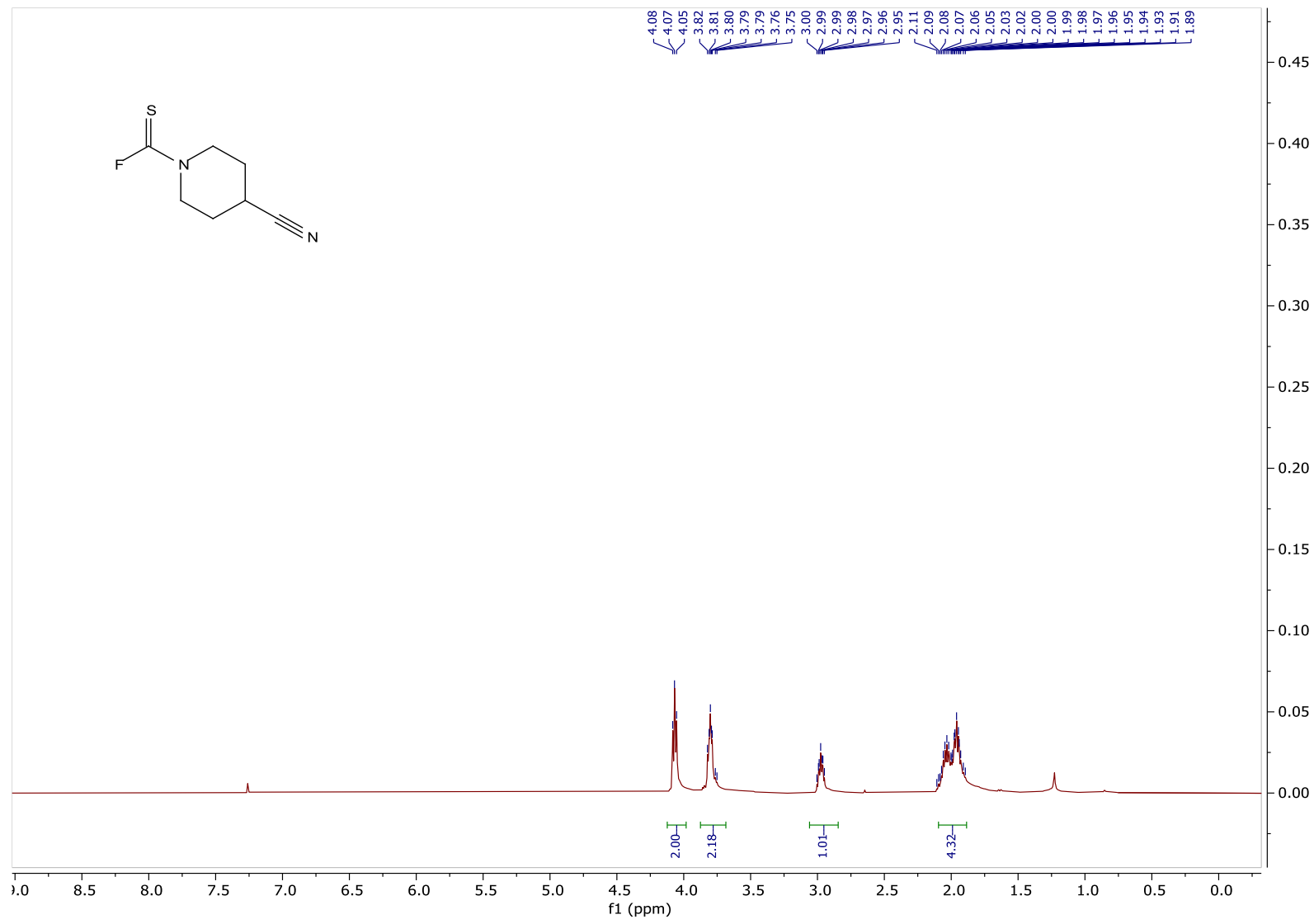

$^{1}$H NMR (400 MHz, $CDCl_3$) of **2k.**

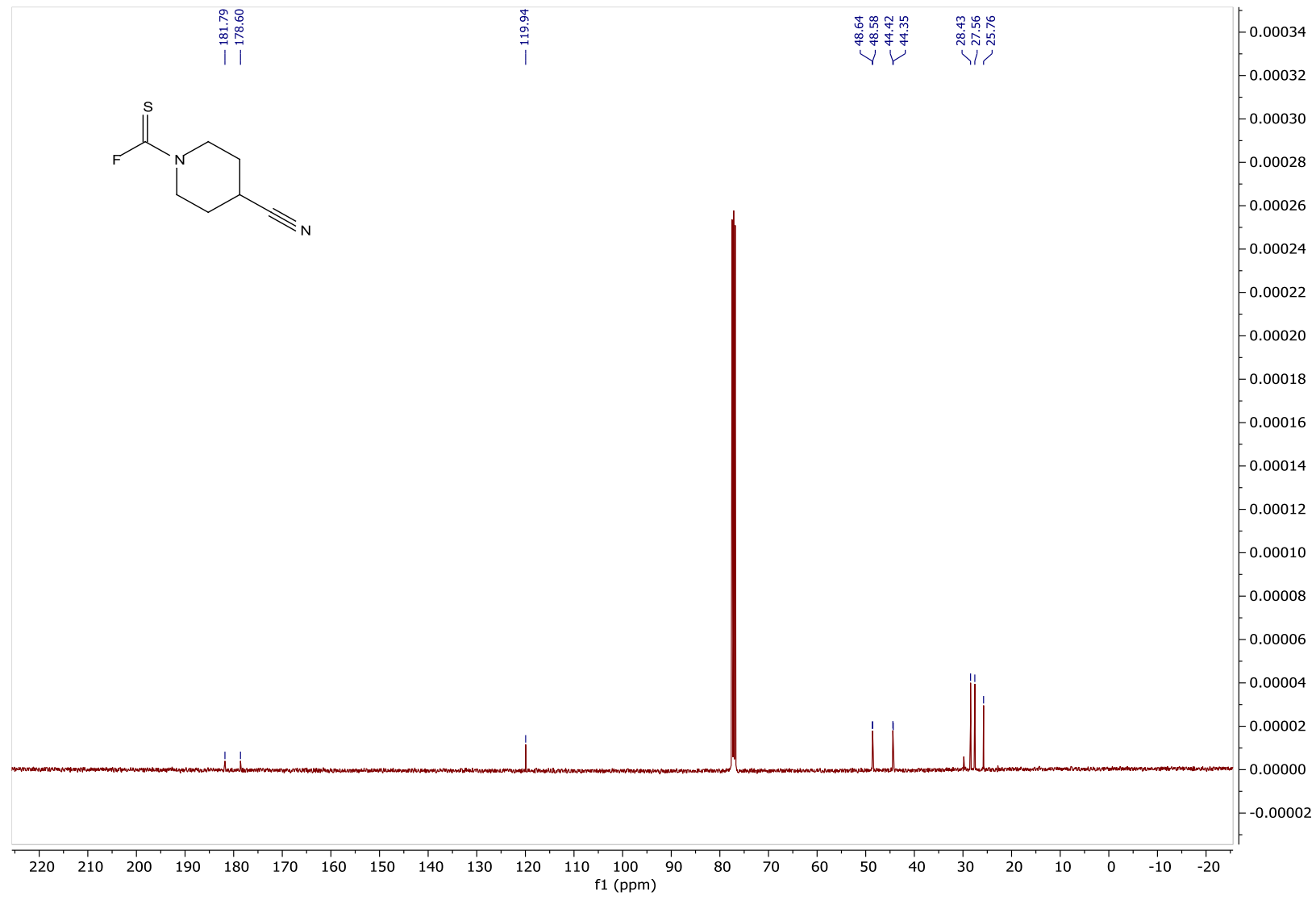

$^{13}$C NMR (101 MHz, $CDCl_3$) of **2k**.

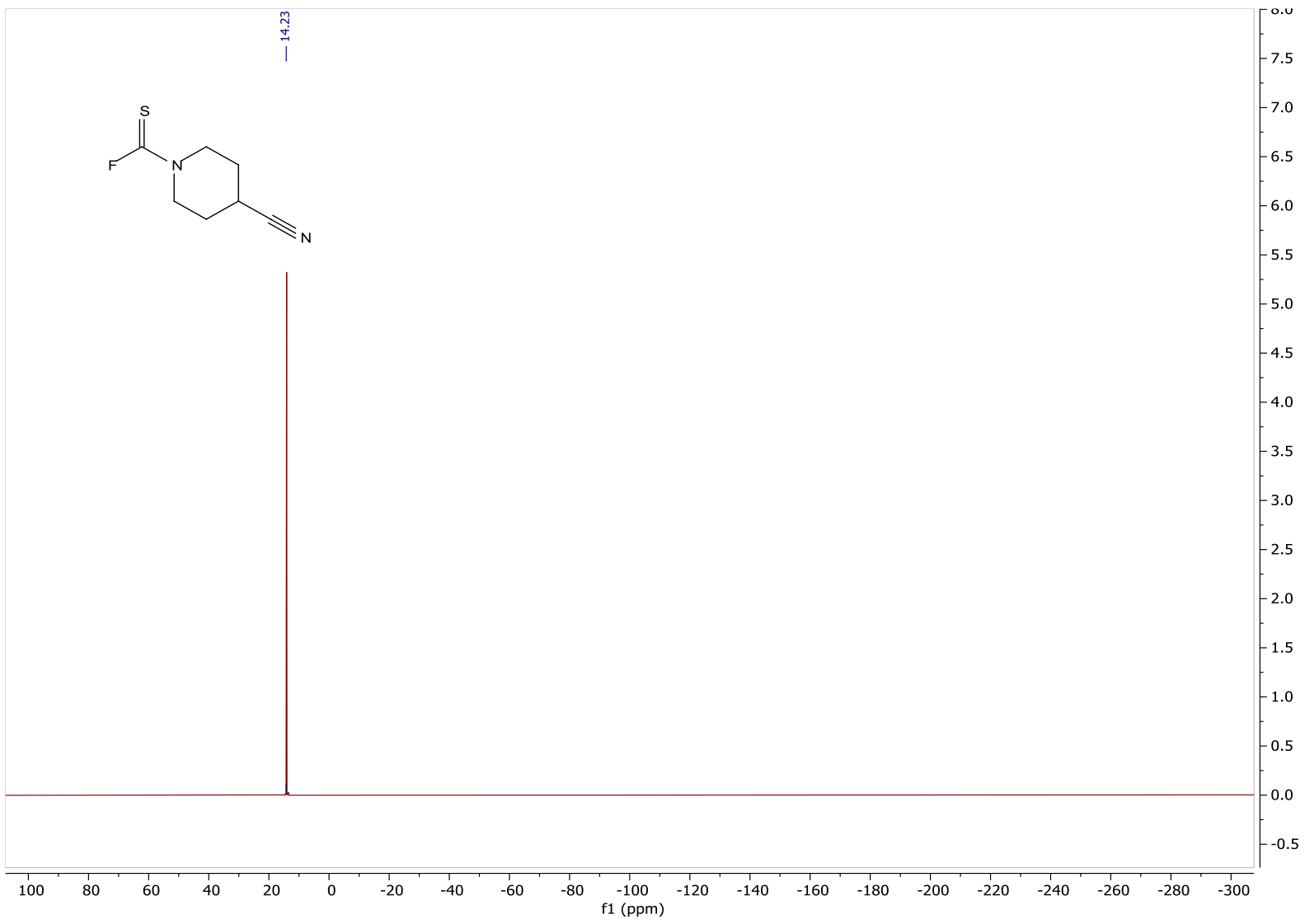

$^{19}$F NMR (376 MHz, $CDCl_3$) of **2k**.

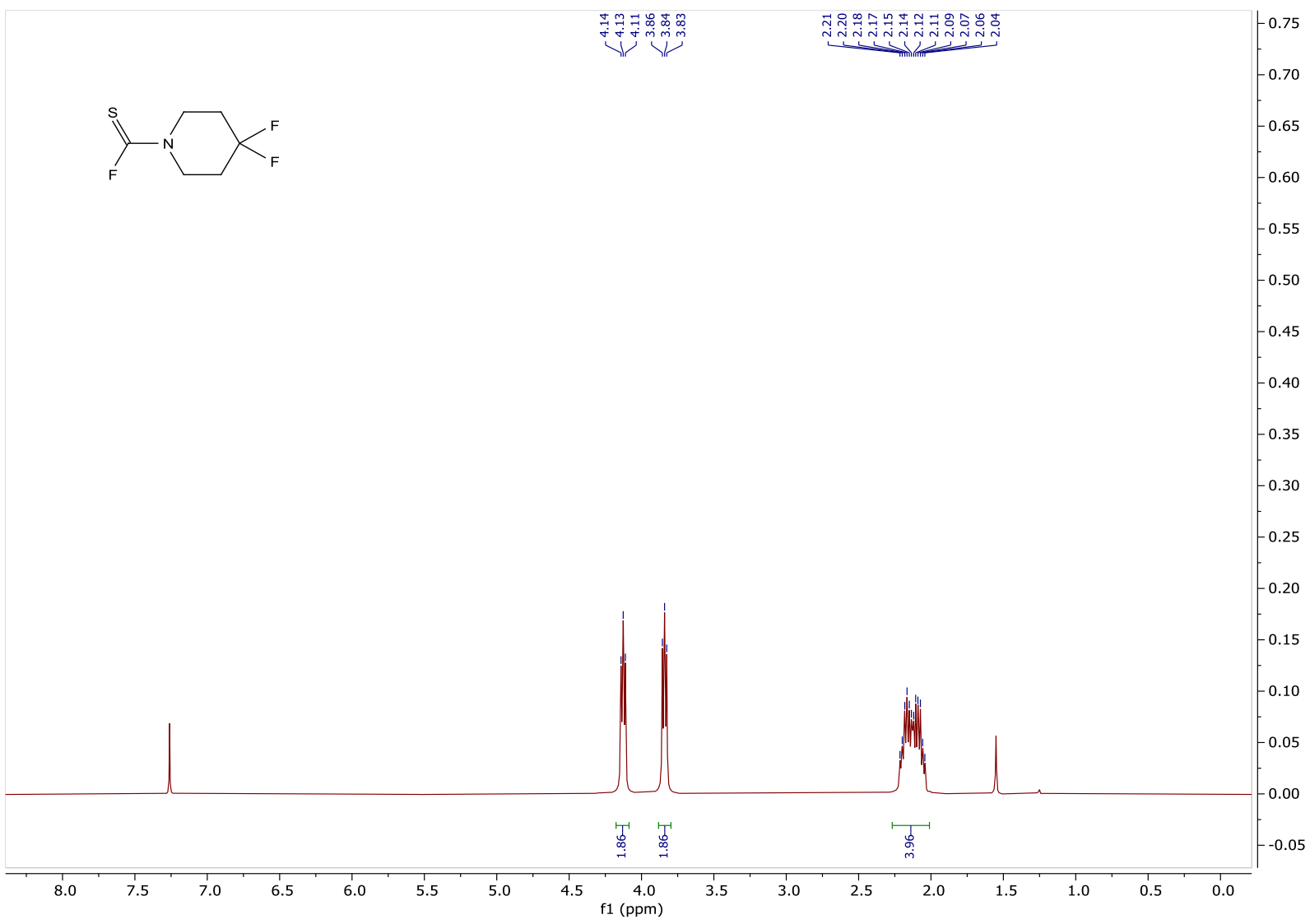

$^{1}$H NMR (400 MHz, $CDCl_3$) of **2l**.

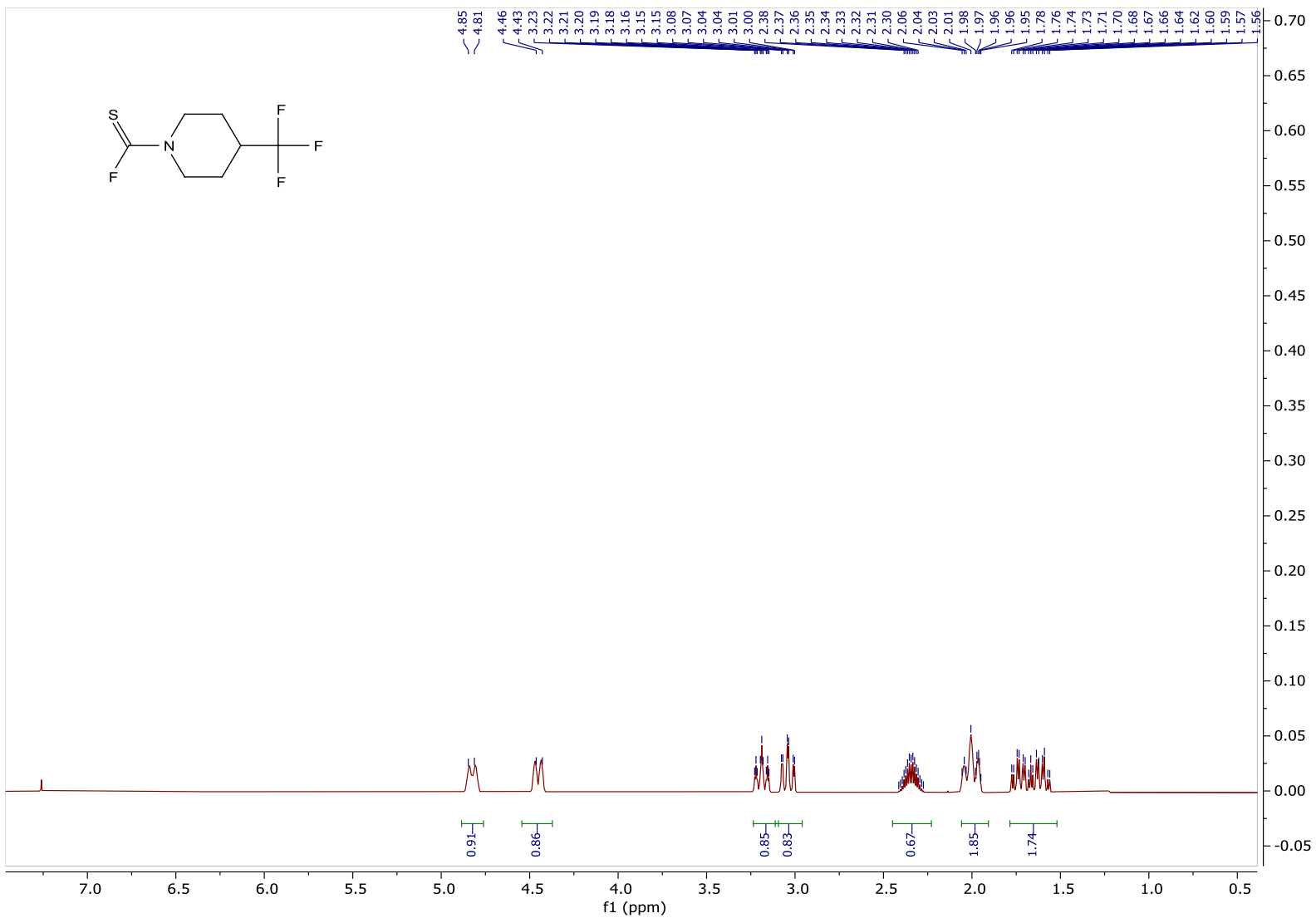


$^{1}$H NMR (400 MHz, $CDCl_3$) of **2m**.

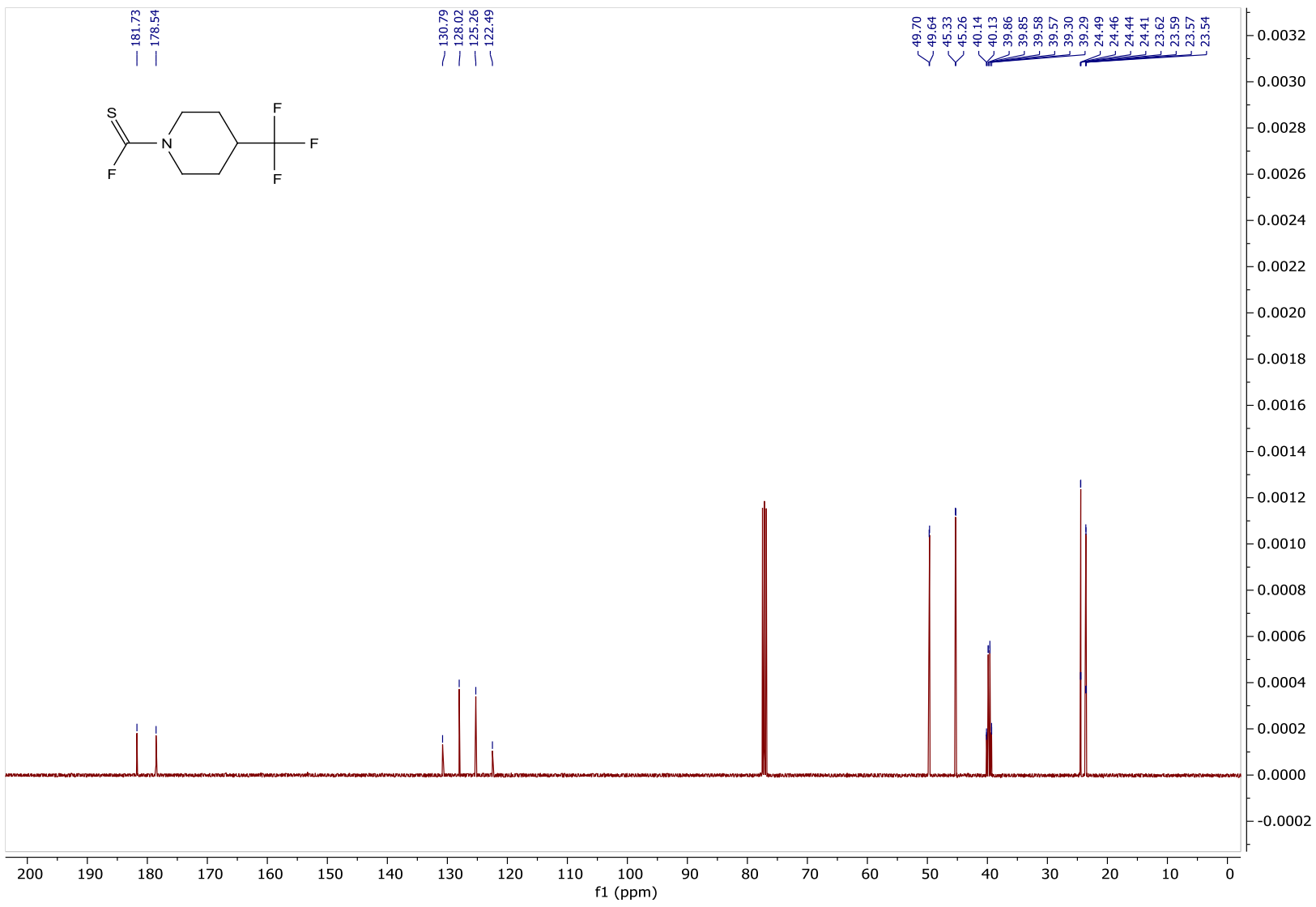


$^{13}$C NMR (101 MHz, $CDCl_3$) of **2m**.

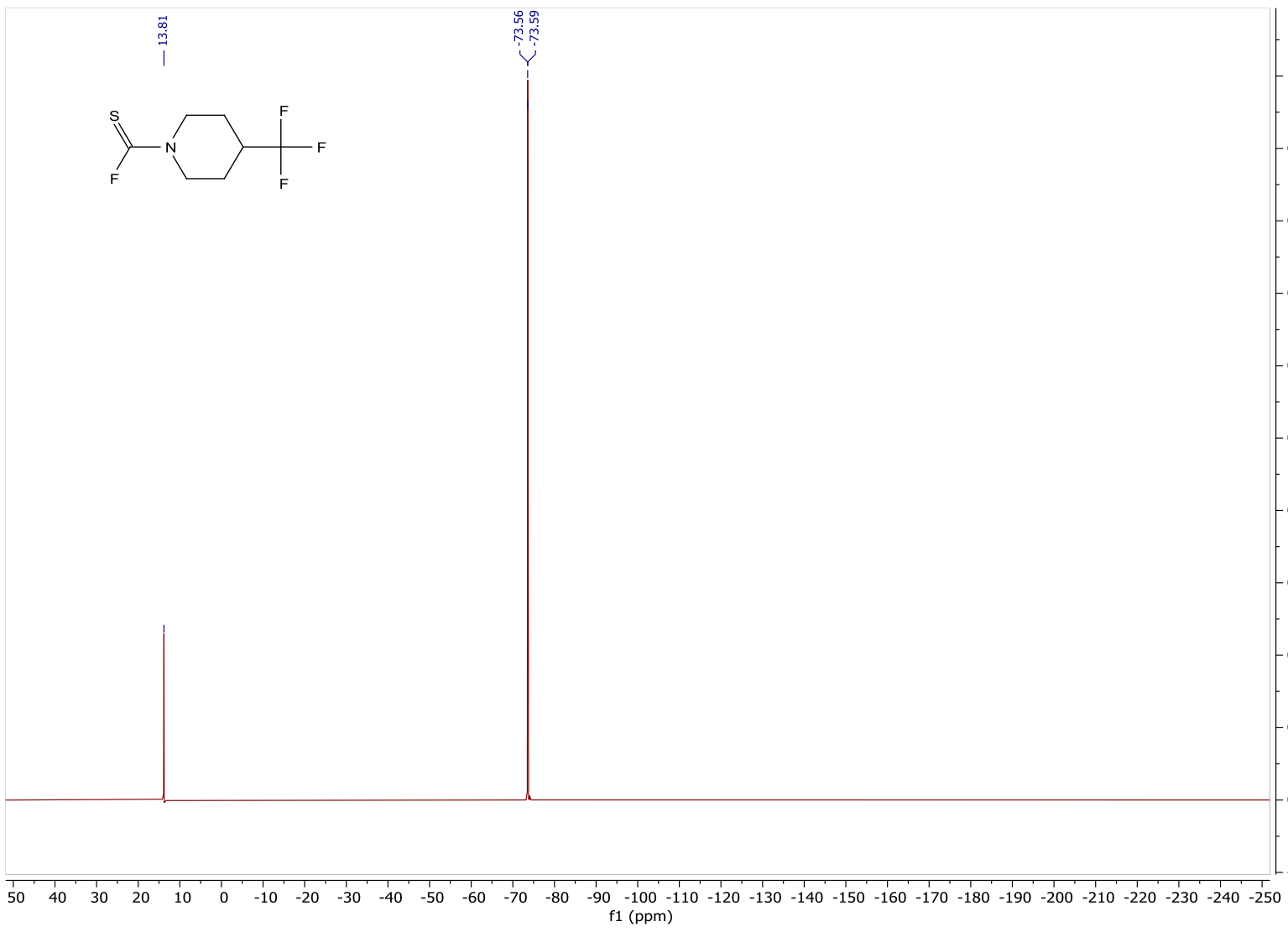


$^{19}$F NMR (376 MHz, $CDCl_3$) δ of **2m**.

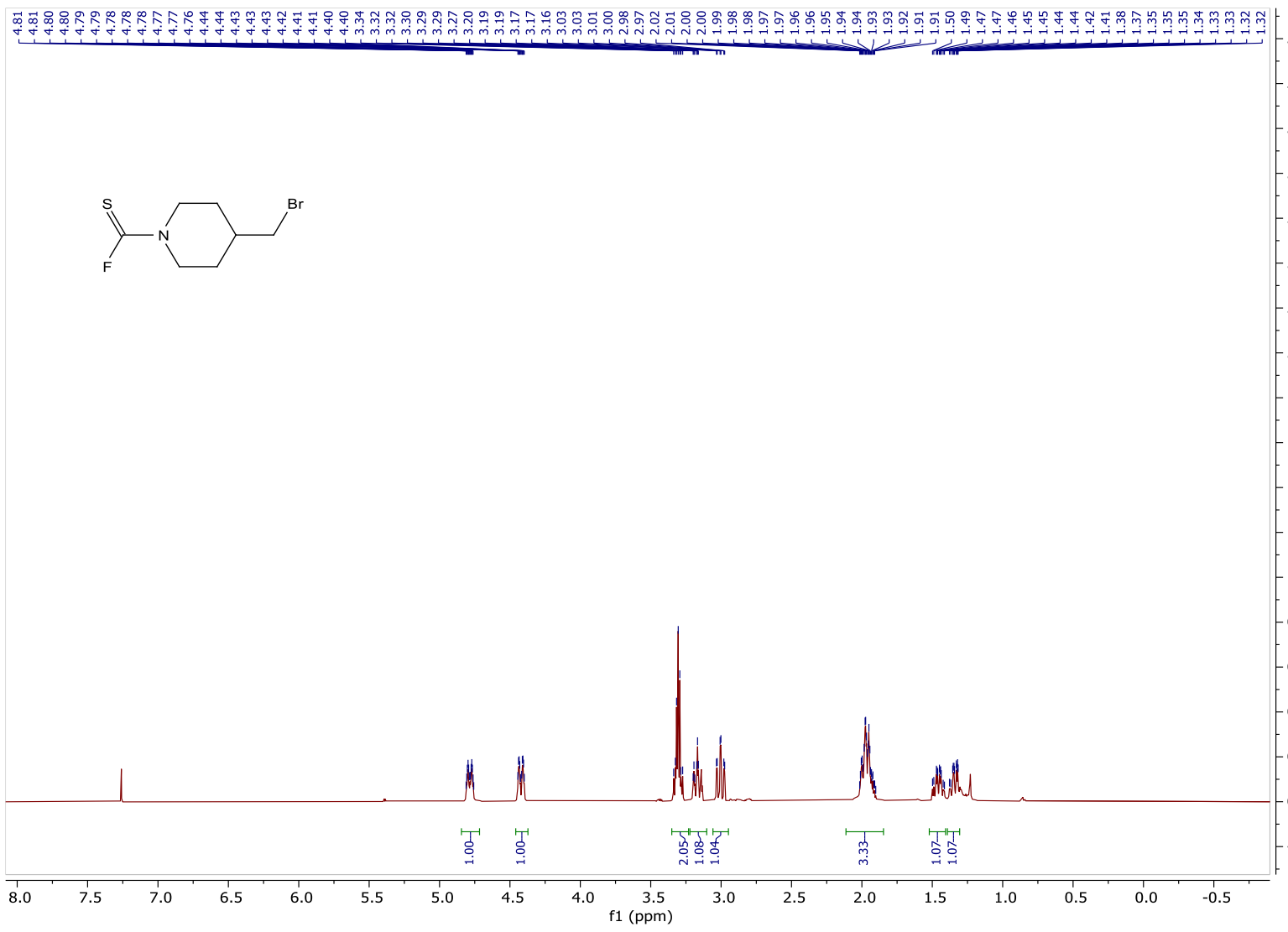


$^{1}$H NMR (500 MHz, $CDCl_3$) of **2n.**

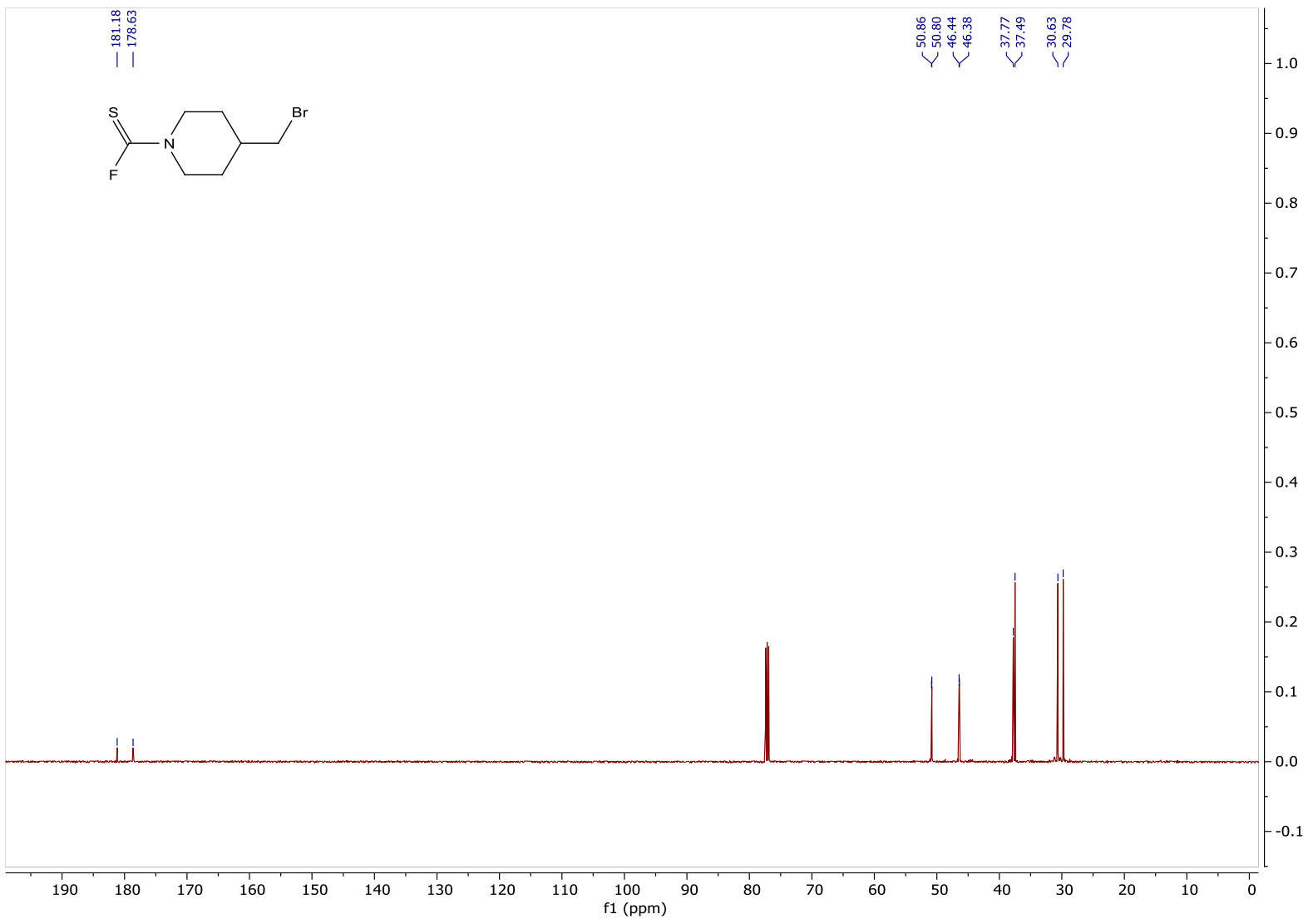


$^{13}$C NMR (126 MHz, $CDCl_3$) of **2n**.

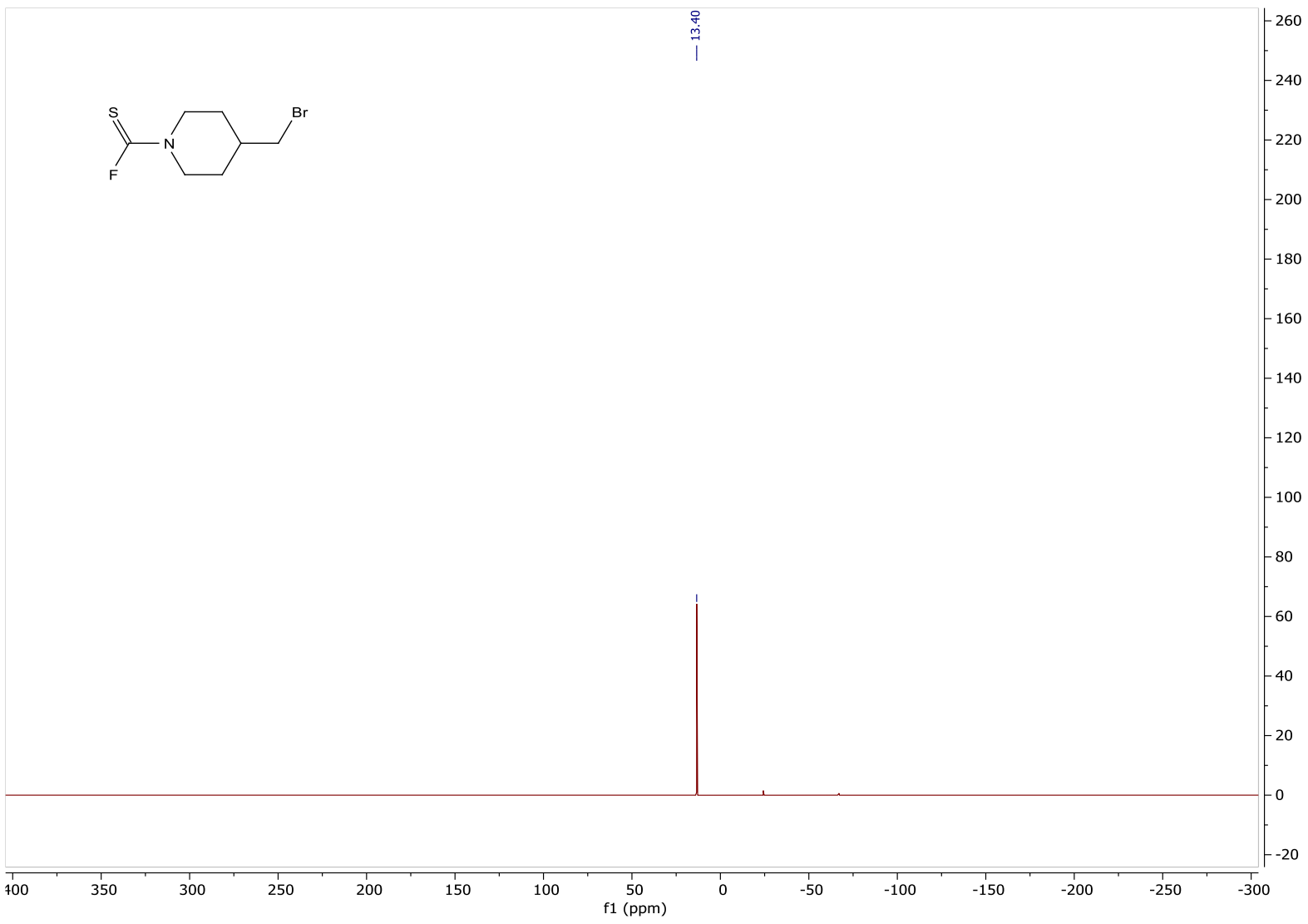


$^{19}$F NMR (471 MHz, $CDCl_3$) of **2n**.

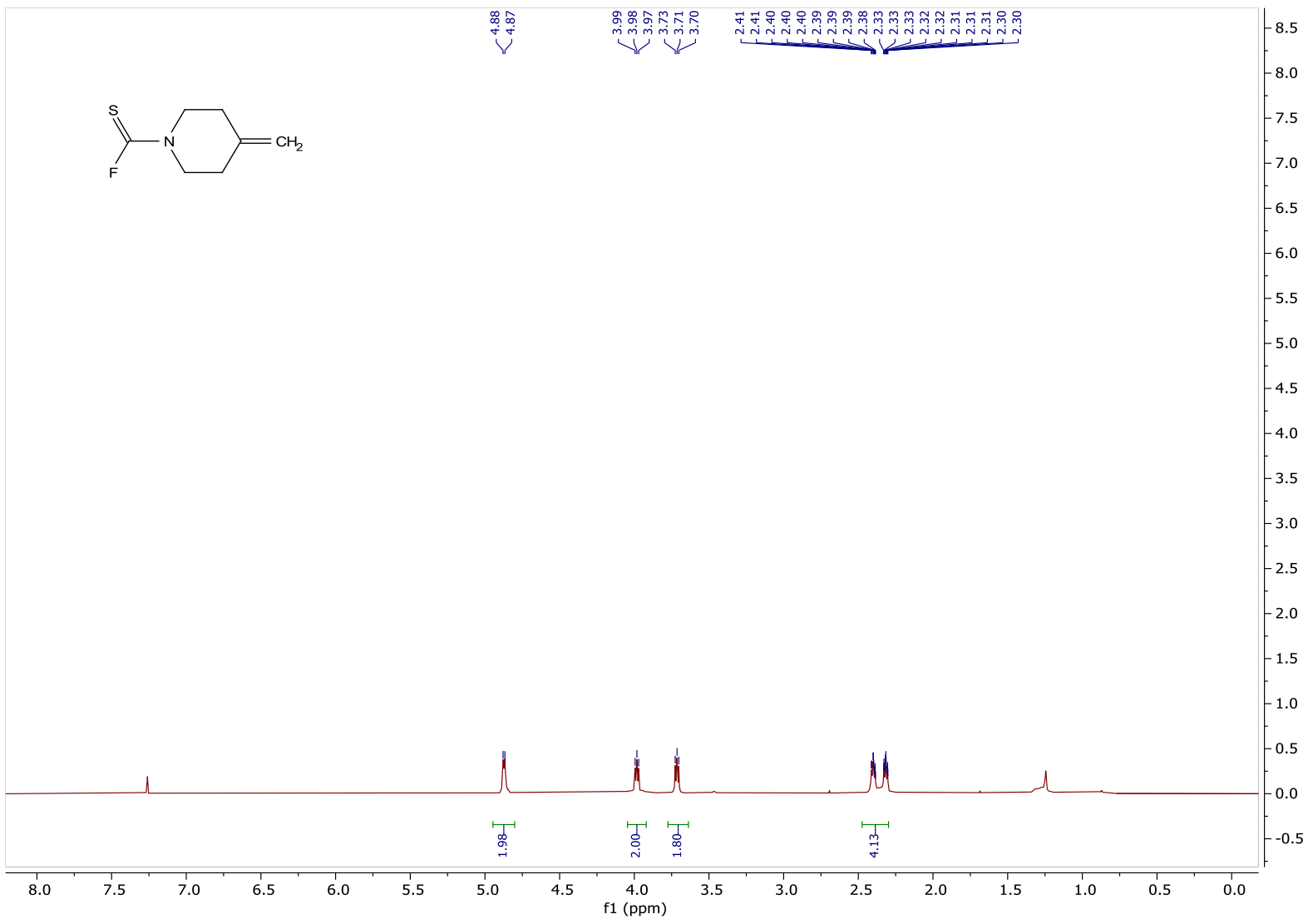

$^{1}$H NMR (500 MHz, $CDCl_3$) of **2o**.

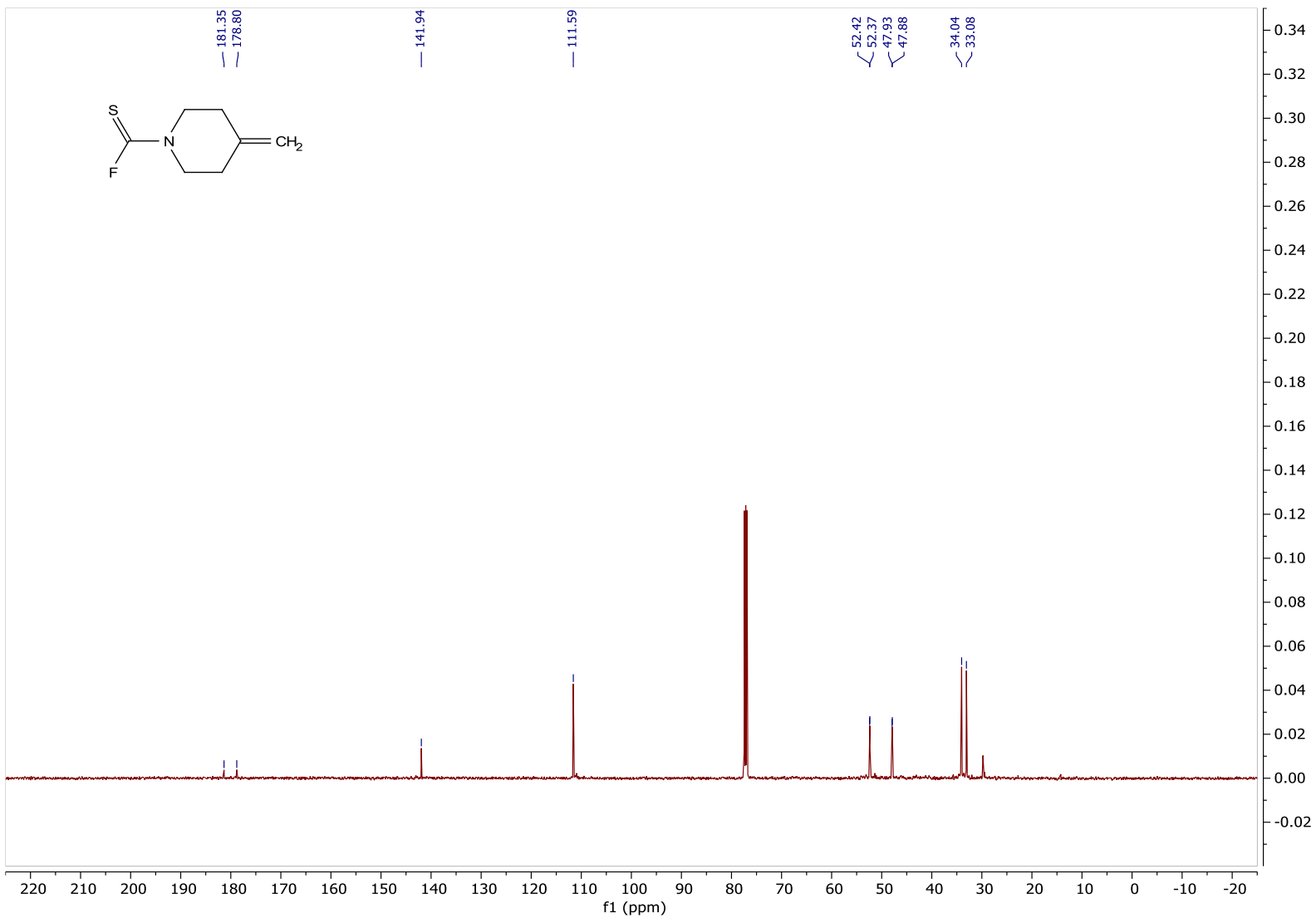

$^{13}$C NMR (126 MHz, $CDCl_3$) of **2o**.

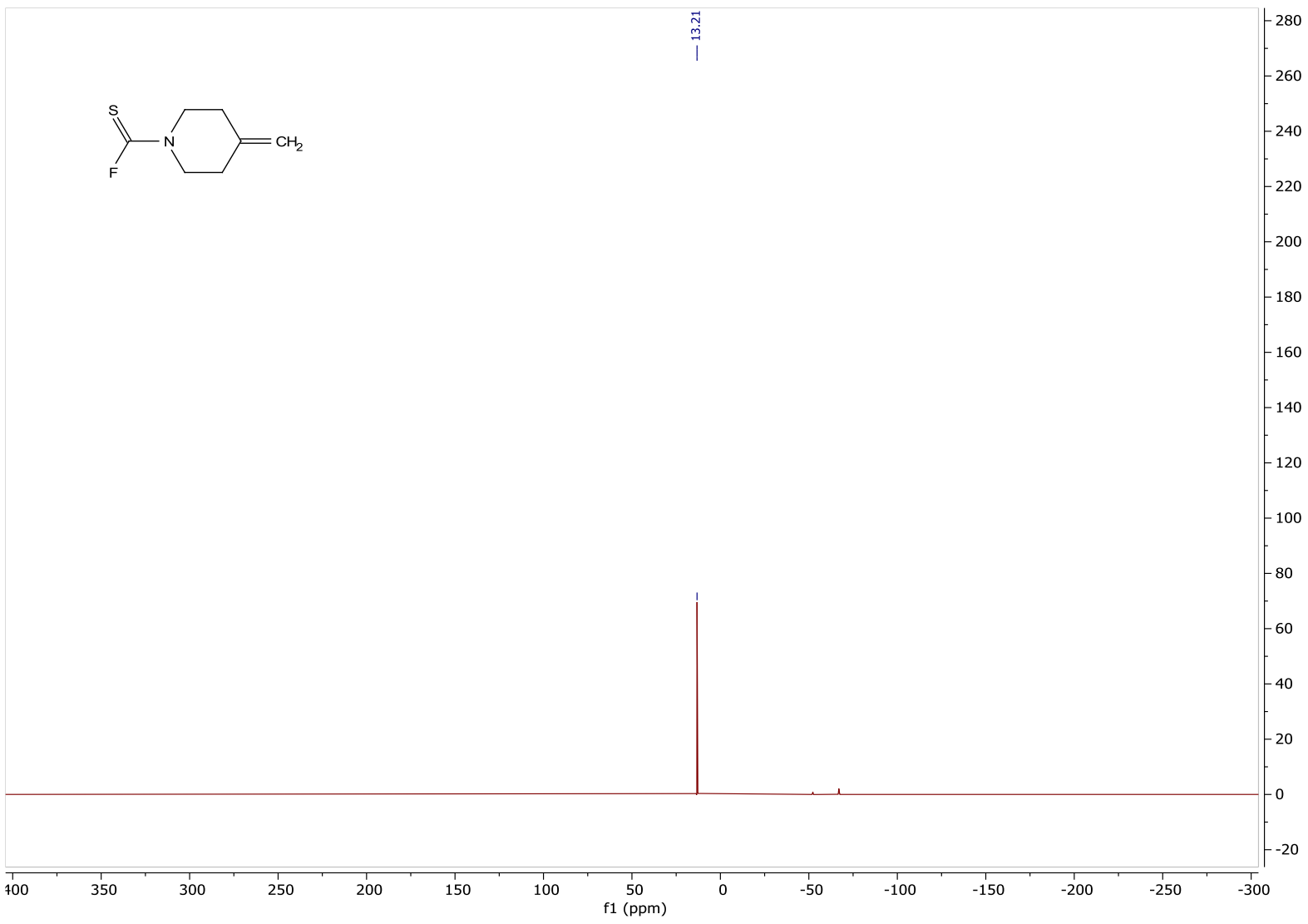

$^{19}F$ NMR (471 MHz, $CDCl_3$) of **2o**.

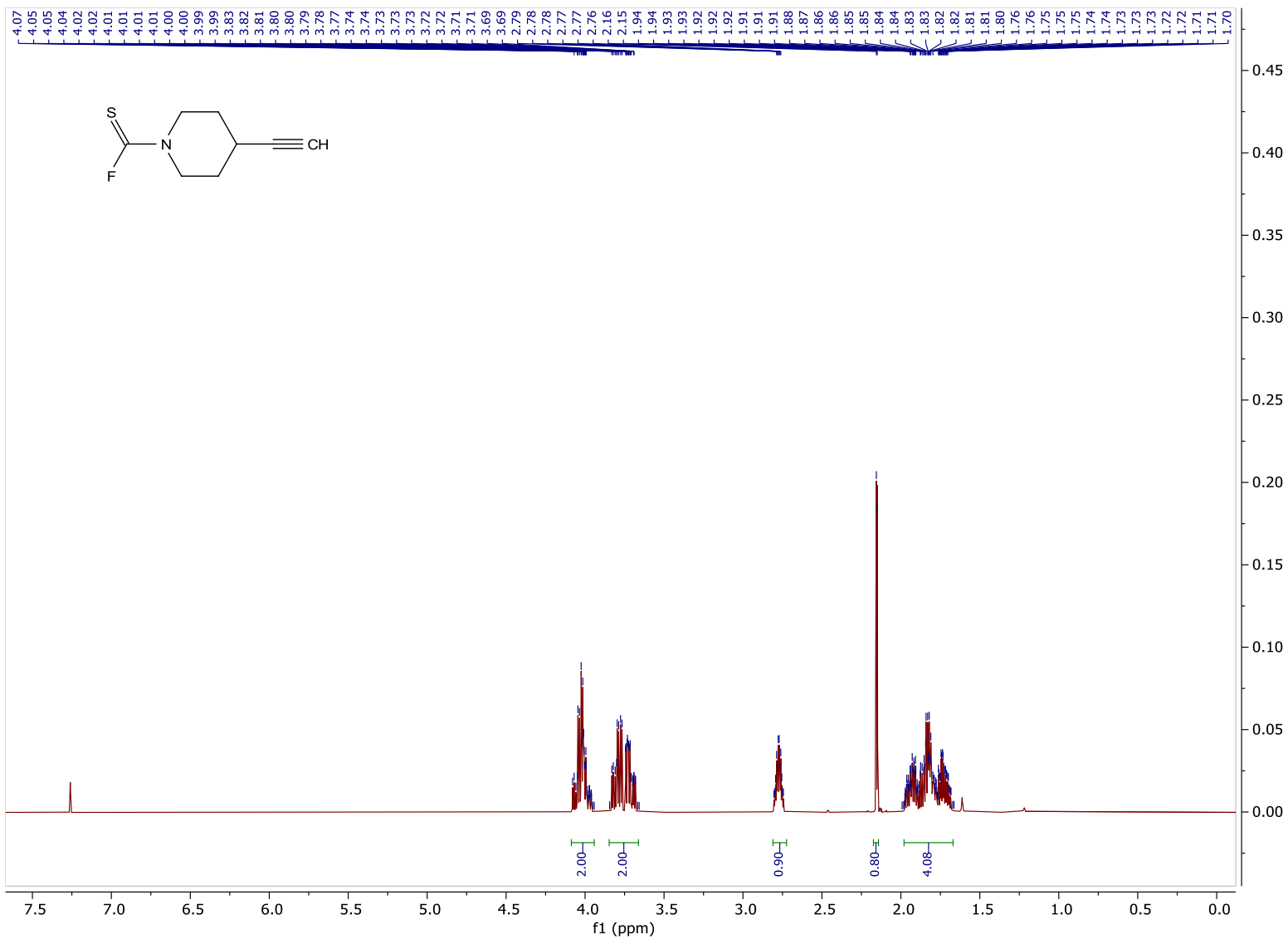

$^{1}H$ NMR (400 MHz, $CDCl_3$) of **2p**.

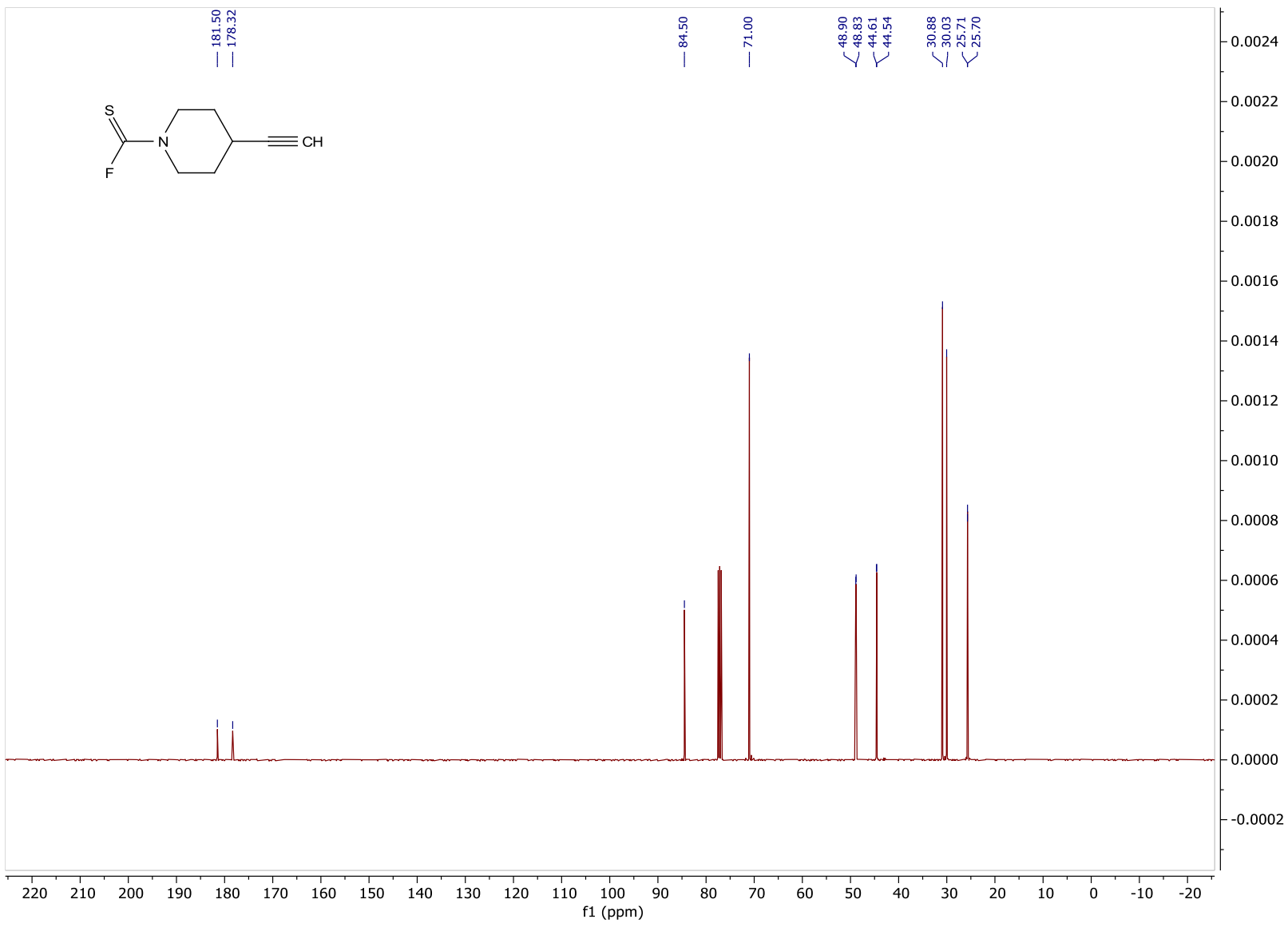

$^{13}C$ NMR (101 MHz, $CDCl_3$) of **2p**.

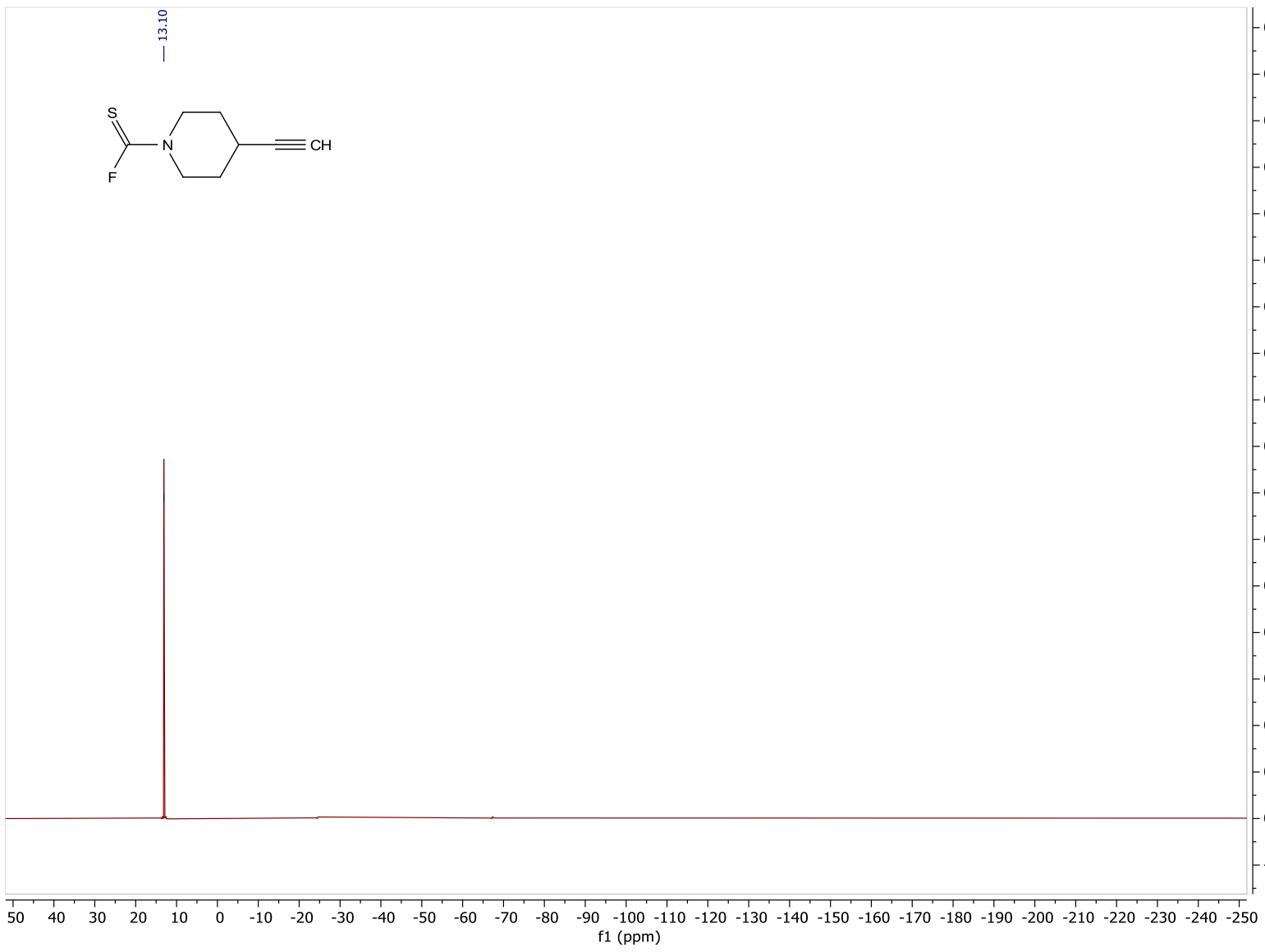

$^{19}F$ NMR (376 MHz, $CDCl_3$) of **2p**.

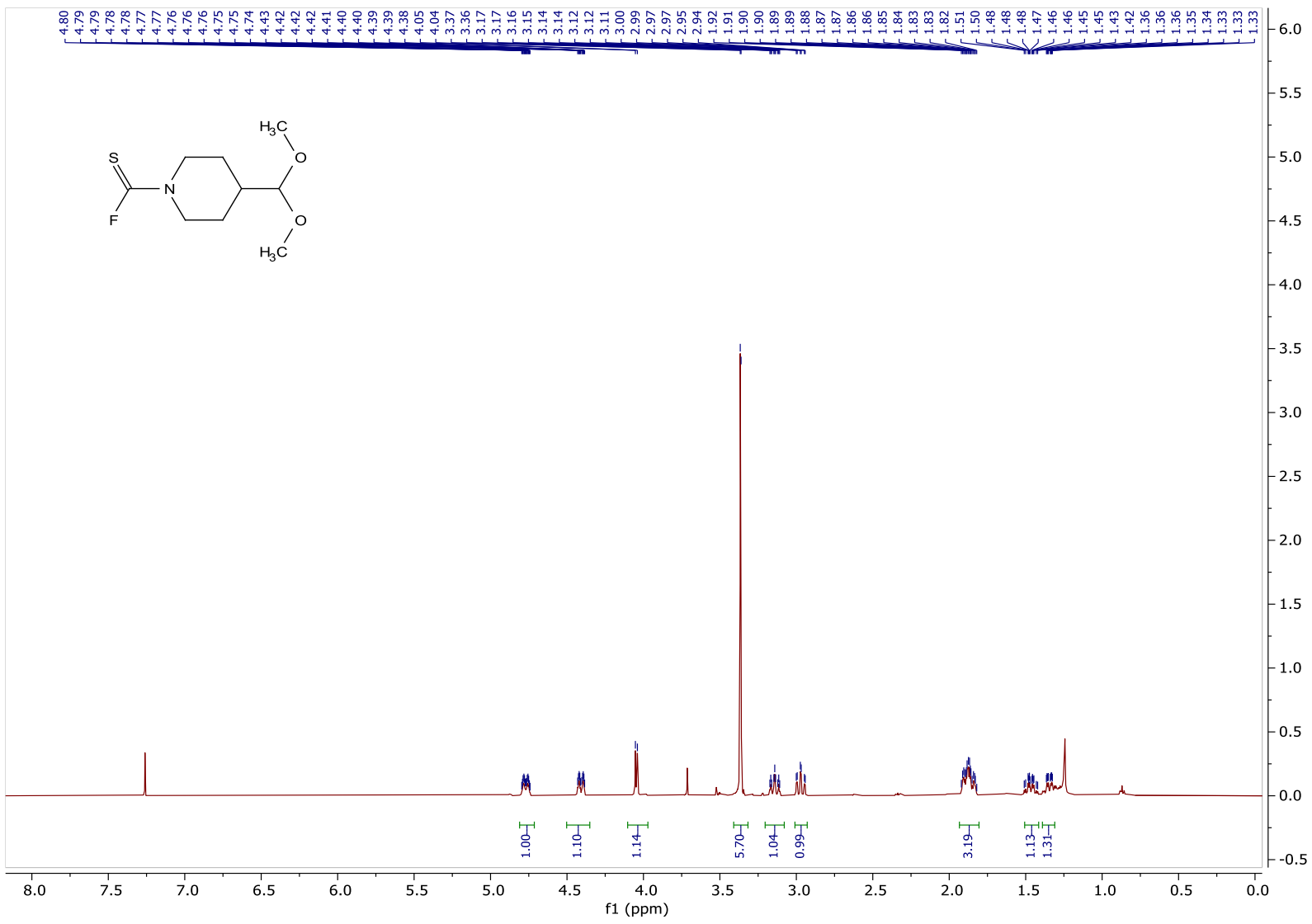

$^{1}$H NMR (500 MHz, $CDCl_3$) of **2q**.

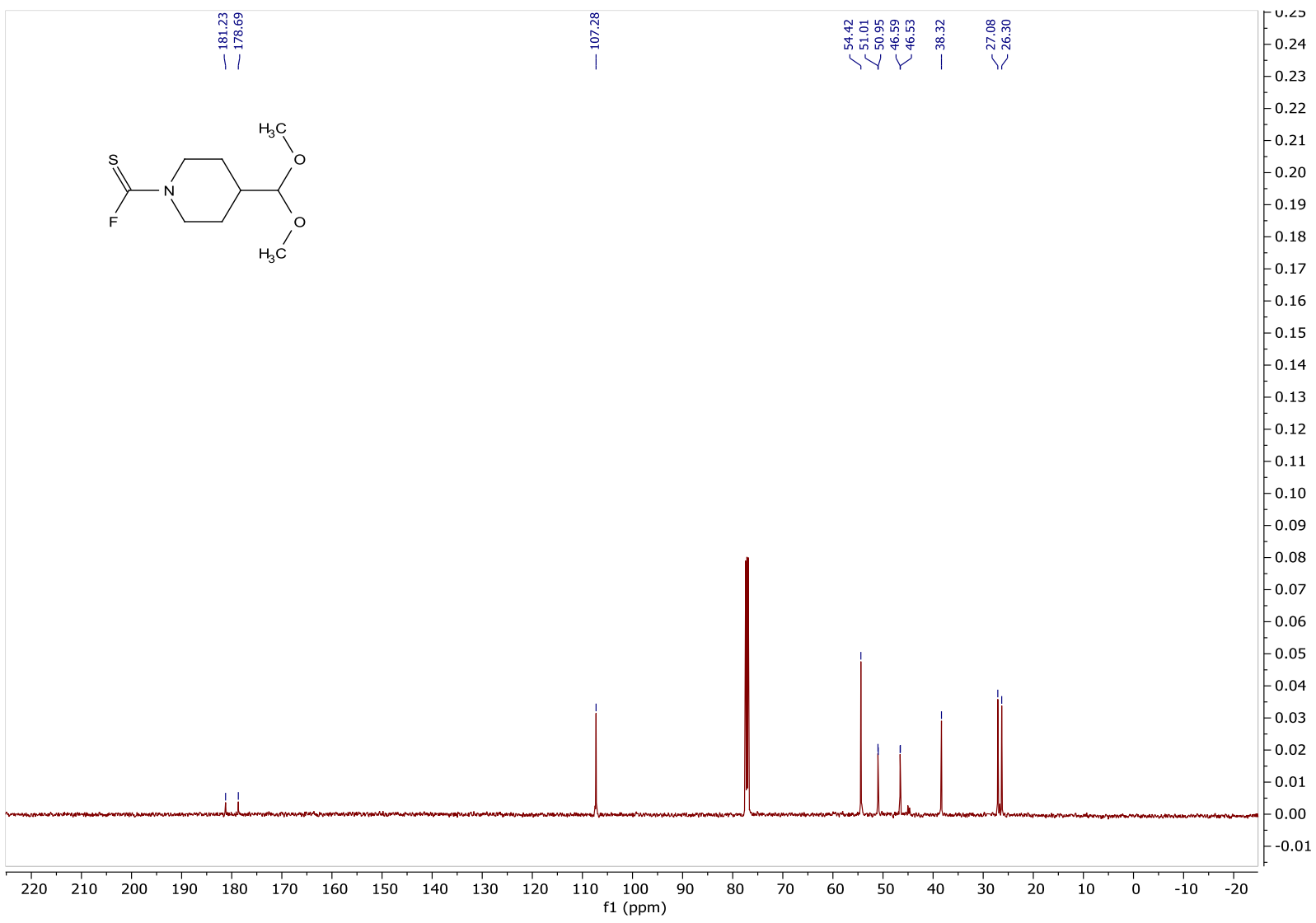

$^{13}$C NMR (126 MHz, $CDCl_3$) of **2q**.

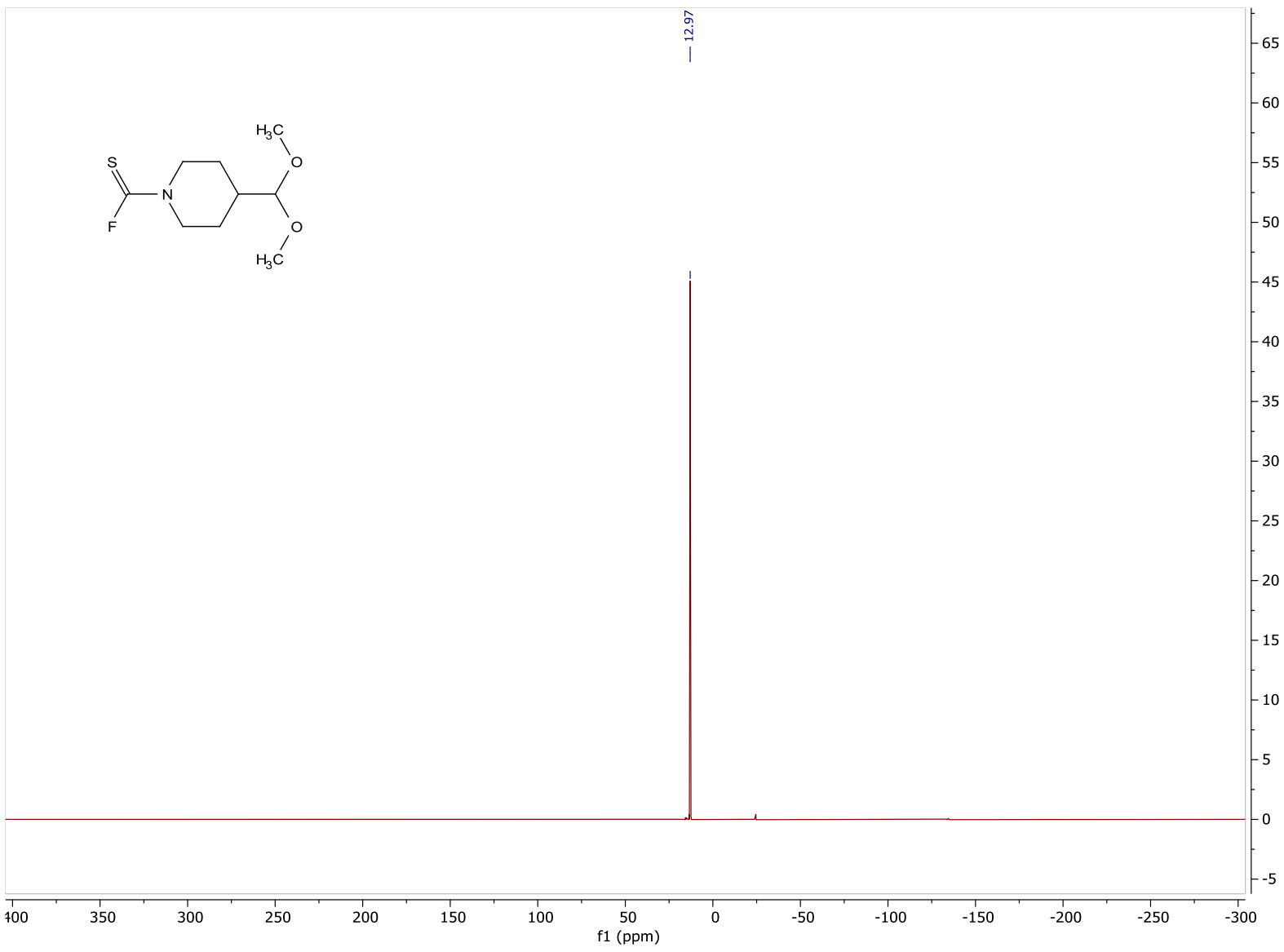

$^{19}$F NMR (471 MHz, $CDCl_3$) of **2q**.

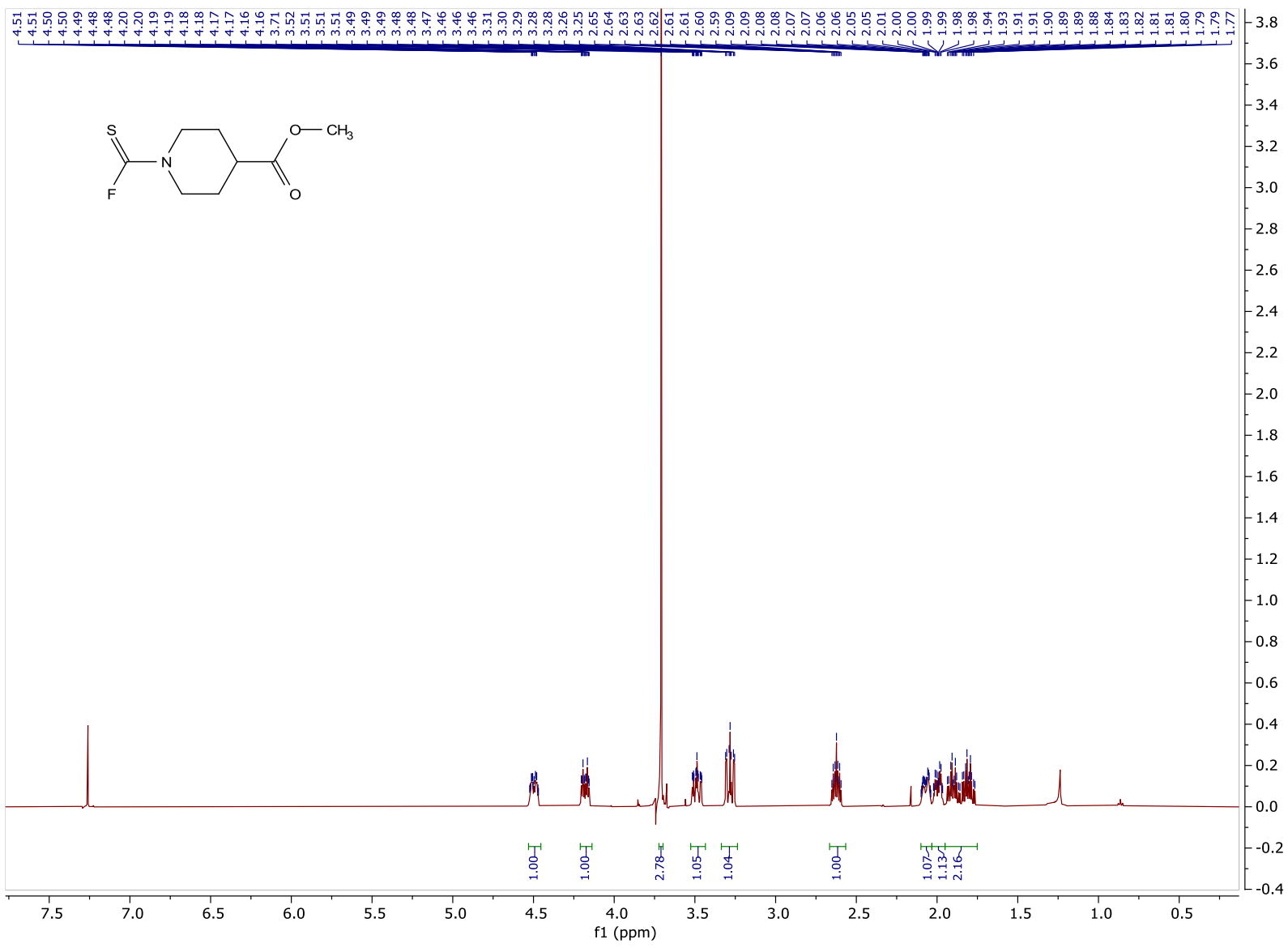

$^{1}$H NMR (500 MHz, $CDCl_3$) of **2r**.

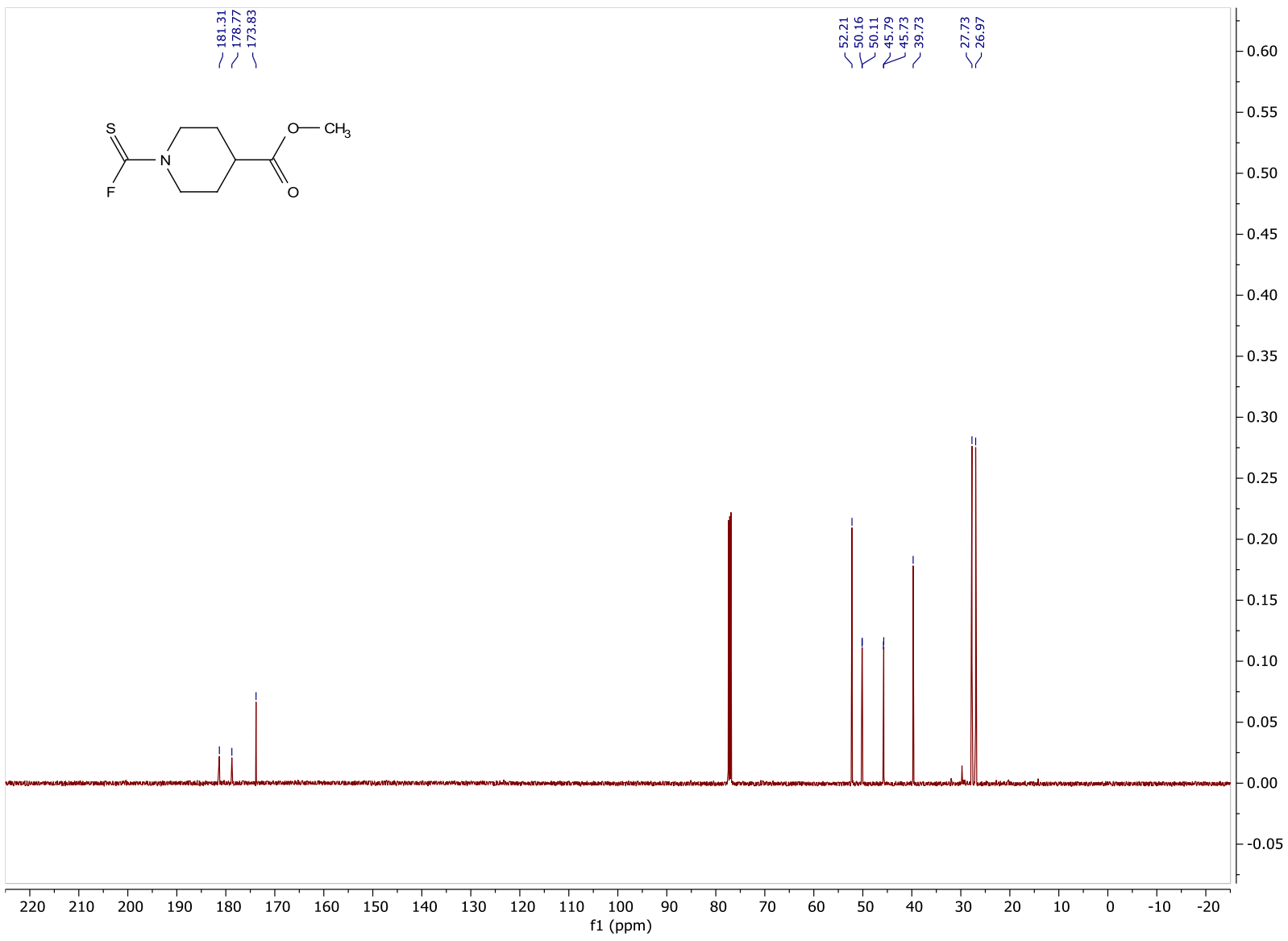


$^{13}C$ NMR (126 MHz, $CDCl_3$) of **2r**.

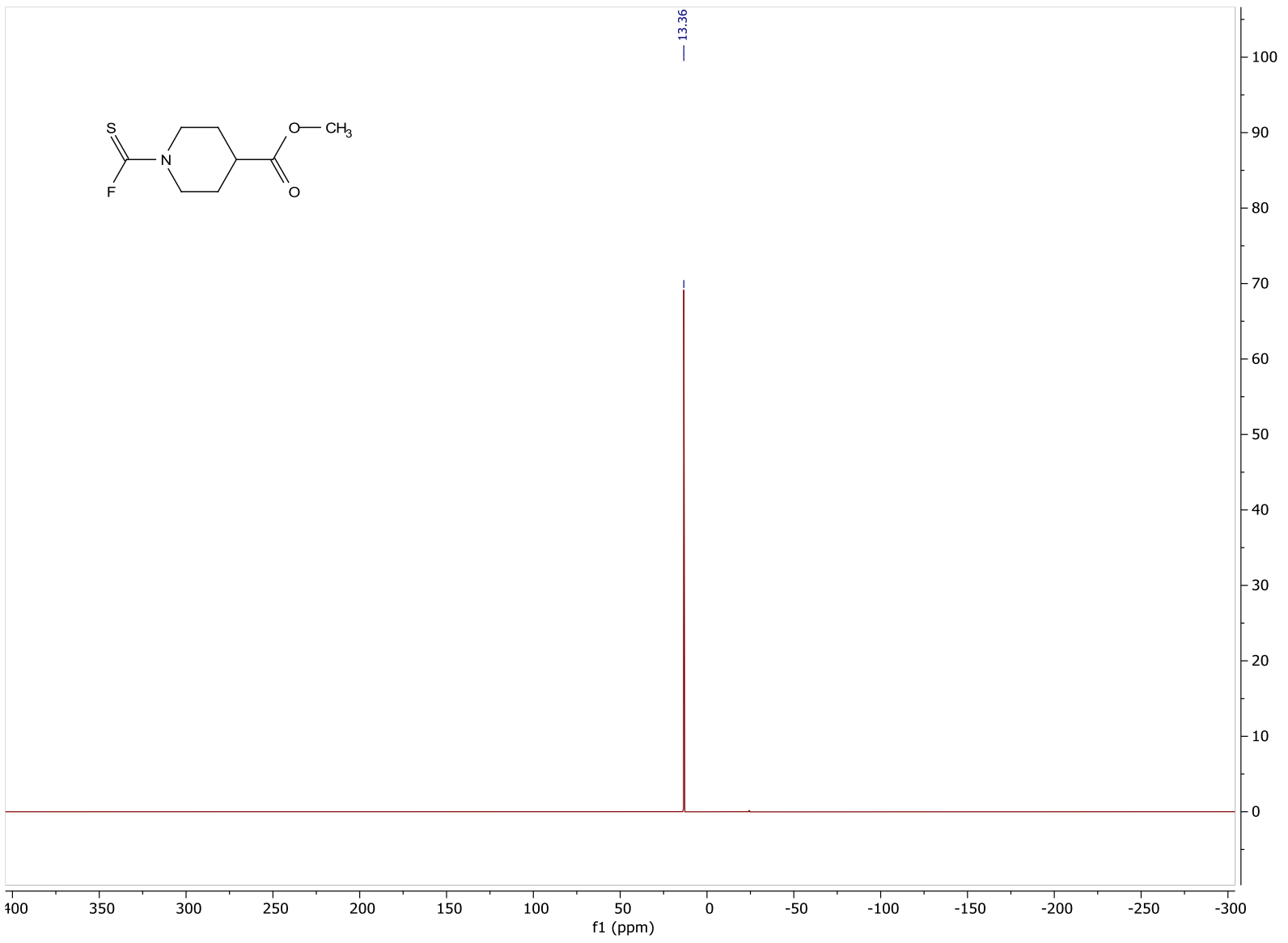


$^{19}F$ NMR (471 MHz, $CDCl_3$) of **2r**.

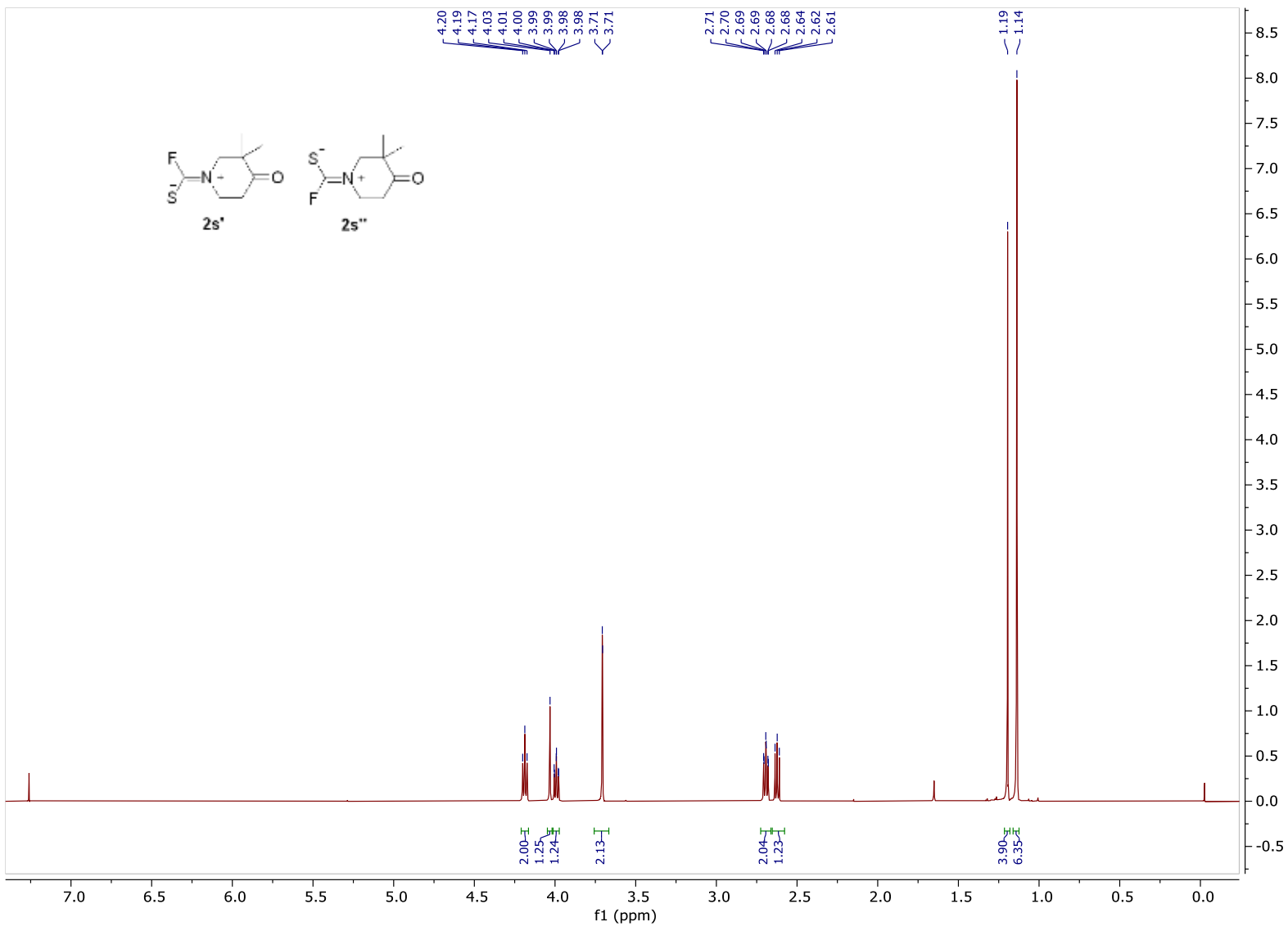

$^{1}$H NMR (500 MHz, $CDCl_3$) of **2s’** and **2s”**.

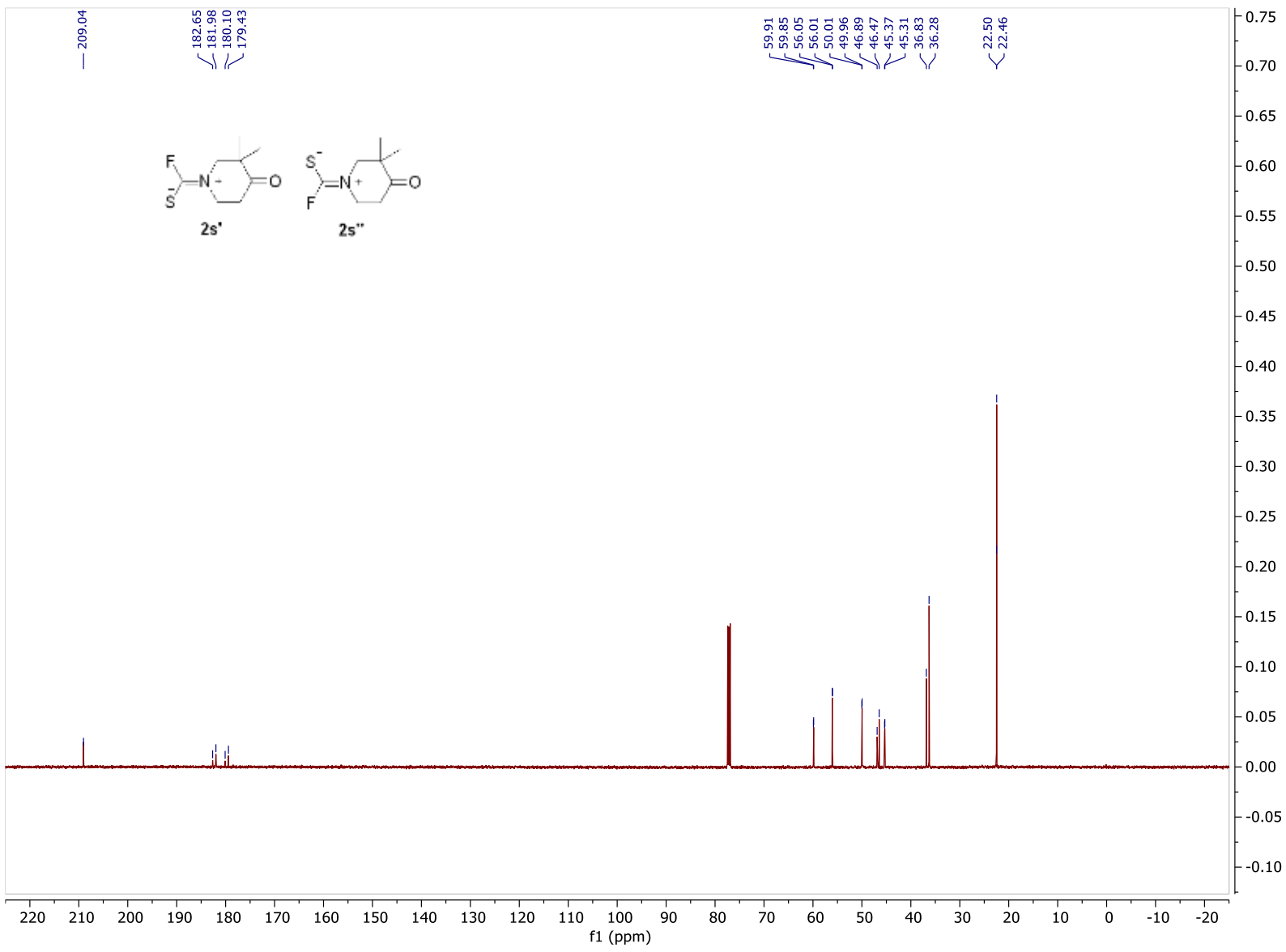

$^{13}$C NMR (126 MHz, $CDCl_3$) of **2s’** and **2s”**.

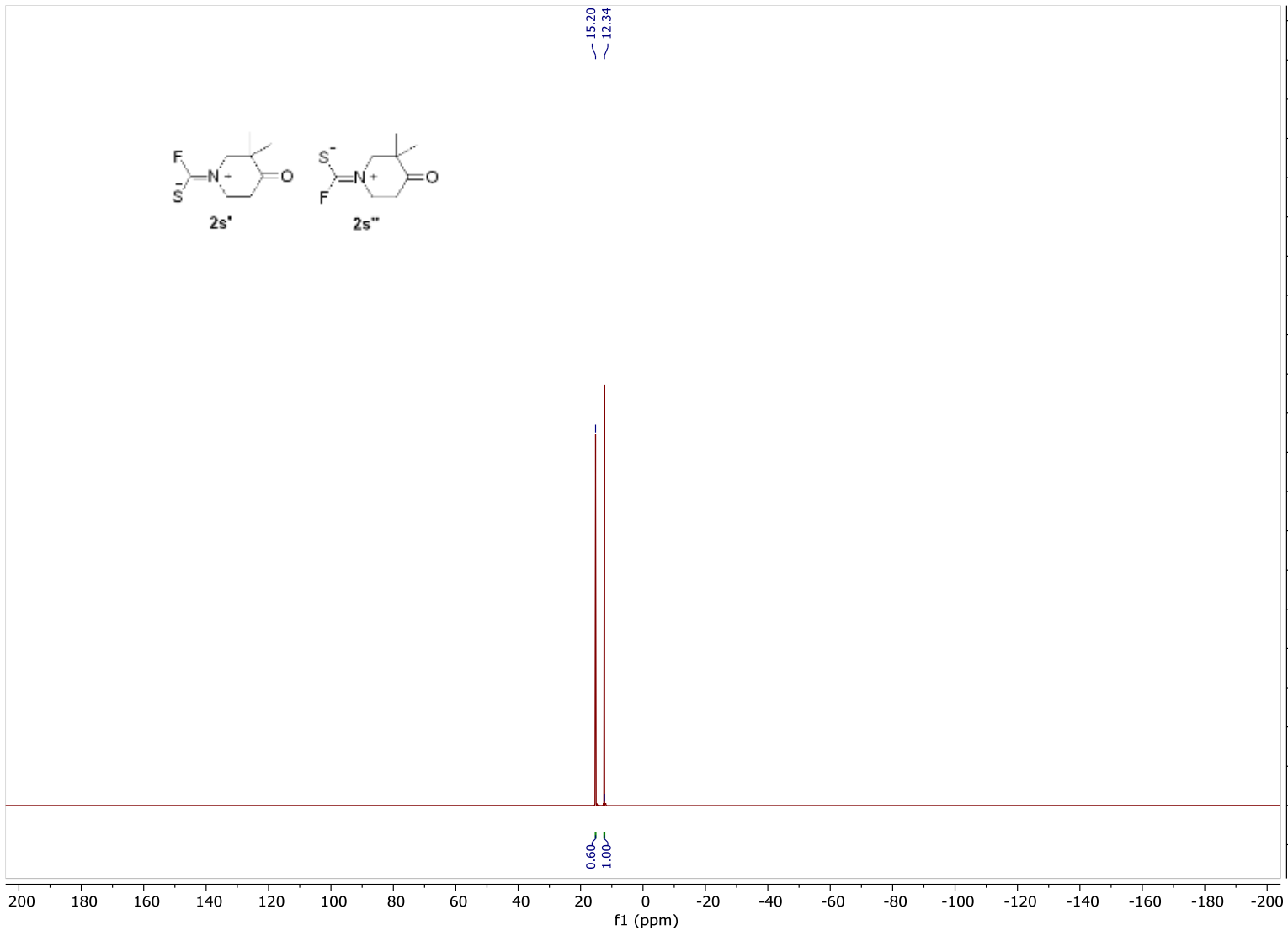

$^{19}F$ NMR (471 MHz, $CDCl_3$) of **2s'** and **2s''**.

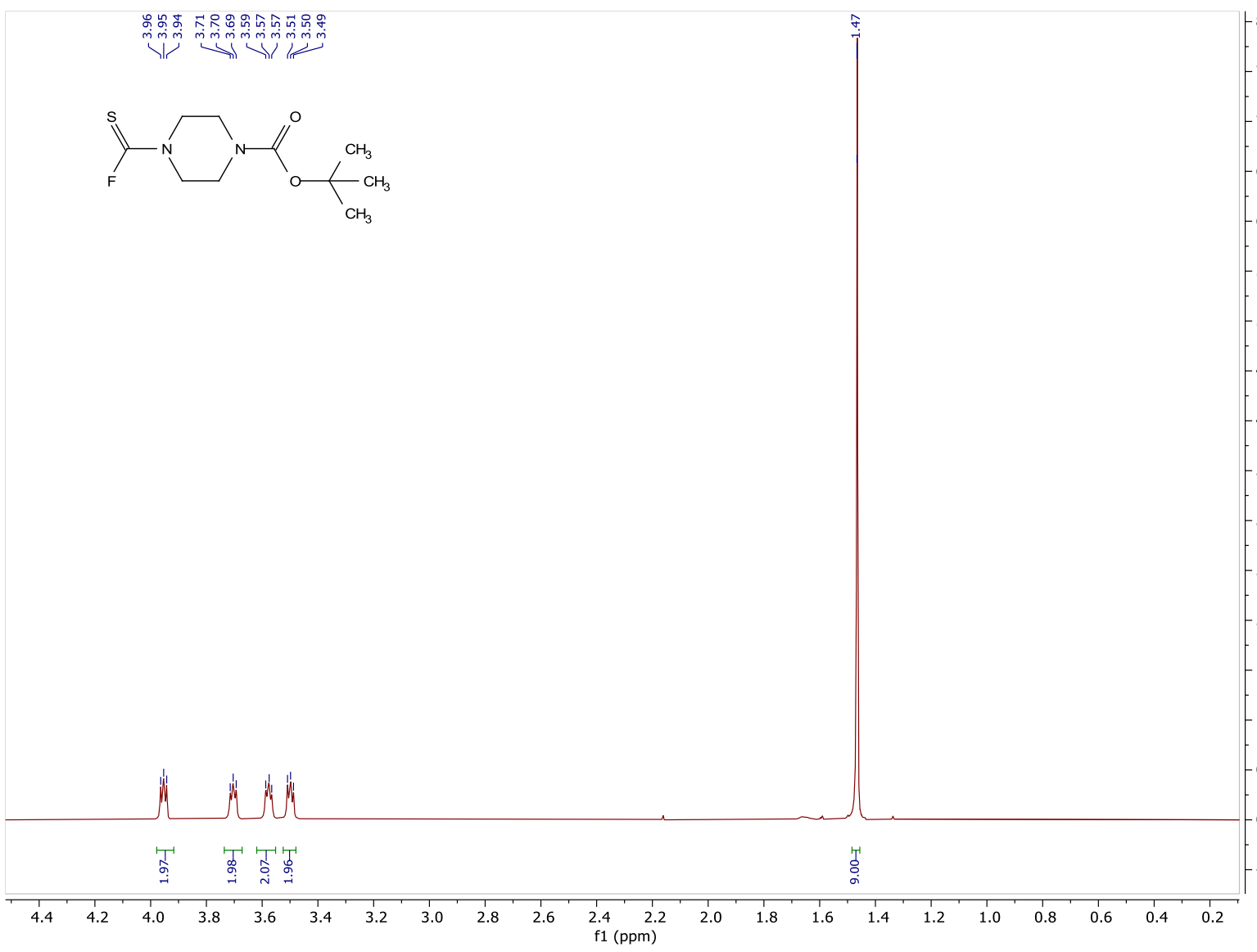

$^{1}H$ NMR (500 MHz, $CDCl_3$) of **2t**.

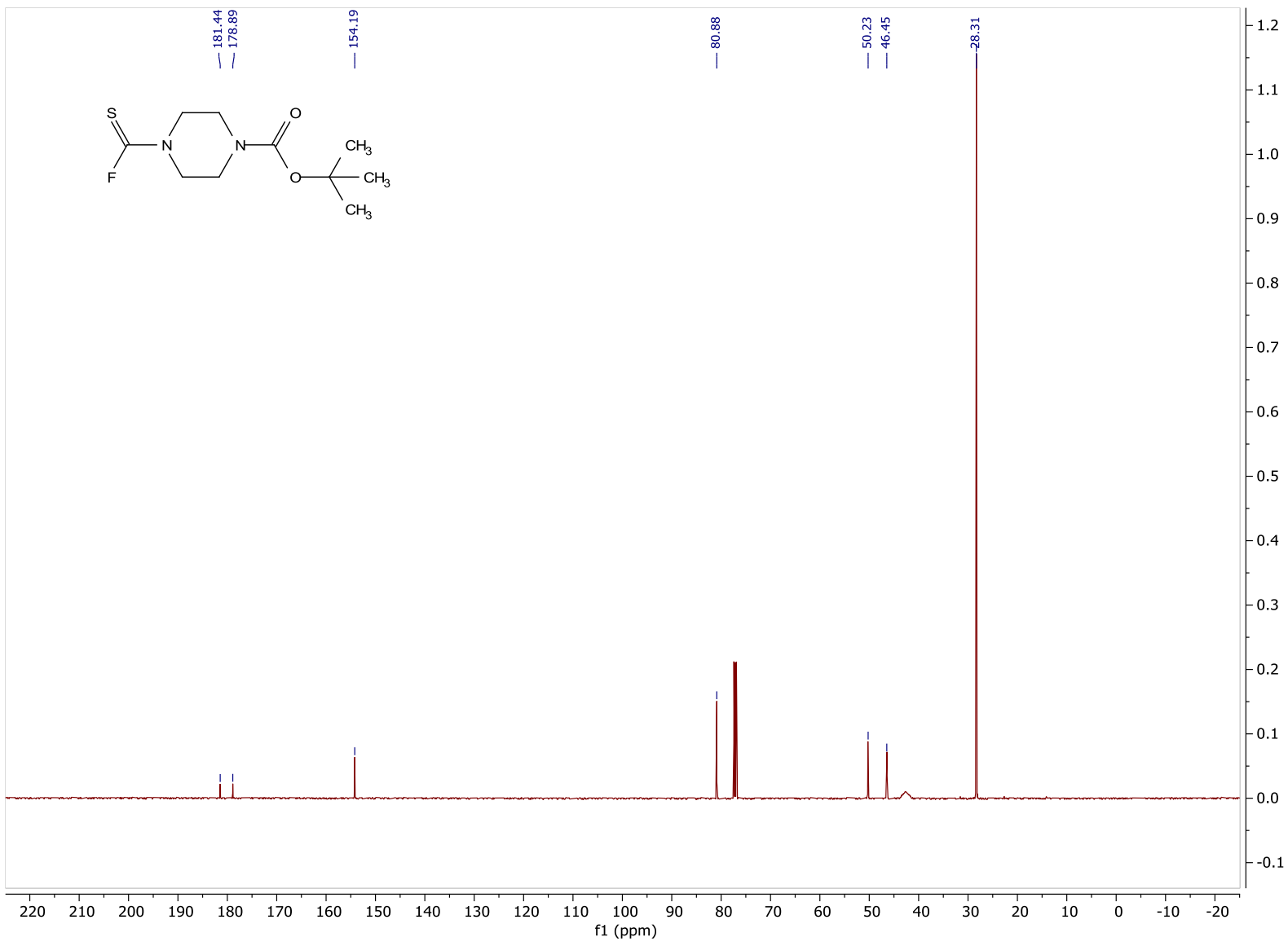


$^{13}C$ NMR (126 MHz, $CDCl_3$) of **2t**.

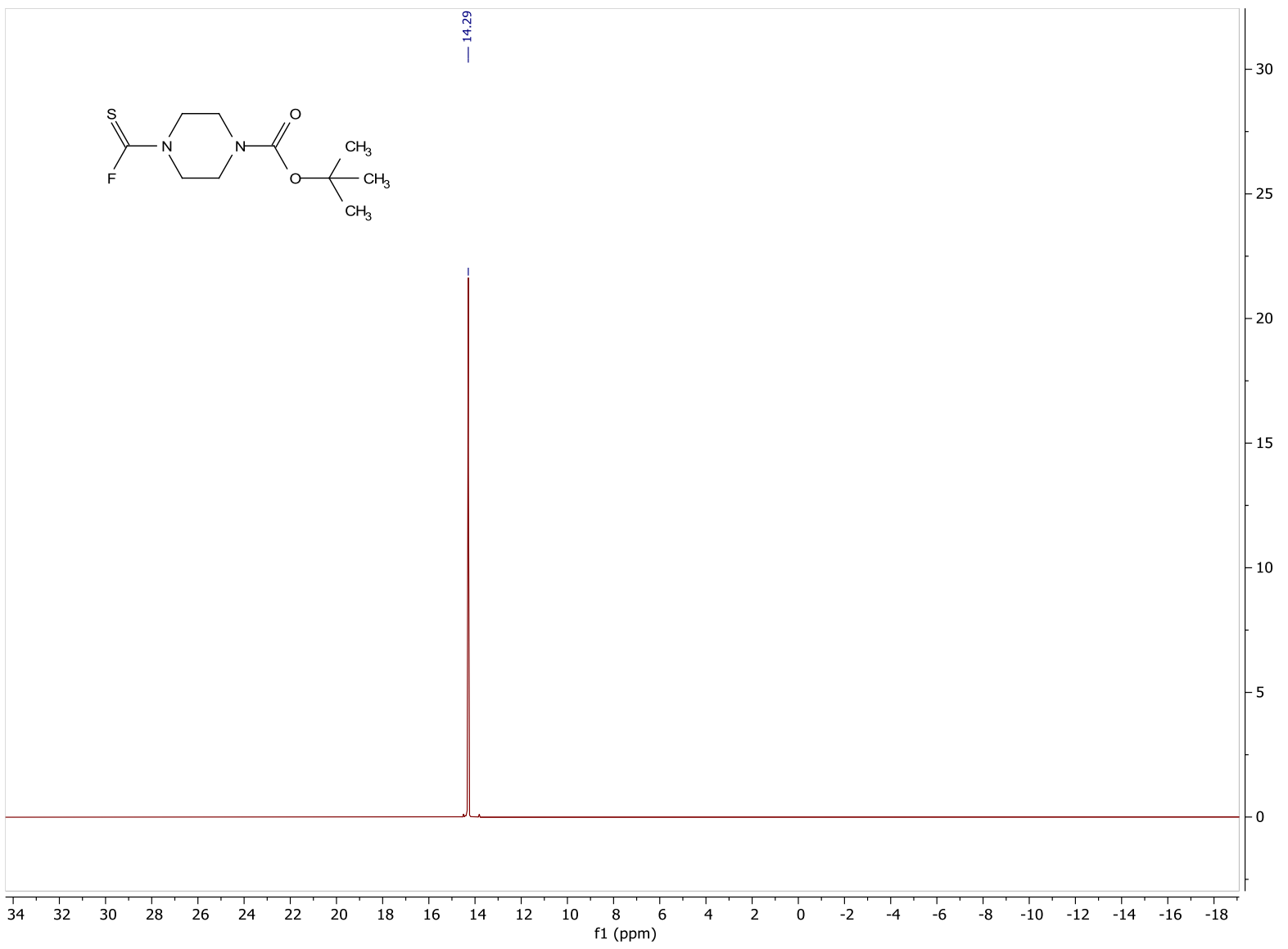


$^{19}F$ NMR (471 MHz, $CDCl_3$) of **2t**.

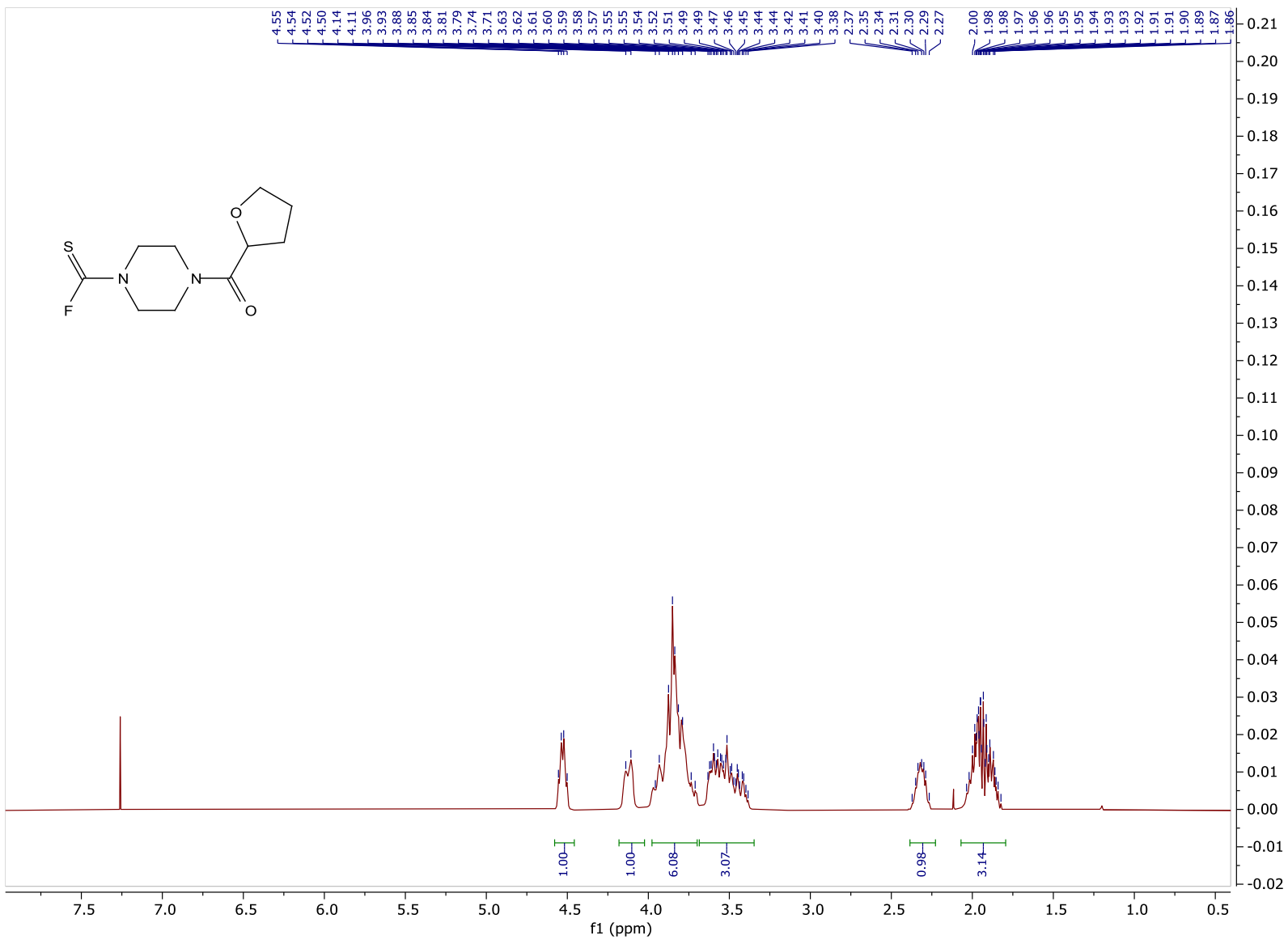


$^{1}$H NMR (400 MHz, $CDCl_3$) of **2u**.

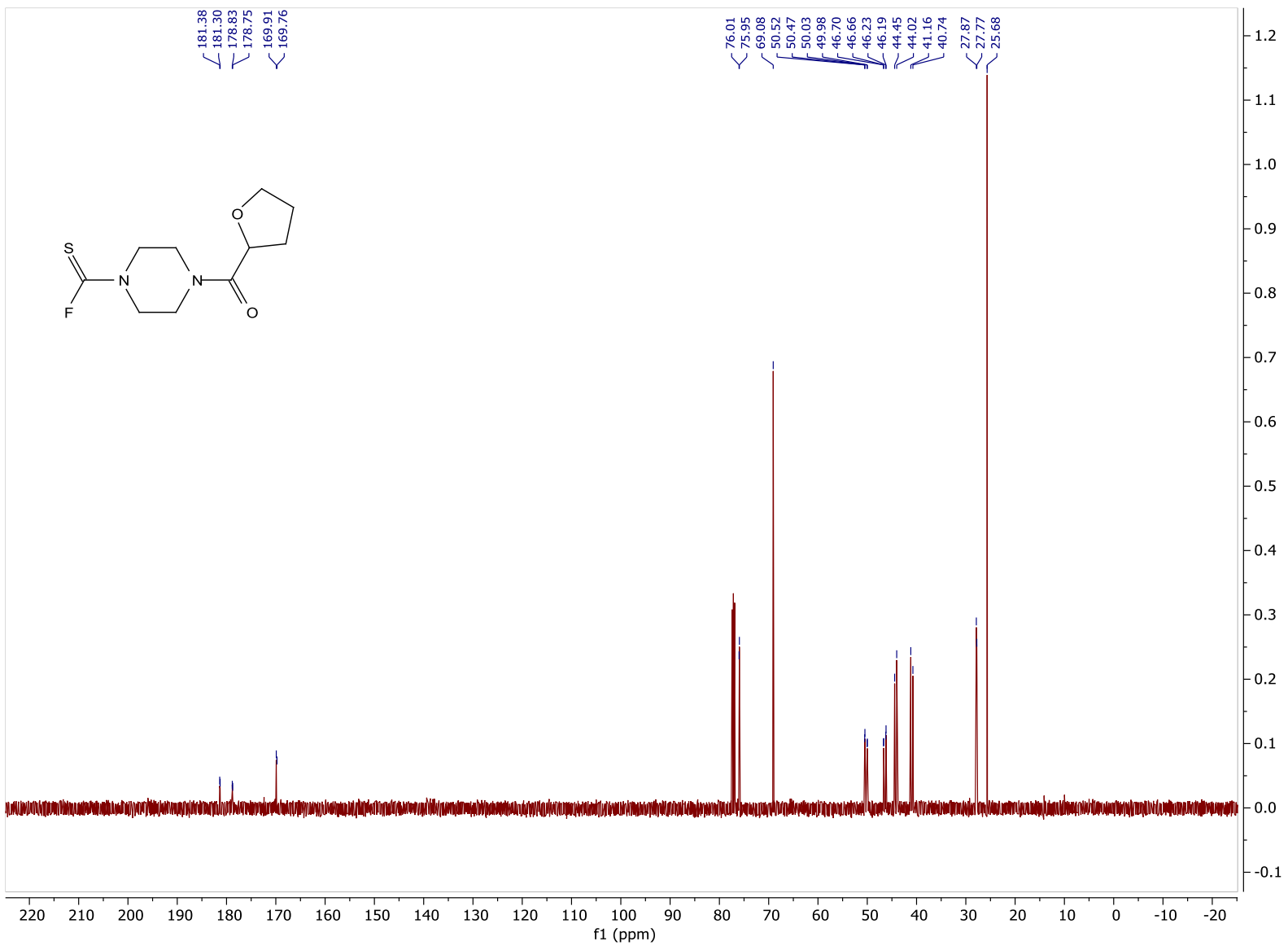


$^{13}$C NMR (126 MHz, $CDCl_3$) of **2u**

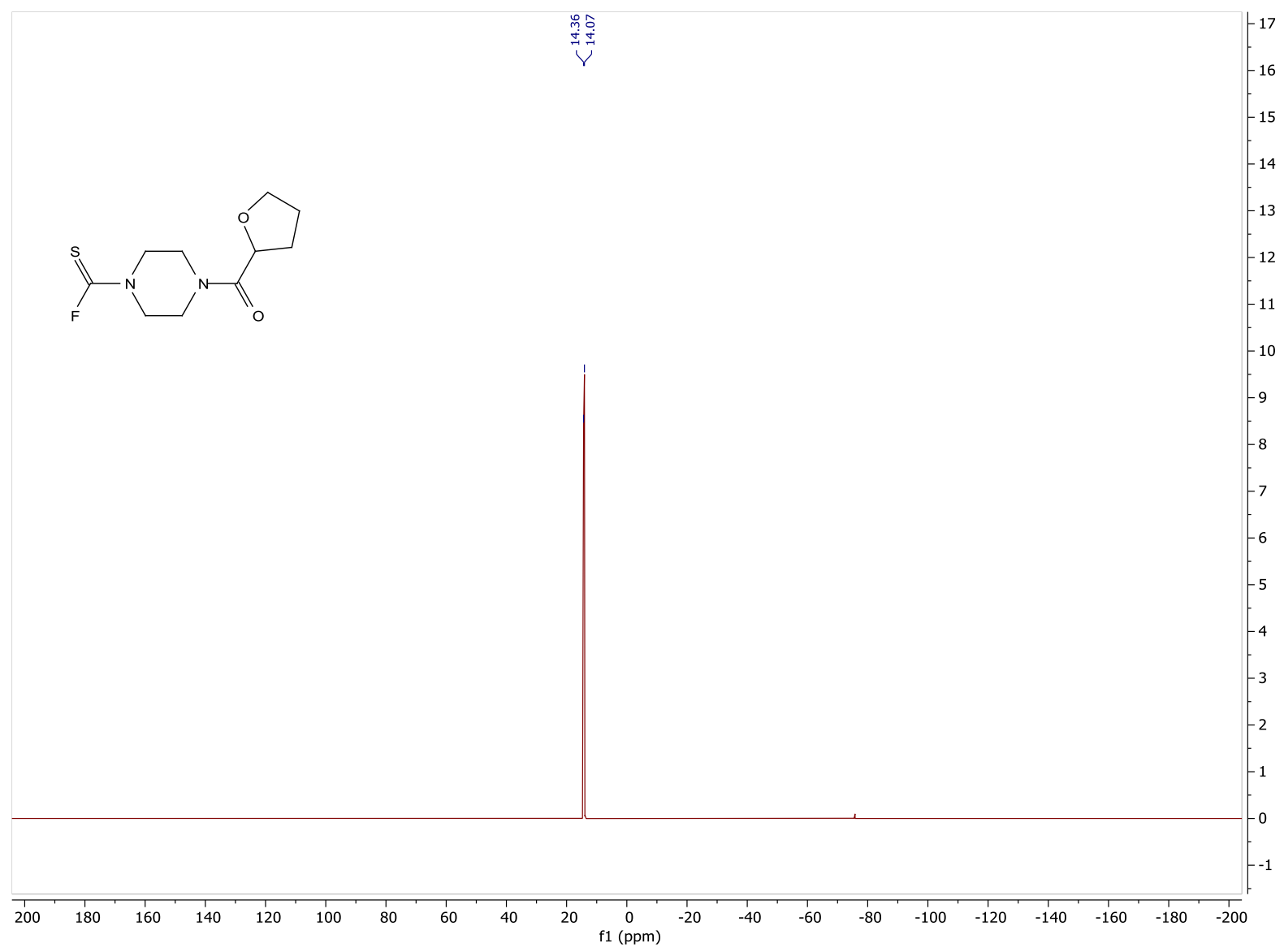

$^{19}$F NMR (471 MHz, $CDCl_3$) of **2u**

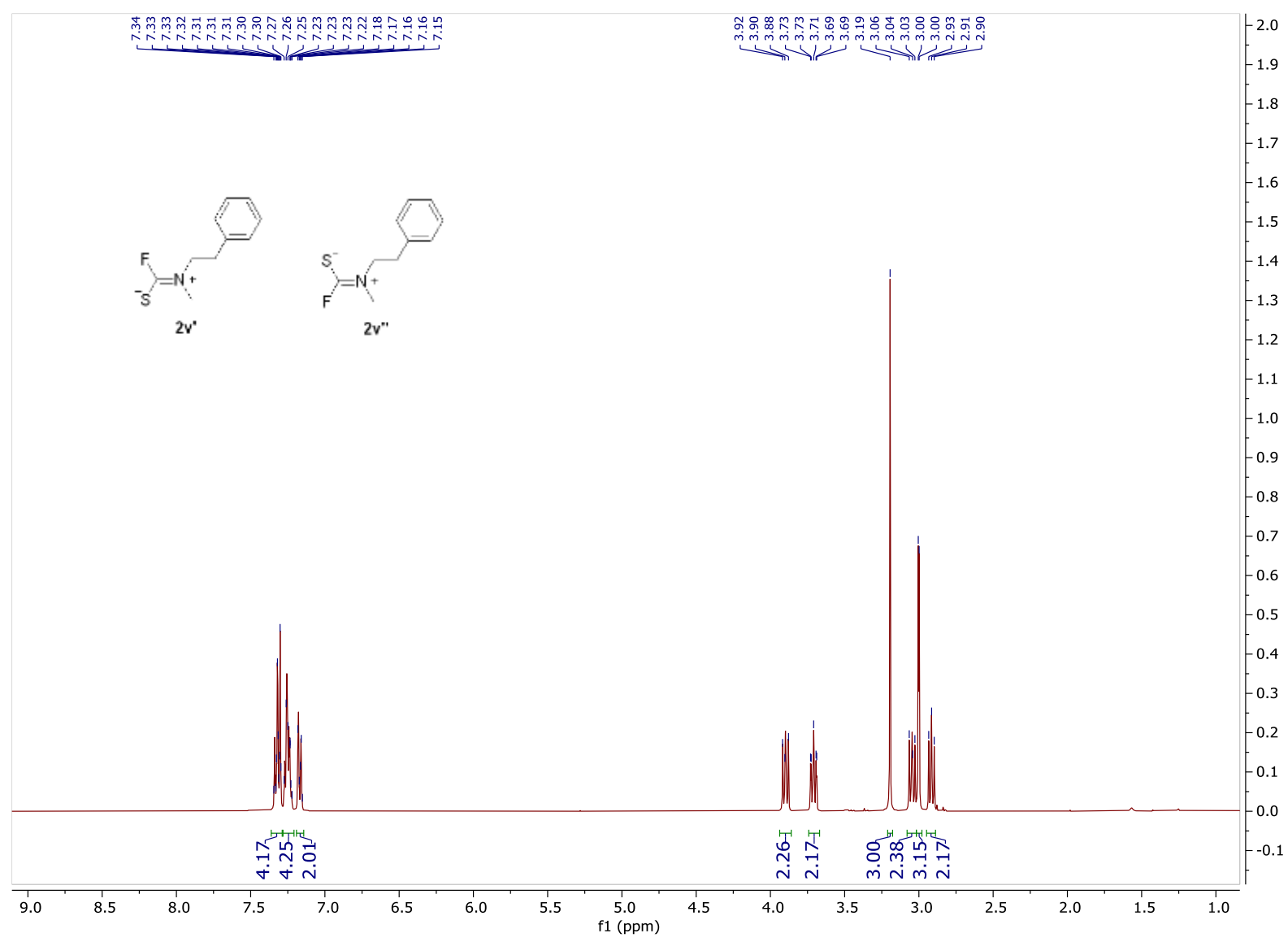

$^{1}$H NMR (400 MHz, $CDCl_3$) of **2v'** and **2v''.**

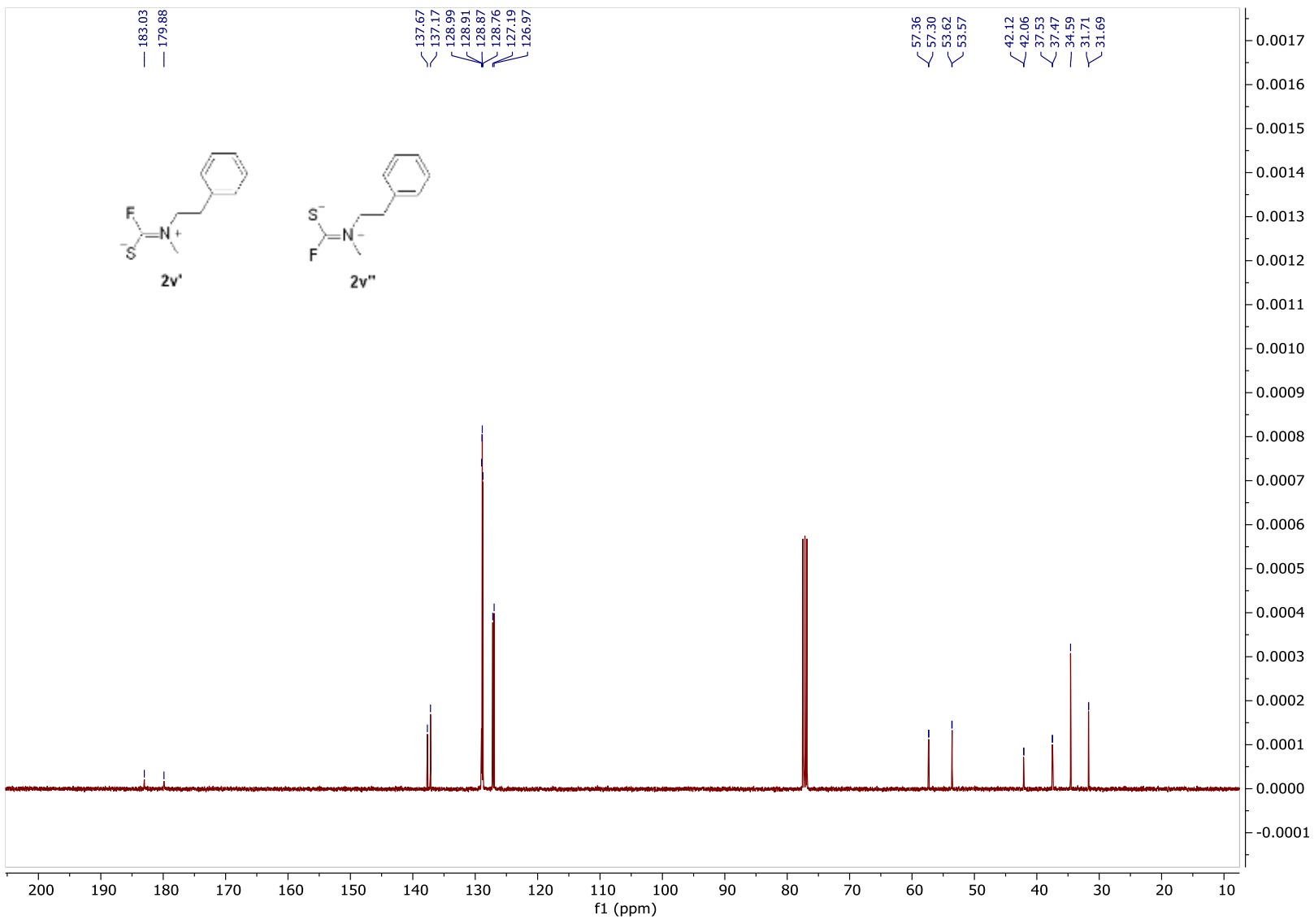

$^{13}$C NMR (101 MHz, CDCl$_3$) of **2v'** and **2v''.**

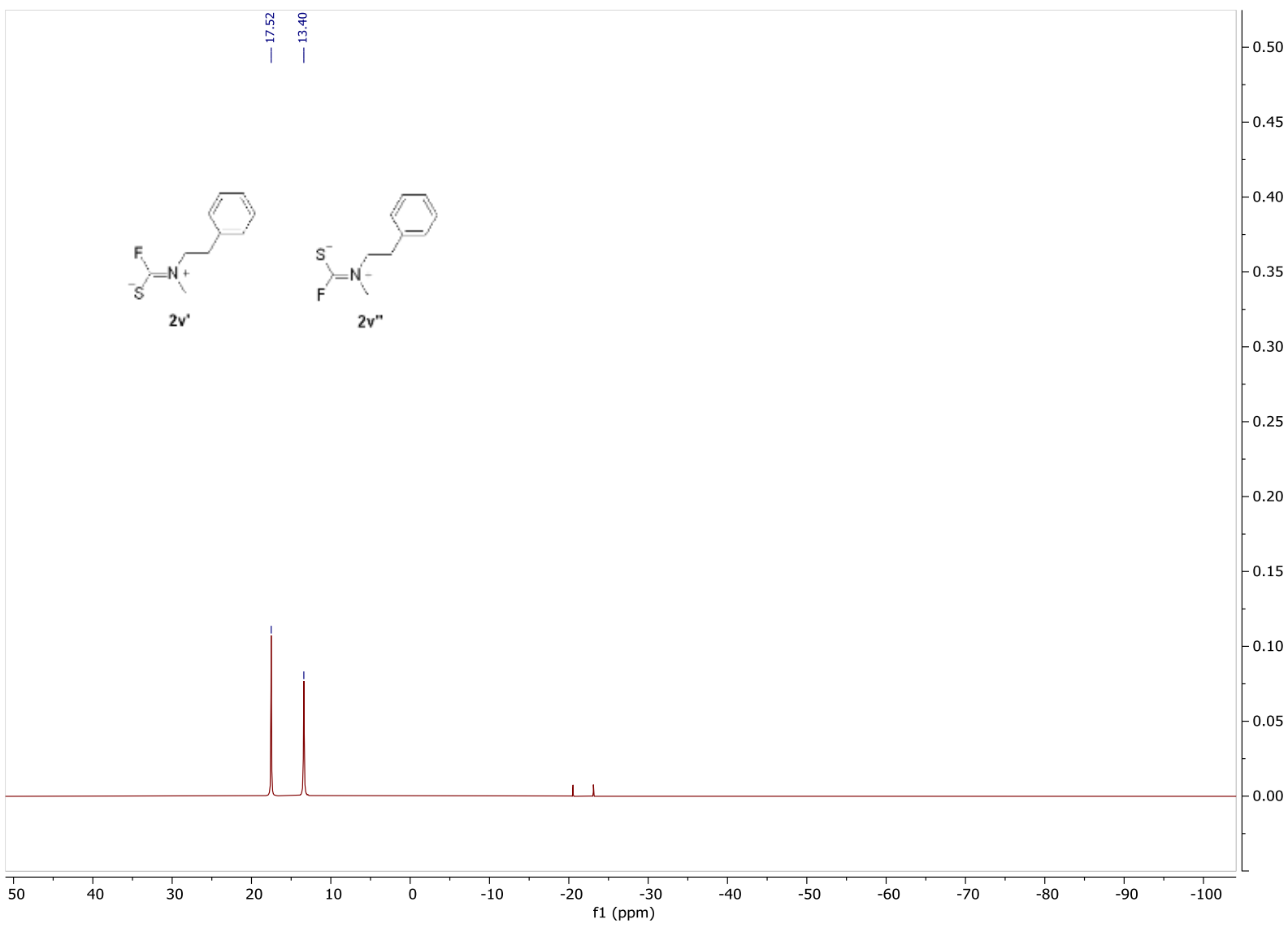

$^{19}$F NMR (367 MHz, CDCl$_3$) of **2v'** and **2v''.**

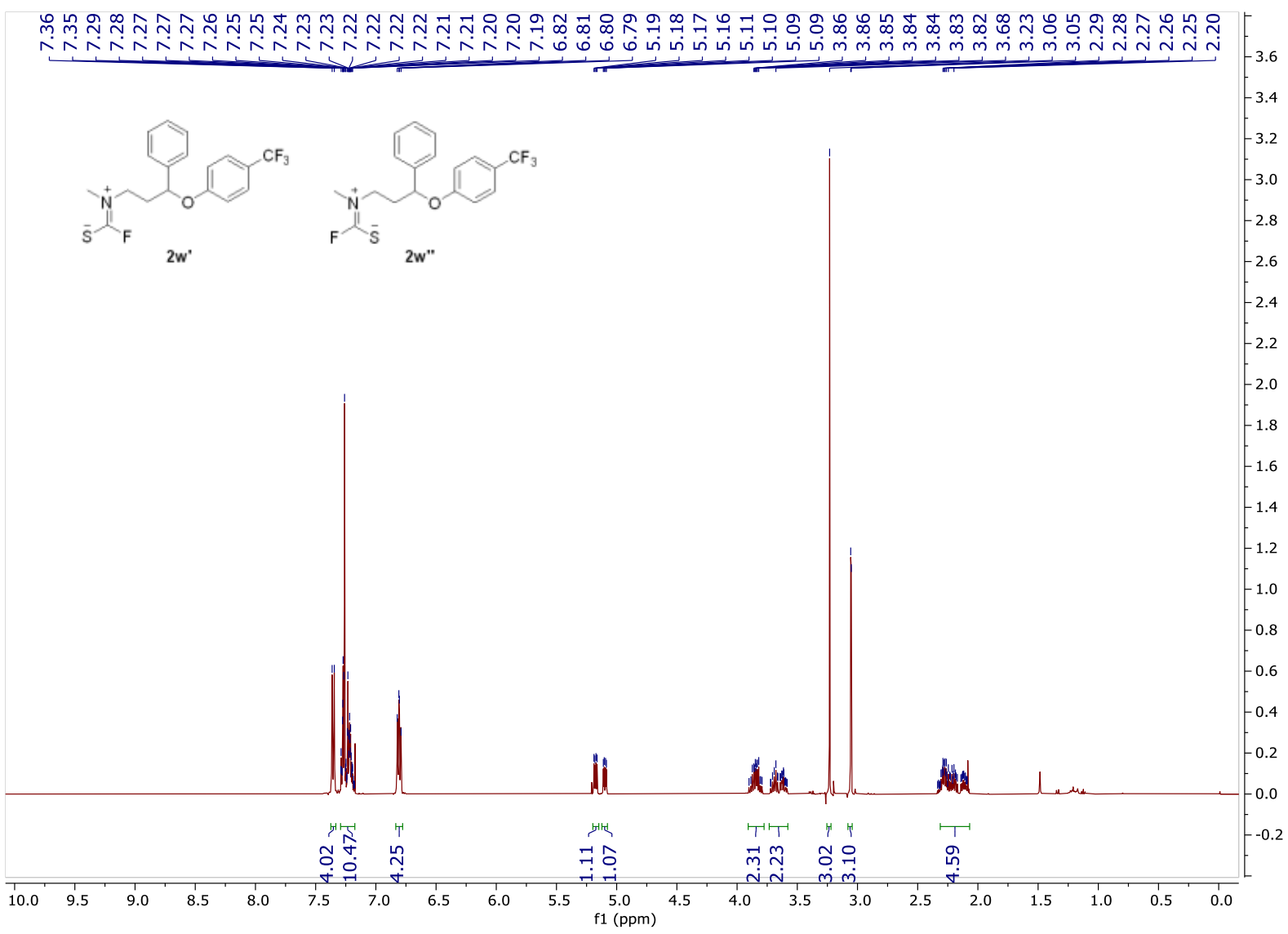


$^{1}$H NMR (400 MHz, $CDCl_3$) of **2w' and 2w''.**

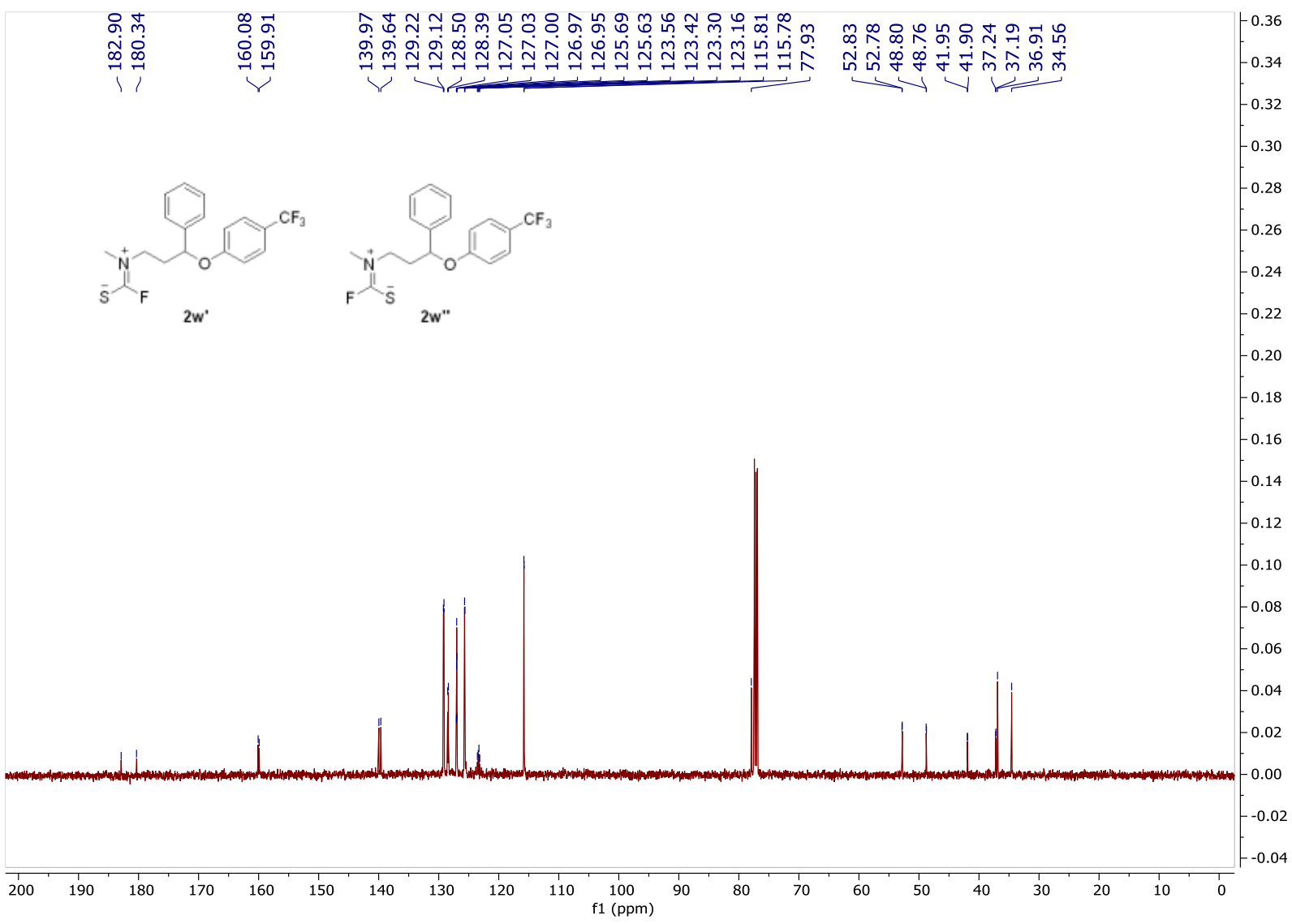


$^{13}$C NMR (101 MHz, $CDCl_3$) of **2w' and 2w''.**

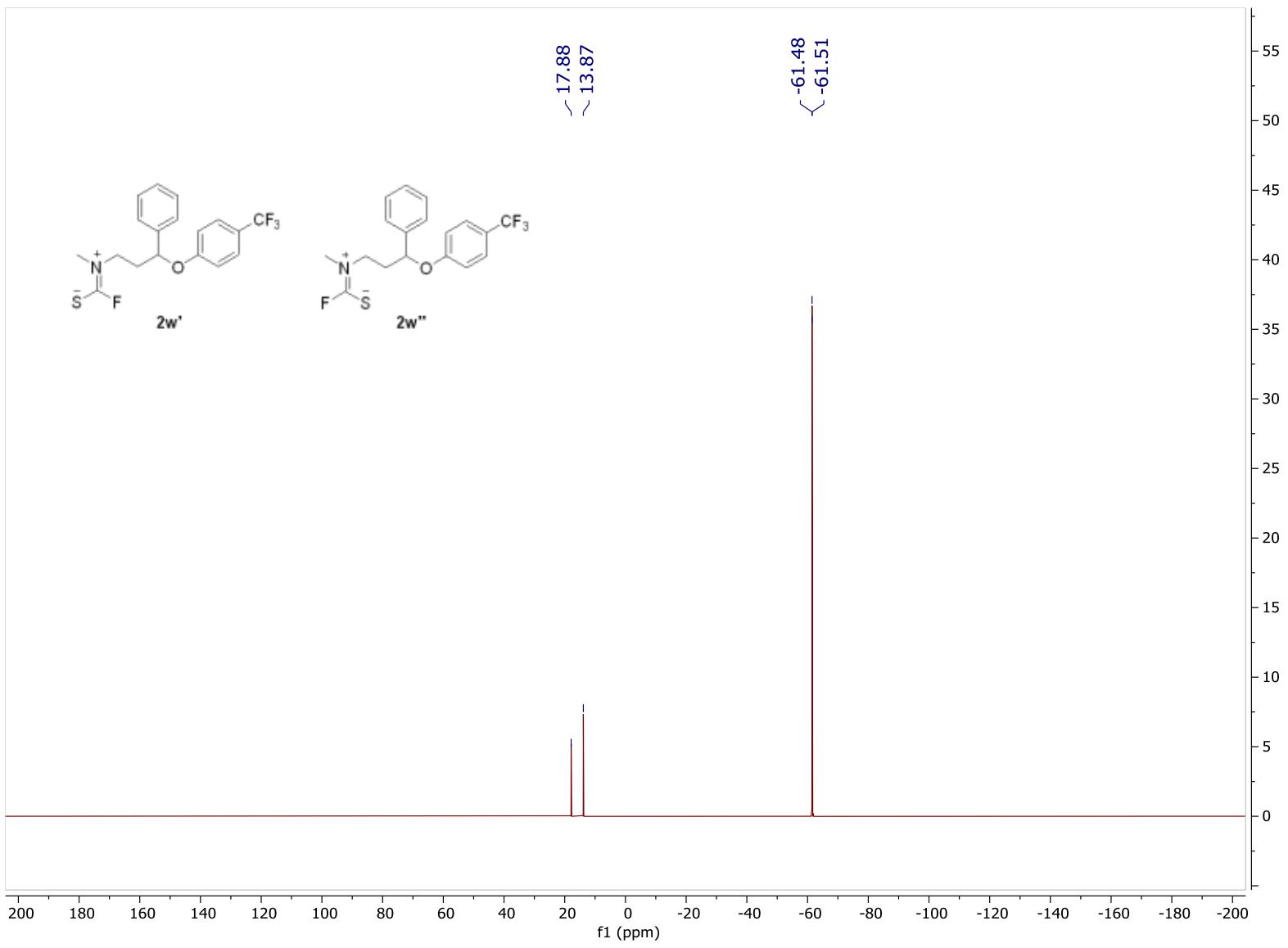

$^{19}$F NMR (367 MHz, $CDCl_3$) of **2w’** and **2w”.**

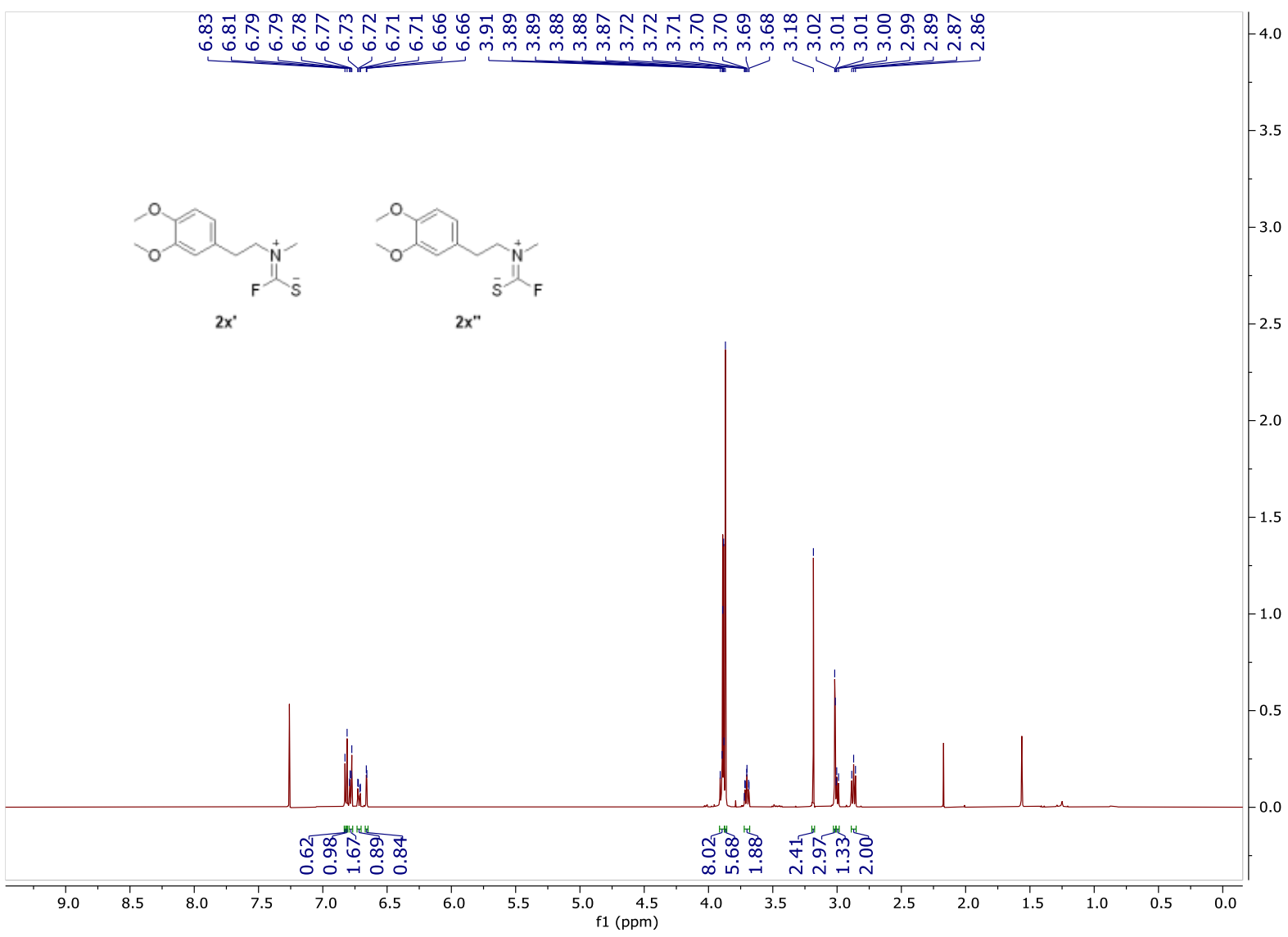

$^{1}$H NMR (400 MHz, $CDCl_3$) of **2x’** and **2x”.**

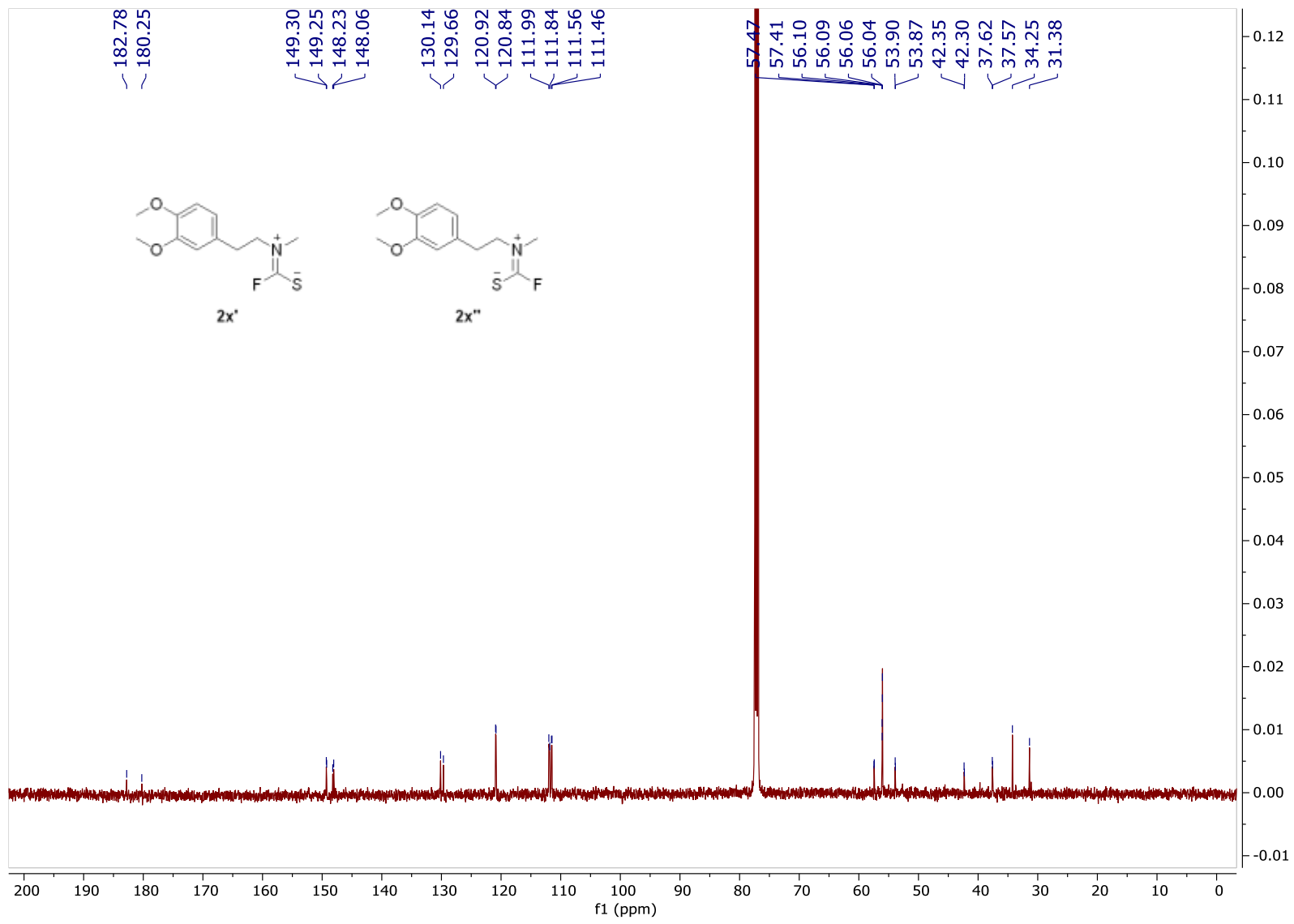

$^{13}$C NMR (101 MHz, $CDCl_3$) of **2x'** and **2x''.**

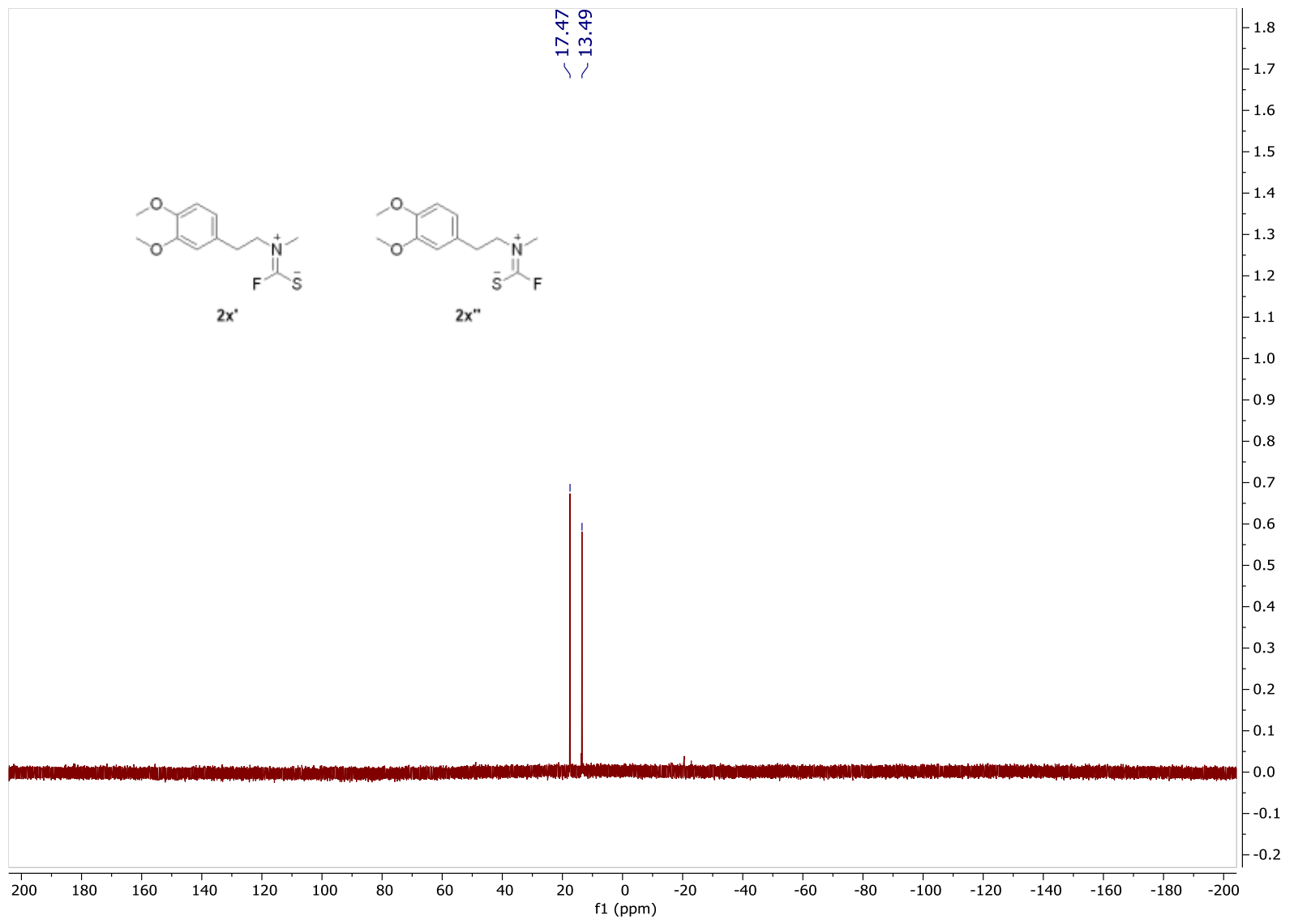

$^{19}$F NMR (367 MHz, $CDCl_3$) of **2x'** and **2x''.**

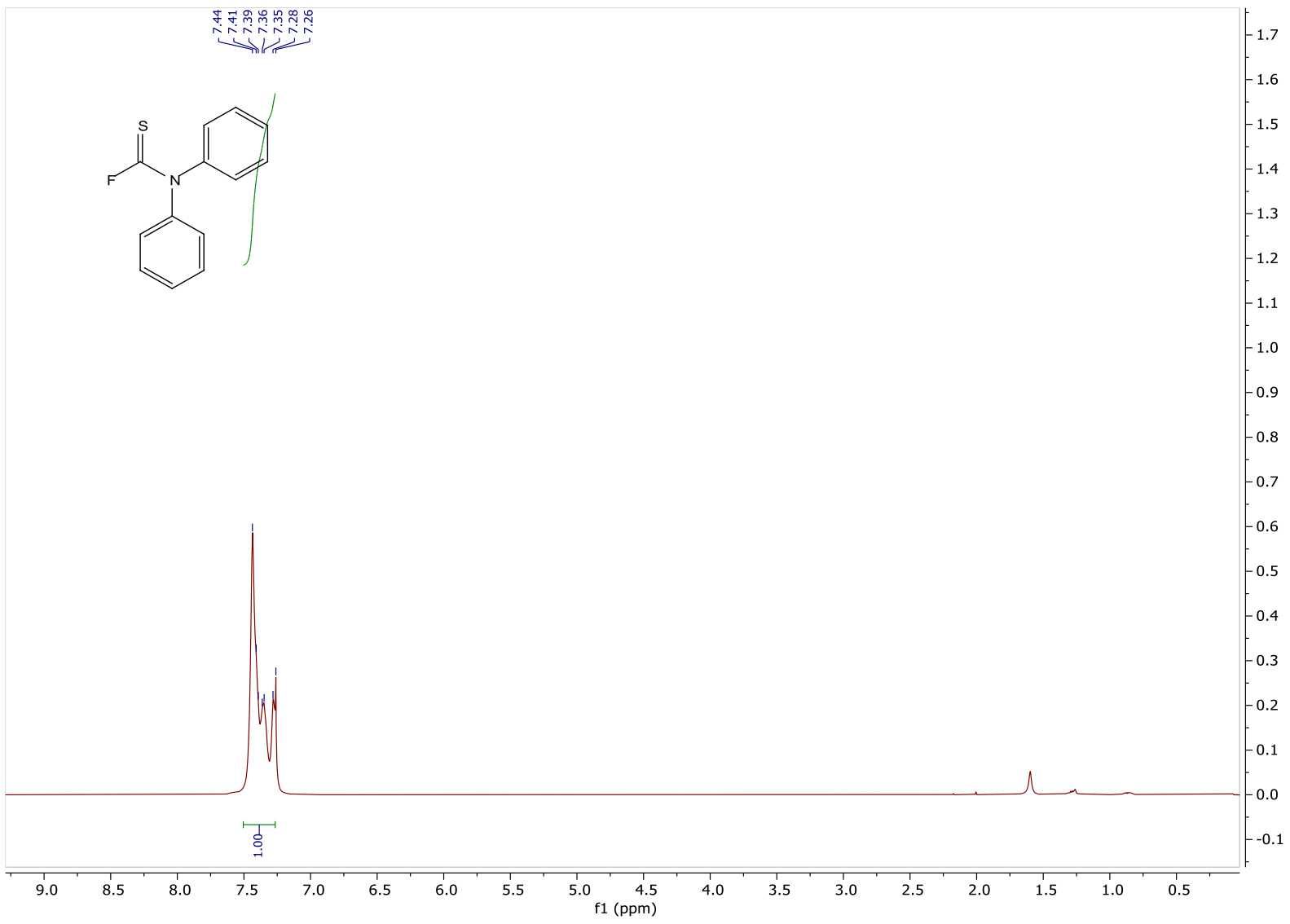

$^{1}$H NMR (500 MHz, CDCl3) of **2y.**

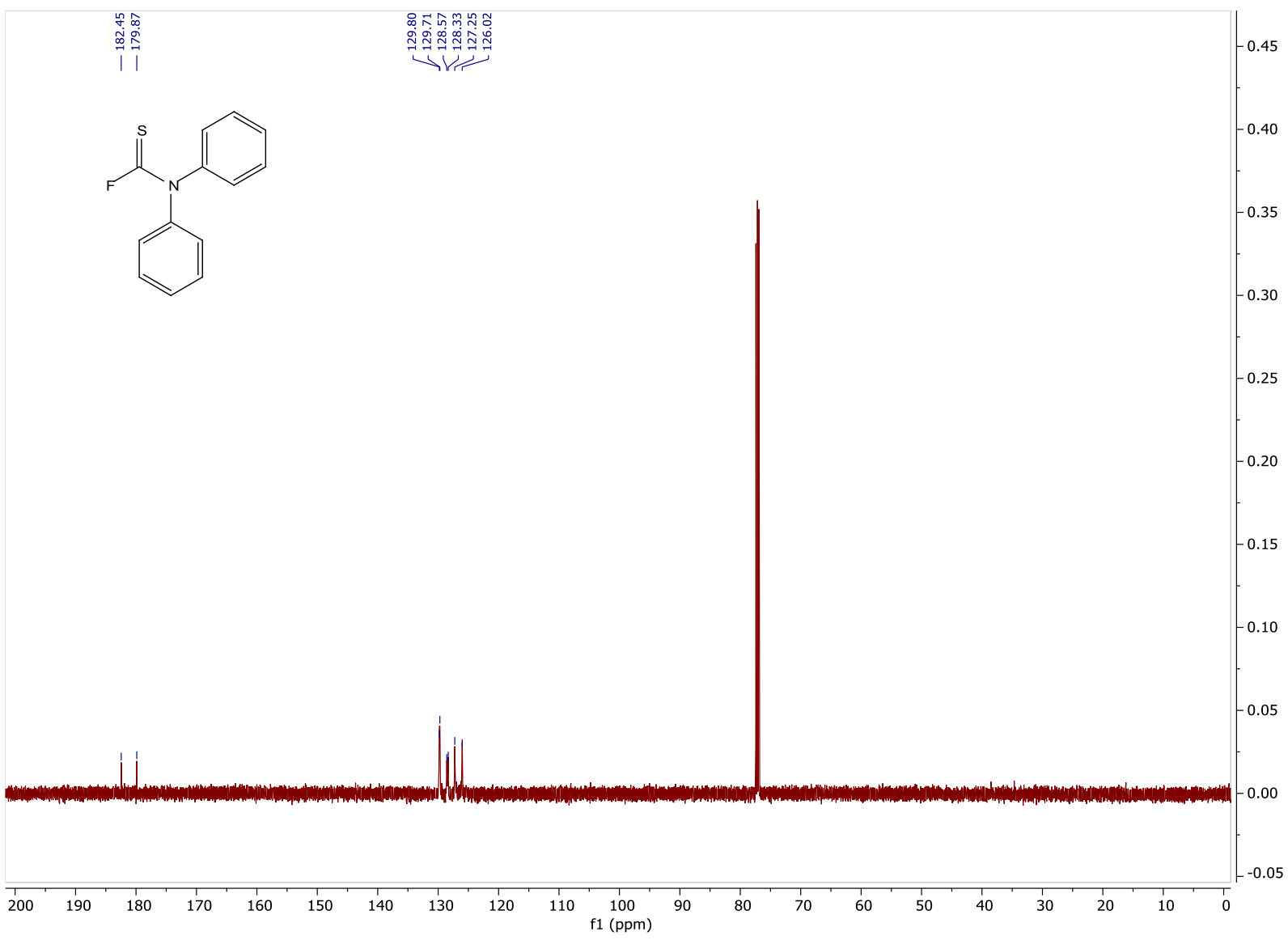

$^{13}$C NMR (101 MHz, CDCl3) of **2y.**

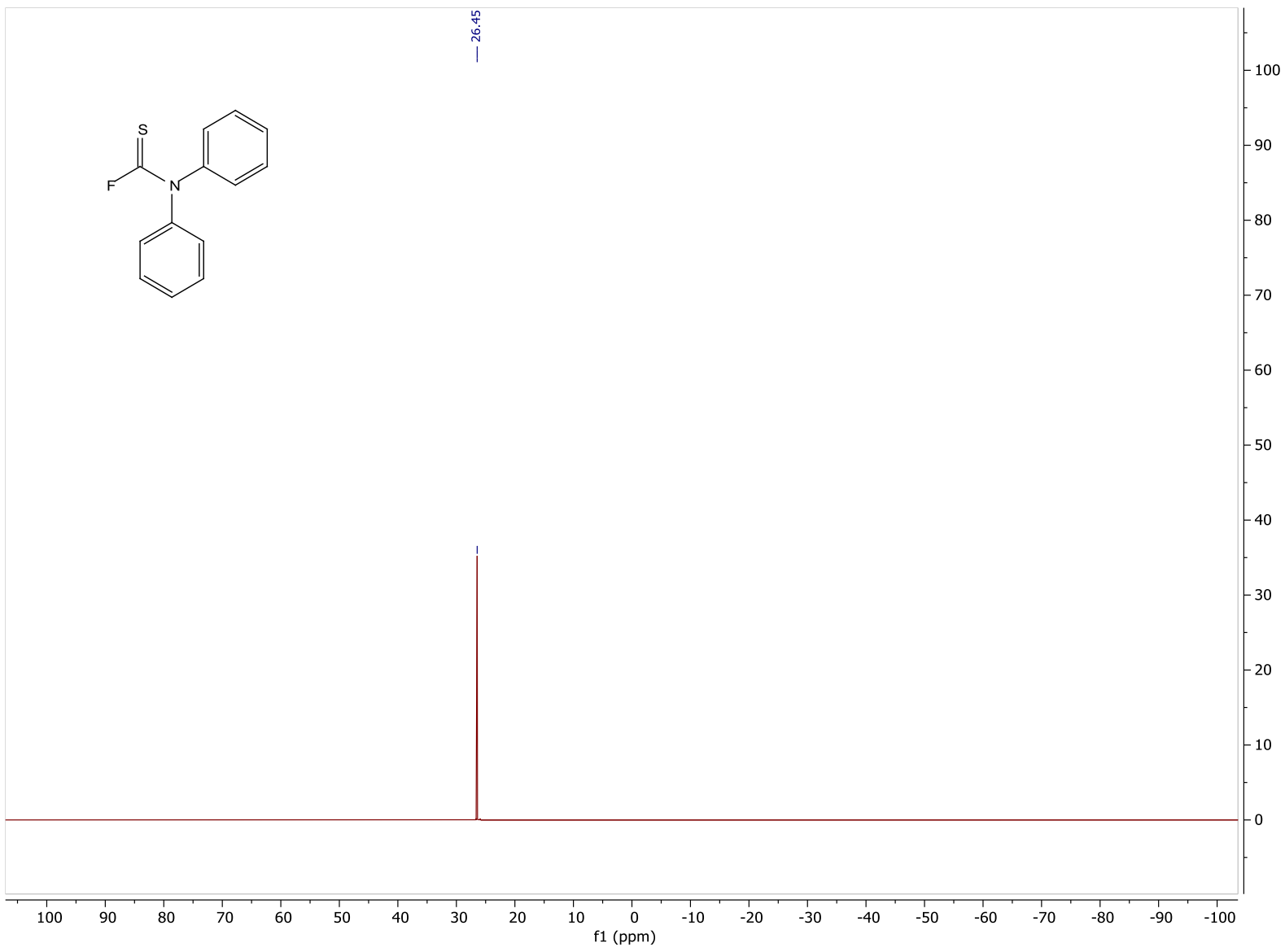


$^{19}F$ NMR (367 MHz, $CDCl_3$) of **2y.**

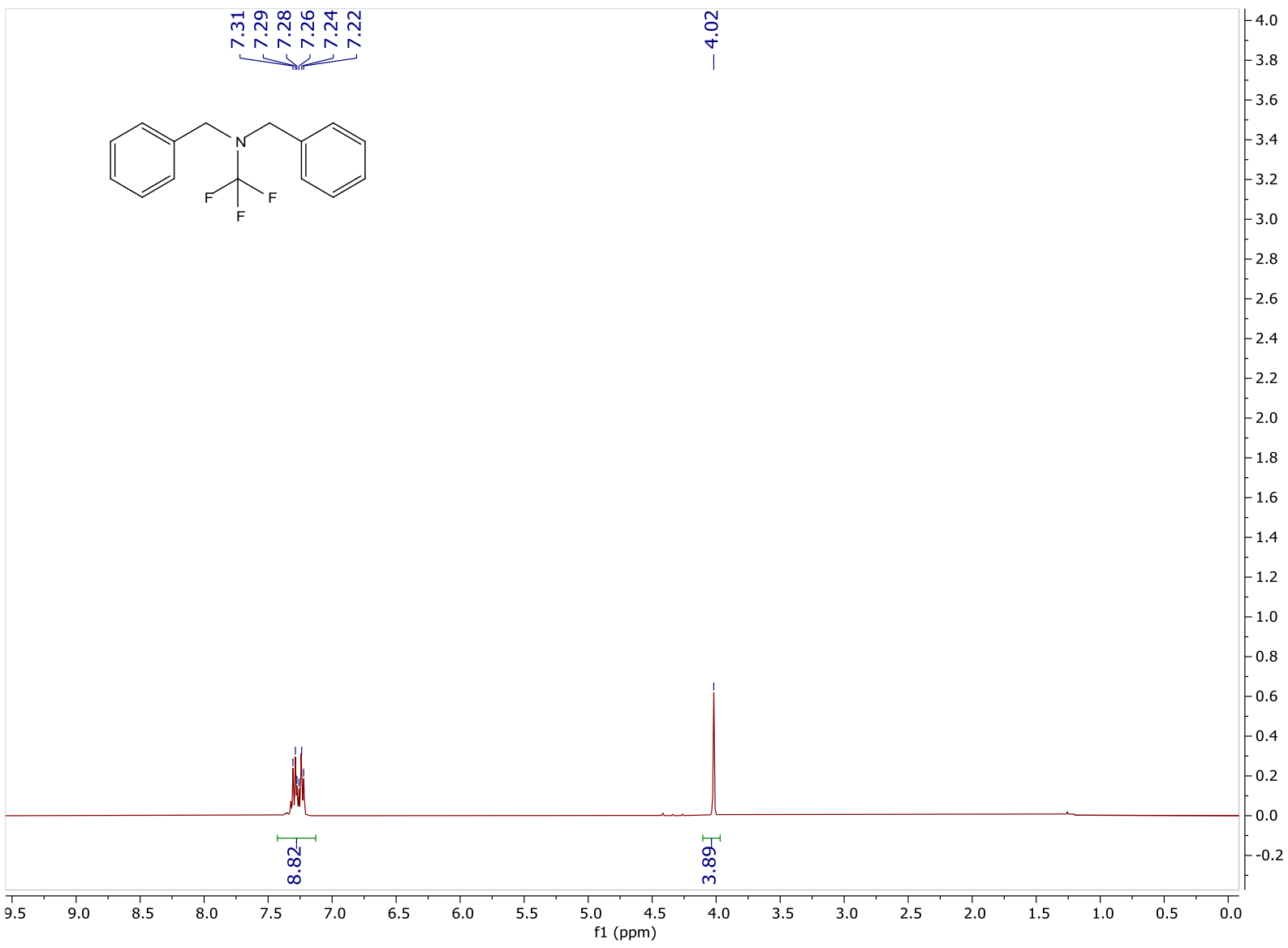


$^{1}H$ NMR (400 MHz, $CDCl_3$) of **3a**.

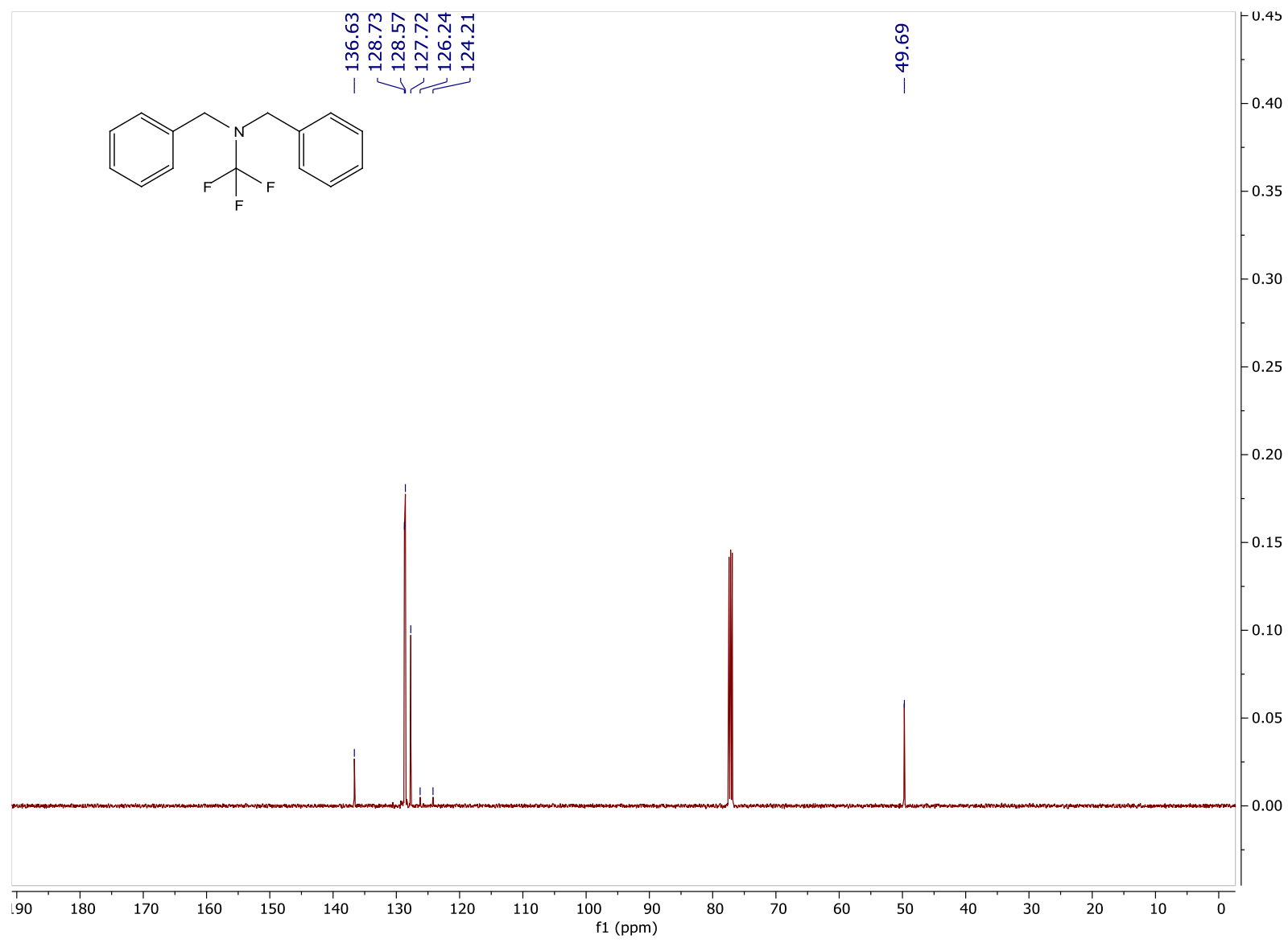


$^{13}$C NMR (126 MHz, $CDCl_3$) of **3a**

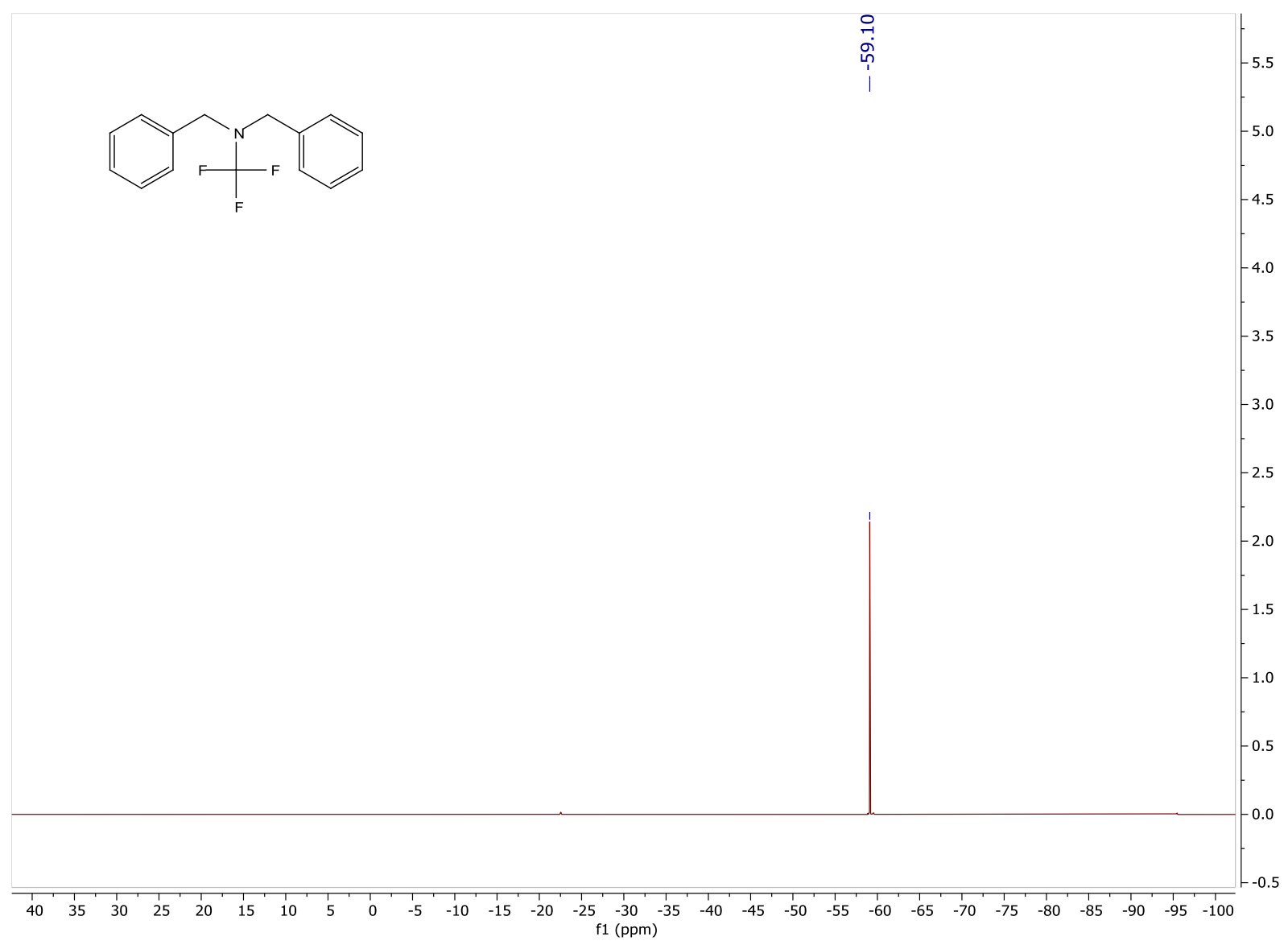


$^{19}$F NMR (376 MHz, $CDCl_3$) of **3a**

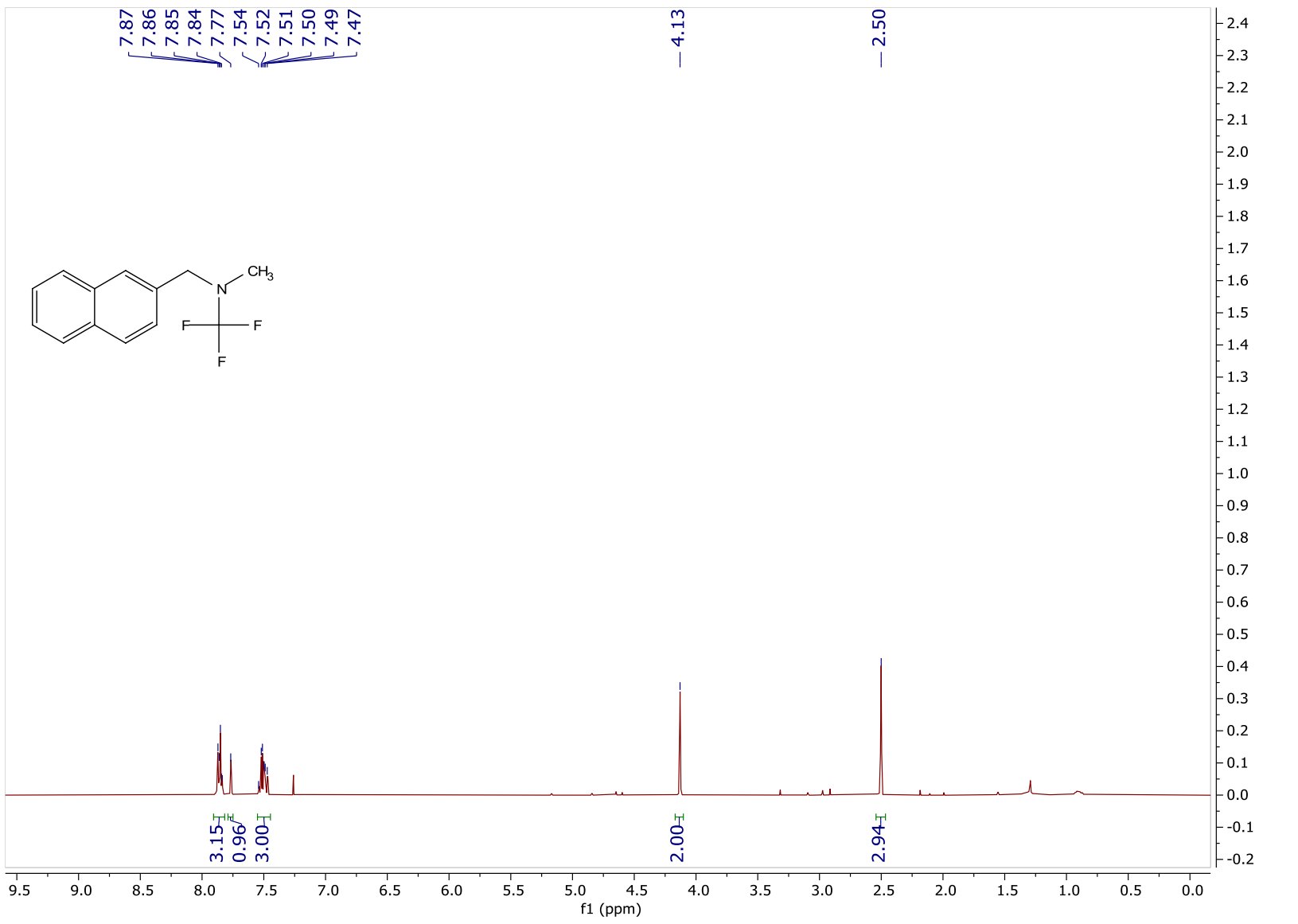

$^{1}$H NMR (400 MHz, $CDCl_3$) of **3b.**

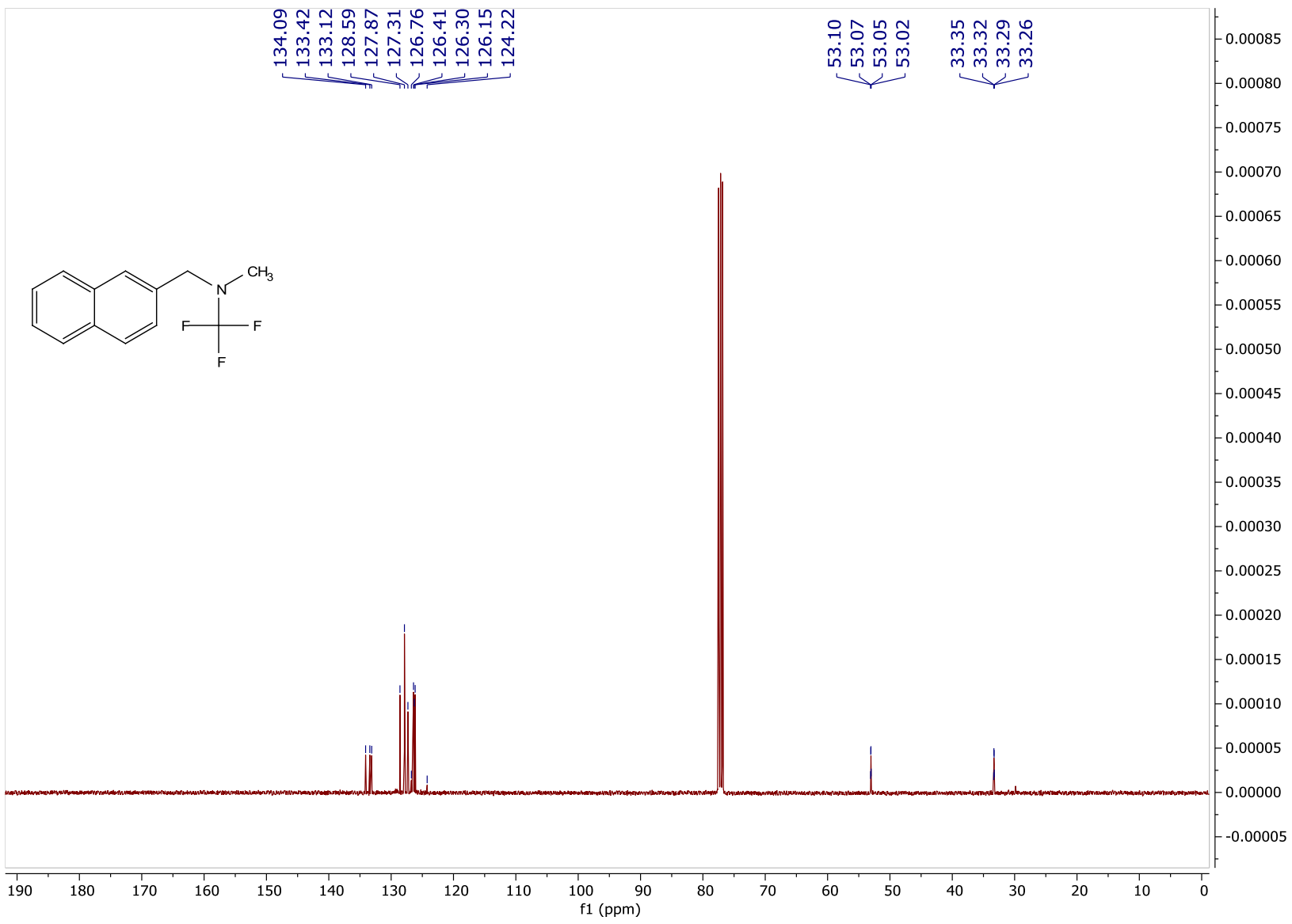

$^{13}$C NMR (101 MHz, $CDCl_3$) of **3b.**

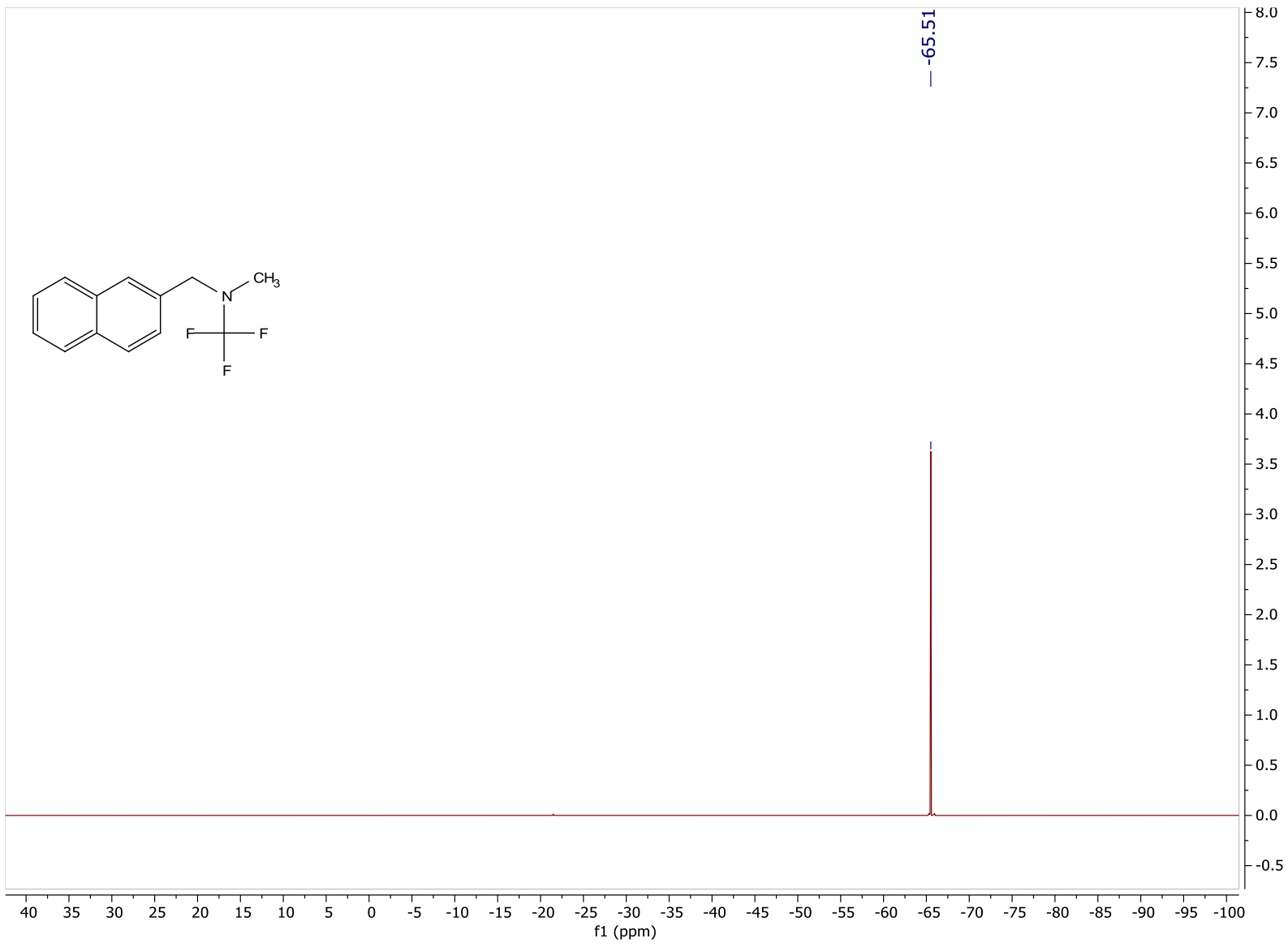


$^{19}$F NMR (376 MHz, $CDCl_3$) of **3b.**

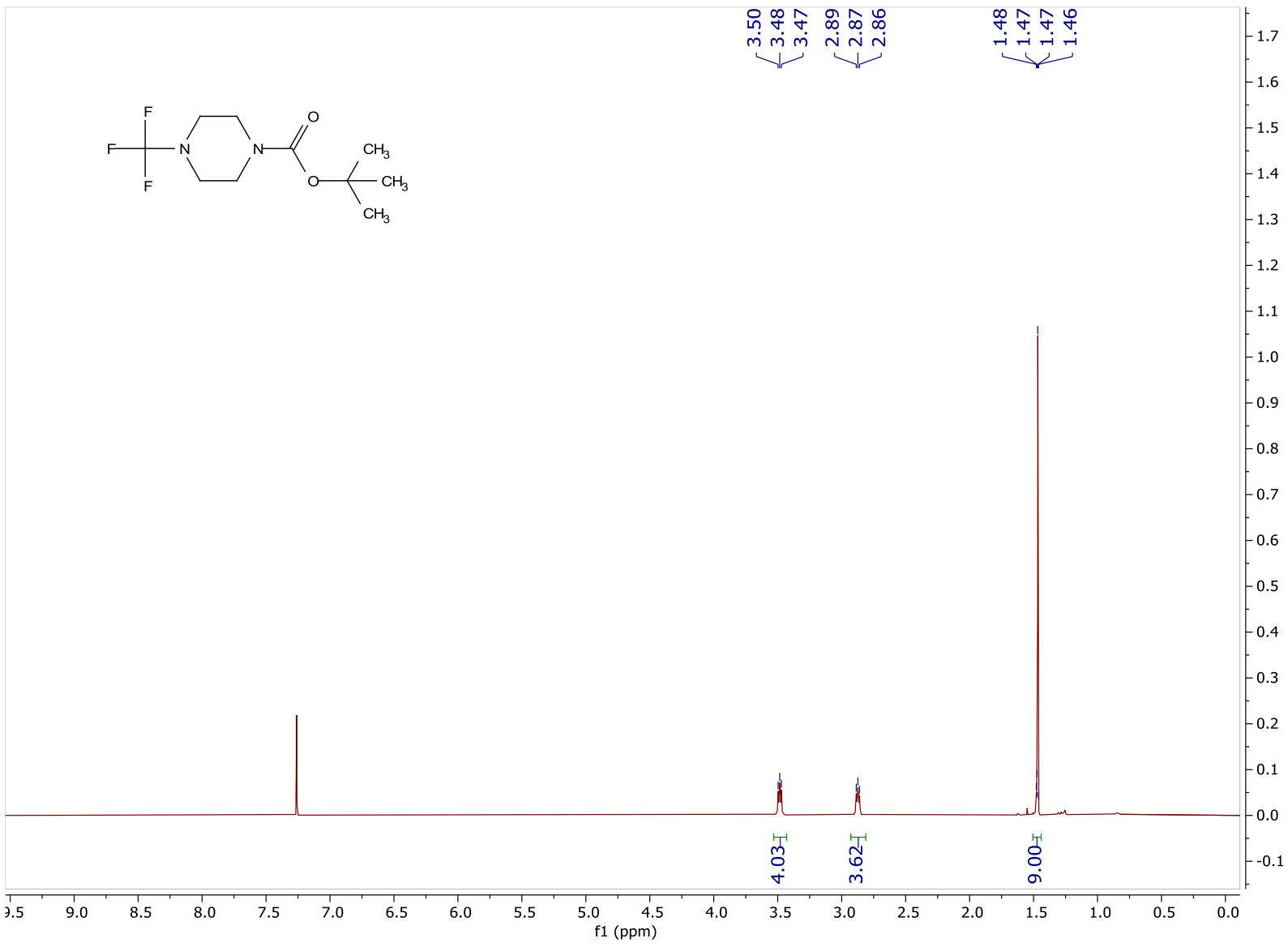


$^{1}$H NMR (400 MHz, $CDCl_3$) of **3c.**

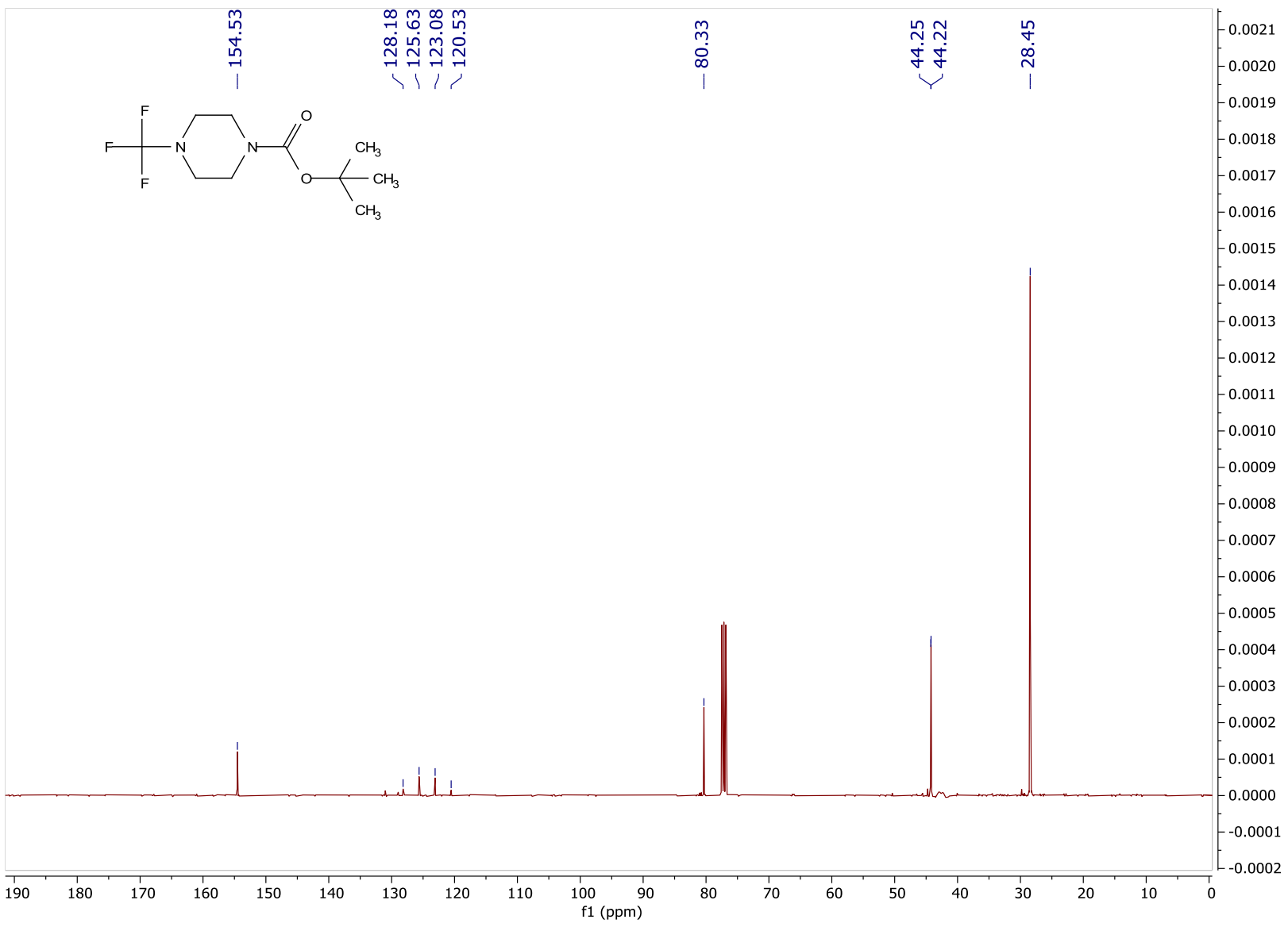


$^{13}$C NMR (101 MHz, $CDCl_3$) of **3c.**

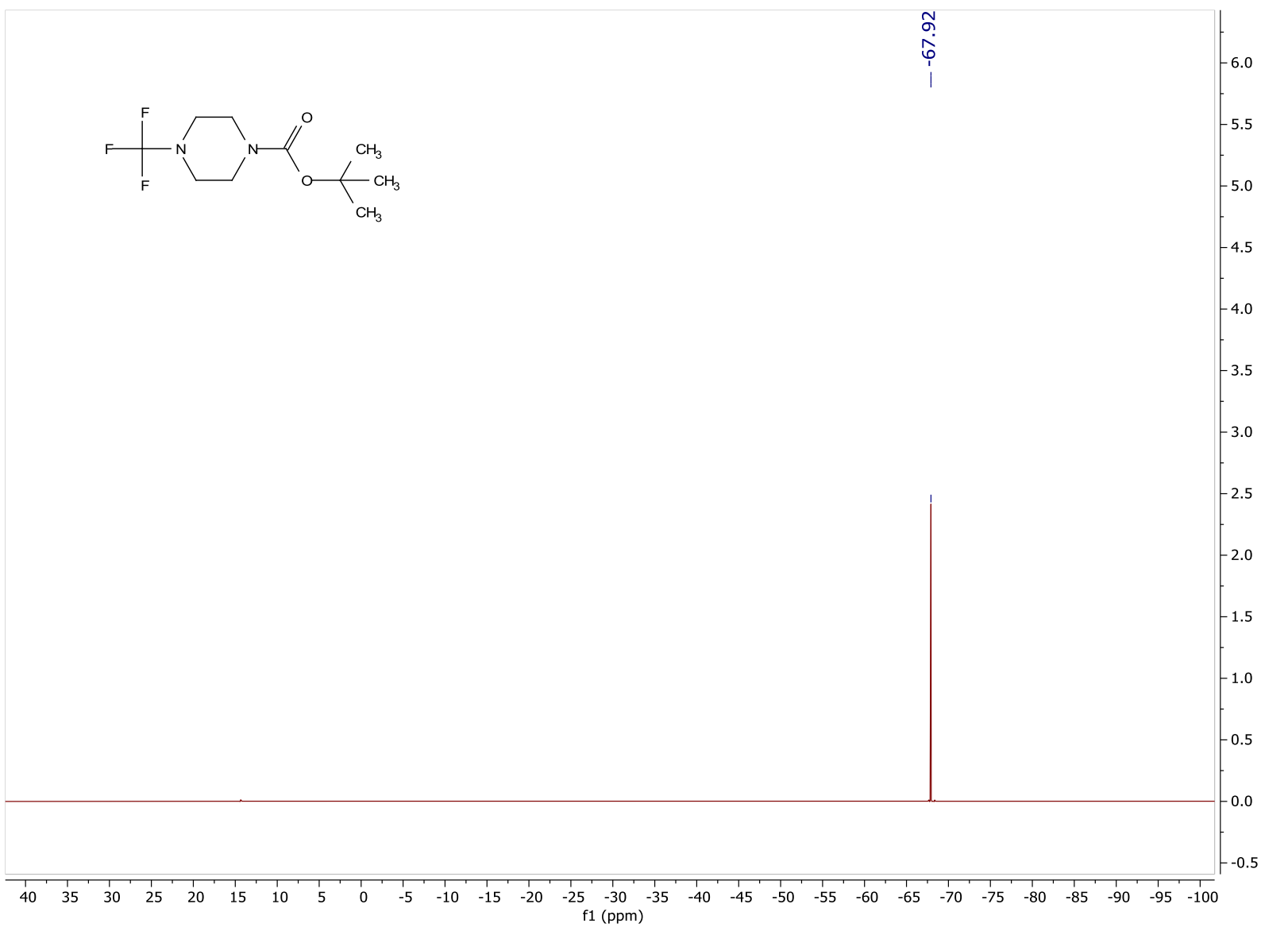


$^{19}$F NMR (376 MHz, $CDCl_3$) of **3c.**

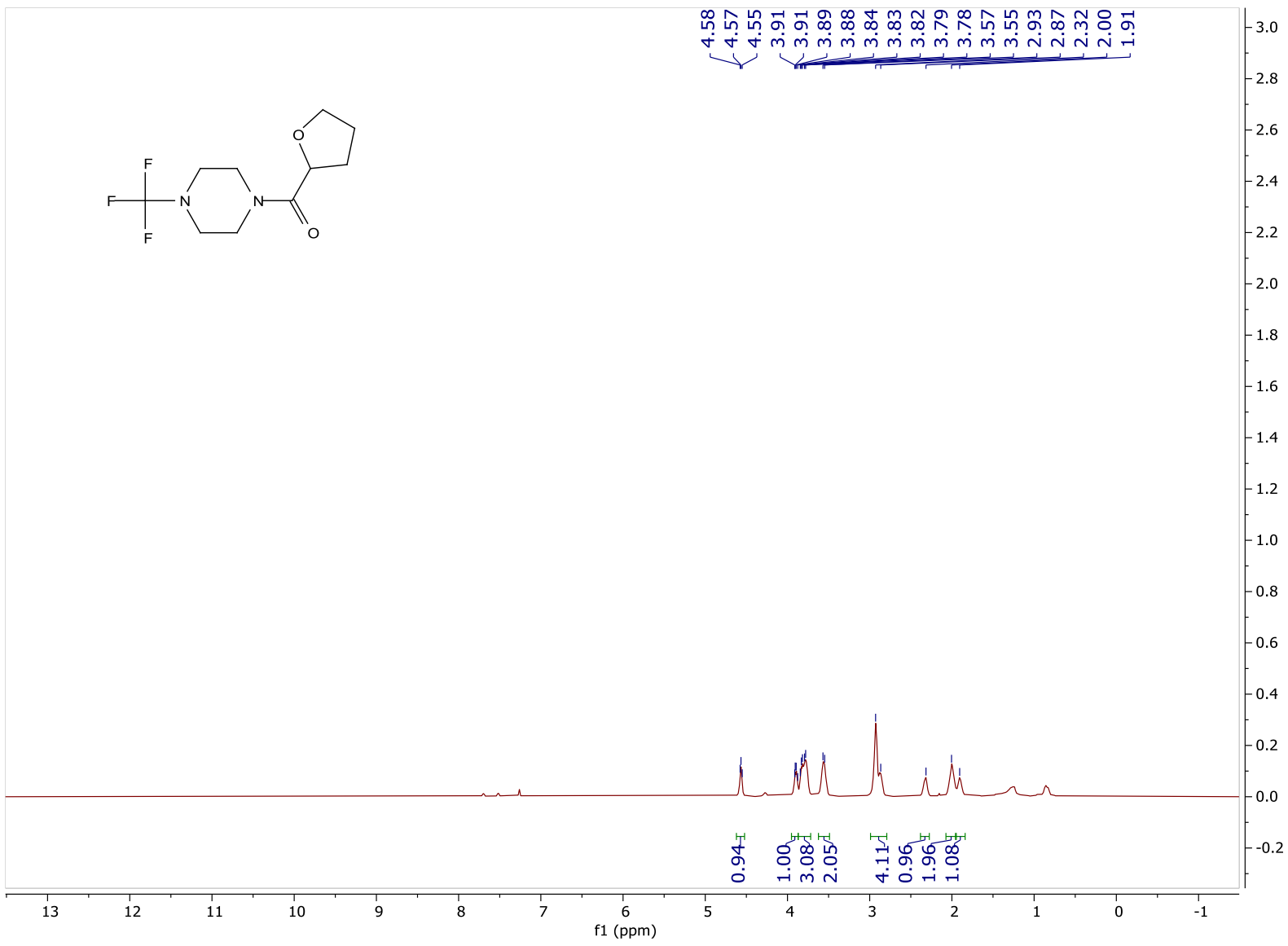

$^{1}H$ NMR (500 MHz, $CDCl_3$) of **3d.**

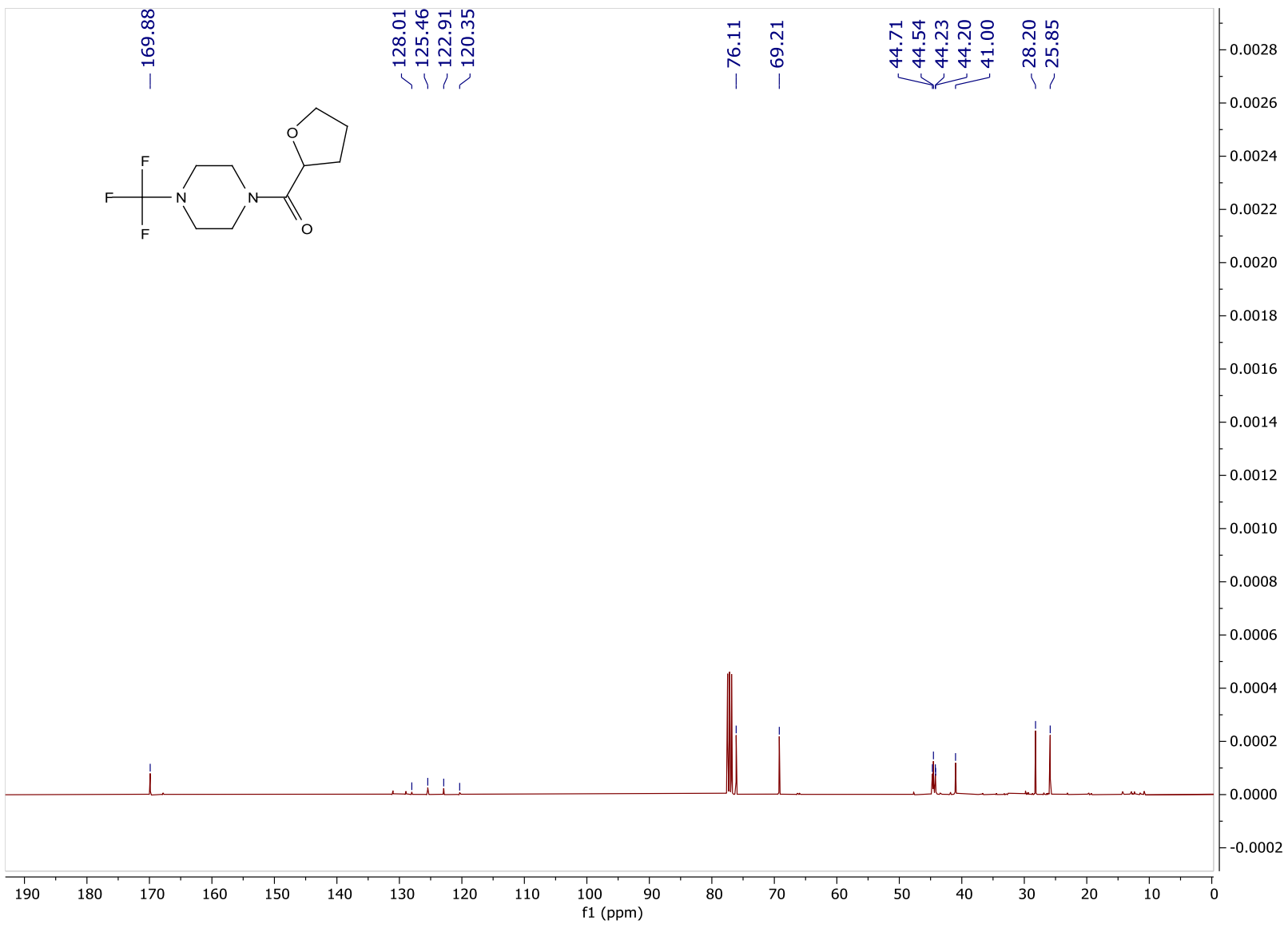

$^{13}C$ NMR (101 MHz, $CDCl_3$) of **3d.**

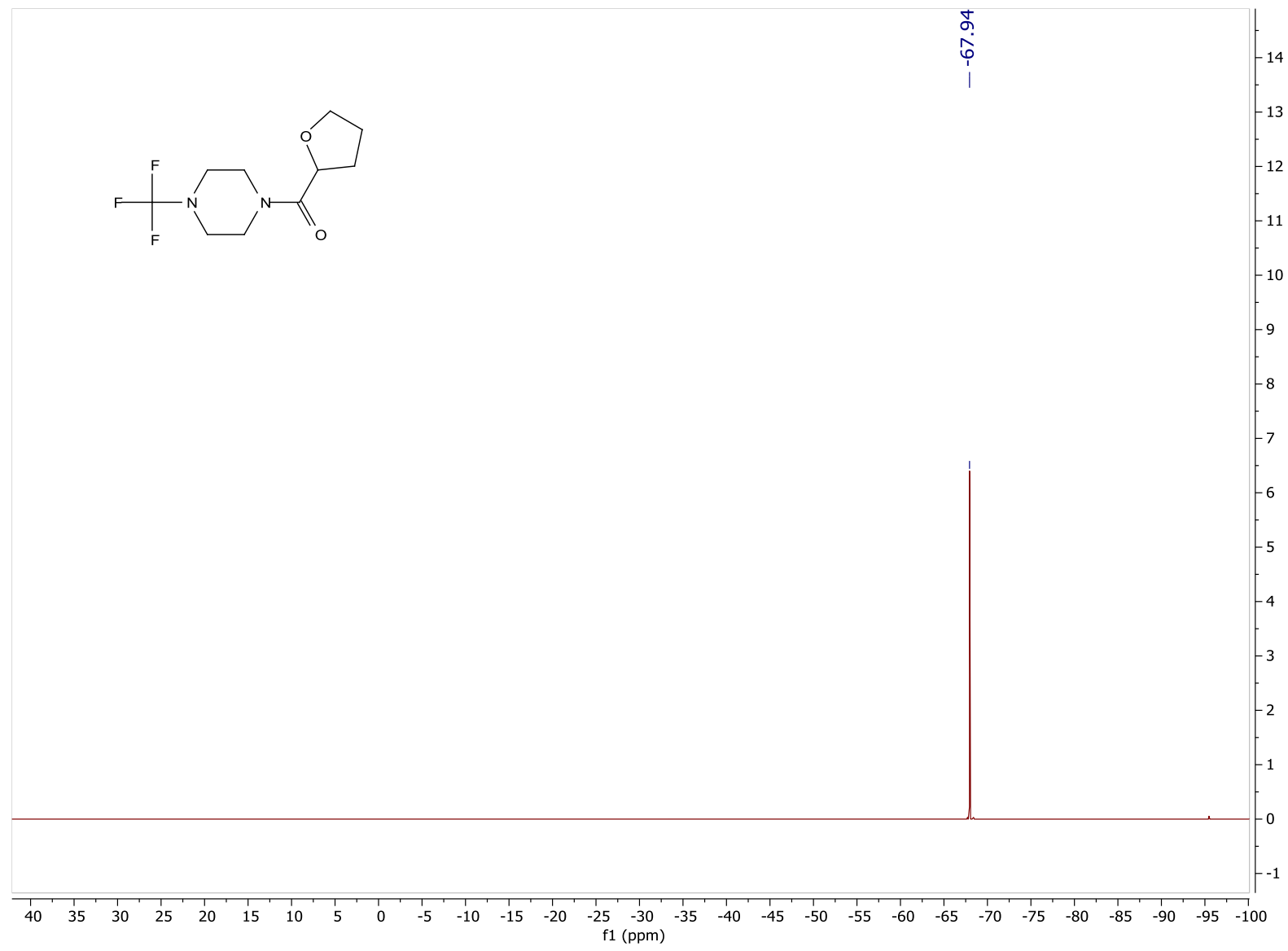


$^{19}$F NMR (376 MHz, $CDCl_3$) δ **3d.**

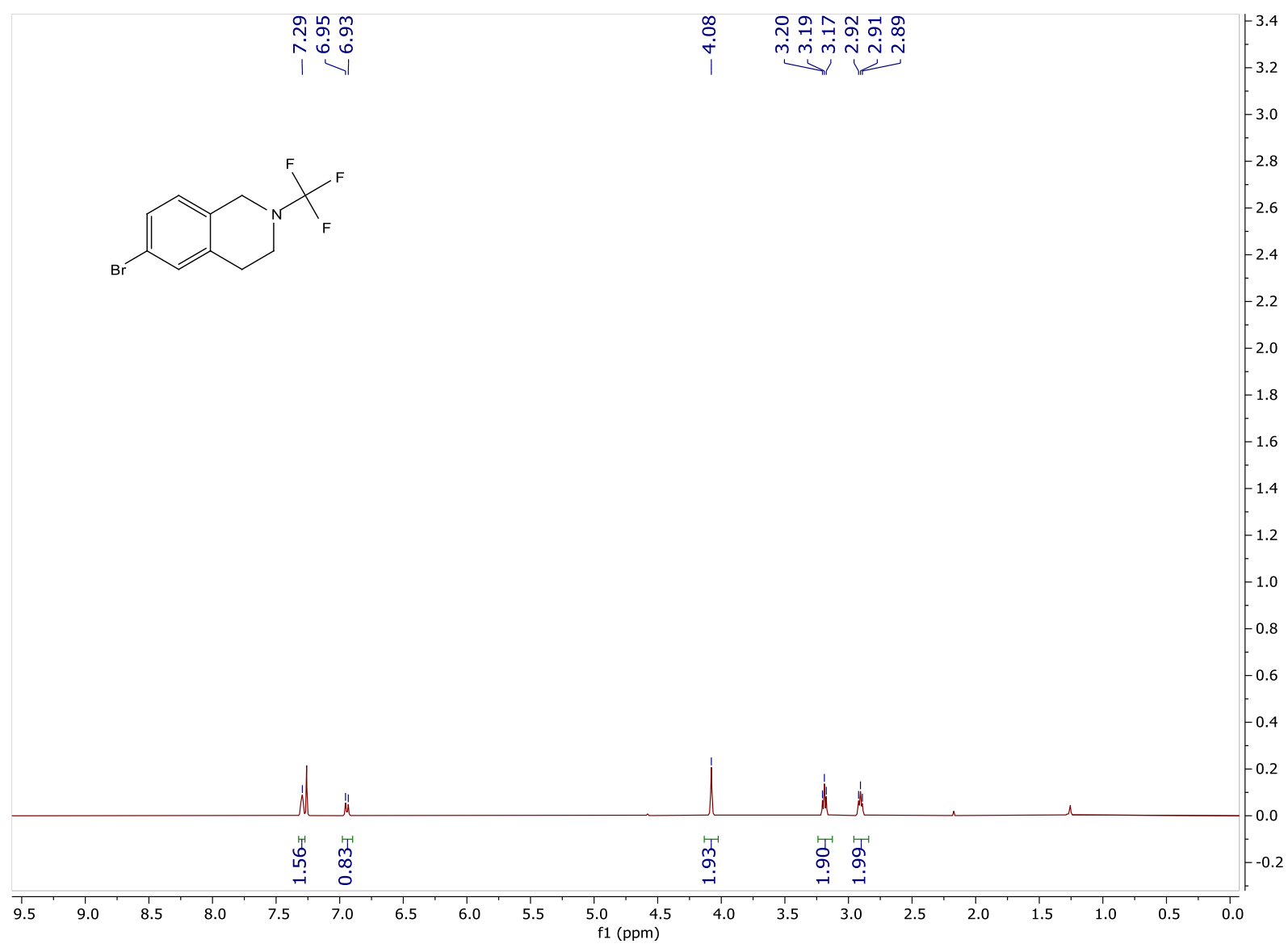


$^{1}$H NMR (400 MHz, $CDCl_3$) of **3e.**

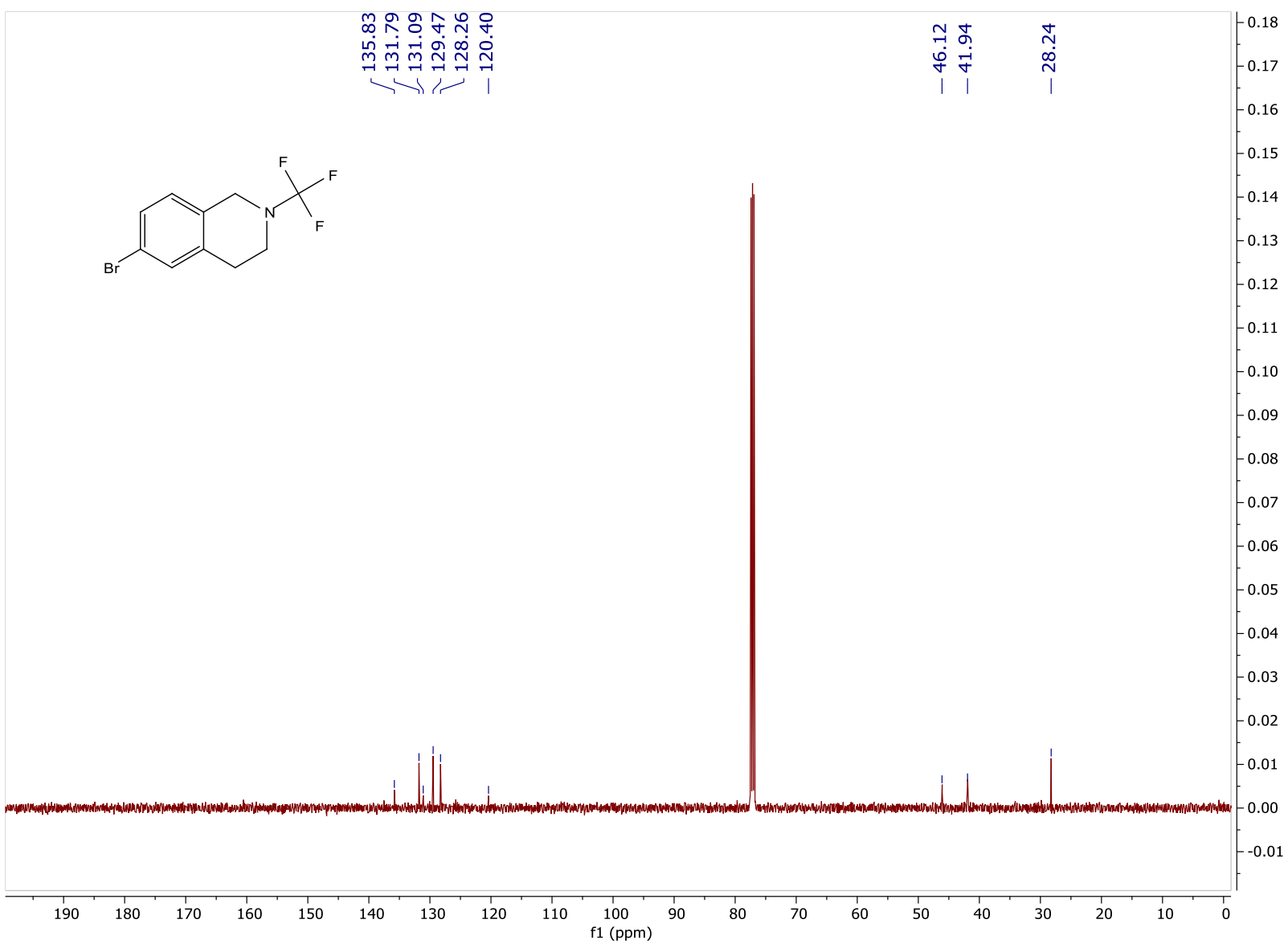


$^{13}$C NMR (126 MHz, $CDCl_3$) of **3e.**

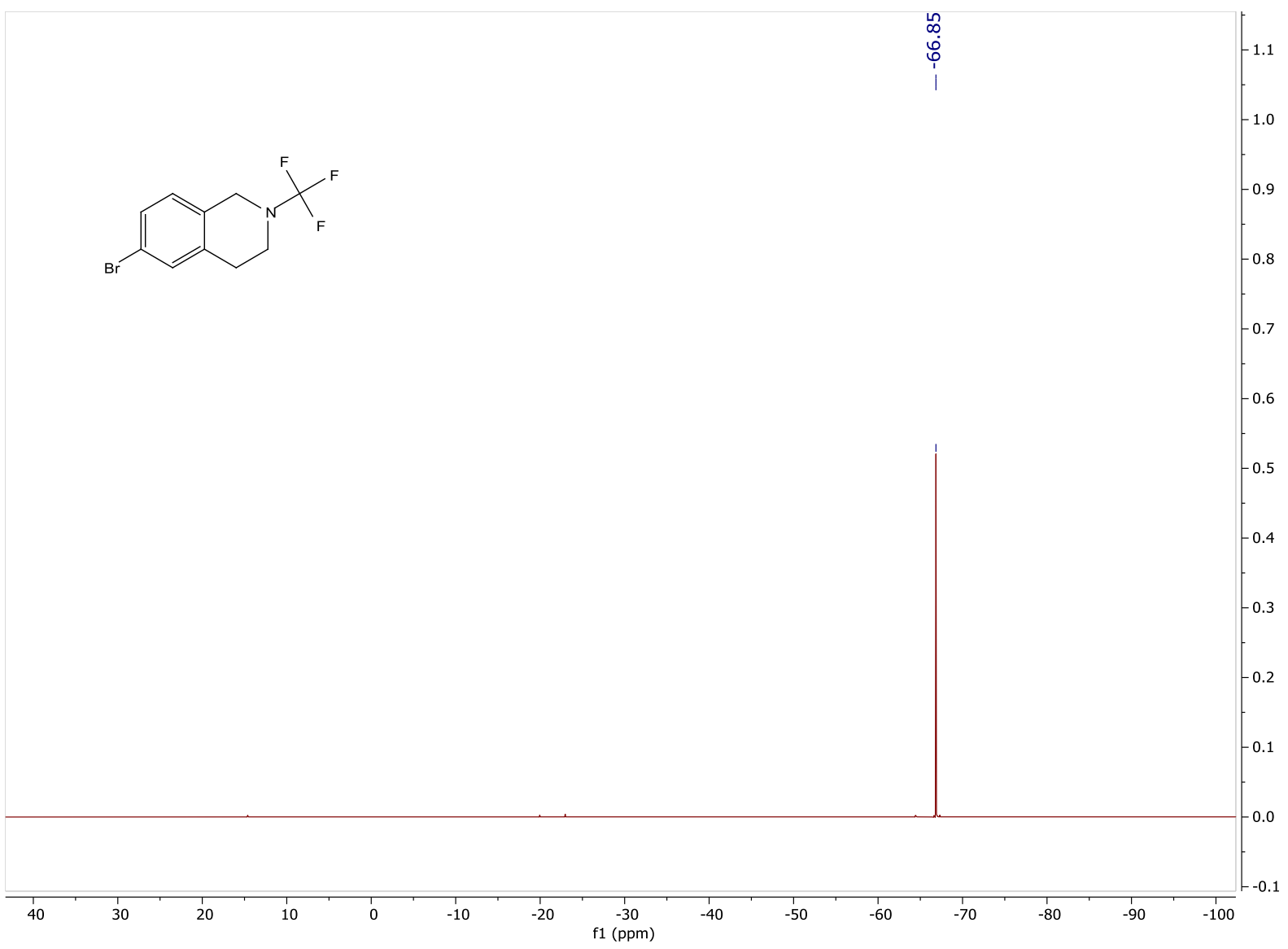


$^{19}$F NMR (376 MHz, $CDCl_3$) of **3e.**

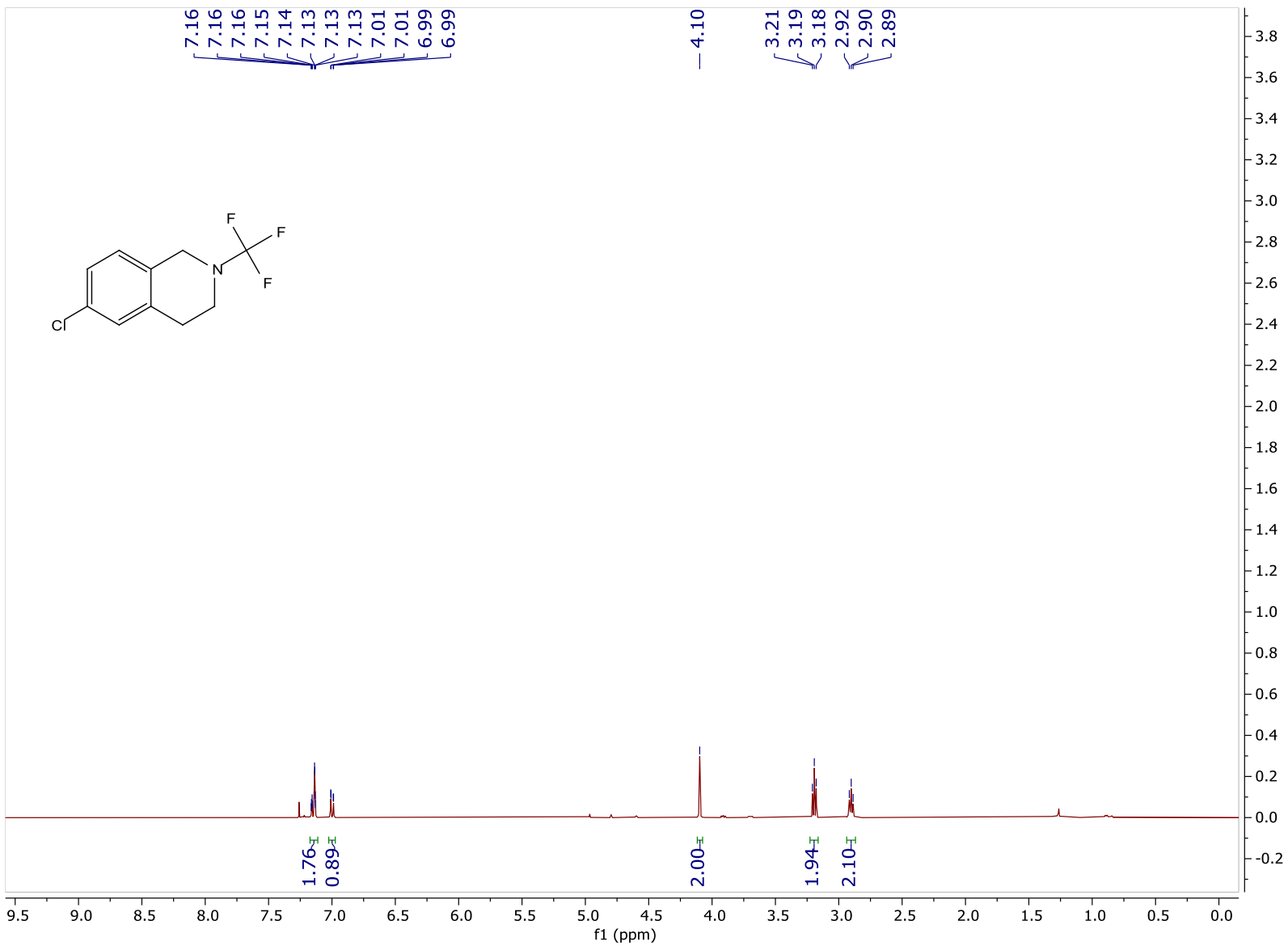

$^{1}$H NMR (400 MHz, $CDCl_3$) of **3f.**

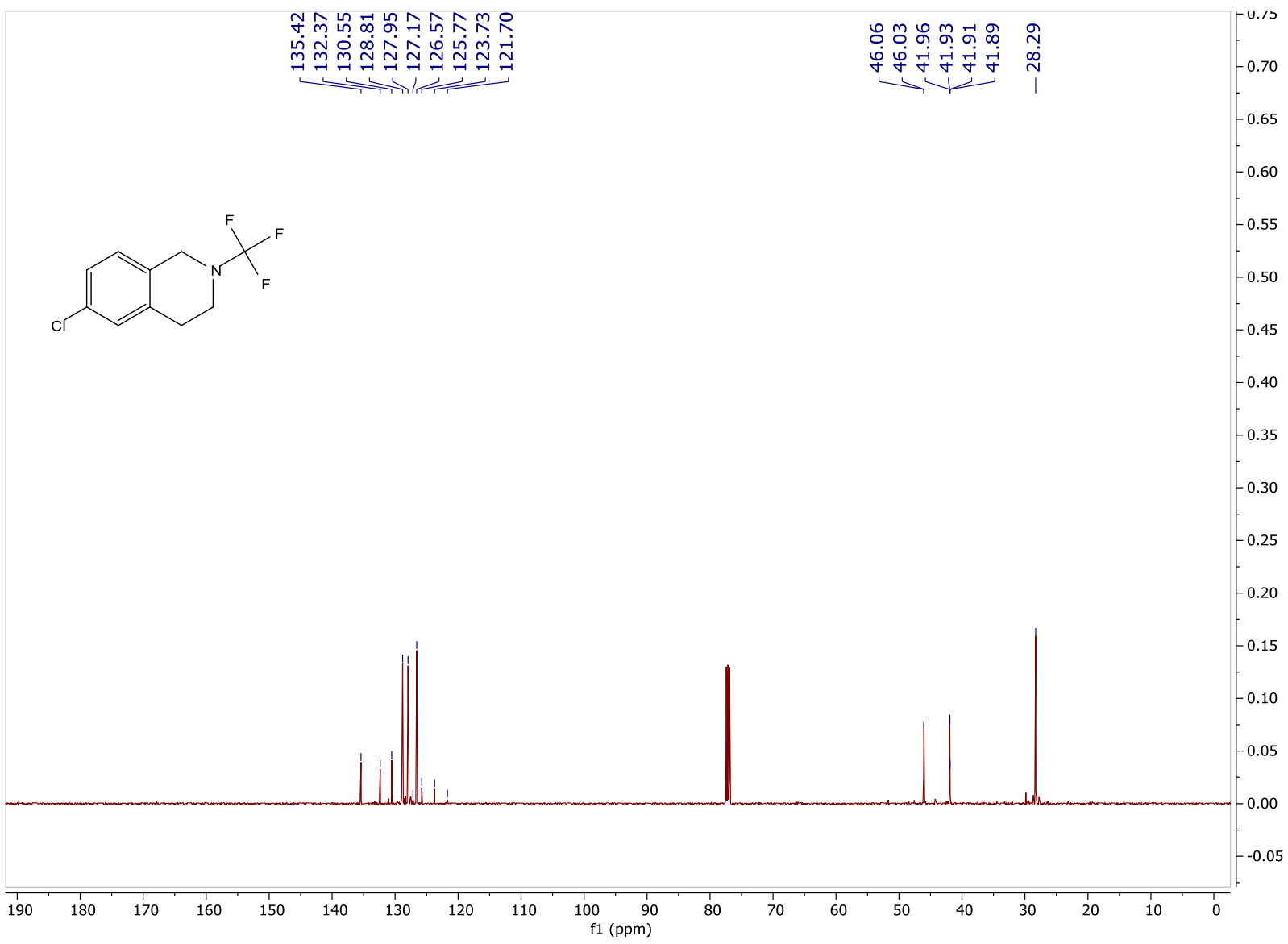

$^{13}$C NMR (126 MHz, $CDCl_3$) of **3f.**

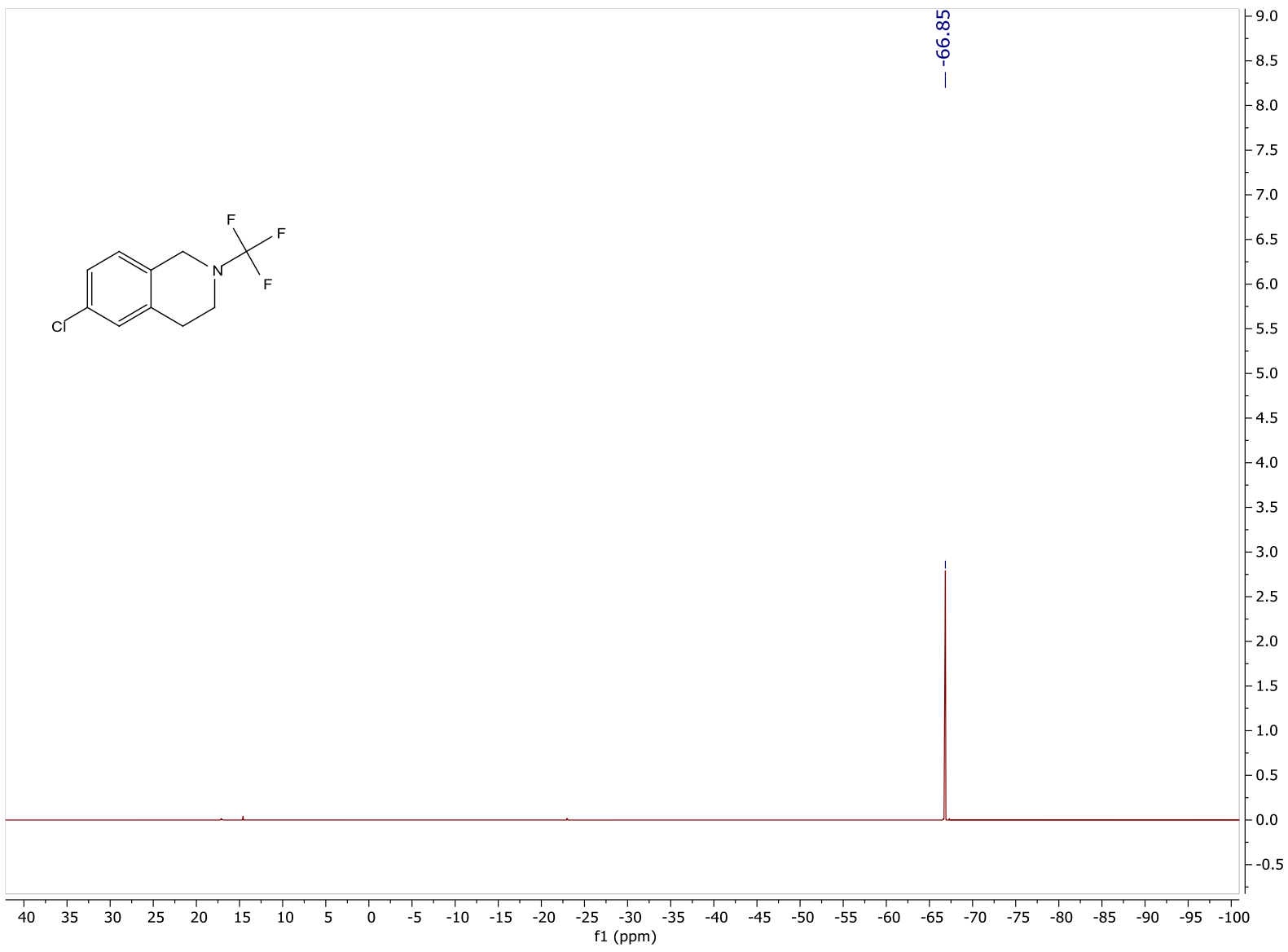


$^{19}$F NMR (376 MHz, $CDCl_3$) δ of **3f.**

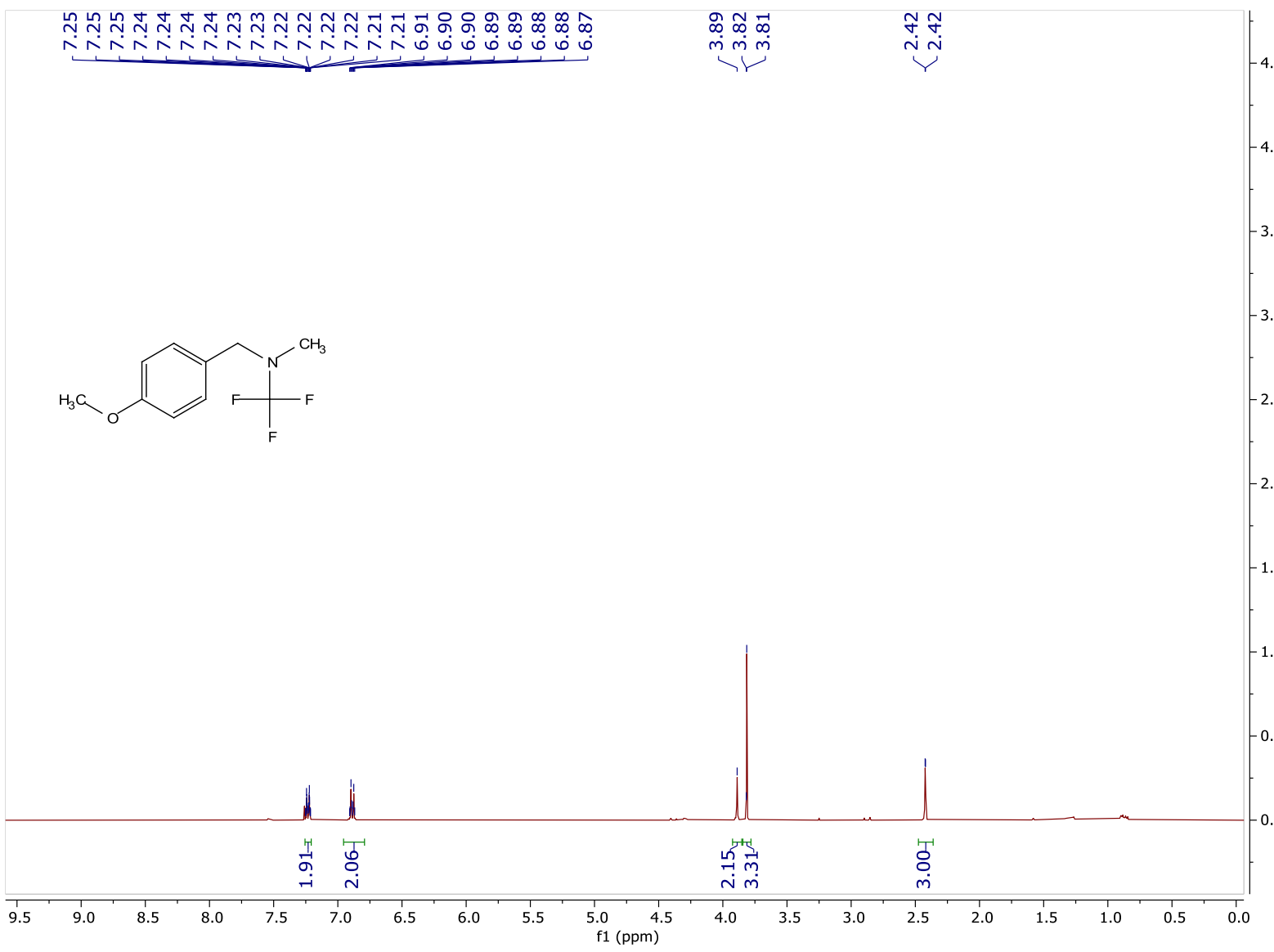


$^{1}$H NMR (400 MHz, $CDCl_3$) of **3g.**

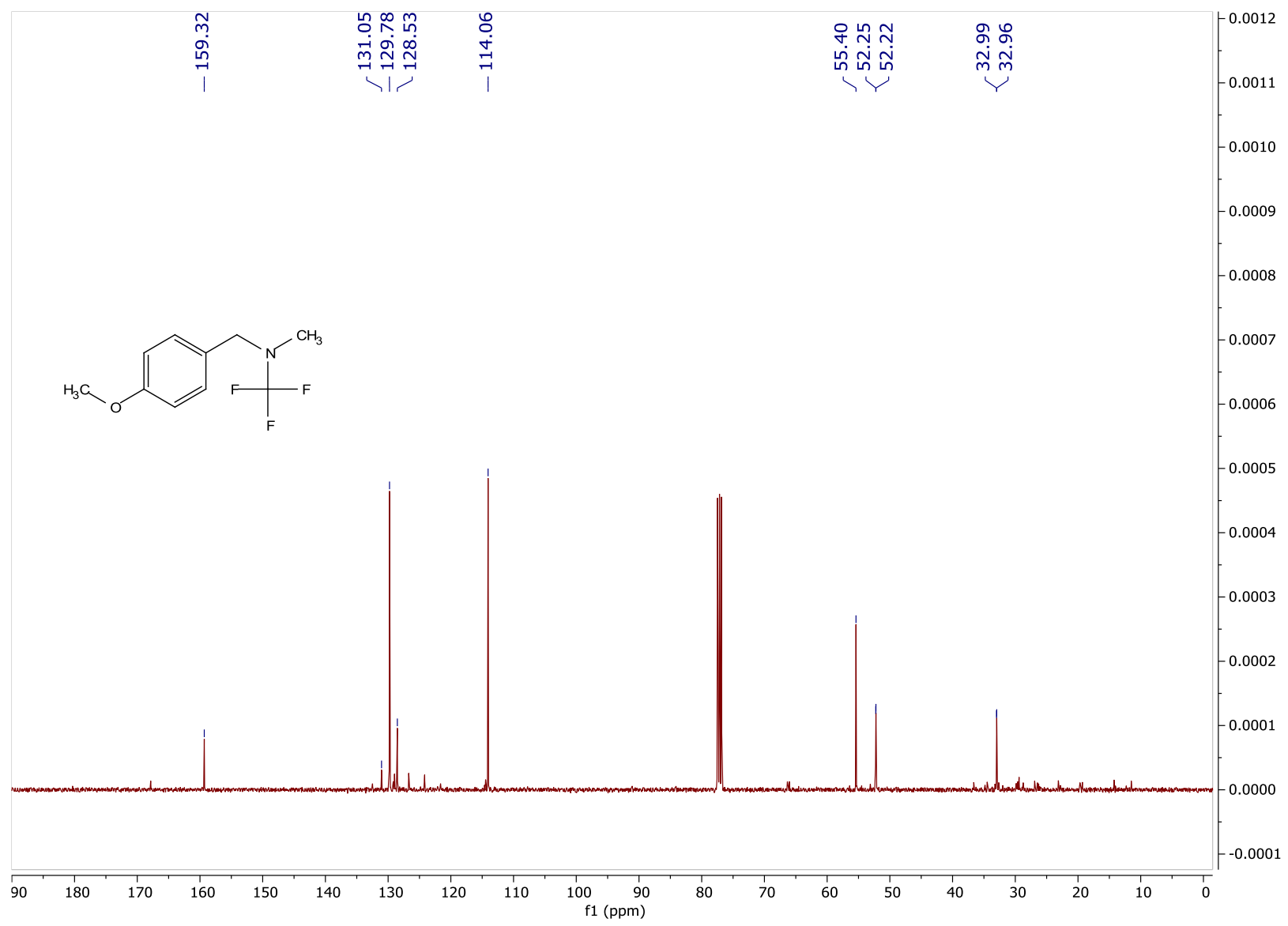

$^{13}C$ NMR (101 MHz, $CDCl_3$) of **3g.**

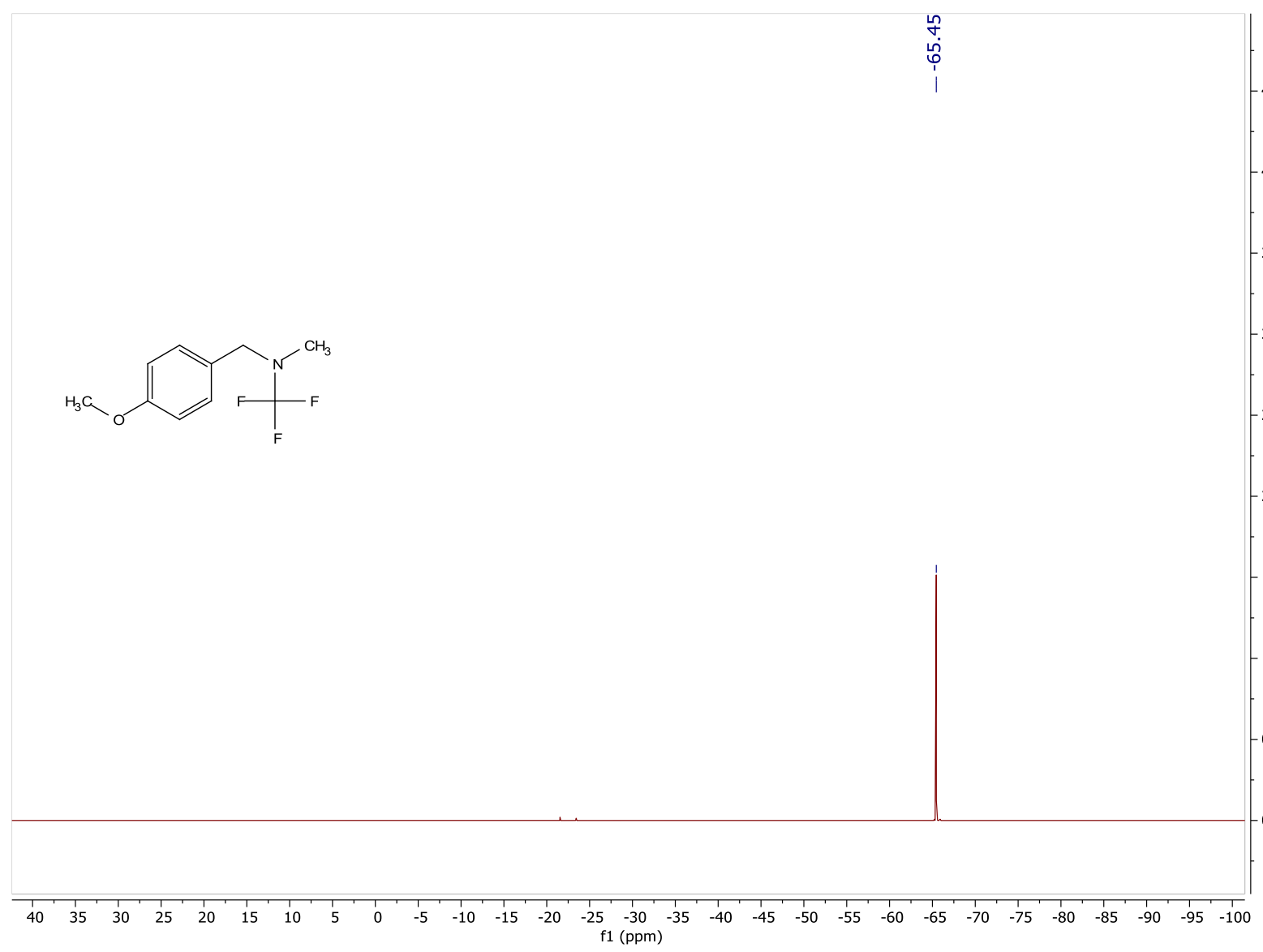

$^{19}F$ NMR (376 MHz, $CDCl_3$) of **3g.**

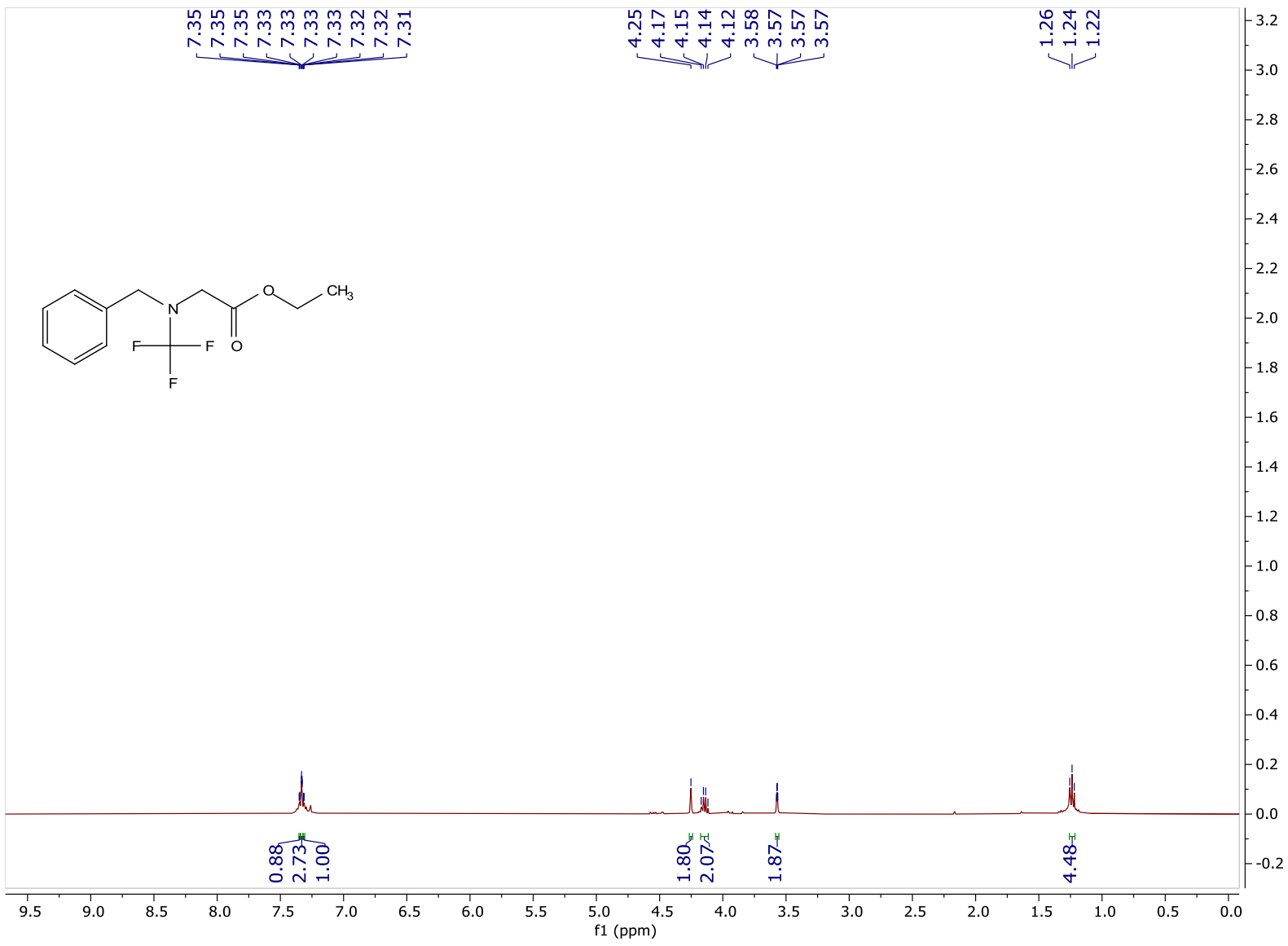

$^{1}H$ NMR (400 MHz, $CDCl_3$) of **3h.**

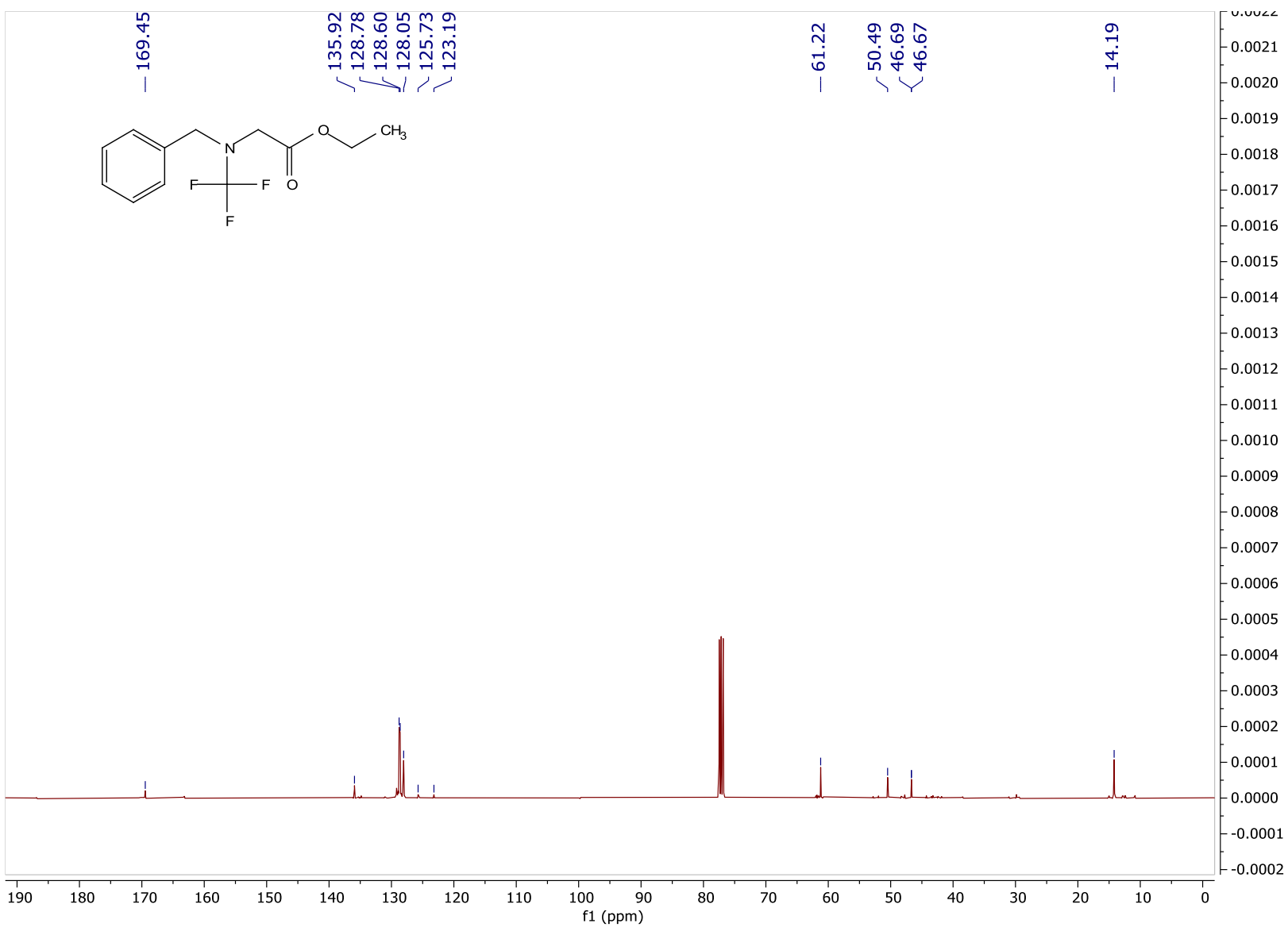

$^{13}C$ NMR (101 MHz, $CDCl_3$) of **3h.**

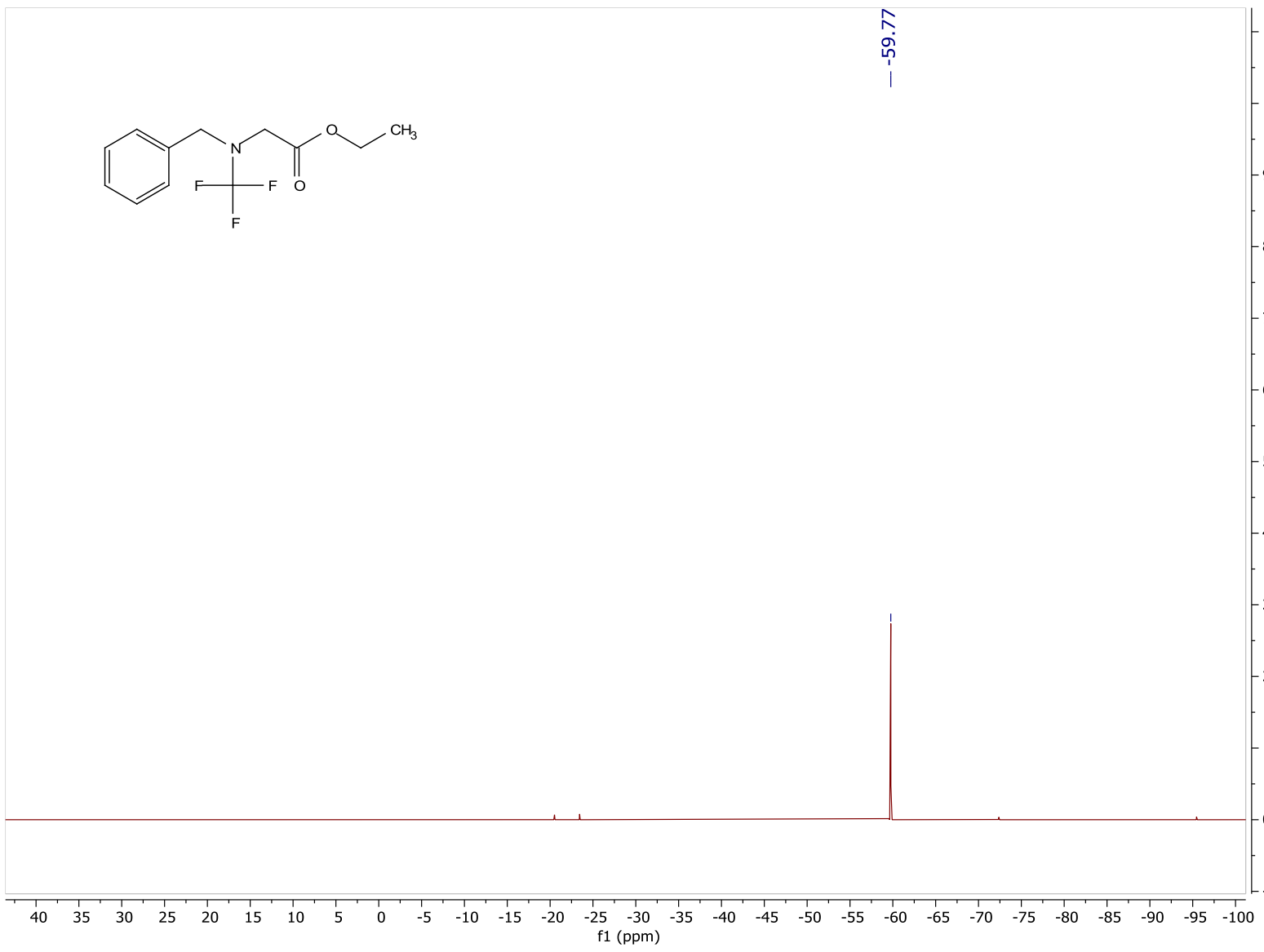

$^{19}$F NMR (376 MHz, $CDCl_3$) of **3h.**

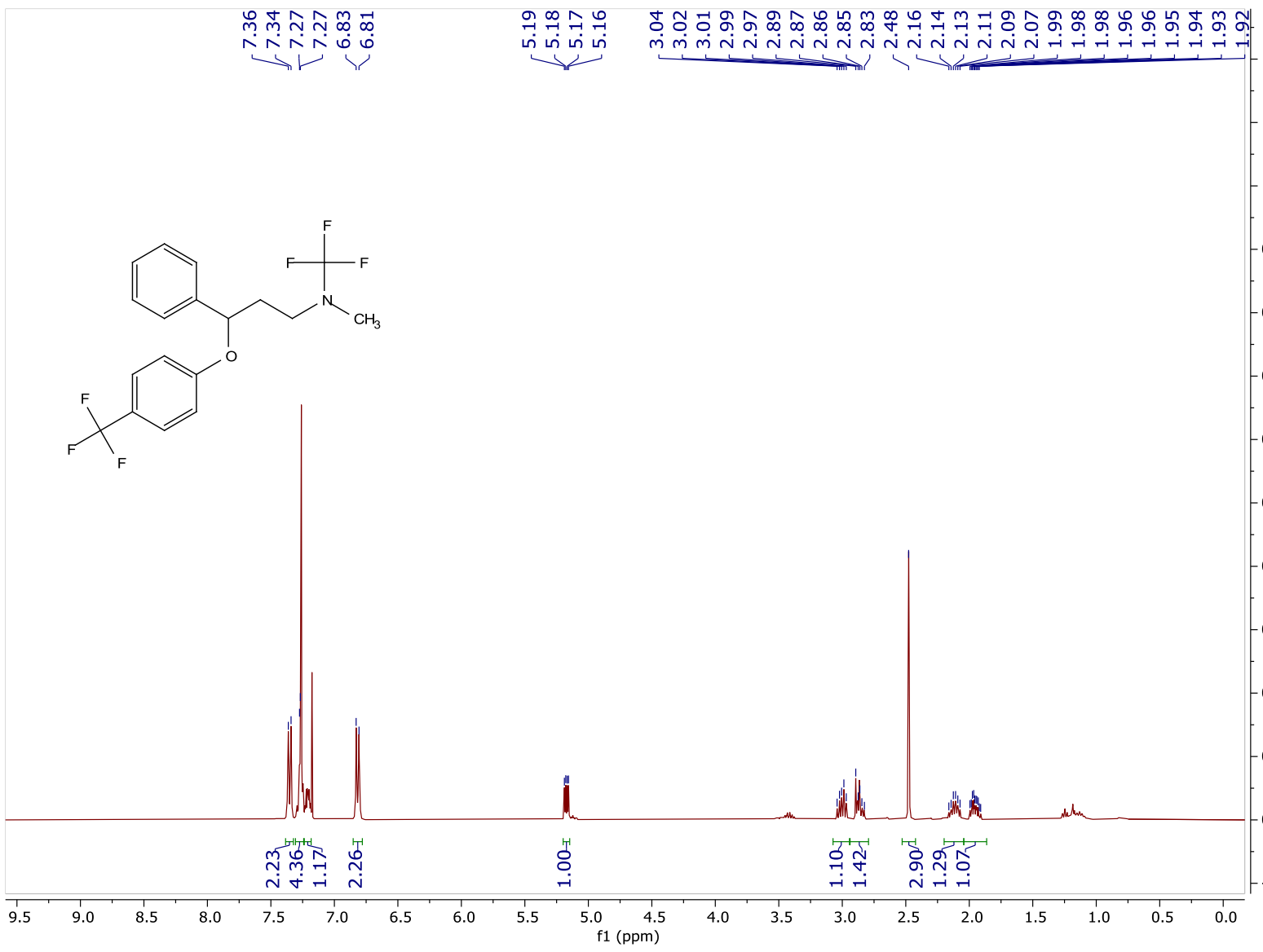

$^{1}$H NMR (400 MHz, $CDCl_3$) of **3i.**

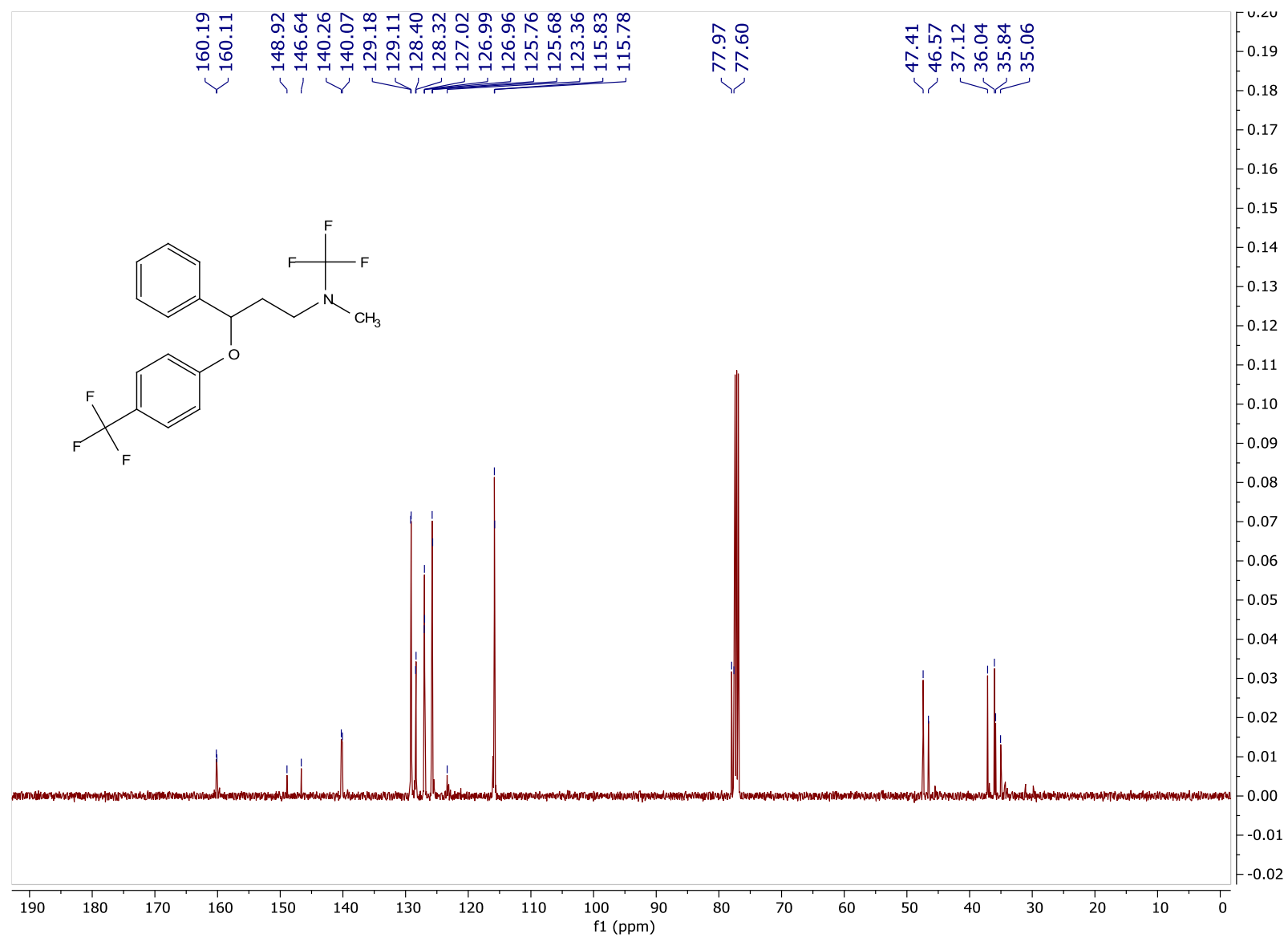

$^{13}C$ NMR (126 MHz, $CDCl_3$) of **3i.**

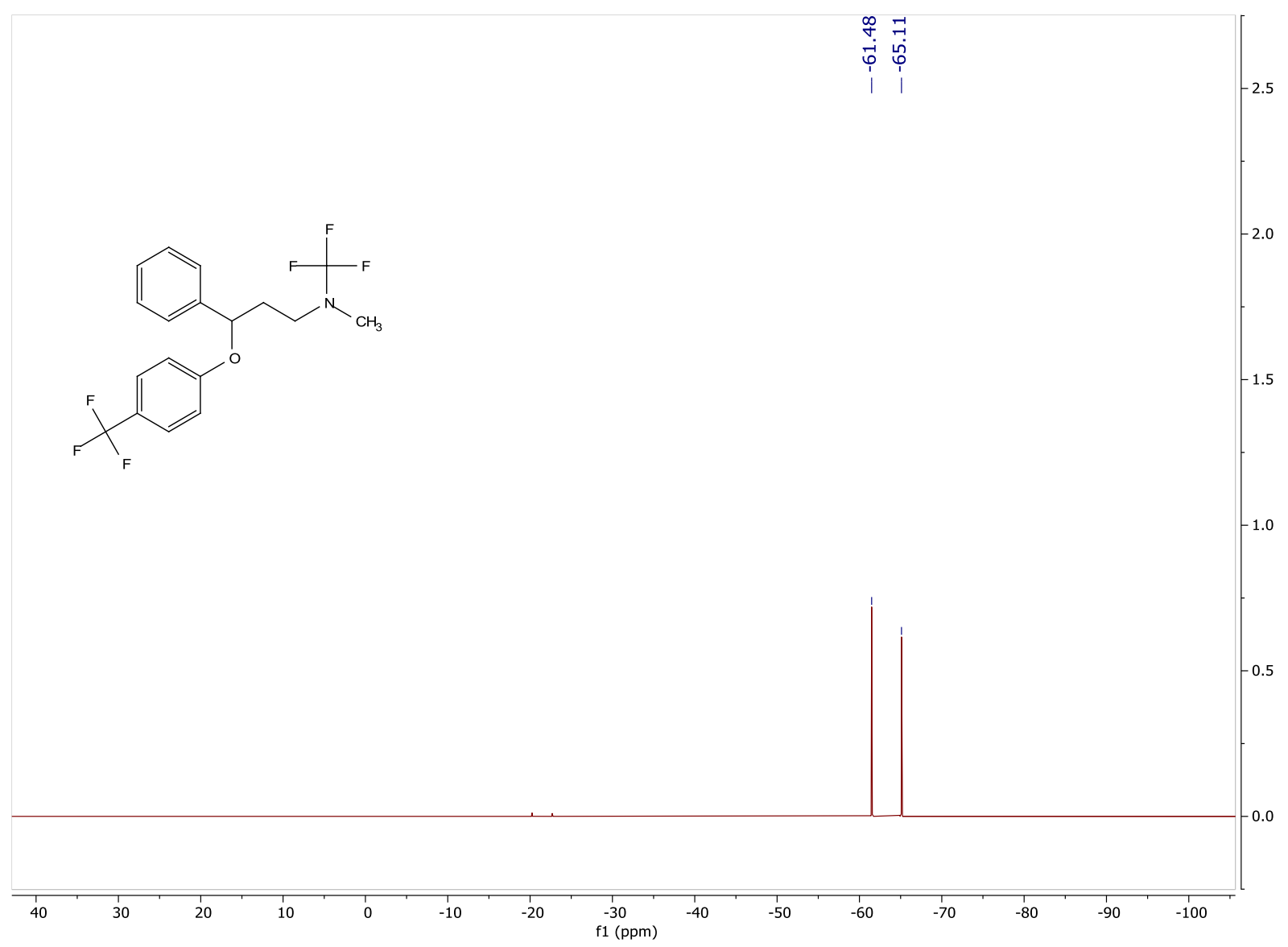

$^{19}F$ NMR (376 MHz, $CDCl_3$) of **3i.**

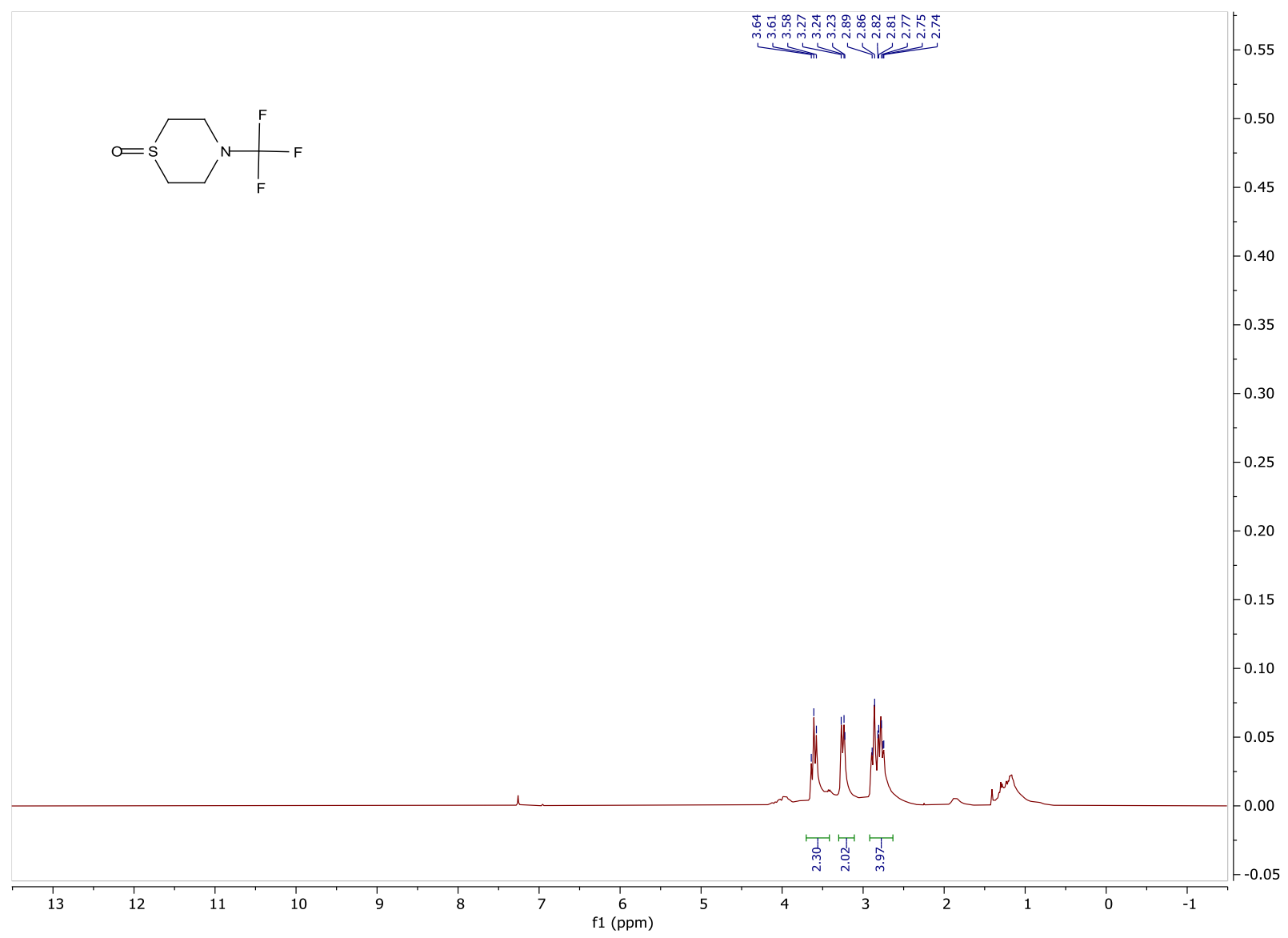

$^{1}$H NMR (400 MHz, $CDCl_3$) of **3j**.

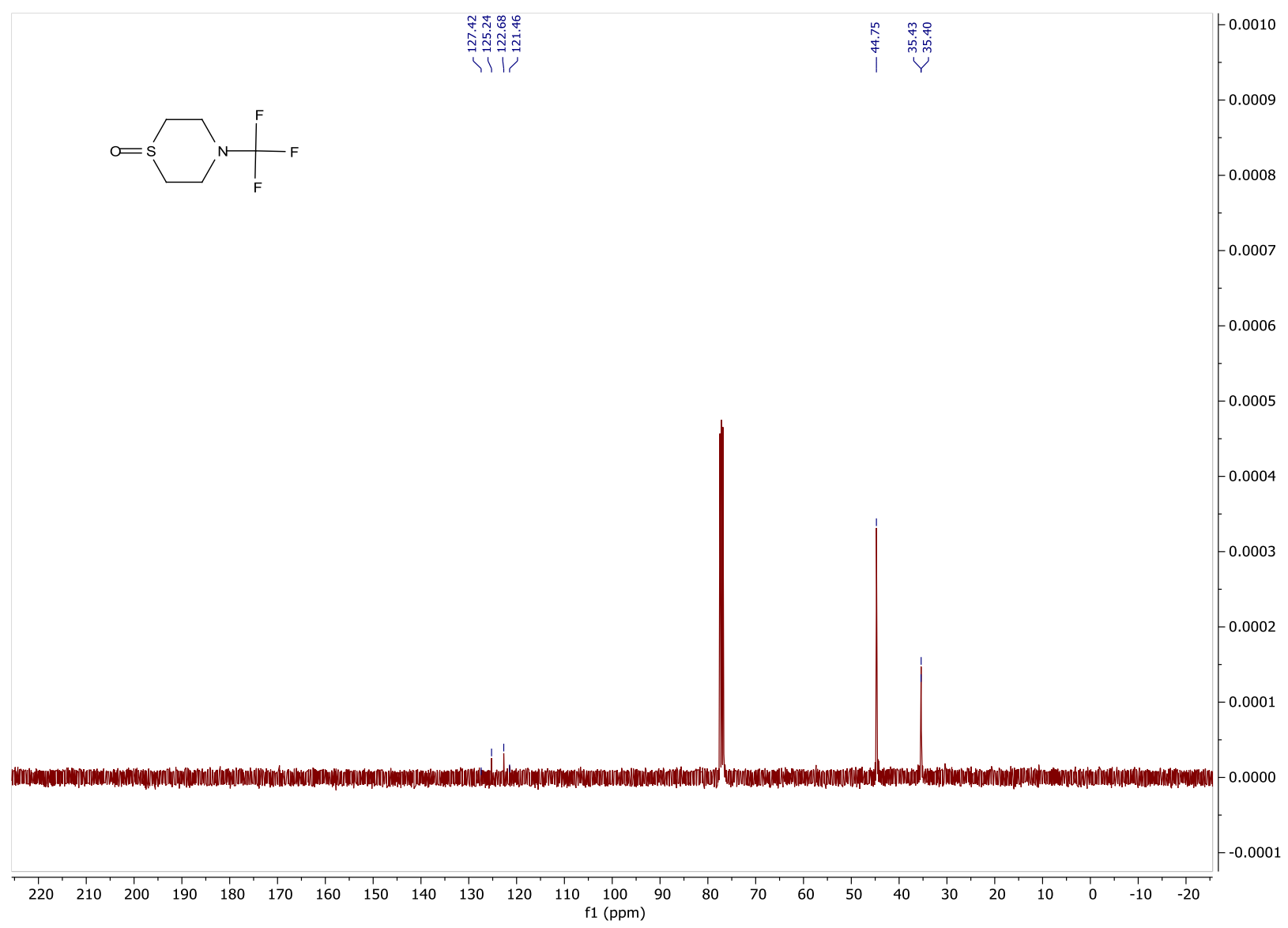

$^{13}$C NMR (101 MHz, $CDCl_3$) of **3j**.

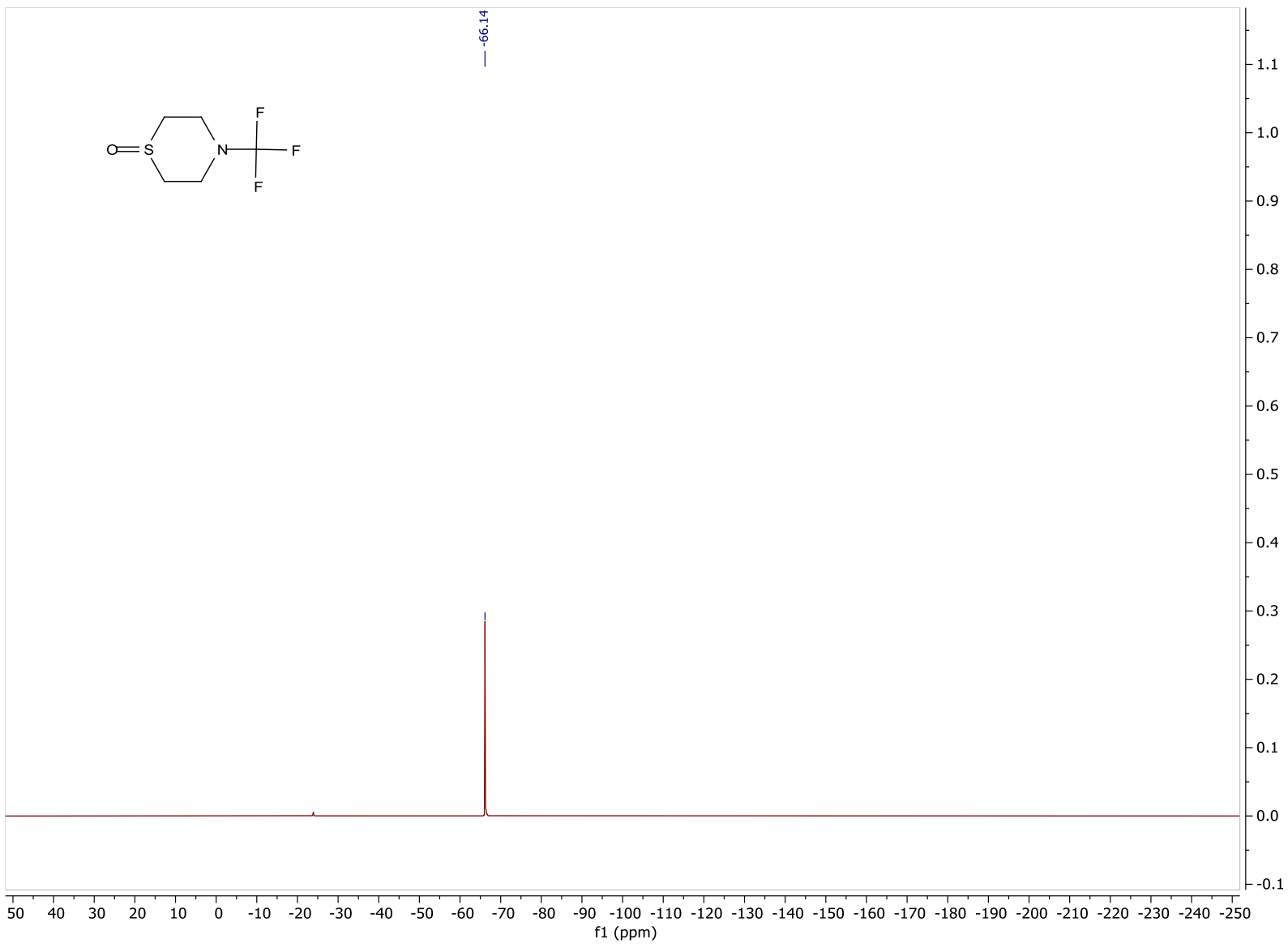


$^{19}$F NMR (376 MHz, $CDCl_3$) of **3j**.

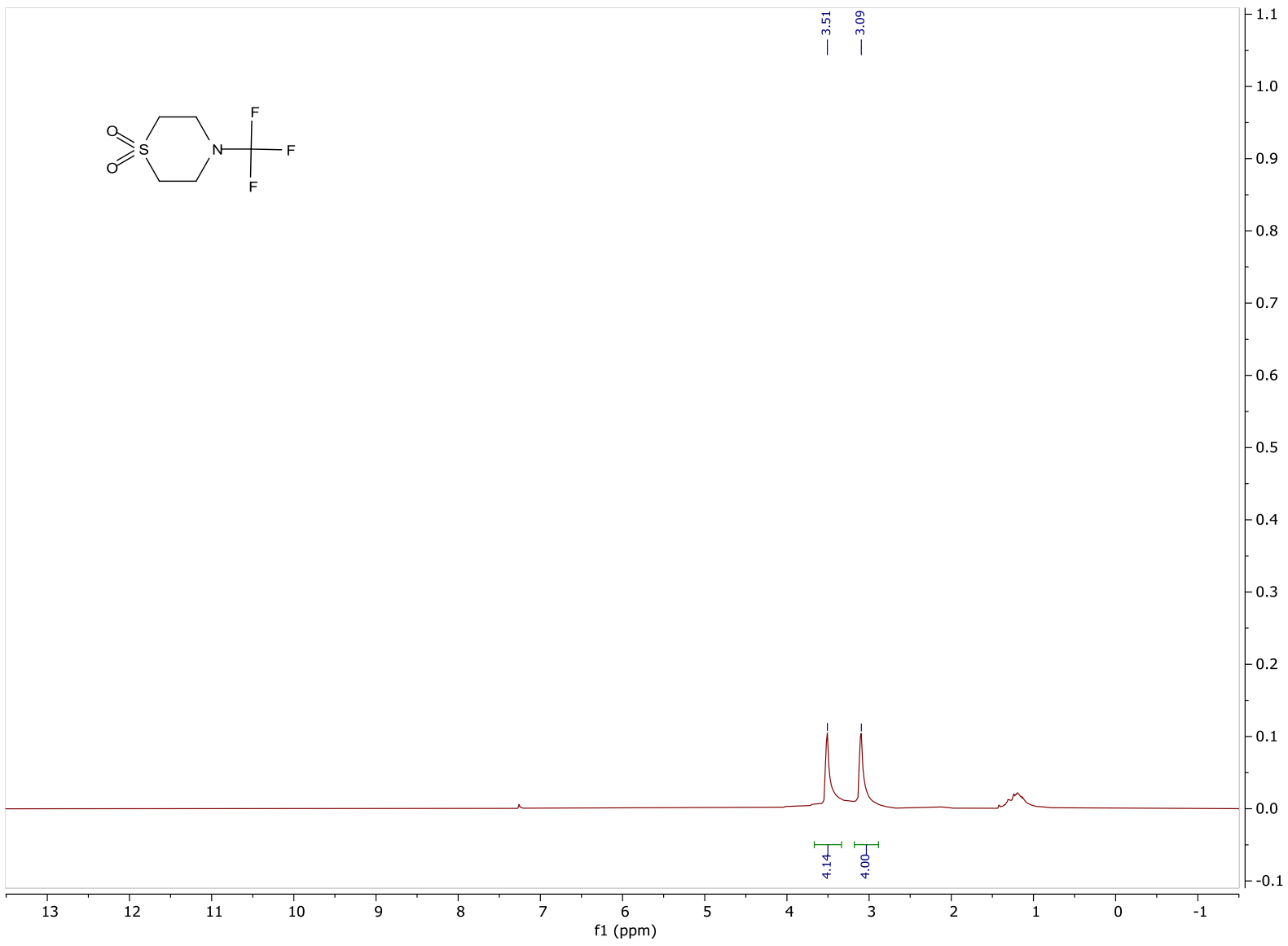


$^{1}$H NMR (400 MHz, $CDCl_3$) of **3k**.

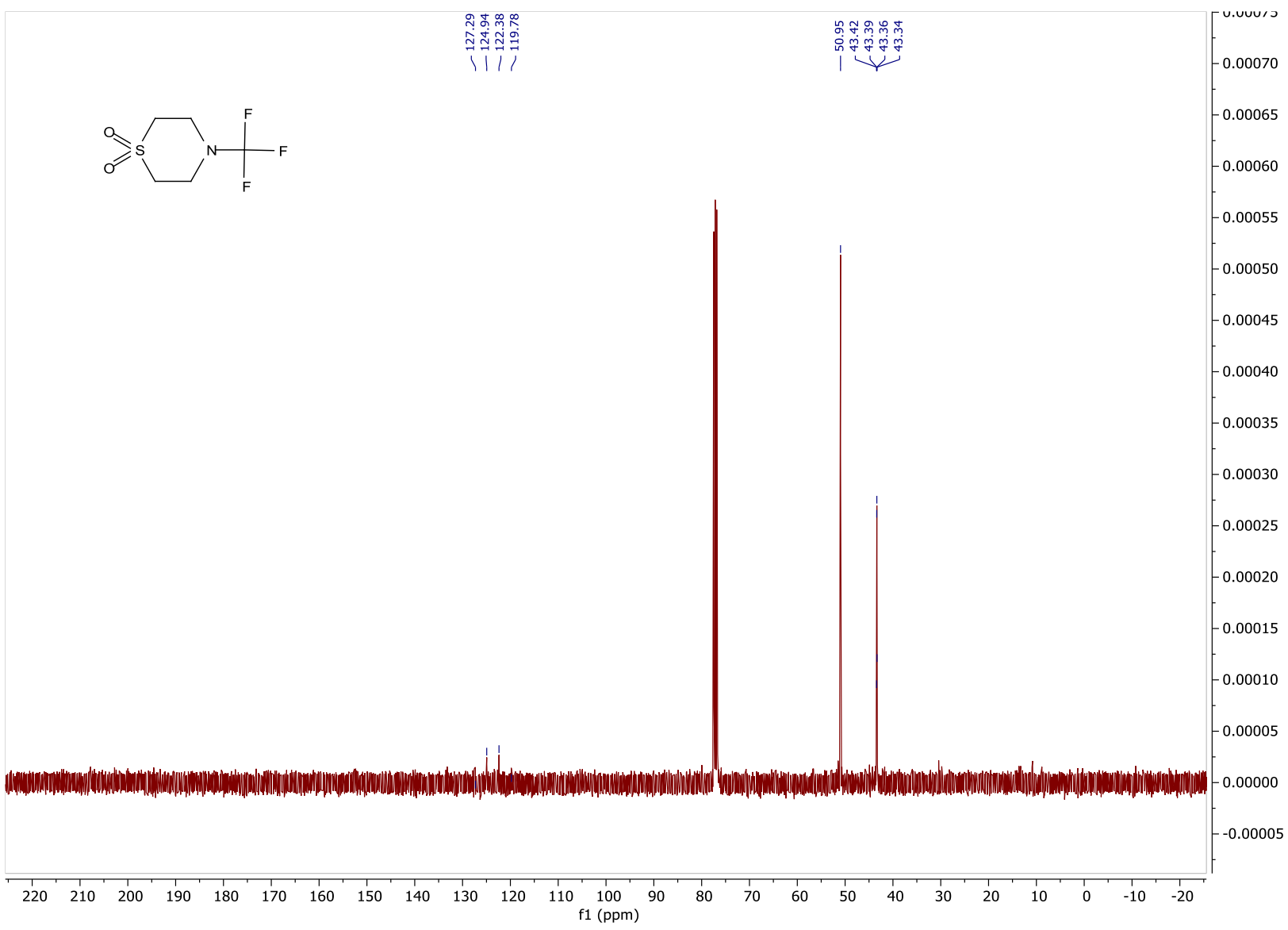

$^{13}C$ NMR (101 MHz, $CDCl_3$) of **3k**.

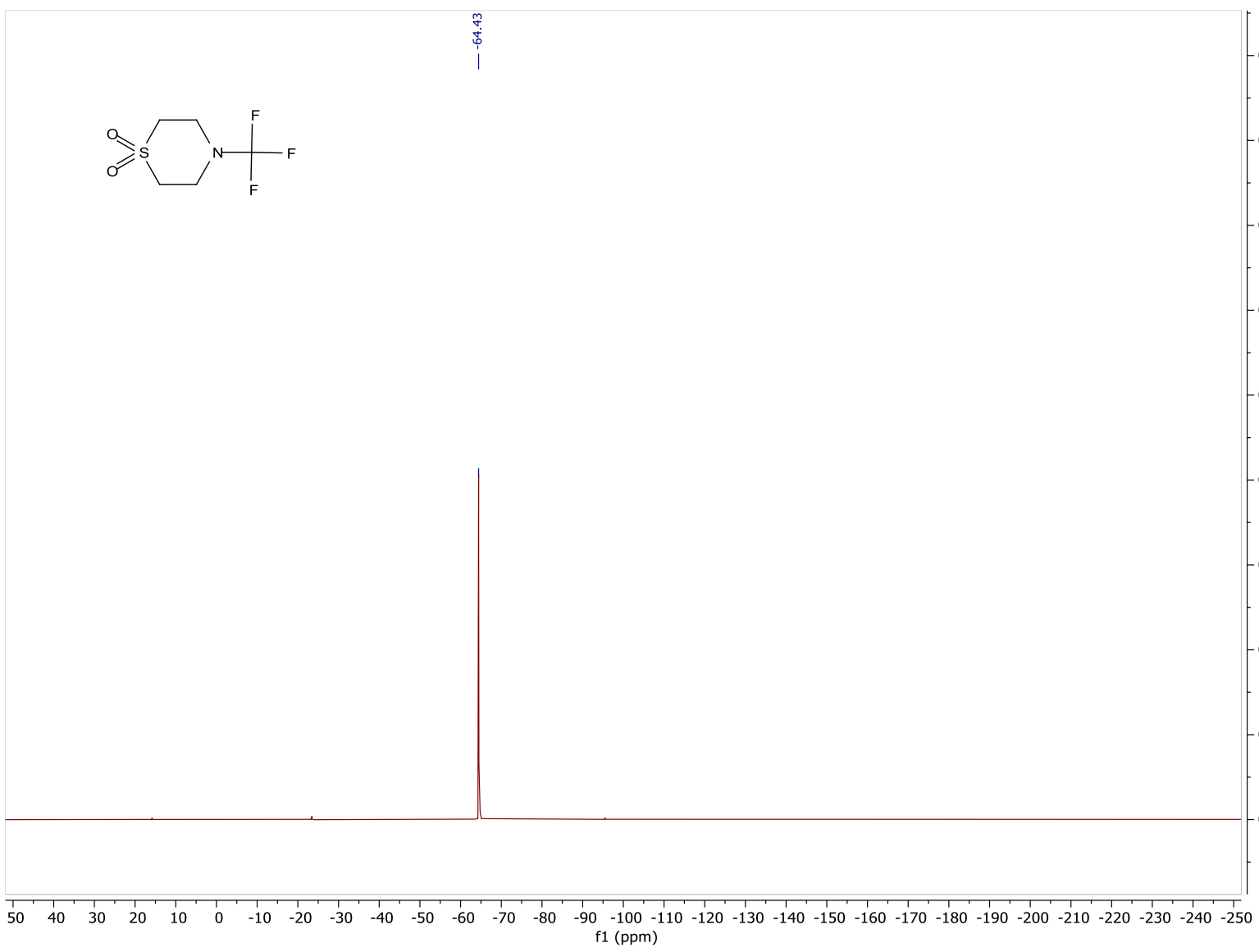

$^{19}F$ NMR (376 MHz, $CDCl_3$) of **3k**.

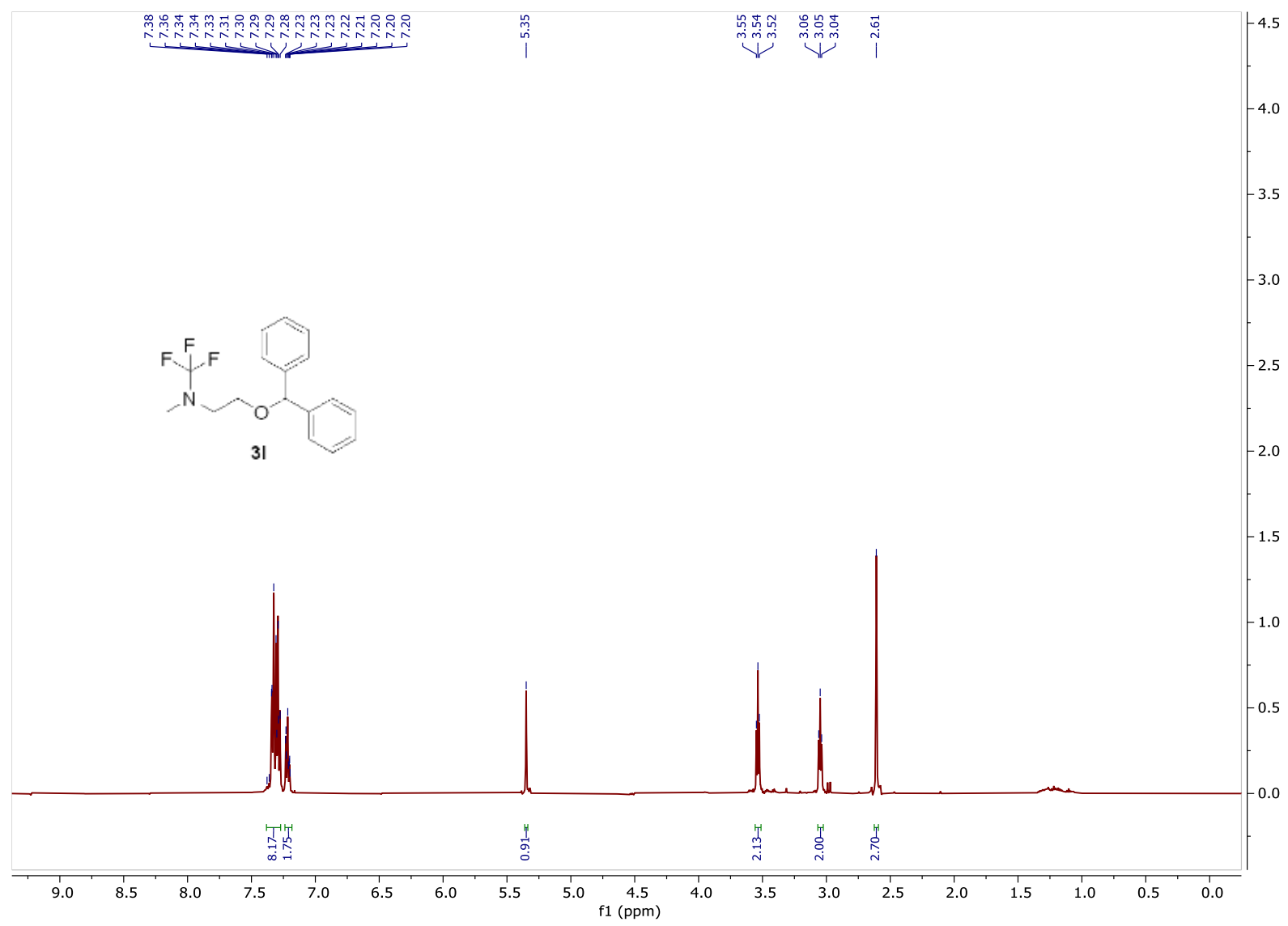

$^{1}H$ NMR (400 MHz, $CDCl_3$) of **3l.**

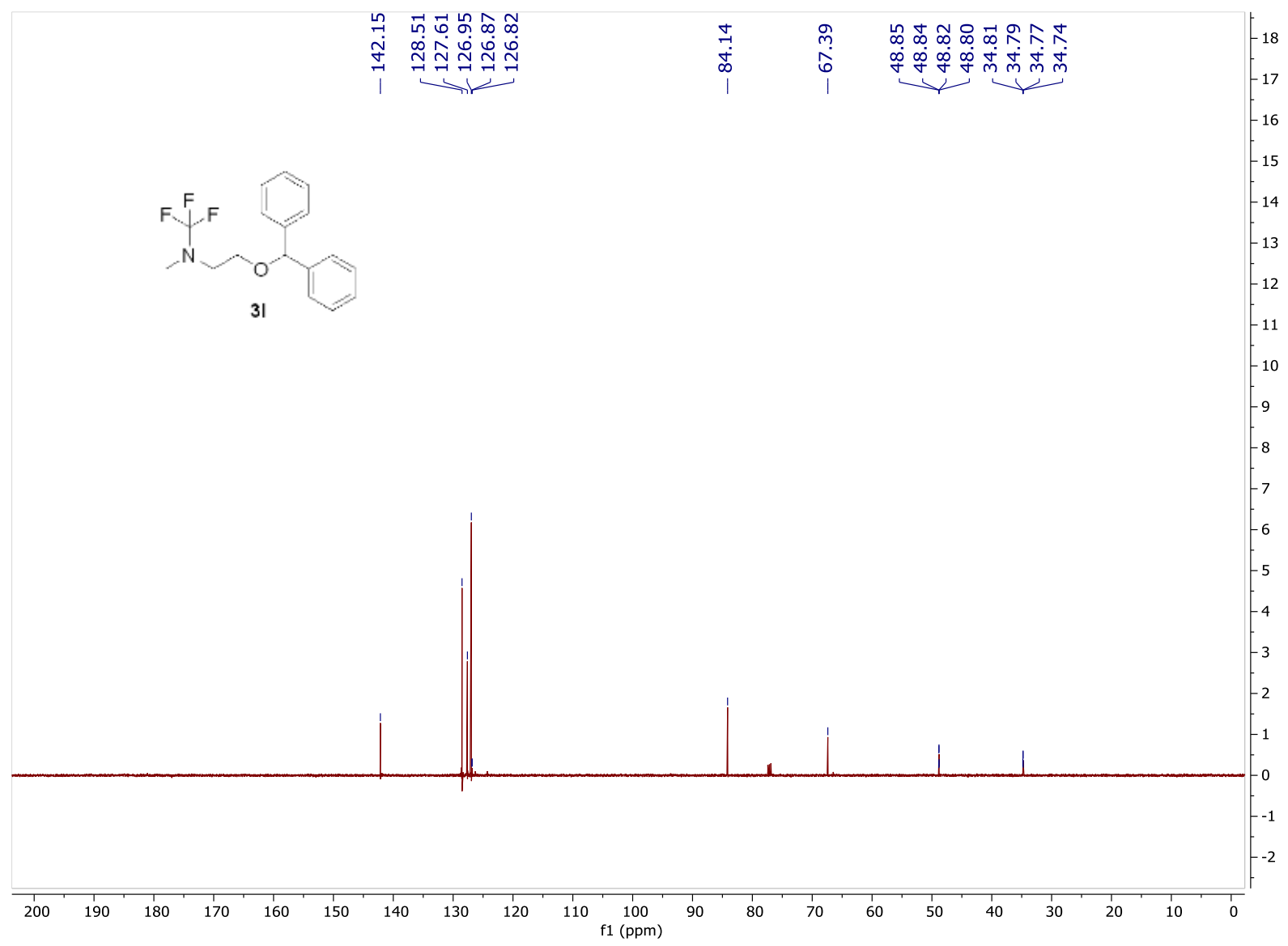

$^{13}C$ NMR (101 MHz, $CDCl_3$) of **3l.**

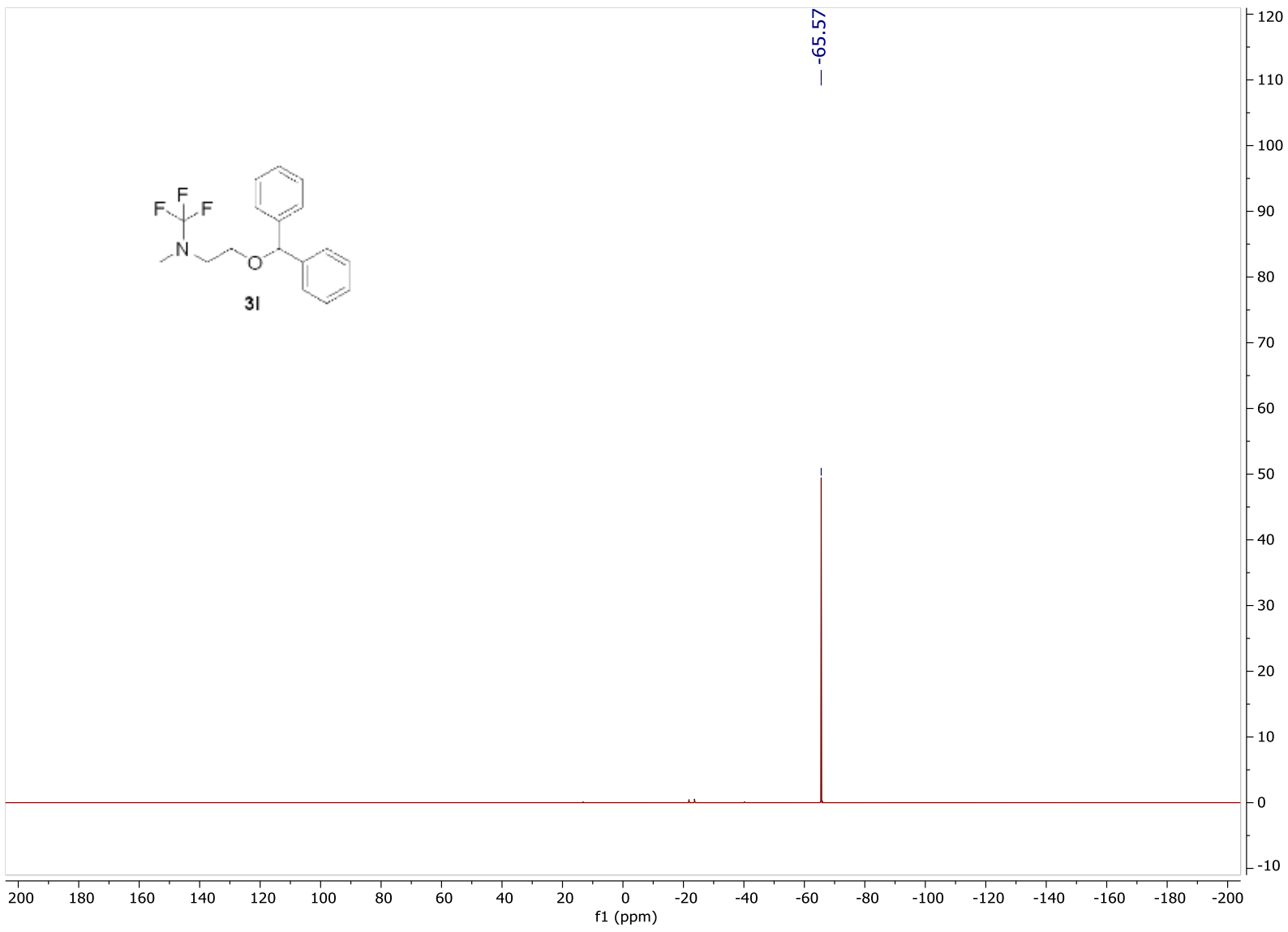

$^{19}$F NMR (367 MHz, $CDCl_3$) of **3l.**

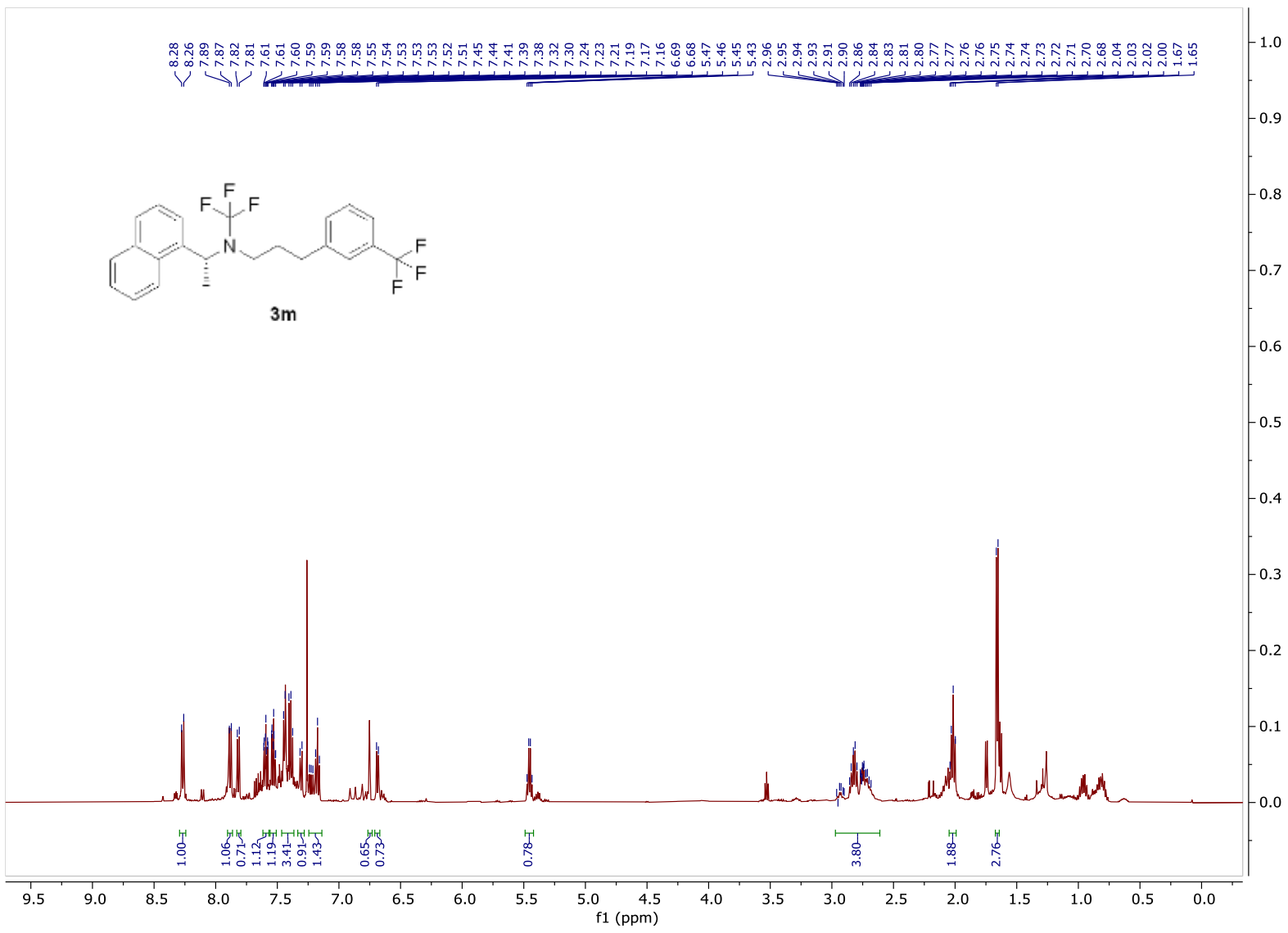

$^{1}$H NMR (400 MHz, $CDCl_3$) of **3m.**

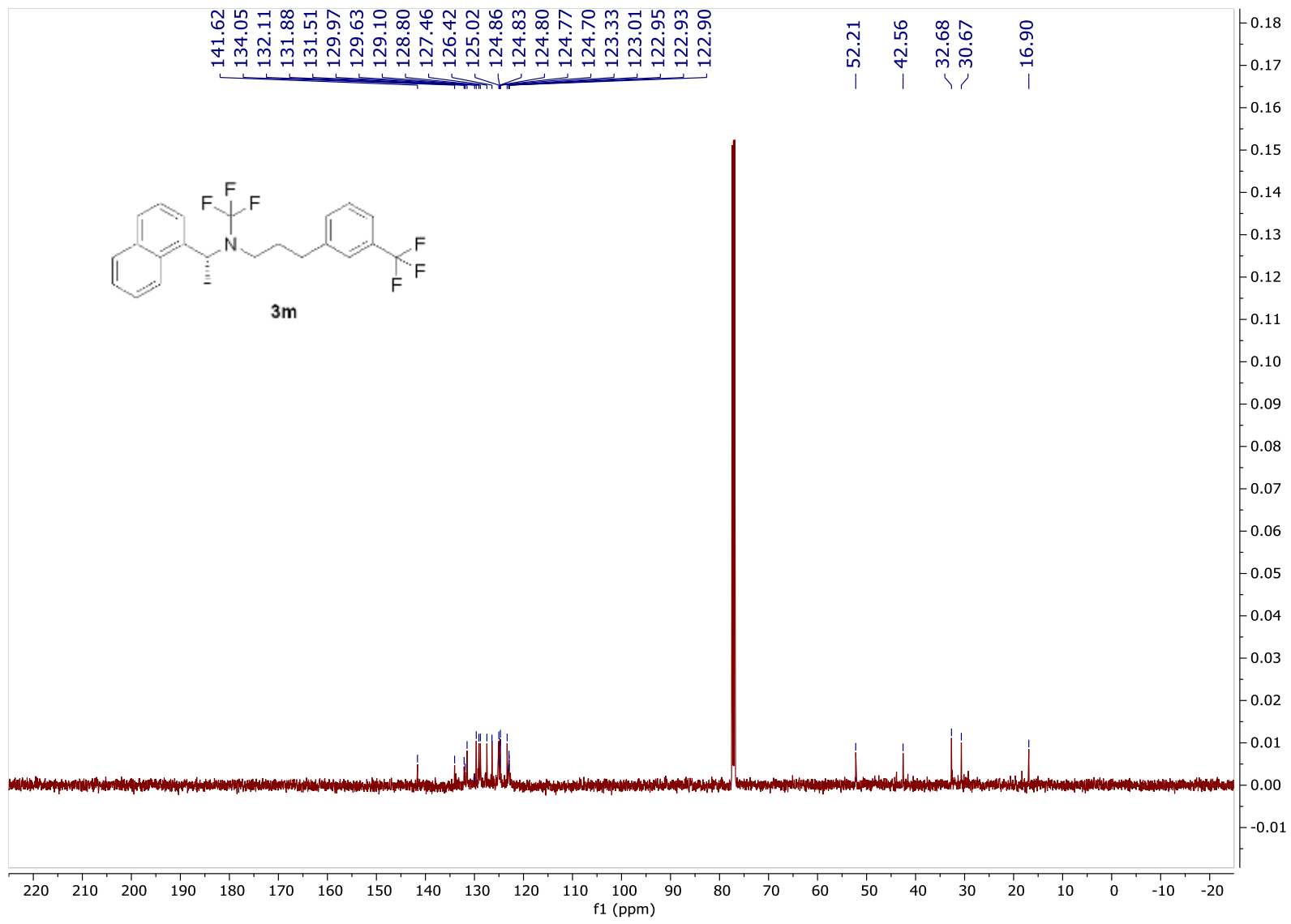

$^{13}$C NMR (101 MHz, $CDCl_3$) of **3m.**

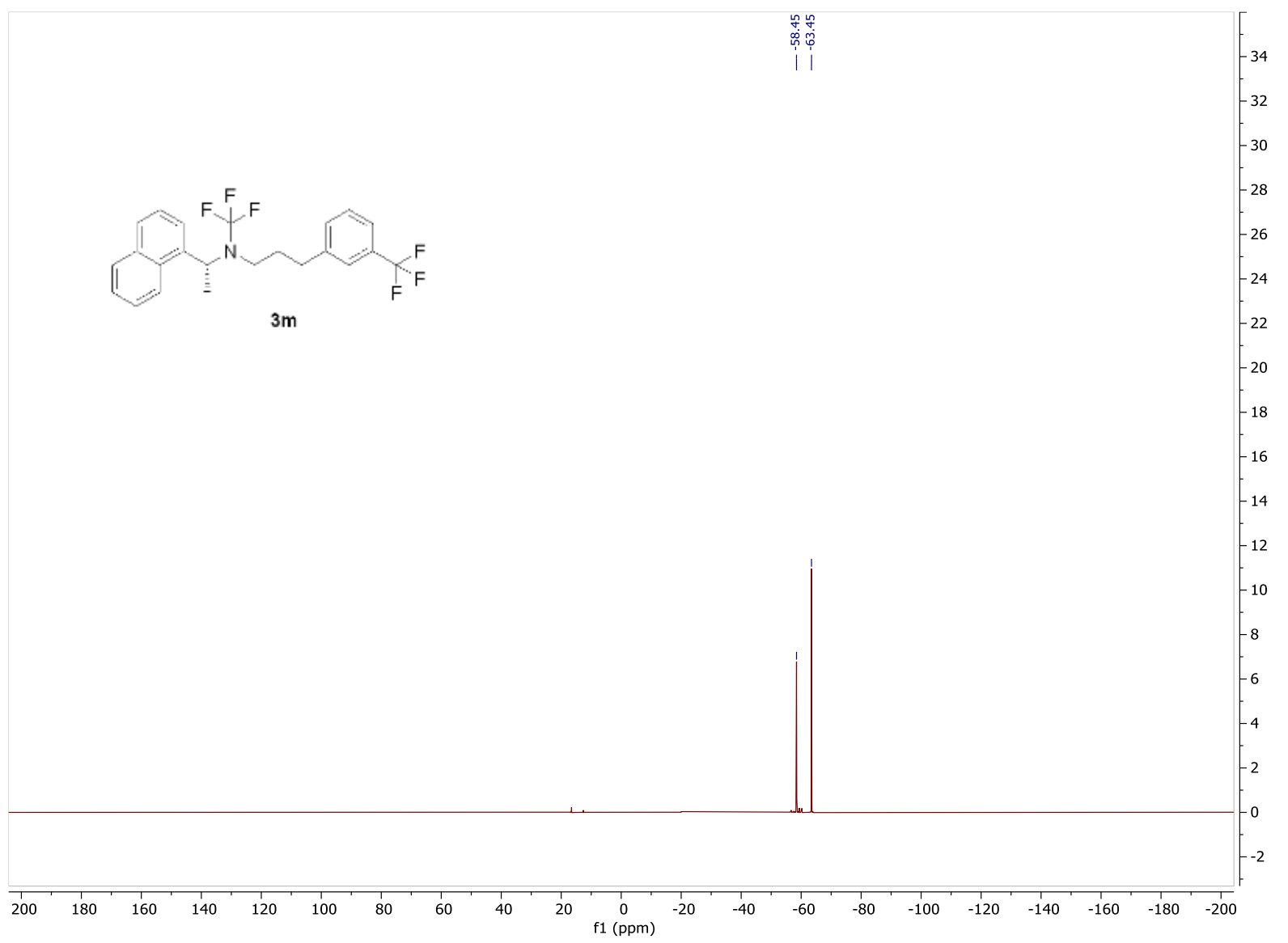

$^{19}$F NMR (367 MHz, $CDCl_3$) of **3m.**

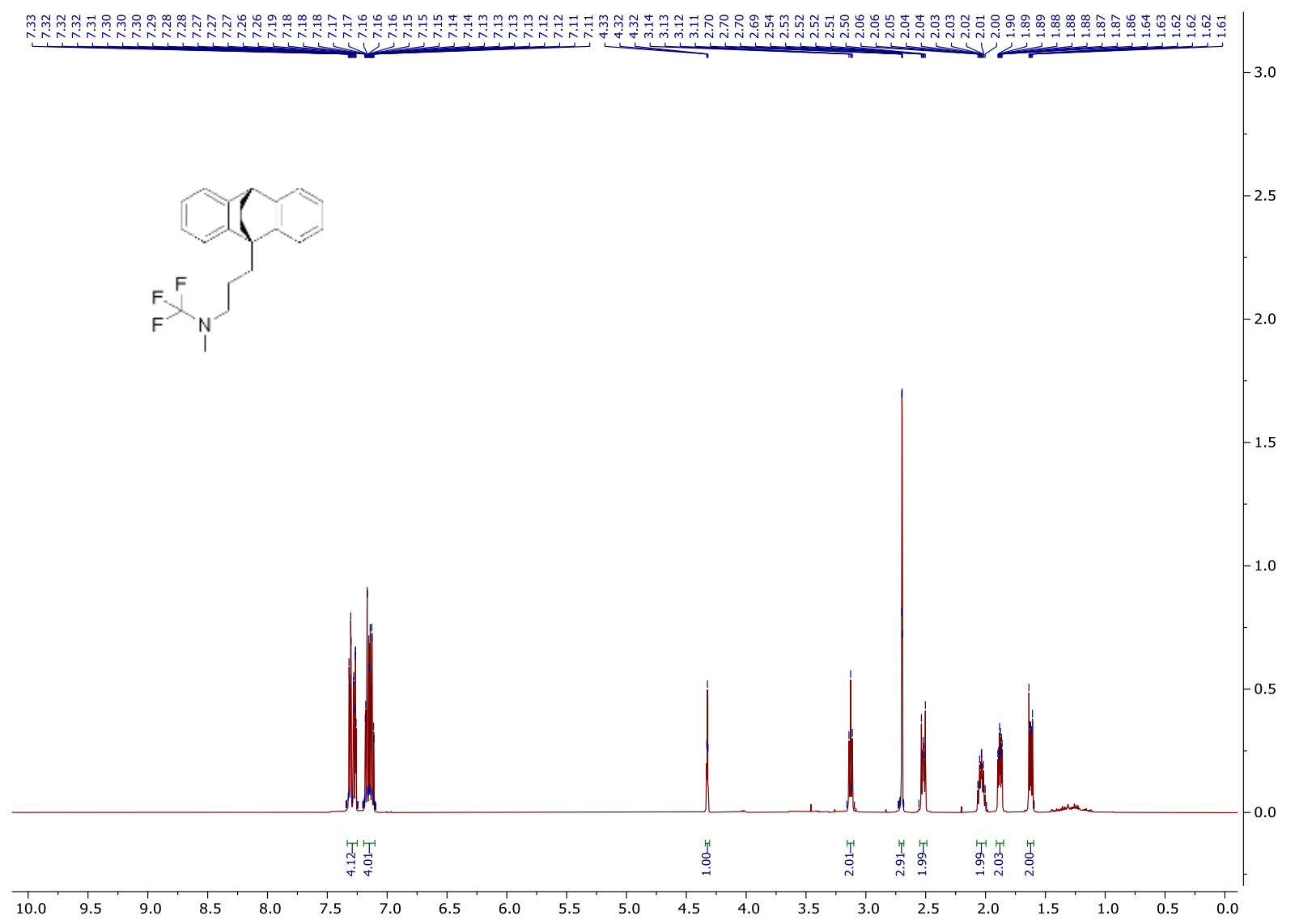

$^{1}H$ NMR (400 MHz, $CDCl_3$) of **3n**.

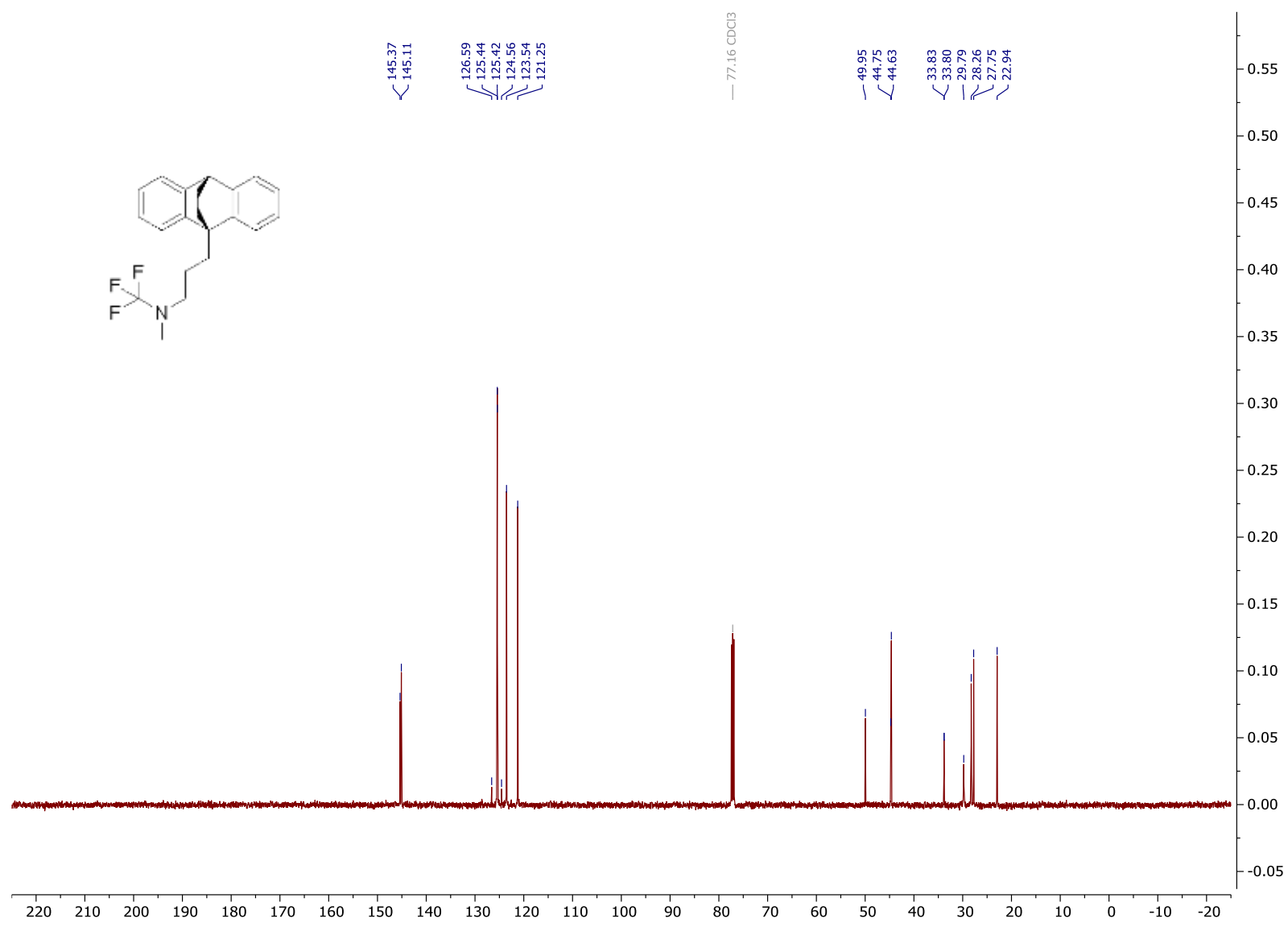

$^{13}C$ NMR (101 MHz, $CDCl_3$) of **3n**.

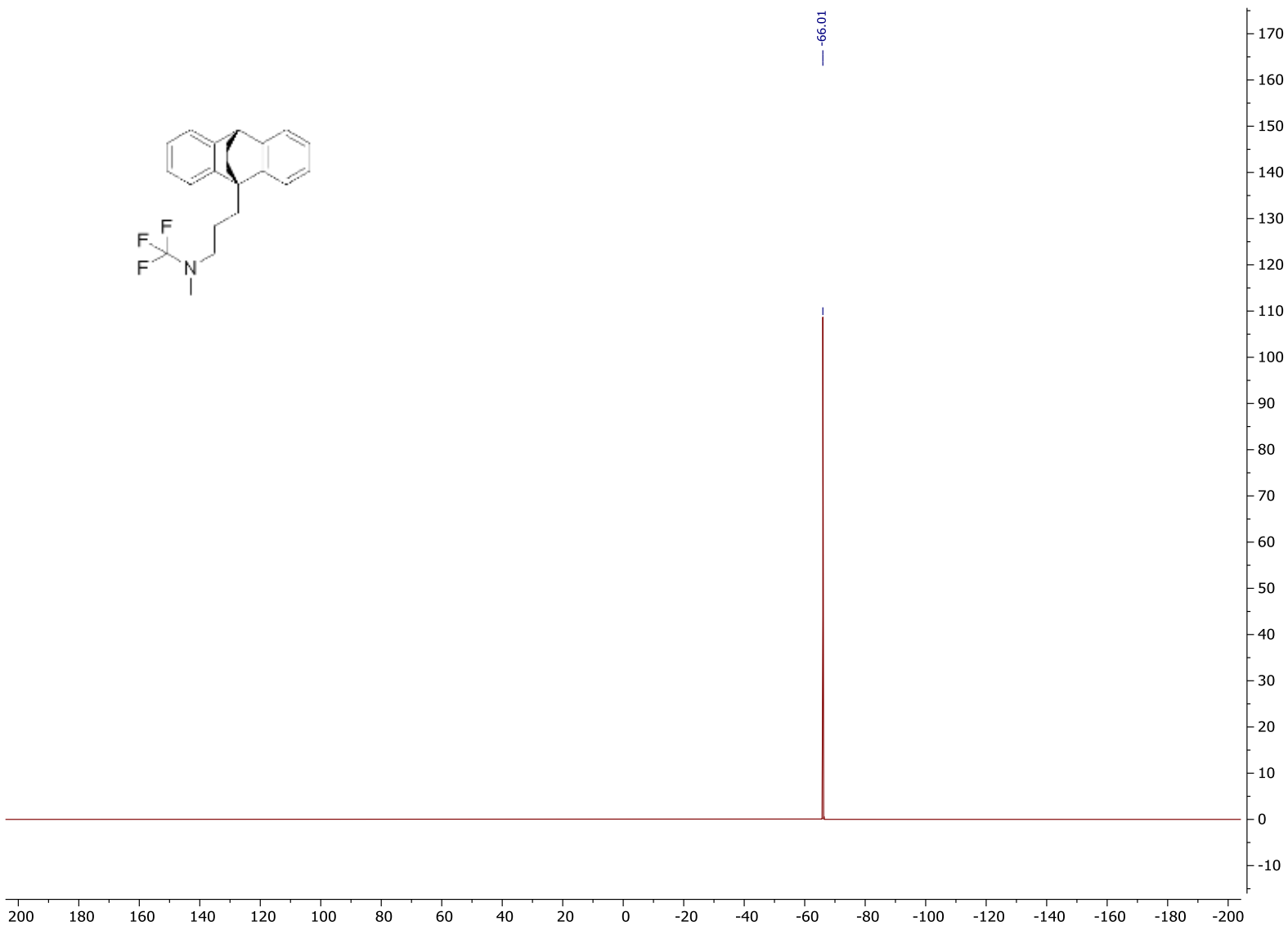


$^{19}$F NMR (376 MHz, $CDCl_3$) of **3n.**

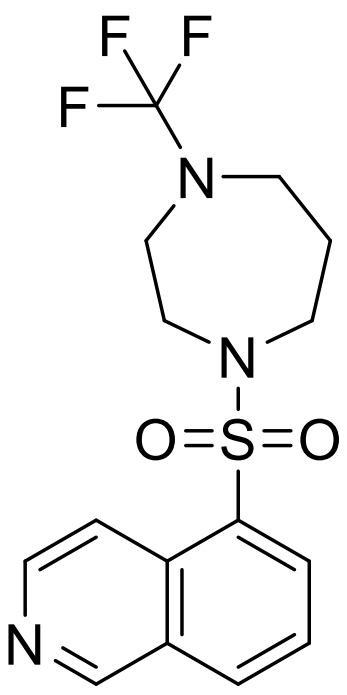

**3o**

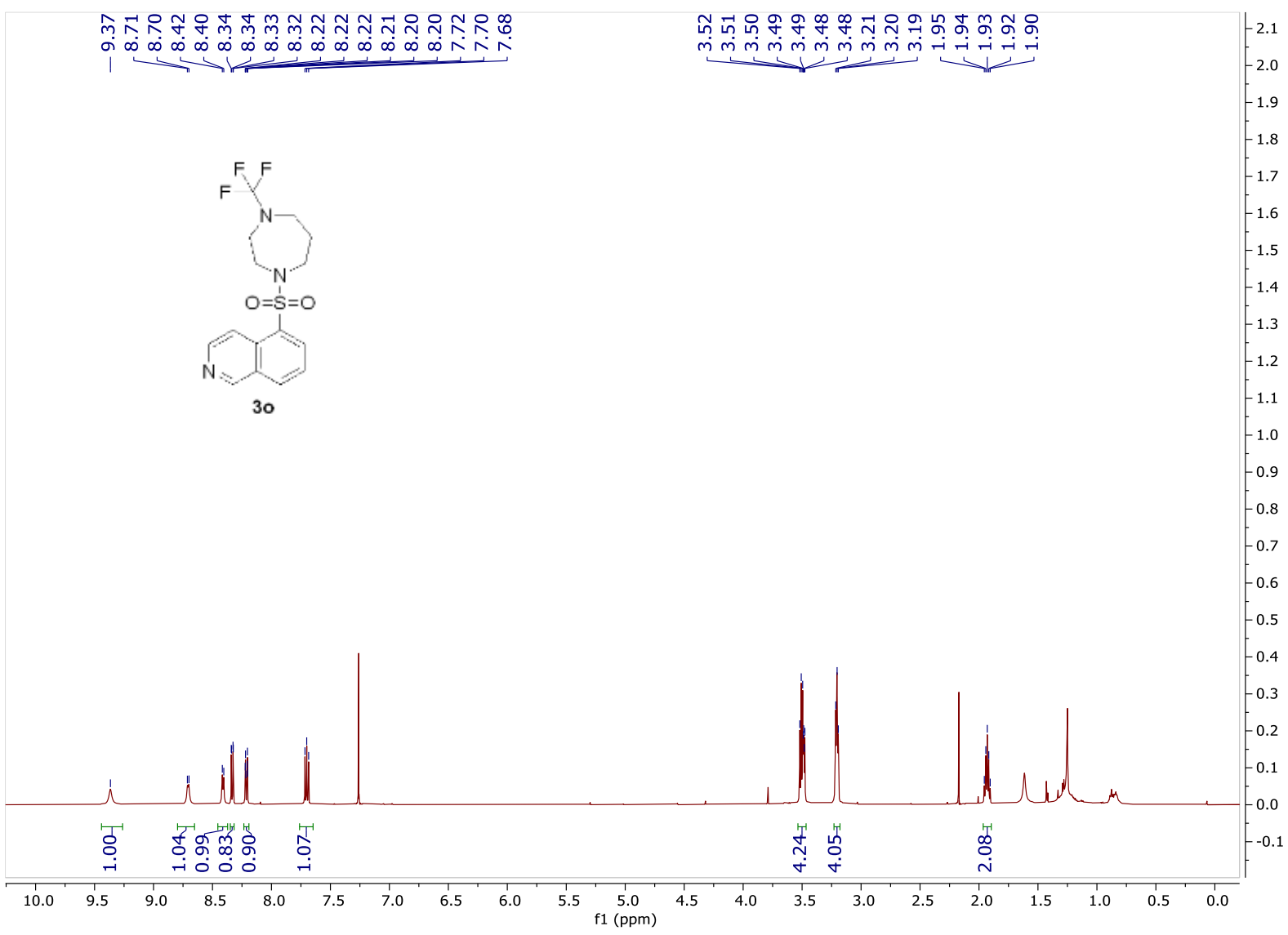


$^{1}$H NMR (400 MHz, $CDCl_3$) of **3o.**

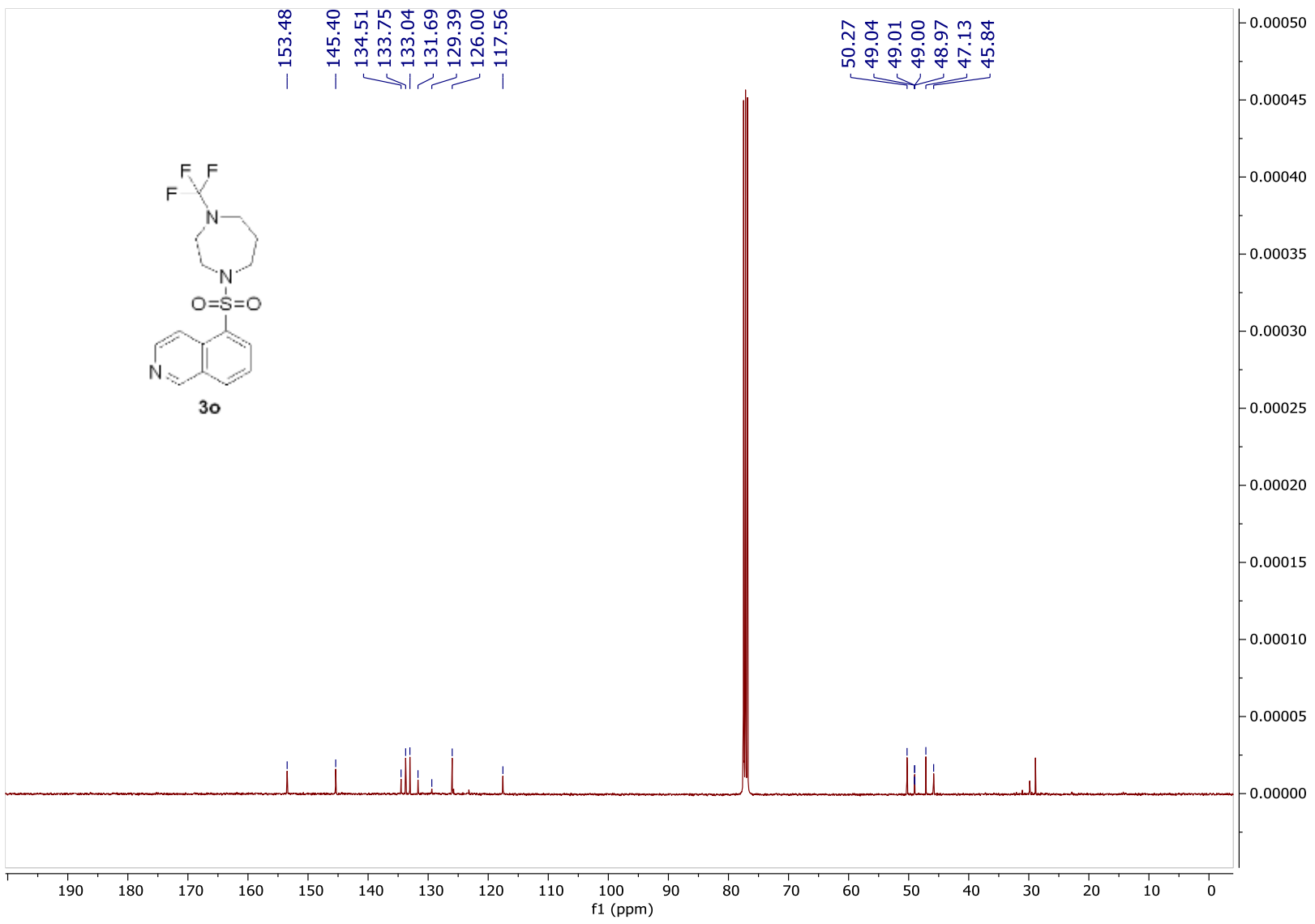


$^{13}$C NMR (101 MHz, $CDCl_3$) of **3o**.

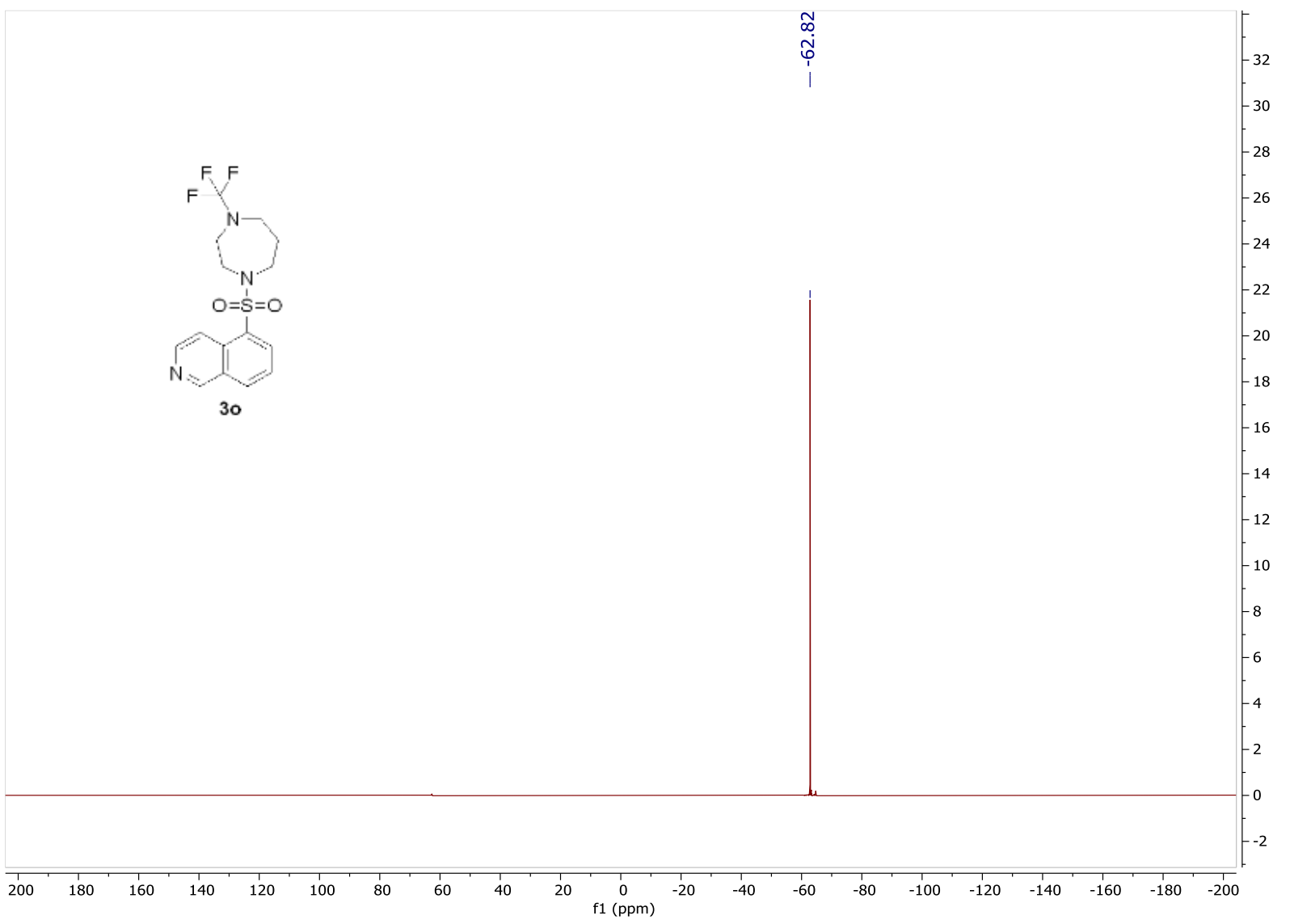


$^{19}$F NMR (376 MHz, $CDCl_3$) of **3o.**

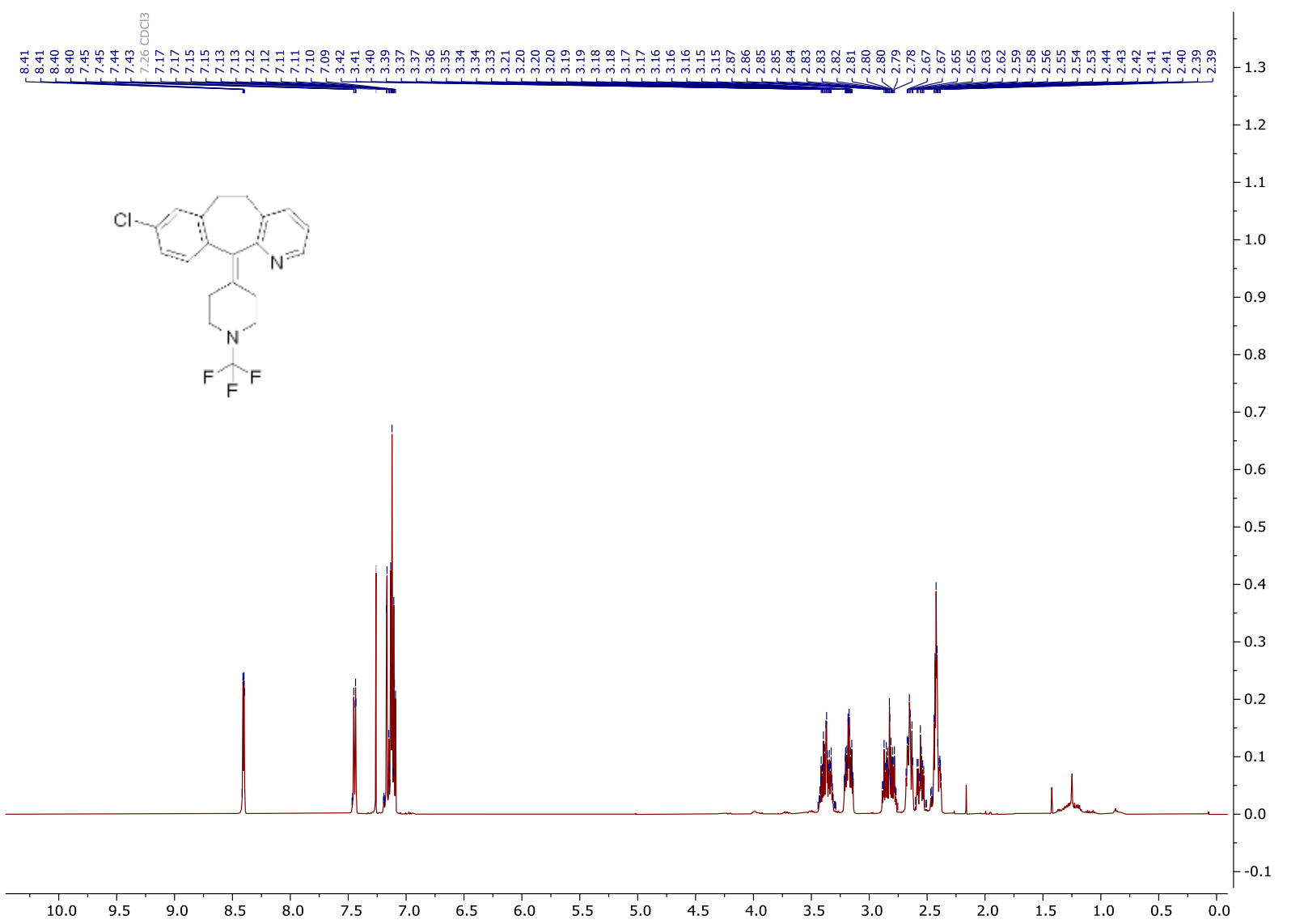

$^{1}H$ NMR (400 MHz, $CDCl_3$) of **3p**.

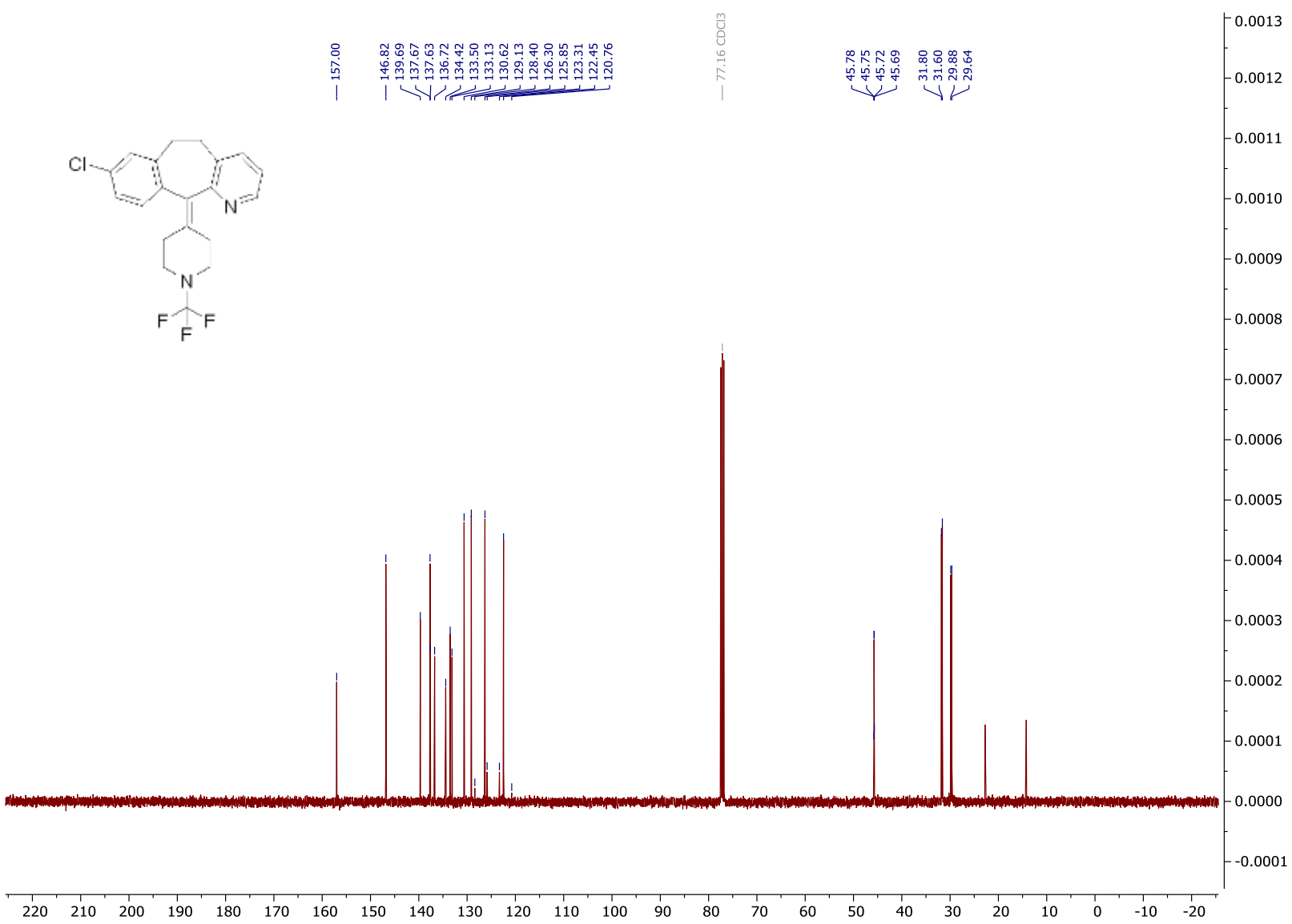

$^{13}C$ NMR (101 MHz, $CDCl_3$) of **3p**.

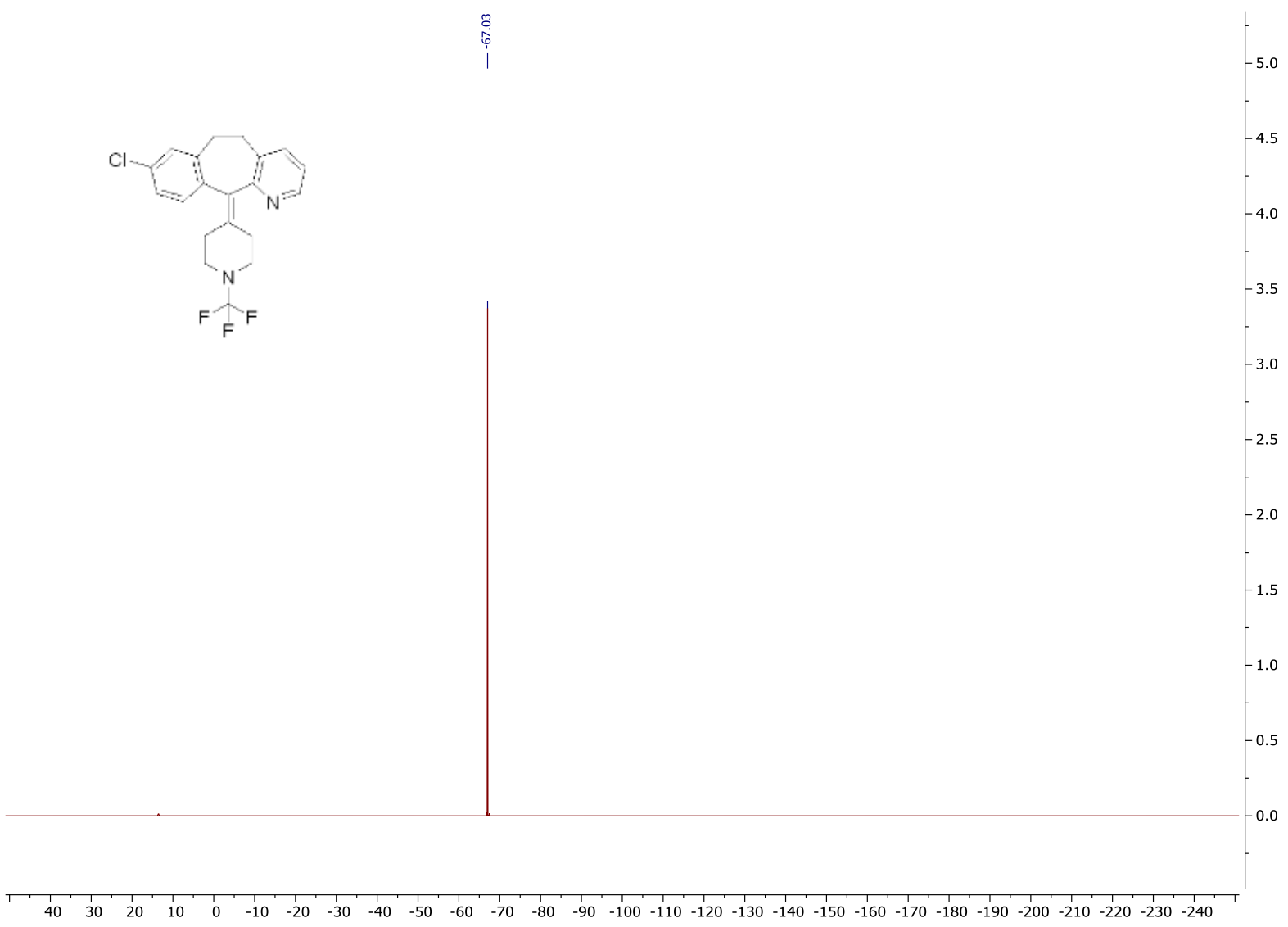


$^{19}$F NMR (376 MHz, $CDCl_3$) of **3p**.

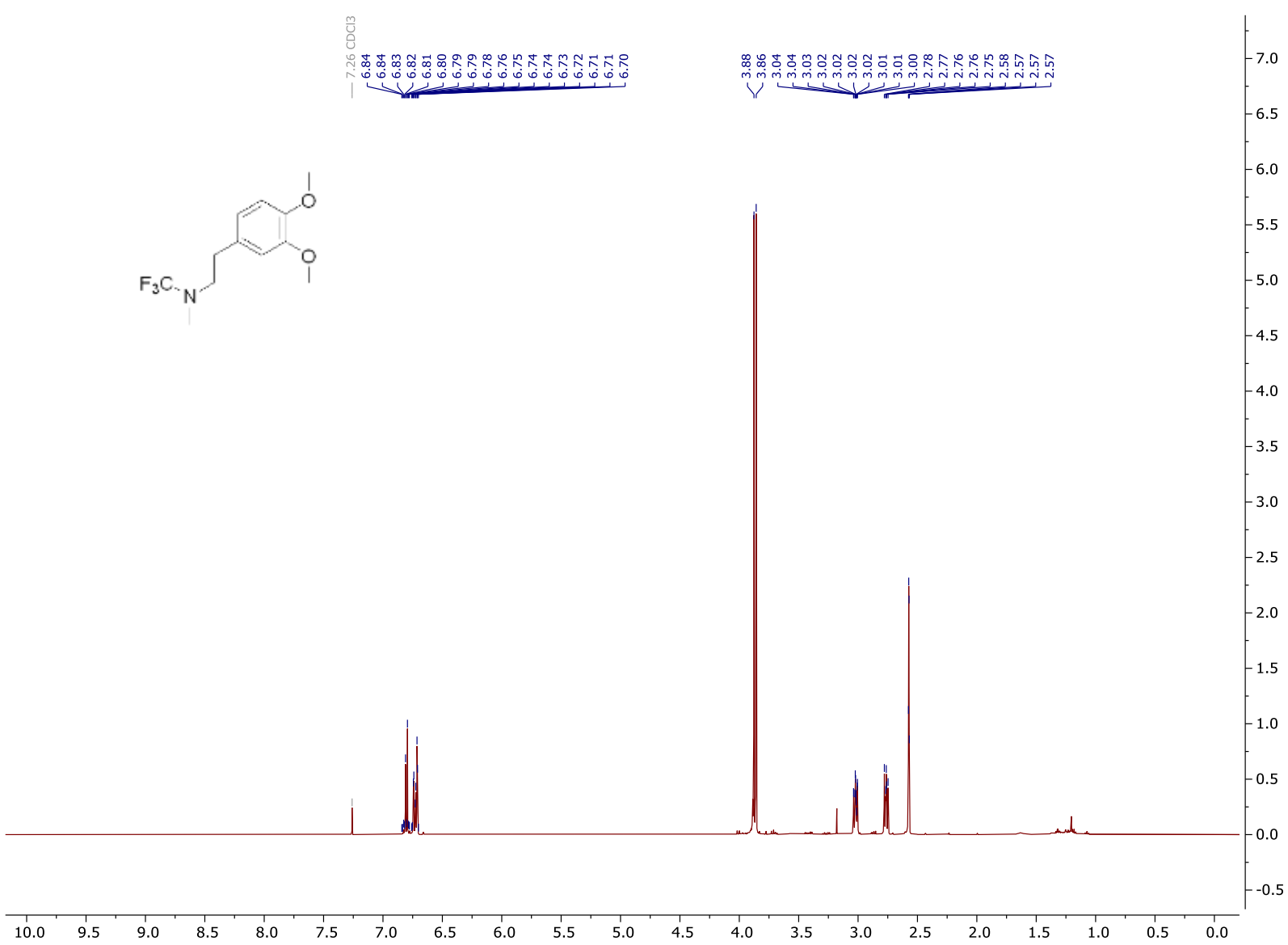


$^{1}$H NMR (400 MHz, $CDCl_3$) of **3q**.

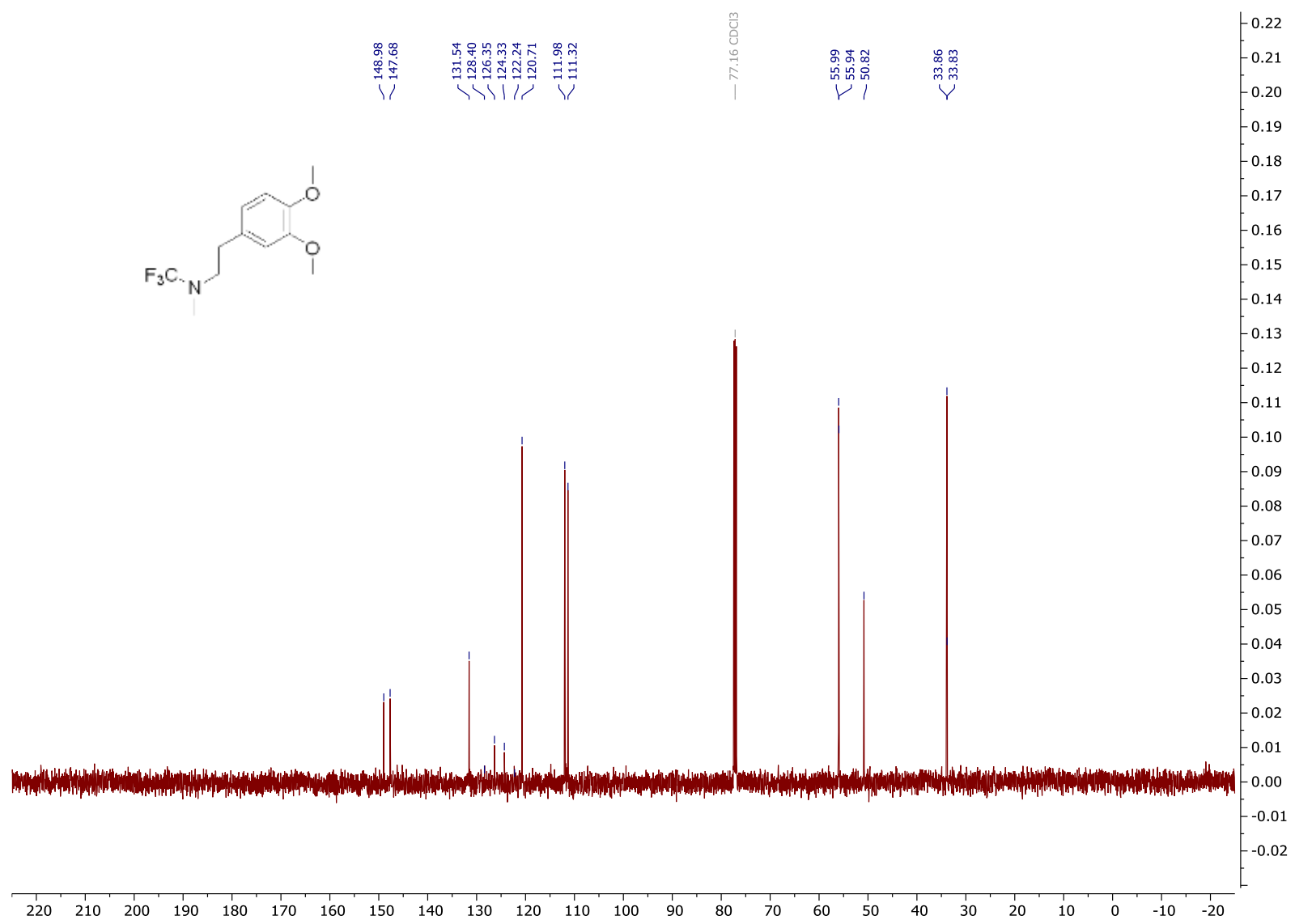

$^{13}$C NMR (101 MHz, CDCl$_3$) of **3q**.

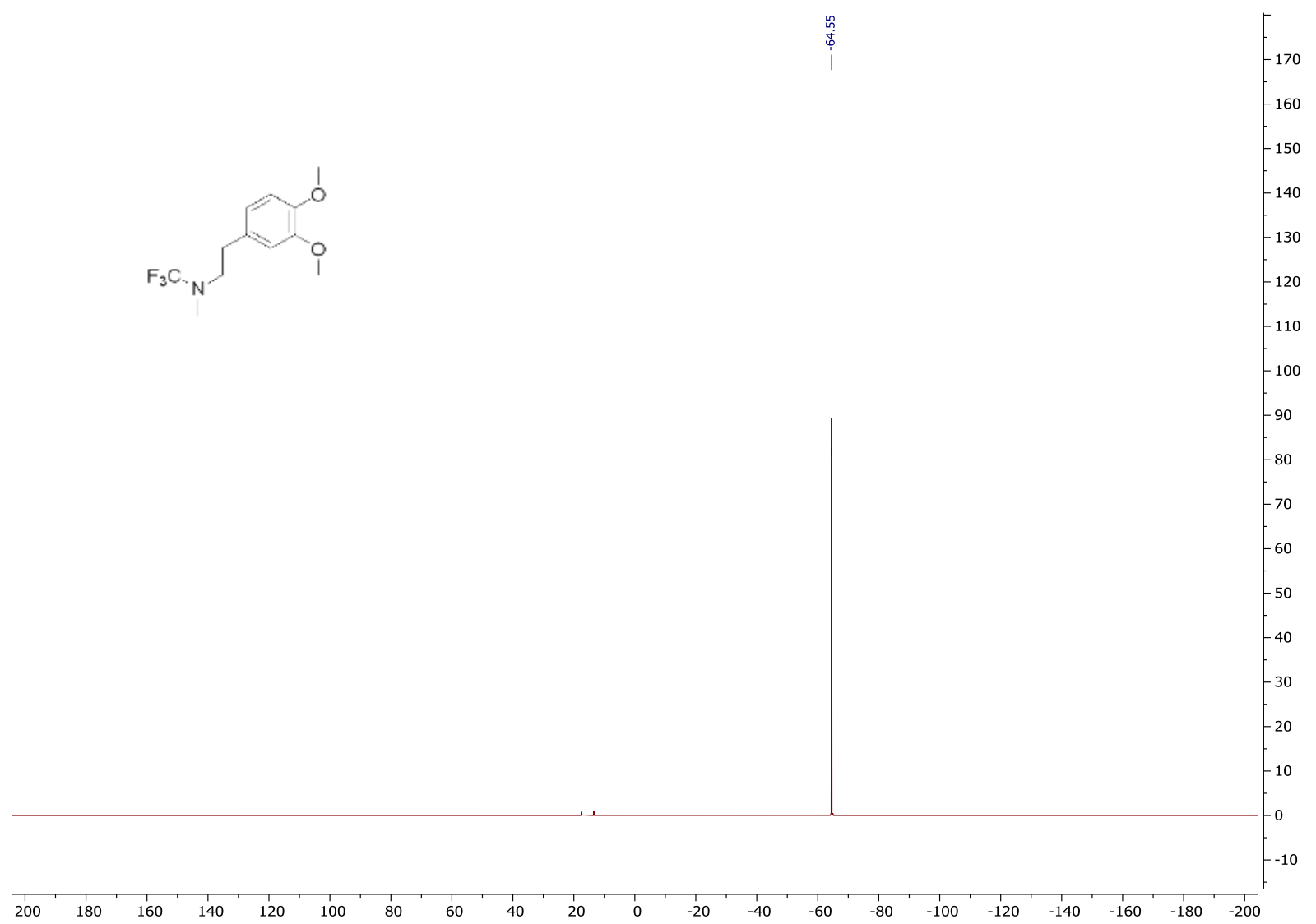

$^{19}$F NMR (376 MHz, CDCl$_3$) of **3q**.

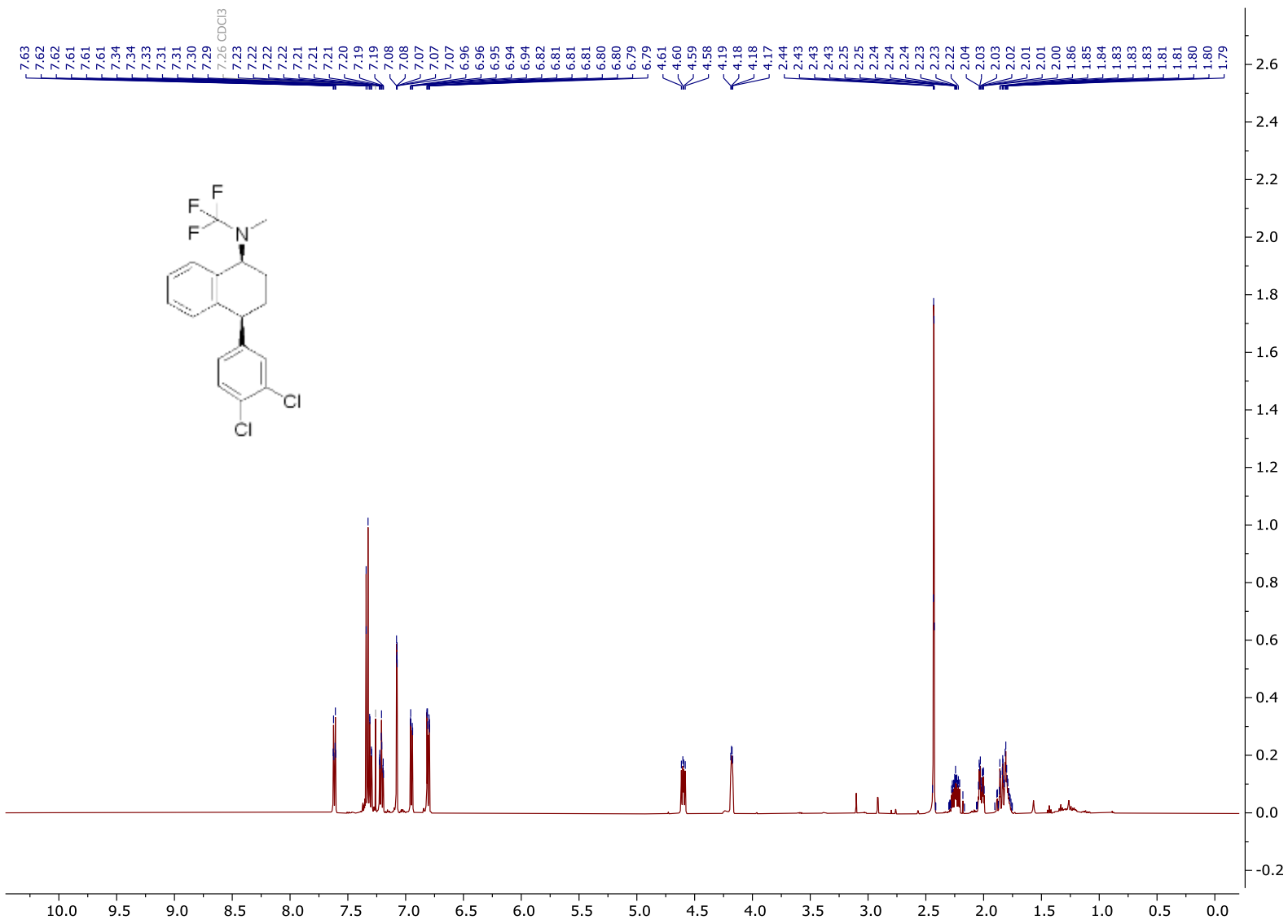

$^{1}$H NMR (400 MHz, $CDCl_3$) of **3r**.

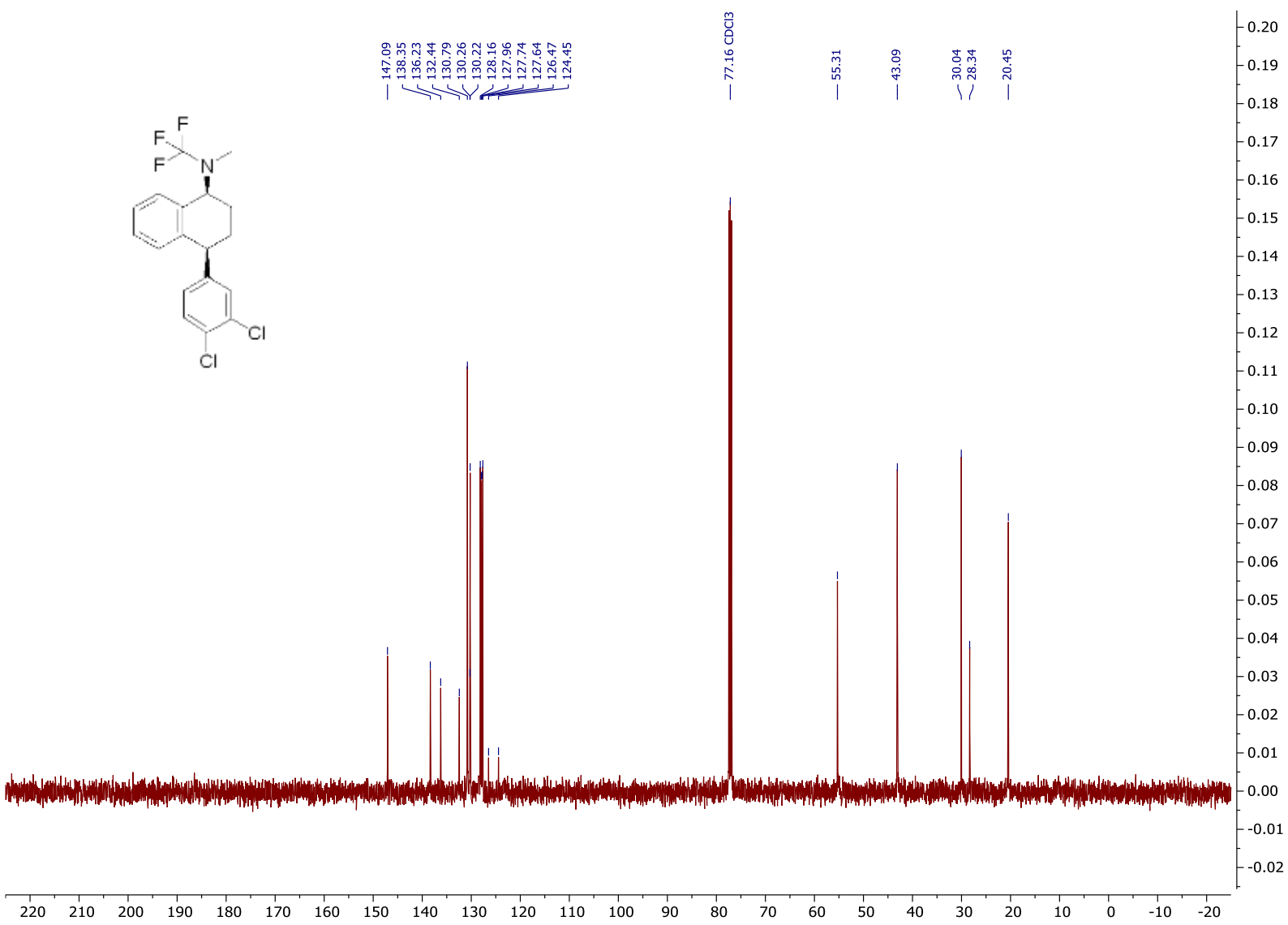

$^{13}$C NMR (101 MHz, $CDCl_3$) of **3r**.

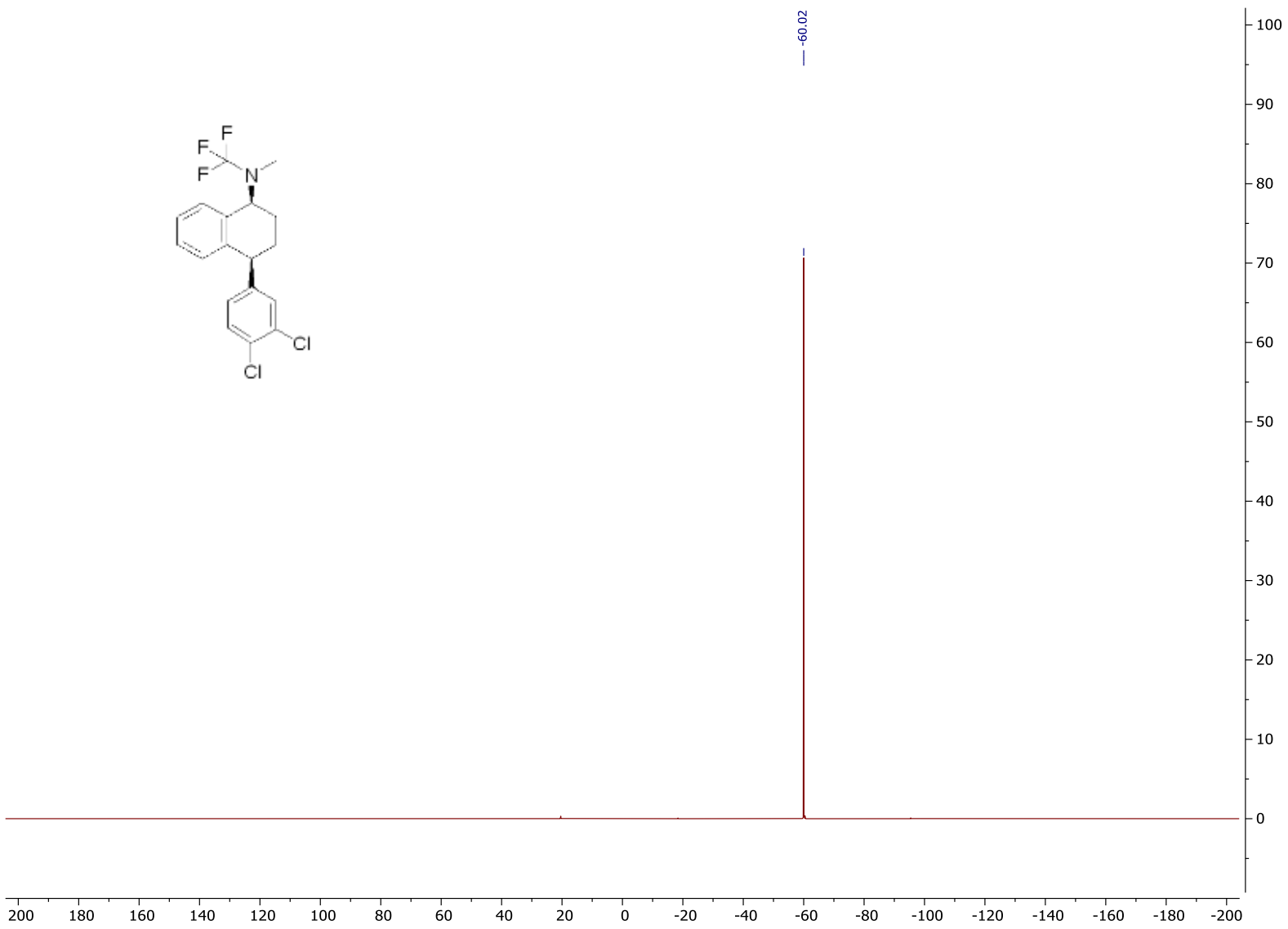

$^{19}$F NMR (376 MHz, $CDCl_3$) of **3r**.

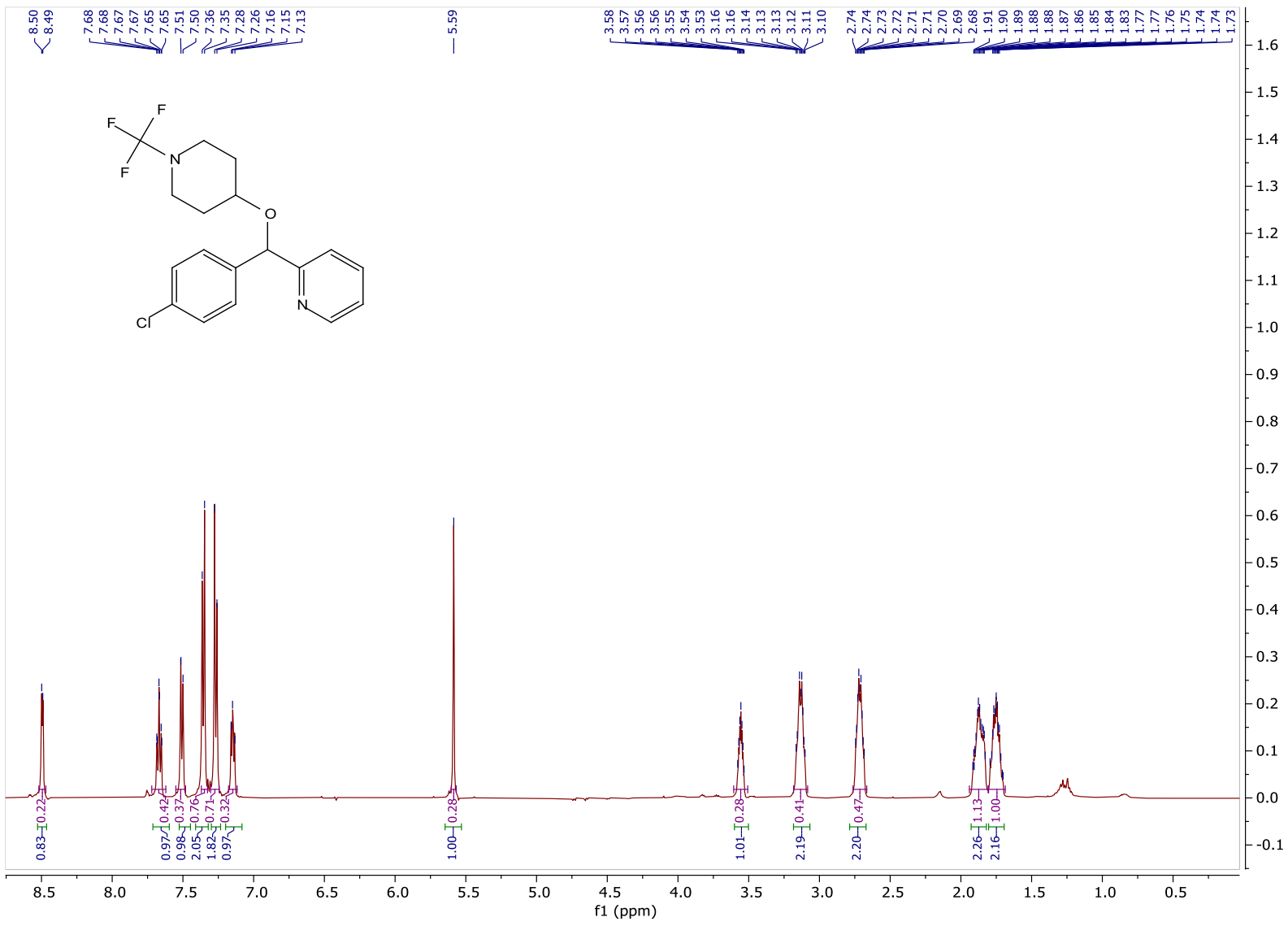

$^{1}$H NMR (400 MHz, $CDCl_3$) of **3s**.

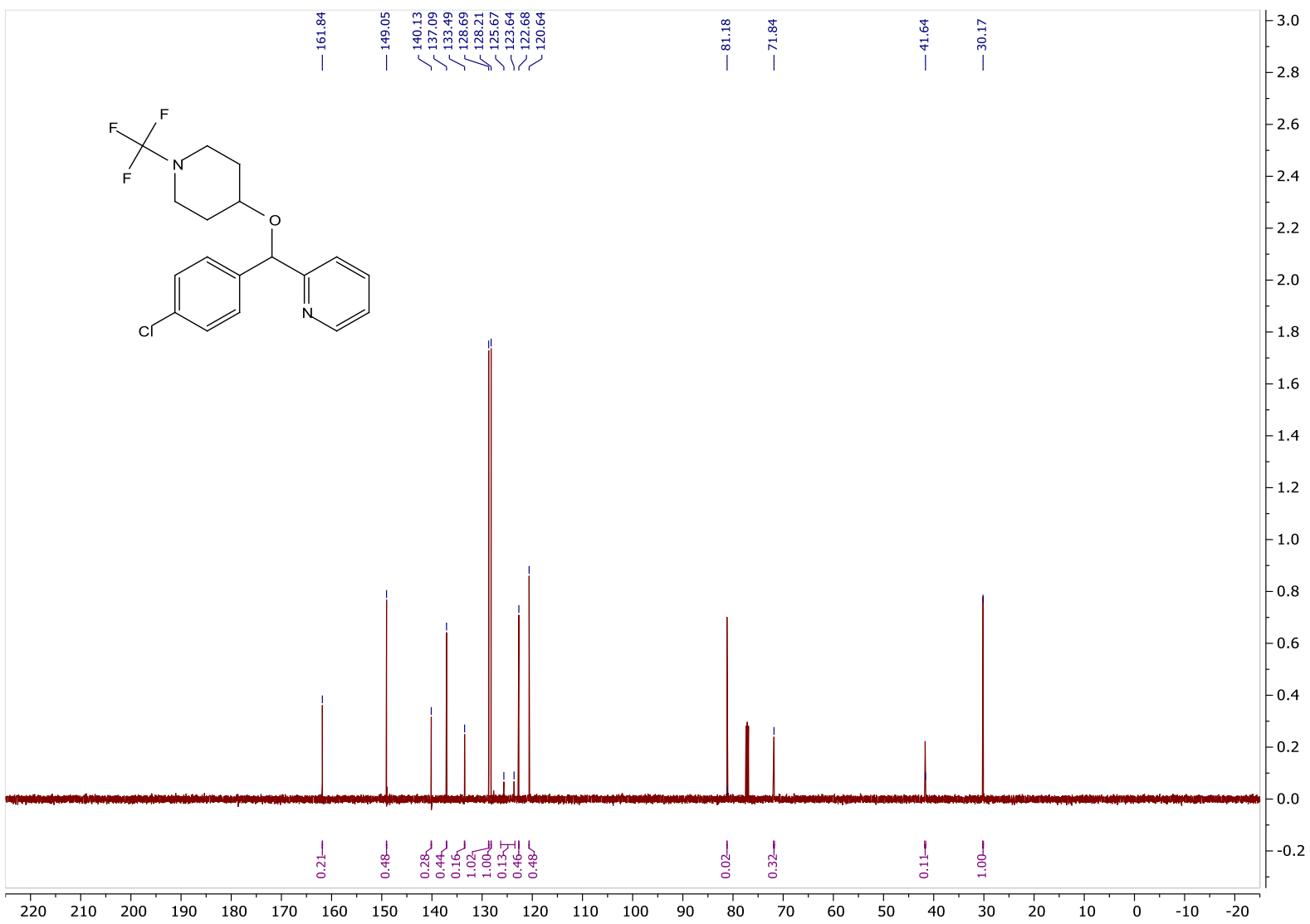

$^{13}C$ NMR (101 MHz, $CDCl_3$) of **3s**.

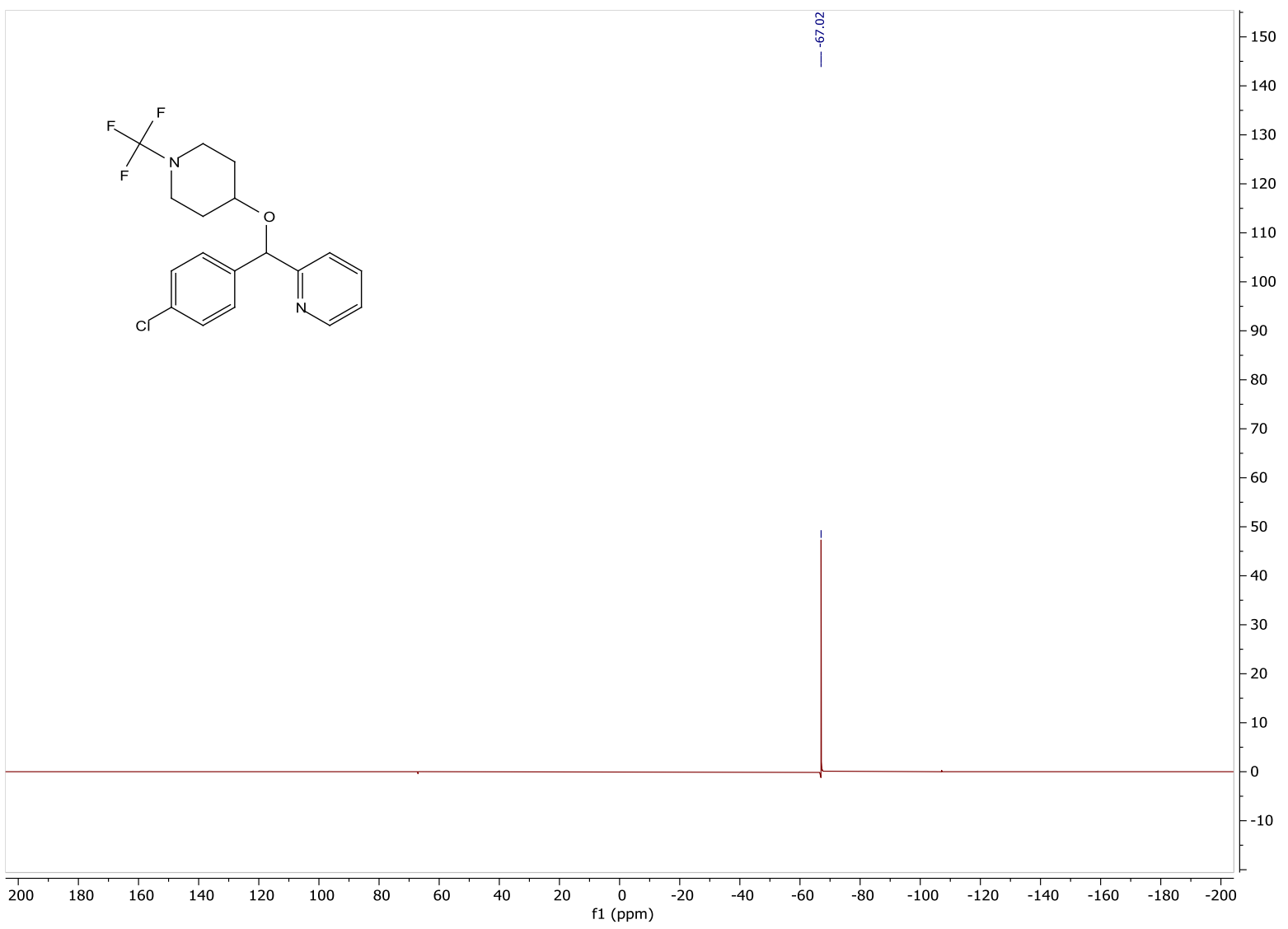


$^{19}F$ NMR (376 MHz, $CDCl_3$) of **3s**.

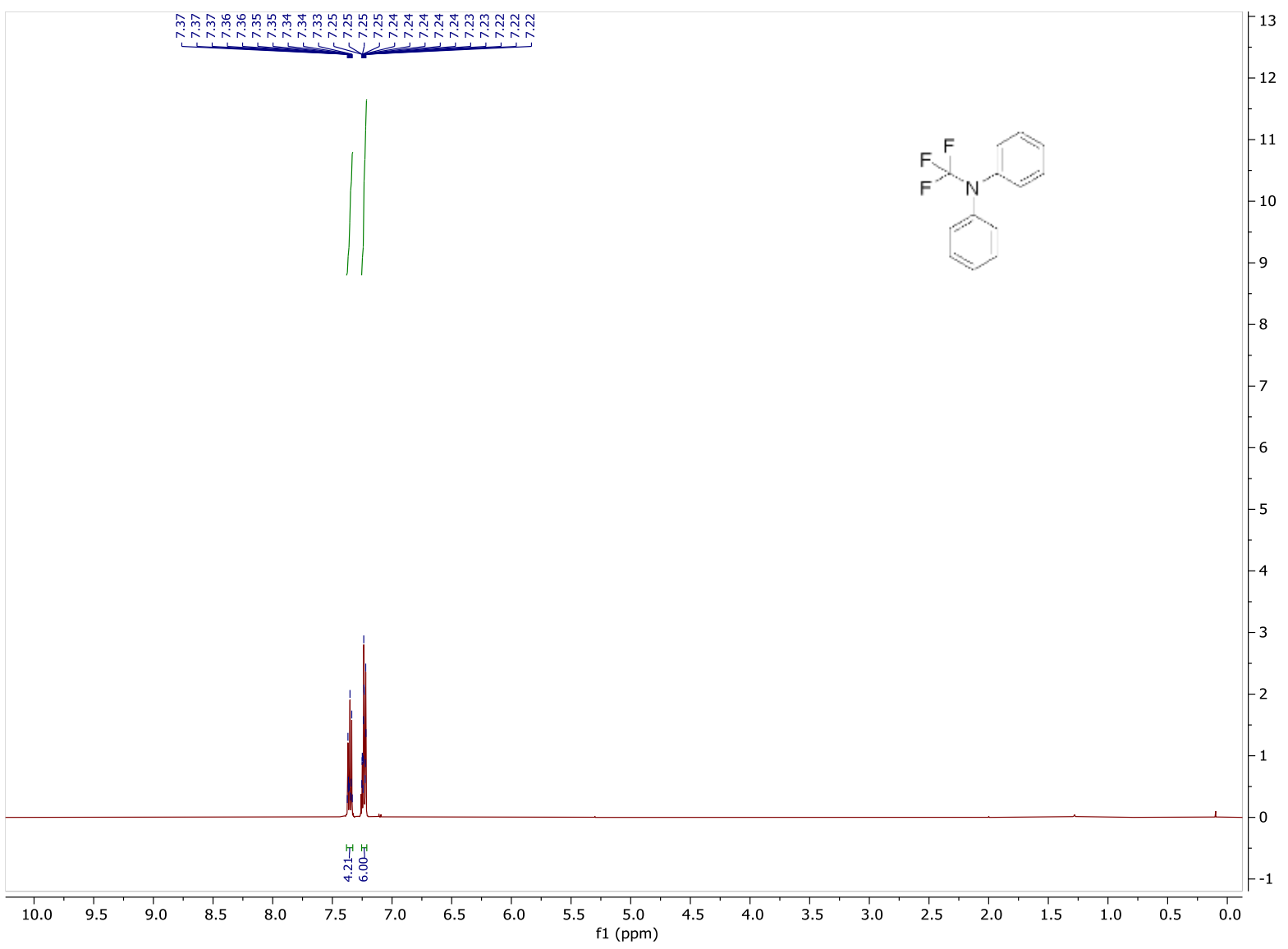


$^{1}H$ NMR (500 MHz, $CDCl_3$) of **3aa.**

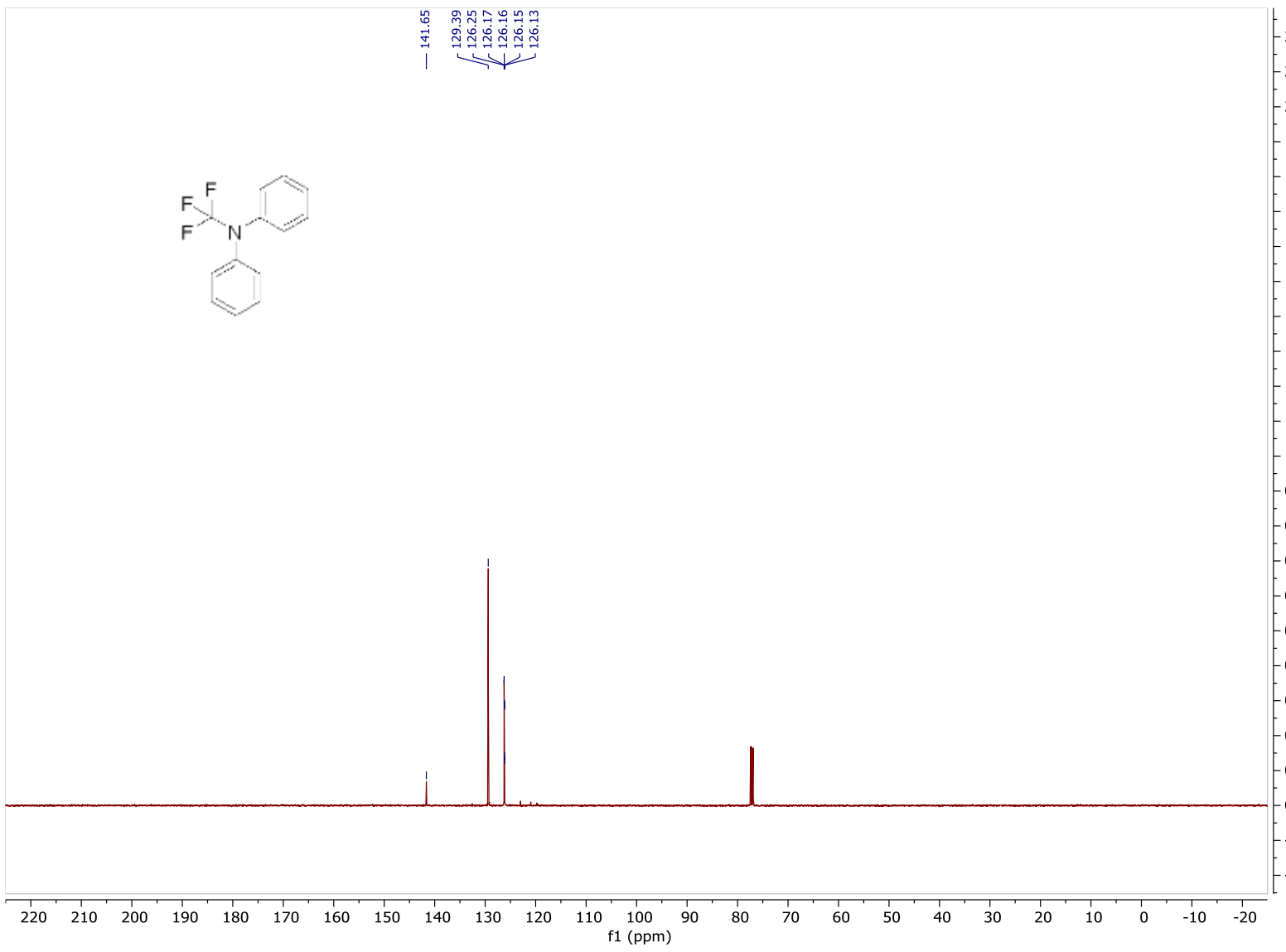

$^{13}C$ NMR (101 MHz, $CDCl_3$) of **3aa.**

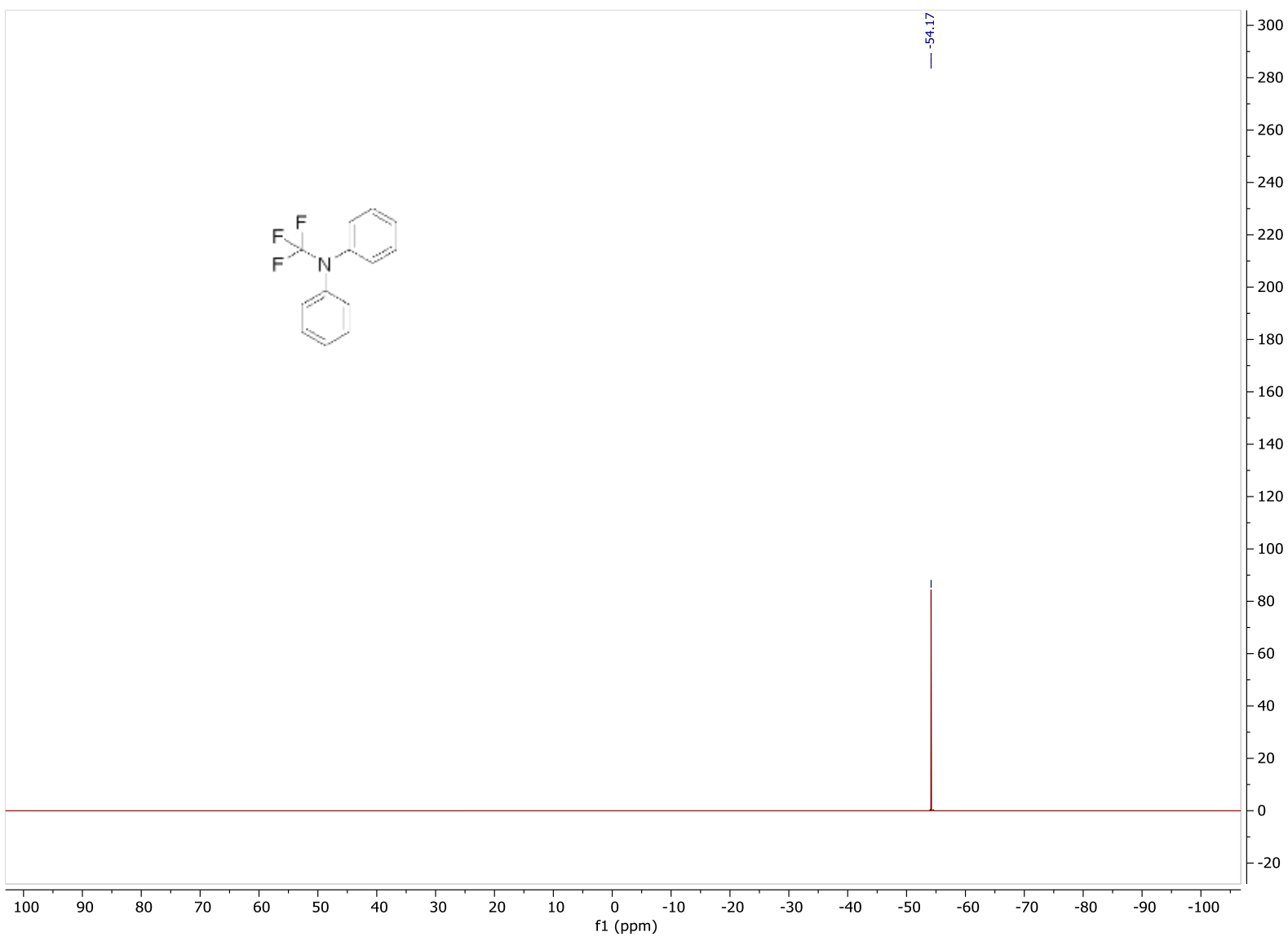

$^{19}F$ NMR (367 MHz, $CDCl_3$) of **3aa.**

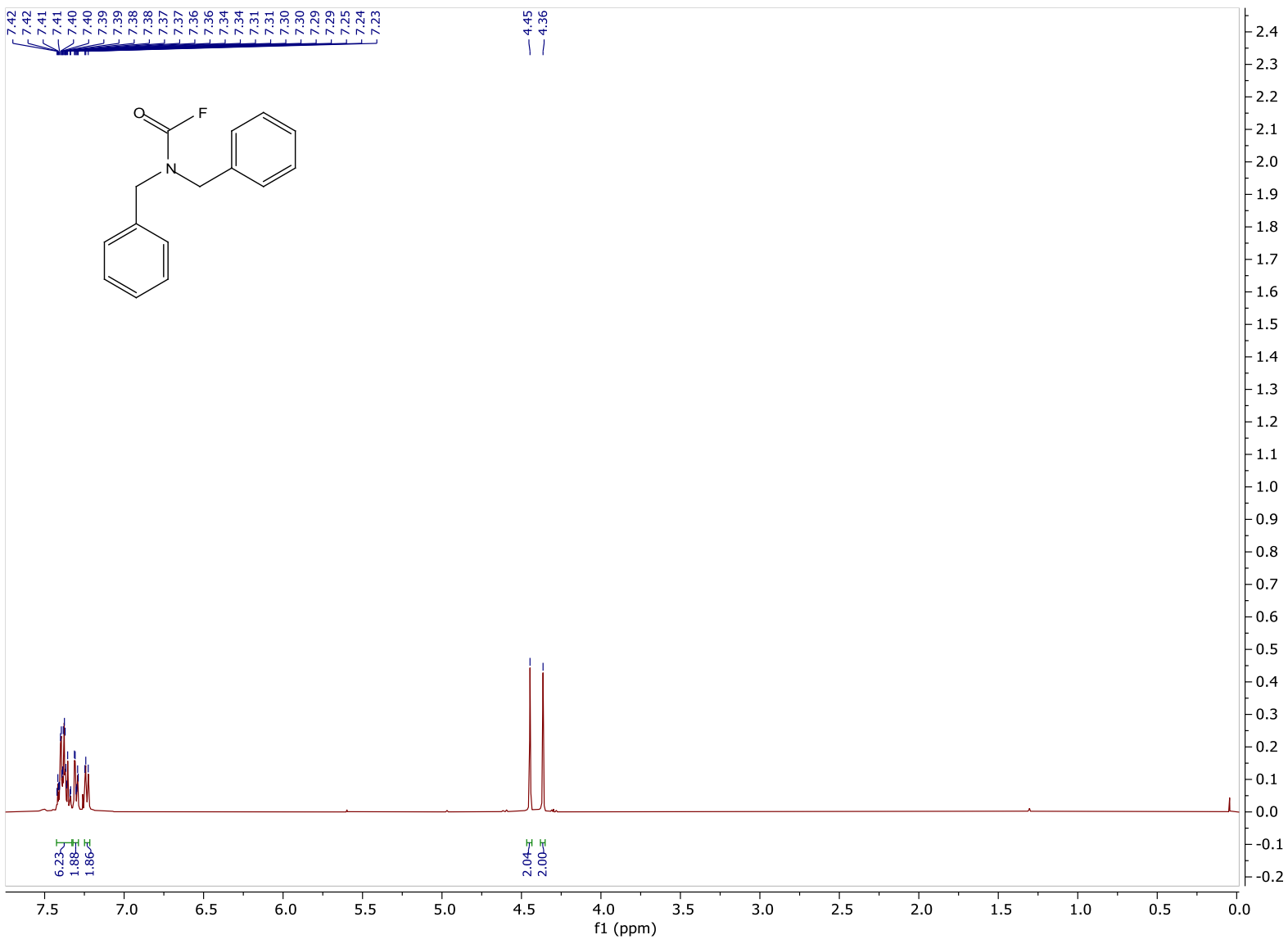

$^{1}H$ NMR (400 MHz, $CDCl_3$) of **4a**

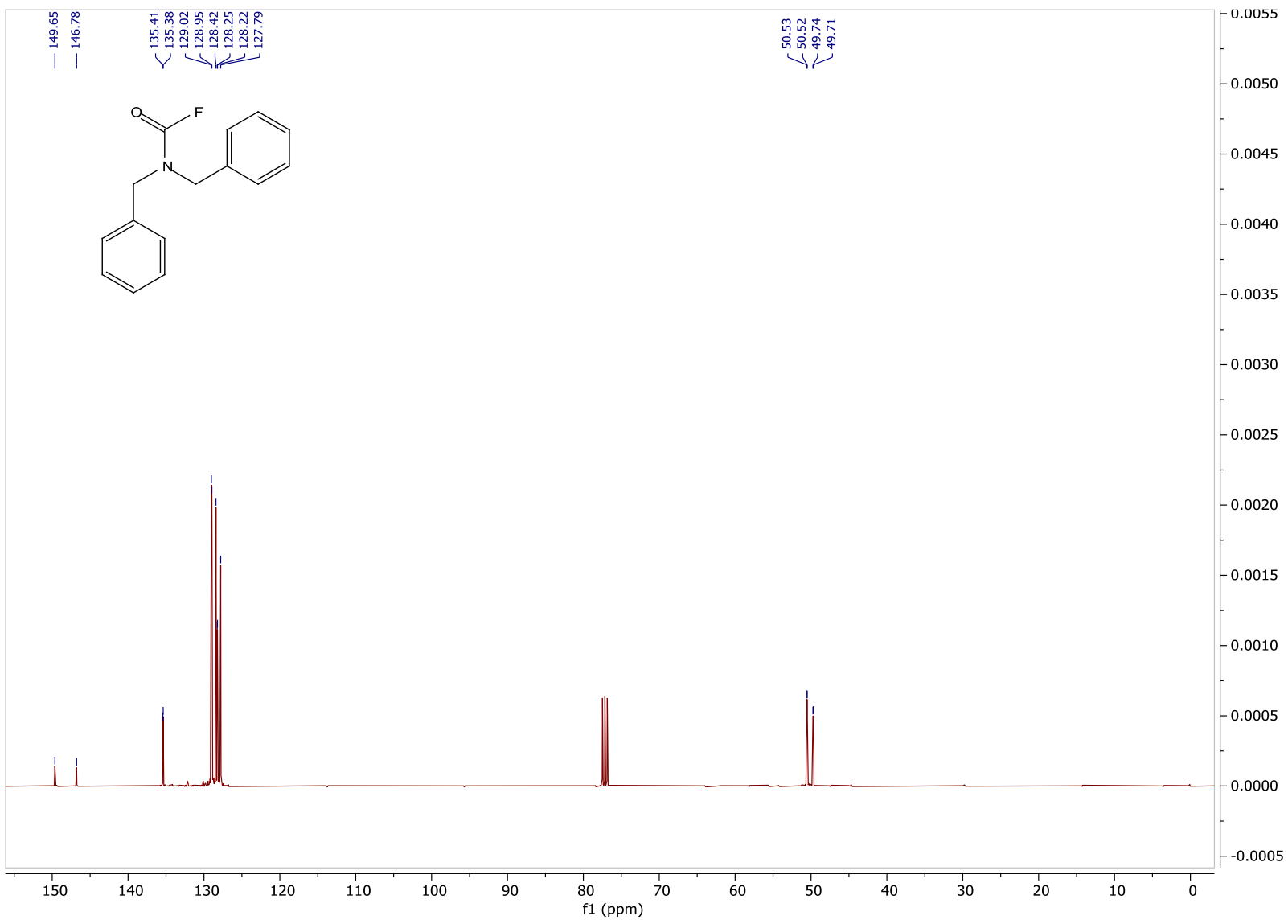

$^{13}C$ NMR (101 MHz, $CDCl_3$) of **4a**.

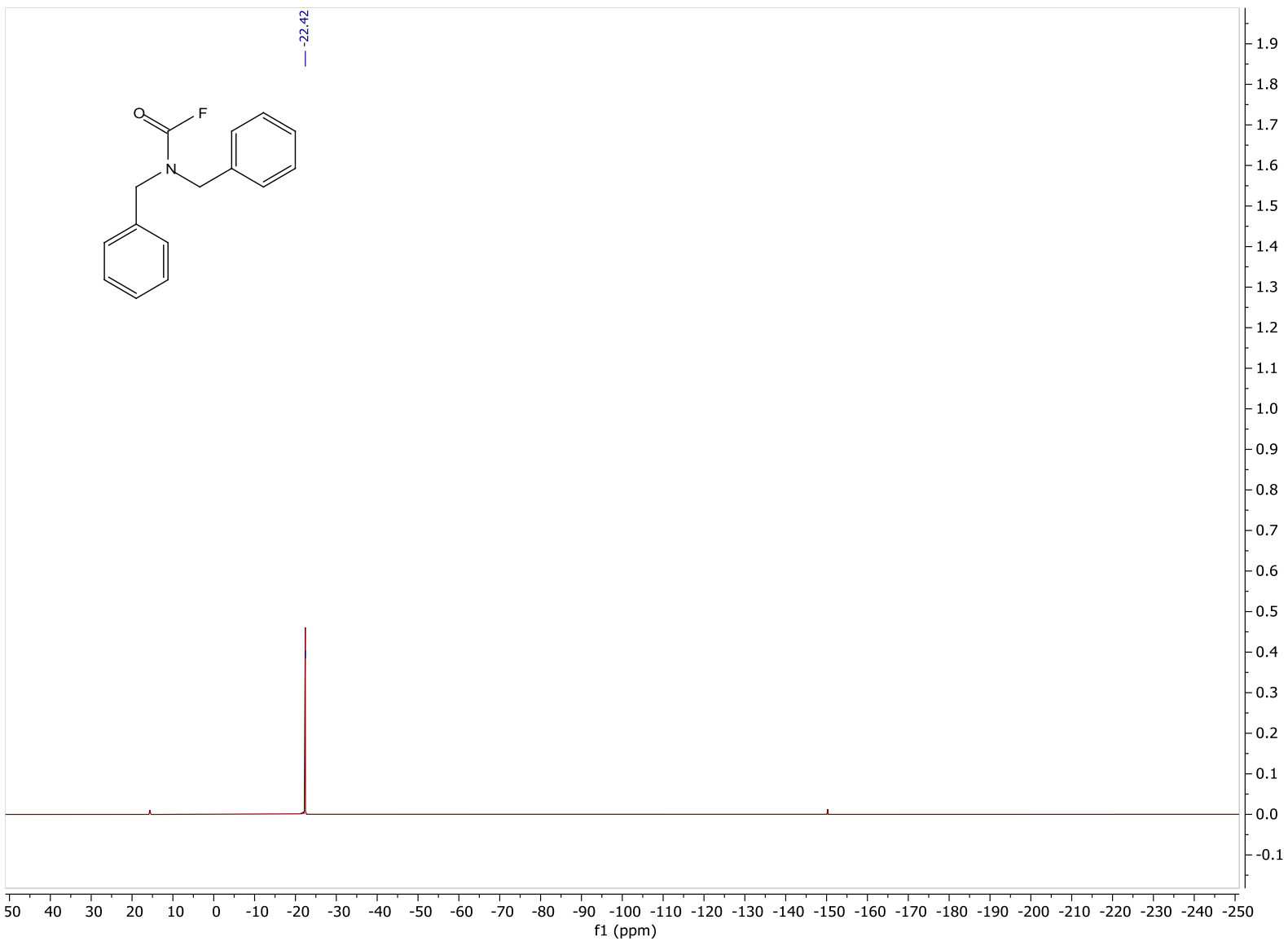

$^{19}F$ NMR (376 MHz, $CDCl_3$) of **4a**.

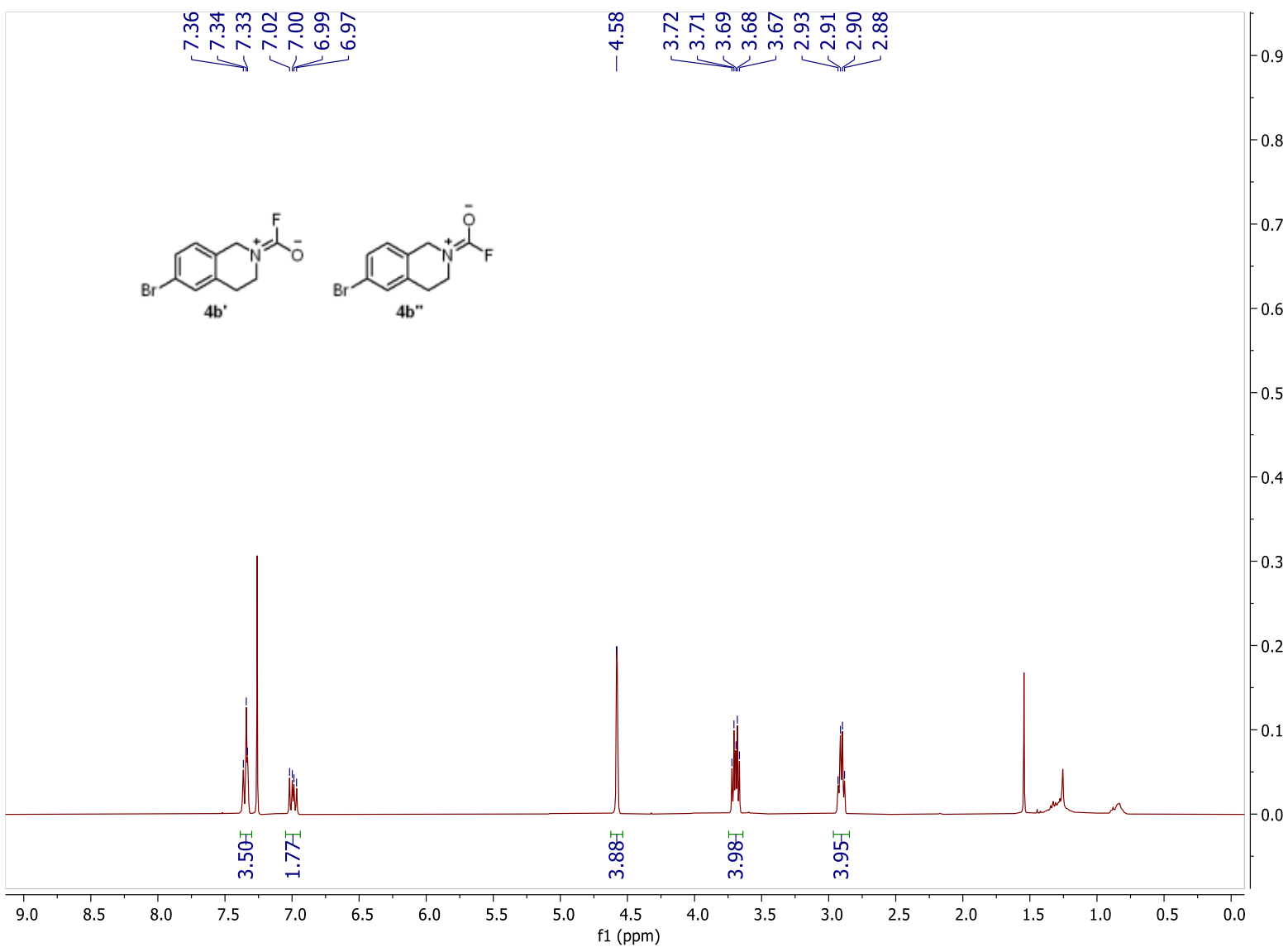

$^{1}H$ NMR (400 MHz, $CDCl_3$) of **4b'** and **4b''**.

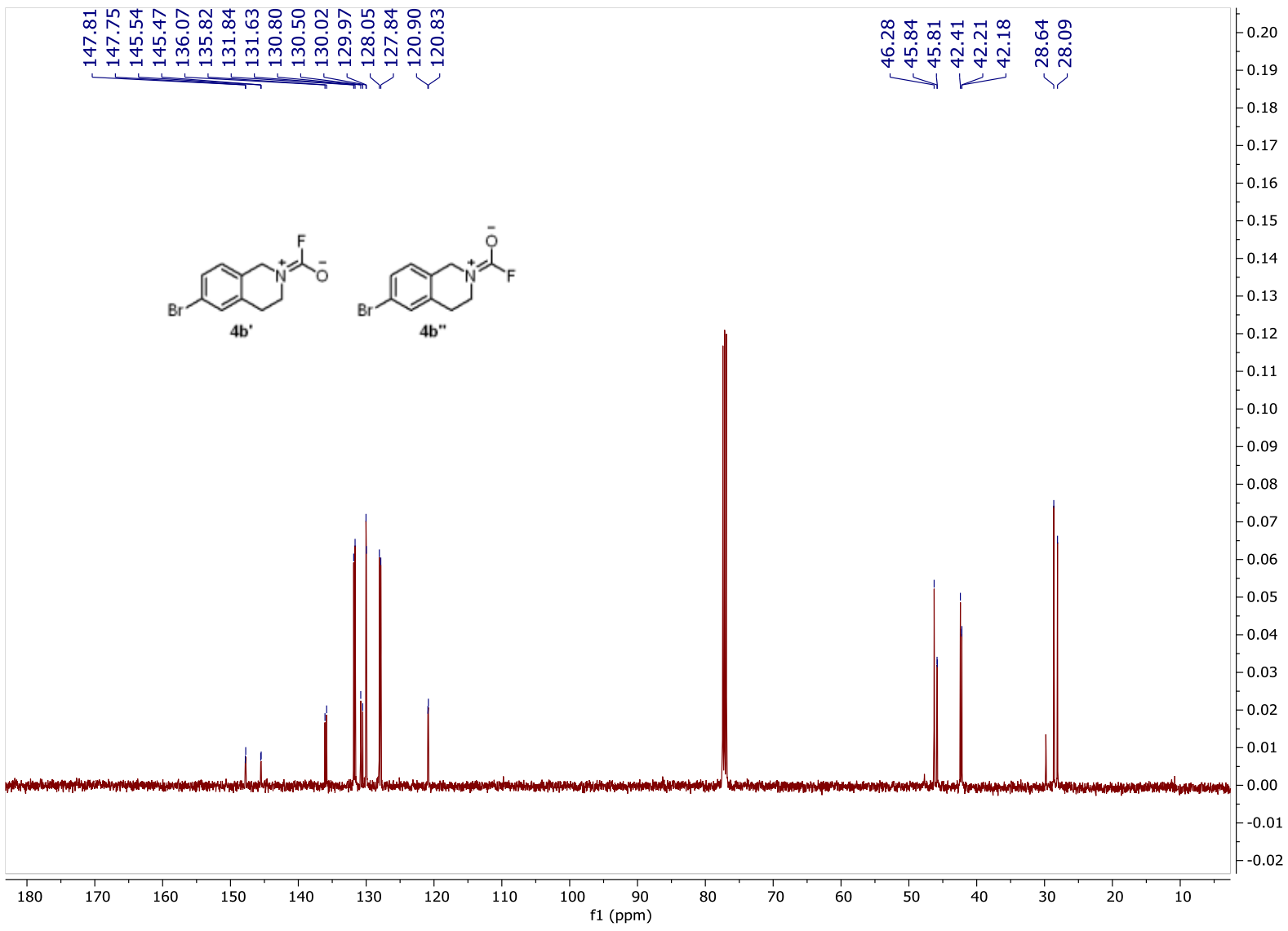


$^{13}$C NMR (126 MHz, $CDCl_3$) of **4b'** and **4b''**.

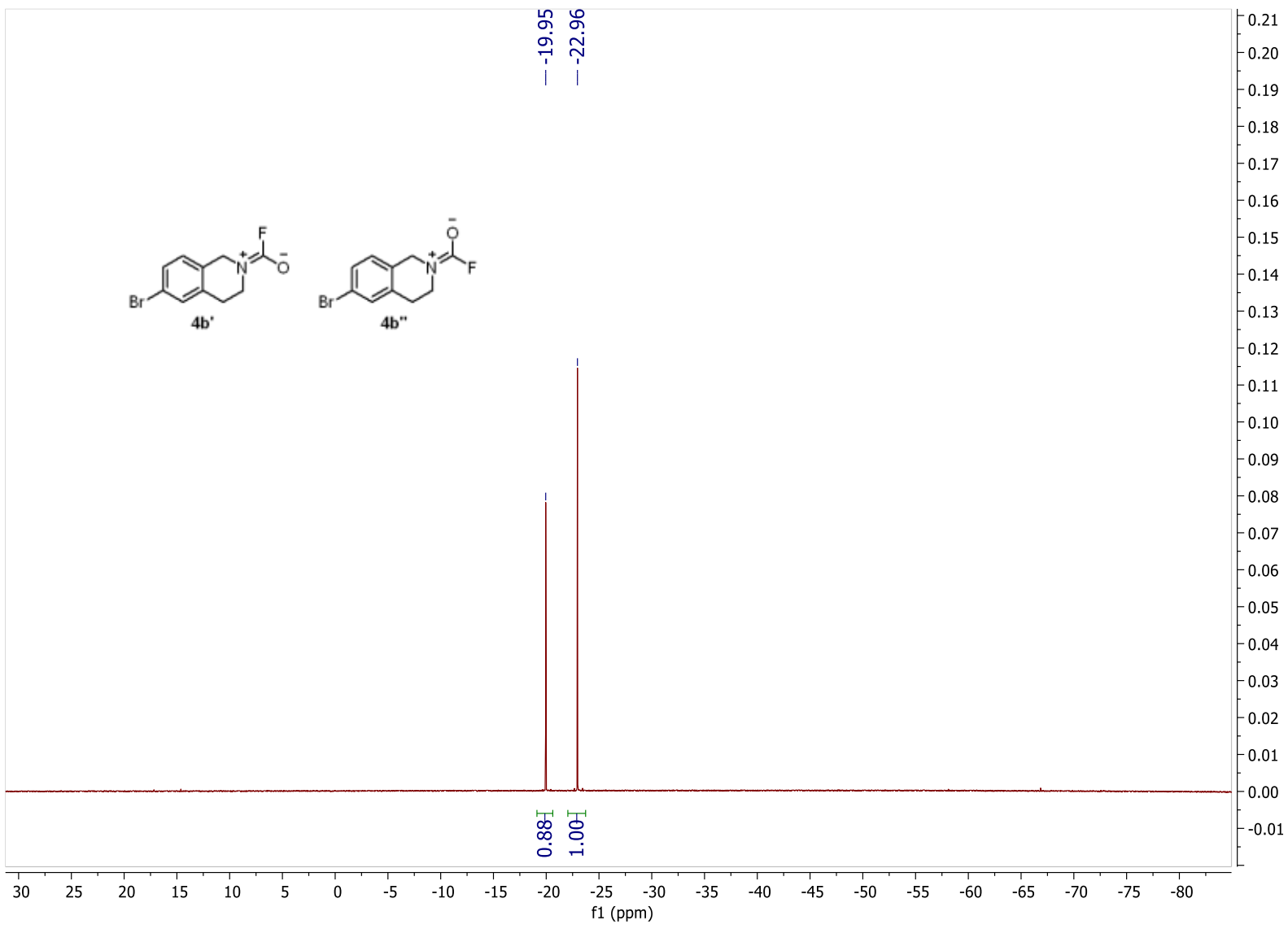


$^{19}$F NMR (376 MHz, $CDCl_3$) of **4b'** and **4b''**.

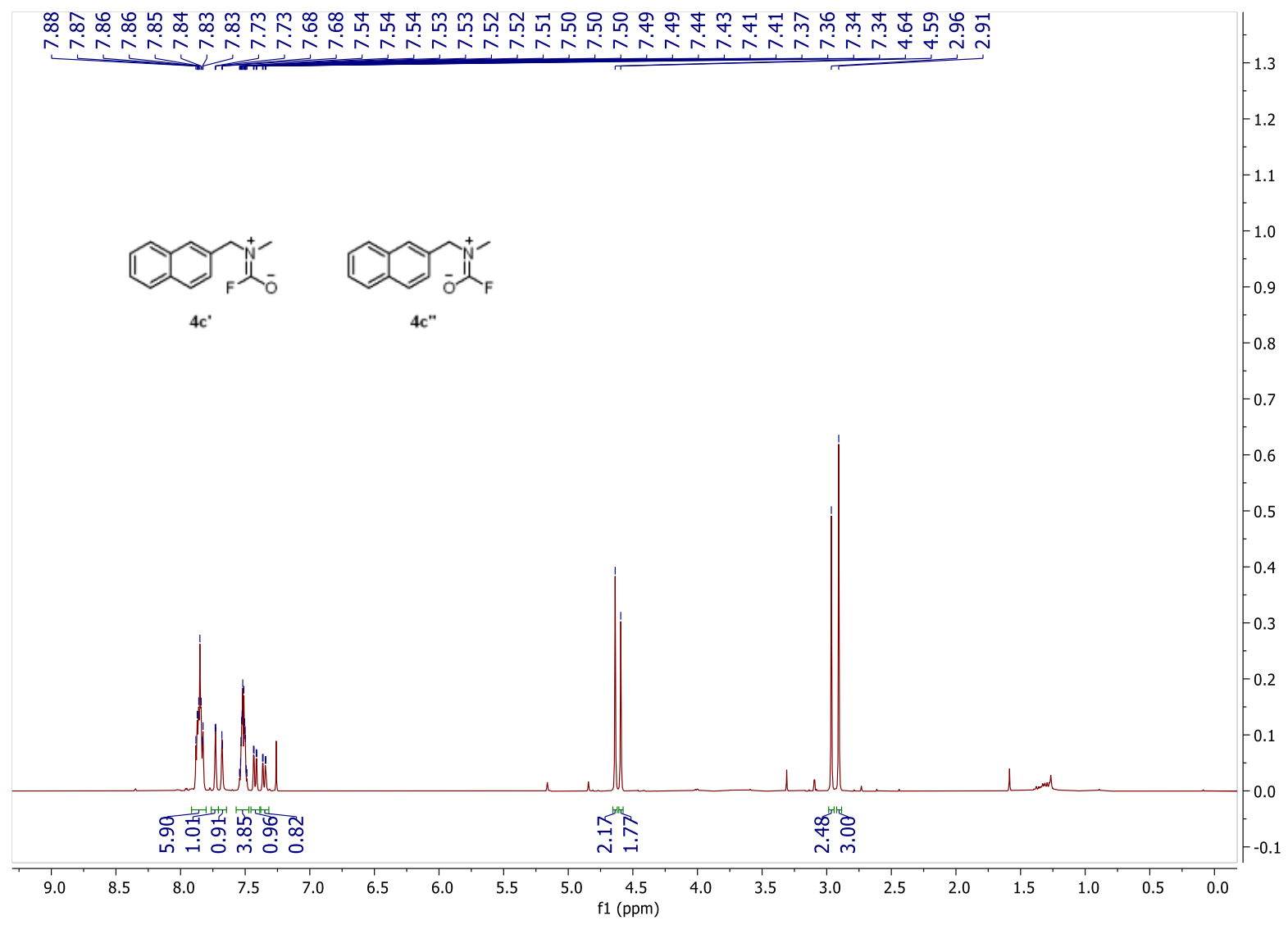

$^{1}$H NMR (400 MHz, $CDCl_3$) of **4c'** and **4c''**.

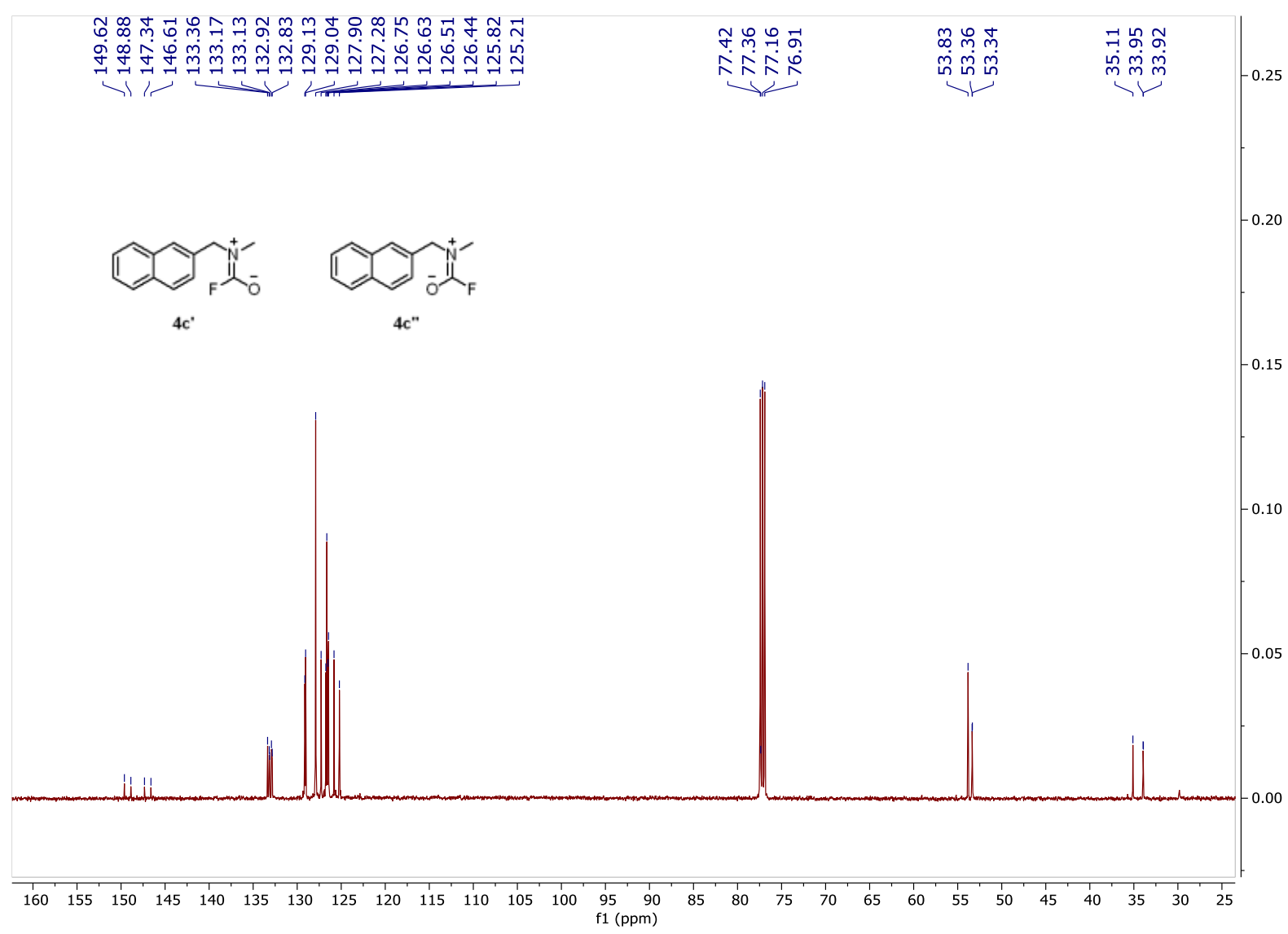

$^{13}$C NMR (126 MHz, $CDCl_3$) of **4c'** and **4c''**.

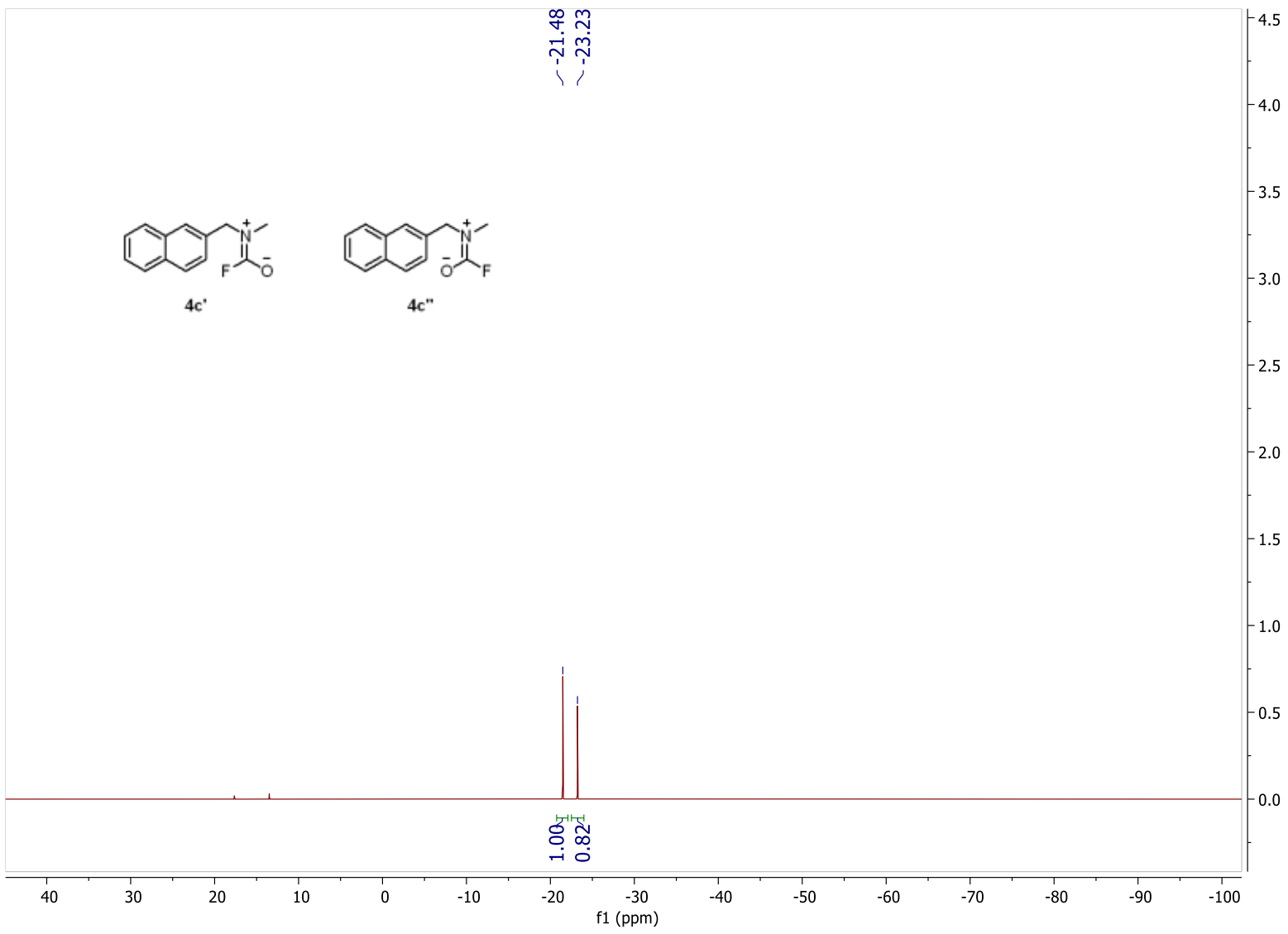

$^{19}$F NMR (376 MHz, $CDCl_3$) of **4c’** and **4c’’**.

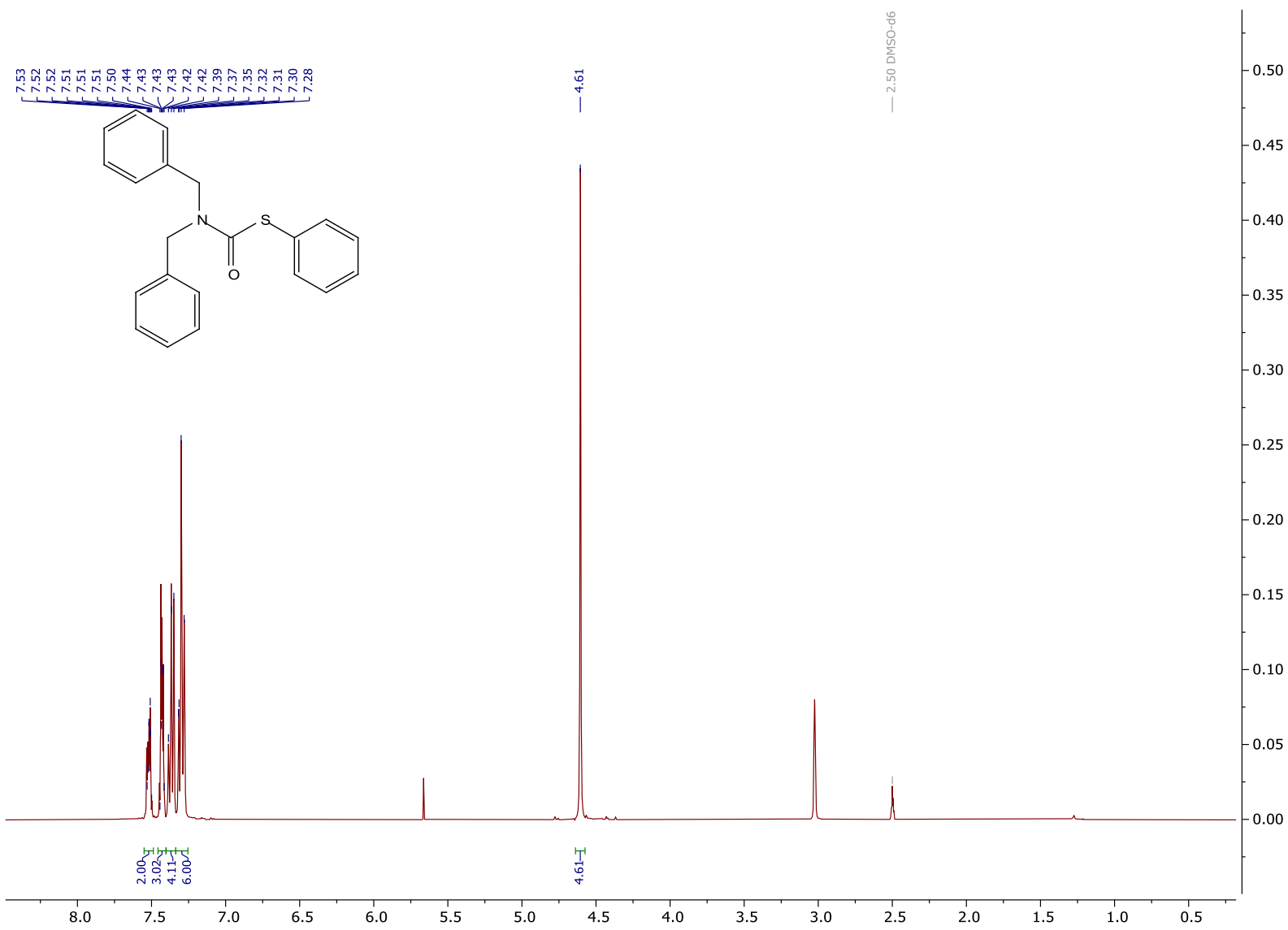

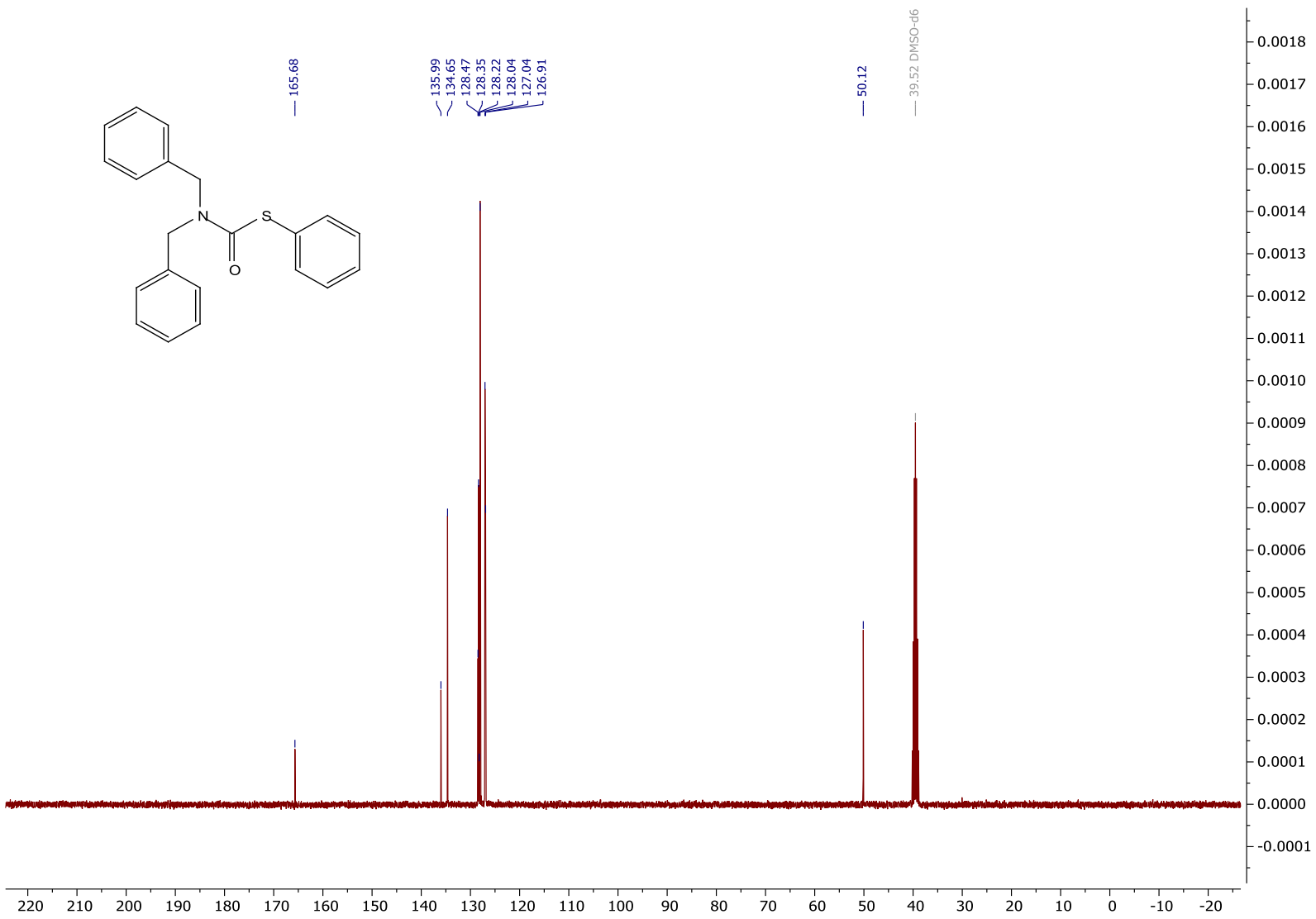


$^{13}$C NMR (101 MHz, DMSO-$D_6$, 100 °C) of **4d**.

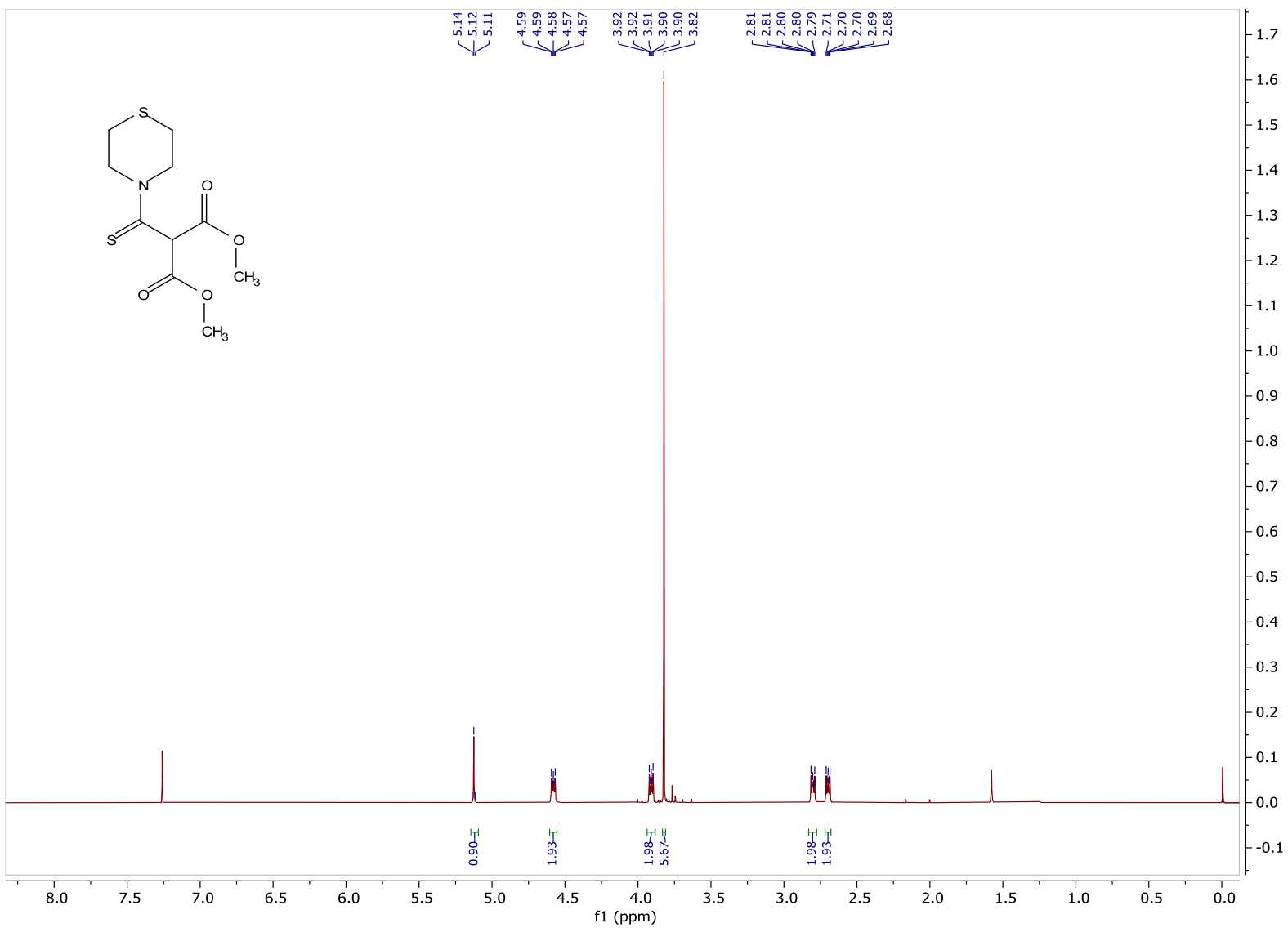


$^{1}$H NMR (400 MHz, $CDCl_3$) of **5a**.

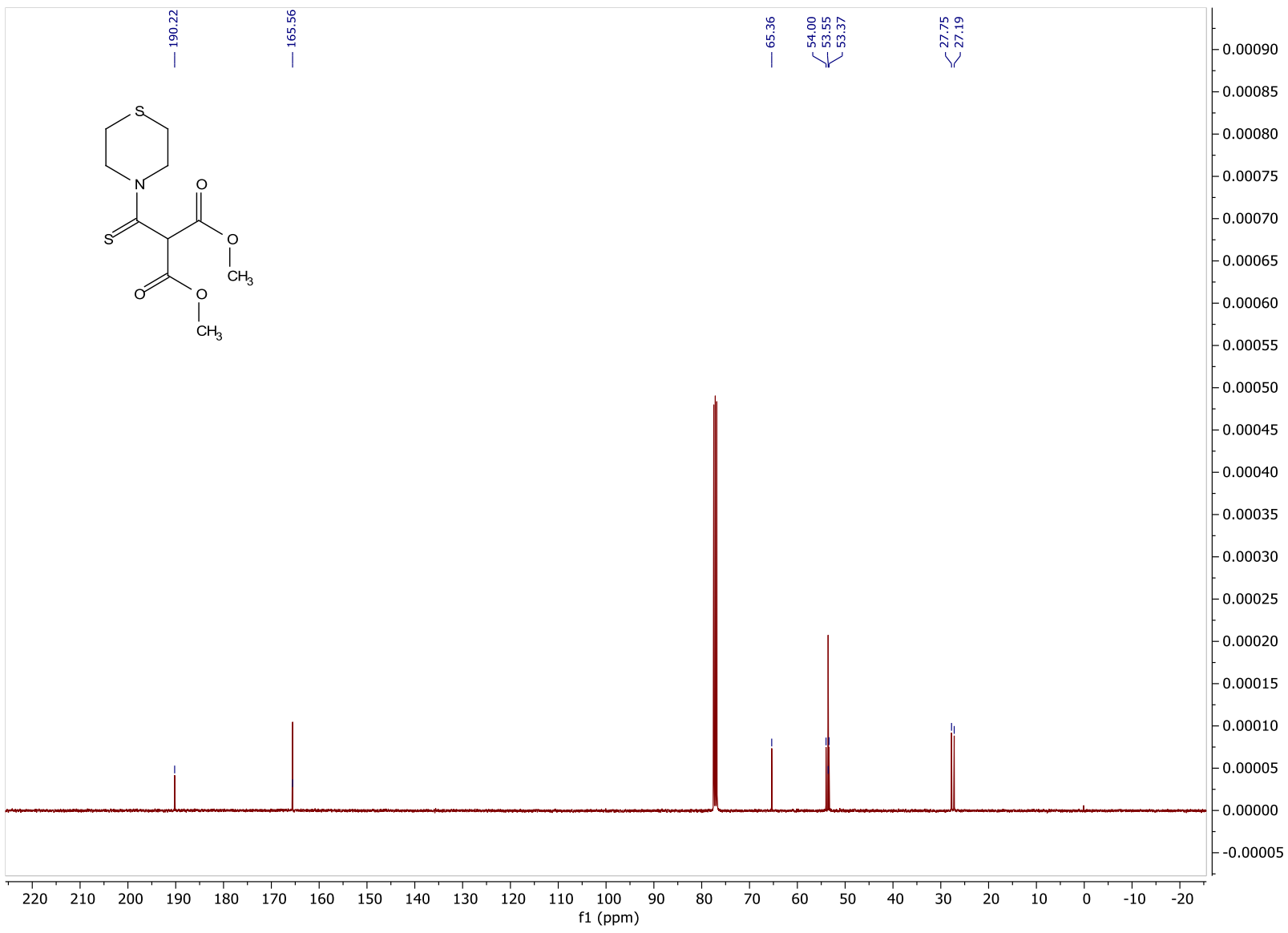

$^{13}C$ NMR (101 MHz, $CDCl_3$) of **5a**.

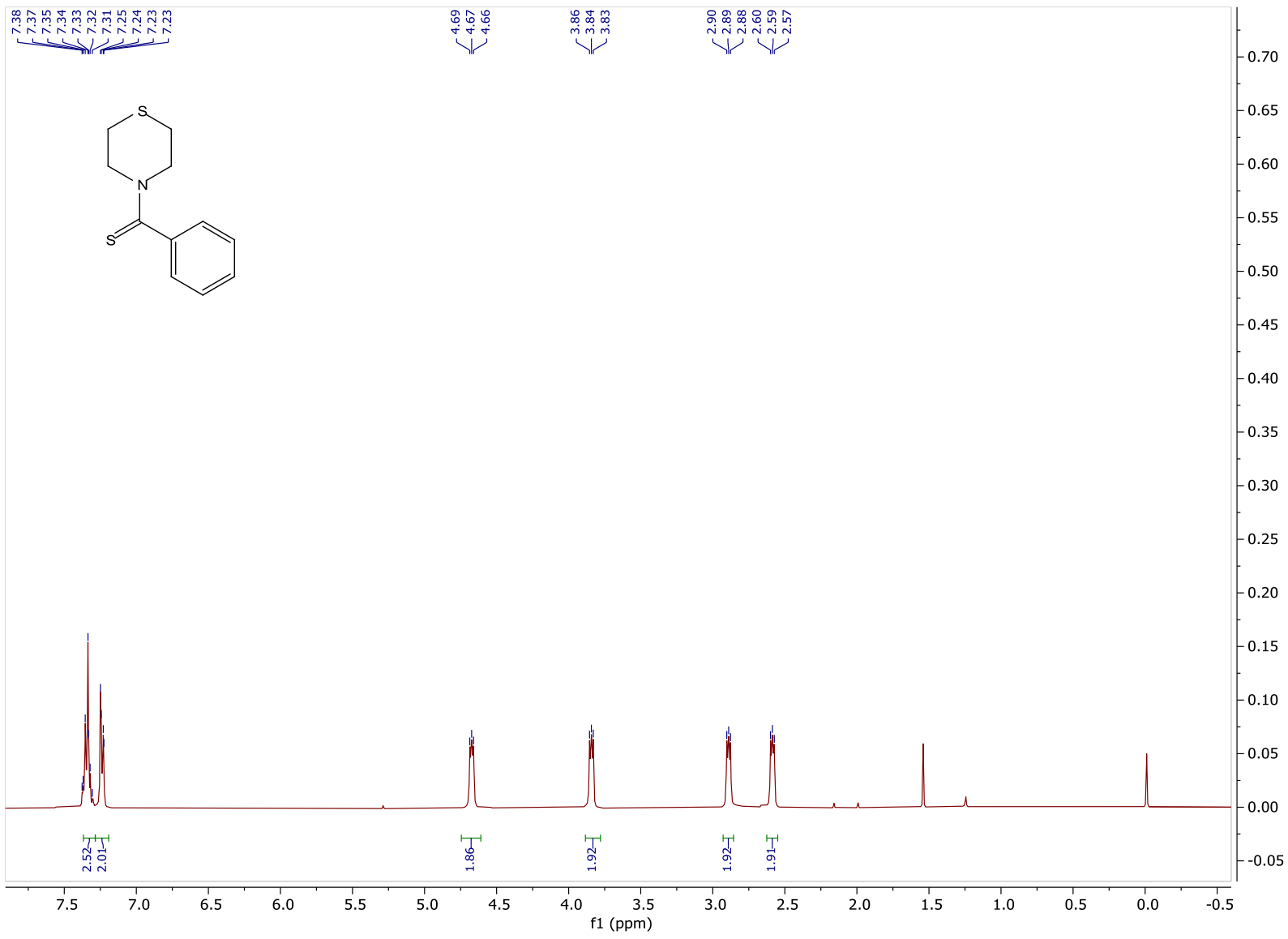

$^{1}H$ NMR (400 MHz, $CDCl_3$) of **5b**.

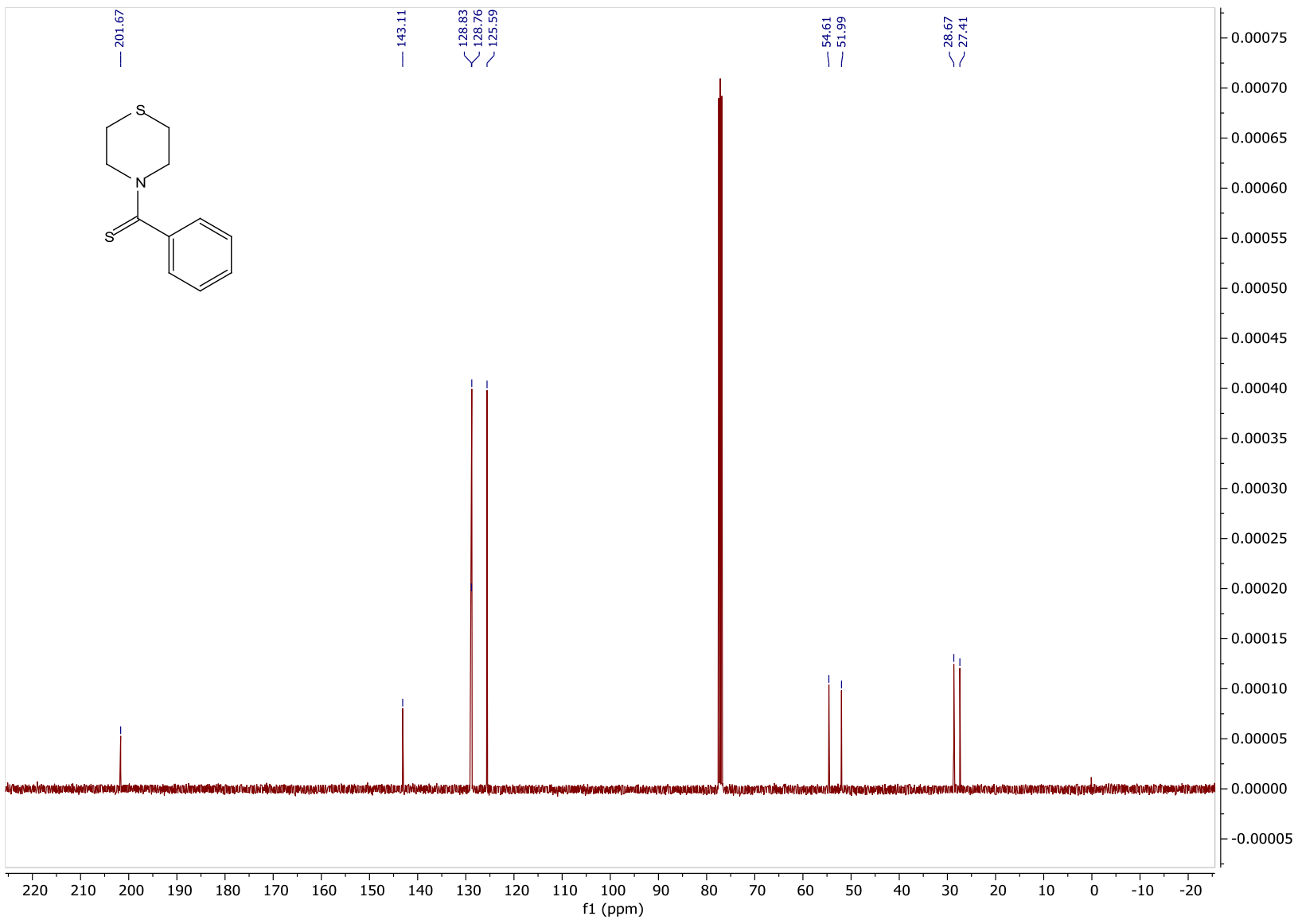

$^{13}C$ NMR (101 MHz, $CDCl_3$) of **5b**.

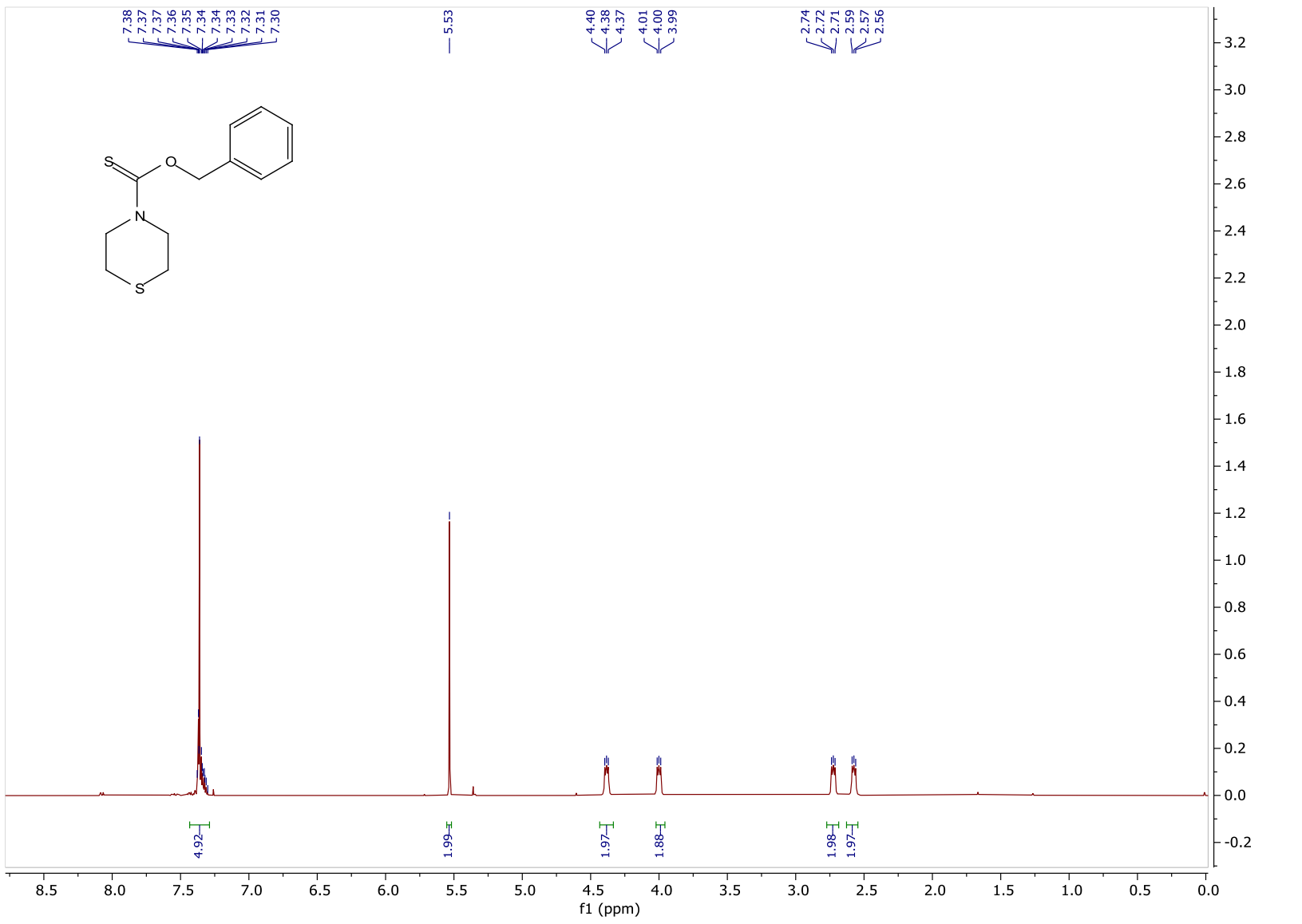

$^{1}H$ NMR (400 MHz, $CDCl_3$) of **7a**.

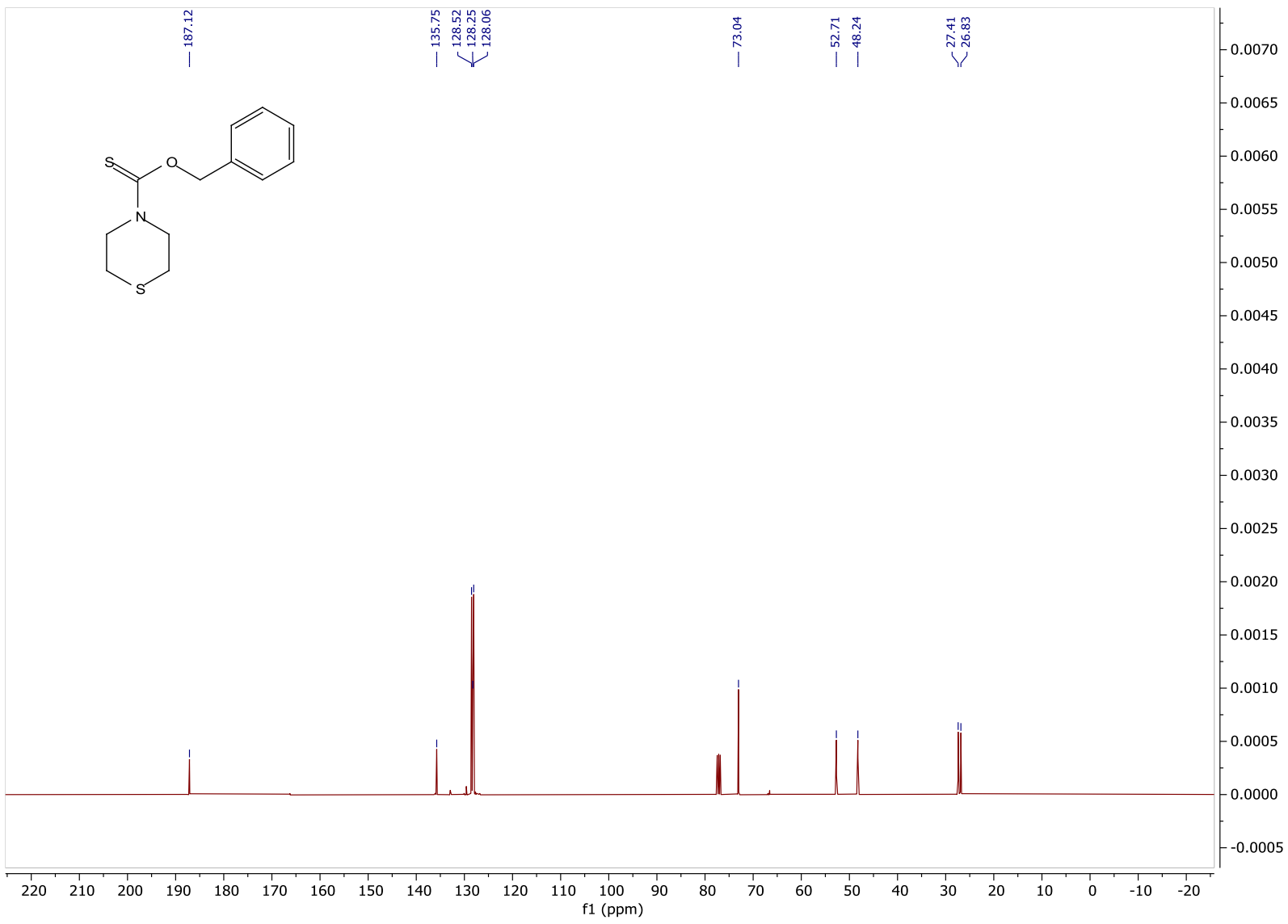

$^{13}C$ NMR (101 MHz, $CDCl_3$) of **7a**.

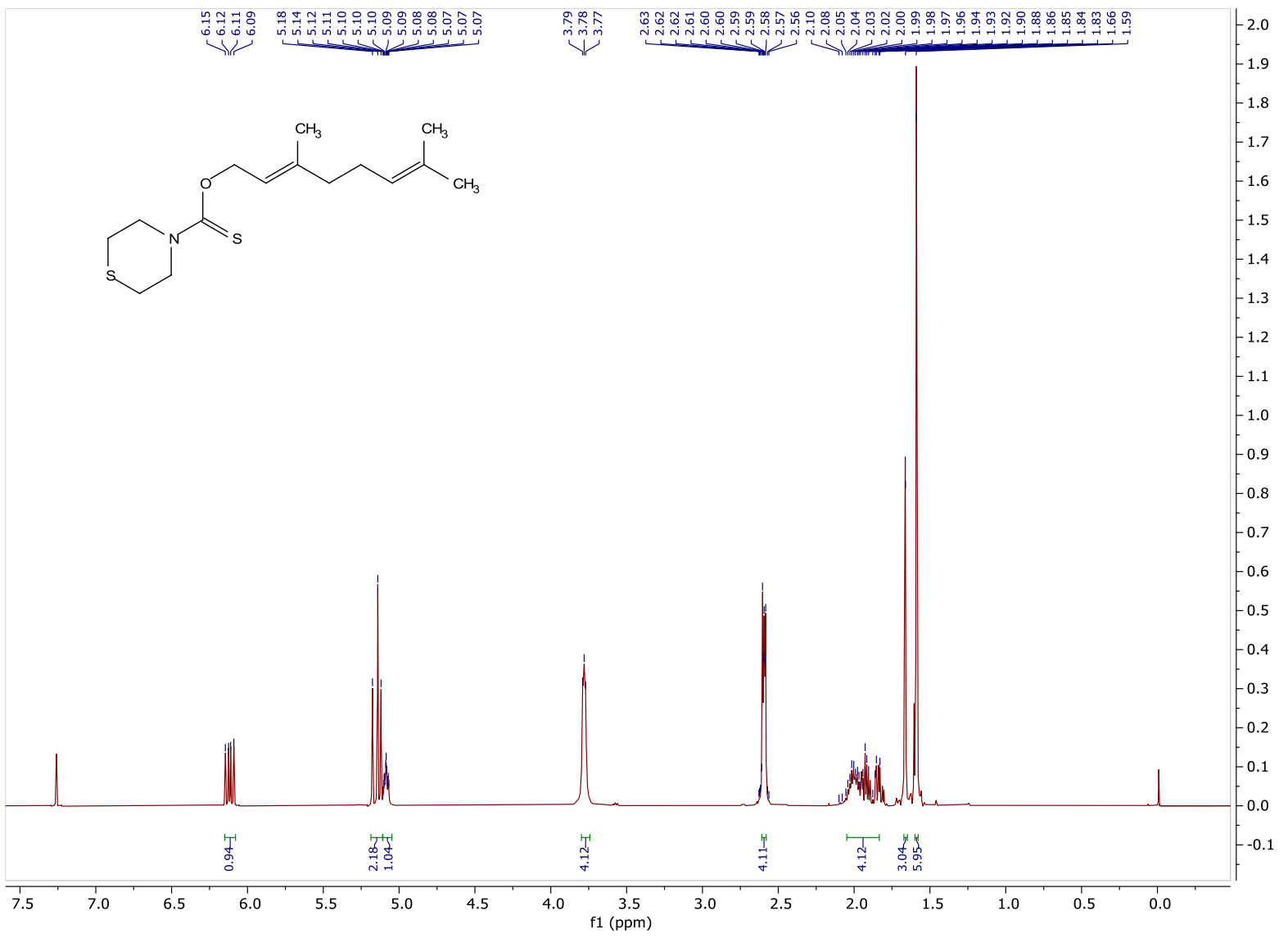

$^{1}H$ NMR (500 MHz, $CDCl_3$) of **7b**.

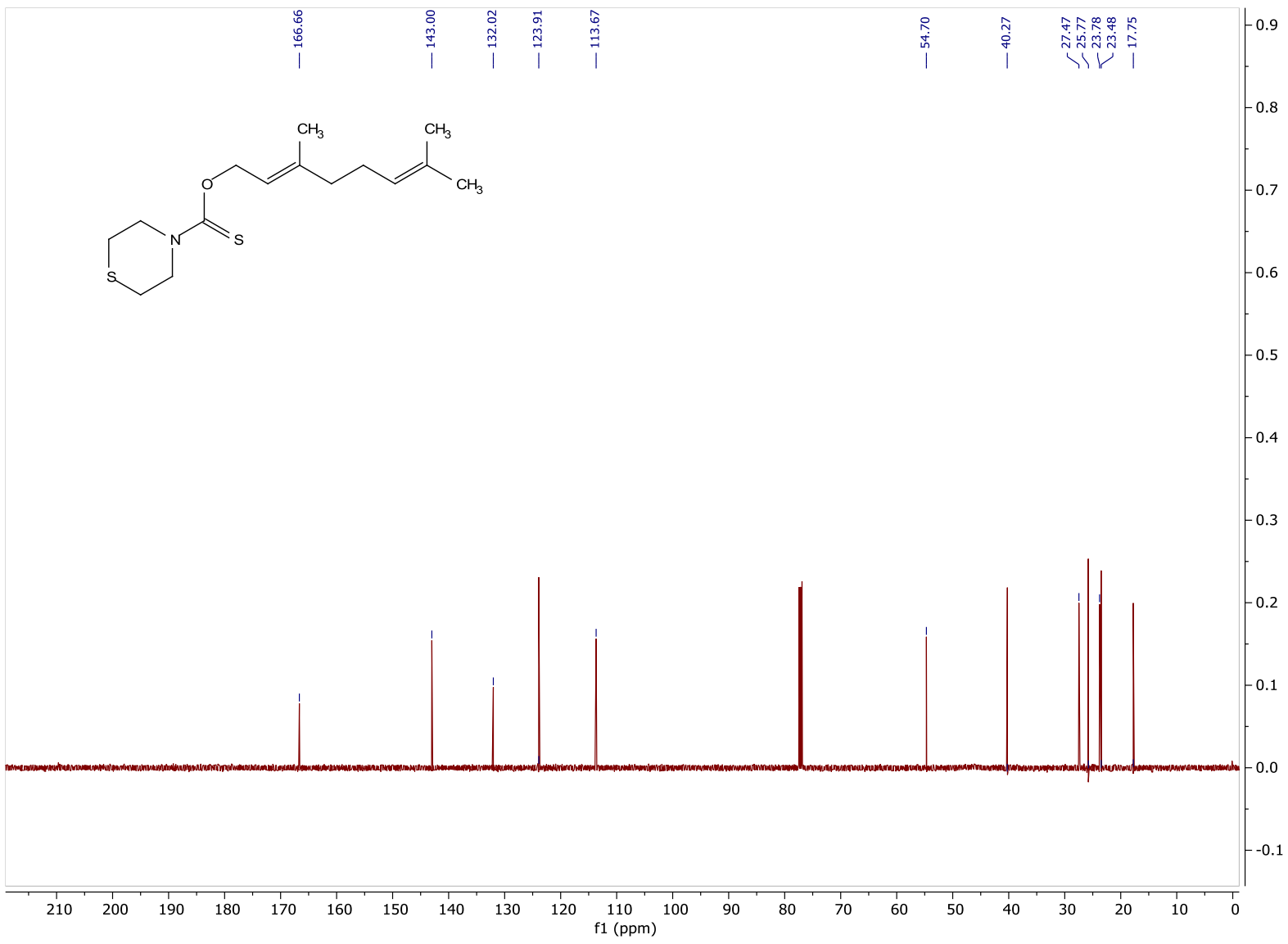


$^{13}$C NMR (126 MHz, $CDCl_3$) of **7b**.

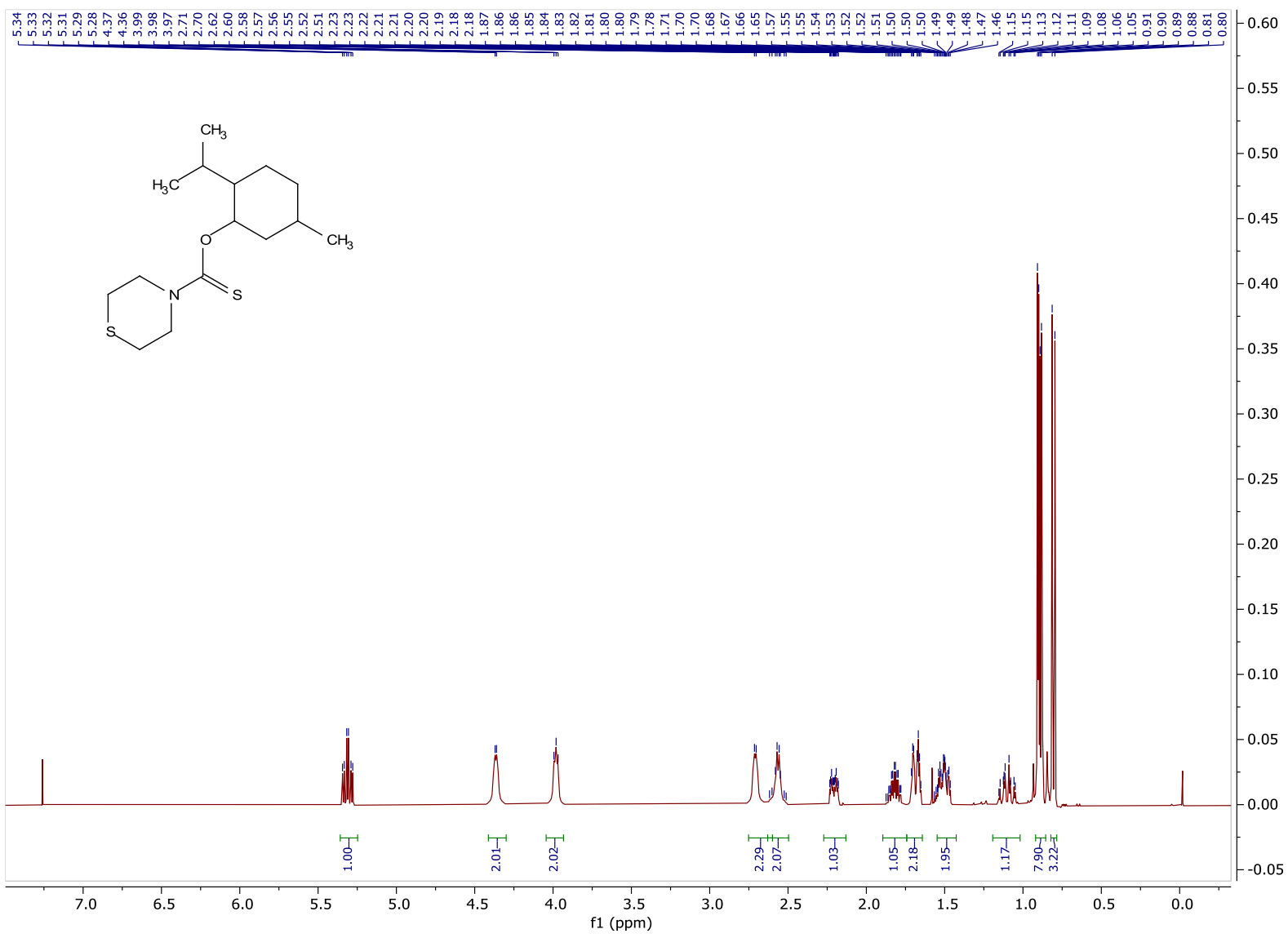


$^{1}$H NMR (400 MHz, $CDCl_3$) of **7c**.

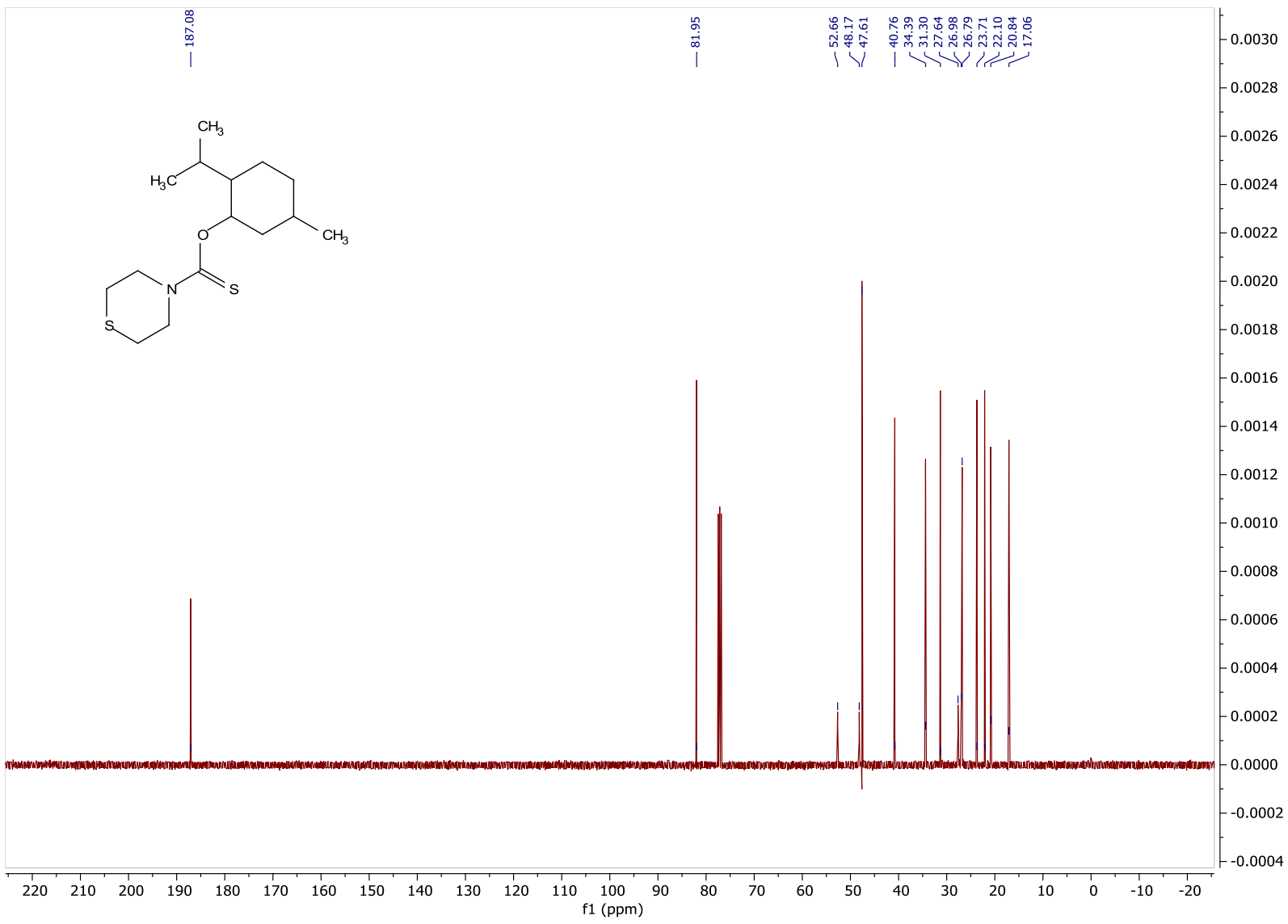

$^{13}C$ NMR (101 MHz, $CDCl_3$) of **7c**.

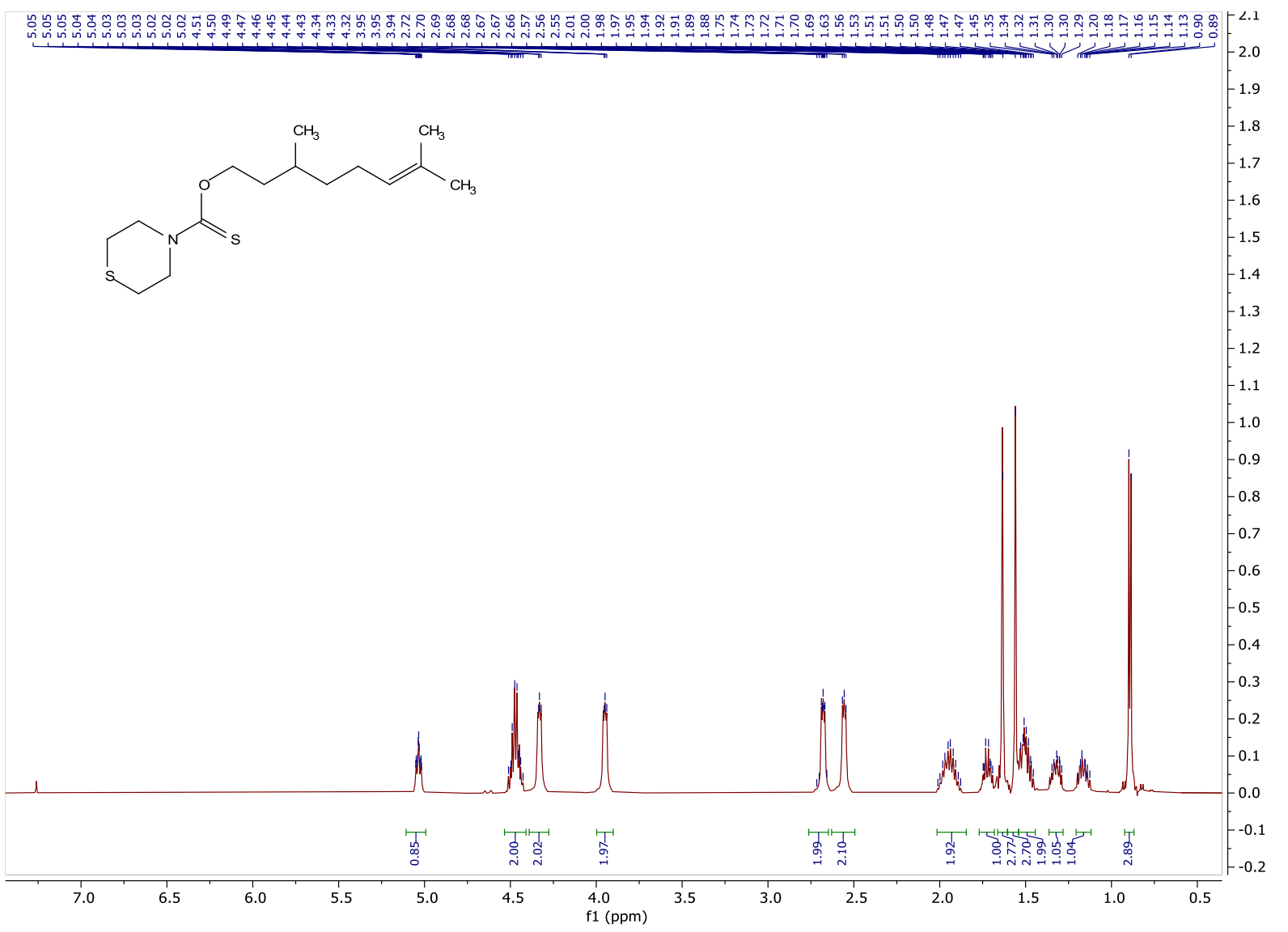

$^{1}H$ NMR (500 MHz, $CDCl_3$) of **7d**

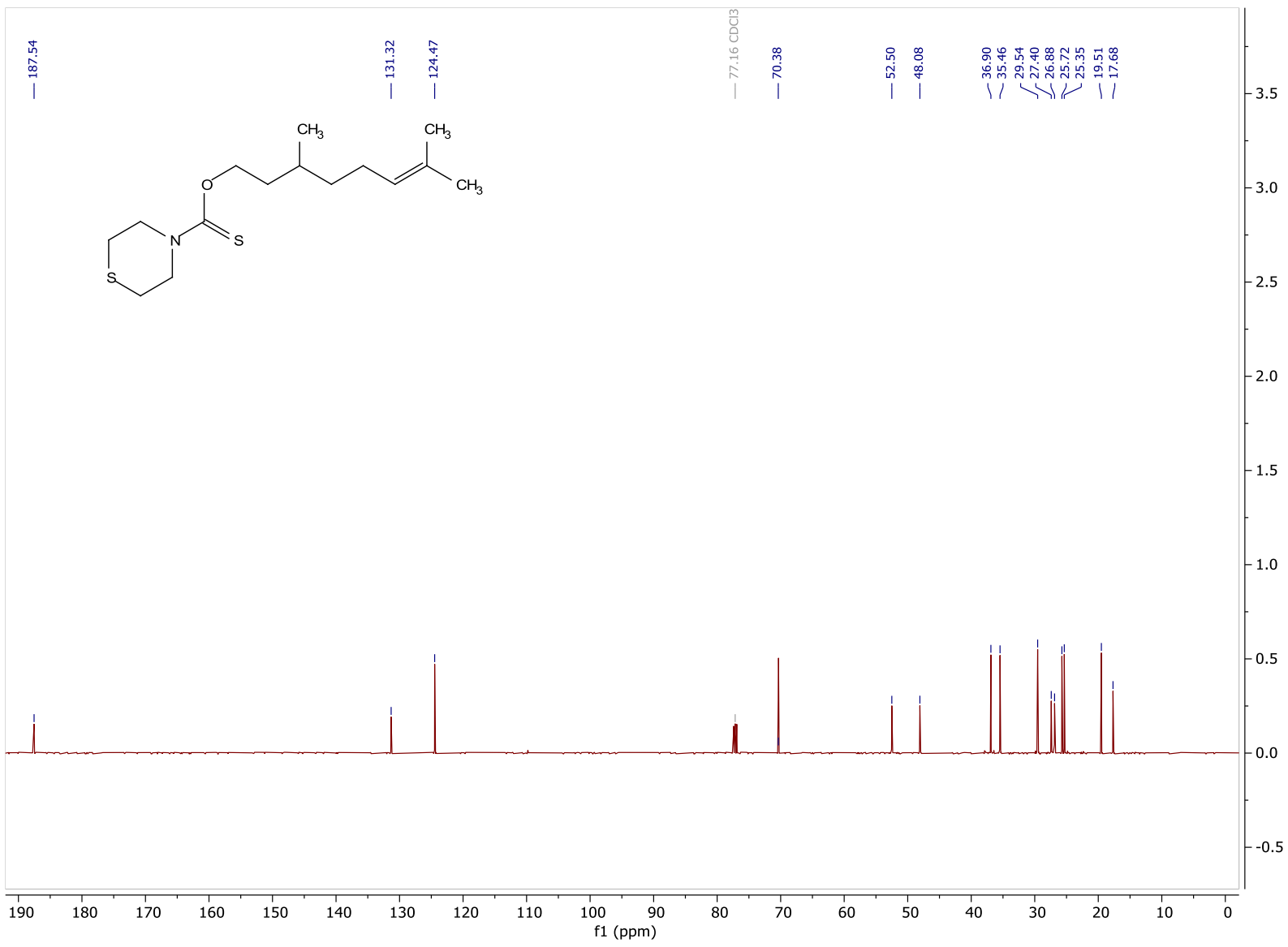

$^{13}C$ NMR (126 MHz, $CDCl_3$) of **7d**

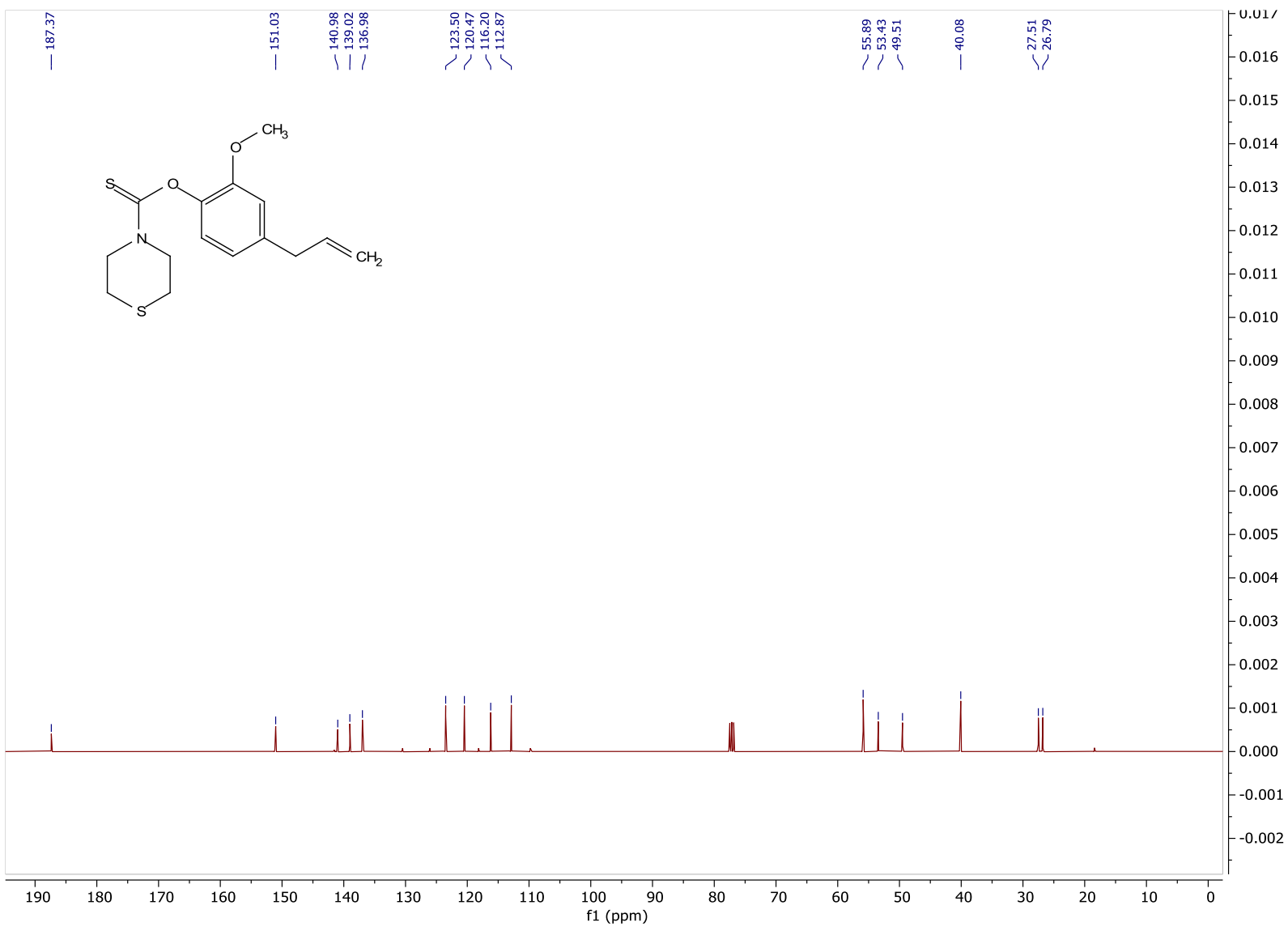

$^{13}C$ NMR (101 MHz, $CDCl_3$) of **7e**

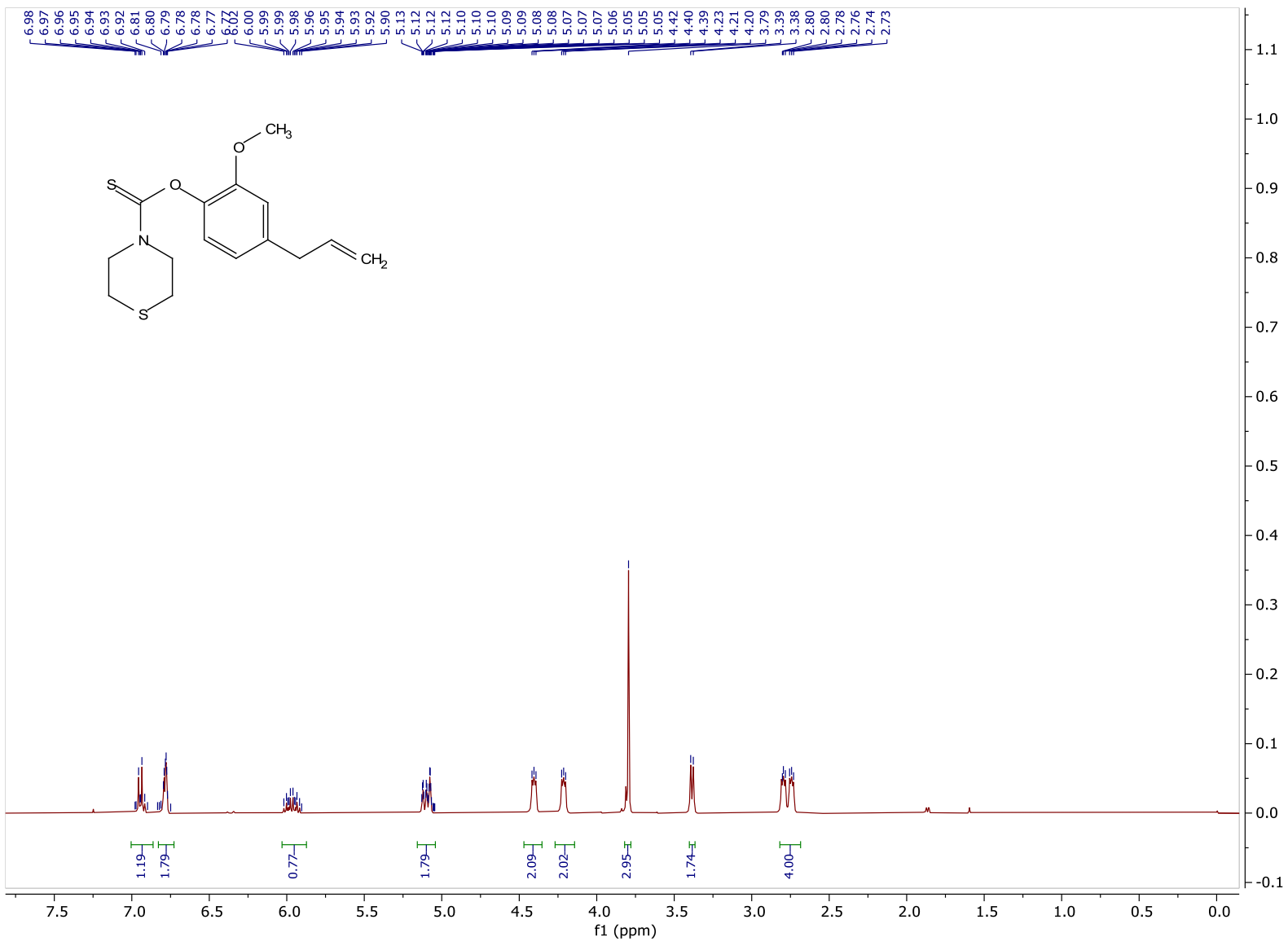

$^{1}$H NMR (400 MHz, $CDCl_3$) of **7e**

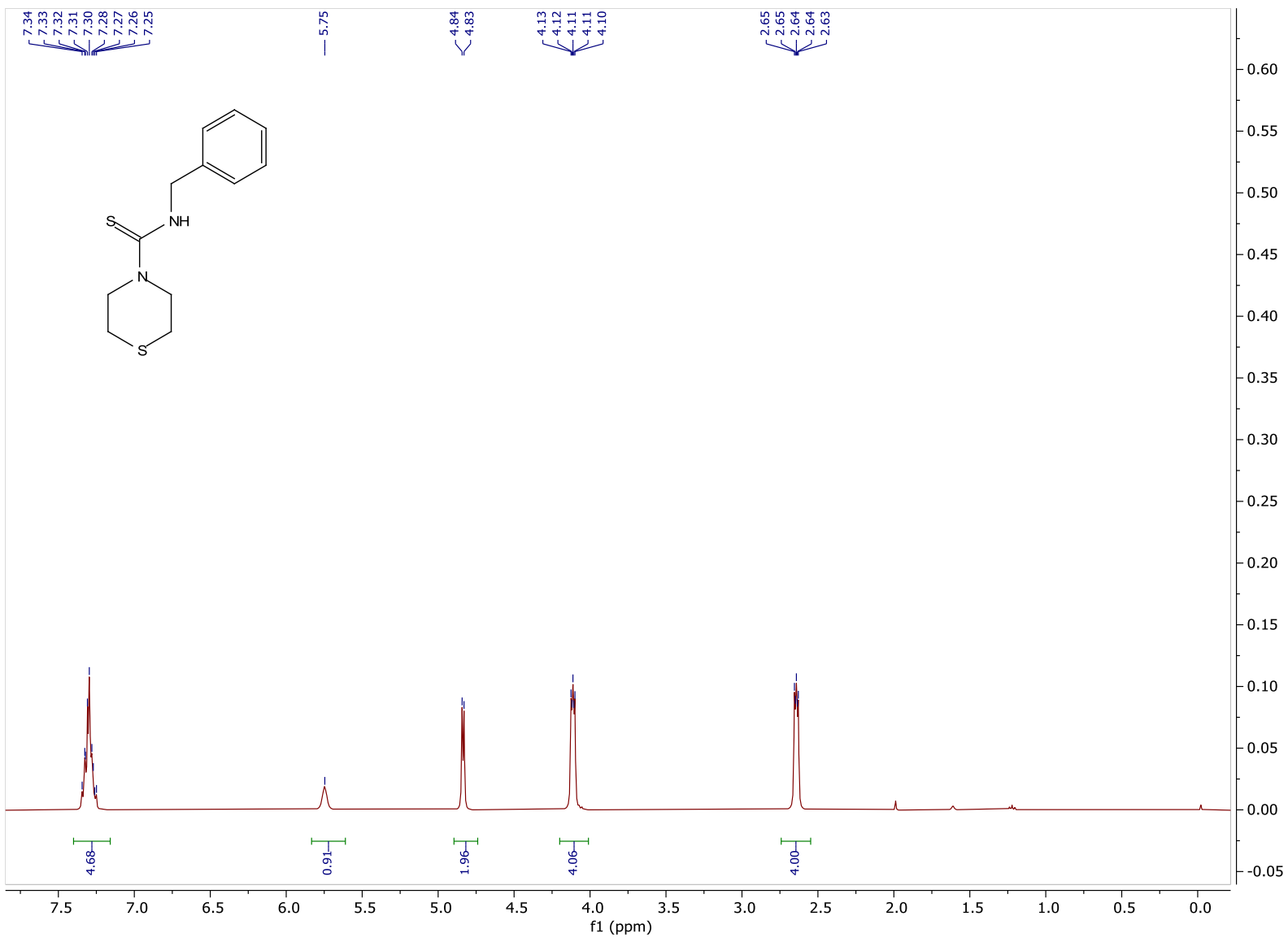

$^{1}$H NMR (400 MHz, $CDCl_3$) of **8a**

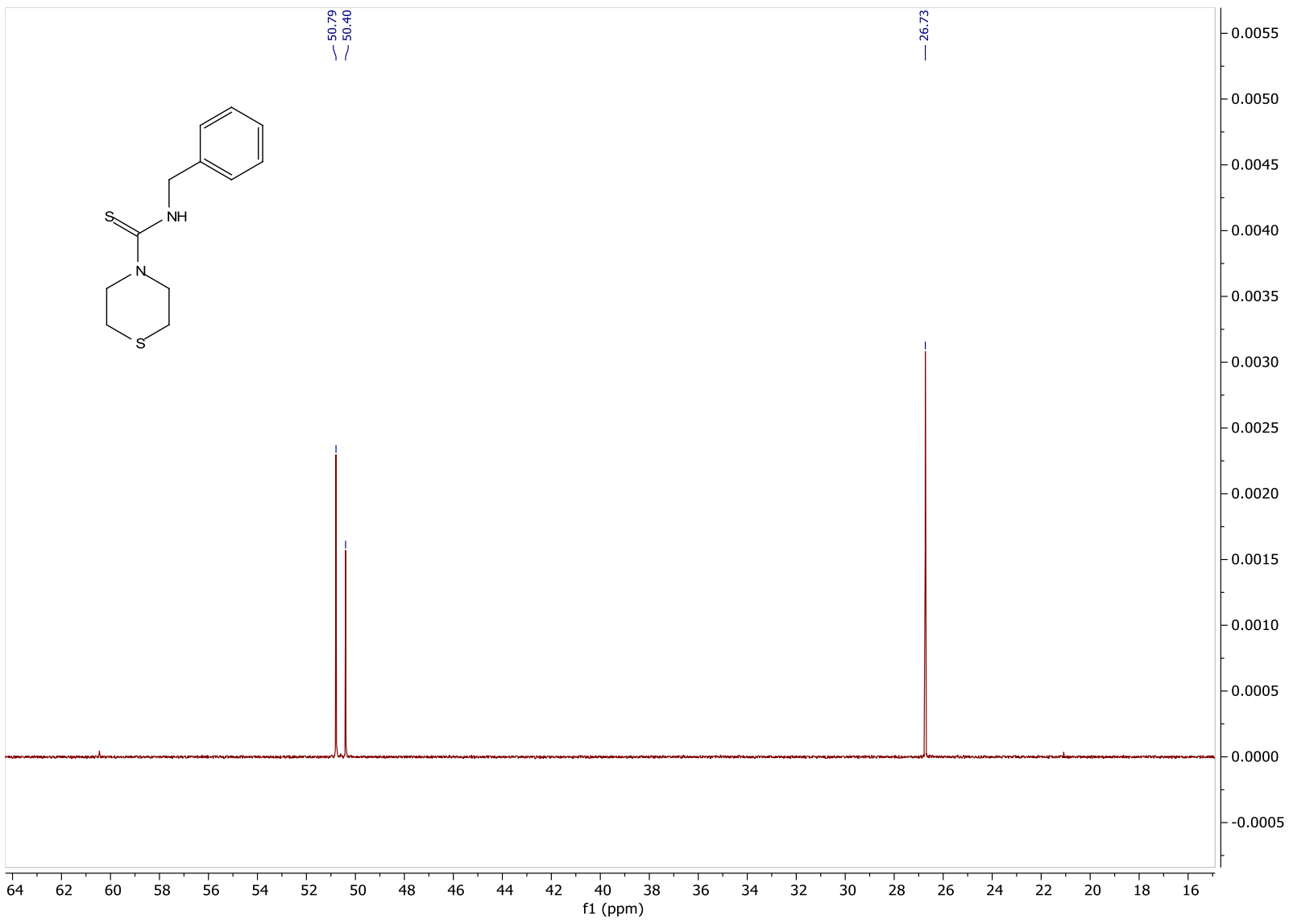

$^{13}$C NMR (101 MHz, $CDCl_3$) of **8a**

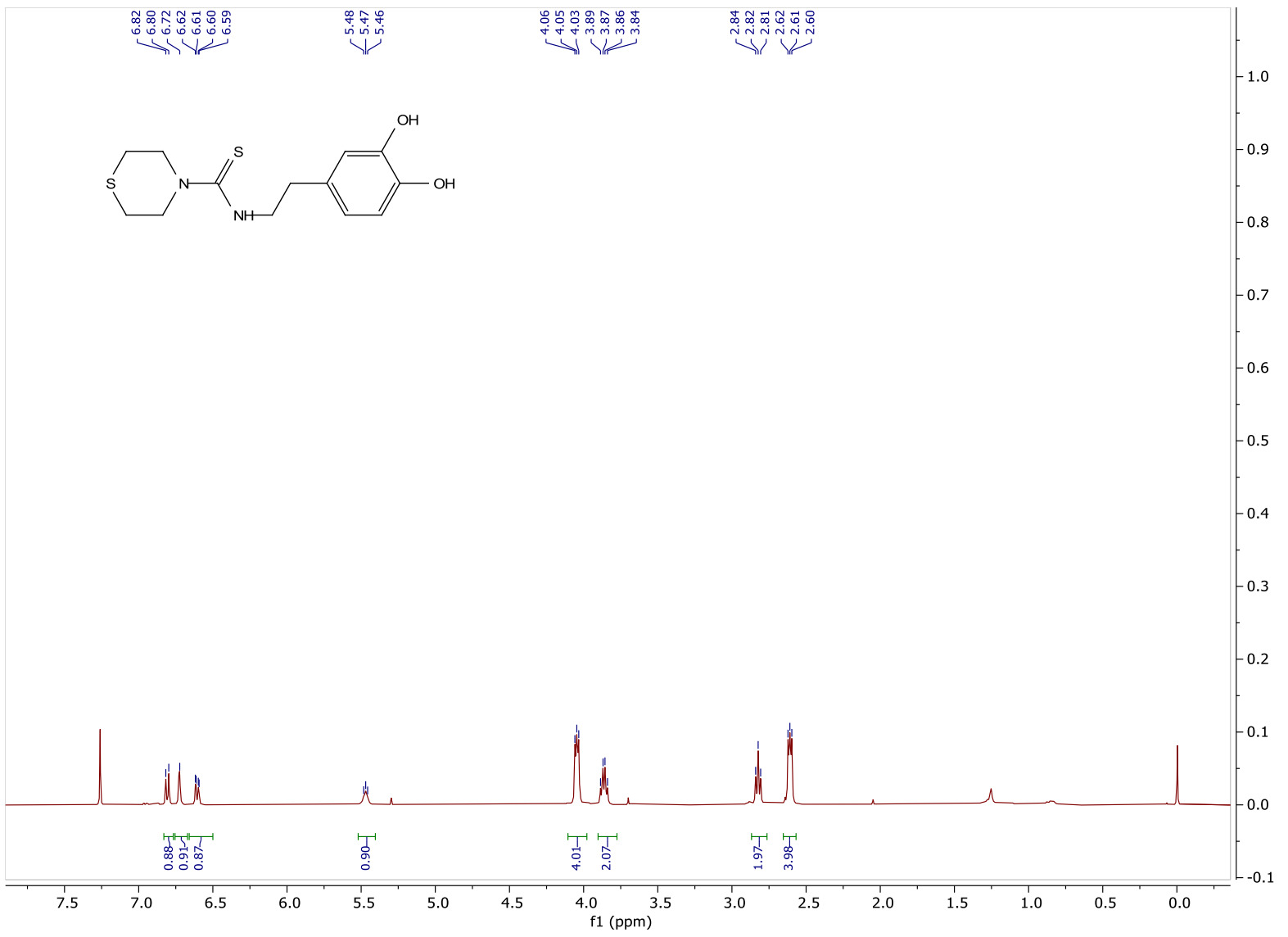

$^{1}$H NMR (400 MHz, $CDCl_3$) of **8b**

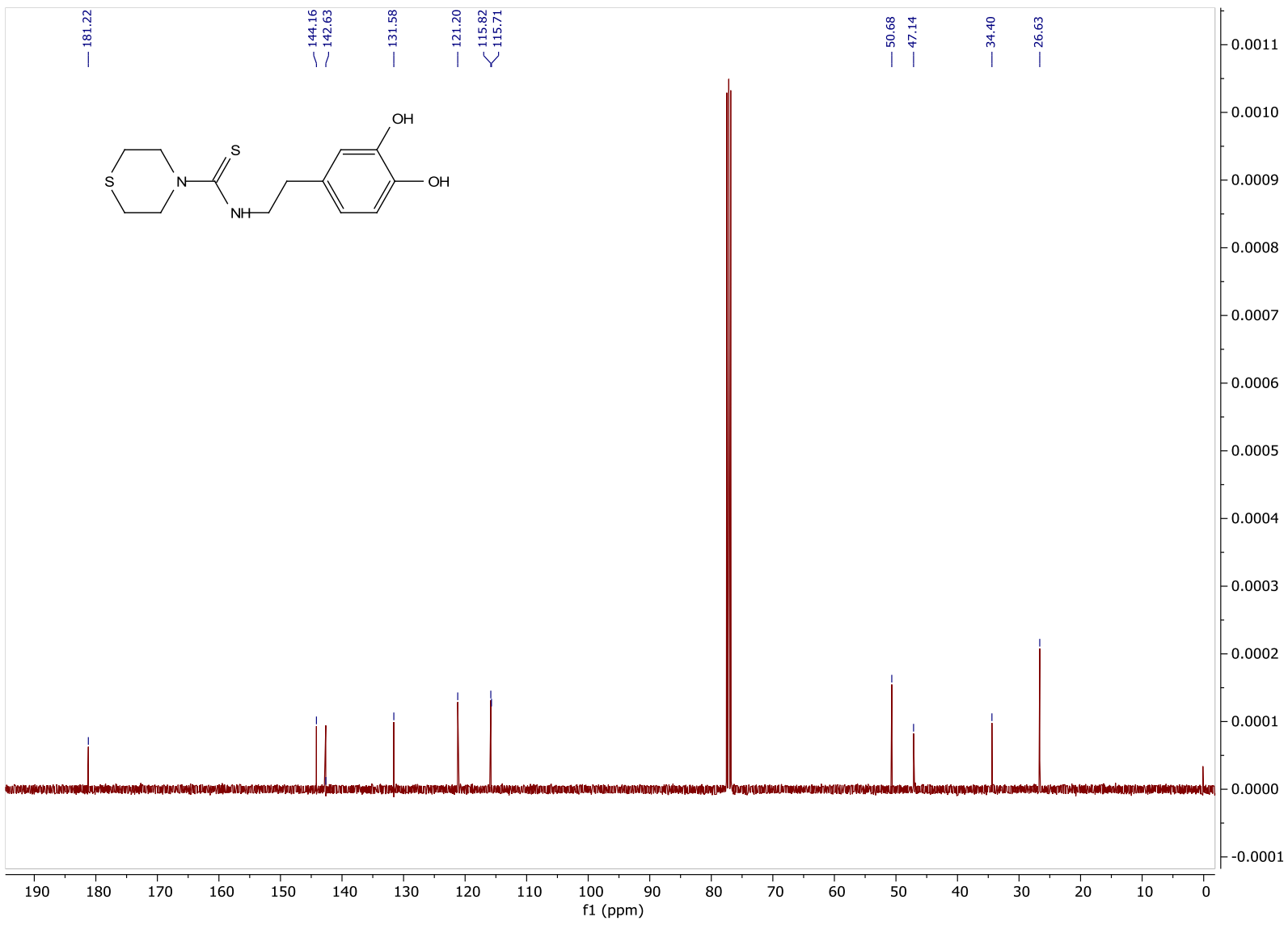

$^{13}C$ NMR (101 MHz, $CDCl_3$) of **8b**

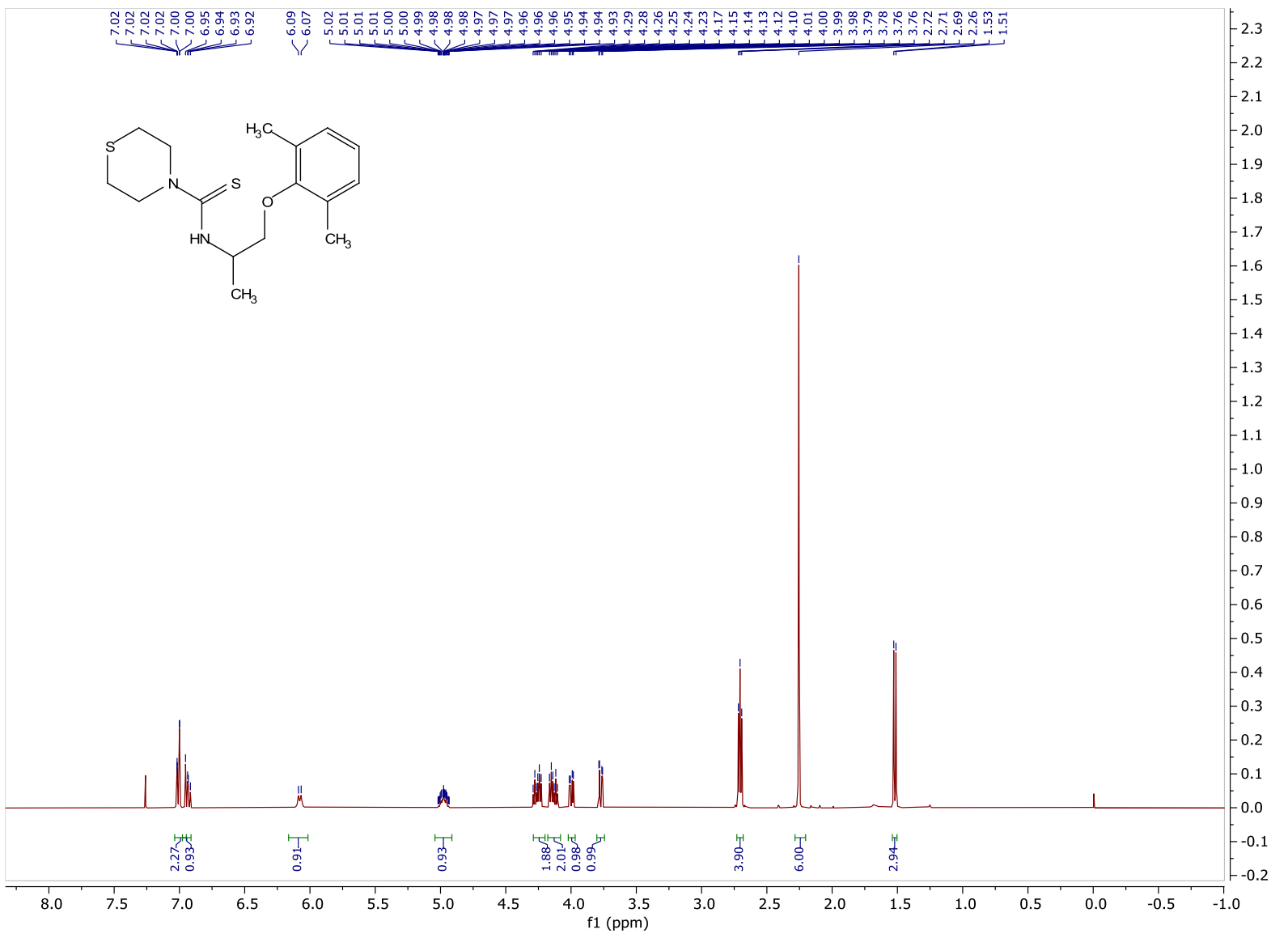

$^{1}H$ NMR (400 MHz, $CDCl_3$) of **8c**

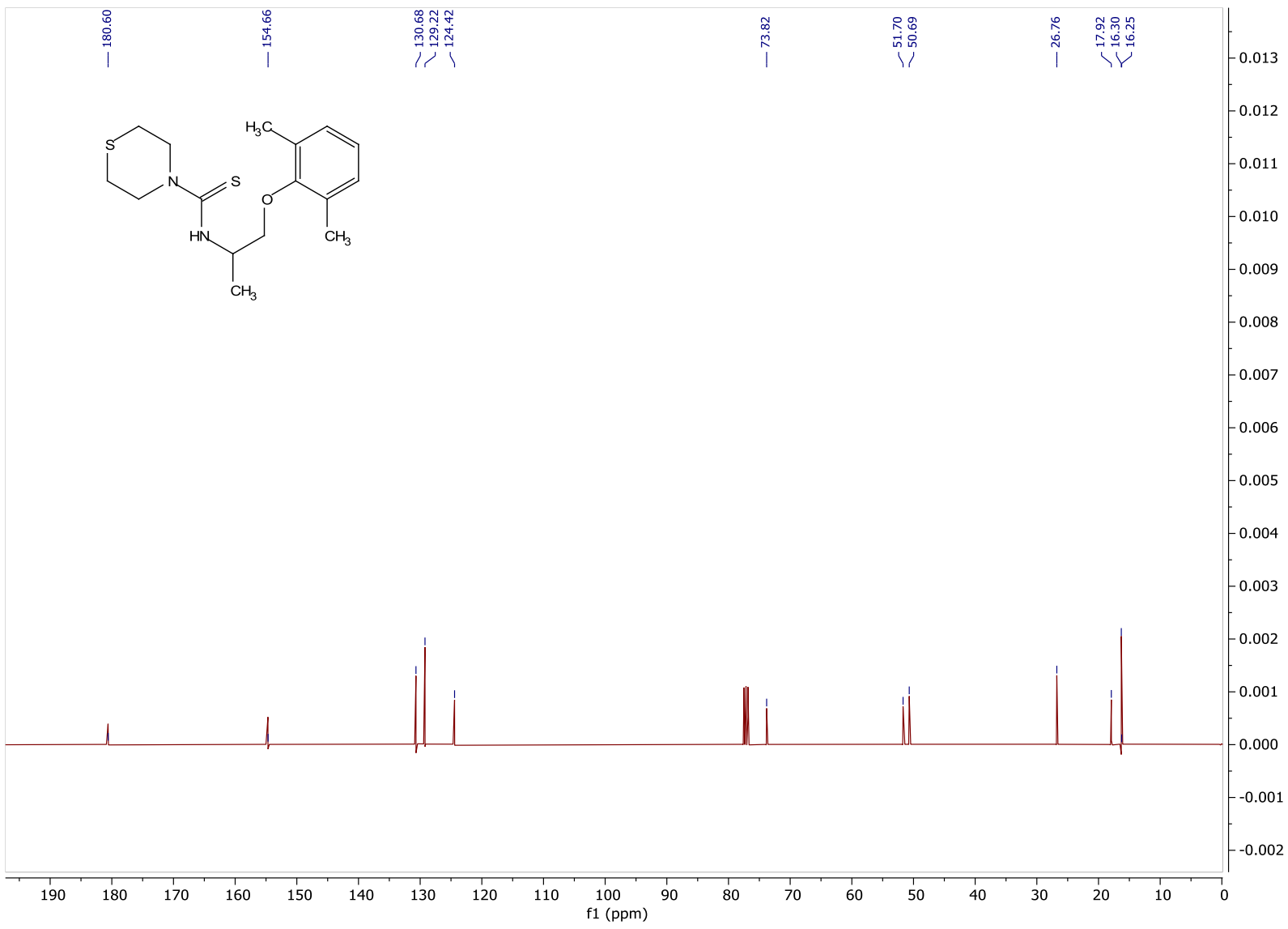

$^{13}$C NMR (101 MHz, $CDCl_3$) of **8c**

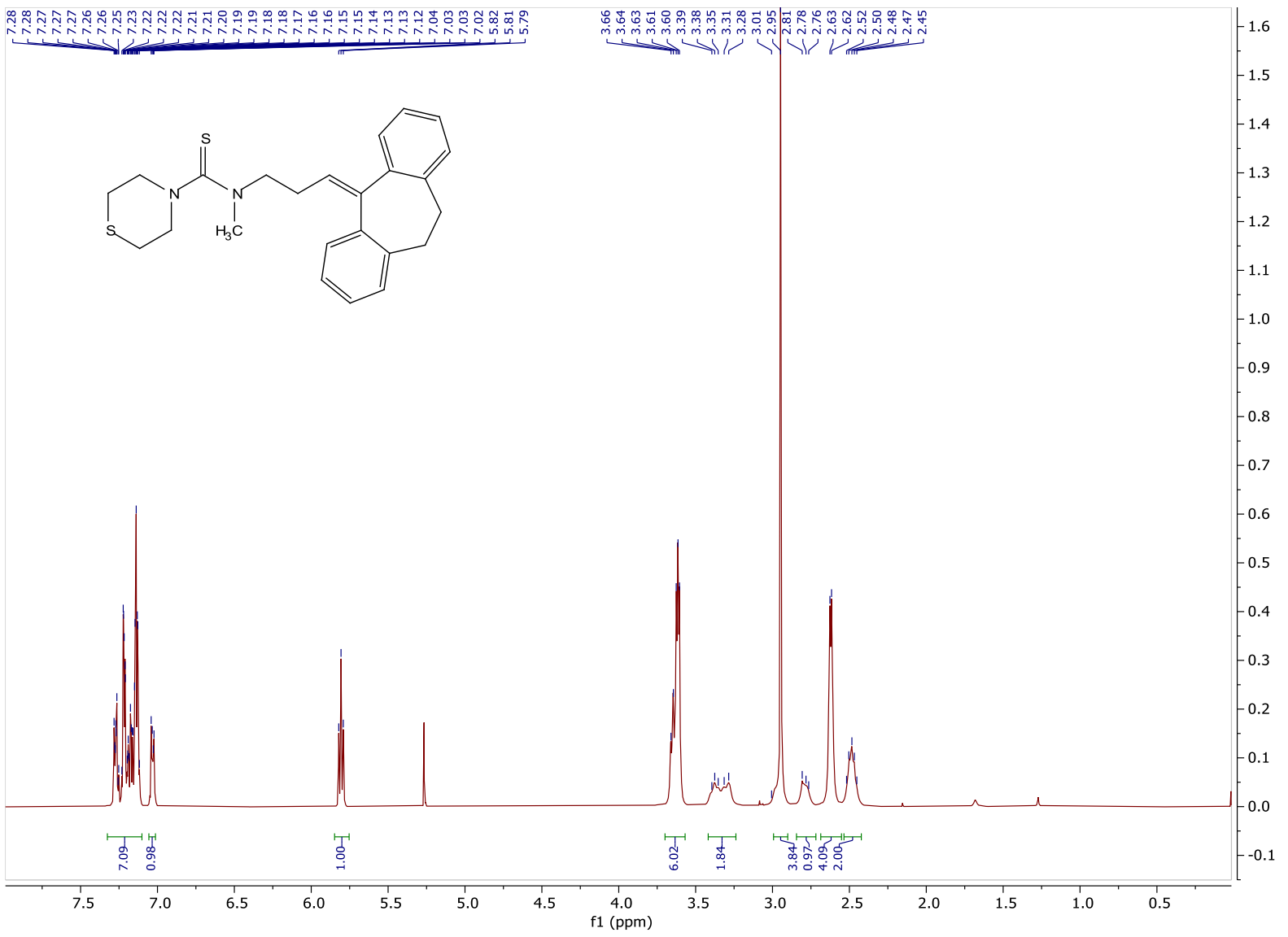

$^{1}$H NMR (500 MHz, $CDCl_3$) of **8d**

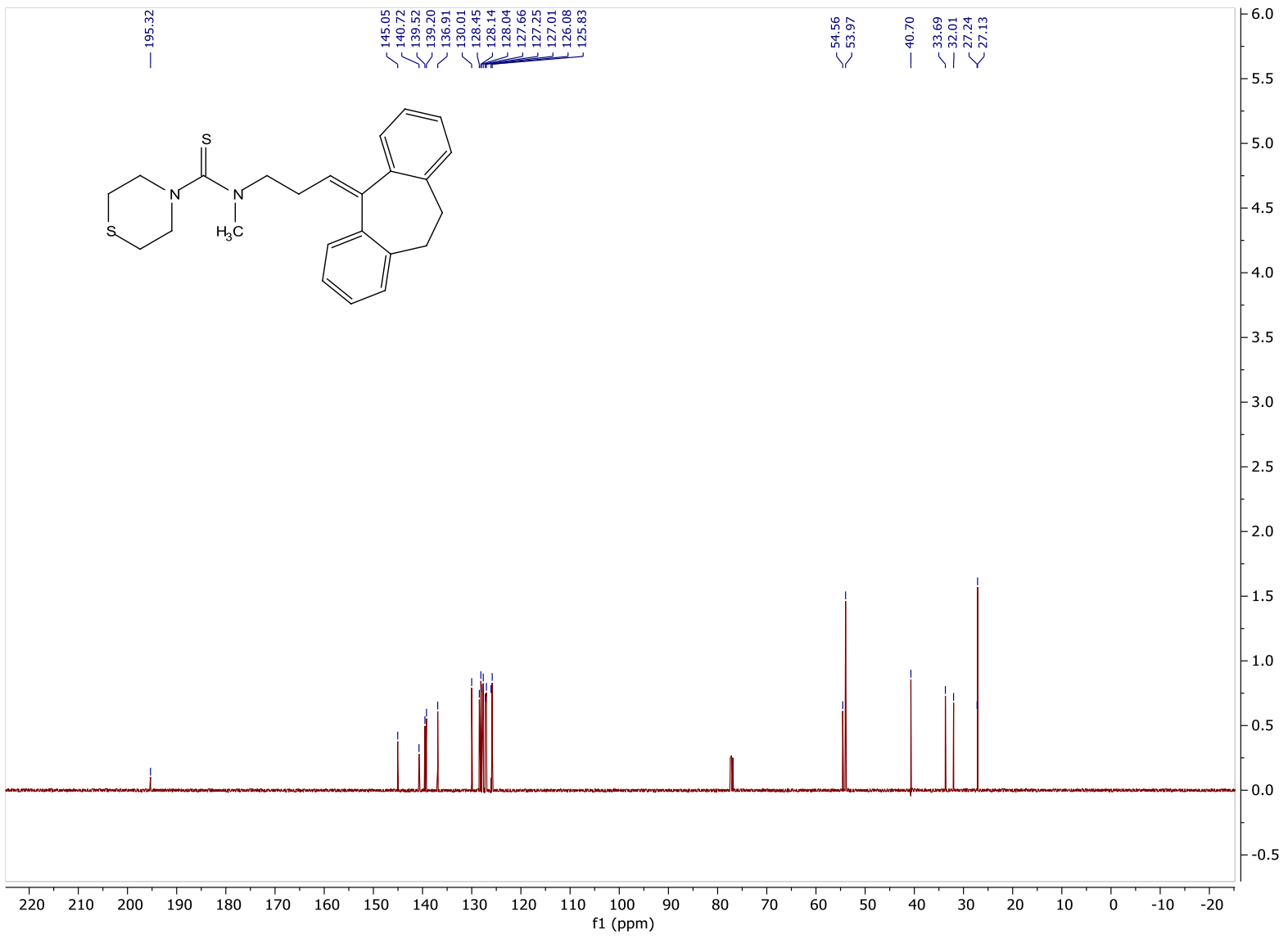

$^{13}C$ NMR (126 MHz, $CDCl_3$) of **8d**

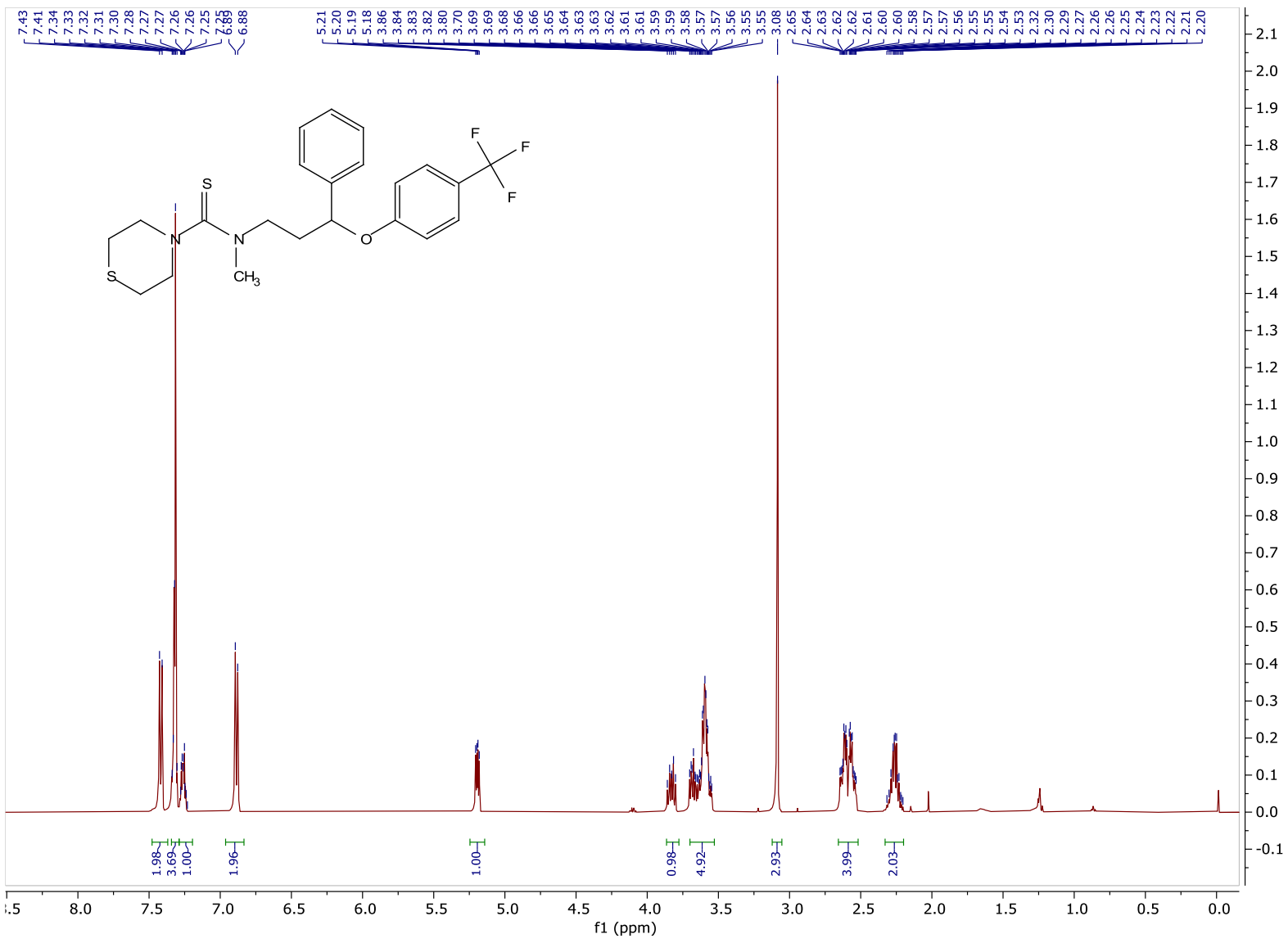

$^{1}H$ NMR (500 MHz, $CDCl_3$) of **8e**

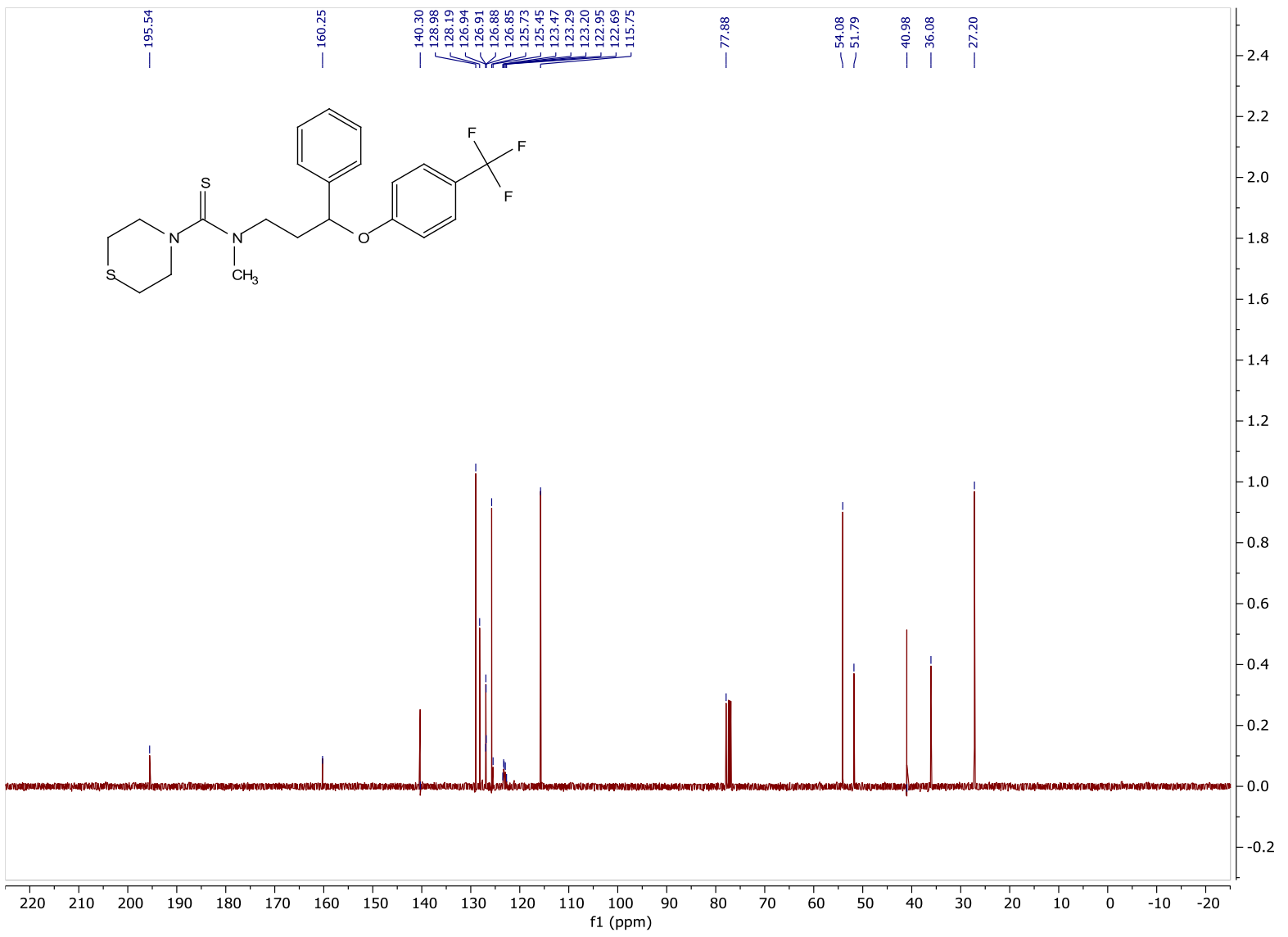

$^{13}C$ NMR (126 MHz, $CDCl_3$) of **8e**

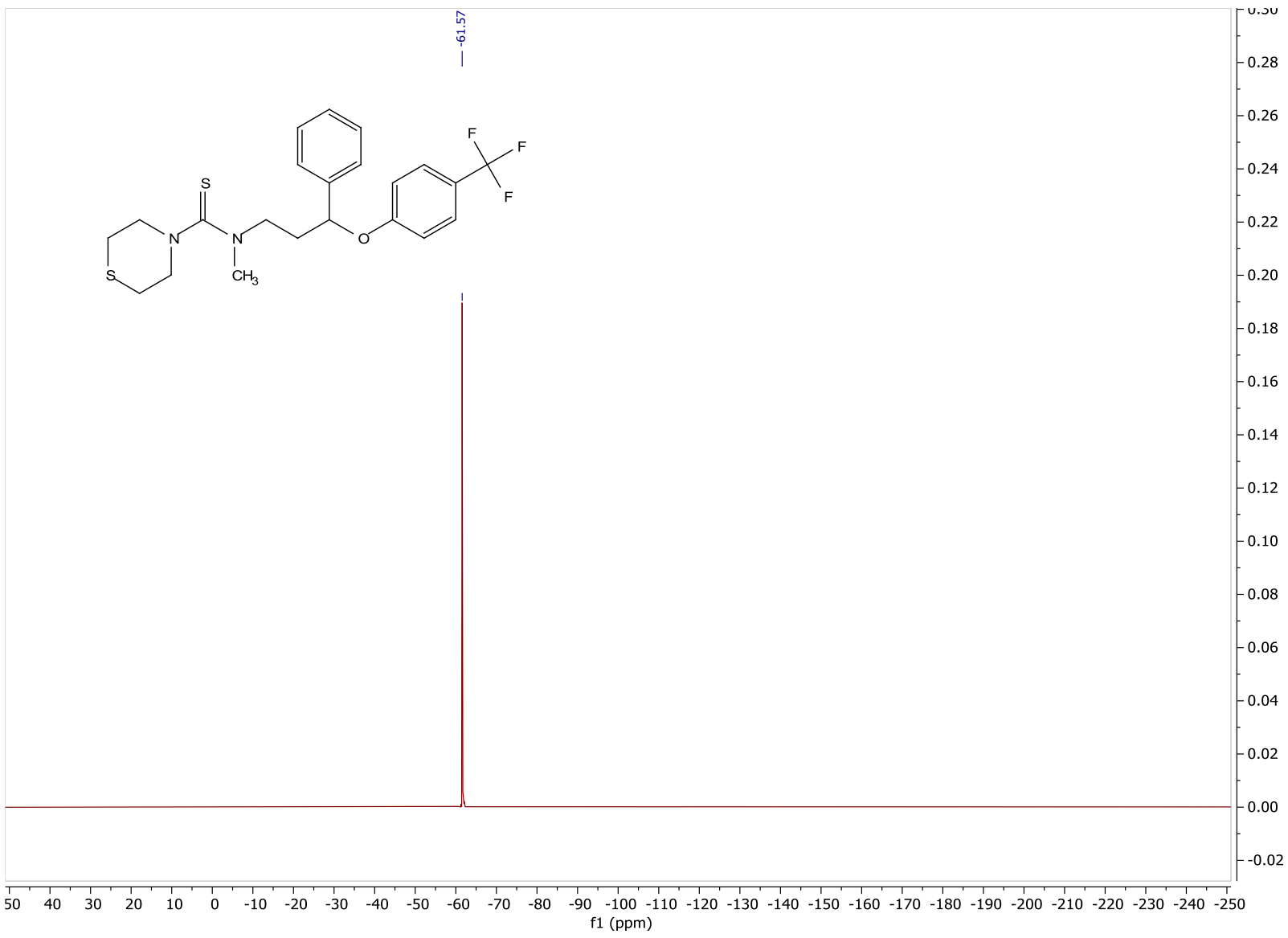

$^{19}F$ NMR (376 MHz, $CDCl_3$) of **8e**

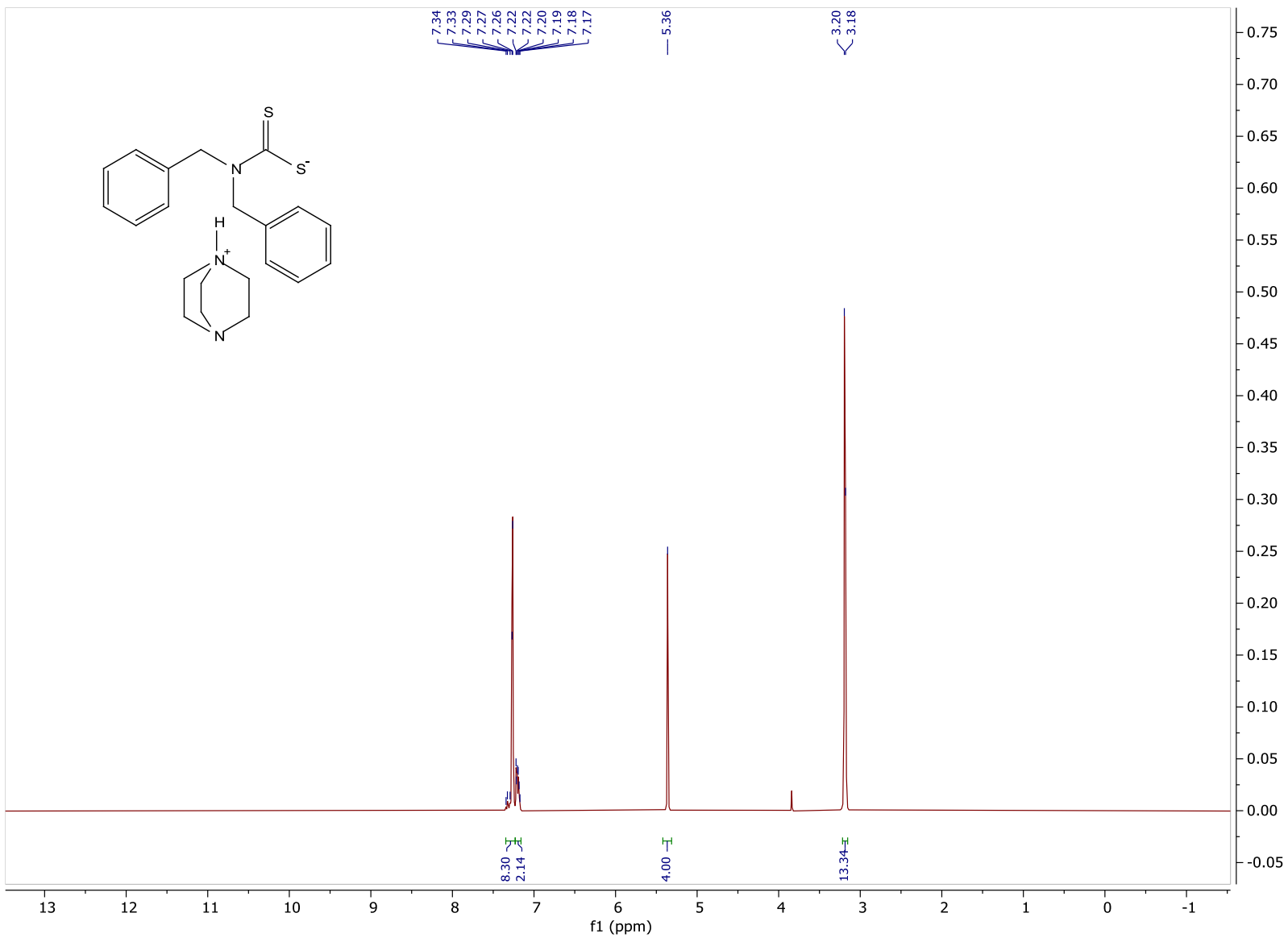

$^{1}$H NMR (400 MHz, CDCl$_3$) of **M1**

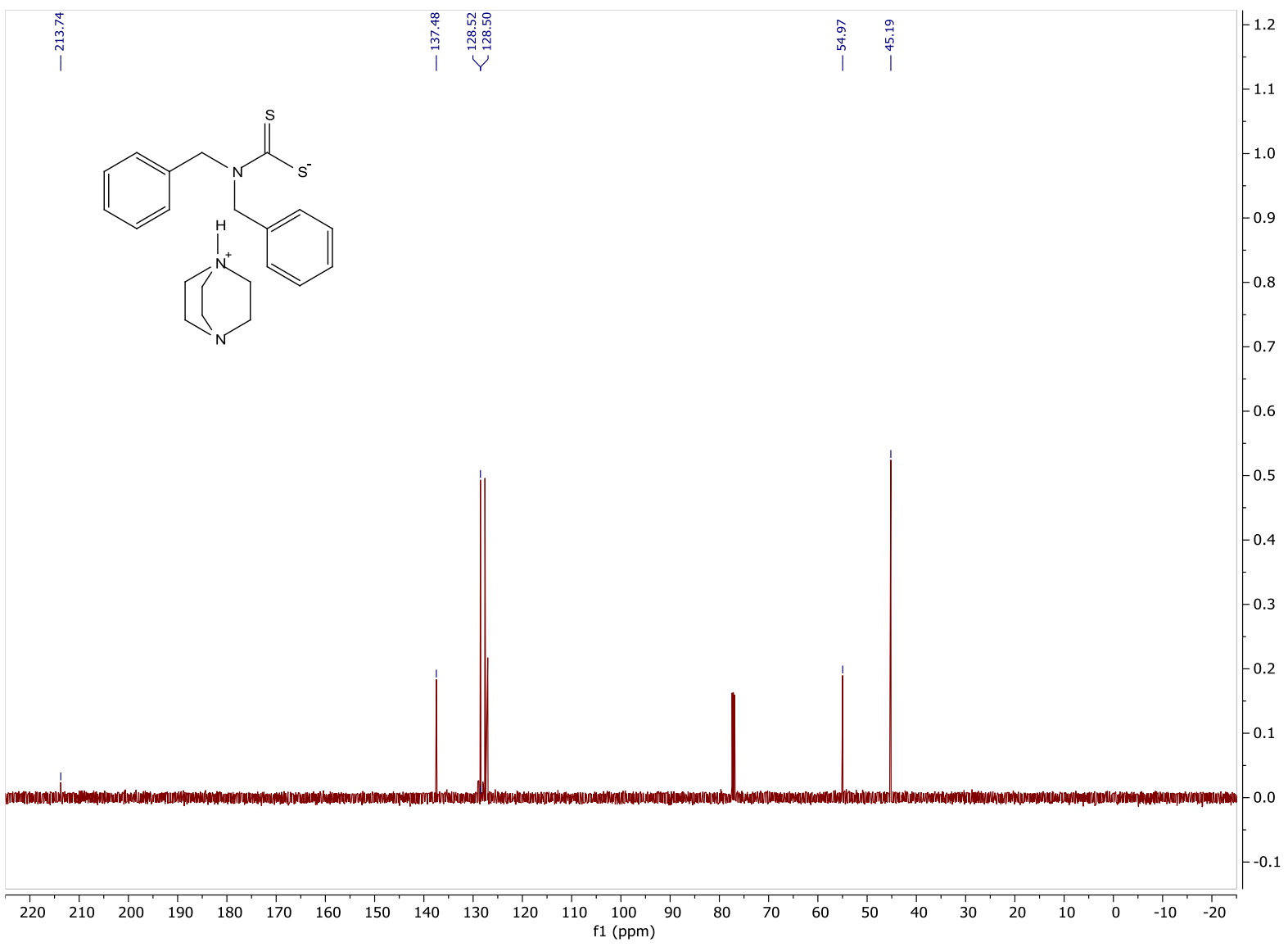

$^{13}$C NMR (126 MHz, CDCl$_3$) of **M1**

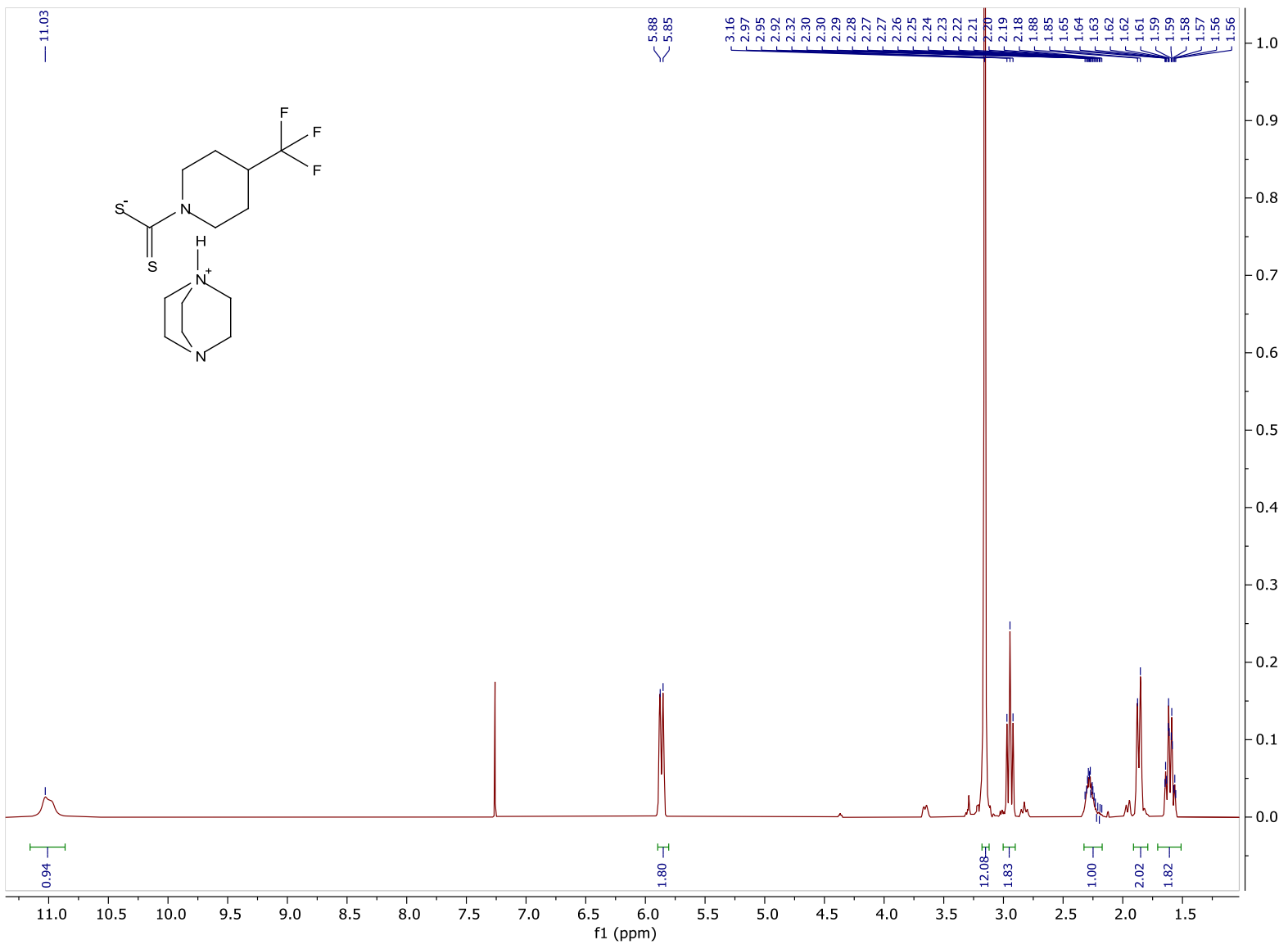

$^{1}$H NMR (500 MHz, CDCl$_3$) of **1m'**

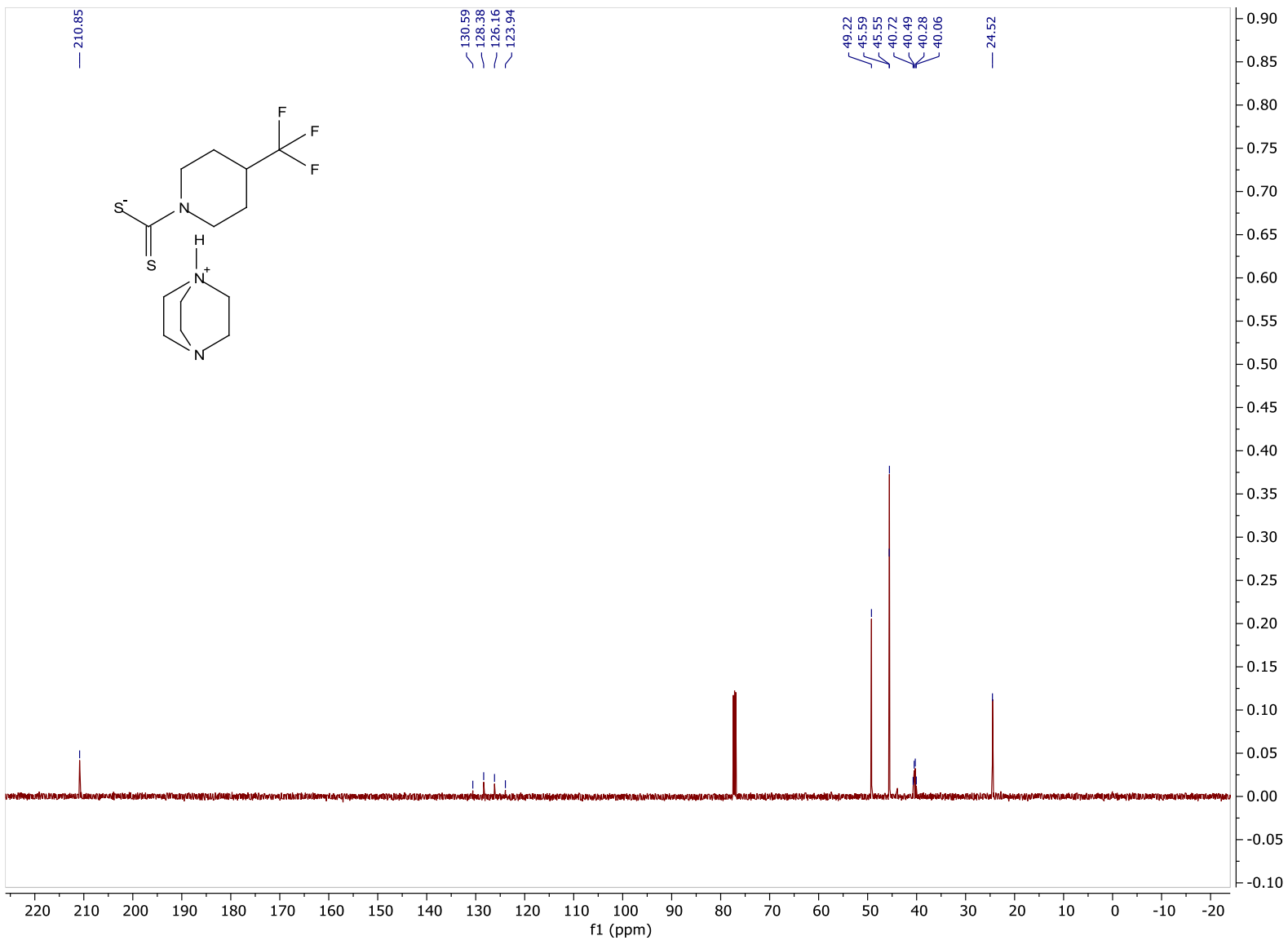

$^{13}$C NMR (126 MHz, CDCl$_3$) of 1m'

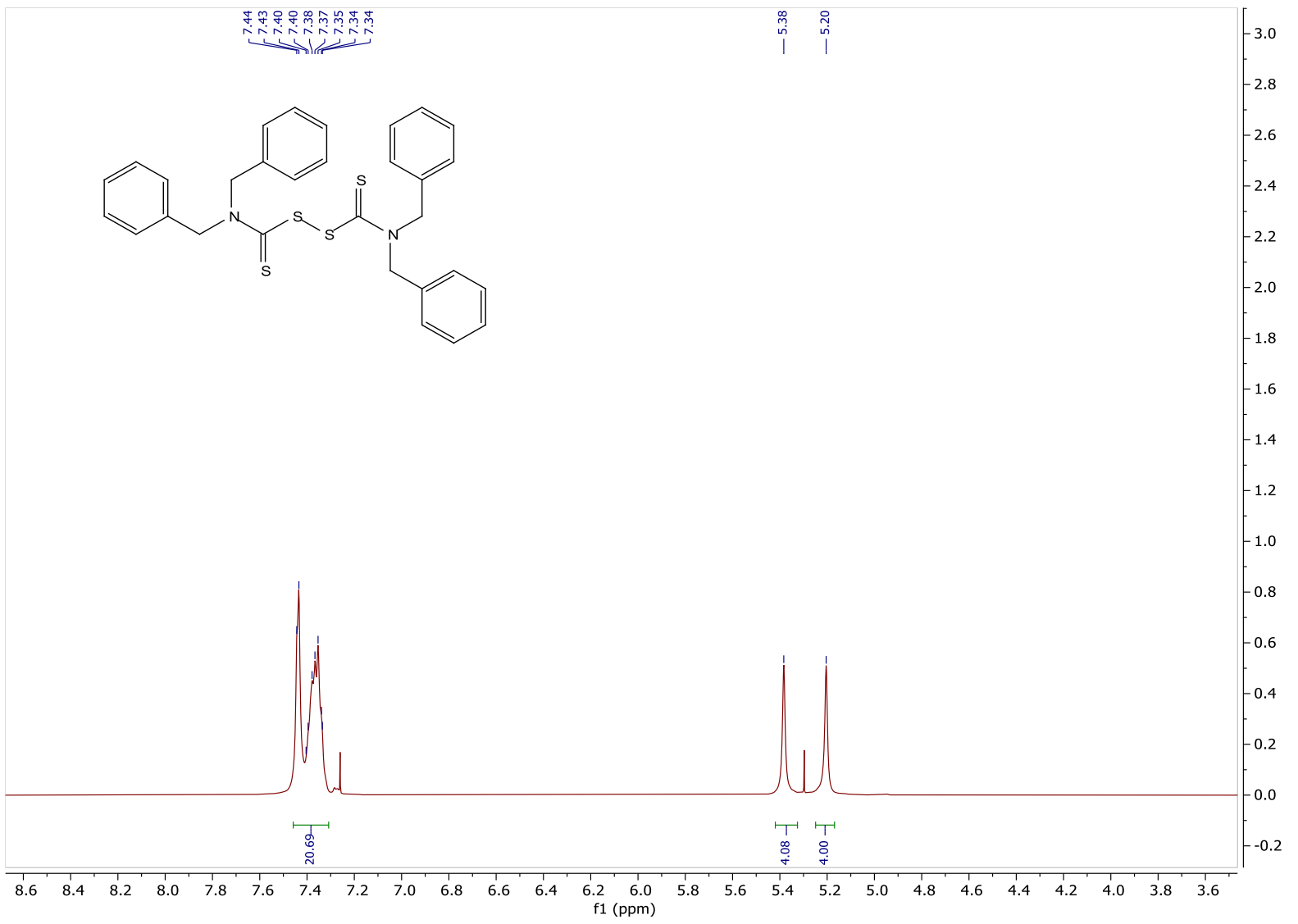

$^{1}H$ NMR (500 MHz, $CDCl_3$) of **M2**

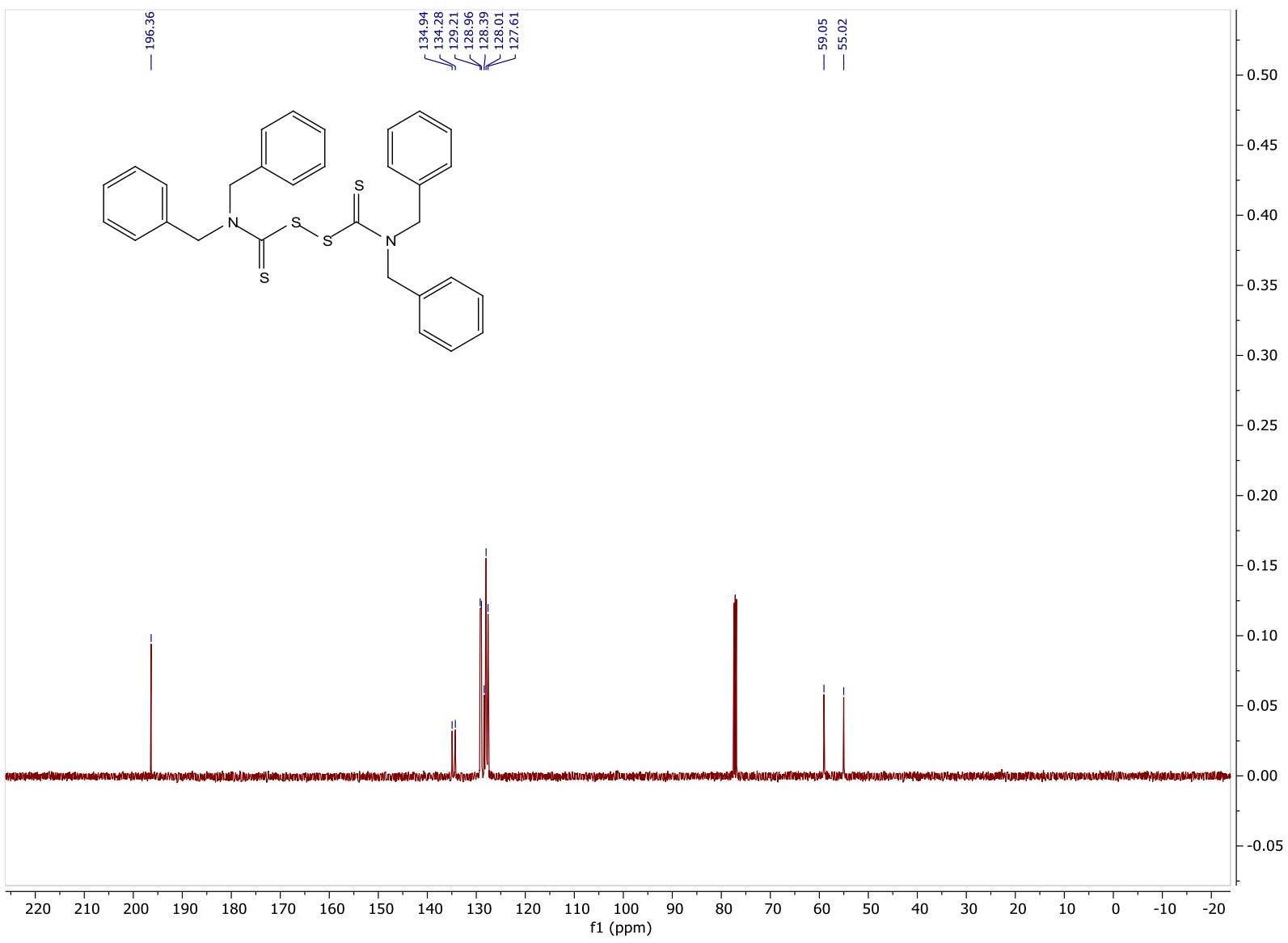

$^{13}C$ NMR (126 MHz, $CDCl_3$) of **M2**

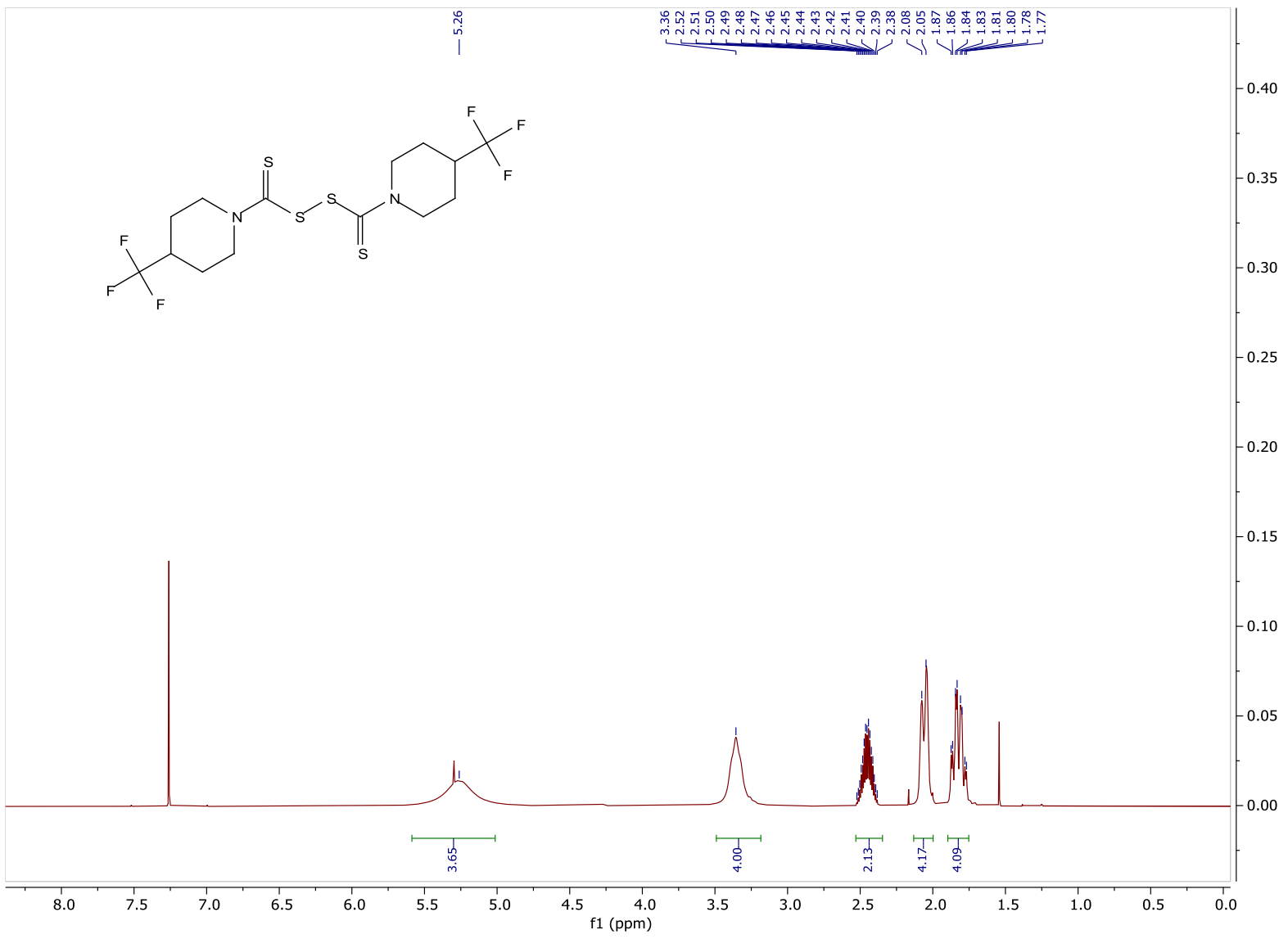


$^{1}$H NMR (400 MHz, $CDCl_3$) of **1m''**

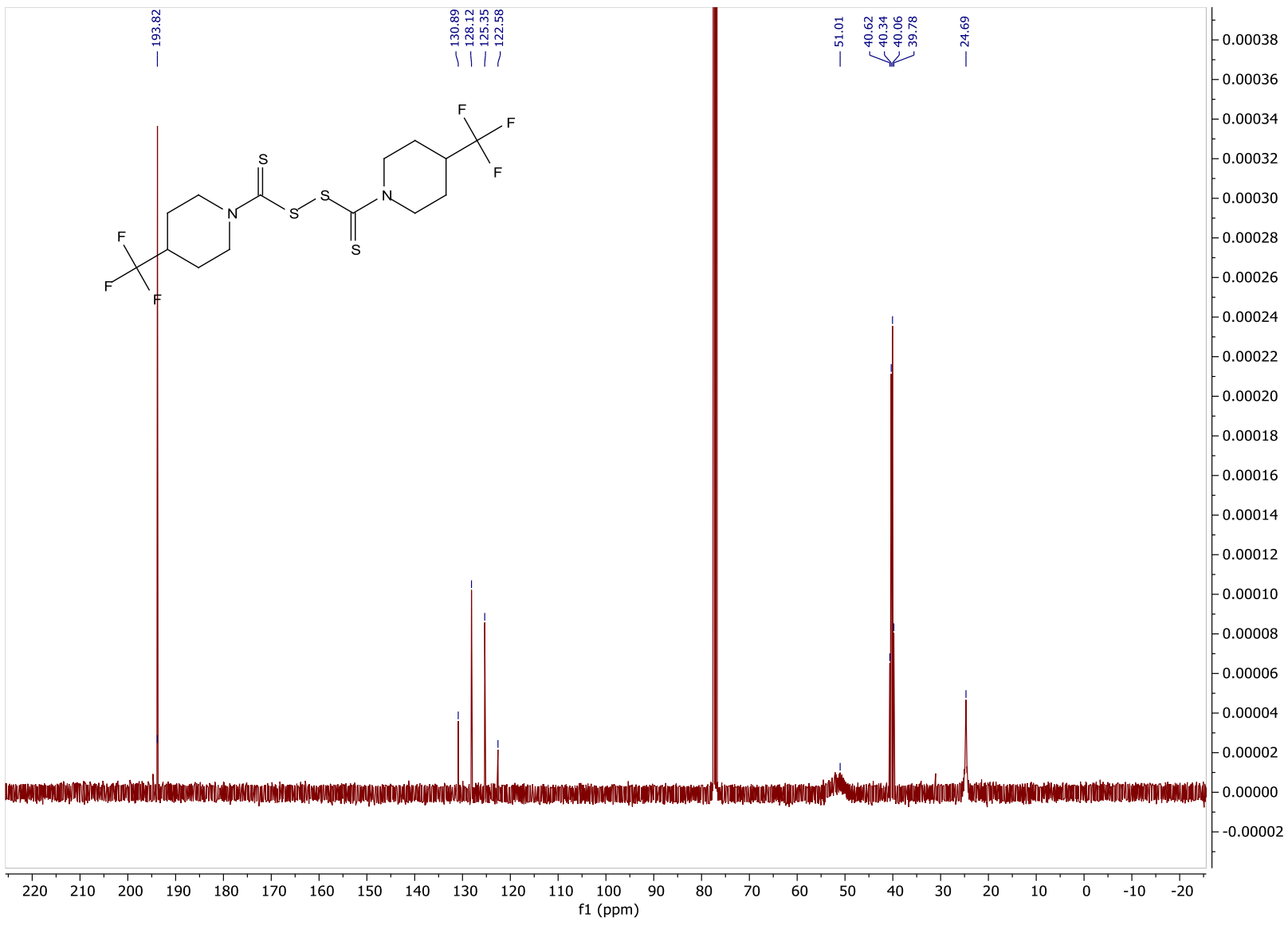


$^{13}$C NMR (101 MHz, $CDCl_3$) of 1**m''**

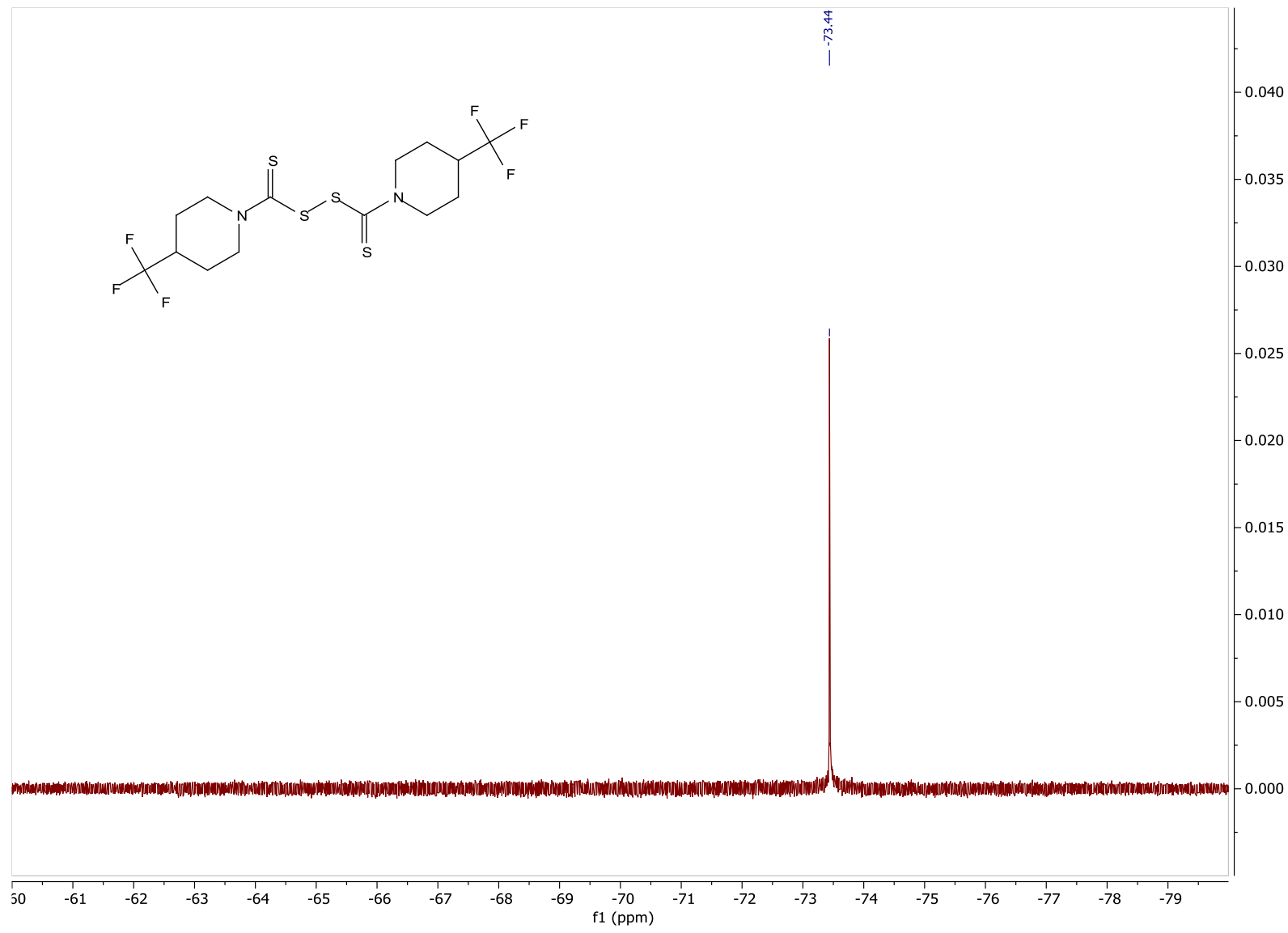


$^{19}$F NMR (376 MHz, $CDCl_3$) of 1**m**''

## Supplementary References


1. Wang, Y. and Gu, M. The concept of spectral accuracy for MS. *Anal. Chem.* **82**, 7055–7062 (2010).
2. Dolomanov, O. V., Bourhis, L. J., Gildea, R. J., Howard, J. A. K. and Puschmann, H. OLEX2: a complete structure solution, refinement and analysis program. *J. Appl. Crystallogr.* **42**, 339–341 (2009).
3. Sheldrick, G. M. A short history of SHELX. *Acta Crystallogr A* **64**, 112–122 (2008).
4. Frisch, M. J., *Journal*, 2016.
5. Whaley, R., Petitet, A. and Dongarra, J. Automated empirical optimizations of software and the ATLAS project. *Parallel Comput.* **27**, 3–35 (2001).
6. Whaley, R. C. and Petitet, A. Minimizing development and maintenance costs in supporting persistently optimized BLAS. *Softw. Pract. Exp.* **35**, 101–121 (2005).
7. Zhao, Y. and Truhlar, D. G. Design of density functionals that are broadly accurate for thermochemistry, thermochemical kinetics, and nonbonded interactions. *J Phys Chem A* **109**, 5656–5667 (2005).
8. Goerigk, L., Hansen, A., Bauer, C., Ehrlich, S., Najibi, A. and Grimme, S. A look at the density functional theory zoo with the advanced GMTKN55 database for general main group thermochemistry, kinetics and noncovalent interactions. *Phys. Chem. Chem. Phys.* **19**, 32184–32215 (2017).
9. Weigend, F. and Ahlrichs, R. Balanced basis sets of split valence, triple zeta valence and quadruple zeta valence quality for H to Rn: Design and assessment of accuracy. *Phys. Chem. Chem. Phys.* **7**, 3297–3305 (2005).
10. Tomasi, J., Mennucci, B. and Cammi, R. Quantum mechanical continuum solvation models. *Chem. Rev.* **105**, 2999–3094 (2005).
11. Cossi, M., Rega, N., Scalmani, G. and Barone, V. Energies, structures, and electronic properties of molecules in solution with the C-PCM solvation model. *J. Comput. Chem.* **24**, 669–681 (2003).
12. Peng, C., Ayala, P. Y., Schlegel, H. B. and Frisch, M. J. Using redundant internal coordinates to optimize equilibrium geometries and transition states. *J. Comput. Chem.* **17**, 49–56 (1996).

13. Peng, C. and Bernhard Schlegel, H. Combining synchronous transit and quasi-newton methods to find transition states. *Isr. J. Chem.* **33**, 449–454 (1993).
14. Hratchian, H. P. and Schlegel, H. B. Using hessian updating To increase the efficiency of a hessian based predictor-corrector reaction path following method. *J. Chem. Theory Comput.* **1**, 61–69 (2005).
15. Hratchian, H. P. and Schlegel, H. B. Accurate reaction paths using a Hessian based predictor-corrector integrator. *J Chem Phys* **120**, 9918–9924 (2004).
16. Dykstra, C. E., *Theory and applications of computational chemistry: The first forty years*, Elsevier, Amsterdam, Boston, 2005.
17. Openeye toolkits 2022.2.1, (https://www.eyesopen.com).
18. Jmol: an open-source Java viewer for chemical structures in 3D, https://jmol.sourceforge.net (accessed 13 March, 2025).
19. Persistence of Vision (TM) Raytracer, https://www.povray.org, (accessed 11 April 2025).
20. Schotten, C., Nicholls, T. P., Bourne, R. A., Kapur, N., Nguyen, B. N. and Willans, C. E. Making electrochemistry easily accessible to the synthetic chemist. *Green Chem.* **22**, 3358–3375 (2020).
21. Song, H., Wang, Q., Wang, X., Zhang, Y., Duan, X.-H. and Hu, M. Efficient N-trifluoromethylation of amines with carbon disulfide and silver fluoride as reagents. *CC Chem* **7**, 381–391 (2025).
22. Schiesser, S., Chepliaka, H., Kollback, J., Quennesson, T., Czechtizky, W. and Cox, R. J. N-Trifluoromethyl amines and azoles: An underexplored functional group in the medicinal chemist's toolbox. *J. Med. Chem.* **63**, 13076–13089 (2020).
23. Yu, J., Lin, J.-H. and Xiao, J.-C. Reaction of thiocarbonyl fluoride generated from difluorocarbene with amines. *Angew. Chem. Int. Ed.* **56**, 16669–16673 (2017).
24. Zhen, L., Fan, H., Wang, X. and Jiang, L. Synthesis of thiocarbamoyl fluorides and isothiocyanates using $CF_3SiMe_3$ and elemental sulfur or $AgSCF_3$ and KBr with amines. *Org. Lett.* **21**, 2106–2110 (2019).
25. Stewart, W. E. and Siddall, T. H. Nuclear magnetic resonance studies of amides. *Chem. Rev.* **70**, 517–551 (1970).
26. Reeves, L. W. and Shaw, K. N. Nuclear magnetic resonance studies of multi-site chemical exchange. II. Hindered rotation in N,N-dimethyl carbamyl fluoride. *Can. J. Chem.* **49**, 3671–3682 (1971).
27. Taponard, A., Jarrosson, T., Khrouz, L., Médebielle, M., Broggi, J. and Tlili, A. Metal-free $SF_6$ activation: A new $SF_5$-based reagent enables deoxyfluorination and pentafluorosulfanylation reactions. *Angew. Chem. Int. Ed.* **61**, e202204623 (2022).
28. Onida, K., Vanoye, L. and Tlili, A. Direct synthesis of thiocarbamoyl fluorides and trifluoromethylamines through fluorinative desulfurization. *Eur. J. Org. Chem.* **2019**, 6106–6109 (2019).
29. Quan, Q., Ma, M., Wang, Z., Gu, Y. and Chen, M. Visible-light-enabled organocatalyzed controlled alternating terpolymerization of perfluorinated vinyl ethers. *Angew. Chem. Int. Ed.* **60**, 20443–20451 (2021).
30. Hu, C., et al. Synthesis of N-difluoromethyl carbonyl compounds from N-difluoromethylcarbamoyl fluorides. *Angew. Chem. Int. Ed.* **63**, e202319758 (2024).
31. Zhang, D., Ding, L.-A., Xiang, Q., Li, Z. and Wu, Q. PIII/PV-catalytic platform enabling N-fluoro-thiocarbonylation of diaryl amines for modular synthesis of tertiary N-trifluoromethylamines. *Chin. Chem. Lett.* **37**, 111461 (2026).
32. Liang, S., Wei, J., Jiang, L., Liu, J., Mumtaz, Y. and Yi, W. One-pot synthesis of trifluoromethyl amines and perfluoroalkyl amines with $CF_3SO_2Na$ and $RfSO_2Na$. *Chem. Commun.* **55**, 8536–8539 (2019).

33. Yao, Z., Cai, Z., Zhen, L. and Jiang, L. Cobalt-catalyzed switchable intramolecular thioenolization/C–H thiolation and C($sp^2$)–H/C($sp^3$)–H dehydrogenative coupling. *Org. Lett.* **22**, 4505–4510 (2020).
34. Xu, W., Liu, F., Li, J., Li, M., Xie, J. and Zhu, C. Thiocarbamoyl fluoride synthesis by deconstructive diversification of arylated tetrahydroisoquinolines. *J. Org. Chem.* **86**, 12443–12451 (2021).
35. Sokolnicki, T., Franczyk, A., Janowski, B. and Walkowiak, J. Synthesis of bio-based silane coupling agents by the modification of eugenol. *Adv. Synth. Catal.* **363**, 5493–5500 (2021).
36. Li, T.-T., Song, X.-H., Wang, M.-S. and Ma, N. Cerium ammonium nitrate-catalyzed aerobic oxidative coupling of dithiocarbamates: facile synthesis of thioureas and bis(aminothiocarbonyl)disulfides. *RSC Adv.* **4**, 40054–40060 (2014).
37. Liu, P., Li, C., Zhang, J. and Xu, X. Facile and versatile synthesis of alkyl and aryl isothiocyanates by using triphosgene and cosolvent. *Synth. Commun.* **43**, 3342–3351 (2013).
38. Guan, Z.-P., Yang, X.-X., Zhao, S.-Y., Yi, Z.-Q., Wu, Y.-X., Li, Y.-Y. and Dong, Z.-B. Conversion of acids to S-alkyl dithiocarbamates by decarboxylative sulfuration using visible-light photocatalysis. *Org. Lett.* **26**, 8323–8328 (2024).
39. Kapanda, C. N., Muccioli, G. G., Labar, G., Poupaert, J. H. and Lambert, D. M. Bis(dialkylaminethiocarbonyl)disulfides as potent and selective monoglyceride lipase inhibitors. *J. Med. Chem.* **52**, 7310–7314 (2009).
40. Sekirnik, R., Rose, N. R., Thalhammer, A., Seden, P. T., Mecinović, J. and Schofield, C. J. Inhibition of the histone lysine demethylase JMJD2A by ejection of structural Zn(ii). *Chem. Commun.*, 6376–6378 (2009).
41. Xu, H.-X., et al. Continuous-flow step-economical synthesis of thiuram disulfides via visible-light photocatalytic aerobic oxidation. *Green Chem.* **23**, 1280–1285 (2021).
42. Mondal, S., Mondal, S. and Saha, A. Thiuram disulfide mediated Cu-catalyzed amidation of terminal alkynes: An efficient synthesis of alkynyl amides. *J. Org. Chem.* **89**, 2182–2189 (2024).
