# Supplementary material for: Practical and divergent electrochemical access to thiocarbamoyl fluorides and N-trifluoromethyl amines from secondary amines
Source: Nat Commun. 2026 Jun 20;17:7762. doi: 10.1038/s41467-026-74660-2 (PMC13434417; doi:10.1038/s41467-026-74660-2)
Supplement: Supplementary file 2 [file 41467_2026_74660_MOESM2_ESM.pdf]

nature portfolio

Peer Review File

Practical and Divergent Electrochemical Access to Thiocarbamoyl Fluorides and N-Trifluoromethyl Amines from Secondary Amines

Corresponding Author: Professor Kevin Lam

This file contains all reviewer reports in order by version, followed by all author rebuttals in order by version.

Version 0:

Reviewer comments:

Reviewer #1

(Remarks to the Author)
In this manuscript, Lam and co-workers present an electrochemical method for the fluorination of amines, enabling access to thiocarbamoyl fluorides, N-CF_3 amines, and carbamoyl fluorides. The introduction of fluorine-containing motifs into organic scaffolds is of considerable interest in modern synthesis, and the authors' approach to electrochemically constructing the N–CF_3 unit is particularly noteworthy. Although several strategies have been previously reported for this transformation, many suffer from practical limitations—such as the reliance on stoichiometric silver fluoride to convert N–CF=S intermediates into N–CF_3 groups. The authors provide a clear and concise overview of these existing methods, effectively contextualizing their contribution. The results are generally convincing. However, major revisions are necessary before this manuscript can be considered suitable for publication, as outlined below.
1.Limited Substrate Scope: The current study focuses exclusively on alkyl amines. To better demonstrate the generality and utility of this method, the authors should extend the scope to include aryl amines. Investigating the electronic effects of substituents on the aromatic ring would significantly strengthen the mechanistic discussion and practical relevance.
2.Lack of Functional Group Tolerance: All substrates tested are relatively simple, small molecules. It would be valuable to examine more complex substrates bearing diverse functional groups, which would better illustrate the method's compatibility and potential for late-stage functionalization.
3.Utility of N–CF_3 Products: As indicated in Scheme 2C, the N–CF_3 products appear to exhibit limited stability. Given the significant effort required to install the N-CF_3 group, its transformation into the seemingly less useful N-CF=O moiety represents a poor return on synthetic investment. It would be greatly beneficial if the authors could explore further transformations of the N–CF=O unit into more versatile functional groups, thereby enhancing the utility of the overall strategy.
4.Technical Corrections:
In Scheme 1D, DABCO should be clearly indicated under the first arrow for clarity.
In Scheme 3D, the proposed mechanism mislabels Br^+ as a by-product in the conversion of M2 to M3; it should be designated as a reagent.
The placement of Scheme 3E within the area of Scheme 3D is confusing and makes it difficult to locate; it should be repositioned for better readability.

Reviewer #2

(Remarks to the Author)
The group of Lam reports an interesting electrochemical protocol for the fluorinative functionalisation of amines, enabling the synthesis of thiocarbamoyl fluoride and trifluoromethylamine derivatives. Using Et3N·3HF as a convenient fluoride source, the method proceeds via in situ electrooxidation to thiuram intermediates, which can be selectively converted to thiocarbamoyl fluorides or further electrolyzed to afford trifluoromethylamines. The process exhibits good functional group tolerance and offers a practical route to diverse fluorinated amines, highlighting its potential for late-stage functionalization in medicinal chemistry and materials science. This work is well-designed and methodologically robust, providing notable advantages in operational safety and simplicity. The compelling results constitute a valuable contribution to the fields of electrochemical fluorination and amine functionalization. The manuscript can be considered for publication after the revisions outlined below.

1. Examples of aromatic amines and primary amines are not provided. How reactive are these types of substrates, and can they yield the desired products? If not, were any other byproducts observed?
2. What is the Faradaic efficiency of the model reaction?
3. Apart from Et3N·3HF, have other fluoride sources been screened, and could the corresponding products also be obtained?
4. What is the stability profile of the generated thiocarbamoyl fluorides under ambient or storage conditions?
5. The authors suggest that bromide ions (Br-) may be anodically oxidized to bromonium species (Br+) to participate in the reaction. Has the generation of bromine radicals (Br•) or molecular bromine (Br2) been detected? Could the specific mechanistic role be further clarified through control experiments, such as adding radical scavengers or using different halide salts?
6. The reaction can selectively yield either thiocarbamoyl fluorides or trifluoromethylamines. Is this selectivity controlled solely by the charge passed, or could it also be potential-dependent? Could additional controlled experiments at different electrolysis stages be provided to illustrate the conversion of intermediates?
7. In Scheme 3, figs. F, G, and H are too small. It is recommended to enlarge them or move fig. H to the Supporting Information.

Reviewer #3

(Remarks to the Author)
This manuscript by Lam and coworkers reports a unified and scalable electrochemical platform enabling divergent synthesis of thiocarbamoyl fluorides and N-trifluoromethyl amines from simple secondary amines under mild conditions. The approach avoids expensive and unsafe reagents and demonstrates broad substrate tolerance and gram-scale applicability, highlighting promising utility in medicinal and materials chemistry. The practical relevance of this work is significant. The manuscript shows potential to make a broad impact in synthetic methodology and sustainable chemistry, and may become appropriate for publication in Nature Communications.
However, in its current form, the manuscript exhibits major shortcomings in presentation and scientific communication that undermine both clarity and rigor. The claimed performance advantages over state-of-the-art methods are not sufficiently supported by quantitative comparisons. Moreover, the proposed mechanistic rationale, particularly the central bromide-mediated catalytic cycle, contains several significant chemical and electrochemical inconsistencies that substantially weaken its credibility. Addressing these mechanistic issues is essential prior to publication.
Therefore, I believe that the manuscript may be suitable for publication in Nature Communications after major revision.
1) The following points are provided as suggestions for improvement: In Figure 1D, the carbamoyl fluoride products seem to arise from derivatization of the trifluoromethylated amines rather than a direct transformation. Moreover, the manuscript provides no evidence supporting electrocatalytic involvement in this step. Therefore, the authors should consider revising the manuscript title and restructuring the figure to accurately reflect the experimental pathway.
2) In this paper, the authors primarily focus on the substrate scope of alkyl-substituted secondary amines. It would be helpful if they could clarify whether primary amines or diaryl amines are also compatible with the reaction conditions, and provide some representative examples.
3) Please perform control experiments without applying current to verify whether any product is formed under these conditions. Please provide a more detailed discussion and explicitly highlight the advantages of your method in comparison with Tyrra's report (J. Fluor. Chem. 109, 189-194 (2001)).
4) Regarding the proposed mechanism, the bromide cycle involved in the formation of M3 is not clearly understood. The authors should provide a more detailed explanation, along with additional evidence to support a Br^+-based pathway.
5) The manuscript exhibits substantial inconsistencies in figure ordering and substrate descriptions, which detract from the scientific clarity of the work. Comprehensive revision is required to address these issues.
6) The manuscript contains inconsistent font sizes throughout the text. Please revise to ensure uniform formatting.
7) A considerable number of substrates provided yields lower than 60%. Could the authors comment on the possible reasons for these relatively low efficiencies?
8) The study primarily investigates simple substrates; evaluation of more complex natural products would significantly strengthen the work. In addition, the limited number of trifluoromethylated examples does not sufficiently support the claim of a broad substrate scope.
9) The manuscript contains numerous writing errors, such as unmatched or incomplete parentheses. These should be carefully checked and corrected.
10) In application parts, the author claim that "Carbon–fluorine bonds are generally considered highly stable and unreactive…...suitably reactive nucleophiles." The authors focus their derivatization mainly on thiocarbamoyl fluorides. However, the C–F bond in thiocarbamoyl fluorides is generally considered reactive due to the electrophilic nature of the carbon adjacent to the C=S group. Therefore, the statement regarding the stability or inertness of this C–F bond seems inaccurate and should be revised to reflect its known chemical lability toward nucleophiles.
11) Many NMR spectra in the Supporting Information require correction. Several spectra, including 2c, 2n, 2q, 2r, 2t, 4b, 4c, and M2, have mislabeled chemical structures or missing/incorrect proton integrations. For compounds exhibiting rotamers, variable-temperature NMR should be performed to obtain cleaner, well-resolved spectra. The authors should revise the SI to ensure all spectra are accurately assigned.
12) The observation that Et_4NCl improves yield while Et_4NI is ineffective is interesting. A rational on why chloride is effective would strengthen the mechanistic discussion.
13) Please ensure that all references are formatted according to the journal's guidelines. In addition, please revise the citation list appropriately, as ref. 36 and 37, according to the reviewer, do not contribute to the mechanistic discussion and therefore do not support the explanation provided.

14) The Scheme 3 requires improved layout and resolution. At present, the images remain unclear even when enlarged fivefold, which makes it difficult for reviewers to examine the data.

Version 1:

Reviewer comments:

Reviewer #1

(Remarks to the Author)
I have examined the authors' detailed revisions and point-by-point responses to my comments. The authors have addressed the concerns raised, made appropriate improvements to the manuscript, and provided explanations for each modification. The overall quality of the paper has been enhanced. I therefore recommend accepting the manuscript for publication.

Reviewer #2

(Remarks to the Author)
The author has addressed all of my concerns. I recommend publishing it as is.

Reviewer #3

(Remarks to the Author)
The authors have thoroughly addressed the reviewers' comments and suggestions, and the manuscript has been improved accordingly. It can be accepted in its current form.

Open Access This Peer Review File is licensed under a Creative Commons Attribution 4.0 International License, which permits use, sharing, adaptation, distribution and reproduction in any medium or format, as long as you give appropriate credit to the original author(s) and the source, provide a link to the Creative Commons license, and indicate if changes were made.
In cases where reviewers are anonymous, credit should be given to 'Anonymous Referee' and the source.
The images or other third party material in this Peer Review File are included in the article's Creative Commons license, unless indicated otherwise in a credit line to the material. If material is not included in the article's Creative Commons license and your intended use is not permitted by statutory regulation or exceeds the permitted use, you will need to obtain permission directly from the copyright holder.
To view a copy of this license, visit https://creativecommons.org/licenses/by/4.0/

Faculty of Engineering and Science
School of Science
Kevin Lam, PhD.
Professor of Synthetic Electrochemistry

Point-by-point response to the reviewers' comments

Reviewer comments are reproduced in full or in substance, followed by our responses.

Reviewer #1 (Remarks to the Author):

In this manuscript, Lam and co-workers present an electrochemical method for the fluorination of amines, enabling access to thiocarbamoyl fluorides, N-CF_3 amines, and carbamoyl fluorides. The introduction of fluorine-containing motifs into organic scaffolds is of considerable interest in modern synthesis, and the authors' approach to electrochemically constructing the N–CF_3 unit is particularly noteworthy. Although several strategies have been previously reported for this transformation, many suffer from practical limitations—such as the reliance on stoichiometric silver fluoride to convert N–CF=S intermediates into N–CF_3 groups. The authors provide a clear and concise overview of these existing methods, effectively contextualizing their contribution. The results are generally convincing. However, major revisions are necessary before this manuscript can be considered suitable for publication, as outlined below.

1A. Limited Substrate Scope: The current study focuses exclusively on alkyl amines. To better demonstrate the generality and utility of this method, the authors should extend the scope to include aryl amines. B. Investigating the electronic effects of substituents on the aromatic ring would significantly strengthen the mechanistic discussion and practical relevance.

We thank the Reviewer very much for this helpful and insightful suggestion. We investigated aryl-substituted substrates in the revised study. Direct use of aromatic amines did not provide the desired thiocarbamoyl fluorides or *N*-trifluoromethyl amines, which we attribute to the lower nucleophilicity of aryl amines and to the reversibility of the corresponding aryl dithiocarbamate formation under the reaction conditions. By contrast, when we employed the preformed carbamodithioate precursor **1aa** (R_2NCSSR), we obtained the corresponding thiocarbamoyl fluoride **2y** in 66% yield and the *N*-trifluoromethyl amine **3aa** in 34% yield.

During these experiments, we also observed substantial formation of the corresponding carbamothioate byproduct **2y**' (R_2NCSOR), which limited further extension of this substrate class. We have therefore included the successful representative aryl-derived examples **2y** and **3aa** in the revised manuscript and now discuss this limitation explicitly.

To probe the electronic effect of substituents on the aromatic ring, we also examined electron-withdrawing substrate **B40** and electron-donating substrate **B42** (see Supplementary Table 4), again using the corresponding carbamodithioate precursors. In both cases, formation of the N-trifluoromethyl amine was observed. However, substantial formation of the carbamothioate byproduct, together with very similar TLC behaviour, prevented clean isolation of the desired products. We now clarify this point in the revised manuscript.

Lack of Functional Group Tolerance: All substrates tested are relatively simple, small molecules. It would be valuable to examine more complex substrates bearing diverse functional groups, which would better illustrate the method's compatibility and potential for late-stage functionalization.

We thank the Reviewer very much for this helpful suggestion. We have expanded the scope to include more complex examples. In the thiocarbamoyl fluoride series, we have added the fluoxetine-derived substrate **2w**. In

+44 (0)208 331 8435
www.lamresearchgroup.com
k.lam@greenwich.ac.uk
Grenville G211.
University of Greenwich, a charity and company limited by guarantee, registered in England (reg. no. 986729).
Registered office: Old Royal Naval College, Park Row, Greenwich, London SE10 9LS
Medway Campus
Central Avenue
Chatham Maritime
Kent ME4 4TB
Tel: +44 (0)20 8331 8000

Faculty of Engineering and Science
School of Science
Kevin Lam, PhD.
Professor of Synthetic Electrochemistry

the *N*-trifluoromethyl amine series, we have added compounds **3l-3z** to broaden the discussion of late-stage and functional-group tolerance.

A limitation of the *N*-trifluoromethyl amine series is product handling and purification. These compounds are known to be intrinsically less stable and can undergo HF elimination or hydrolysis under mildly acidic or polar conditions, which makes them prone to decomposition on silica gel.[1-4] Consequently, purification by column chromatography or, in some cases, by simple Celite filtration was unsuccessful. For these substrates, yields are therefore reported from NMR analysis of the crude reaction mixtures. We now state this limitation more clearly in the revised manuscript and Supporting Information.

Utility of N–CF_3 Products: As indicated in Scheme 2C, the N–CF_3 products appear to exhibit limited stability. Given the significant effort required to install the N-CF_3 group, its transformation into the seemingly less useful N-CF=O moiety represents a poor return on synthetic investment. It would be greatly beneficial if the authors could explore further transformations of the N–CF=O unit into more versatile functional groups, thereby enhancing the utility of the overall strategy.

We thank the Reviewer very much for this important point. We agree that demonstrating synthetic utility is valuable. There are numerous precedents in the literature showing that carbamoyl fluoride derivatives can be converted into a range of useful products.[3,5-10] In response, we have now included an additional proof-of-concept transformation in Scheme 2D, namely the synthesis of *S*-carbamothioate **4d**.

Technical Corrections: In Scheme 1D, DABCO should be clearly indicated under the first arrow for clarity. In Scheme 3D, the proposed mechanism mislabels Br^+ as a by-product in the conversion of M2 to M3; it should be designated as a reagent. The placement of Scheme 3E within the area of Scheme 3D is confusing and makes it difficult to locate; it should be repositioned for better readability.

We thank the Reviewer very much for these helpful corrections. Scheme 1D has been revised to indicate DABCO clearly, the role of Br^+ in Scheme 3D has been corrected, and the placement of Scheme 3E has been adjusted to improve readability.

Reviewer #2 (Remarks to the Author):

The group of Lam reports an interesting electrochemical protocol for the fluorinative functionalisation of amines, enabling the synthesis of thiocarbamoyl fluoride and trifluoromethylamine derivatives. Using $Et_3N{\cdot}3HF$ as a convenient fluoride source, the method proceeds via in situ electrooxidation to thiuram intermediates, which can be selectively converted to thiocarbamoyl fluorides or further electrolyzed to afford trifluoromethylamines. The process exhibits good functional group tolerance and offers a practical route to diverse fluorinated amines, highlighting its potential for late-stage functionalization in medicinal chemistry and materials science. This work is well-designed and methodologically robust, providing notable advantages in operational safety and simplicity. The compelling results constitute a valuable contribution to the fields of electrochemical fluorination and amine functionalization. The manuscript can be considered for publication after the revisions outlined below.

Examples of aromatic amines and primary amines are not provided. How reactive are these types of substrates, and can they yield the desired products? If not, were any other byproducts observed?

+44 (0)208 331 8435
www.lamresearchgroup.com
k.lam@greenwich.ac.uk
Grenville G211.

University of Greenwich, a charity and company limited by guarantee, registered in England (reg. no. 986729).
Registered office: Old Royal Naval College, Park Row, Greenwich, London SE10 9LS

Medway Campus
Central Avenue
Chatham Maritime
Kent ME4 4TB
Tel: +44 (0)20 8331 8000

Faculty of Engineering and Science
School of Science
Kevin Lam, PhD.
Professor of Synthetic Electrochemistry

We thank the Reviewer very much for raising this important point. The reactivity of aromatic amines is addressed in our response to Reviewer 1, Comment 1. For primary amines, our standard conditions predominantly gave isothiocyanate derivatives as the major stable products, as detected by GC-MS. We attribute this to in situ decomposition of the corresponding thiocarbamoyl fluoride intermediates, in line with prior literature reports.[11-14]

What is the Faradaic efficiency of the model reaction?

We thank the Reviewer very much for this question. The Faradaic efficiencies for the model reactions were 65% for thiocarbamoyl fluoride **2a** and 23% for *N*-trifluoromethyl amine **3a**. The corresponding calculations have now been included in the Supplementary Information.

Apart from $Et_3N{\cdot}3HF$, have other fluoride sources been screened, and could the corresponding products also be obtained?

We thank the Reviewer very much for this helpful question. In addition to $Et_3N{\cdot}3HF$, we screened TBAF·3HF, NH_4F, BF_3OEt_2, CF_3CO_2Na, $TMSCF_3$, $TBAH_2F_3$, TMAF, CsF, and KF. In all cases, the yield was very low or the desired product was not detected. These additional entries have now been added to Table 1.

What is the stability profile of the generated thiocarbamoyl fluorides under ambient or storage conditions?

We thank the Reviewer very much for this question. The representative thiocarbamoyl fluoride was stored in air at room temperature for more than six months without special precautions and remained bench-stable by ^1H NMR analysis. More broadly, the stability of thiocarbamoyl fluorides in organic media, aqueous media, physiologically relevant media, acidic and basic conditions, and in the presence of nucleophilic amino acids has recently been studied in detail by Billard and co-workers, supporting the high stability of this class of compounds under a wide range of conditions.[14]

The authors suggest that bromide ions (Br^-) may be anodically oxidised to bromonium species (Br^+) to participate in the reaction. Has the generation of bromine radicals (Br•) or molecular bromine (Br_2) been detected? Could the specific mechanistic role be further clarified through control experiments, such as adding radical scavengers or using different halide salts?

We thank the Reviewer very much for this important mechanistic question. In the presence of TEMPO, we observed the formation of the TEMPO adduct [Bn_2NCS_2-TEMPO] together with the complete suppression of the desired product. We did not detect a bromine-radical TEMPO adduct, likely because such species are known to be unstable under related conditions, as discussed by Miyake and co-workers.[15]

We have therefore revised the mechanistic discussion to be more precise. Literature precedent supports anodic oxidation of bromide in organic media to a family of reactive bromine species, including Br·, Br^+, Br_2, and Br_3^-.[16-19] Our proposal is accordingly framed as a bromide-mediated pathway, with the Br^+-type formulation used as a simplified representation of the electrophilic bromine species involved in the fluorodesulfurisation sequence.

A. The reaction can selectively yield either thiocarbamoyl fluorides or trifluoromethylamines. Is this selectivity controlled solely by the charge passed, or could it also be potential-dependent? B. Could additional controlled experiments at different electrolysis stages be provided to illustrate the conversion of intermediates?

+44 (0)208 331 8435
www.lamresearchgroup.com
k.lam@greenwich.ac.uk
Grenville G211.

Medway Campus
Central Avenue
Chatham Maritime
Kent ME4 4TB
Tel: +44 (0)20 8331 8000

University of Greenwich, a charity and company limited by guarantee, registered in England (reg. no. 986729).
Registered office: Old Royal Naval College, Park Row, Greenwich, London SE10 9LS

Faculty of Engineering and Science
School of Science
Kevin Lam, PhD.
Professor of Synthetic Electrochemistry

We thank the Reviewer very much for this insightful question. Selectivity is not governed by charge alone. Although we identify two general sets of conditions for the divergent synthesis, several substrates required further adjustment of parameters such as current density and the amount of bromide mediator in order to favour selective thiocarbamoyl fluoride formation.

By contrast, higher loadings of $Et_3N{\cdot}3HF$ (3.0 equiv.) and Et_4NBr (1.0 equiv.) favour formation of the N-trifluoromethyl amine.

To address the Reviewer's suggestion directly, we have now included staged electrolysis experiments that illustrate the conversion of key intermediates: formation of the dimer M2 from the amine salt (Scheme 3A), conversion of M2 to thiocarbamoyl fluoride 2a (Scheme 3D1), and subsequent conversion of 2a to N-trifluoromethyl amine 3a (Scheme 3D2 and Supplementary Fig. 10b by ^{19}F NMR). Under the latter conditions, formation of the N-CF_3 product becomes evident from approximately 11 F onward.

In Scheme 3, figs. F, G, and H are too small. It is recommended to enlarge them or move fig. H to the Supporting Information.

We thank the Reviewer for this suggestion. These panels have been enlarged and the layout of Scheme 3 has been revised for clarity.

Reviewer #3 (Remarks to the Author):

This manuscript by Lam and coworkers reports a unified and scalable electrochemical platform enabling divergent synthesis of thiocarbamoyl fluorides and N-trifluoromethyl amines from simple secondary amines under mild conditions. The approach avoids expensive and unsafe reagents and demonstrates broad substrate tolerance and gram-scale applicability, highlighting promising utility in medicinal and materials chemistry. The practical relevance of this work is significant. The manuscript shows potential to make a broad impact in synthetic methodology and sustainable chemistry, and may become appropriate for publication in Nature Communications. However, in its current form, the manuscript exhibits major shortcomings in presentation and scientific communication that undermine both clarity and rigor. The claimed performance advantages over state-of-the-art methods are not sufficiently supported by quantitative comparisons. Moreover, the proposed mechanistic rationale, particularly the central bromide-mediated catalytic cycle, contains several significant chemical and electrochemical inconsistencies that substantially weaken its credibility. Addressing these mechanistic issues is essential prior to publication. Therefore, I believe that the manuscript may be suitable for publication in Nature Communications after major revision.

The following points are provided as suggestions for improvement: In Figure 1D, the carbamoyl fluoride products seem to arise from derivatization of the trifluoromethylated amines rather than a direct transformation. Moreover, the manuscript provides no evidence supporting electrocatalytic involvement in this step. Therefore, the authors should consider revising the manuscript title and restructuring the figure to accurately reflect the experimental pathway.

We thank the Reviewer very much for this thoughtful suggestion. The title has been revised and Scheme 1D has been restructured so that it reflects the experimental sequence more accurately.

Faculty of Engineering and Science
School of Science
Kevin Lam, PhD.
Professor of Synthetic Electrochemistry

In this paper, the authors primarily focus on the substrate scope of alkyl-substituted secondary amines. It would be helpful if they could clarify whether primary amines or diaryl amines are also compatible with the reaction conditions, and provide some representative examples.

We thank the Reviewer very much for raising this point. The reactivity of aromatic amines is addressed in our response to Reviewer 1, Comment 1, and the behaviour of primary amines is addressed in our response to Reviewer 2, Comment 1.

A. Please perform control experiments without applying current to verify whether any product is formed under these conditions. B. Please provide a more detailed discussion and explicitly highlight the advantages of your method in comparison with Tyrra's report (J. Fluor. Chem. 109, 189-194 (2001).

We thank the Reviewer very much for this helpful suggestion. We performed the reaction under otherwise identical conditions, without applying current, and observed no product formation. This control experiment has now been added to Table 1. We recovered 25% of the starting amine; the remainder is consistent with formation of the corresponding salt M1, which is not recovered during work-up.

Tyrra's 2001 report describes a stoichiometrically controlled desulfurisation-fluorination sequence using AgF to access thiocarbamoyl fluorides and *N*-trifluoromethyl amines from preformed thiuram disulfides or silver dithiocarbamates.[20] While important, that approach relies on expensive silver fluoride and on prefunctionalised amine precursors.

By contrast, our method is a one-pot electrochemical protocol that starts directly from secondary amines and uses $Et_3N{\cdot}3HF$ as the fluoride source. It avoids stoichiometric AgF, avoids pre-isolation of thiuram disulfides or silver dithiocarbamates, and proceeds under operationally simple conditions. We have revised the manuscript to make this comparison with Tyrra's work more explicit.

Regarding the proposed mechanism, the bromide cycle involved in the formation of M3 is not clearly understood. The authors should provide a more detailed explanation, along with additional evidence to support a Br^+-based pathway.

We thank the Reviewer very much for this important mechanistic comment. As noted above, the exact nature of the active bromine species in solution is likely more nuanced than a single discrete formulation. Nevertheless, literature precedent supports the generation of strongly electrophilic bromine species upon anodic oxidation of bromide.[18,21] In addition, our DFT calculations support involvement of a Br^+-type pathway: the bromine-coordinated intermediates M3 and M3a are lower in energy than the corresponding pathway that does not involve bromine coordination. We now explain this point more carefully in the revised mechanistic discussion.

The manuscript exhibits substantial inconsistencies in figure ordering and substrate descriptions, which detract from the scientific clarity of the work. Comprehensive revision is required to address these issues.

We thank the Reviewer very much for identifying these presentation issues. The figure order, substrate descriptions, and related cross-references have been checked carefully and corrected throughout the revised manuscript.

The manuscript contains inconsistent font sizes throughout the text. Please revise to ensure uniform formatting.

+44 (0)208 331 8435
www.lamresearchgroup.com
k.lam@greenwich.ac.uk
Grenville G211.
University of Greenwich, a charity and company limited by guarantee, registered in England (reg. no. 986729).
Registered office: Old Royal Naval College, Park Row, Greenwich, London SE10 9LS

Medway Campus
Central Avenue
Chatham Maritime
Kent ME4 4TB
Tel: +44 (0)20 8331 8000

Faculty of Engineering and Science
School of Science
Kevin Lam, PhD.
Professor of Synthetic Electrochemistry

We thank the Reviewer very much for noting this. Font sizes and formatting have been standardised throughout the revised manuscript.

A considerable number of substrates provided yields lower than 60%. Could the authors comment on the possible reasons for these relatively low efficiencies

We thank the Reviewer very much for this important point. For the thiocarbamoyl fluoride series, lower isolated yields often reflect purification challenges on small scale, because the desired products can have polarity very similar to that of the accompanying impurities. In some cases, partial over-oxidation of the product also contributes to diminished yield.

For the *N*-trifluoromethyl amine series, lower yields mainly reflect the intrinsic instability of these products, particularly toward HF elimination and hydrolysis, together with losses during handling and purification.[1-4,22] In some cases, the products also showed significant retention on the Celite pad during filtration. We have added this discussion to the revised manuscript.

The study primarily investigates simple substrates; evaluation of more complex natural products would significantly strengthen the work. In addition, the limited number of trifluoromethylated examples does not sufficiently support the claim of a broad substrate scope.

We thank the Reviewer very much for this helpful suggestion. The revised manuscript now includes additional complex and late-stage examples in the *N*-trifluoromethyl amine series, as well as a list of unsuccessful substrates in the SIs. These additions better define both the scope and the current limitations of the method.

The manuscript contains numerous writing errors, such as unmatched or incomplete parentheses. These should be carefully checked and corrected.

We thank the Reviewer very much for highlighting these issues. The manuscript has been carefully proofread and corrected throughout.

In application parts, the author claim that "Carbon–fluorine bonds are generally considered highly stable and unreactive……suitably reactive nucleophiles." The authors focus their derivatization mainly on thiocarbamoyl fluorides. However, the C–F bond in thiocarbamoyl fluorides is generally considered reactive due to the electrophilic nature of the carbon adjacent to the C=S group. Therefore, the statement regarding the stability or inertness of this C–F bond seems inaccurate and should be revised to reflect its known chemical lability toward nucleophiles.

We thank the Reviewer very much for this important correction. We agree that the earlier wording was too broad in the context of thiocarbamoyl fluorides. We have therefore revised the statement to read: "the thiocarbamoyl fluorides proved to be versatile intermediates that could be further elaborated through reaction with suitable nucleophiles."

Many NMR spectra in the Supporting Information require correction. Several spectra, including 2c, 2n, 2q, 2r, 2t, 4b, 4c, and M2, have mislabeled chemical structures or missing/incorrect proton integrations. For compounds exhibiting rotamers, variable-temperature NMR should be performed to obtain cleaner, well-resolved spectra. The authors should revise the SI to ensure all spectra are accurately assigned.

+44 (0)208 331 8435
www.lamresearchgroup.com
k.lam@greenwich.ac.uk
Grenville G211.
University of Greenwich, a charity and company limited by guarantee, registered in England (reg. no. 986729).
Registered office: Old Royal Naval College, Park Row, Greenwich, London SE10 9LS

Medway Campus
Central Avenue
Chatham Maritime
Kent ME4 4TB
Tel: +44 (0)20 8331 8000

Faculty of Engineering and Science
School of Science
Kevin Lam, PhD.
Professor of Synthetic Electrochemistry

We thank the Reviewer very much for identifying these issues. The Supporting Information has been revised carefully throughout. We corrected mislabeled structures, missing or inaccurate integrations, and assignments for compounds that display rotameric mixtures. Consistent with the literature precedent reported by Billard and co-workers,[14] the affected compounds are now described and labelled as rotameric pairs: 2j'/2j'' (formerly 2c), 2g'/2g'' (formerly 2n), 2h'/2h'' (formerly 2q), 2i'/2i'' (formerly 2r), 2f'/2f'' (formerly 2t), 4b'/4b'' (formerly 4b), 4c'/4c'' (formerly 4c), and M2. We did not perform variable-temperature NMR measurements for every case, but we have corrected the assignments and presentation throughout the SI to ensure consistency and clarity.

The observation that Et_4NCl improves yield while Et_4NI is ineffective is interesting. A rational on why chloride is effective would strengthen the mechanistic discussion.

We thank the Reviewer very much for this observation. This point arose from a typographical error in the original submission. Et_4NCl is not genuinely effective in this transformation; its yield is only marginally different from that obtained with Et_4NI. In practice, Et_4NBr is the only halide additive that produces a substantial beneficial effect. We now clarify this point in the revised manuscript and note that this behaviour is consistent with favourable interaction between sulfur, a softer donor, and an electrophilic bromine species.[23]

Please ensure that all references are formatted according to the journal's guidelines. In addition, please revise the citation list appropriately, as ref. 36 and 37, according to the reviewer, do not contribute to the mechanistic discussion and therefore do not support the explanation provided.

We thank the Reviewer very much for this helpful suggestion. The references have been reformatted to match the journal style, and the non-supporting references previously cited in this mechanistic context have been removed.

The Scheme 3 requires improved layout and resolution. At present, the images remain unclear even when enlarged fivefold, which makes it difficult for reviewers to examine the data.

We thank the Reviewer very much for this comment and apologise for the poor image quality in the original submission. Scheme 3 has now been rebuilt with improved layout and higher-resolution panels.

Bibliography

1. Fu, J.-L., Wu, J.-Y., Shi, J.-F., Huang, L.-L., Wang, H. and Li, Q. A mild and practical approach to N-CF_3 secondary amines via oxidative fluorination of isocyanides. Nat. Commun. 16, 4873 (2025).

2. Kubíčková, A., Dračínský, M., Stroka, M., Puzzini, L., Kurčina, A., Andris, E., Štěpánová, S., Kašička, V., Klepetářová, B. and Beier, P. Stable Secondary N-(Per)fluoroalkylamides from 1,2,3-Triazoles via Ketenimines. ChemRxiv 2025 (2025).

3. Wycich, G., Ponce-de-León, J., Liu, L. and Schoenebeck, F. Desulfurizative Fluorination of N-CF_3 Thioformamides for the Efficient Synthesis of N(CF_3)(CF_2H) Amines with Enhanced Stability. Angew. Chem. Int. Ed. 64, e202506154 (2025).

4. Schiesser, S., Chepliaka, H., Kollback, J., Quennesson, T., Czechtizky, W. and Cox, R. J. N-Trifluoromethyl Amines and Azoles: An Underexplored Functional Group in the Medicinal Chemist's Toolbox. J. Med. Chem. 63, 13076–13089 (2020).

5. Scattolin, T., Bouayad-Gervais, S. and Schoenebeck, F. Straightforward access to N-trifluoromethyl amides, carbamates, thiocarbamates and ureas. Nature 573, 102–107 (2019).

6. DesMarteau, D. D. and Lu, C. Synthesis of N,N-bis(trifluoromethyl)amino difluoromethylene trifluorovinyl ether. J. Fluor. Chem. 132, 1194–1197 (2011).

+44 (0)208 331 8435
www.lamresearchgroup.com
k.lam@greenwich.ac.uk
Grenville G211.

University of Greenwich, a charity and company limited by guarantee, registered in England (reg. no. 986729).
Registered office: Old Royal Naval College, Park Row, Greenwich, London SE10 9LS

Medway Campus
Central Avenue
Chatham Maritime
Kent ME4 4TB
Tel: +44 (0)20 8331 8000

Faculty of Engineering and Science
School of Science
Kevin Lam, PhD.
Professor of Synthetic Electrochemistry

UNIVERSITY OF GREENWICH

7. Zivkovic, F. G., Bahns, F., Hsu, C.-M. and Schoenebeck, F. Access to N-Monofluoromethylated (Thio)Carbamates, Formamides, Alkynamides, and Related Derivatives. Angew. Chem. Int. Ed. 64, e202505478 (2025).

8. Ma, L., Jiang, L., Tian, G., Lin, Z., Lin, Z., Xu, Y., Guo, Z., Wang, L., Liu, J. and Yi, W. Synthesis of a Library of N-monofluoromethyl Compounds from N-monofluoromethyl Carbamoyl/Thiocarbamoyl Fluorides. Angew. Chem. Int. Ed. 64, e202508594 (2025).

9. Hu, C., Jiang, L., Guo, Z., Mumtaz, Y., Liu, J., Qin, J., Chen, Y., Lin, Z. and Yi, W. Synthesis of N-Difluoromethyl Carbonyl Compounds from N-Difluoromethylcarbamoyl Fluorides. Angew. Chem. Int. Ed. 63, e202319758 (2024).

10. Bouayad-Gervais, S., Scattolin, T. and Schoenebeck, F. N-Trifluoromethyl Hydrazines, Indoles and Their Derivatives. Angew. Chem. Int. Ed. 59, 11908–11912 (2020).

11. Boas, U., Pedersen, B. and Christensen, J. r. B. Tetramethyl Fluoro Formamidinium Hexafluorophoshate – An Improved Synthesis and Some New Uses. Synth. Commun. 28, 1223–1231 (1998).

12. Boas, U. and Jakobsen, M. H. A new synthesis of aliphatic isothiocyanates from primary amines, convenient for in situ use. J. Chem. Soc., Chem. Commun., 1995–1996 (1995).

13. Yu, J., Lin, J.-H. and Xiao, J.-C. Reaction of Thiocarbonyl Fluoride Generated from Difluorocarbene with Amines. Angew. Chem. Int. Ed. 56, 16669–16673 (2017).

14. Delobel, C., Chefdeville, E., Toulgoat, F. and Billard, T. Deep study of thiocarbamoyl fluorides: Synthesis and properties. Adv. Synth. Catal. 366, 3474–3480 (2024).

15. Chen, D.-F., Chrisman, C. H. and Miyake, G. M. Bromine Radical Catalysis by Energy Transfer Photosensitization. ACS Catal. 10, 2609–2614 (2020).

16. Fuchigami, T. and Sano, M. Electrolytic partial fluorination of organic compounds. Part 16. Selective indirect anodic fluorination of dithioacetals using a bromine mediator. J. Electroanal. Chem. 414, 81–84 (1996).

17. Takahashi, K., Inagi, S. and Fuchigami, T. Electrochemical Fluorination Using Halogen Mediators in Ionic Liquid Hydrogen Fluoride Salt. J. Electrochem. Soc. 160, G3046 (2013).

18. Takiguchi, T. and Nonaka, T. Influence of Br− Concentration on $(Br)^+$-Mediated Indirect Electrooxidation of Alcohols to the Corresponding Carbonyl Compounds. Bull. Chem. Soc. Jpn. 60, 3137–3142 (2006).

19. Konno, A., Fukui, K., Fuchigami, T. and Nonaka, T. Anodic generation of polybromochloride ions and their utilization as precisely-controlled oxidizing reagents for ex-cell. Indirect electrooxidation of alcohols. Tetrahedron 47, 887–894 (1991).

20. Tyrra, W. Die Desulfonierung–Fluorierung von Thiuramdisulfiden, $[R_2NC(S)S]_2$ und Silberdithiocarbamaten, $Ag[SC(S)NR_2]$ (R = CH_3, CH_3CH_2, $C_6H_5CH_2$), mit Silber(I)fluorid, AgF — ein einfacher Zugang zu Diorgano(trifluormethyl)aminen, R_2NCF_3, und Thiocarbamoylfluoriden, $R_2NC(S)F$. J. Fluor. Chem. 109, 189–194 (2001).

21. Mitsudo, K., Tachibana, Y., Sato, E. and Suga, S. Electrochemical Synthesis of Dibenzothiophenes via Intramolecular C–S Cyclization with a Halogen Mediator. Org. Lett. 24, 8547–8552 (2022).

22. Song, H., Wang, Q., Ruan, X., Hu, W., Liu, L., Duan, X.-H. and Hu, M. The synthesis of N-Trifluoromethyl hydroxylamine from (trifluoromethyl)trimethylsilane. J. Fluor. Chem. 280, 110356 (2024).

23. Hagooly, Y., Gatenyo, J., Hagooly, A. and Rozen, S. Toward the Synthesis of the Rare N-(Trifluoromethyl)amides and the N-(Difluoromethylene)-N-(trifluoromethyl)amines $[RN(CF_3)CF_2R']$ Using BrF_3. J. Org. Chem. 74, 8578–8582 (2009).

+44 (0)208 331 8435

www.lamresearchgroup.com

k.lam@greenwich.ac.uk

Grenville G211.

University of Greenwich, a charity and company limited by guarantee, registered in England (reg. no. 986729).
Registered office: Old Royal Naval College, Park Row, Greenwich, London SE10 9LS

Medway Campus

Central Avenue
Chatham Maritime
Kent ME4 4TB

Tel: +44 (0)20 8331 8000
